# Sulphamethazine derivatives as immunomodulating agents: New therapeutic strategies for inflammatory diseases

**DOI:** 10.1371/journal.pone.0208933

**Published:** 2018-12-19

**Authors:** Hina Siddiqui, Haroon M. Haniffa, Almas Jabeen, Atta-ur -Rahman, M. Iqbal Choudhary

**Affiliations:** 1 H.E.J. Research Institute of Chemistry, International Center for Chemical and Biological Sciences, University of Karachi, Karachi, Pakistan; 2 Department of Physical Sciences, Faculty of Applied Sciences, South Eastern University, Oluvil, Sri Lanka; 3 Dr. Panjwani Center for Molecular Medicine and Drug Research, International Center for Chemical and Biological Sciences, University of Karachi, Karachi, Pakistan; 4 Department of Biochemistry, Faculty of Science, King Abdulaziz University, Jeddah, Saudi Arabia; Aligarh Muslim University, INDIA

## Abstract

Sulfamethazine (SMZ) (**1**) is an antibacterial *sulfa drug* which suppresses the synthesis of dihydrofolic acid. It is used for the treatment of infections in livestock; such as gastrointestinal, and respiratory tract infections. During the current study, synthesis, characterization, and evaluation of immunomodulatory activities of derivatives of sulfamethazine (SMZ) (**3–39**) was carried out. These derivatives were synthesized by the reaction of sulfamethazine with a range of acid chlorides. All the compounds were characterized by using modern spectroscopic techniques, such as ^1^H-, and ^13^C-NMR, EI-MS, and HRFAB-MS. Compounds **3–10**, **14**, and **15** were identified as new compounds. Immunomodulatory effect of compounds **3–39** on different parameters of innate immune response was evaluated, including the production of Reactive Oxygen Species (ROS) from human whole blood and isolated polymorphonuclear neutrophils (PMNs), nitric oxide (NO), and pro-inflammatory cytokine TNF-α. All the new compounds, except **14** and **15**, showed a significant anti-inflammatory activity. Compounds **3–39** were also evaluated for their anti-bacterial activity and cytotoxicity (3T3 mouse fibroblast cell lines). All the compounds were found to be non-cytotoxic against normal cell lines.

## Introduction

Chronic inflammation is a persistent inflammatory condition which can cause tissue destruction. Macrophages are critical players in the initiation of inflammatory process, which transforms into chronic inflammation in some cases [1]. The white blood cells move in bulk, and begin to attack internal organs or other important tissues and cells [2]. Chronic inflammation is linked to a variety of ailments, including osteoarthritis, asthma, atherosclerosis, inflammatory bowel disease, psoriasis, cancers, Crohn’s disease, and ankylosing spondylitis [3].

Oxidative stress has a crucial role in the pathogenesis of chronic inflammatory diseases. During inflammation, activated phagocytes including neutrophils, monocytes and macrophages, utilize NADPH oxidase system to generate highly toxic free radicals, called oxidative burst. The deamination of L-arginine through an enzyme nitric oxide synthase (NOS) generates nitric oxide (NO) which reacts with superoxide to produces highly reactive peroxynitrite radicals. Physiological concentrations of ROS and reactive nitrogen species (RNS) have various roles, such as regulation of cellular metabolism, intracellular signaling and maintain cellular redox status, etc. ROS also defend against invading pathogens. The overexpression of these molecules during chronic inflammation is the key factor in pathogenesis of various metabolic, and autoimmune diseases [4].

Cytokines play a major role in immune system by facilitating interactions between various immune cells. TNF-α is among the key promoters of chronic inflammation, and thus play a pivotal role in the pathogenesis of inflammatory, and autoimmune diseases. It is known to enhance the production of pro-inflammatory cytokines, leukocyte adhesion molecules, prostaglandin E-2 (PGE-2), collagenase, nitric oxide (NO), etc. TNF-α inhibitor reduces the inflammation, and joint damage in rat model of inflammatory arthritis. TNF and its various superfamily members are among the most important therapeutic targets against the chronic inflammation [5].

Certain natural and synthetic drugs have been developed for the treatment of chronic inflammation and associated diseases. These drugs are divided in two groups; steroidal anti-inflammatory drugs, such as corticosteroids, and non-steroidal anti-inflammatory drugs (NSAIDs) (e.g. asprin, naproxen, and Ibuprofen) [6]. Short-term use of corticosteroids is linked with mild side effects, including cutaneous effects, electrolyte abnormalities, hypertension, hyperglycemia, and neuropsychologic effects. While, long-term use of corticosteroid is associated with more serious consequences, such as osteoporosis, aseptic joint necrosis, adrenal insufficiency, hyperlipidemia, growth suppression, and possible congenital malformations [7]. Because of the above mentioned adverse effects, there is an urgent need for developing new, safe and effective anti-inflammatory drugs.

The process of finding new uses of existing drugs is termed drug repositioning or drug repurposing, which has gained major importance in recent years. The libraries of drug molecules and rapid advances in disease biology, genomics and bioinformatics has enhanced the pace of activity-based, as well as *in silico* drug repositioning. It is a promising, fast, and cost effective method that can meet the challenges of traditional *de novo* drug discovery and development [8, 9].

Sulfonamides are well known antimicrobial agents. They have been reported to possess various biological activities such as anti-cancer, anti-angiogenesis, anti-convulsant, anti-diuretics, anti-inflammatory, hypoglycemic, carbonic anhydrase, histone deacetylase, HIV protease, and cholinestrases inhibitors.

Sulfamethazine (SMZ) is an antibacterial *sulfa drug*. It is known to inhibit the synthesis of dihydrofolic acid. Mostly this drug have been used for the treatment of various infections in livestock, such as gastrointestinal and respiratory tract infections [10]. Since the amide is an important functional group present in a number of drugs, the synthesis of amide linkages has special significance in organic synthesis, and in pharmaceuticals industries [11]. Amide derivatives of sulfamethazine have also been reported for other biological activities, such as anti-bacterial [12–15] and anti-cancer [16] properties.

A total of 37 amide derivatives of sulfamethazine were synthesized by using conventional protocols being used in synthetic chemistry. Among them, **3–10, 14**, and **15** were identified as new analogues. Interestingly all these compounds were not been reported for their anti-inflammatory activities. Present study describes the effects of test compounds **3–39** on various inflammatory mediators of innate immune responses, including effect on the production of intracellular ROS fromzymosan-activated whole blood phagocytes, and isolated PMNs, nitric oxide (NO) production from lippopolysaccharide (LPS) activated J774.2 mouse macrophages, and the production of pro-inflammatory cytokine TNF-α from THP-1 human monocytic leukemia cell line. All the compounds were also evaluated for their anti-bacterial, and cytotoxicity activities.

## Materials and methods

### General experimental conditions

Melting points were determined on a Büchi 535 melting point apparatus. IR spectra were recorded on a FTIR-8900 spectrophotometer. UV Spectra were obtained on a Hitachi UV 3200 spectrophotometer. Electron impact mass spectra (EI-MS) were recorded on a JEOL JMS-600H mass spectrometer with a MASPEC data system. FAB- and HRFAB-MS were performed on a Jeol JMS HX 110 mass spectrometer using glycerol as the matrix. The ^1^H-NMR spectra were recorded in deuterated solvents on Bruker Avance 300 and 400, MHz NMR spectrometers, while the ^13^C-NMR experiments were conducted on the same instrument at 75.45, 100, and 125 MHz respectively. The chemical shifts (*δ*) were in ppm, relative to the chemical shift of tetramethylsilane (TMS), as internal standard and coupling constants *J* in Hz. Thin-layer chromatography (TLC) analyses were performed on precoated ALUGRAM, SIL G/UV254 aluminum plates (Kieselgel 60, 20 x 20, 0.5mm thick, E. Merk, Germany). The reagents and solvents were obtained from Aldrich (St. Louis, Missouri, (USA)), E. Merck Darmstadt, (Germany), and Fluka (Buchs, Switzerland). Reagents and solvents were used without purification. Chromatograms, developed on TLC plates, were visualized under ultraviolet light at 254 nm for fluorescence quenching spots and 365 nm for florescent spots. Chromatograms, developed on TLC plates, were visualized under the ultraviolet light at 254 nm for fluorescence quenching spots, and 365 nm for florescent spots. General reaction for the synthesis of compounds **3–39** can be seen in the supporting information S39 Fig.

A solution of sulfamethazine drug (0.5 mmol, 0.14 g) in acetonitrile (3.0 mL) was stirred at room temperature for 15–20 minutes. Then the corresponding acid chloride (0.5 mmol) was added slowly, and the reaction mixture was refluxed for 3–4 hours. Progress of the reaction was monitored by TLC (Silica gel, hexane: ethyl acetate, 4: 6). After complete consumption of substrate, the reaction mixture was cooled down, and the precipitate was filtered off and washed with hexane, ethyl acetate, and acetone to remove impurities. Structures of all synthetic compounds **3–39** were characterized by different spectroscopic techniques; such as ^1^H-, and ^13^C NMR, EI-MS, FAB-MS, HR-FAB-MS, UV and IR spectroscopy.

#### 2,3-Dibromo-*N*-(4-(*N*-(4,6-dimethylpyrimidin-2-yl)sulfamoyl)phenyl)propanamide (3)

Slightly brown solid; yield: 50%; m. p. 185–187 °C; TLC (Hexane: EtOAc, 6:4 v/v): R_f_ = 0.52; IR (KBr): ν_max_ cm^-1^ 3276, 3185, 3039, 2996, 1704, 1598, 1434, 1361, 1320, 1162, 1083, 853, 585; UV/Vis (MeOH): λ_max_ nm 207, 272; ^1^H-NMR (300 MHz, DMSO-*d*_*6*_): *δ* 11.20 (1H, s, SO_2_NH), 10.90 (1H, s, CONH), 7.97 (2H, d, *J*_2′, 3′/6′, 5′_ = 8.4 Hz, H-2′/H-6′), 7.74 (2H, d, *J*_*3′*, *2′/5′*, *6*′_ = 8.4 Hz, H-3′/H-5′), 6.75 (1H, s, pyrimidine-H), 4.88 (1H, dd, *J* = 5.1 Hz, *J* = 10.2 Hz, H-2), 3.97 (2H, m, H-2a, H-2b), 2.25 (6H, s, pyrimidine-CH_3_); ^13^C-NMR (100 MHz, DMSO-*d*_*6*_): *δ* 167.2 (CONH), 165.3 (C-3", C-5"), 156.0 (C-1"), 142.1 (C-1'), 135.6 (C-4'), 129.4 (C-3', C-5'), 118.4 (C-2', C-4'), 113.4 (C-4"), 44.2 (C-2), 30.7 (C-3), 22.7 (CH_3_-3", CH_3_-5"); EI-MS *m/z* (% rel. abund.): 350.0 [M+4]^+^-SO_2_ -Br, 1.3], 348.0 [M+2]^+^-SO_2_ -Br, 65], 346.0 [M^+^-SO_2_-Br, 30], 267.1 (16), 239.1 (8), 213.2 (86), 186.0 (2.2); HR-FAB-MS (positive mode) *m/z*: [M+H]^+^calcd. for C_15_H_17_Br_2_N_4_O_3_S, 490.9388; found, 490.9388.

#### 3-(Chloromethyl)-*N*-(4-(*N*-(4,6-dimethylpyrimidin-2-yl)sulfamoyl)phenyl)benzamide (4)

White solid; yield:46%; m. p. 201–203 °C; TLC (Hexane: EtOAc, 6:4 v/v): R_f_ = 0.53; IR (KBr): ν_max_ cm^-1^ 3405, 3344, 3062, 2948, 1663, 1594, 1529, 1319, 1158,1078, 709, 582;UV/Vis (MeOH): λ_max_ nm224, 272; ^1^H-NMR (400 MHz, DMSO-*d*_*6*_): *δ* 11.46 (1H, s, SO_2_NH), 10.60 (1H, s, CONH), 7.97 (2H, d, *J*_*2′*, *3′/6′*, *5′*_ = 8.8 Hz, H-2′/H-6′), 7.92 (2H, d, *J*_*3′*,*2′/5′*, *6*′_ = 8.8 Hz, H-3′/H-5′), 7.96 (1H, d, *J*_*6*,*5*_ = 7.6 Hz, H-6), 7.94 (1H, s, H-2), 7.66 (1H, d, *J*_*4*,*5*_ = 7.6 Hz, H-4), 7.55 (1H, t, *J* = 7.6 Hz, H-5), 6.75 (1H, s, pyrimidine-H), 4.84 (2H, s, CH_2_Cl), 2.25 (6H, s, CH_3_); ^13^C-NMR (100 MHz, DMSO-*d*_*6*_): *δ* 167.5 (CONH), 161.7 (C-3", C-5"), 156.1 (C-1"), 142.6 (C-1'), 138.0 (C-3), 136.2 (C-4'), 134.8 (C-1), 132.2 (C-4), 129.0 (C-5), 128.8 (C-6), 128.1 (C-3', C-5'), 127.6 (C-2), 119.2 (C-2', C-4'), 113.5 (C-4"), 45.5 (CH_2_Cl-3), 22.9 (CH_3_-3", CH_3_-5"); FAB-MS (positive mode) *m/z* 433.0 [M+2]^+^, 431.2 [M+H]^+^; HRFAB-MS (positive mode) *m/z*: [M+H]^+^calcd. for C_20_H_20_ClN_4_O_3_S, 431.0945; found, 431.0945.

#### 4-(Chloromethyl)-*N*-(4-(*N*-(4,6-dimethylpyrimidin-2-yl)sulfamoyl)phenyl)benzamide (5)

White solid; yield: 46%; m. p. 246–248 °C; TLC (Hexane: EtOAc, 6:4 v/v): R_f_ = 0.52; IR (KBr): ν_max_ cm^-1^ 3301, 2855, 1656, 1595, 1518, 1397,1320, 1160,1083, 883, 585; UV/Vis (MeOH): λ_max_ nm 213, 273; ^1^H-NMR (400 MHz, DMSO-*d*_*6*_): *δ* 10.58 (1H, s, CONH), 7.96 (2H, d, *J*_*2′*, *3′/6′*, *5′*_ = 8.8 Hz, H-2′/H-6′), 7.92 (2H, d, *J*_*3′*, *2′/5′*, *6′*_ = 8.8 Hz, H-3′/H-5′), 7.95 (2H, d, *J*_*2*, *3/6*, *5*_ = 8.0 Hz, H-2/H-6), 7.58 (2H, d, *J*_*3*, *2/5*, *6*_ = 8.0 Hz, H-3/H-5), 6.75 (1H, s, pyrimidine-H), 4.83 (2H, s, CH_2_Cl), 3.65 (1H, s, SO_2_NH), 2.25 (6H, s, CH_3_); ^13^C-NMR (100 MHz, DMSO-*d*_*6*_): *δ* 166.4 (CONH), 165.4 (C-3", C-5"), 156.1 (C-1"), 142.7 (C-4), 141.3 (C-1'), 134.9 (C-4'), 134.2 (C-1), 129.0 (C-3', C-5'), 128.7 (C-3, C-5), 128.1 (C-2, C-6), 119.6 (C-2', C-4'), 113.4 (C-4"), 45.3 (CH_2_Cl-4), 22.5 (CH_3_-3", CH_3_-5"); EI-MS *m/z* (% rel. abund.): 368.1 [M+2]^+^-SO_2_ (64), 23], 366.1 [M^+^-SO_2_ (64), 68], 348.1 (29), 332.2 (63), 213.1 (72), 153.1 (40), 119.1 (100); HRFAB-MS (positive mode) *m/z*: [M+H]^+^calcd. for C_20_H_20_ClN_4_O_3_S, 431.0945; found, 431.0945.

#### *N*-(4-(*N*-(4,6-dimethylpyrimidin-2-yl)sulfamoyl)phenyl)-3,5-bis(trifluoromethyl)benzamide (6)

White solid; yield: 53%; m. p. 257–259 °C; TLC (Hexane: EtOAc, 6:4 v/v): R_f_ = 0.53; IR (KBr): ν_max_ cm^-1^ 3419, 3312, 2925, 1684, 1598, 1539, 1281, 1136, 676, 585; UV/Vis (MeOH): λ_max_ nm 225, 277; ^1^H-NMR (400 MHz, DMSO-*d*_*6*_): *δ* 11.44 (1H, s, SO_2_NH), 10.90 (1H, s, CONH), 8.58 (2H, s, H-2/H-6), 8.38 (1H, s, H-4), 8.01 (2H, d, *J*_*2′*, *3′/6′*, *5′*_ = 8.4 Hz, H-2′/H-6′), 7.92 (2H, d, *J*_*3′*, *2′/5′*, *6′*_ = 8.4 Hz, H-3′/H-5′), 6.75 (1H, s, pyrimidine-H), 2.25 (6H, s, CH_3_); ^13^C NMR (100 MHz, DMSO-*d*_*6*_): *δ* 167.4 (CONH), 162.9 (C-3", C-5"), 156.1 (C-1"), 142.0 (C-1'), 136.7 (C-1), 135.4 (C-4'), 130.2 (C-3, C-5), 129.1 (C-3', C-5'), 128.6 (C-2, C-6), 125.3 (C-4), 124.3 (CF_3_-4), 119.5 (C-2', C-4'), 113.6 (C-4"), 22.8 (CH_3_-3", CH_3_-5"); EI-MS *m/z* (% rel. abund.): 455.2 (14), 454.2 [M^+^-SO_2_ (64), 70], 453.2 (100), 365.1 (2), 332.1 (3), 241.1 (33), 213.1 (55); HRFAB-MS (positive mode) *m/z*: [M+H]^+^calcd. for C_21_H_17_F_6_N_4_O_3_S, 519.0926; found, 519.0926.

#### *N*-(4-(*N*-(4,6-dimethylpyrimidin-2-yl)sulfamoyl)phenyl)-3,5-difluorobenzamide (7)

White solid; yield: 62%; m. p. 237–240 °C; TLC (Hexane: EtOAc, 8:2 v/v): R_f_ = 0.58; IR (KBr): ν_max_ cm^-1^ 3387, 3311, 3122, 2924, 1673, 1596, 1532, 1326, 1159, 589; UV/Vis (MeOH): λ_max_ nm 208, 277; ^1^H-NMR (400 MHz, DMSO-*d*_*6*_): *δ* 10.66 (1H, s, CONH), 7.97 (2H, d, *J*_*2′*, *3′/6′*, *5′*_ = 8.8 Hz, H-2′/H-6′), 7.91 (2H, d, *J*_*3′*, *2′/5′*, *6′*_ = 8.8 Hz, H-3′/H-5′), 7.68 (2H, d, *J*_*2*_,_*3F/6*_,_*5F*_ = 6.4 Hz, H-2/ H-6), 7.53 (1H, t, *J*_*3F*_,_*5F*_ = 6.4 Hz, H-4), 6.75 (1H, s, pyrimidine-H), 3.66 (1H, s, SO_2_NH), 2.24 (6H, s, CH_3_); ^13^C-NMR (100 MHz, DMSO-*d*_*6*_): *δ* 167.2 (CONH), 163.2 (C-3", C-5"), 160.8 (C-3, C-5), 156.1 (C-1"), 142.2 (C-1'), 137.7 (C-1), 135.4 (C-4'), 129.0 (C-3', C-5'), 119.4 (C-2', C-4'), 113.4 (C-4"), 111.4 (C-2, C-6), 107.2 (C-4), 22.7 (CH_3_-3", CH_3_-5"); EI-MS *m/z* (% rel. abund.): 355.1 (20), 354.1 (95), 353.1 [M^+^-SO_2_ (64), 100], 213.1 (66), 141.1 (33), 113.1 (18); HRFAB-MS (positive mode) *m/z*: [M+H]^+^calcd. for C_19_H_17_F_2_N_4_O_3_S, 419.0989; found, 419.0989.

#### *N*-(4-(*N*-(4,6-dimethylpyrimidin-2-yl)sulfamoyl)phenyl)-3-fluoro-5-(trifluoromethyl)benzamide (8)

White solid; yield: 50%; m. p. 210–212 °C; TLC (Hexane: EtOAc, 6:4 v/v): R_f_ = 0.48; IR (KBr): ν_max_ cm^-1^ 3386, 3306, 3200, 2924, 1633, 1594, 1528, 1358,1315, 1166, 1132, 836, 574; UV/Vis (MeOH): λ_max_ nm 207, 282; ^1^H-NMR (400 MHz, DMSO-*d*_*6*_): *δ* 10.81 (1H, s, CONH), 8.16 (1H, s, H-6), 8.12 (1H, *J*_*2*_, _*3F*_ = 9.2 Hz, H-2), 7.99 (2H, d, *J*_*2′*, *3′/6′*, *5′*_ = 8.8 Hz, H-2′/H-6′), 7.97 (H, d, *J*_*4*_, _*3F*_ = 7.6 Hz, H-4), 7.92 (2H, d, *J*_*3′*, *2′/5′*, *6′*_ = 8.8 Hz, H-3′/H-5′), 6.75 (1H, s, pyrimidine-H), 3.81 (1H, s, SO_2_NH), 2.24 (6H, s, CH_3_); ^13^C-NMR (100 MHz, DMSO-*d*_*6*_): *δ* 163.1 (CONH), 163.0 (C-3", C-5"), 160.5 (C-3), 156.1 (C-1"), 142.2 (C-1'), 137.9 (C-1), 135.6 (C-4'), 130.9 (C-5), 129.0 (C-3', C-5'), 123.1 (CF_3_-5), 120.7 (C-6), 119.4 (C-2', C-4'), 119.1 (C-2), 116.1 (C-4), 113.5 (C-4"), 22.7 (CH_3_-3", CH_3_-5"); EI-MS *m/z* (% rel. abund.): 406.2 (8), 405.2 (63), 404.1 (98), 403.2 [M^+^-SO_2_ (64), 100], 385.2 (2), 282.1 (9), 213.1 (96), 191.1 (86), 163.1 (73); HRFAB-MS (positive mode) *m/z*: [M+H]^+^ calcd. for C_20_H_17_F_4_N_4_O_3_S, 469.0957; found, 469.0957.

#### N-(4-(*N*-(4,6-dimethylpyrimidin-2-yl)sulfamoyl)phenyl)-3-fluoro-4-(trifluoromethyl)benzamide(9)

White solid; yield: 89%; m. p. 204–206 °C; TLC (Hexane: EtOAc, 6:4 v/v): R_f_ = 0.56; IR (KBr):ν_max_ cm^-1^ 3405, 3129, 2949, 1685, 1593, 1530, 1313, 1315, 1170, 1134, 839, 579;UV/Vis (MeOH): λ_max_ nm 209, 274; ^1^H-NMR (400 MHz, DMSO-*d*_*6*_): *δ* 10.83 (1H, s, CONH), 8.06 (1H, d, *J*_*5*_,_*6*_ = 11.6 Hz, H-5), 7.98 (2H, d, *J*_*2′*, *3′/6′*, *5′*_ = 8.8 Hz, H-2′/H-6′), 7.97 (2H, m, H-2, H-6), 7.93 (2H, d, *J*_*3′*, *2′/5′*, *6′*_ = 8.4 Hz, H-3′/H-5′), 6.75 (1H, s, pyrimidine-H), 4.10 (1H, s, SO_2_NH), 2.25 (6H, s, CH_3_); ^13^C-NMR (100 MHz, DMSO-*d*_*6*_): *δ* 167.3 (CONH), 163.3 (C-3", C-5"), 159.8 (C-3), 156.1 (C-1"), 142.1 (C-1'), 140.1 (C-1), 135.5 (C-4'), 129.0 (C-3', C-5'), 127.7 (C-5), 124.4 (C-6), 123.6 (CF_3_-4), 120.9 (C-4), 116.4 (C-2), 119.4 (C-2', C-4'), 113.4 (C-4"), 22.7 (CH_3_-3", CH_3_-5"); EI-MS *m/z* (% rel. abund.): 406.4 (1.), 405.4 (16.4), 404.1 (72.1), 403.2 [M^+^-SO_2_ (64), 100], 333.2 (2), 282.1 (3), 213.1 (68), 191.1 (37), 163.1 (33); HR-FAB-MS (positive mode) *m/z*: [M+H]^+^calcd. for C_20_H_17_F_4_N_4_O_3_S, 469.0957; found, 469.0957.

#### *N*-(4-(*N*-(4,6-dimethylpyrimidin-2-yl)sulfamoyl)phenyl)-4-fluoro-3-(trifluoromethyl)benzamide (10)

White solid; yield: 64%; m. p. 218–220 °C; TLC (Hexane: EtOAc, 6:4 v/v): R_f_ = 0.50; IR (KBr): ν_max_ cm^-1^, 3316, 3097, 2931, 1692, 1632, 1591, 1498, 1313, 1164,1135, 836, 581; UV/Vis (MeOH): λ_max_ nm 207, 274; ^1^H-NMR (400 MHz, DMSO-*d*_*6*_): *δ* 10.71 (1H, s, CONH), 8.35 (1H, *J*_*6*,*5*_ = 6.0 Hz, H-6), 8.33 (1H, s, H-2), 7.98 (2H, d, *J*_*2′*, *3′/6′*, *5′*_ = 8.8 Hz, H-2′/H-6′), 7.91 (2H, d, *J*_*3′*, *2′/5′*, *6′*_ = 8.8 Hz, H-3′/H-5′), 7.35 (1H, *J*_*5*,*6*_ = 9.2 Hz, H-5), 6.75 (1H, s, pyrimidine-H), 3.66 (1H, s, SO_2_NH), 2.24 (6H, s, CH_3_); ^13^C-NMR (100 MHz, DMSO-*d*_*6*_): *δ* 167.3 (CONH), 163.4 (C-3", C-5"), 162.0 (C-4), 156.1 (C-1"), 142.3 (C-1'), 135.3 (C-4'), 135.2 (C-6), 130.1 (C-1), 129.0 (C-3', C-5'), 127.6 (C-2), 123.6 (CF_3_-3), 119.4 (C-2', C-4'), 117.4 (C-5), 113.4 (C-4"), 22.7 (CH_3_-3", CH_3_-5"); EI-MS *m/z* (% rel. abund.): 406.2 (8), 405.3 (54), 404.2 (97), 403.2 [M^+^-SO_2_ (64), 100], 335.3 (3), 282.1 (7), 213.1 (88), 191.1 (83), 163.1 (53); HRFAB-MS (positive mode) *m/z*: [M+H]^+^calcd. for C_20_H_17_F_4_N_4_O_3_S, 469.0957; found, 469.0957.

#### 2-Chloro-*N*-(4-(*N*-(4,6-dimethylpyrimidin-2-yl)sulfamoyl)phenyl)propanamide (11)

White solid; yield:55%;m. p. 256–258 °C; TLC (Hexane: EtOAc, 6:4 v/v): R_f_ = 0.53; IR (KBr): ν_max_ cm^-1^3276, 3188, 3047, 2997, 1706, 1600, 1544, 1430, 1350, 1161,1081, 855, 583;UV/Vis (MeOH): λ_max_ nm212, 261; ^1^H-NMR (400 MHz, DMSO-*d*_*6*_): *δ* 11.38 (1H, s, SO_2_NH), 10.63 (1H, s, CONH), 7.94 (2H, d, *J*_*3′*, *2′/5′*, *6′*_ = 8.4 Hz, H-3′/H-5′), 7.73 (2H, d, *J*_*2′*, *3′/6′*, *5′*_ = 8.4 Hz, H-2′/H-6′), 6.75 (1H, s, pyrimidine-H), 4.66 (1H, q, *J* = 6.8 Hz, H-2), 2.25 (6H, s, pyrimidine-CH_3_). 1.59(1H, d, *J* = 6.8 Hz, CH_3_-2); FAB-MS (positive mode) *m/z* 371.2 [M + H + 2]^+^, 369.1 [M+ H]^+^.

#### *N*-(4-(*N*-(4,6-dimethylpyrimidin-2-yl)sulfamoyl)phenyl)-4-fluorobenzamide (12)

White solid; yield:50%; m. p. 190–192 °C; TLC (Hexane: EtOAc, 8:2 v/v): R_f_ = 0.62; IR (KBr): ν_max_ cm^-1^ 3453, 3243, 3066, 2928, 1625, 1531, 1502, 1317,1160, 851, a1572; UV/Vis (MeOH): λ_max_ nm 213, 274; ^1^H-NMR (300 MHz, DMSO-*d*_*6*_): *δ*10.59 (1H, s, CONH), 8.04 (2H, dd, *J*_*2*, *(3*,*4F)/6*, *(5*, *4F)*_ = 9.0 Hz, 2.1 Hz, H-2/H-6), 7.96 (2H, d, *J*_*2′*, *3′/6′*, *5′*_ = 9.0 Hz, H-2′/H-6′), 7.91 (2H, d, *J*_*3′*, *2′/5′*, *6′*_ = 9.0 Hz, H-3′/H-5′), 7.36 (2H, t, *J*_*3*, *(2*,*4F)/5*, *(6*, *4F)*_ = 9.0 Hz, H-3/H-5), 6.75 (1H, s, pyrimidine-H), 3.99 (1H, s, SO_2_NH), 2.24 (6H, s, CH_3_); EI-MS *m/z* (% rel. abund.): 338.3 (7), 337.3 (96), 336.2 [M^+^-SO_2_ (64), 100], 335.2 (96), 213.2 (86), 185.1 (3), 123.0 (100), 95.0 (55).

#### *N*-(4-(*N*-(4,6-dimethylpyrimidin-2-yl)sulfamoyl)phenyl)-3-nitrobenzamide (13)

Slightly yellow solid; yield:80%; m. p. 251–253 °C; TLC (Hexane:EtOAc, 8:2 v/v): R_f_ = 0.54;IR (KBr): ν_max_ cm^-1^3381, 3072, 2925, 1668, 1592, 1524, 1348, 1319, 1157, 1084; UV/Vis (MeOH): λ_max_ nm 216, 272; ^1^H-NMR (400 MHz, DMSO-*d*_*6*_): *δ* 11.52 (s, 1H, SO_2_NH), 10.85 (1H, s, CONH), 8.78 (1H, s, H-2), 8.44(1H, d, *J*_*4*_, _*5*_ = 8.4 Hz, H-4), 8.38 (1H, d, *J*_*6*_,_*5*_ = 8.0 Hz, H-6), 7.99 (2H, d, *J*_2′, 3′/6′, 5′_ = 8.0 Hz, H-2′/H-6′), 7.93 (2H, d, *J*_3′, 2′/5′, 6′_ = 8.0 Hz, H-3′/H-5′),7.84 (1H, t, *J* = 8.0 Hz, H-5), 6.75 (1H, s, pyrimidine-H), 2.25 (6H, s, CH_3_), FAB-MS (positive mode) *m/z* 428 [M+H]^+^.

#### *N*-(4-(*N*-(4,6-dimethylpyrimidin-2-yl)sulfamoyl)phenyl)-2-fluoro-3-(trifluoromethyl)benzamide (14)

White solid; yield:57%; m. p. 215–217 °C; TLC (Hexane: EtOAc, 6:4 v/v): R_f_ = 0.53; IR (KBr): ν_max_ cm^-1^ 3405, 3129, 2949, 1685, 1593, 1530, 1313, 1315, 1170, 1134, 839, 579; UV/Vis (MeOH): λ_max_ nm 211, 272; ^1^H-NMR (400 MHz, DMSO-*d*_*6*_): *δ*, 11.47 (1H, s, SO_2_NH), 10.95 (1H, s, CONH), 8.01 (1H, d, *J*_6, 5_ = 6.4 Hz, H-6), 7.98 (2H, d, *J*_*2′*, *3′/6′*, *5′*_ = 8.4 Hz, H-2′/H-6′), 7.93 (1H, d, *J*_*4*,*5*_ = 6.4 Hz, H-4), 7.84 (2H, d, *J*_*3′*, *2′/5′*, *6′*_ = 8.4 Hz, H-3′/H-5′), 7.54 (1H, t, *J* = 7.6 Hz, H-5), 6.75 (1H, s, pyrimidine-H), 2.25 (6H, s, CH_3_);); ^13^C-NMR (100 MHz, DMSO-*d*_*6*_): *δ* 167.5 (CONH), 161.7 (C-3", C-5"), 157.0 (C-2), 156.1 (C-1"), 141.9 (C-1'), 135.5 (C-4'), 134.6 (C-4), 129.4 (C-6), 129.2 (C-3', C-5'), 126.2 (C-1), 125.2 (C-5), 123.7 (CF_3_-3), 121.0 (C-3), 118.8 (C-2', C-4'), 113.5 (C-4"), 22.7 (CH_3_-3", CH_3_-5"); EI-MS *m/z* (% rel. abund.): 406.2 (2), 405.3 (21), 404.2 (94), 403.2 [M^+^-SO_2_(64), 100], 282.1(4), 213.1(51), 191.1(50), 163.1(15); HR-FAB-MS (positive mode) *m/z*: [M+H]^+^calcd. for C_20_H_17_F_4_N_4_O_3_S, 469.0957; found, 469.0957.

#### *N*-(4-(*N*-(4,6-dimethylpyrimidin-2-yl)sulfamoyl)phenyl)-4,7,7-trimethyl-3-oxo-2-oxabicyclo[2.2.1]heptane-1-carboxamide (15)

White solid; yield:50%;m. p. 254–256 °C; TLC (Hexane: EtOAc, 6:4 v/v): R_f_ = 0.48; IR (KBr): ν_max_ cm^-1^3368, 3234, 2967, 1791, 1692, 1597, 1528, 1434, 1321, 1159,1095, 875, 583; UV/Vis (MeOH): λ_max_ nm211, 261; ^1^H-NMR (400 MHz, DMSO-*d*_*6*_): *δ* 11.55 (1H, s, SO_2_NH),10.13 (1H, s, CONH), 7.93 (2H, d, *J*_*3′*, *2′/5′*, *6′*_ = 8.8 Hz, H-3′/H-5′), 7.87 (2H, d, *J*_*2′*, *3′/6′*, *5′*_ = 8.8 Hz, H-2′/H-6′), 6.75 (1H, s, pyrimidine-H), 2.45 (1H, m, H-6a), 2.25 (6H, s, pyrimidine-CH_3_), 1.98 (1H, m, H-6b), 1.97 (1H, m, H-5a), 1.58 (1H, m, H-5b), 1.02 (6H, s, CH_3_-7), 0.88 (3H, s, CH_3_-4); ^13^C-NMR (100 MHz, DMSO-*d*_*6*_): *δ* 177.7 (COO), 167.1 (CONH), 165.8 (C-3", C-5"), 156.1 (C-1"), 141.3 (C-1'), 135.6 (C-4'), 128.8 (C-3', C-5'), 120.0 (C-2', C-4'), 113.5 C-4"), 91.0 (C-2), 54.5 (C-4), 53.6 (C-7), 30.0 (C-6), 28.3 (C-5), 22.9 (CH_3_-3", CH_3_-5"), 16.4 (CH_3_-7), 16.2 (CH_3_-7), 9.4 (CH_3_-4); FAB-MS (positive mode) *m/z* 459.1 [M+H]^+^; HRFAB-MS (positive mode) *m/z*: [M+H]^+^calcd. for C_22_H_27_N_4_O_5_S, 459.1702; found, 459.1702.

#### *N*-(4-(*N*-(4,6-dimethylpyrimidin-2-yl)sulfamoyl)phenyl)-3-fluorobenzamide (16)

White solid; yield: 35%; m. p. 236–238 °C; TLC (Hexane: EtOAc, 8:2 v/v): R_f_ = 0.48; IR (KBr): ν_max_ cm^-1^ 3383, 3121, 2924, 1671, 1591, 1529, 1467, 1432, 1320, 1160, 560; UV/Vis (MeOH): λ_max_ nm 207, 278; ^1^H-NMR (400 MHz, DMSO-*d*_*6*_): *δ* 11.39 (1H, s,SO_2_NH), 10.58 (1H, s, CONH), 7.97 (2H, d, *J*_*2′*, *3′/6′*, *5′*_ = 8.4 Hz, H-2′/H-6′), 7.91 (2H, d, *J*_*3′*, *2′/5′*, *6′*_ = 8.4 Hz, H-3′/H-5′), 7.79 (1H, d, *J*_*6*,*5*_ = 7.6 Hz, H-6), 7.74 (1H, s, H-2), 7.58 (1H, q, *J* = 8.0 Hz, H-4), 7.46 (1H, t, *J* = 8.4 Hz, H-5), 6.75 (1H, s, pyrimidine-H), 2.25 (6H, s, CH_3_); EI-MS *m/z* (% rel. abund.): 338.3 (3), 337.2 (20), 336.1 [M^+^-SO_2_ (64), 98], 335.1 (100), 213.1(65), 185.1(2), 123.0 (50).

#### *N*-(4-(*N*-(4,6-dimethylpyrimidin-2-yl)sulfamoyl)phenyl)-2-fluorobenzamide (17)

White solid; yield:65%; m. p. 233–235 °C; TLC (Hexane: EtOAc, 6:4 v/v): R_f_ = 0.61; IR (KBr): ν_max_ cm^-1^ 3405, 3129, 2949, 1685, 1593, 1530, 1313,1315, 1170, 1134, 839, 579; UV/Vis (MeOH): λ_max_ nm 212, 278; ^1^H-NMR (400 MHz, DMSO-*d*_*6*_): *δ* 11.41 (1H, s, SO_2_NH), 10.74 (1H, s, CONH), 7.96 (2H, d, *J*_*2′*, *3′/6′*, *5′*_ = 8.4 Hz, H-2′/H-6′), 7.85 (2H, d, *J*_*3′*, *2′/5′*, *6′*_ = 8.4 Hz, H-3′/H-5′), 7.60 (1H, t, *J* = 7.2 Hz, H-5), 7.57 (1H, q, *J* = 7.2 Hz, H-3), 7.35 (1H, t, *J* = 8.0 Hz, H-4), 7.31 (1H, t, *J* = 7.2 Hz, H-5), 6.75 (1H, s, pyrimidine-H), 2.25 (6H, s, CH_3_); ^13^C-NMR (100 MHz, DMSO-*d*_*6*_): *δ* 167.9 (CONH), 163.1 (C-3", C-5"), 157.6 (C-2), 156.1 (C-1"), 142.3 (C-1'), 135.2 (C-4'), 132.7 (C-4), 129.8 (C-5), 129.2 (C-3', C-5'),), 124.4 (C-1), 124.5 (C-6), 118.7 (C-2', C-4'), 116.0 (C-3), 113.5 (C-4"), 22.8 (CH_3_-3", CH_3_-5") EI-MS *m/z* (% rel. abund.): 338.3 (3), 337.2 (20), 336.3 [M^+^-SO_2_ (64), 100], 335.1 (95), 213.1(70), 123.0 (83), 95.0 (22); HRFAB-MS (positive mode) *m/z*: [M+H]^+^calcd. for C_19_H_18_FN_4_O_3_S, 401.1084; found, 401.1084.

#### *N*-(4-(*N*-(4,6-dimethylpyrimidin-2-yl)sulfamoyl)phenyl)-3,4-difluorobenzamide (18)

Slightly yellow solid; yield:74%; TLC (Hexane: EtOAc, 8:2 v/v): R_f_ = 0.53; m.p. 212–218 °C; IR (KBr): ν_max_ cm^-1^ 3245, 3062, 2926, 1672, 1624, 1513, 1316, 1284, 1159, 843, 579; UV/Vis (MeOH): λ_max_ nm 207, 277;^1^H-NMR (300 MHz, DMSO-*d*_*6*_): *δ* 10.68 (1H, s, CONH), 8.06 (1H, m, H-6), 7.97 (2H, d, *J*_*2′*, *3′/6′*, *5′*_ = 8.8 Hz, H-2′/H-6′), 7.93 (1H, d, *J* = 7.2 Hz, H-6), 7.92 (2H, d, *J*_*3′*, *2′/5′*, *6′*_ = 8.8 Hz, H-3′/H-5′), 7.86 (1H, m, H-2), 7.61 (1H, m, H-5), 6.74 (1H, s, pyrimidine-H), 4.96 (1H, s, SO_2_NH), 2.24 (6H, s, CH_3_); EI-MS *m/z* (% rel. abund.): 355.2 (17), 356.2 (2.6), 354.1 [M^+^-SO_2_(64), 95], 353.1 (100), 232.1(30), 213.2 (65), 141.0 (53), 113.0 (20).

#### *N*-(4-(*N*-(4,6-dimethylpyrimidin-2-yl)sulfamoyl)phenyl)-4-(trifluoromethyl)benzamide (19)

White solid; yield: 64%; m. p. 225–228 °C; TLC (Hexane: EtOAc, 6:4 v/v): R_f_ = 0.64; IR (KBr): ν_max_ cm^-1^ 3444, 3239, 2925, 1626, 1591, 1528, 1319, 1167, 1065, 835, 580; UV/Vis (MeOH): λ_max_ nm 223, 273; ^1^H-NMR (400 MHz, DMSO-*d*_*6*_*)*: *δ* 10.78 (1H, s, CONH), 7.98 (2H, d, *J*_*2′*, *3′/6′*, *5′*_ = 8.8 Hz, H-2′/H-6′), 7.91 (2H, d, *J*_*3′*, *2′/5′*, *6′*_ = 8.8 Hz, H-3′/H-5′), 8.14 (2H, d, *J*_*2*, *3/6*, *5*_ = 8.0 Hz, H-2/H-6), 7.93 (2H, d, *J*_*3*, *2/5*, *6*_ = 8.0 Hz, H-3/H-5), 6.75 (1H, s, pyrimidine-H), 3.59 (1H, s, SO_2_NH), 2.25 (6H, s, CH_3_); EI-MS *m/z* (% rel. abund.): 387.3 (19), 386.2 [M^+^-SO_2_ (64), 97], 385.3 (100), 297.2 (4), 264.1 (3), 213.1(63), 173.1 (42), 145.1 (31).

#### *N*-(4-(*N*-(4,6-dimethylpyrimidin-2-yl)sulfamoyl)phenyl)-3- trifluoromethyl)benzamide (20)

White solid; yield:65%;m. p. 237–239 °C; TLC (Hexane: EtOAc, 6:4 v/v): R_f_ = 0.63; IR (KBr): ν_max_ cm^-1^3386, 3115, 2921, 1669, 1593, 1532, 1314,1260, 1165,1124, 694, 558;UV/Vis (MeOH): λ_max_ nm 211, 277; ^1^H-NMR (400 MHz, DMSO-*d*_*6*_): *δ*11.51 (1H, s, SO_2_NH), 10.74 (1H, s, CONH), 8.28 (1H, s, H-2), 8.24 (1H, d, *J*_*6*,*5*_ = 8.0 Hz, H-6), 7.99 (2H, d, *J*_*2′*, *3′/6′*, *5′*_ = 8.8 Hz, H-2′/H-6′), 7.96 (H, d, *J*_*4*,*5*_ = 8.0 Hz, H-4), 7.92 (2H, d, *J*_*3′*, *2′/5′*, *6′*_ = 8.8 Hz, H-3′/H-5′, 7.78 (H, t, *J* = 7.6 Hz, H-5), 6.75 (1H, s, pyrimidine-H), 2.25 (6H, s, CH_3_); EI-MS *m/z* (% rel. abund.): 387.2 (20.5), 386.2 [M^+^-SO_2_, 88.4], 385.2 (100), 297.2 (2), 264.1 (3), 213.1(57), 173.1(45), 145.0(29).

#### 3-chloro-*N*-(4-(*N*-(4,6-dimethylpyrimidin-2-yl)sulfamoyl)phenyl)benzamide (21)

White solid; yield:65%; m. p. 237–246 °C; TLC (Hexane: EtOAc, 8:2 v/v): R_f_ = 0.48; IR (KBr): ν_max_ cm^-1^ 3384, 3063, 2927, 1665, 1592, 1521, 1316, 113, 959, 552; UV/Vis (MeOH): λ_max_ nm 212, 278; ^1^H-NMR (400 MHz, DMSO-*d*_*6*_): *δ* 11.44 (1H, s, SO_2_NH), 10.62 (1H, s, CONH), 7.99 (1H, s, H-2), 7.97 (2H, d, *J*_*2′*, *3′/6′*, *5′*_ = 8.0 Hz, H-2′/H-6′), 7.89 (1H, d, *J*_*6*,*5*_ = 6.8 Hz, H-6), 7.87 (2H, d, *J*_*3′*, *2′/5′*, *6′*_ = 8.0 Hz, H-3′/H-5′), 7.67 (1H, d, *J*_*4*,*5*_ = 6.8 Hz, H-4), 7.56 (1H, t, *J* = 8.0 Hz, H-5), 6.75 (1H, s, pyrimidine-H), 2.25 (6H, s, CH_3_); EI-MS *m/z* (% rel. abund.): 354.0 [M + 2]^+^-SO_2_ (64), 37], 353.0 (52), 352.0 [M^+^-SO_2_ (64), 100], 351.0 (88), 230.0 (3), 213.1 (73), 139.0 (43), 111.0 (21).

#### 4-chloro-*N*-(4-(*N*-(4,6-dimethylpyrimidin-2-yl)sulfamoyl)phenyl)benzamide (22)

White solid yield: 94%; m. p. 223–226 °C; TLC (Hexane: EtOAc, 8:2 v/v): R_f_ = 0.66;IR (KBr): ν_max_ cm^-1^ 3308, 3200, 2924, 1658, 1595, 1520, 1312, 1159, 581; UV/Vis (MeOH): λ_max_ nm 208, 276; ^1^H-NMR (400 MHz, DMSO-*d*_*6*_): *δ* 11.40 (s, 1H, SO_2_NH), 10.59 (1H, s, CONH), 7.97 (2H, d, *J*_*2′*,*3′/6′*,*5′*_ = 8.4 Hz, H-2′/H-6′), 7.97 (2H, d, *J*_*2*, *3/6*, *5*_ = 8.4 Hz, H-2/H-6), 7.91 (2H, d, *J*_*3′*, *2′/5′*, *6′*_ = 8.4 Hz, H-3′/H-5′), 7.61 (2H, d, *J*_*3*,*2/5*,*6*_ = 8.4 Hz, H-3/H-5), 6.75 (1H, s, pyrimidine-H), 2.24 (6H, s, CH_3_); EI-MS *m/z* (% rel. abund.): 354.0 [M+2]^+^-SO_2_ (64), 34], 353.0 (4), 352.0 [M^+^-SO_2_ (64), 100], 351.0 (86), 230.0 (3), 213.0 (71), 139.0 (78), 111.0 (24).

#### 2,5-dichloro-*N*-(4-(*N*-(4,6-dimethylpyrimidin-2-yl)sulfamoyl)phenyl)benzamide (23)

White solid; yield: 88%; m. p. 230–232 °C; TLC (Hexane: EtOAc, 6:4 v/v): R_f_ = 0.53; IR (KBr): ν_max_ cm^-1^ 3239, 3061, 2966, 1657, 1598, 1540, 1399, 1263, 138, 1080, 830, 585; UV/Vis (MeOH): λ_max_ nm 210, 228, 268; ^1^H-NMR (400 MHz, DMSO-*d*_*6*_*)*: *δ* 11.43 (1H, s, SO_2_NH), 10.90 (1H, s, CONH), 7.97 (2H, d, *J*_*2′*, *3′/6′*, *5′*_ = 8.4 Hz, H-2′/H-6′), 7.82 (2H, d, *J*_*3′*, *2′/5′*, *6′*_ = 8.4 Hz, H-3′/H-5′), 7.75 (1H, s, H-6), 7.59 (2H, s, H-3, H-4), 6.75 (1H, s, pyrimidine-H), 2.25 (6H, s, CH_3_); EI-MS *m/z* (% rel. abund.): 390.1[M + 4]^+^-SO_2_ (64), 10], 389.1 (22), 388.1[M+2]^+^-SO_2_ (64), 59], 387.0 (74), 386.1[M^+^-SO_2_ (64), 99], 385.1 (86), 213.1 (100), 173.0 (56), 145.0 (22).

#### 2,4-dichloro-*N*-(4-(*N*-(4,6-dimethylpyrimidin-2-yl)sulfamoyl)phenyl)benzamide (24)

White solid; yield:62%; m. p. 233–236 °C; TLC (Hexane: EtOAc, 8:2 v/v): R_f_ = 0.43; IR (KBr):ν_max_ cm^-1^3600, 3064, 2923, 1660, 1592, 1540, 1140, 849, 706, 585; UV/Vis (MeOH): λ_max_ nm213, 272; ^1^H-NMR (400 MHz, DMSO-*d*_*6*_): *δ* 11.49 (1H, s, SO_2_NH), 10.87 (1H, s, CONH), 7.97 (2H, d,*J*_*2′*, *3′/6′*, *5′*_ = 8.4 Hz, H-2′/H-6′), 7.82 (2H, d, *J*_*3′*, *2′/5′*, *6′*_ = 8.4 Hz, H-3′/H-5′), 7.76 (1H, s, H-3), 7.64 (1H, d, *J*_*5*,*6*_ = 8.0 Hz, H-6), 7.55 (1H, dd, *J* = 8.0 Hz, *J* = 1.6 Hz, H-5), 6.75 (1H, s, pyrimidine-H), 2.25 (6H, s, CH_3_); FAB-MS (positive mode) *m/z* 455.0 [M+H +4]^+^, 453.0 [M+H+2]^+^, 451.1 [M+H]^+^.

#### *N*-(4-(*N*-(4,6-dimethylpyrimidin-2-yl)sulfamoyl)phenyl)-4-nitrobenzamide (25)

Slightly yellow solid; yield: 51%; m. p. 237–239 °C; TLC (Hexane: EtOAc, 8:2 v/v): R_f_ = 0.42; IR (KBr): ν_max_ cm^-1^ 3300, 3079, 2924, 1632, 1593, 1528, 1342, 1317, 1164, 842; UV/Vis (MeOH): λ_max_ nm 207, 258; ^1^H-NMR (400 MHz, DMSO-*d*_*6*_): *δ* 10.87 (1H, s, CONH), 8.36 (2H, d, *J*_*3*, *2/5*, *6*_ = 8.8 Hz, H-3/H-5), 8.17 (2H, d, *J*_*2*, *3/6*, *5*_ = 8.8 Hz, H-2/H-6), 7.95 (2H, d, *J*_*2′*, *3′/6′*, *5′*_ = 8.8 Hz, H-2′/H-6′), 7.93 (2H, d, *J*_*3′*, *2′/5′*, *6′*_ = 8.8 Hz, H-3′/H-5′), 6.75 (1H, s, pyrimidine-H), 3.50 (s, 1H, SO_2_NH), 2.25 (6H, s, CH_3_); EI-MS *m/z* (% rel. abund.): 364.2 (22), 363.1 [M^+^-SO_2_ (64), 93], 362.1 (100), 333.2 (8), 316.1 (9), 213.1 (59), 150.0 (20).

#### *N*-(4-(*N*-(4,6-dimethylpyrimidin-2-yl)sulfamoyl)phenyl)-3,5-dinitrobenzamide (26)

Slightly yellow solid; yield: 26% m. p. 265–267 °C; TLC (Hexane: EtOAc, 8:2 v/v): R_f_ = 0.64; IR (KBr):ν_max_ cm^-1^3392, 3121, 2925, 1686, 1598, 1542, 1342, 1155;UV/Vis (MeOH): λ_max_ nm2 12, 252; ^1^H-NMR (300 MHz, DMSO-*d*_*6*_): *δ* 11.72 (s, 1H, SO_2_NH), 11.10 (1H, s, CONH), 9.15 (2H, d, *J*_*5*,*3*_ = 2.1 Hz, H-2), 9.00 (1H, t, *J* = 2.1 Hz, H-3), 8.01 (2H, d, *J*_*2′*, *3′/6′*, *5′*_ = 8.7 Hz, H-2′/H-6′), 7.94 (2H, d, *J*_*3′*, *2′/5′*, *6′*_ = 9.0 Hz, H-3′/H-5′), 6.74 (1H, s, pyrimidine-H), 2.24 (6H, s, CH_3_); EI-MS *m/z* (% rel. abund.): 409.2 (20), 408.2 [M^+^-SO_2_ (64), 86], 407.1 (100), 378.2 (22), 239.1 (30), 213.2 (84), 195.1 (22).

#### 2-chloro-*N*-(4-(*N*-(4,6-dimethylpyrimidin-2-yl)sulfamoyl)phenyl)-4-nitrobenzamide (27)

White solid; yield: 47%; m. p. 216–219 °C; TLC (Hexane: EtOAc, 6:4 v/v): R_f_ = 0.48; IR (KBr): ν_max_ cm^-1^ 3258, 3104, 2927, 1700, 1594, 1532, 1323, 1157, 584; UV/Vis (MeOH): λ_max_ nm 214, 261; ^1^H-NMR (300 MHz, DMSO-*d*_*6*_): *δ* 11.67 (s, 1H, SO_2_NH), 11.08 (1H, s, CONH), 8.41 (1H, d, *J*_*3*,*5*_ = 2.0 Hz, H-3), 8.29 (1H, dd, *J* = 8.4 Hz, *J* = 1.6 Hz, H-5), 7.99 (2H, d, *J*_*2′*, *3′/6′*, *5′*_ = 8.4 Hz, H-2′/H-6′), 7.91 (1H, d, *J*_*6*,*5*_ = 8.4.0 Hz, H-6), 7.83 (2H, d, *J*_*3′*, *2′/5′*, *6′*_ = 8.4.0 Hz, H-3′/H-5′), 6.75 (1H, s, pyrimidine-H), 2.25 (6H, s, CH_3_); EI-MS *m/z* (% rel. abund.): 399.3[M+2]^+^-SO_2_ (64), 29], 397.3 [M^+^-SO_2_ (64), 88], 396.3 (98), 362.3 (18), 350.3 (10), 213.2 (97.4), 184.1 (16).

#### 2-Bromo-*N*-(4-(*N*-(4,6-dimethylpyrimidin-2-yl)sulfamoyl)phenyl)benzamide (28)

White solid; yield:47%; m. p. 235–237 °C; TLC (Hexane: EtOAc, 8:2 v/v): R_f_ = 0.52; IR (KBr): ν_max_ cm^-1^ 3237, 3059, 2854, 1659, 1595, 1535, 1395, 1139, 845, 585; UV/Vis (MeOH): λ_max_ nm 213, 264; ^1^H-NMR (400 MHz, DMSO-*d*_*6*_): *δ* 11.46 (s, 1H, SO_2_NH), 10.83 (1H, s, CONH), 7.97 (2H, d, *J*_*2′*, *3′/6′*, *5′*_ = 8.4 Hz, H-2′/H-6′), 7.84 (2H, d, *J*_*3′*, *2′/5′*, *6′*_ = 8.4 Hz, H-3′/H-5′), 7.71 (1H, d, *J*_*3*,*4*_ = 7.6 Hz, H-3), 7.55 (1H, d, *J*_*6*,*5*_ = 7.2 Hz, H-6), 7.49 (1H, t, *J* = 7.2 Hz, H-5), 7.42 (1H, t, *J* = 8.0 Hz, H-4), 6.76 (1H, s, pyrimidine-H), 2.25 (6H, s, CH_3_); EI-MS *m/z* (% rel. abund.): 398.0 [M +2]^+^-SO_2_ (64), 94], 397.0 (81), 396.0 [M^+^-SO_2_ (64), 100], 395.0 (70), 317.1 (4), 213.0 (100), 184.9 (76), 155.0 (13), 157.0 (14).

#### 4-Bromo-*N*-(4-(*N*-(4,6-dimethylpyrimidin-2-yl)sulfamoyl)phenyl)benzamide (29)

White solid; yield: 82%; m. p. 227–228 °C; TLC (Hexane: EtOAc, 4:6 v/v): R_f_ = 0.44; IR (KBr):ν_max_ cm^-1^ 3276, 3059, 2854, 1655, 1594, 1521, 1397, 1313, 1158, 581; UV/Vis (MeOH): λ_max_ nm 208, 274; ^1^H-NMR (400 MHz, DMSO-*d*_*6*_): *δ* 10.64 (1H, s, CONH), 7.96 (2H, d, *J*_*2′*, *3′/6′*, *5′*_ = 8.8 Hz, H-2′/H-6′), 7.92 (2H, d, *J*_*2*,*3/6*,*5*_ = 7.6 Hz, H-2/H-6), 7.90 (2H, d, *J*_*3′*, *2′/5′*, *6′*_ = 8 Hz, H-3′/H-5′), 7.74 (2H, d, *J*_*3*,*2/5*,*6*_ = 8 Hz, H-3/H-5), 6.75 (1H, s, pyrimidine-H), 4.2 (1H, s, SO_2_NH), 2.24 (6H, s, CH_3_); EI-MS *m/z* (% rel. abund.): 398.9 (15.9), 397.9 [M+2]^+^-SO_2_ (64), 68], 396.9 (80.7), 395.9 [M^+^-SO_2_ (64), 71], 394.9 (60), 213.0 (100), 184.9 (63), 154.9 (19), 156.9 (19).

#### 3-Bromo-*N*-(4-(*N*-(4,6-dimethylpyrimidin-2-yl)sulfamoyl)phenyl)benzamide (30)

White solid; yield:76%; m. p 228–230 °C; TLC (Hexane: EtOAc, 8:2 v/v): R_f_ = 0.52IR (KBr): ν_max_ cm^-1^ 3385, 3062, 2945, 1665, 1592, 1523, 1316, 1159, 582, 553; UV/Vis (MeOH): λ_max_ nm 213, 278; ^1^H-NMR (300 MHz, DMSO-*d*_*6*_): *δ* 11.56 (s, 1H, CONH), 10.62 (1H, s, SO_2_NH), 8.13 (1H, t, *J* = 1.8 Hz, H-2), 7.98 (2H, d, *J*_*2′*, *3′/6′*, *5′*_ = 9.0 Hz, H-2′/H-6′), 7.93 (1H, d, *J*_*6*,*5*_ = 7.2 Hz, H-6), 7.90 (2H, d, *J*_*3′*, *2′/5′*, *6′*_ = 8.7 Hz, H-3′/H-5′), 7.80 (1H, dd, *J* = 7.2 Hz, *J* = 1.0 Hz, H-4), 7.50 (1H, t, *J* = 7.8 Hz, H-5), 6.75 (1H, s, pyrimidine-H), 2.24 (6H, s, CH_3_); EI-MS *m/z* (% rel. abund.): 398.2 [M+2]^+^-SO_2_ (64), 87.6], 397.1 (100), 396.2 [M^+^-SO_2_ (64), 82], 395.1 (76), 213.2 (67), 183.0 (33), 185.0 (32).

#### *N*-(4-(*N*-(4,6-dimethylpyrimidin-2-yl)sulfamoyl)phenyl)-3-iodobenzamide (31)

White solid; yield:55%; m. p. 208–210 °C; TLC (Hexane: EtOAc, 6:4 v/v): R_f_ = 0.44; IR (KBr): ν_max_ cm^-1^ 3388, 3074, 2923, 1664, 1592, 1525, 1316, 1160, 1081, 556;UV/Vis (MeOH): λ_max_ nm 217, 275; ^1^H-NMR (400 MHz, DMSO-*d*_*6*_): *δ* 11.39 (1H, s, SO_2_NH), 10.59 (1H, s, CONH), 8.28 (1H, s, H-2), 7.94 (2H, d, *J*_*2′*, *3′/6′*, *5′*_ = 8.8 Hz, H-2′/H-6′), 7.97 (2H, d, *J*_*6*,*5*_ = 7.6 Hz, H-4, H-6), 7.90 (2H, d, *J*_*3′*, *2′/5′*, *6′*_ = 8.8 Hz, H-3′/H-5′), 7.33 (H, t, *J* = 7.6 Hz, H-5), 6.75 (1H, s, pyrimidine-H), 2.25 (6H, s, CH_3_); EI-MS *m/z* (% rel. abund.): 446.1 (3), 445.1 (18), 444.1 [M^+^-SO_2_ (64), 100], 443.1 (85), 316.2 (4), 291.2 (2), 231.0 (50), 213.2 (48), 203.0 (20), 76.0 (13).

#### *N*-(4-(*N*-(4,6-dimethylpyrimidin-2-yl)sulfamoyl)phenyl)-3-methoxybenzamide (32)

White crystalline solid; yield: 62%; m. p. 188–190 °C; TLC (Hexane: EtOAc, 6:4 v/v): R_f_ = 0.49; IR (KBr): ν_max_ cm^-1^ 3346, 3158, 3062, 2925, 1665, 1592, 1530, 1319, 1163, 1068, 842, 587; UV/Vis (MeOH): λ_max_ nm 212, 277; ^1^H-NMR (400 MHz, DMSO-*d*_*6*_): *δ* 10.51 (1H, s, CONH), 7.96 (2H, d, *J*_*2′*, *3′/6′*, *5′*_ = 8.8 Hz, H-2′/H-6′), 7.92 (2H, d, *J*_*3′*, *2′/5′*, *6′*_ = 8.8 Hz, H-3′/H-5′), 7.52 (1H, d, *J*_*6*,*5*_ = 7.6 Hz, H-6), 7.47 (1H, s, H-2), 7.44 (1H, t, *J* = 7.6 Hz, H-5), 7.16 (1H, d, *J*_*4*,*5*_ = 8.0 Hz, H-4), 6.75 (1H, s, pyrimidine-H), 3.82 (3H, s, CH_3_O-), 3.47 (1H, s, SO_2_NH), 2.25 (6H, s, CH_3_); EI-MS *m/z* (% rel. abund.): 349.3 (20), 348.3 [M^+^-SO_2_ (64), 100], 347.1 (58), 259.2 (3), 213.2 (31), 135.1 (77), 107.1 (20).

#### *N*-(4-(*N*-(4,6-dimethylpyrimidin-2-yl)sulfamoyl)phenyl)-3,5-dimethoxybenzamide (33)

White solid; yield:41%; m. p. 226–227 °C; TLC (Hexane: EtOAc, 6:4 v/v): R_f_ = 0.43; IR (KBr): ν_max_ cm^-1^ 3306, 2932, 2949, 1653, 1593, 1510, 1313, 1315, 1162, 1049, 945; UV/Vis (MeOH): λ_max_ nm 214, 278; ^1^H-NMR (400 MHz, DMSO-*d*_*6*_): *δ* 11.44 (1H, s, SO_2_NH), 10.45 (1H, s, CONH), 7.96 (2H, d, *J*_*2′*, *3′/6′*, *5′*_ = 8.4 Hz, H-2′/H-6′), 7.90 (2H, d, *J*_*3′*, *2′/5′*, *6′*_ = 8.4 Hz, H-3′/H-5′), 7.82 (2H, d, *J*_*2*,*4/6*,*4*_ = 2 Hz, H-2/H-6), 6.75 (1H, s, pyrimidine-H), 6.72 (1H, s, H-4), 3.80 (6H, s, CH_3_O-) 2.25 (6H, s, CH_3_); EI-MS *m/z* (% rel. abund.): 379.3 (27), 378.3 [M^+^-SO_2_ (64), 100], 377.1 (65), 256.2 (4), 213.1 (17), 165.1 (80), 122.1 (15)

#### *N*-(4-(*N*-(4,6-dimethylpyrimidin-2-yl)sulfamoyl)phenyl)-4-methylbenzamide (34)

White solid; yield: 83%; m. p. 216–218 °C; TLC (Hexane: EtOAc, 6:4 v/v): R_f_ = 0.63; IR (KBr): ν_max_ cm^-1^ 3371, 3135, 2794, 1667, 1593, 1534, 1320, 1161, 1083, 831, 558; UV/Vis (MeOH): λ_max_ nm 210, 274; ^1^H-NMR (400 MHz, DMSO-*d*_*6*_): *δ*10.46 (1H, s, CONH), 7.96 (2H, d, *J*_2′, 3′/6′, 5′_ = 9.2 Hz, H-2′/H-6′), 7.93 (2H, d, *J*_3′, 2′/5′, 6′_ = 9.2 Hz, H-3′/H-5′), 7.86 (2H, d, *J*_*2*_,_*3/6*_,_*5*_ = 8.0 Hz, H-2/H-6), 7.33 (2H, d, *J*_*3*_,_*2/5*_,_*6*_ = 8.0 Hz, H-3/H-5), 3.95 (1H, s, SO_2_NH), 6.75 (1H, s, pyrimidine-H), 2.37 (3H, s, CH_3_-4). 2.25 (6H, s, pyrimidine-CH_3_); EI-MS *m/z* (% rel. abund.): 333.0 (28.4), 332.0 [M^+^-SO_2_ (64), 100], 331.0 (79.9), 213.2 (26), 119.0 (100), 91.0 (46).

#### *N*-(4-(*N*-(4,6-dimethylpyrimidin-2-yl)sulfamoyl)phenyl)-2-methylbenzamide (35)

White solid; yield:63%; m. p. 216–218 °C; TLC (Hexane: EtOAc, 6:4 v/v): R_f_ = 0.62; IR (KBr): ν_max_ cm^-1^ 3330, 3240, 3162, 2924, 1664, 1595, 1526, 1315, 1157, 1091, 878, 581; UV/Vis (MeOH): λ_max_ nm 211, 272; ^1^H-NMR (400 MHz, DMSO-*d*_*6*_*)*: *δ* 11.41 (1H, s, SO_2_NH), 10.62 (1H, s, CONH), 7.95 (2H, d, *J*_*2′*, *3′/6′*, *5′*_ = 8.4 Hz, H-2′/H-6′), 7.87 (2H, d, *J*_*3′*, *2′/5′*, *6′*_ = 8.4 Hz, H-3′/H-5′), 7.45 (1H, d, *J*_*6*_,_*5*_ = 7.2, H-6), 7.39 (1H, t, *J* = 6.8 Hz, H4), 7.30 (1H, d, *J*_*3*_,_*4*_ = 7.6 Hz, H-3), 7.29 (1H, t, *J* = 7.6 Hz, H-5), 6.75 (1H, s, pyrimidine-H), 2.49 (3H, s, CH_3_-2), 2.25 (6H, s, pyrimidine-CH_3_); EI-MS *m/z* (% rel. abund.): 333.0 (24), 332.0 [M^+^-SO_2_ (64), 99], 331.0 (65), 213.2 (18), 119.0 (100), 91.0 (40).

#### *N*-(4-(*N*-(4,6-dimethylpyrimidin-2-yl)sulfamoyl)phenyl)-3-methylbenzamide (36)

White solid; yield: 61%; m. p. 219–220 °C; TLC (Hexane: EtOAc, 6:4 v/v): R_f_. = 0.58; IR (KBr): ν_max_ cm^-1^3349, 3062, 2924, 1659, 1594, 1527, 1318, 1157, 1082, 857, 580; UV/Vis (MeOH): λ_max_ nm 213, 273; ^1^H-NMR (400 MHz, DMSO-*d*_*6*_): *δ* 11.53 (1H, s, SO_2_NH), 10.50 (1H, s, CONH), 7.96 (2H, d, *J*_*2′*, *3′/6′*, *5′*_ = 8.4 Hz, H-2′/H-6′), 7.92 (2H, d, *J*_*3′*, *2′/5′*, *6′*_ = 8.4 Hz, H-3′/H-5′), 6.75 (1H, s, pyrimidine-H), 2.38 (3H, s, CH_3_-3), 2.25 (6H, s, pyrimidine-CH_3_); EI-MS *m/z* (% rel. abund.): 333.0 (24), 332.1 [M^+^-SO_2_ (64), 100], 331.0 (65), 213.2 (29), 119.0 (84), 91.0 (33).

#### *N*-(4-(*N*-(4,6-dimethylpyrimidin-2-yl)sulfamoyl)phenyl)-3-phenylpropanamide (37)

White solid; yield: 50%; m. p. 195–197 °C; TLC (Hexane: EtOAc, 6:4 v/v): R_f_ = 0.67; IR (KBr): ν_max_ cm^-1^ 3287, 3176, 3034, 2928, 1697, 1596, 1535, 1429, 1160, 1083, 858, 582; UV/Vis (MeOH): λ_max_ nm 211, 266; ^1^H-NMR (400 MHz, DMSO-*d*_*6*_): *δ* 10.27 (s, 1H, CONH), 7.89 (2H, d, *J*_*3′*, *2′/5′*, *6′*_ = 9.0 Hz, H-3′/H-5′), 7.70 (2H, d, *J*_*2′*, *3′/6′*, *5′*_ = 9.0 Hz, H-2′/H-6′), 7.13–7.28 (5H, m, C_6_H_5_-), 6.75 (1H, s, Pyrimidine-H), 2.88 (2H, t, *J* = 7.5 Hz, CH_2_-3), 2.64 (2H, t, *J* = 7.5 Hz, CH_2_-2), 2.25 (6H, s, CH_3_); EI-MS *m/z* (% rel. abund.): 347.2 (23), 346.1 [M^+^-SO_2_ (64), 100], 345.1.0 (88), 214.2 (92), 105.1 (13), 91.0 (22).

#### *N*-(4-(*N*-(4,6-dimethylpyrimidin-2-yl)sulfamoyl)phenyl)-3,5-dimethylbenzamide (38)

White solid; yield:55%;m. p. 216–218 °C; TLC (Hexane: EtOAc, 6:4 v/v): R_f_ = 0.55; IR (KBr):ν_max_ cm^-1^3332, 3092, 2921, 1659, 1595, 1522, 1312, 1164, 1086, 836, 582; UV/Vis (MeOH): λ_max_ nm 213, 278; ^1^H-NMR (400 MHz, DMSO-*d*_*6*_): *δ*10.46 (1H, s, CONH), 7.95 (2H, d, *J*_*2′*, *3′/6′*, *5′*_ = 9.2 Hz, H-2′/H-6′), 7.91 (2H, d, *J*_*3′*, *2′/5′*, *6′*_ = 9.2 Hz, H-3′/H-5′), 7.54 (2H, s, H-2/H-6), 7.22 (1H, s, H-4), 6.75 (1H, s, pyrimidine-H), 3.52 (1H, s, SO_2_NH), 2.34 (6H, s, CH_3_-3), 2.25 (6H, s, pyrimidine-CH_3_); FAB-MS (positive mode) *m/z* 411.3 [M+ H]^+^.

#### *N*-(4-(*N*-(4,6-dimethylpyrimidin-2-yl)sulfamoyl)phenyl)benzamide (39)

White solid; yield:79%; m. p. 233–236 °C; TLC (Hexane: EtOAc, 4:6 v/v): R_f_ = 0.45; IR (KBr): ν_max_ cm^-1^ 3344, 3108, 2925, 1660, 1595, 1528, 1157; UV/Vis (MeOH): λ_max_ nm 209, 207; ^1^H-NMR (400 MHz, DMSO-*d*_*6*_): *δ* 11.49 (s, 1H, SO_2_NH), 10.54 (1H, s, CONH), 7.97 (2H, d, *J*_*2′*, *3′/6′*, *5′*_ = 9.2 Hz, H-2′/H-6′), 7.95 (2H, d, *J*_*3′*, *2′/5′*, *6′*_ = 9.2 Hz, H-3′/H-5′), 7.94 (2H, d, *J*_*2*, *3/6*, *5*_ = 8.4 Hz, H-2/H-6), 7.60 (1H, t, *J* = 7.2 Hz, H-4), 7.50 (2H, t, *J* = 7.6 Hz, H-3/H-5), 6.75 (1H, s, pyrimidine-H), 2.25 (6H, s, CH_3_). EI-MS *m/z* (% rel. abund.): 213.0 (85.9), 214.1 [M^+^-SO_2_ (64), 100], 215.1 (10.9), 228.2 (1.4), 213.1 (100), 199.1 (7.2).

#### Immunomodulatory studies

All studies on human blood cells was carried out after an approval from independent ethics committee (Prof. Dr. Ghazala H. Rizwani Chair, IEC, Prof. Qamar Amin, Prof, Dr. Muddasir Uddin, Dr. Shahnaz Ghazi, Dr. Samiuz Zaman, Prof. Dr. Ahsana Dar Farooq, Dr. M. Raza Shah member IEC), International Center for Chemical and Biological Sciences, University of Karachi, No: ICCBS/IEC-008-BC-2015/Protocol/1.0. Informed consents were obtained from the volunteers before drawing the blood. All the experiments were performed in accordance with relevant guidelines and regulations. The approval of institutional ethical committee approval can be seen from supporting information.

LSM (50494, MP Biomedicals, lllkirch, France); HBSS++ and HBSS—(14025, Gibco, California, USA); Ethanol (1070172511, Merck, Darmstadt, Germany); FBS (A11-104, PAA, Pasching, Austria); RPMI-1640 (R8758, Sigma Aldrich, St. Louis, USA); 10 mL sterile syringes (305559, BD Biosciences, NJ, USA); Luminol (A14597, Alfa Aesar, Karlsrushe, Germany); 96-well white half area plates (3693,Costar, NY, USA); Zymosan (26701494, Wako, Shanghai, China).

#### Isolation of human polymorphonuclear cells (PMNs)

Heparinized blood was obtained by vein puncture aseptically from healthy volunteers. The PMNs were isolated by ficoll-Hypaque density gradient method. The cells were isolated after centrifugation from the tube base. Cells were washed twice and suspended in Hank’s Balance salt solution (HBSS^--^) (Ca^2+^ and Mg^2+^ free), RBCs were lysed using hypotonic solution. Cells were adjusted to their required concentration (1x10^6^ cells/mL) using HBSS^++^containing Ca^2+^ and Mg^2+^.

All studies on human blood cells was carried out after an approval from independent ethics committee (Prof. Dr. Ghazala H. Rizwani Chair, IEC, Prof. Qamar Amin, Prof, Dr. Muddasir Uddin, Dr. Shahnaz Ghazi, Dr. Samiuz Zaman, Prof. Dr. Ahsana Dar Farooq, Dr. M. Raza Shah member IEC), International Center for Chemical and Biological Sciences, University of Karachi, No: ICCBS/IEC-008-BC-2015/Protocol/1.0. Informed consents were obtained from the volunteers before drawing the blood. All the experiments were performed in accordance with relevant guidelines and regulations.

#### Oxidative burst assay

Luminol-enhanced chemiluminescence assay was performed, as described by Helfandet al., 1982 [17], with some modifications. Briefly 25 μL of diluted whole blood in HBSS^++^ (Hanks Balanced Salt Solution, containing calcium chloride and magnesium chloride) [Sigma, St. Louis, USA] was incubated with 25 μL of three different concentrations of compounds (1, 10, and 100 μg/mL), each in triplicate. Control wells received HBSS^++^ and cells, but no compounds. Test was performed in white half area 96-well plates [Costar, NY, USA], which was incubated at 37 °C for 15 minutes in the thermostat chamber of luminometer [Labsystems, Helsinki, Finland]. After incubation, 25 μL of serum opsonized zymosan (SOZ) [Fluka, Buchs, Switzerland] and 25 μL of intracellular reactive oxygen species detecting probe, luminol [Research Organics, Cleveland, OH, USA] were added into each well, except blank wells (containing only HBSS^++^). The level of the ROS was recorded in luminometer in term of relative light units (RLU).

#### Measurement of nitric oxide

The mouse macrophage cell line J774.2 (European Collection of Cell Cultures, UK) was cultured in 75 cc flasks IWAKI (Asahi Techno Glass, Japan) in DMEM Sigma-Aldrich (Steinheim, Germany) supplemented with 10% fetal bovine serum GIBCO (New York, USA)1% streptomycin/penicillin. Flasks were kept at 37 °C in atmosphere of humidified air containing 5% CO_2_, cells were seeded in 96-well plate (10^6^ cells/mL) and were induced by 30 μg/mL *e*. *coli* lipopolysaccharide (LPS) (DIFCO Laboratories michigon, USA). The test compounds were added at concentrations of (1, 10 and 100 μg/mL) and plate was incubated at 37 °C in 5% CO_2_. Nitrite accumulation in culture supernatant was measured using the Griess reagents as described previously [18].

#### Cytokine production and their quantification

RPMI-1640 (R8758, Sigma Aldrich, St. Louis, USA); L-glutamine (25030081, Gibco, California, USA); D-glucose (G8270, Sigma Aldrich, St. Louis, USA); 2-mercaptoethanol (190242, MP Biomedicals, lllkirch, France); sodium pyruvate (11360–070, Gibco, California, USA); FBS (A11-104, PAA Laboratories, Pasching, Austria); HEPES (H3375, Sigma Aldrich, St. Louis, USA); PMA (151864, MP Biomedicals, lllkirch, France); lipopolysaccharide B of *E*. *coli* 0111:B4 (LPS) (3922–25, DIFCO Laboratories, USA); 24- well tissue culture plates (CLS3524, Corning, Dublin, USA); Flat bottom 96-well tissue culture plates (CLS3300, Corning, New York, USA). BSA (151429, MP Biomedicals, lllkirch, France); PBS tablets (41620016–4, Bioworld, Dublin, Ireland); Tween 20 (822184, Merck, Damstadt, Germany); Human TNF-α DuoSet ELISA Kit (DY210, R&D Systems, Minneapolis, USA); Human IL-1β DuoSet ELISA Kit (DY201, R&D Systems, Minneapolis, USA); and Human IL-2 DuoSet ELISA Kit (DY202, R&D Systems, Minneapolis, USA); ELISA plate sealers (DY992, R&D Systems, Minneapolis, USA); Clear flat bottom 96-well microplates (DY990, R&D Systems, Minneapolis, USA). Human monocytic leukemia cells THP-1 were obtained from (European Collection of Cell Cultures, UK). The cells were maintained in RPMI-1640 containing 5.5 mmol/L glucose (BioM Laboratories, Chemical Division, Malaysia), 50 μmol/L mercaptoethanol (Merck Damstadt, Germany), 10% FBS (fetal bovine serum), 2 mmol/L; L-glutamine (PAA Laboratories, GmbH, Pasching, Austria). Cells were grown in 75 cc flasks, harvested and 2.5×10^5^ cells/mL was then plated in 24-well tissue culture plates. 20 ng/mL of phorbol-12-myristate-13-acetate (PMA), (SERVA, Heidelberg, Germany) was added followed by incubation for 24 hours at 37 °C in 5% CO_2_to convert them into macrophage like cell. Cells were then stimulated with *E*. *coli* lipopolysacchride B, (DIFCO Laboratories, michigon, USA) at a final concentration of 50 ng/mL and treated with compounds at three different concentration (1, 10 and 100 μg/mL) and then incubated for 4 hours at 37 °C in 5% CO_2_. The supernatants collected were analyzed for the level of TNF -*α* using the human TNF-*α* Duo Set ELISA (R&D Systems, Minneapolis, USA), and according to manufacturer’s instructions [18].

#### Cytotoxicity assay

Cytotoxic activity of compounds was evaluated in 96-well flat-bottom micro titer plates by using the standard MTT (3-[4, 5-dimethylthiazole-2-yl]-2, 5-diphenyl-tetrazolium bromide) colorimetric assay [19]. In this assay, mouse fibroblast 3T3 cells were grown in Dulbecco’s Modified Eagle Medium (DMEM), along with 5% Fetal Bovine Serum (FBS), 100 μg/mL of streptomycin, and 100 IU/ mL of penicillin in 75 cm^2^ flasks. The flasks were then incubated at 37 °C in 5% CO_2_. Cells were harvested, followed by counting with haemocytometer, and dilution with a medium. Cell culture was prepared with the concentration of 5 x 10^4^ cells/mL, followed by introduction (100 μL/ well) into 96-well plates. Medium was removed after overnight incubation. Freshly prepared medium (200 μL) was added with various concentrations of test compounds (1–30 μM). After 48 hours, 200 μL MTT dye (0.5 mg/mL) was added into all wells, and again incubated for 4 hours. DMSO (100 μL) was added to each well. The formation of formazan dye within the cells was calculated by measuring the absorbance at 540 nm using a using a micro plate reader. The cytotoxicity was recorded as concentration causing 50% growth inhibition (IC_50_).

#### Anti-bacterial activity

The anti-bacterial activity of synthesized compound were evaluated by the Microplate Alamar Blue Assay [20, 21]. *Staphylococcus aureus* (ATCC 25923), *Pseudomonas aeruginosa*(POA 286), *Salmonella typhi* (ATCC 14028), *Bacillus subtilis* ATCC 6633) and *Escherichia coli* (ATCC 12022) were used in this study as a standard bacterial strains and they were obtained from microbial bank of PCMD, International Center for Chemical and Biological Sciences, University of Karachi, Karachi-75270, Pakistan. The bacterial colonies grown in Trypton Soya Agar (TSA) (Oxoid Limited, UK) were inoculated in Mueller Hinton Broth (MHB) (Oxoid Limited, UK) and incubated overnight at 37 °C. Fully grown bacterial cultures were then diluted to adjust with 0.5 McFarland Turbidity Index (equivalent to 1.5 × 10^8^ CFU/mL). Stock solutions (4 mg/mL) of different test compounds were prepared in DMSO and 10μL each of these stock solutions were placed in well of flat bottom, polystyrene, sterile 96-wells plate except the positive control wells (media + bacteria). This gives 200μg/mL concentrations of compounds in final 200 μL solution. Each compound was run in triplicates. Finally, bacterial suspension (5 x 10^6^ CFU/mL) was added in each well. Plates were sealed by parafilm and incubated at 37 °C for 18–22 hrs. Next day, 20 μL of 0.02% resazurin sodium salt dye (Chem-Impex-Int’LInc) was added in each well and incubated in shaking incubator at 80 rpm, 37 °C for 2–3 hrs. The color change from blue to reddish pink indicates the growth of bacteria. For quantitative analysis, plates were read at 570 nm and 600 nm in Multiskan GOmicroplate spectrophotometer, (Thermo Scientific, USA). The % inhibition of bacterial growth was calculated using below mentioned formula.

For the calculation of the % difference in reduction between treated and control cells in cytotoxicity/proliferation assays:

$$=\frac{(\varepsilon \text{OX})\lambda 2\;A\lambda 1-(\varepsilon OX)\lambda 1\;A\lambda 2\;of\;test\;agent}{(\varepsilon OX)\lambda 2\;A{}^{\circ}\lambda 1-(\varepsilon OX)\lambda 1\;A{}^{\circ}\lambda 2\;of\;positive\;growth\;control}\times 100$$

For the % inhibition of test compound as compared to positive control cell:

=100-(%ddifferenceinreductionbetweentreatedandpositivecontrolcells)

#### Statistical analysis

Results are expressed as mean values ± SD (Standard Deviation). The statistical significance of the difference between control and treated samples was calculated by the Student’s *t*-test. The differences were considered to be highly significant at *P* ≤ 0.05.

## Results and discussion

During the current study 37 membered library of amide derivatives of sulfamethazine was synthesized by using conventional methods being employed in organic synthesis. The purified yields of resulting amides ware between 42–96%. Progress of the reaction was monitored by thin layer chromatography (TLC). After complete consumption of substrate, the reaction mixture was cooled, precipitates were filtered, washed with mixture of hexane, and ethyl acetate, followed by washing with acetone. Structures of all synthetic compounds **3–39** were elucidated by modern spectroscopic technique; such as ^1^H- and ^13^C-NMR, EI-MS, FAB-MS, HRFAB-MS, UV, and IR spectroscopy.

### General structure elucidation of a representative compound 6

**Fig 1:**
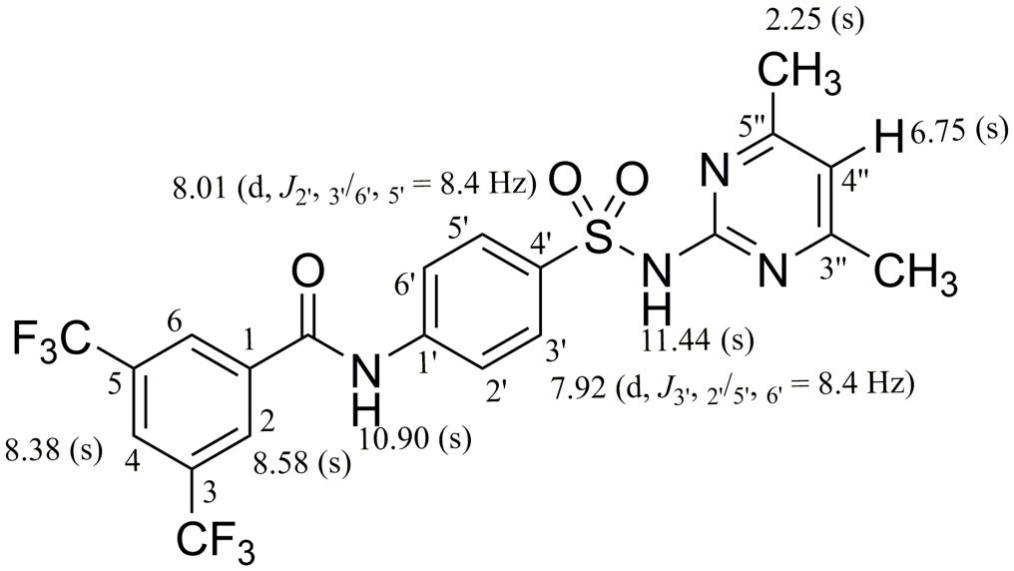
^1^H-NMR chemical shifts of compound 6.

^1^H- and ^13^C-NMR spectra of one of the most bioactive members of the series (compounds **6**) is presented here. In ^1^H-NMR spectrum, recorded in DMSO-*d*_*6*_, downfield broad singlets of the acidic SO_2_NH proton, and CONH appeared at *δ*_H_11.44 and10.90, respectively. The *para*-disubstituted aromatic H-2′/H-6′ resonated at *δ*_H_ 8.01 (2H, d, *J*_2*′*, *3′/6′*, *5′*_ = 8.4 Hz) with *ortho* couplings with H-3′/H-5′. Similarly, H-3′/H-5′ resonated at *δ*_H_ 7.92 (2H, d, *J*_*3′*, *2′/5′*, *6′*_ = 8.4 Hz) showed *ortho* couplings with H-2′/H-6′. The other downfield set of protons H-2/H-6, and H-4 of 3,5-bis(trifluoromethyl)benzoyl substituent appeared as singlets at *δ*_H_ 8.58 and 8.38, respectively. This downfield signal was due to the electron withdrawing effect attachedCF_3_ groups. The two CH_3_ protons attached to pyrimidine ring appeared as singlet at *δ*_H_ 2.25. The characteristic signal of methine H-4″ of pyrimidine ring appeared as a singlet at *δ*_H_ 6.75 (Fig 1).

**Fig 2:**
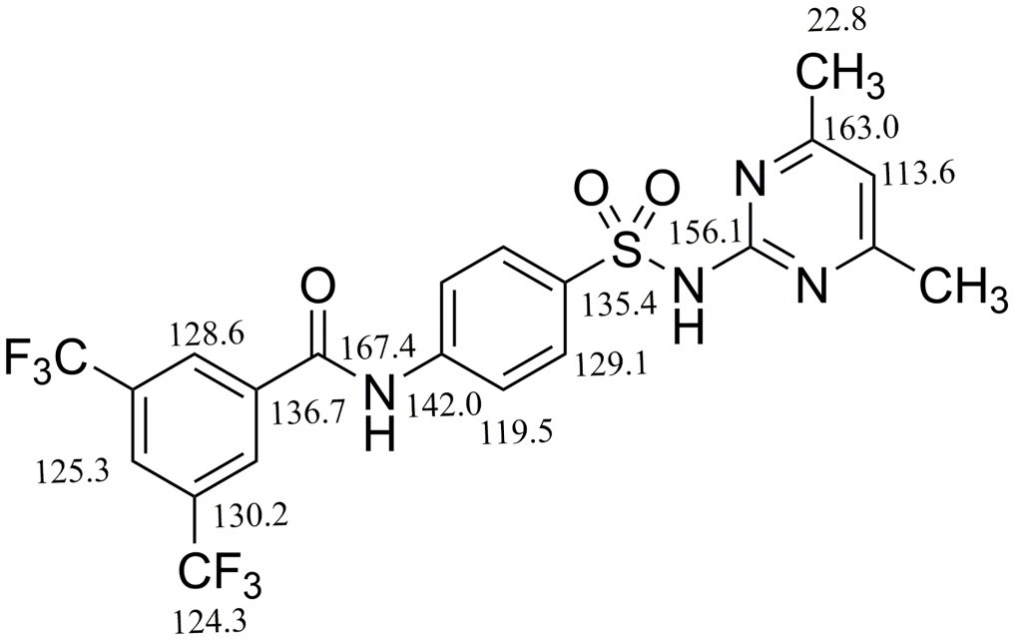
^13^C-NMR chemical shifts of compound 6.

In ^13^C-NMR broad-band decoupled DEPT spectrum of **6**, in DMSO-d_6_ showed 21 carbon signals, including 2 methyl, 8 methine, and 11 quarternary carbons. The downfield signal of amide carbonyl carbon appeared at *δ*C 167.4. The second most downfield signal of C-5'' appeared at *δ*_C_162.9 due to the quaternary carbon directly, attached to nitrogen of the pyrimidine ring. Another quaternary carbon (C-1″) which is in proximity three nitrogen atoms appeared at *δ*156.1. Two quaternary carbons (C-1ʹ and C-4ʹ) that are directly attached to NH of amide and SO_2_ of sulphonamide appeared at *δ*_C_ 142.0, and 135.4, respectively. Four aromatic methine carbons of the *para*-disubstituted aromatic ring appeared at *δ*_C_ 119.5, and 129.1 assigned to C-2'/C-6', and C-3'/ C-5', respectively. Three aromatic methine carbons of 3, 5-*bis*(trifluoromethyl)benzoyl substituent appeared at *δ*_C_125.3, and 128.6 assigned to C-2/C-6 and C-4, respectively. C-4'' of pyrimidine ring appeared at *δ*_C_113.6. CF_3_ carbon appeared at *δ*_C_124.3 while aromatic quaternary C-3/5, *ipso* to CF_3_ group resonated at *δ*_C_130.2. Aromatic quaternary carbon attached to CO of amide bond resonated at *δ*_C_ 136.7. The most up field methyl carbons attached to C-3''and C-5'' appeared at *δ*_C_ 22.8 (Fig 2).

The EI-MS of compound **6** did not show the molecular ion peak [M]^+^ at 518.1but showed the peak at 454.2 [M^+^-SO_2_(64)] arose from the elimination SO_2_ group. Loss of COPh (CF_3_)_2_ from *m/z* 454.2 [M^+^-SO_2_(64)] yielded a peak at *m/z* 320, while a radical cation for COPh(CF_3_)_2_ appeared at *m/z* 241.

HRFAB-MS (positive mode) displayed the [M+H]^+^ at *m/z* 519.0926, supporting the composition of C_21_H_17_F_6_N_4_O_3_S(Calcd.519.0926).

### Anti-inflammatory activities *in vitro*

**Fig 3:**
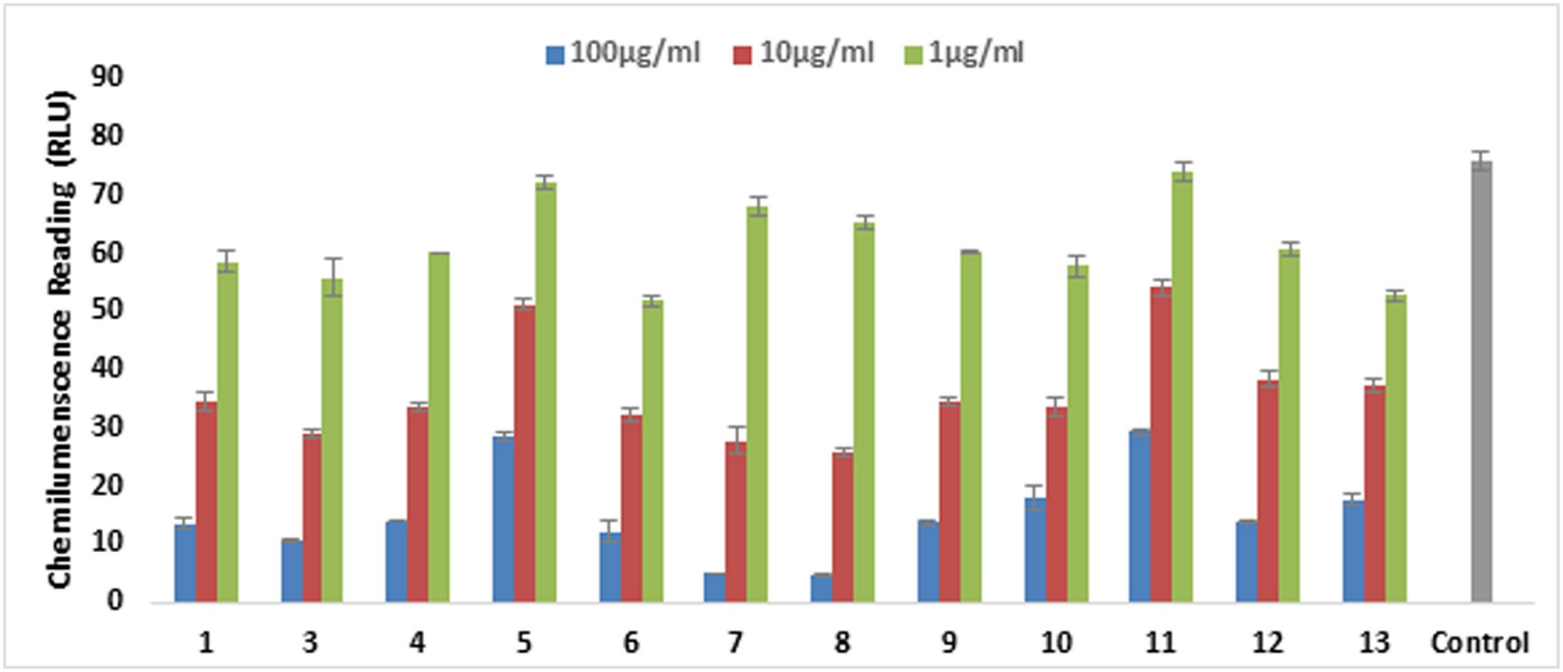
The graph represents the effect of compounds 1 and 3–13 on oxidative burst. Compounds were tested on three different concentrations. Results are presented in relative light units (RLU) and oxidative burst activity of whole blood using luminol as a probe. Each vertical bar represents a mean of triplicate. Error bars represent mean Standard deviation of three determinations. Significance difference was compared to the control = no drug.

**Fig 4:**
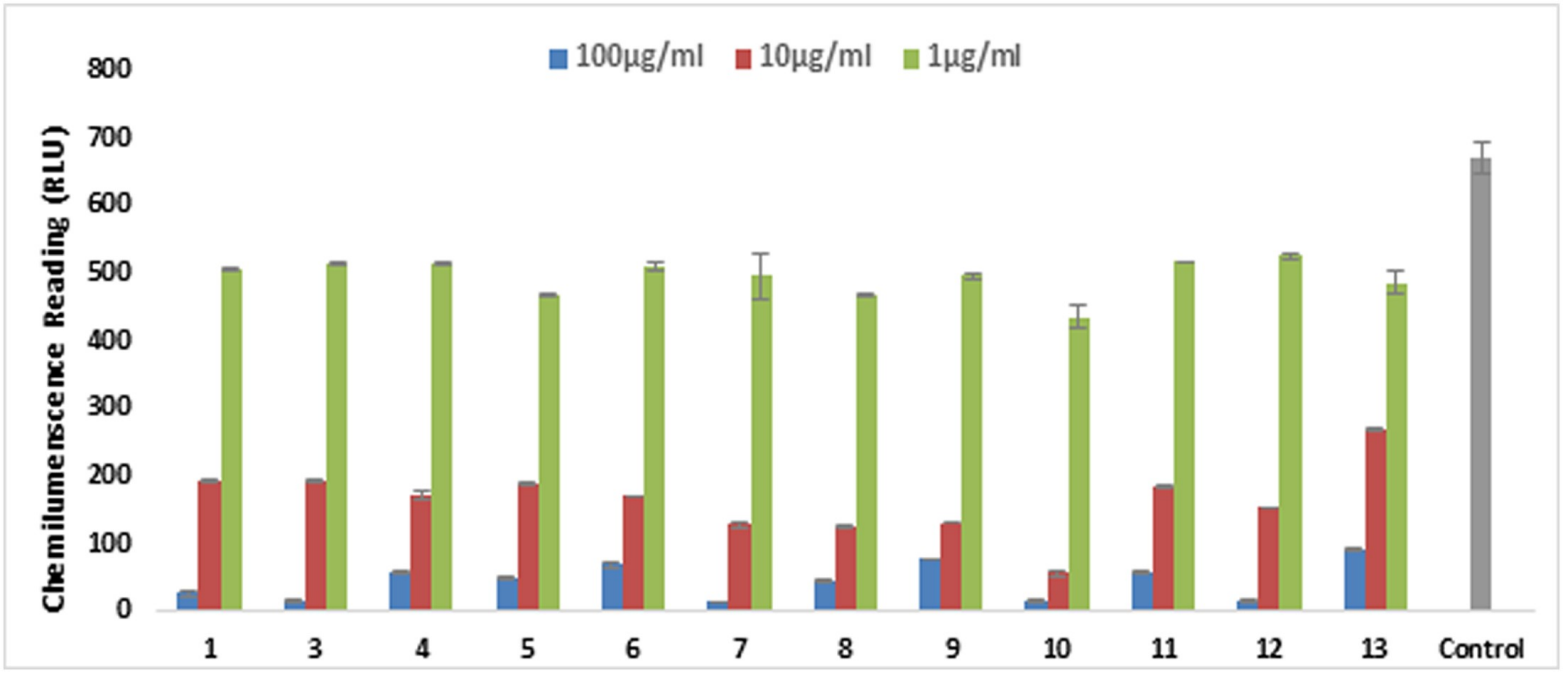
The graph represents the effect of compounds 1 and 3–13 on oxidative burst. Compounds were tested on three different concentrations. Results are presented in relative light units (RLU) and oxidative burst activity of polymorphonuclear neutrophils (PMNs) using luminol as a probe. Each vertical bar represents a mean of triplicate. Error bars represent mean Standard deviation of three determinations. Significance difference was compared to the control = no drug.

All sulfamethazine amide derivatives **3**–**39** were evaluated for their *in vitro* protein anti-inflammatory activity, as well as on various parameters of inflammation. Among them compounds **3–10, 14**, and **15** were identified as new compounds. All the compounds have not been reported for their anti-inflammatory activities earlier. During the current study, compounds **3–39** and parent drug sulfamethazine (**1**) were initially tested for their inhibitory potential on the production of intracellular ROS from zymosan activated whole blood phagocytes. Compounds **1**, and **3**–**13** inhibited the production of ROS from human whole blood cells. Compound **1**, and its derivatives **3**–**13** were further evaluated for the inhibition of the ROS production from neutrophils, isolated from human whole blood. All tested compounds were found to be the potent inhibitors of ROS, except compounds **5** and **11** which showed a moderate level of inhibition (IC_50_ = 20.5 ± 2.2, and 34.6 ± 1.2 μg/mL, respectively) as compared to standard drug ibuprofen (IC_50_ = 11.2 ± 1.9 μg/mL). Fig 3 represents the effect of compounds **1** and **3**–**13** on oxidative burst in the graphical form. Among all tested derivatives, **8** was found to be the most potent inhibitor of ROS from whole blood, and isolated PMNs with an IC_50_ values of 4.2 ± 0.2 and 2.4 ± 0.07, respectively. Fig 4 represents the effect of compounds **1** and **3**–**13** on oxidative burst activity of polymorphonuclear neutrophils (PMNs).

The parent drug **1** along with synthesized derivatives were tested for their NO (nitric oxide) scavenging activity, produced from LPS stimulated mouse macrophage cell lines. All compounds showed a promising inhibitory effect (Table 1), except **11** which showed relatively moderate level of inhibition (IC_50_ = 26.3 ± 1.8 μg/mL) compared to standard LNMMA (IC_50_ = 24.2 ± 0.6 μg/mL). Compounds **3** and **4** were found to be the most potent inhibitor of NO with an IC_50_ value of <1 μg/mL.

**Table 1:** Effect of compounds (1, and 3–39) on oxidative burst from whole blood phagocytes and isolated PMNs, nitric oxide, TNF-α production, and cytotoxicity on 3T3 cells. The IC_50_ (μg/mL) was calculated using three doses (1, 10, and 100 μg/mL) of each compound. Values are expressed as mean ± SD of three determinations.

S. No.	Oxidative Burst Inhibition (IC_50_μg/mL)		NO Inhibition (IC_50_ μg/mL)	TNF-α Inhibition (IC_50_ μg/mL)	Cytotoxicity NIH-3T3 cell line (IC_50_ μg/mL)
	WB	PMNs			
Sulfamethazine (**1)**	8.9 ± 1.3	5.2 ± 0.05	18.9 ± 1.7	>100	>30
**3**	6.5 ± 1.3	3.6 ± 0.05	<1	<1	>30
**4**	7.3 ± 0.4	6.1 ± 0.08	<1	<1	>30
**5**	20.5 ± 2.2	3.0 ± 0.06	6.9 ± 1.9	<1	>30
**6**	5.7 ± 0.8	3.4 ± 0.1	7.8 ± 0.3	<1	>30
**7**	5.8 ± 0.6	2.9 ± 0.3	8.0 ± 0.3	38.9 ± 0.7	>30
**8**	4.2 ± 0.2	2.4 ± 0.07	7.3 ± 0.3	9.5 ± 0.4	>30
**9**	5.7 ± 0.2	2.8 ± 0.05	7.3 ± 0.4	2.1 ± 0.2	>30
**10**	10.6 ± 0.5	3.4 ± 0.4	7.3 ± 0.2	34.5 ± 4.1	>30
**11**	34.6 ± 1.2	6.2 ± 0.13	26.3 ± 1.8	>100	>30
**12**	10.6 ± 0.5	3.3 ± 0.05	10.8 ± 2.1	9.9 ± 0.2	>30
**13**	9.2 ± 0.6	4.9 ± 0.2	10.9 ± 0.5	19.5 ± 1.0	>30
**14**	NA	NT	NT	NT	>30
**15**	NA	NT	NT	NT	>30
**16**	NA	NT	NT	NT	>30
**17**	NA	NT	NT	NT	>30
**18**	NA	NT	NT	NT	>30
**19**	NA	NT	NT	NT	>30
**20**	NA	NT	NT	NT	>30
**21**	NA	NT	NT	NT	>30
**22**	NA	NT	NT	NT	>30
**23**	NA	NT	NT	NT	>30
**24**	NA	NT	NT	NT	>30
**25**	NA	NT	NT	NT	>30
**26**	NA	NT	NT	NT	>30
**27**	NA	NT	NT	NT	>30
**28**	NA	NT	NT	NT	>30
**29**	NA	NT	NT	NT	>30
**30**	NA	NT	NT	NT	>30
**31**	NA	NT	NT	NT	>30
**32**	NA	NT	NT	NT	>30
**33**	NA	NT	NT	NT	>30
**34**	NA	NT	NT	NT	>30
**35**	NA	NT	NT	NT	>30
**36**	NA	NT	NT	NT	>30
**37**	NA	NT	NT	NT	>30
**38**	NA	NT	NT	NT	>30
**39**	NA	NT	NT	NT	>30
**40** (Ibuprofen)	11.2 ± 1.9	2.5 ± 0.6	-	-	-
**41** (L-NMMA)	-	-	24.2 ± 0.6	-	-
**42** (Pentoxifillin)	-	-	-	94.8 ± 2.1	-
**43** (Cyclohexamide)	-	-	-	-	0.26 ± 0.1

WB = Whole Blood, PMNs = Polymorphonuclear neutrophils, NT = Not tested, NA = Not active

L-NMMA = NG monomethyl L-arginine acetate

When tested for their effect on production of pro-inflammatory cytokine TNF-α, all compounds were found to be the potent inhibitors of this cytokine except parent compound **1** and derivative **11**. Compounds **3–6** were found to be the most potent inhibitors of TNF-α with an IC_50_ value of >1μg/mL. Among 37 synthetic derivatives of sulfamethazine 11 derivatives showed a significant anti-inflammatory potential at a varying levels. Compounds **3**, **4**, **6**, **7**, **8**, **9**, and **13** were found to have a better anti-inflammatory activity than parent compound **1**.

All the new compounds, except **14** and **15**, showed anti-inflammatory activities. The cytotoxicity of compounds **3–39** was also evaluated against the mouse fibroblast NIH-3T3 cell line. All the compounds were found to be non-toxic (Table 1). These results showed that modification of sulfonamide’s primary amino group brings promising improvement in the anti-inflammatory profile of parent drug **1**. Limited Structure-Activity-Relationship shows that there is no particular trend for the potent activity which compounds 3–39 follows, however, it was observed that compounds with alkyl and/or halogen substituents at meta or para positions of benzamide ring showed potent activity.

### Antibacterial activity

The synthesized compounds **3–39** were evaluated for their antibacterial activity by Microplate Alamar Blue Assay [20, 21] in comparison to ofloxacin (standard antibiotic) against *Staphylococcus aureus* (ATCC 25923), *Pseudomonas aeruginosa* (POA 286), *Salmonella typhi* (ATCC 14028), *Bacillus subtilis* (ATCC 6633) and *Escherichia coli* (ATCC 12022). The results indicated that most of the compounds, including **1**, showed moderate activity against *Staphylococcus aureus*. The compounds were found to be in active against above mentioned bacteria (Table 2).

**Table 2:** Antimicrobial activity of the synthesized compounds determined by Microplate Alamar Blue Assay.

Percentage (%) Inhibition of compound					
Type	*Gram positive*		*Gram negative*		
Compounds (200 μg/mL)	*Bacillus* *subtilis*	*Staphylococcus* *aureus*	*Escherichia* *coli*	*Pseudomonas* *aerugenosa*	*Slamonella* *typhi*
**1**	NI	42.17	NI	8.92	NI
**3**	NI	60.05	NI	5.93	NI
**4**	NI	3.21	NI	NI	NI
**5**	NI	34.98	NI	NI	NI
**6**	NI	32.66	NI	NI	NI
**7**	NI	54.33	NI	NI	13.10
**8**	NI	47.26	NI	4.31	29.44
**9**	NI	27.94	NI	NI	0.94
**10**	NI	1.09	NI	NI	NI
**11**	35.07	NI	3.93	26.5	29.58
**12**	NI	49.69	NI	NI	18.97
**13**	NI	34.30	NI	6.58	NI
**14**	NI	27.35	NI	1.04	25.43
**15**	38.37	14.12	NI	29.36	29.34
**16**	NI	47.23	NI	NI	19.36
**17**	NI	24.33	NI	NI	11..01
**18**	NI	49.73	NI	NI	22.46
**19**	NI	49.37	NI	2.37	0.99
**20**	NI	30.78	NI	1.66	9.30
**21**	NI	54.62	NI	27.99	14.61
**22**	NI	44.33	NI	9.78	11.59
**23**	NI	5.76	NI	1.54	NI
**24**	NI	48.40	7.45	NI	18.41
**25**	NI	17.48	1.21	6.35	4.64
**26**	NI	34.74	NI	NI	NI
**27**	NI	50.12	NI	NI	3.72
**28**	NI	58.46	4.65	3.99	11.11
**29**	NI	52.33	NI	14.97	14.10
**30**	NI	34.21	NI	NI	4.26
**31**	NI	13.01	NI	NI	NI
**32**	NI	32.92	NI	NI	19
**33**	NI	31.34	NI	NI	NI
**34**	NI	NI	NI	15.42	0.65
**35**	NI	NI	NI	16.48	0.20
**36**	NI	NI	NI	20.54	NI
**37**	NI	10.19	NI	NI	NI
**38**	NI	NI	NI	18.96	NI
**39**	NI	41.09	1.84	NI	12.49
Ofloxacin	93.77	94.08	94.43	94.14	89.75

NI: No inhibition

## Conclusion

During the current study we have synthesized new derivatives of sulfamethazine, and evaluated their immunomodulating effect. Results showed that these compounds possess a promising anti-inflammatory activity which can be used further as a candidate for the treatment of inflammation and related disorder. Further optimization of the synthesized compounds is required in order to obtain enhanced biological activities.

## Supporting information

S1 Fig Spectral data of compound 3. (PDF)

S2 Fig Spectral data of compound 4. (PDF)

S3 Fig Spectral data of compound 5. (PDF)

S4 Fig Spectral data of compound 6. (PDF)

S5 Fig Spectral data of compound 7. (PDF)

S6 Fig Spectral data of compound 8. (PDF)

S7 Fig Spectral data of compound 9. (PDF)

S8 Fig Spectral data of compound 10. (PDF)

S9 Fig Spectral data of compound 11. (PDF)

S10 Fig Spectral data of compound 12. (PDF)

S11 Fig Spectral data of compound 13. (PDF)

S12 Fig Spectral data of compound 14. (PDF)

S13 Fig Spectral data of compound 15. (PDF)

S14 Fig Spectral data of compound 16. (PDF)

S15 Fig Spectral data of compound 17. (PDF)

S16 Fig Spectral data of compound 18. (PDF)

S17 Fig Spectral data of compound 19. (PDF)

S18 Fig Spectral data of compound 20. (PDF)

S19 Fig Spectral data of compound 21. (PDF)

S20 Fig Spectral data of compound 22. (PDF)

S21 Fig Spectral data of compound 23. (PDF)

S22 Fig Spectral data of compound 24. (PDF)

S23 Fig Spectral data of compound 25. (PDF)

S24 Fig Spectral data of compound 26. (PDF)

S25 Fig Spectral data of compound 27. (PDF)

S26 Fig Spectral data of compound 28. (PDF)

S27 Fig Spectral data of compound 29. (PDF)

S28 Fig Spectral data of compound 30. (PDF)

S29 Fig Spectral data of compound 31. (PDF)

S30 Fig Spectral data of compound 32. (PDF)

S31 Fig Spectral data of compound 33. (PDF)

S32 Fig Spectral data of compound 34. (PDF)

S33 Fig Spectral data of compound 35. (PDF)

S34 Fig Spectral data of compound 36. (PDF)

S35 Fig Spectral data of compound 37. (PDF)

S36 Fig Spectral data of compound 38. (PDF)

S37 Fig Spectral data of compound 39. (PDF)

S38 Fig Institutional ethical committee approval. (PDF)

S39 Fig General reaction for the synthesis of compounds 3–39. (TIF)
