# Supplementary material for: Sulphamethazine derivatives as immunomodulating agents: New therapeutic strategies for inflammatory diseases
Source: PLoS One. 2018 Dec 19;13(12):e0208933. doi: 10.1371/journal.pone.0208933 (PMC6300282; doi:10.1371/journal.pone.0208933)
Supplement: S1 Fig — (PDF) [file pone.0208933.s001.pdf]

Check

DR.HAROON/DR.HINA/MHH.I.39
ICCBS,U.O.K/1H

3

AVANCE AV - III
300 MHz, LAB # 116

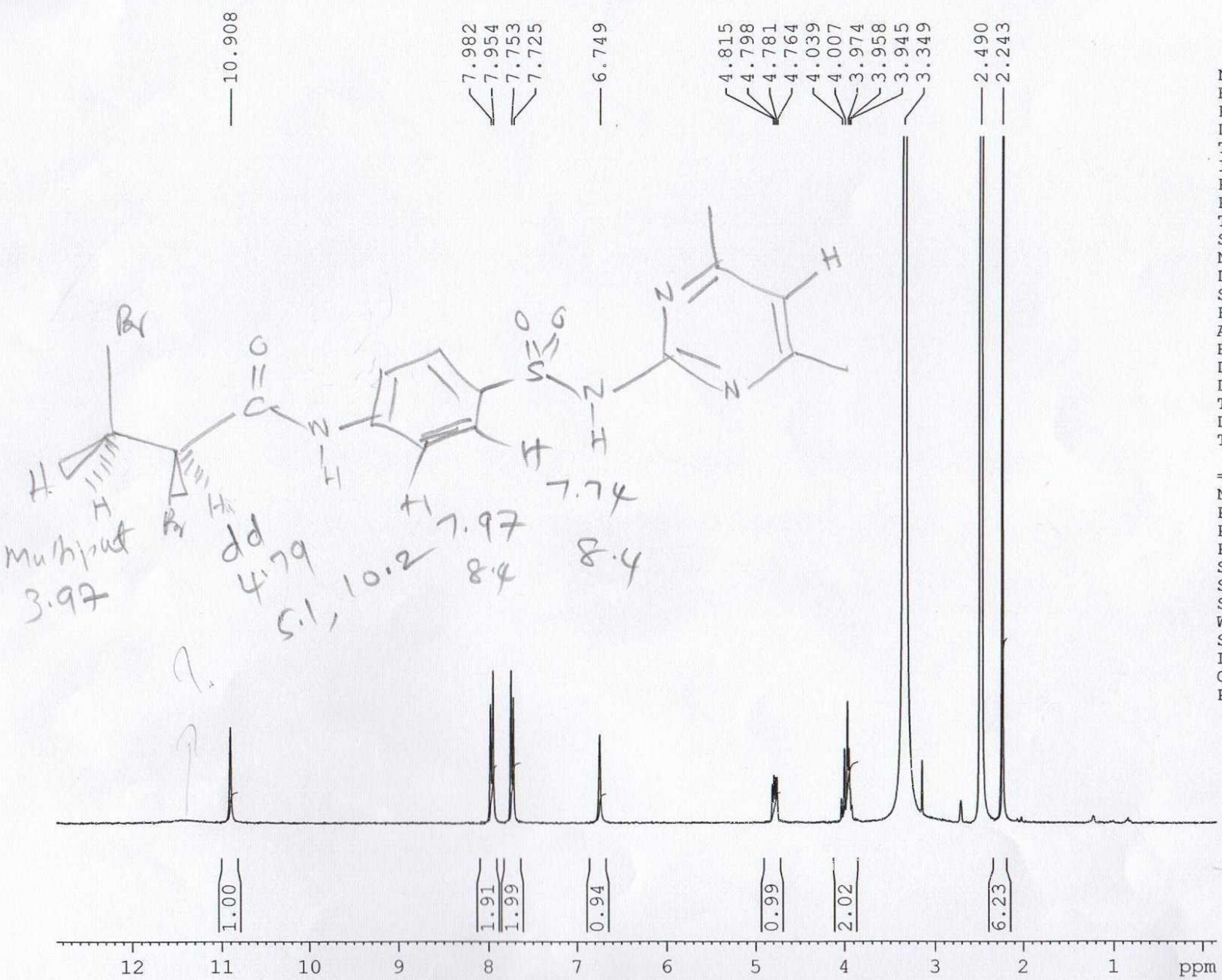

NAME	jan18-17
EXPNO	3
PROCNO	1
Date_	20170118
Time	12.43
INSTRUM	Spect
PROBHD	5 mm BBO BB-1H
PULPROG	zg30
TD	32768
SOLVENT	DMSO
NS	128
DS	0
SWH	6009.615 Hz
FIDRES	0.183399 Hz
AQ	2.7263477 sec
RG	203
DW	83.200 usec
DE	6.50 usec
TE	300.0 K
D1	1.50000000 sec
TD0	1

======== CHANNEL f1 ========

NUC1	1H
P1	12.50 usec
PL1	0.00 dB
PL1W	13.16228485 W
SFO1	300.1324010 MHz
SI	16384
SF	300.1300041 MHz
WDW	EM
SSB	0
LB	0.30 Hz
GB	0
PC	1.00

DR.HAROON/DR.HINA/MHH.I.39
ICCBS,U.O.K/1H

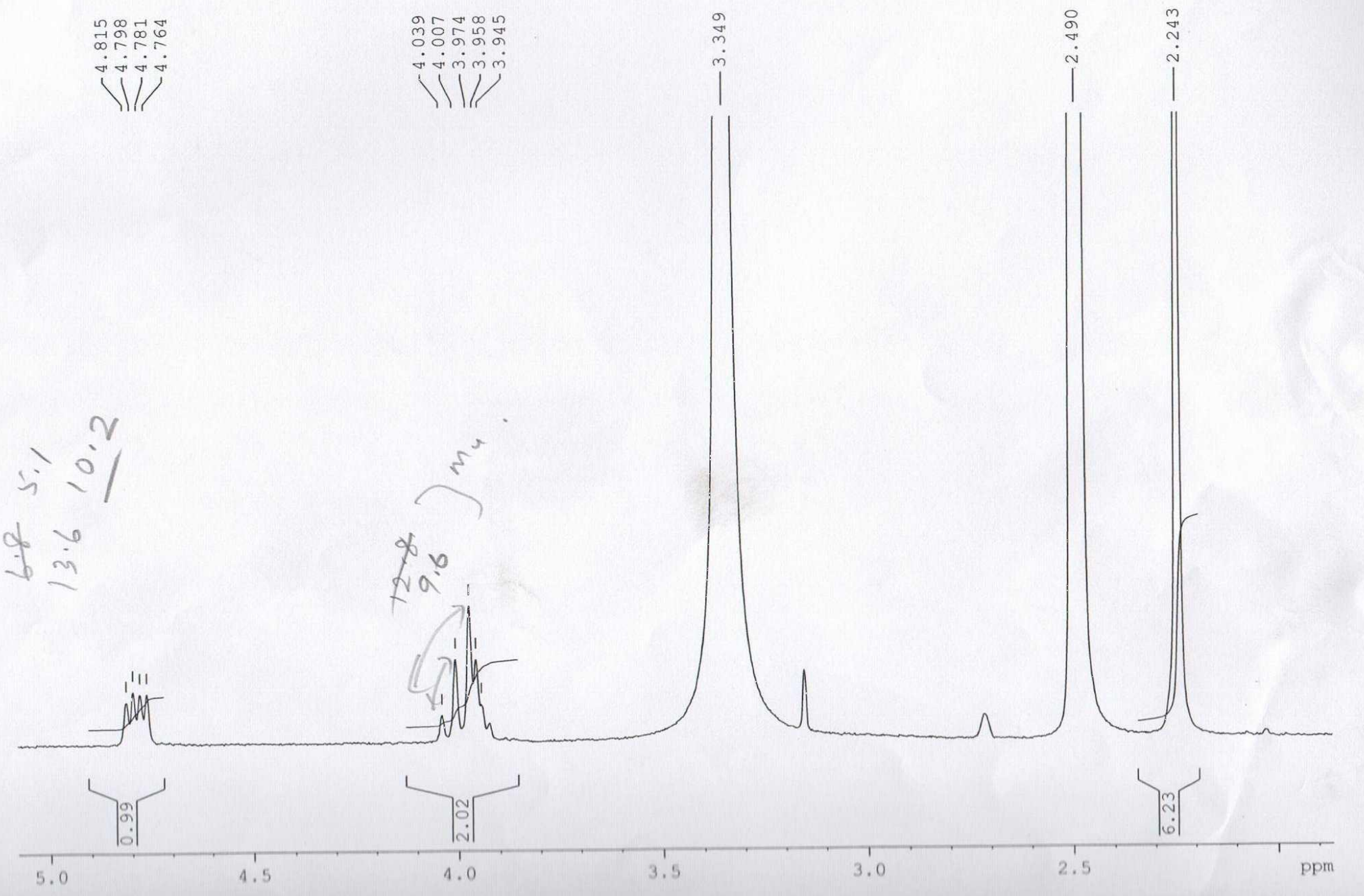

DR.HAROON/DR.HINA/MHH.I.39

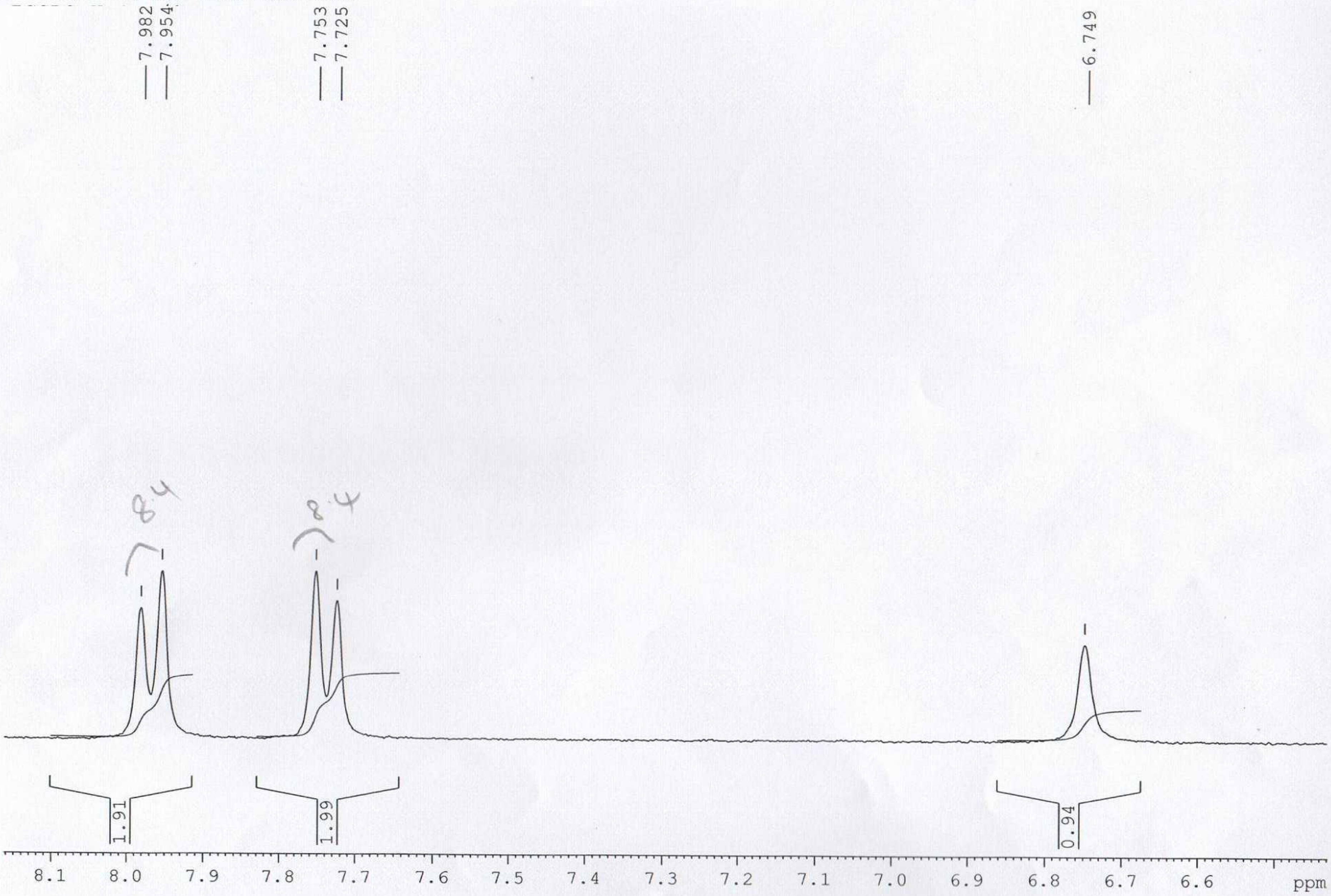

DR.M.H.HAROON/DR.HINA/MHH-1-39/DMSO
ICCBS,U.O.K/BB

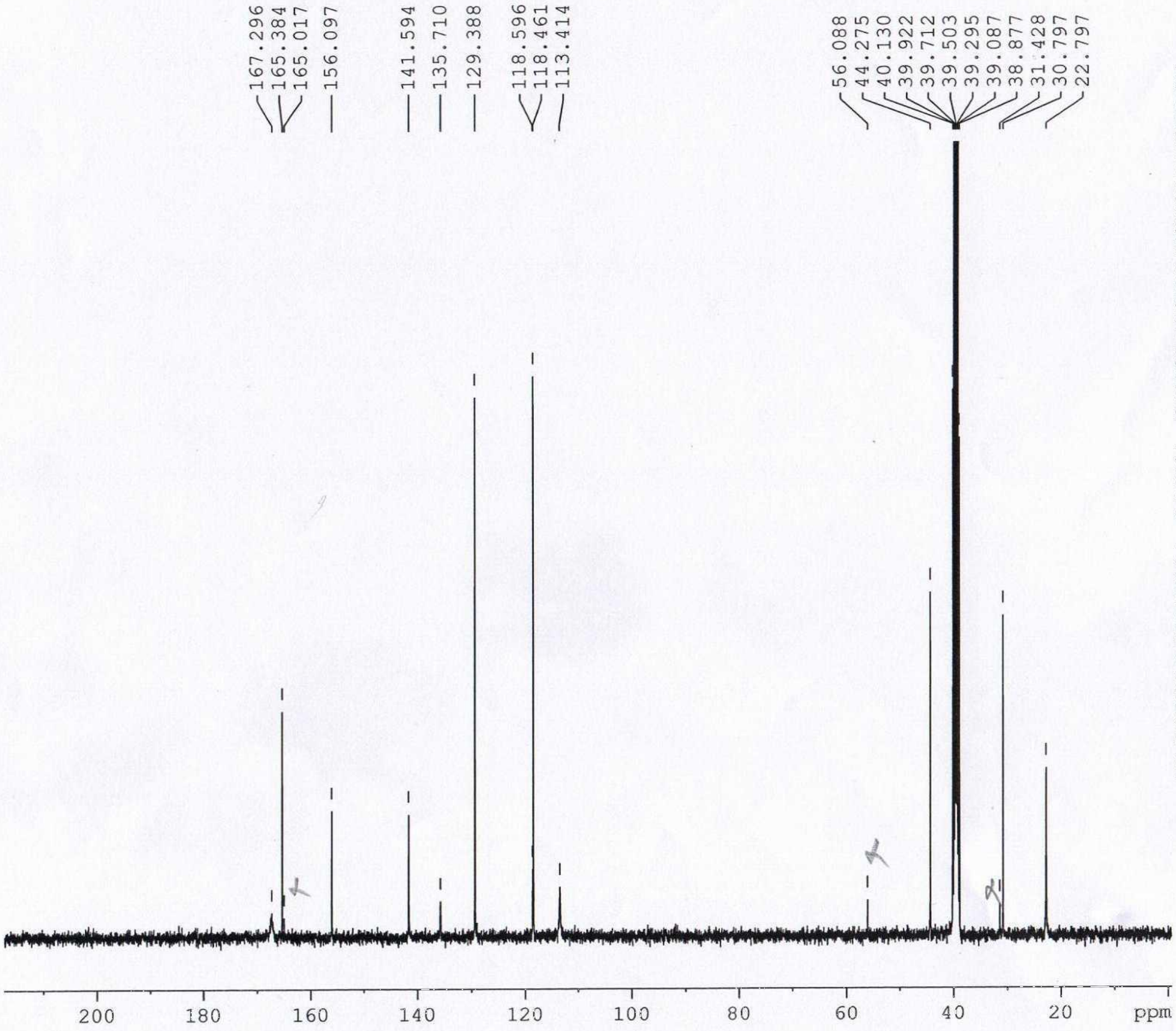

AVANCE 400
LAB NO 117

NAME	may01-17
EXPNO	1
PROCNO	1
Date_	20170501
Time	11.43
INSTRUM	spect
PROBHD	5 mm DUL 13C-1
PULPROG	zgpg
TD	32768
SOLVENT	DMSO
NS	18432
DS	0
SWH	24154.590 Hz
FIDRES	0.737140 Hz
AQ	0.6783476 sec
RG	32768
DW	20.700 usec
DE	6.50 usec
TE	300.0 K
D1	2.00000000 sec
D11	0.03000000 sec
TD0	18
======== CHANNEL f1 ========	
NUC1	13C
P1	8.55 usec
PL1	7.00 dB
SFO1	100.6243395 MHz
======== CHANNEL f2 ========	
CPDPRG2	waltz16
NUC2	1H
PCPD2	80.00 usec
PL2	0.00 dB
PL12	19.00 dB
PL13	20.00 dB
SFO2	400.1324008 MHz
SI	16384
SF	100.6128205 MHz
WDW	EM
SSB	0
LB	1.00 Hz
GB	0
PC	1.00

DR.M.H.HAROON/DR.HINA/MHH-1-39/DMSO
ICCBS,U.O.K/BB

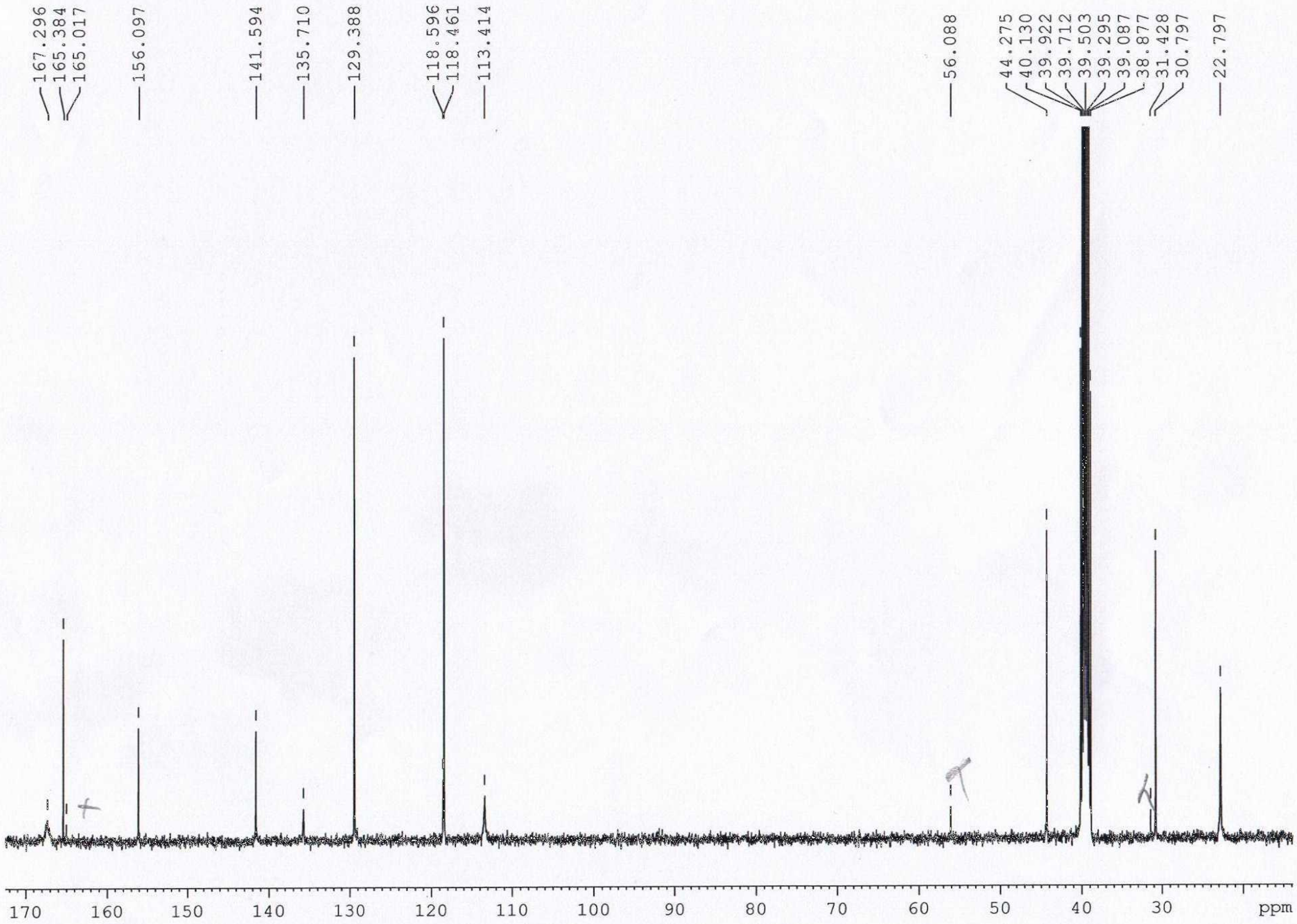

DR.M.H.HAROON/DR.HINA/MHH-1-39/DMSO
ICCBS,U.O.K/BB

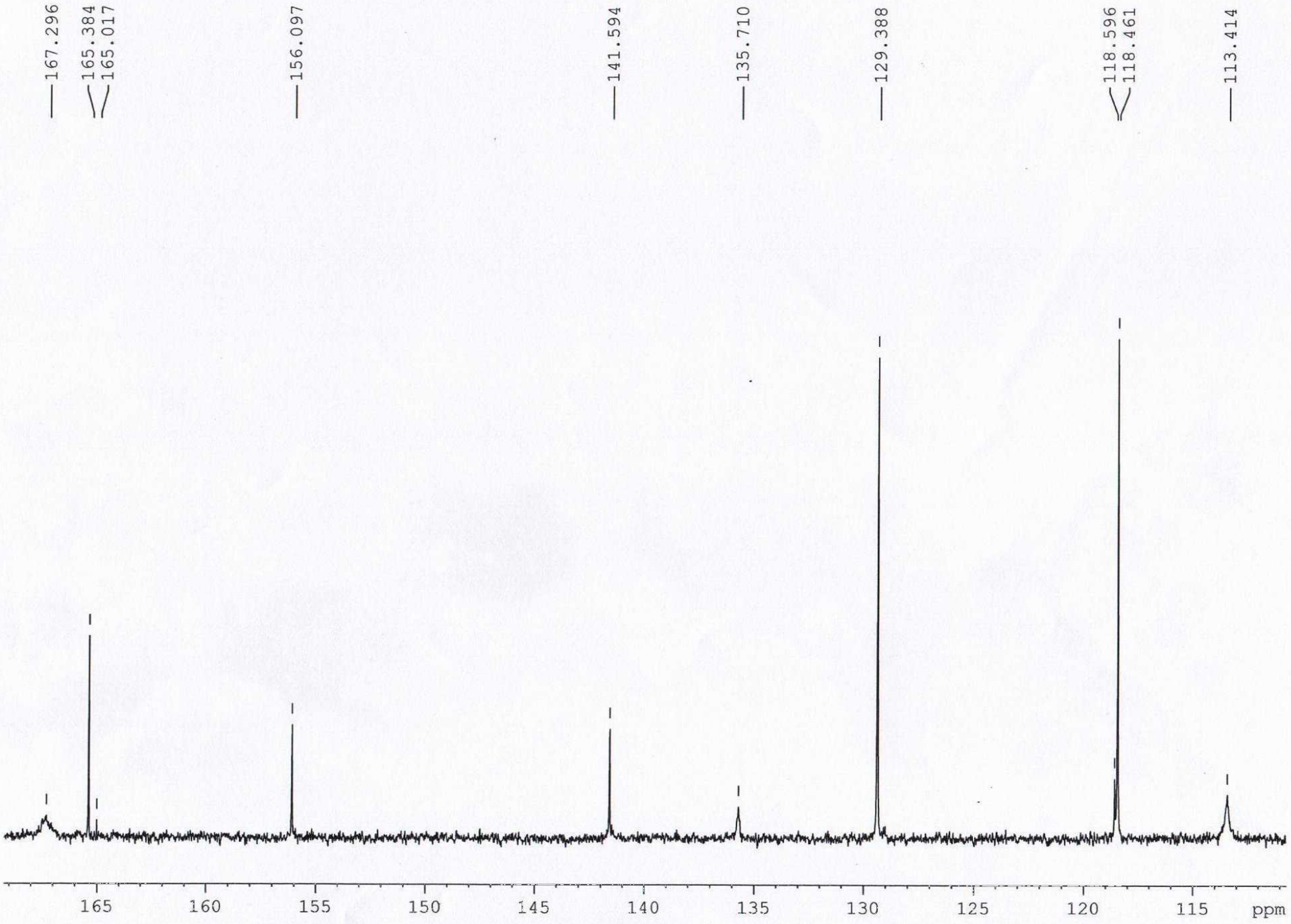

DR.M.H.HAROON/DR.HINA/MHH-1-39/DMSO
ICCBS,U.O.K/DEPT-135

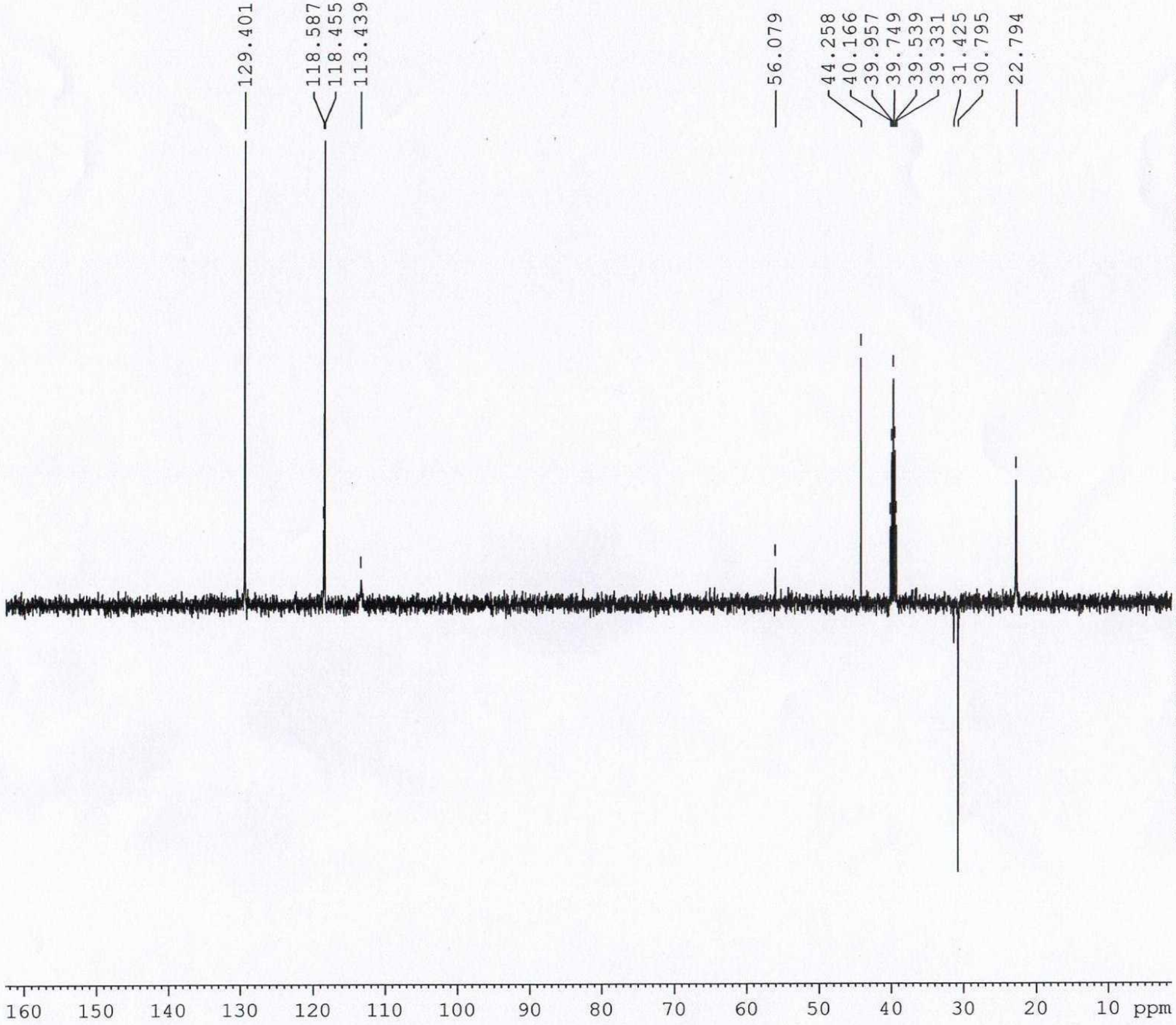

AVANCE 400
LAB NO 117

NAME	may01-17
EXPNO	2
PROCNO	1
Date_	20170502
Time	1.48
INSTRUM	spect
PROBHD	5 mm DUL 13C-1
PULPROG	dept135
TD	32768
SOLVENT	DMSO
NS	9216
DS	2
SWH	19157.088 Hz
FIDRES	0.584628 Hz
AQ	0.8552948 sec
RG	32768
DW	26.100 usec
DE	6.50 usec
TE	300.0 K
CNST2	145.0000000
D1	2.00000000 sec
D2	0.00344828 sec
D12	0.00002000 sec
TD0	9

======== CHANNEL f1 ========

NUC1	13C
P1	8.55 usec
P2	17.10 usec
PL1	7.00 dB
SFO1	100.6220254 MHz

======== CHANNEL f2 ========

CPDPRG2	waltz16
NUC2	1H
P3	9.50 usec
P4	19.00 usec
PCPD2	80.00 usec
PL2	0.00 dB
PL12	19.00 dB
SFO2	400.1320007 MHz
SI	16384
SF	100.6128205 MHz
WDW	EM
SSB	0
LB	1.00 Hz
GB	0
PC	1.40

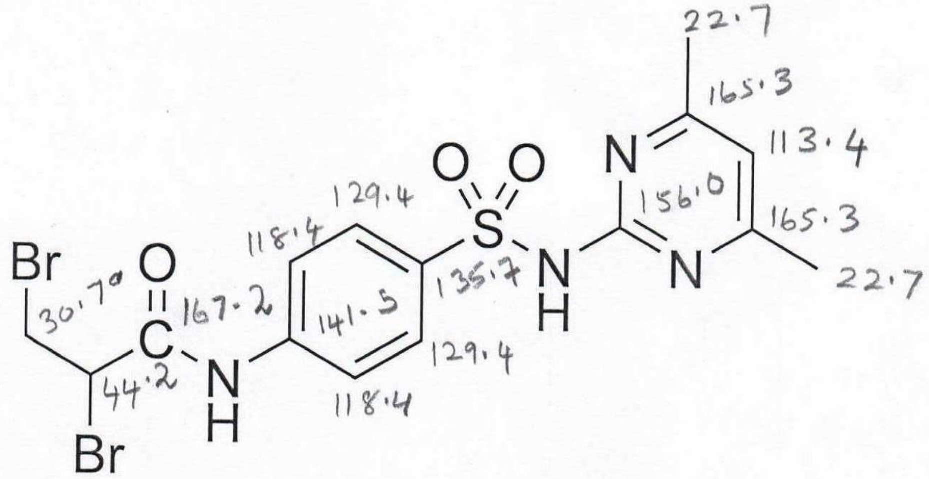
22.7
165.3
113.4
165.3
22.7
156.0
N
N
O
O
S
N
H
129.4
118.4
135.7
141.5
129.4
118.4
Br
O
30.7
167.2
44.2
N
H
Br

MHH-1-39

JEOL HX 110 MASS SPECTROMETER (FAB-HR)

STUDENT NAME	Dr. M. H. Haroon	SAMPLE CODE	DATE	25/8/17
SUPERVISOR NAME	Dr. Hina	MHH-I-39	FAB (+VE / -VE)	FAB+ve

Mass	Theoretical Mass	Delta [ppm]	Delta [mmu]	RDB	Composition
490.9410	490.9404	1.2	0.6	28.0	C_{30} H_6 N_1 Br_1 S_1
	490.9395	3.1	1.5	17.5	C_{23} H_{13} O_1 N_2 Br_2
	490.9428	-3.7	-1.8	12.5	C_{20} H_{17} O_1 N_2 Br_2 S_1
	490.9388	4.5	2.2	8.5	→ C_{15} H_{17} O_3 N_4 Br_2 S_1
	490.9378	6.6	3.2	23.5	C_{27} H_8 O_3 Br_1 S_1
492.9530	492.9527	0.6	0.3	32.0	C_{33} H_4 N_1 Br_1
	492.9534	-0.8	-0.4	22.5	C_{27} H_{10} O_3 Br_1 S_1
	492.9521	1.9	0.9	23.0	C_{25} H_8 O_2 N_3 Br_1 S_1
	492.9545	-3.0	-1.5	7.5	→ C_{15} H_{19} O_3 N_4 Br_2 S_1
	492.9511	3.9	1.9	12.5	C_{18} H_{15} O_3 N_4 Br_2

2/11/2017 3:27:33 PM

File: MHH-I-39
Sample: DR.M.H.HAROON /DR. HINA
Instrument: JEOL MS 600H-1

Date Run: 02-11-2017 (Time Run: 11:33:28)

Ionization mode: EI+

Scan: 12
Base: m/z 213; 11.6%FS TIC: 2375804

R.T.: .98

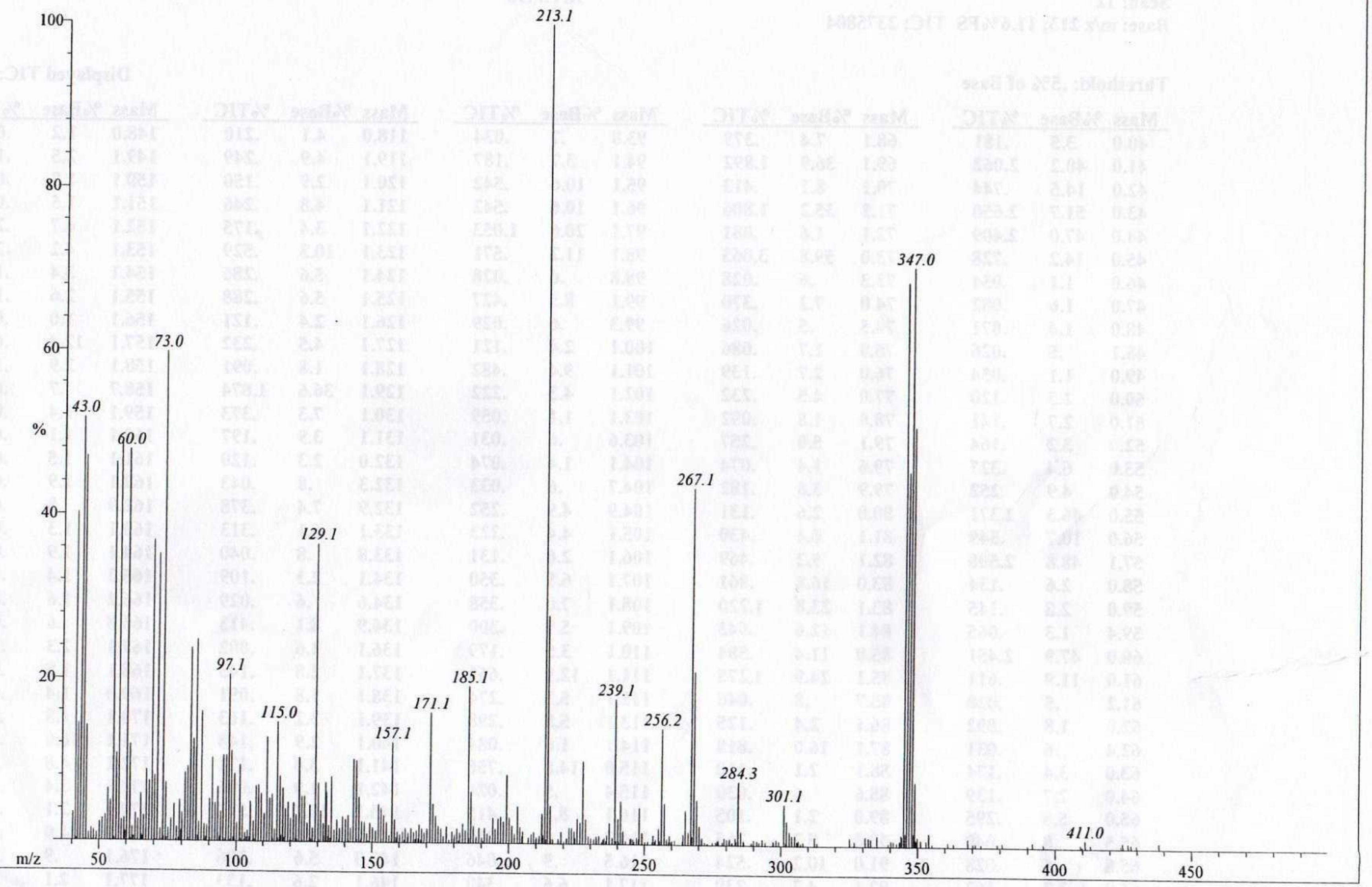

I. C.C.B.S., University of Karachi Analytical Laboratory - Pakistan

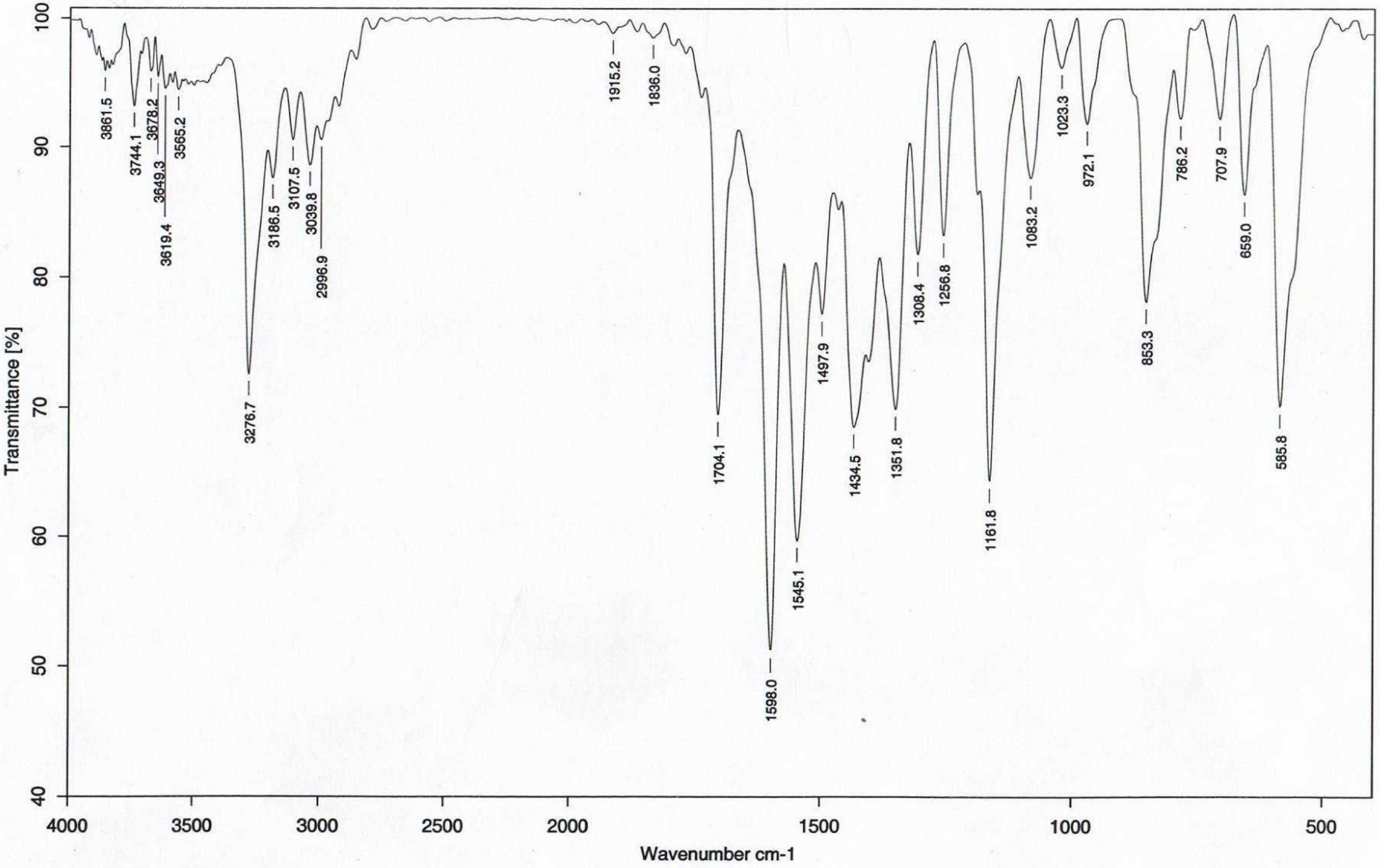

Sample : MHH-1-39/Dr.Haroon/Dr. Hina

Measured : 02/02/2017 on VECTOR22

Resolution : 4 cm-1 (10 scans)

Spectrum : MHH-1-39.0 (in D:\IRSTUDENT)

Technic : Solid

Analyst : Zubair Ahmad

THERMO ELECTRON ~ VISIONpro SOFTWARE V4.10

)perator Name	ARSHAD ALAM.	Date of Report	2/2/2017
)epartment	Analytical Laboratory TWC # 004	Time of Report	3:40:18PM
)rganization	ICCBS Karachi of Universty.		
nformation	Dr.Haroon/ Dr.Hina		

;can Graph

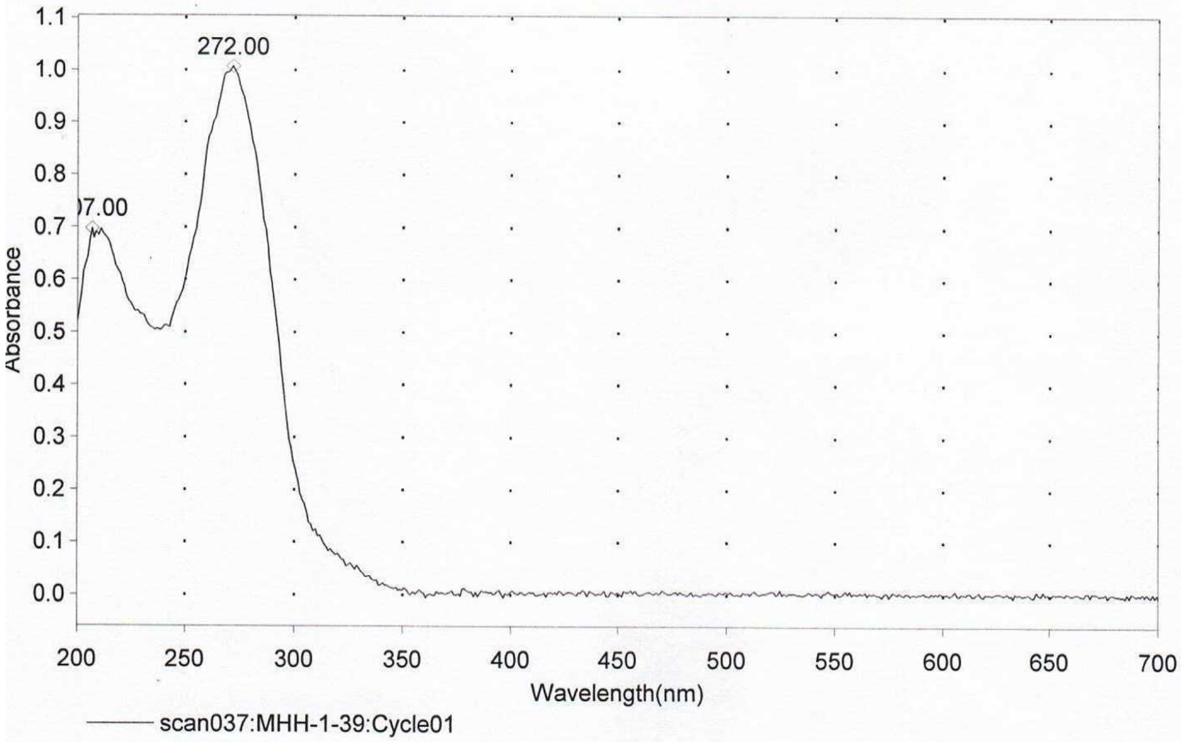

{esults Table - MHH-1-39.sre,MHH-1-39,Cycle01

ım	A	**Peak Pick Method**
:07.00	0.699	Find 8 Peaks Above -3.0000 A
:72.00	1.007	Start Wavelength 200.00 nm
		Stop Wavelength 700.00 nm
		Sort By Wavelength

;ensitivity Auto
