# Supplementary material for: Sulphamethazine derivatives as immunomodulating agents: New therapeutic strategies for inflammatory diseases
Source: PLoS One. 2018 Dec 19;13(12):e0208933. doi: 10.1371/journal.pone.0208933 (PMC6300282; doi:10.1371/journal.pone.0208933)
Supplement: S2 Fig — (PDF) [file pone.0208933.s002.pdf]

AVANCE AV-400 MHz
Lab # 115

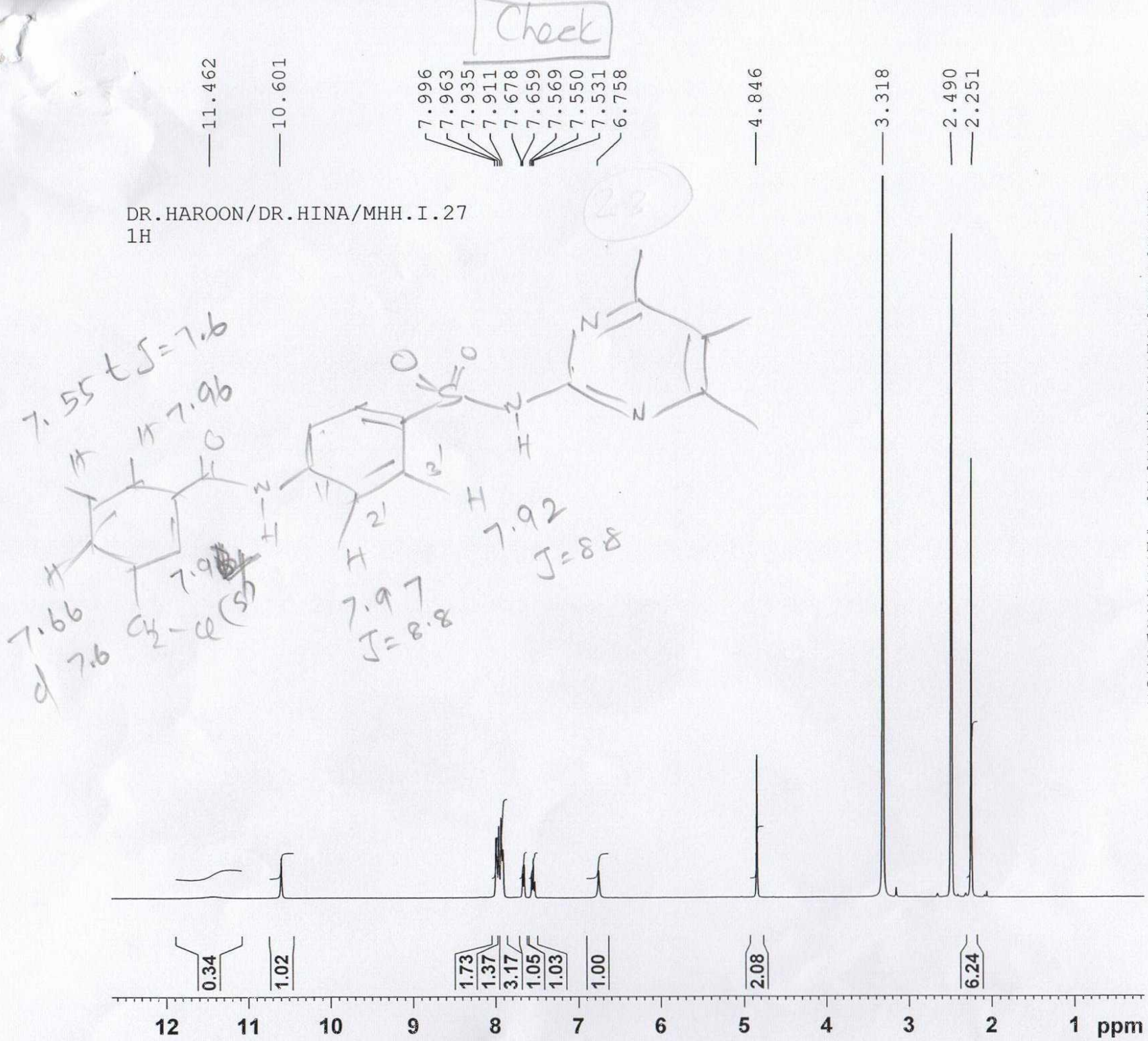

NAME	jan03-17
EXPNO	3
PROCNO	1
Date_	20170103
Time	11.35
INSTRUM	spect
PROBHD	5 mm SEI 1H-13
PULPROG	zg30
TD	65536
SOLVENT	DMSO
NS	64
DS	0
SWH	8012.820 Hz
FIDRES	0.122266 Hz
AQ	4.0894966 sec
RG	512
DW	62.400 usec
DE	6.50 usec
TE	300.0 K
D1	2.00000000 sec
TD0	1

======== CHANNEL f1 ========

NUC1	1H
P1	10.80 usec
PL1	3.00 dB
SFO1	400.0332002 MHz
SI	32768
SF	400.0300041 MHz
WDW	EM
SSB	0
LB	0.30 Hz
GB	0
PC	1.00

(23)

DR.HAROON/DR.HINA/MHH.I.27
1H

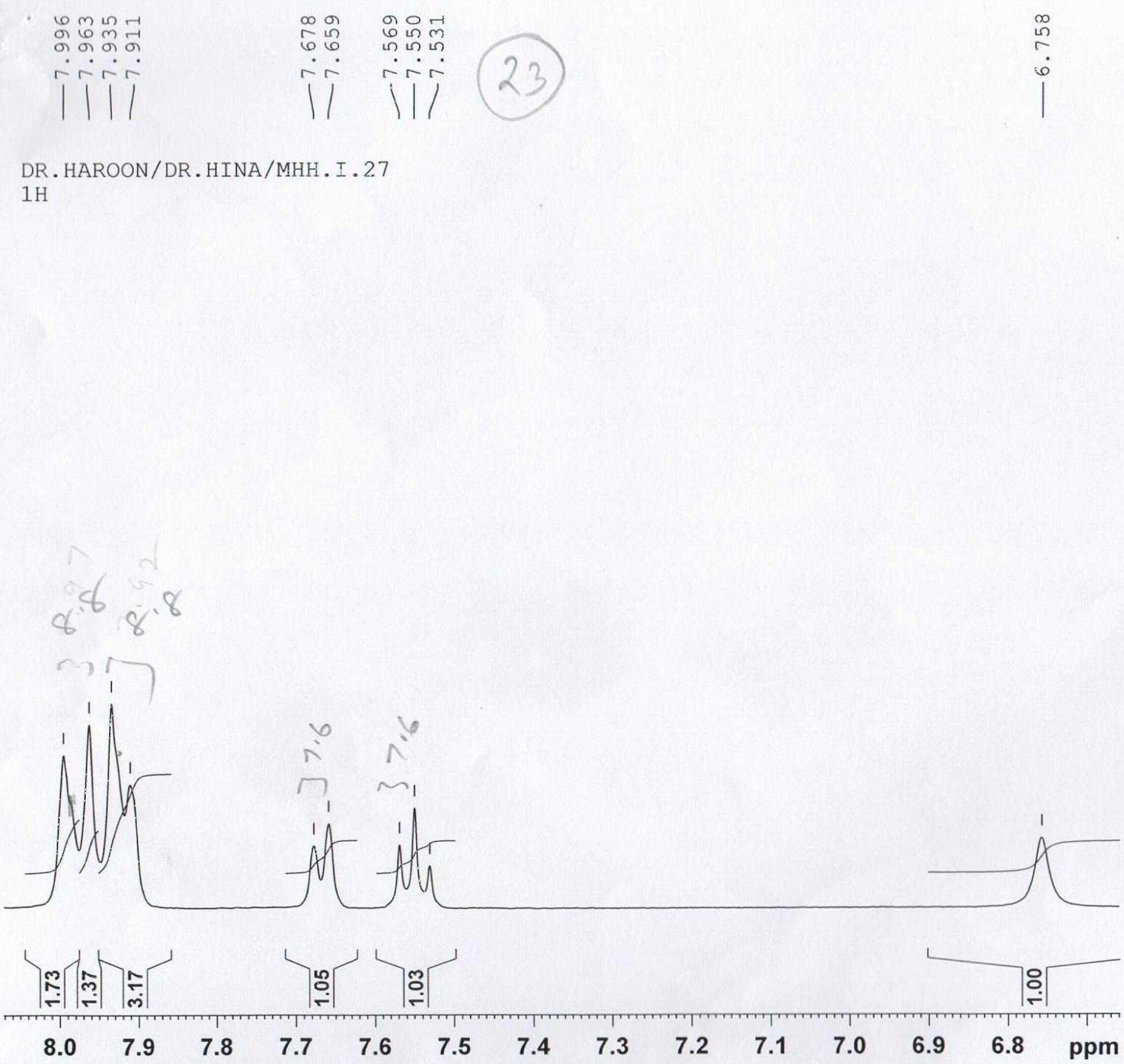

DR.M.H.HAROON/DR.HINA/MHH-1-27/DMSO
ICCBS,U.O.K/BB

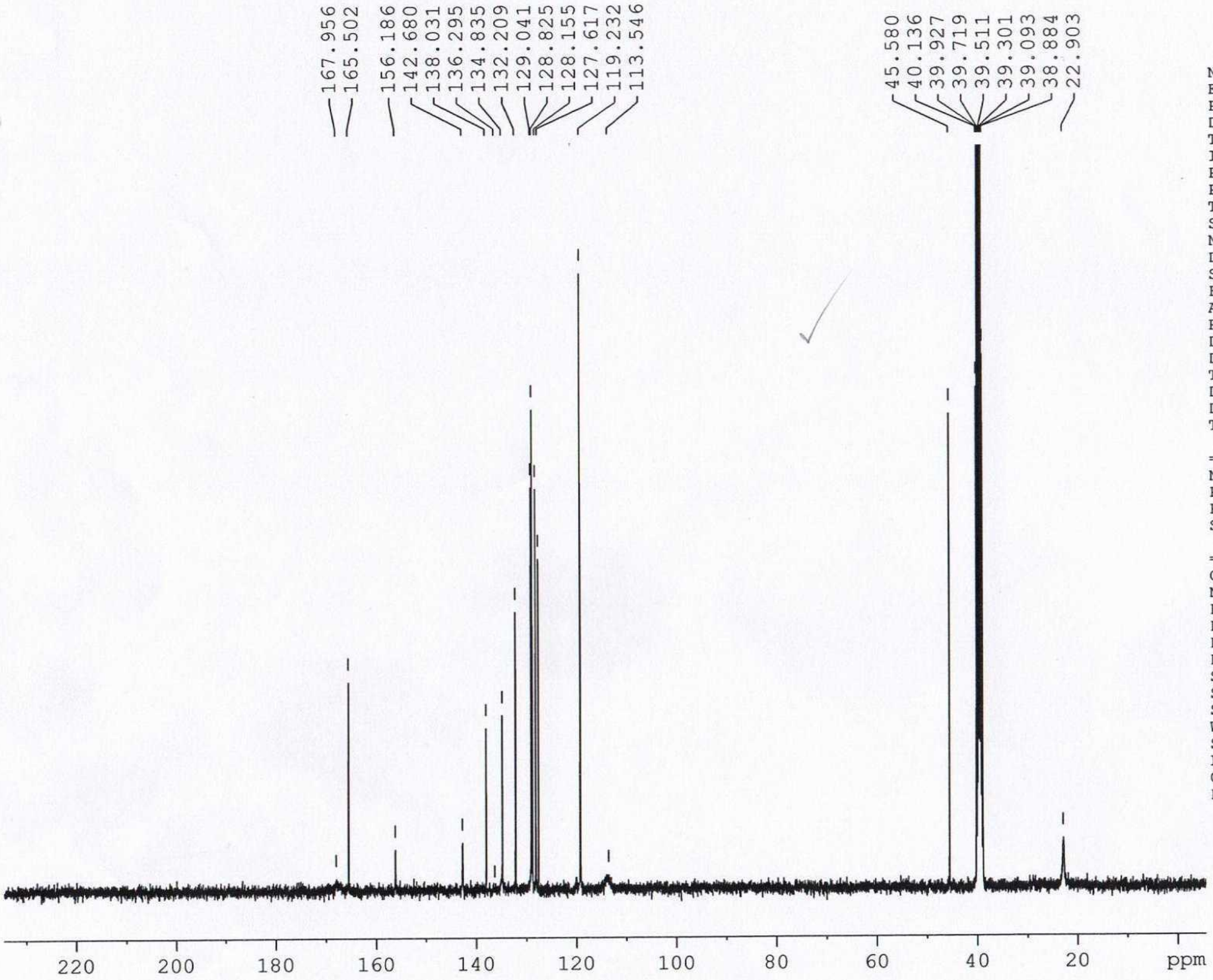

AVANCE 400
LAB NO 117

NAME	apr28-17
EXPNO	1
PROCNO	1
Date_	20170428
Time	13.14
INSTRUM	spect
PROBHD	5 mm DUL 13C-1
PULPROG	zgpg
TD	32768
SOLVENT	DMSO
NS	20480
DS	0
SWH	24154.590 Hz
FIDRES	0.737140 Hz
AQ	0.6783476 sec
RG	32768
DW	20.700 usec
DE	6.50 usec
TE	300.0 K
D1	2.00000000 sec
D11	0.03000000 sec
TD0	20

======== CHANNEL f1 ========

NUC1	13C
P1	8.55 usec
PL1	7.00 dB
SFO1	100.6243395 MHz

======== CHANNEL f2 ========

CPDPRG2	waltz16
NUC2	1H
PCPD2	80.00 usec
PL2	0.00 dB
PL12	19.00 dB
PL13	20.00 dB
SFO2	400.1324008 MHz
SI	16384
SF	100.6128205 MHz
WDW	EM
SSB	0
LB	1.00 Hz
GB	0
PC	1.00

DR.M.H.HAROON/DR.HINA/MHH-1-27/DMSO
ICCBS,U.O.K/BB

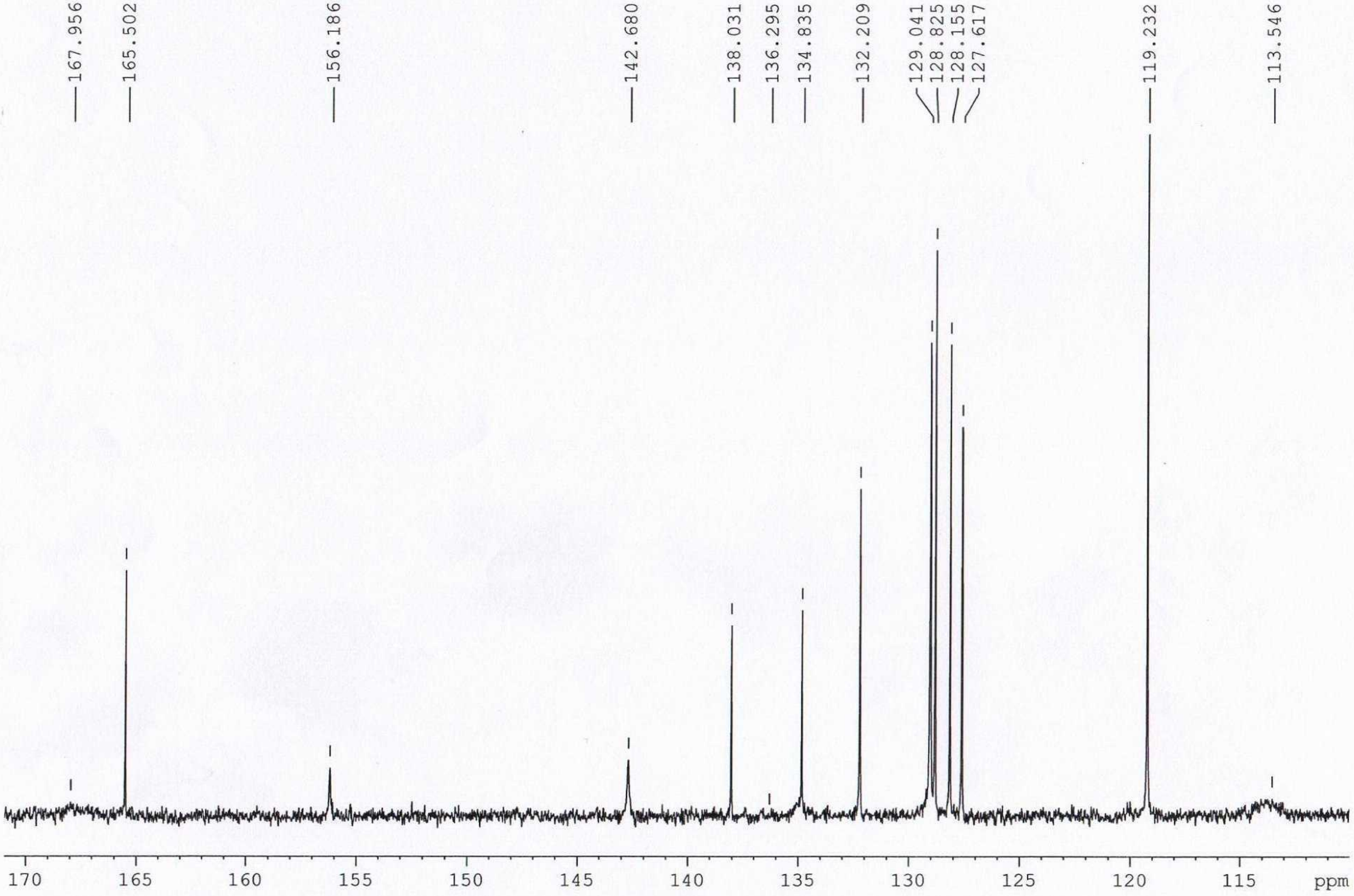

DR.M.H.HAROON/DR.HINA/MHH-1-27/DMSO
ICCBS,U.O.K/DEPT-135

AVANCE 400
LAB NO 117

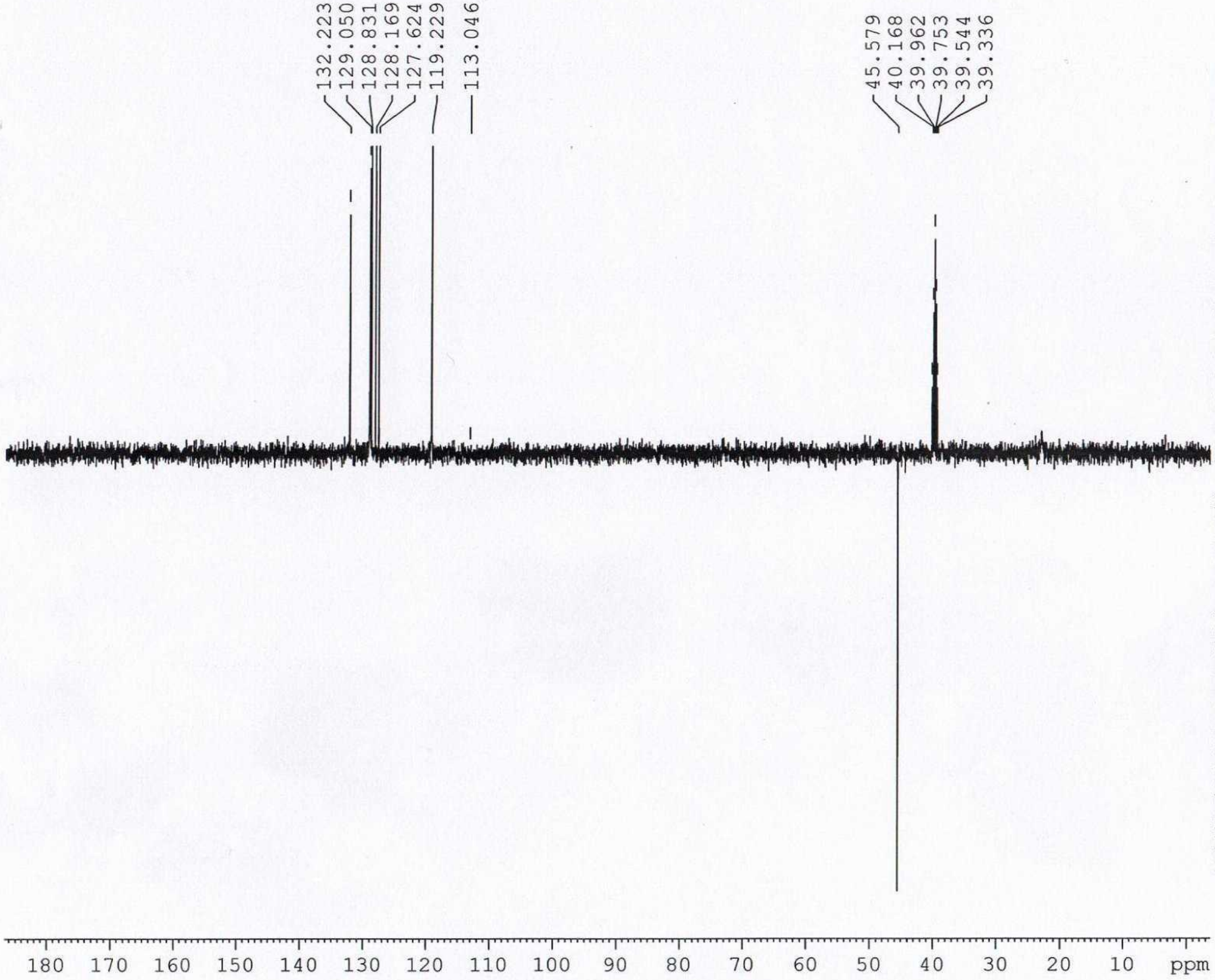

NAME	apr28-17
EXPNO	2
PROCNO	1
Date_	20170429
Time	4.53
INSTRUM	spect
PROBHD	5 mm DUL 13C-1
PULPROG	dept135
TD	32768
SOLVENT	DMSO
NS	7143
DS	2
SWH	19157.088 Hz
FIDRES	0.584628 Hz
AQ	0.8552948 sec
RG	32768
DW	26.100 usec
DE	6.50 usec
TE	300.0 K
CNST2	145.0000000
D1	2.00000000 sec
D2	0.00344828 sec
D12	0.00002000 sec
TD0	9

======== CHANNEL f1 ========

NUC1	13C
P1	8.55 usec
P2	17.10 usec
PL1	7.00 dB
SFO1	100.6220254 MHz

======== CHANNEL f2 ========

CPDPRG2	waltz16
NUC2	1H
P3	9.50 usec
P4	19.00 usec
PCPD2	80.00 usec
PL2	0.00 dB
PL12	19.00 dB
SFO2	400.1320007 MHz
SI	16384
SF	100.6128205 MHz
WDW	EM
SSB	0
LB	1.00 Hz
GB	0
PC	1.40

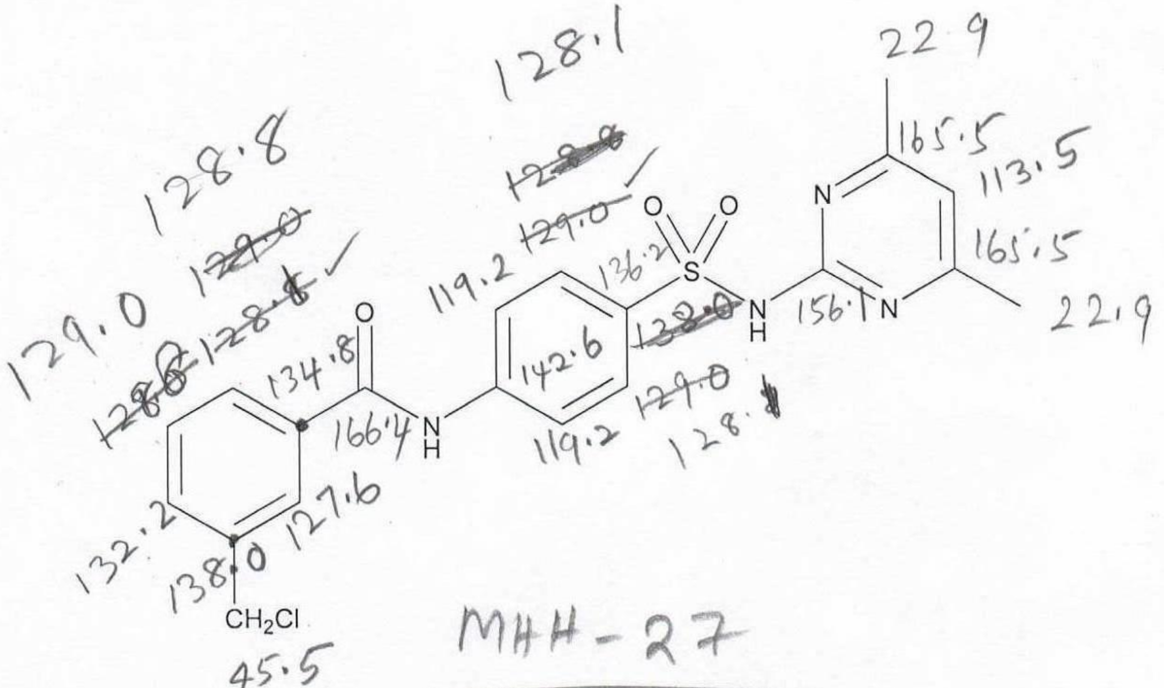

MHH-27

Dept -135°

135.4

Rnd

~~134~~

JEOL HX 110 MASS SPECTROMETER (FAB-HR)

STUDENT NAME	Dr. M. H. Haroon	SAMPLE CODE	DATE	28/5/17
SUPERVISOR NAME	Dr. Hina	MHH-I-27	FAB (+VE / -VE)	FAB +Ve

Mass	Theoretical Mass	Delta [ppm]	Delta [mmu]	RDB	Composition
431.0955	431.0951	0.9	0.4	21.5	$C_{28}\ H_{16}\ O_1\ N_2\ Cl_1$
	431.0946	2.0	0.9	26.0	$C_{31}\ H_{13}\ O_2\ N_1$
	431.0945	2.4	1.0	12.5	$C_{20}\ H_{20}\ O_3\ N_4\ Cl_1\ S_1$ ✓
	431.0967	-2.7	-1.2	21.5	$C_{26}\ H_{15}\ O_1\ N_4\ S_1$
	431.0933	5.1	2.2	26.5	$C_{29}\ H_{11}\ O_1\ N_4$
	431.0980	-5.8	-2.5	21.0	$C_{28}\ H_{17}\ O_2\ N_1\ S_1$
	431.0985	-6.9	-3.0	16.5	$C_{25}\ H_{20}\ O_1\ N_2\ Cl_1\ S_1$
	431.0911	10.2	4.4	17.5	$C_{23}\ H_{16}\ O_3\ N_4\ Cl_1$
	431.0894	14.0	6.1	25.5	$C_{32}\ H_{15}\ S_1$

Observed

mass

calculated

formula.

430.0866

$C_{20}H_{19}ClN_4O_3S$

431.0946

File: MHH-I-27-FABP
Sample: DR.M.H.HAROON /DR. HINA
Instrument: JEOL-600H-2
Inlet: Direct Probe

Date Run: 05-20-2017 (Time Run: 11:01:25)

Ionization mode: FAB+

Scan: 2
Base: m/z 185; 100%FS TIC: 2227484

R.T.: .1

#Ions: 810

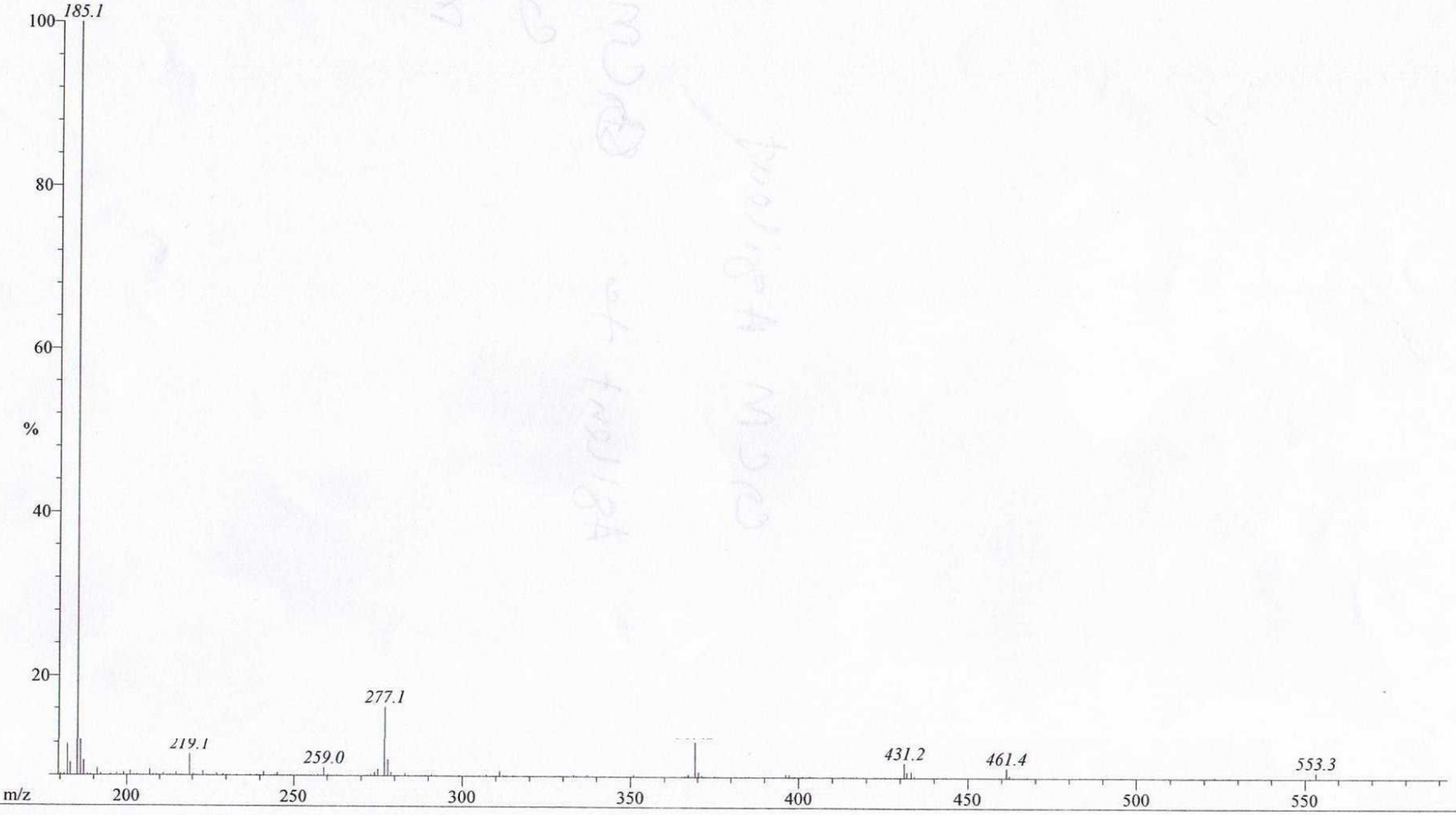

I. C.C.B.S., University of Karachi Analytical Laboratory - Pakistan

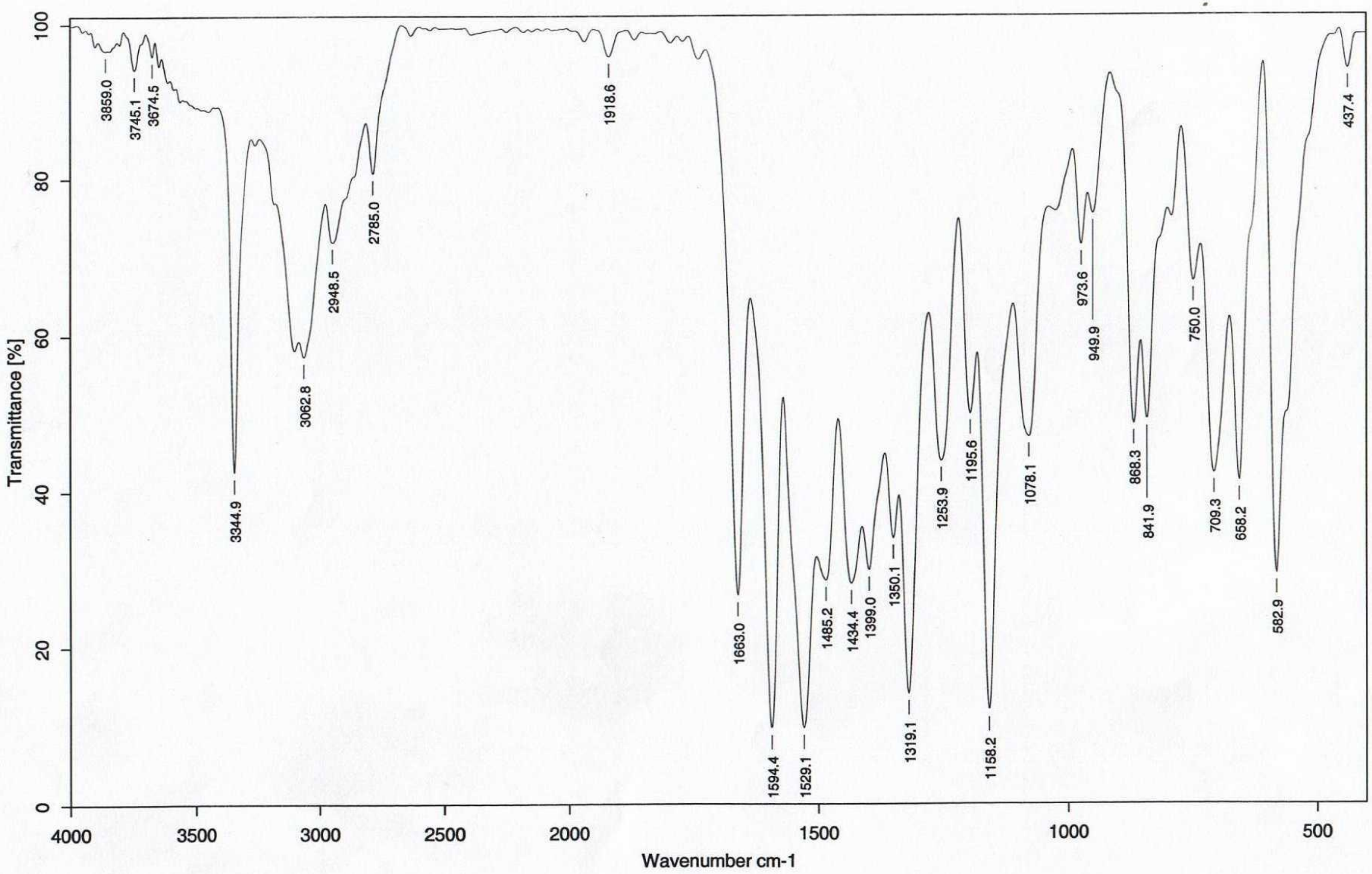

Sample : MHH-1-27/Dr.Haroon

Spectrum : MHH-1-27.0 (in D:\IRSTUDENT)

Measured : 27/01/2017 on VECTOR22

Technic : Liquid

Resolution : 4 cm-1 (10 scans)

Analyst : M. Asif

THERMO ELECTRON ~ VISIONpro SOFTWARE V4.10

Operator Name	ARSHAD ALAM.	Date of Report	1/30/2017
Department	Analytical Laboratory TWC # 004	Time of Report	10:23:41AM
Organization	ICCBS Karachi of Universty.		
Information	Dr Haron/Dr Hina		

Scan Graph

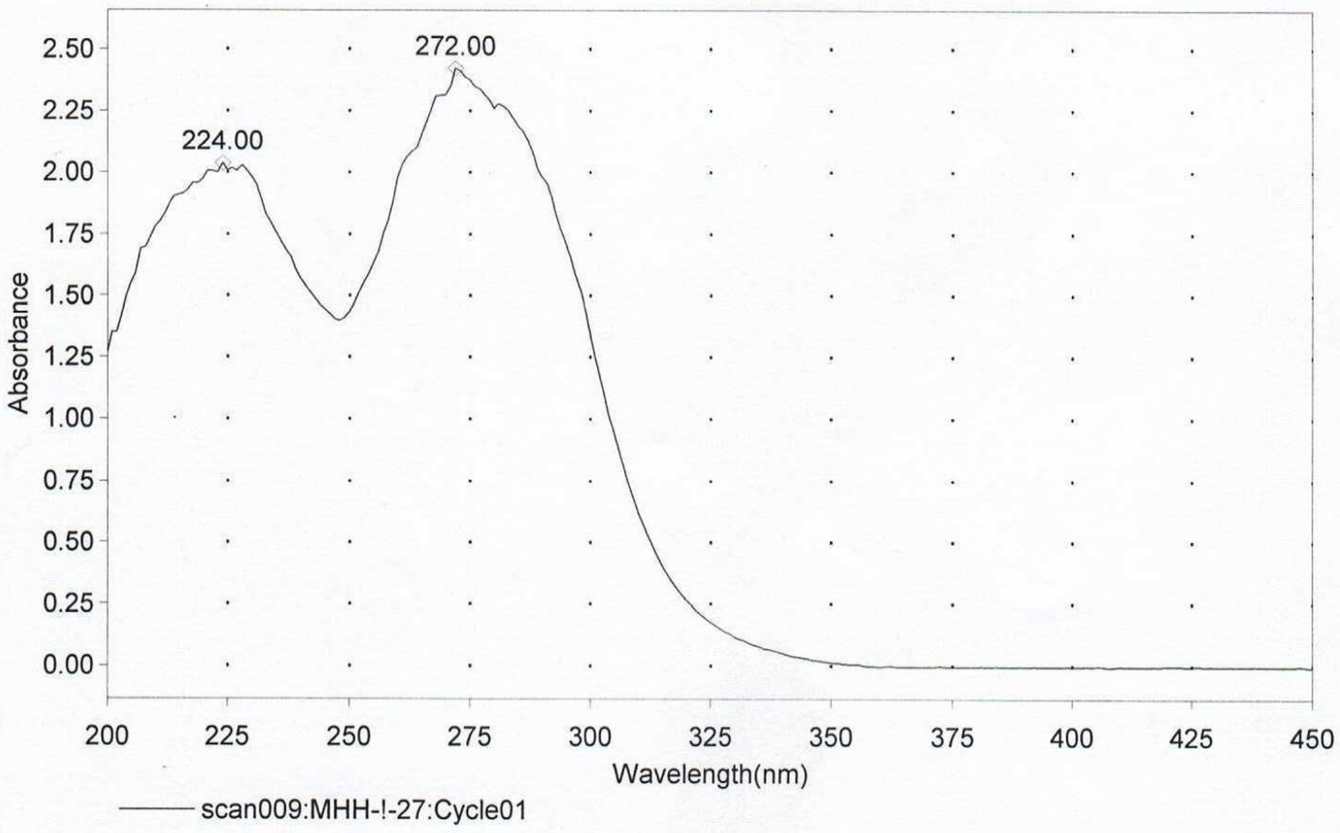

Results Table - MHH-1-27.sre,MHH-!-27,Cycle01

nm	A	**Peak Pick Method**
224.00	2.035	Find 8 Peaks Above -3.0000 A
272.00	2.422	Start Wavelength 200.00 nm
		Stop Wavelength 450.00 nm
		Sort By Wavelength

Sensitivity Auto
