# Supplementary material for: Sulphamethazine derivatives as immunomodulating agents: New therapeutic strategies for inflammatory diseases
Source: PLoS One. 2018 Dec 19;13(12):e0208933. doi: 10.1371/journal.pone.0208933 (PMC6300282; doi:10.1371/journal.pone.0208933)
Supplement: S3 Fig — (PDF) [file pone.0208933.s003.pdf]

DR.HAROON/DR.HINA/MHH.I.28
1H

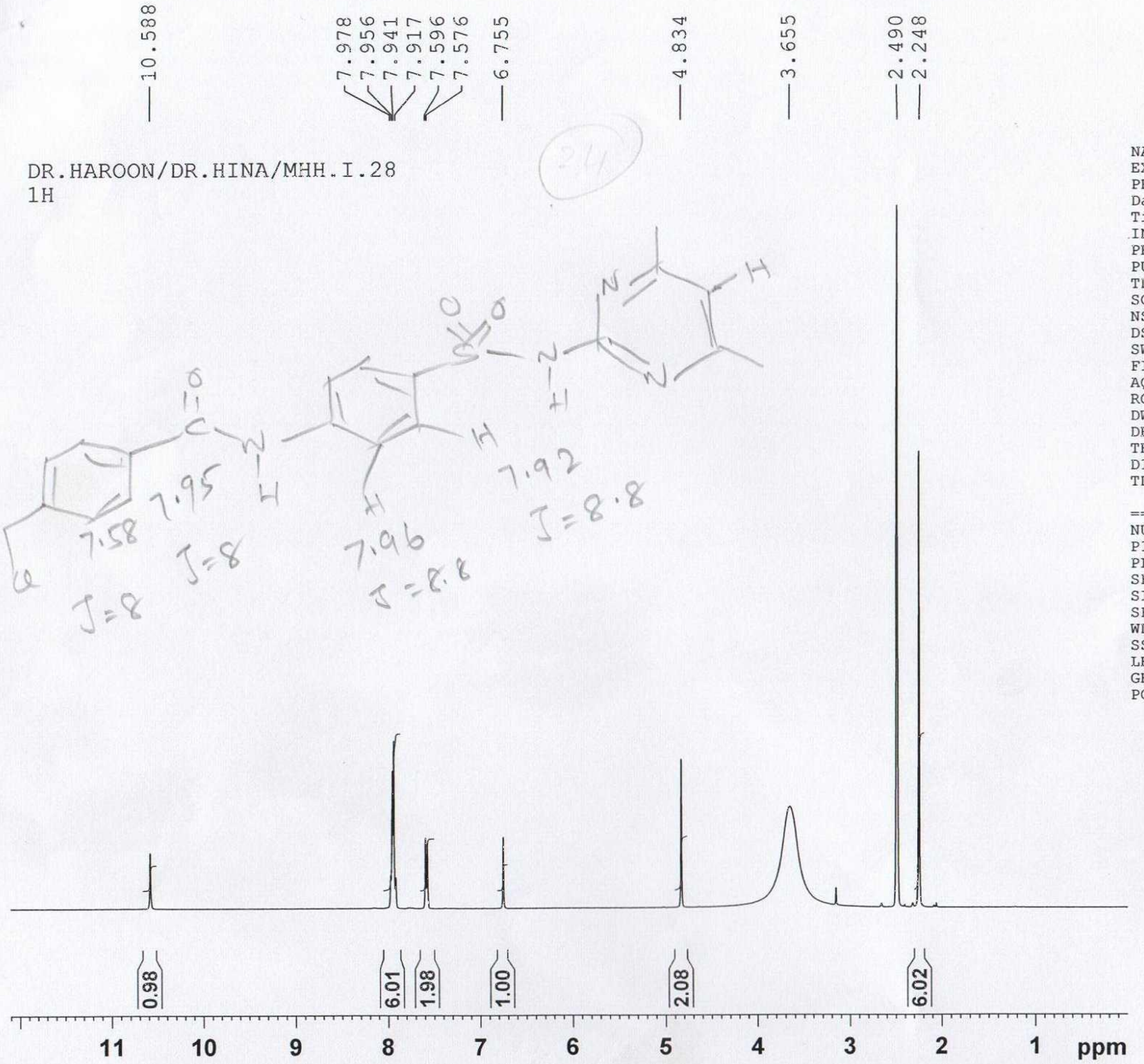

AVANCE AV-400 MHz
Lab # 115

NAME	jan03-17
EXPNO	4
PROCNO	1
Date_	20170103
Time	11.46
INSTRUM	spect
PROBHD	5 mm SEI 1H-13
PULPROG	zg30
TD	65536
SOLVENT	DMSO
NS	64
DS	0
SWH	8012.820 Hz
FIDRES	0.122266 Hz
AQ	4.0894966 sec
RG	512
DW	62.400 usec
DE	6.50 usec
TE	300.0 K
D1	2.00000000 sec
TD0	1

======== CHANNEL f1 ========

NUC1	1H
P1	10.80 usec
PL1	3.00 dB
SFO1	400.0332002 MHz
SI	32768
SF	400.0300041 MHz
WDW	EM
SSB	0
LB	0.30 Hz
GB	0
PC	1.00

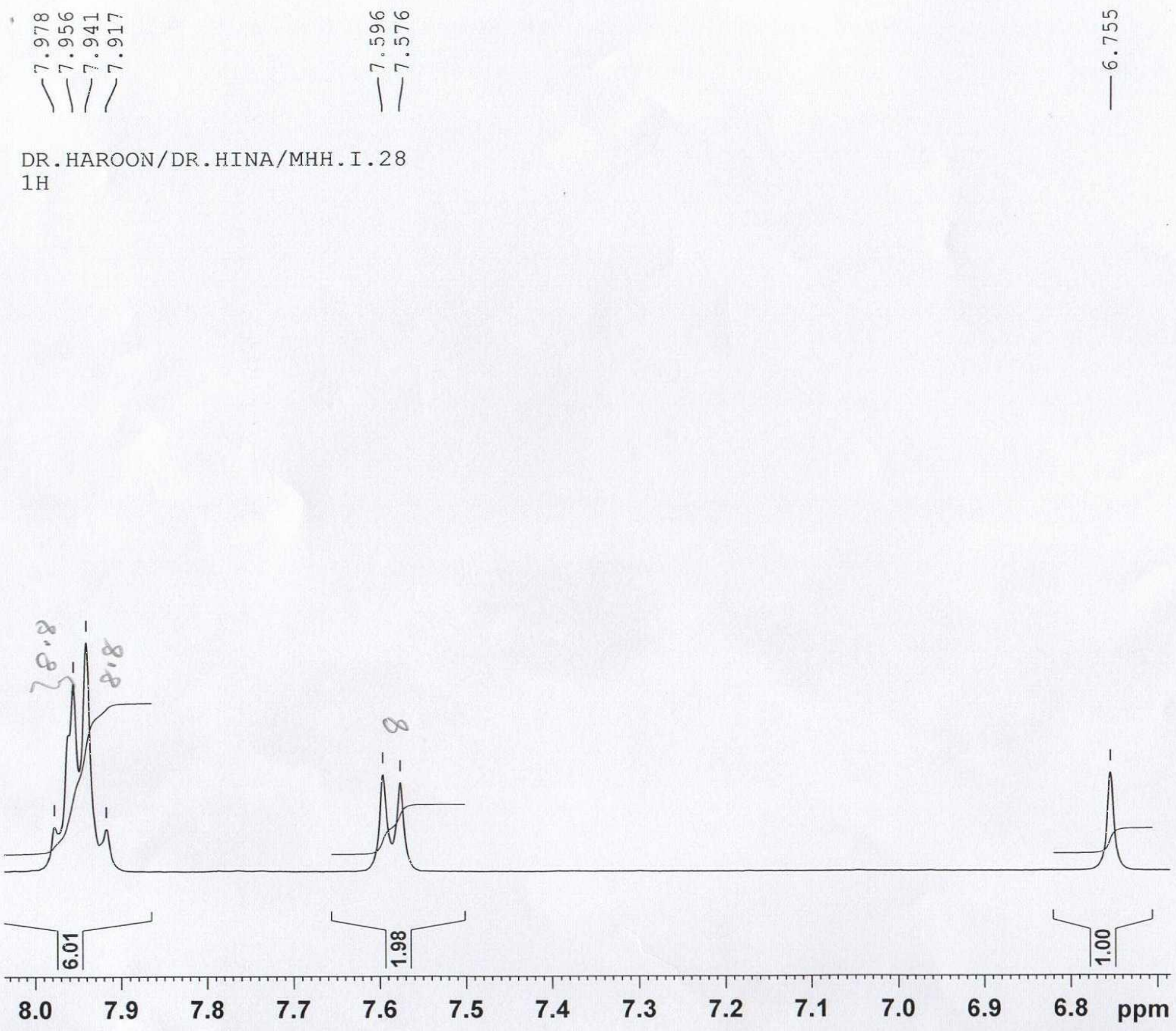
7.978
7.956
7.941
7.917
7.596
7.576
6.755
DR.HAROON/DR.HINA/MHH.I.28
1H
6.01
1.98
1.00
8.0
7.9
7.8
7.7
7.6
7.5
7.4
7.3
7.2
7.1
7.0
6.9
6.8
ppm

DR.M.H.HAROON/DR.HINA/MHH-1-28/DMSO
ICCBS,U.O.K/BB

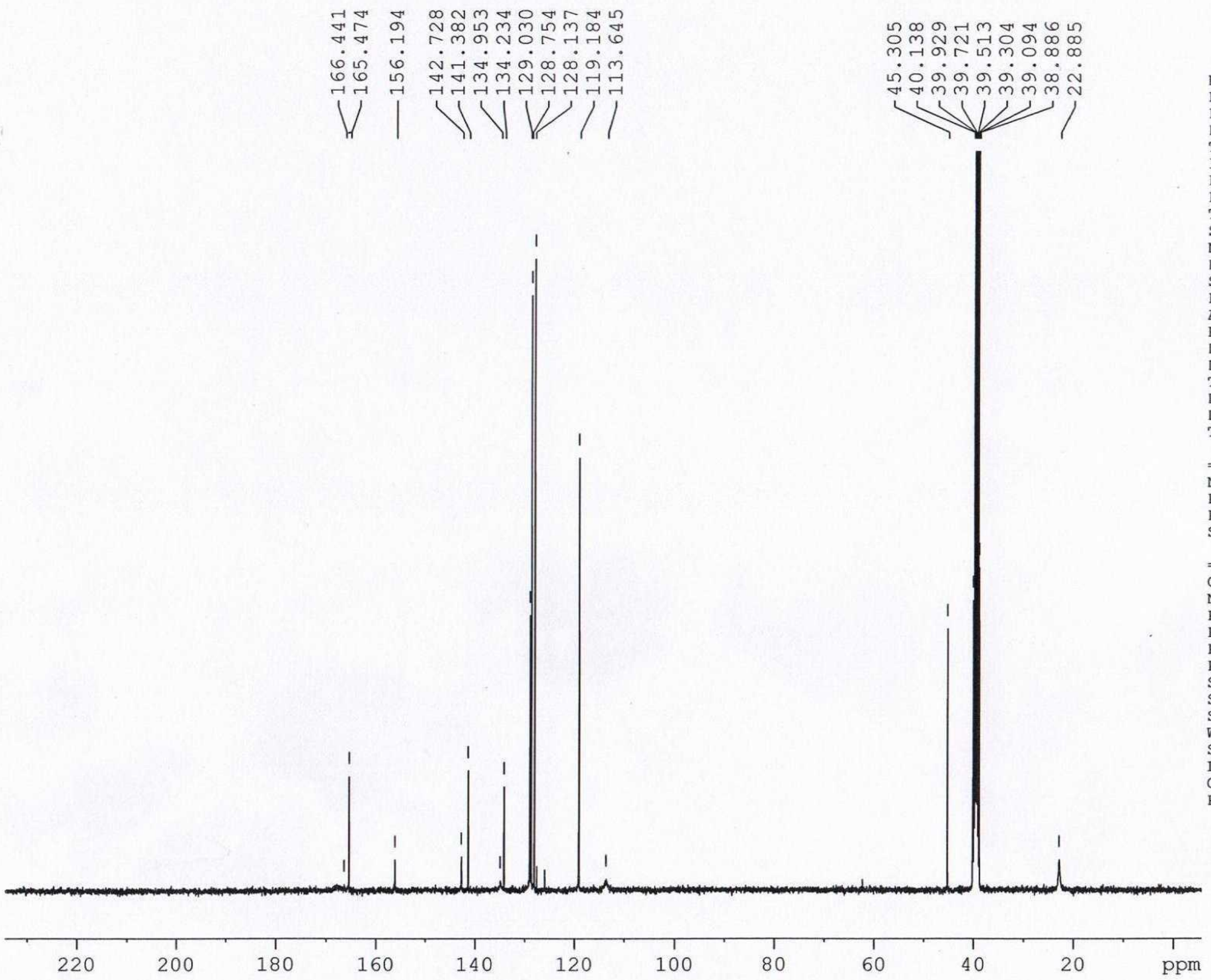

AVANCE 400
LAB NO 117

NAME	apr26-17
EXPNO	11
PROCNO	1
Date_	20170426
Time	16.04
INSTRUM	spect
PROBHD	5 mm DUL 13C-1
PULPROG	zgpg
TD	32768
SOLVENT	DMSO
NS	18432
DS	0
SWH	24154.590 Hz
FIDRES	0.737140 Hz
AQ	0.6783476 sec
RG	32768
DW	20.700 usec
DE	6.50 usec
TE	300.0 K
D1	2.00000000 sec
D11	0.03000000 sec
TD0	18

======== CHANNEL f1 ========

NUC1	13C
P1	8.55 usec
PL1	7.00 dB
SFO1	100.6243395 MHz

======== CHANNEL f2 ========

CPDPRG2	waltz16
NUC2	1H
PCPD2	80.00 usec
PL2	0.00 dB
PL12	19.00 dB
PL13	20.00 dB
SFO2	400.1324008 MHz
SI	16384
SF	100.6128205 MHz
WDW	EM
SSB	0
LB	1.00 Hz
GB	0
PC	1.00

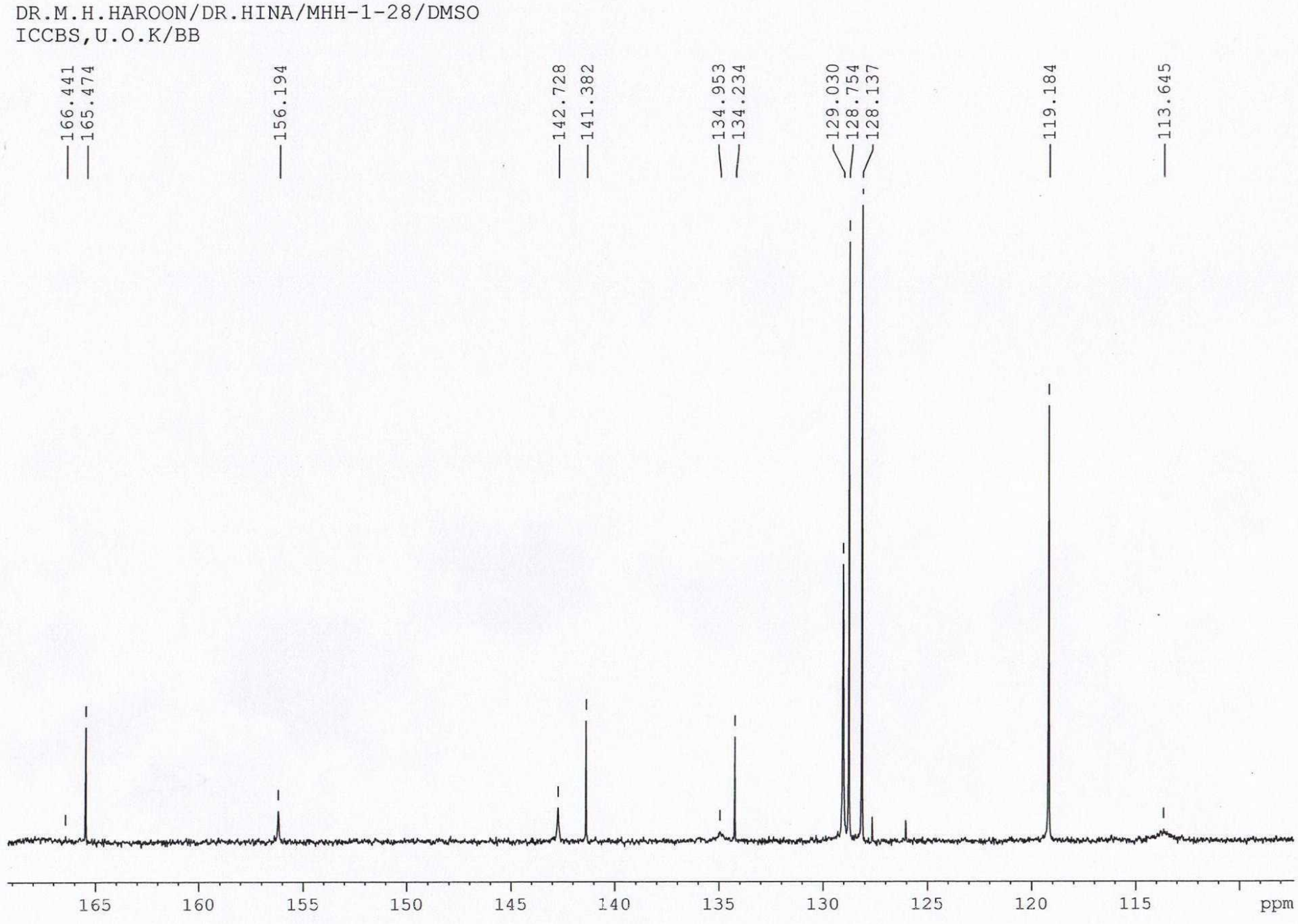

DR.M.H.HAROON/DR.HINA/MHH-1-28/DMSO
ICCBS,U.O.K/BB
166.441
165.474
156.194
142.728
141.382
134.953
134.234
129.030
128.754
128.137
119.184
113.645
165
160
155
150
145
140
135
130
125
120
115
ppm

DR.M.H.HAROON/DR.HINA/MHH-1-28/DMSO
ICCBS,U.O.K/DEPT-135

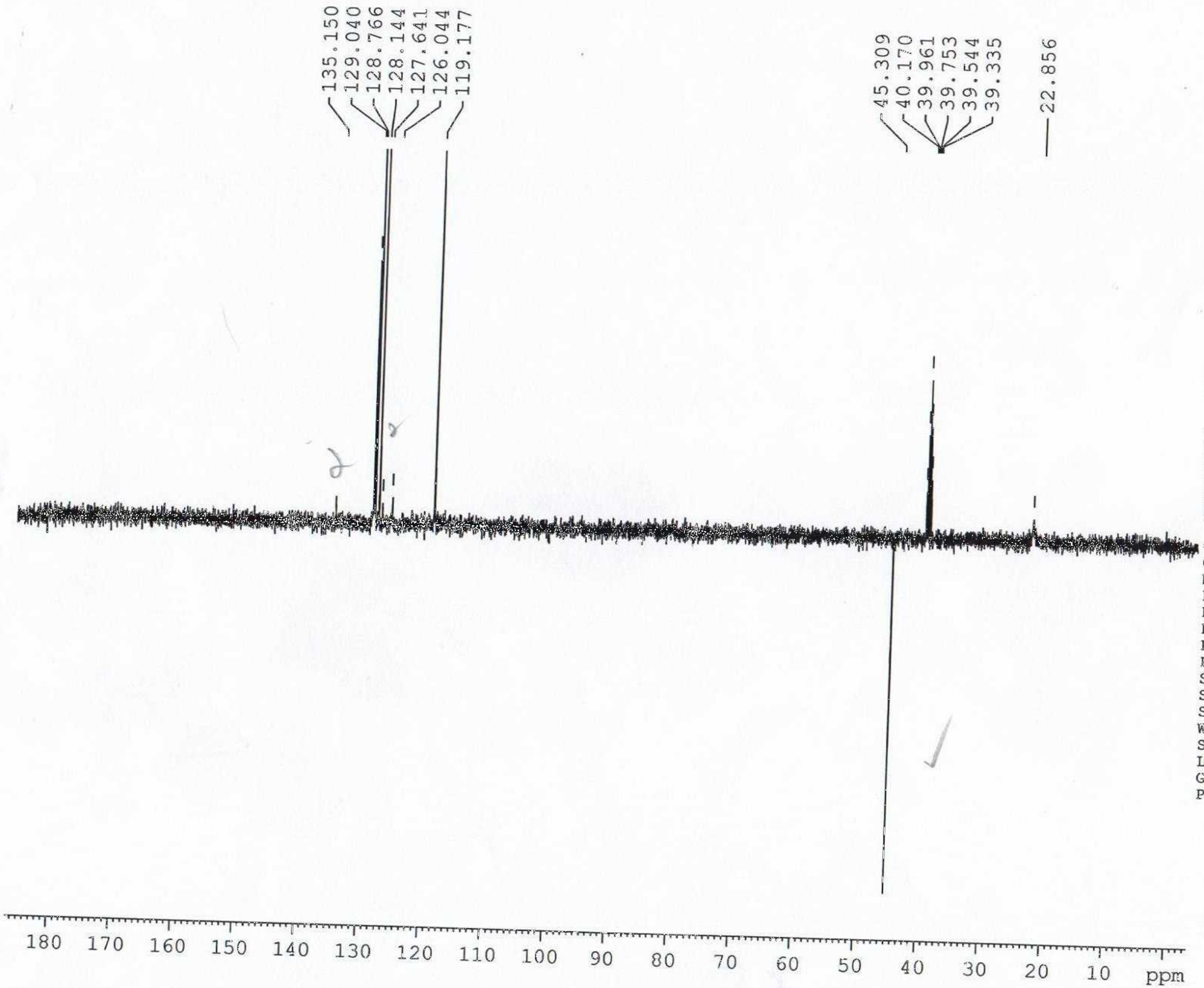

AVANCE 400
LAB NO 117

NAME	apr26-17
EXPNO	12
PROCNO	1
Date_	20170427
Time	6.43
INSTRUM	spect
PROBHD	5 mm DUL 13C-1
PULPROG	dept135
TD	32768
SOLVENT	DMSO
NS	4308
DS	2
SWH	19157.088 Hz
FIDRES	0.584628 Hz
AQ	0.8552948 sec
RG	32768
DW	26.100 usec
DE	6.50 usec
TE	300.0 K
CNST2	145.0000000
D1	2.00000000 sec
D2	0.00344828 sec
D12	0.00002000 sec
TD0	8

======== CHANNEL f1 ========

NUC1	13C
P1	8.55 usec
P2	17.10 usec
PL1	7.00 dB
SFO1	100.6220254 MHz

======== CHANNEL f2 ========

CPDPRG2	waltz16
NUC2	1H
P3	9.50 usec
P4	19.00 usec
PCPD2	80.00 usec
PL2	0.00 dB
PL12	19.00 dB
SFO2	400.1320007 MHz
SI	16384
SF	100.6128205 MHz
WDW	EM
SSB	0
LB	1.00 Hz
GB	0
PC	1.40

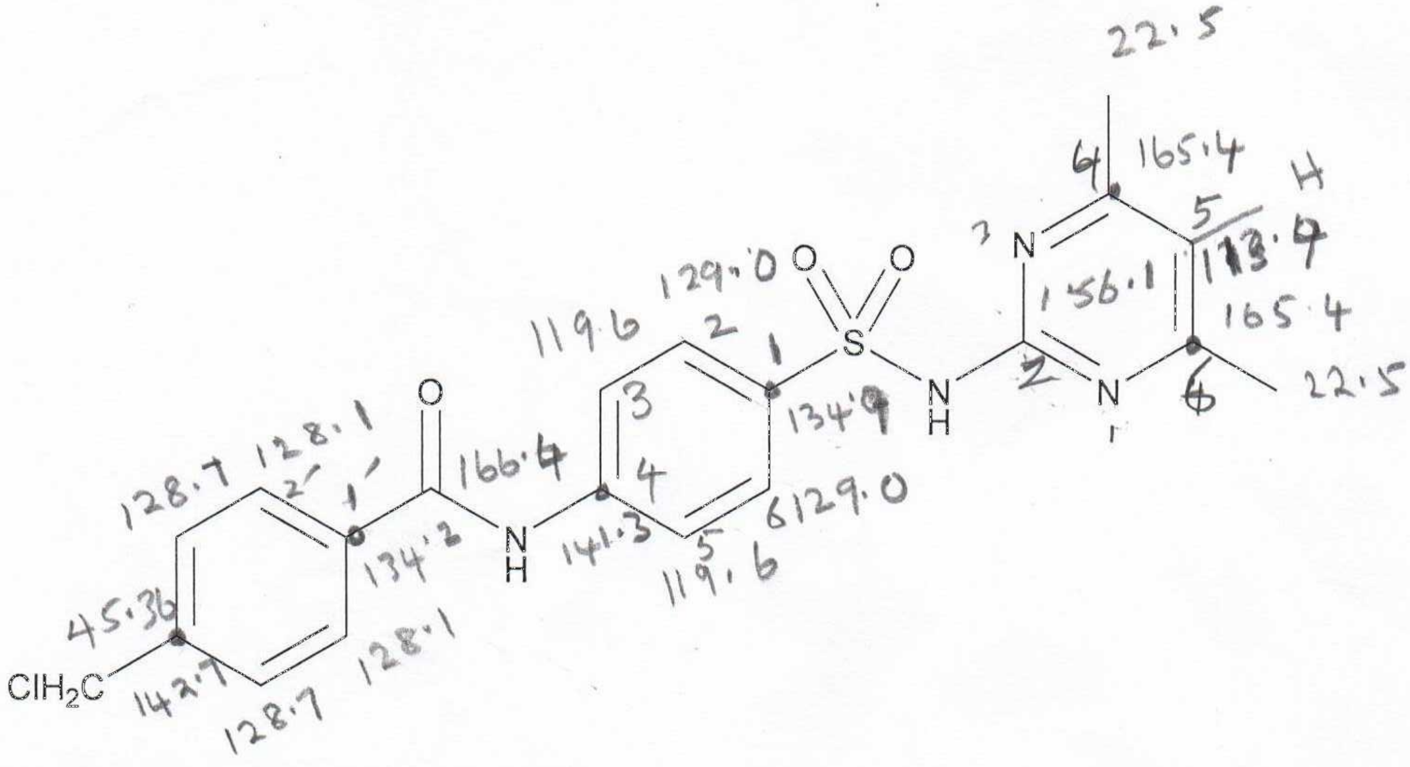

MHH-1-28

Discuss

JEOL HX 110 MASS SPECTROMETER (FAB-HR)

STUDENT NAME	Dr. M. H. Haroon	SAMPLE CODE	DATE	25/5/17
SUPERVISOR NAME	Dr. Hina	MHH-I-28	FAB (+VE / -VE)	FAB +ve

Mass	Theoretical Mass	Delta [ppm]	Delta [mmu]	RDB	Composition
431.0962	431.0967	-1.1	-0.5	21.5	$C_{26} H_{15} O_1 N_4 S_1$
	431.0951	2.5	1.1	21.5	$C_{28} H_{16} O_1 N_2 Cl_1$
	431.0946	3.6	1.6	26.0	$C_{31} H_{13} O_2 N_1$
	431.0945	4.0	1.7	12.5	→ $C_{20} H_{20} O_3 N_4 Cl_1 S_1$
	431.0980	-4.2	-1.8	21.0	$C_{28} H_{17} O_2 N_1 S_1$
	431.0985	-5.3	-2.3	16.5	$C_{25} H_{20} O_1 N_2 Cl_1 S_1$
	431.0933	6.8	2.9	26.5	$C_{29} H_{11} O_1 N_4$
	431.0911	11.8	5.1	17.5	$C_{23} H_{16} O_3 N_4 Cl_1$
	431.0894	15.7	6.8	25.5	$C_{32} H_{15} S_1$
	431.1037	-17.3	-7.5	17.0	$C_{24} H_{18} O_3 N_3 Cl_1$

[illegible]

2/13/2017 12:33:58 PM

File: MHH-I-28 **Date Run: 02-13-2017 (Time Run: 11:44:48)**

Sample: DR.M.H.HAROON /DR. HINA

Instrument: JEOL MS 600H-1

Ionization mode: EI+

Scan: 26 **R.T.: 2.22**

Base: m/z 119; 4.1%FS TIC: 468676

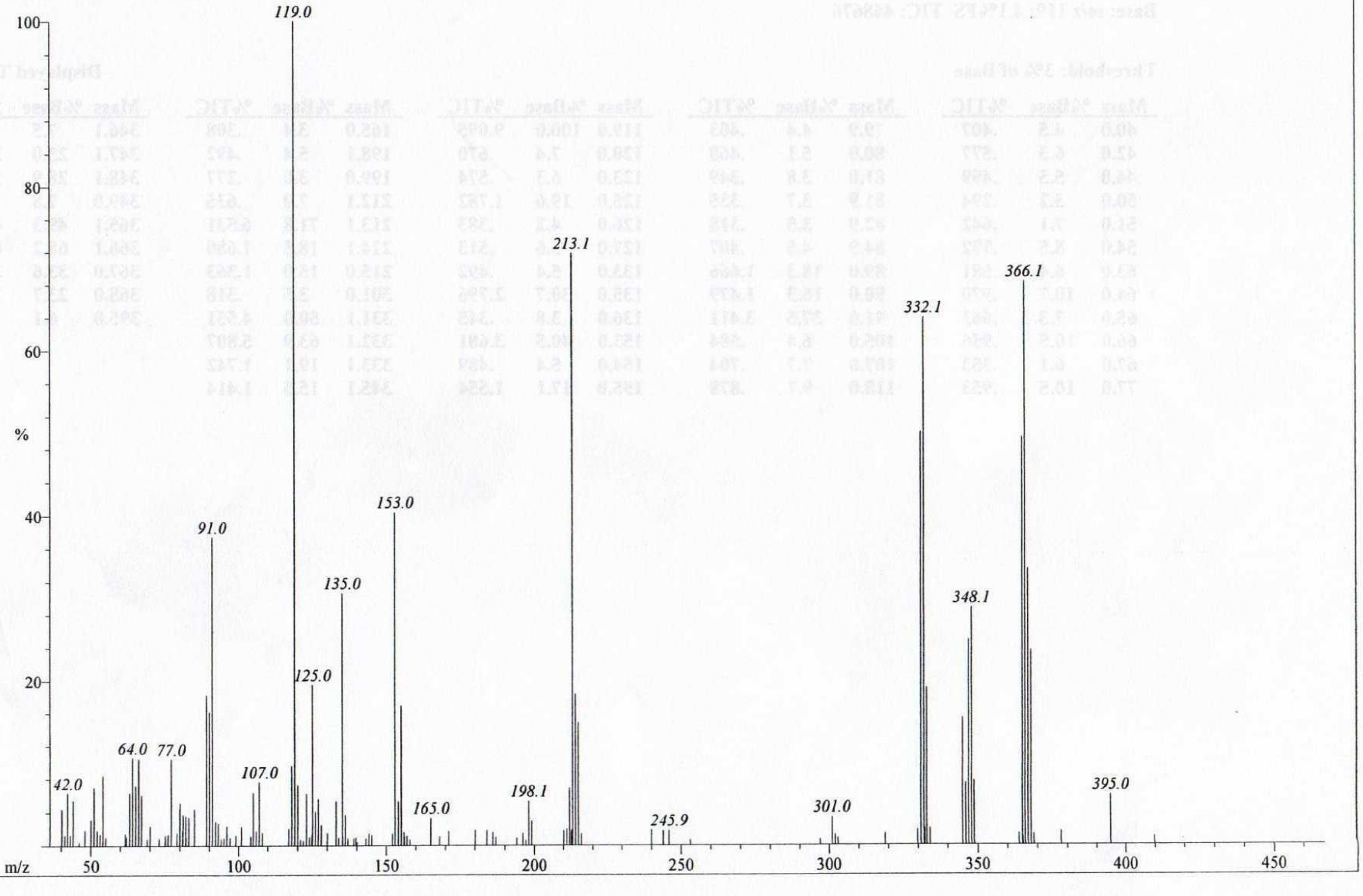

I. C.C.B.S., University of Karachi Analytical Laboratory - Pakistan

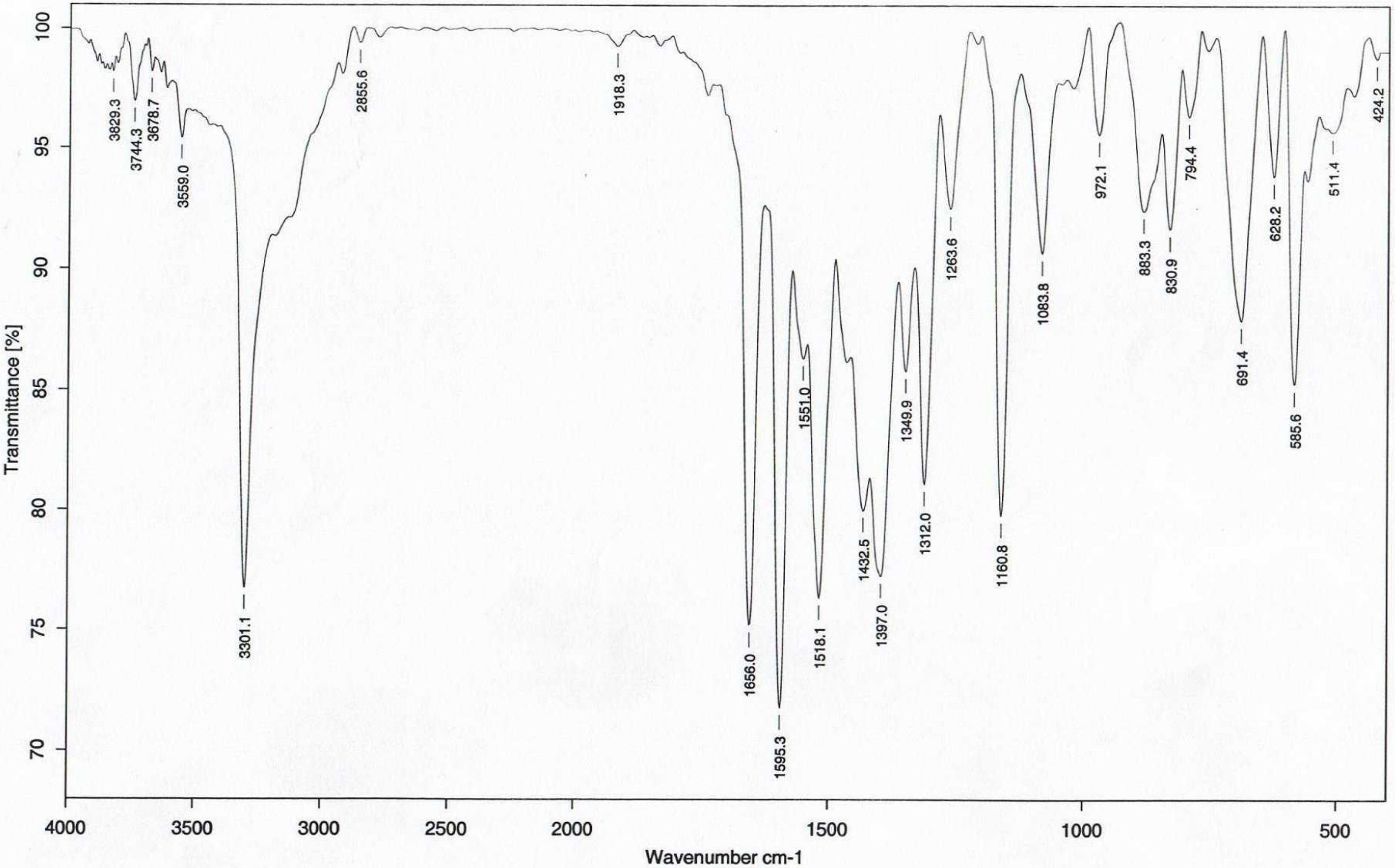

Sample : MHH-1-28/Dr.Haroon	Spectrum : MHH-1-28.1 (in D:\IRSTUDENT)
Measured : 30/01/2017 on VECTOR22	Technic : Solid
Resolution : 4 cm-1 (10 scans)	**Analyst : Zubair Ahmad/ Jamshed**

THERMO ELECTRON ~ VISIONpro SOFTWARE V4.10

Operator Name	ARSHAD ALAM.	Date of Report	1/30/2017
Department	Analytical Laboratory TWC # 004	Time of Report	10:27:44AM
Organization	ICCBS Karachi of Universty.		
Information	Dr Haron/Dr Hina		

Scan Graph

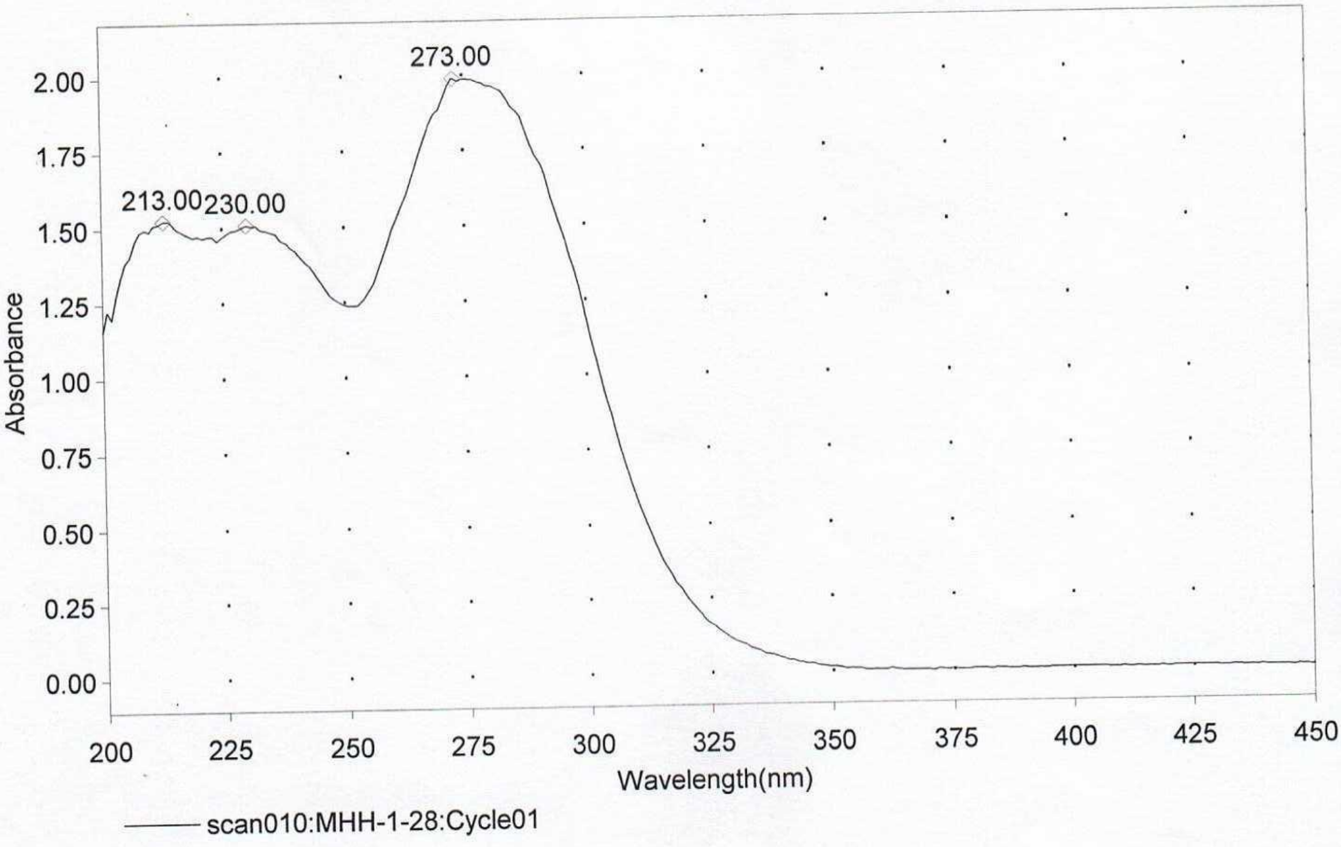

Results Table - MHH-1-28.sre,MHH-1-28,Cycle01

nm	A	**Peak Pick Method**
213.00	1.524	Find 8 Peaks Above -3.0000 A
230.00	1.508	Start Wavelength 200.00 nm
273.00	1.988	Stop Wavelength 450.00 nm
		Sort By Wavelength

Sensitivity Auto
