# Supplementary material for: Sulphamethazine derivatives as immunomodulating agents: New therapeutic strategies for inflammatory diseases
Source: PLoS One. 2018 Dec 19;13(12):e0208933. doi: 10.1371/journal.pone.0208933 (PMC6300282; doi:10.1371/journal.pone.0208933)
Supplement: S4 Fig — (PDF) [file pone.0208933.s004.pdf]

AVANCE AV-400 MHz
Lab # 115

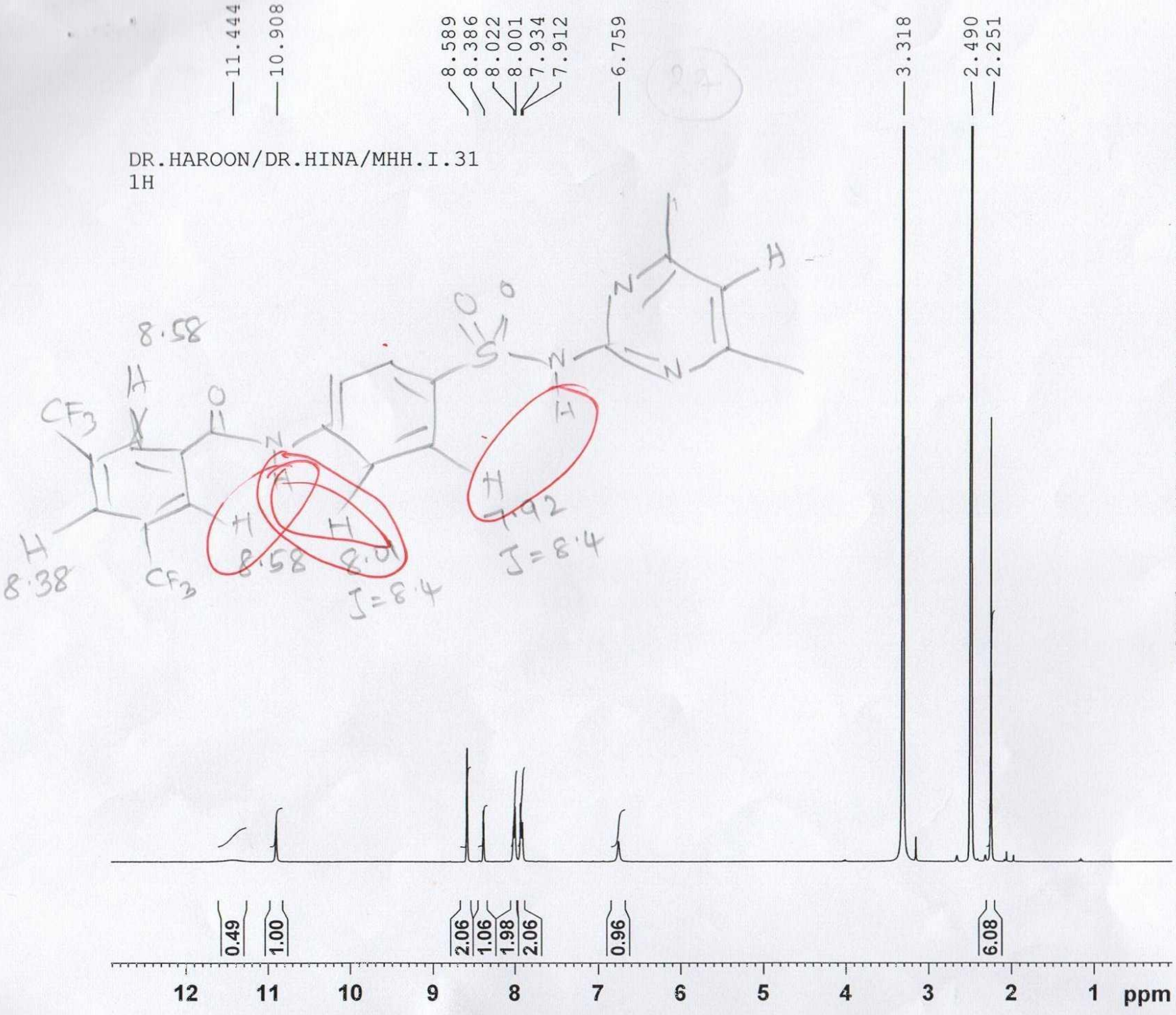

6

NAME	jan06-17
EXPNO	1
PROCNO	1
Date_	20170106
Time	10.33
INSTRUM	spect
PROBHD	5 mm SEI 1H-13
PULPROG	zg30
TD	65536
SOLVENT	DMSO
NS	64
DS	0
SWH	8012.820 Hz
FIDRES	0.122266 Hz
AQ	4.0894966 sec
RG	512
DW	62.400 usec
DE	6.50 usec
TE	300.0 K
D1	2.00000000 sec
TD0	1

======== CHANNEL f1 ========

NUC1	1H
P1	10.80 usec
PL1	3.00 dB
SFO1	400.0332002 MHz
SI	32768
SF	400.0300041 MHz
WDW	EM
SSB	0
LB	0.30 Hz
GB	0
PC	1.00

DR.HAROON/DR.HINA/MHH.I.31
1H

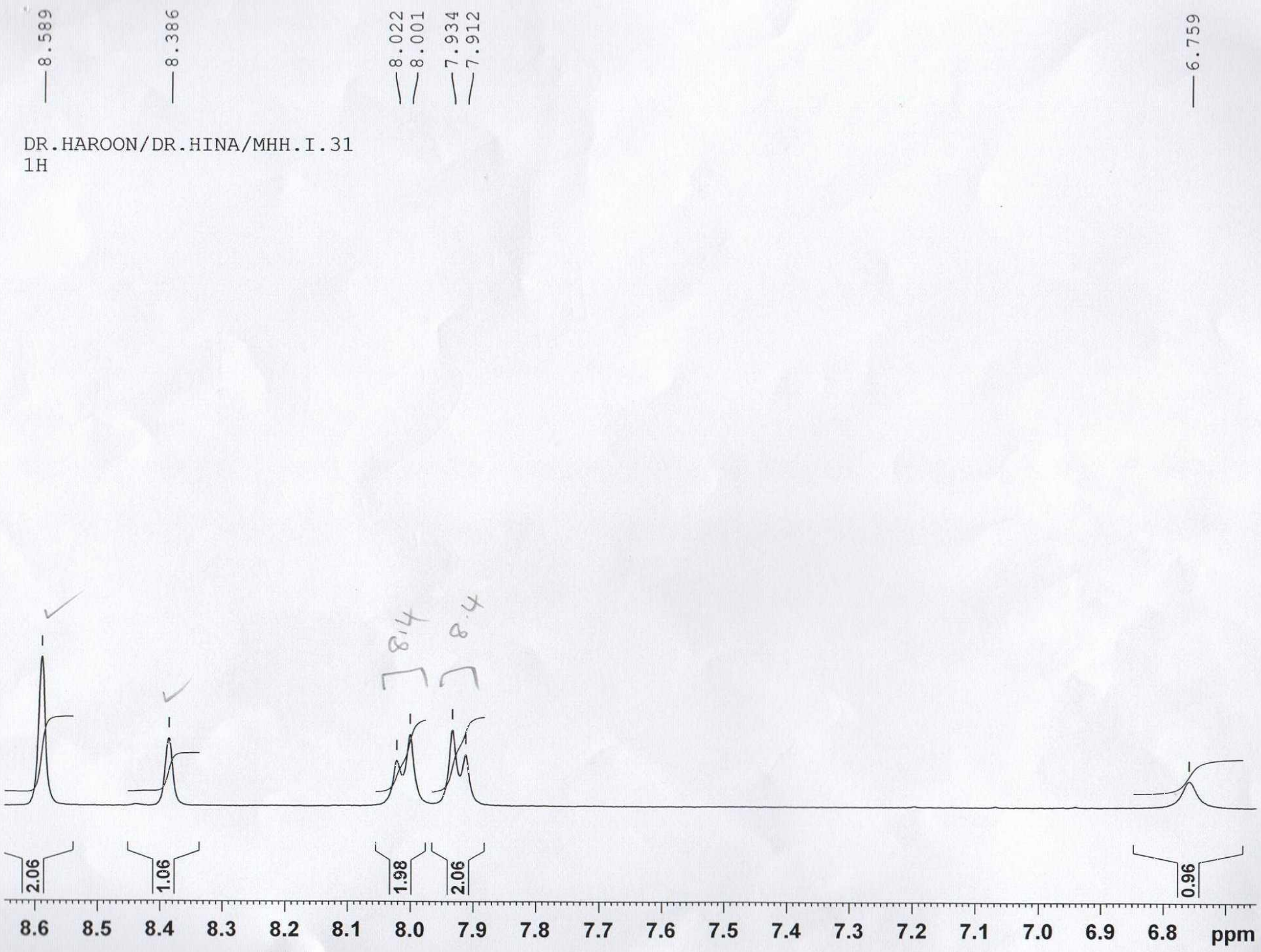

DR.M.H.HAROON/DR.HINA/MHH-1-31/DMSO
ICCBS,U.O.K/BB

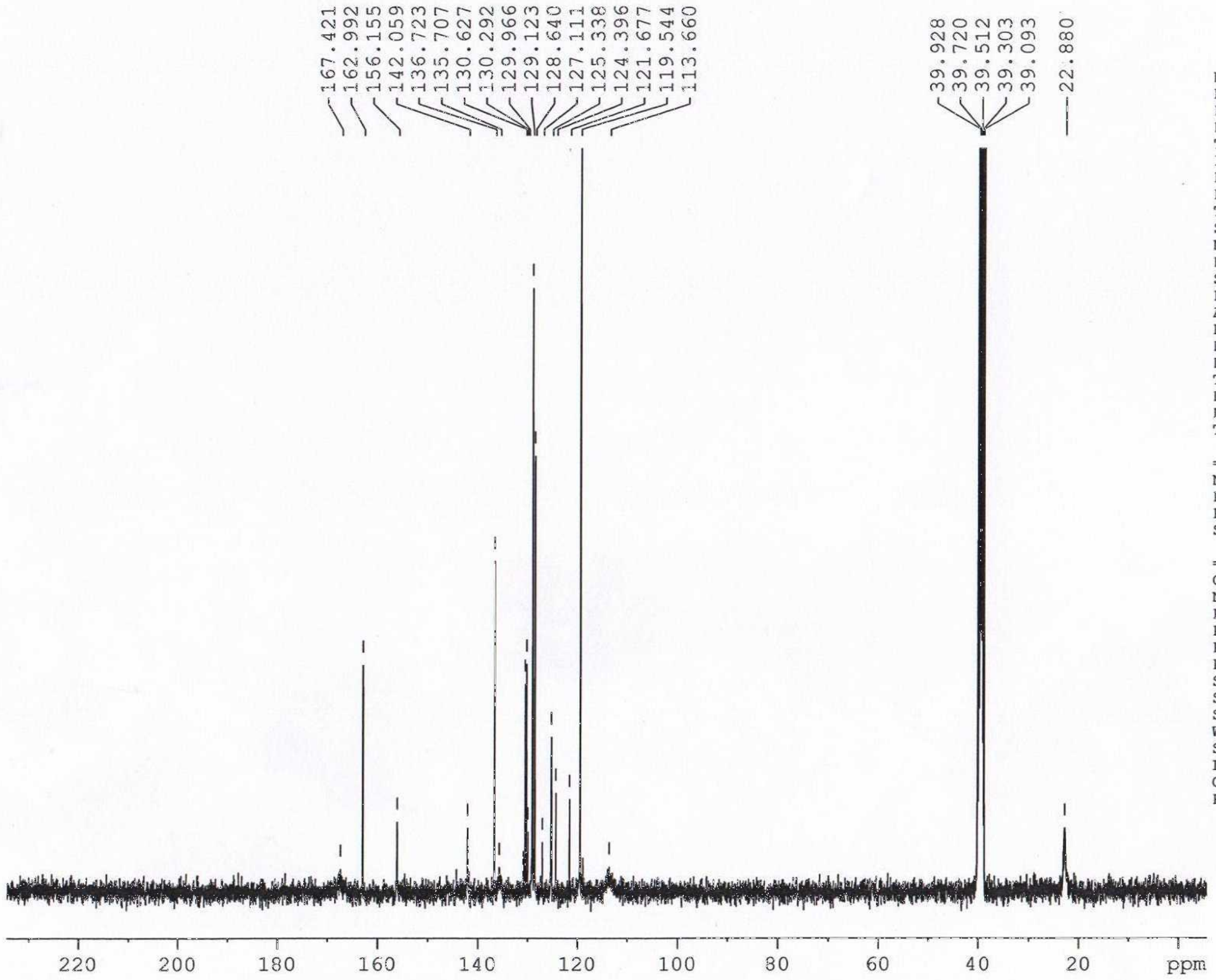

AVANCE 400
LAB NO 117

NAME	apr27-17
EXPNO	15
PROCNO	1
Date_	20170427
Time	15.27
INSTRUM	spect
PROBHD	5 mm DUL 13C-1
PULPROG	zgpg
TD	32768
SOLVENT	DMSO
NS	18432
DS	0
SWH	24154.590 Hz
FIDRES	0.737140 Hz
AQ	0.6783476 sec
RG	32768
DW	20.700 usec
DE	6.50 usec
TE	300.0 K
D1	2.00000000 sec
D11	0.03000000 sec
TD0	18

======== CHANNEL f1 ========

NUC1	13C
P1	8.55 usec
PL1	7.00 dB
SFO1	100.6243395 MHz

======== CHANNEL f2 ========

CPDPRG2	waltz16
NUC2	1H
PCPD2	80.00 usec
PL2	0.00 dB
PL12	19.00 dB
PL13	20.00 dB
SFO2	400.1324008 MHz
SI	16384
SF	100.6128205 MHz
WDW	EM
SSB	0
LB	1.00 Hz
GB	0
PC	1.00

DR.M.H.HAROON/DR.HINA/MHH-1-31/DMSO
ICCBS,U.O.K/BB

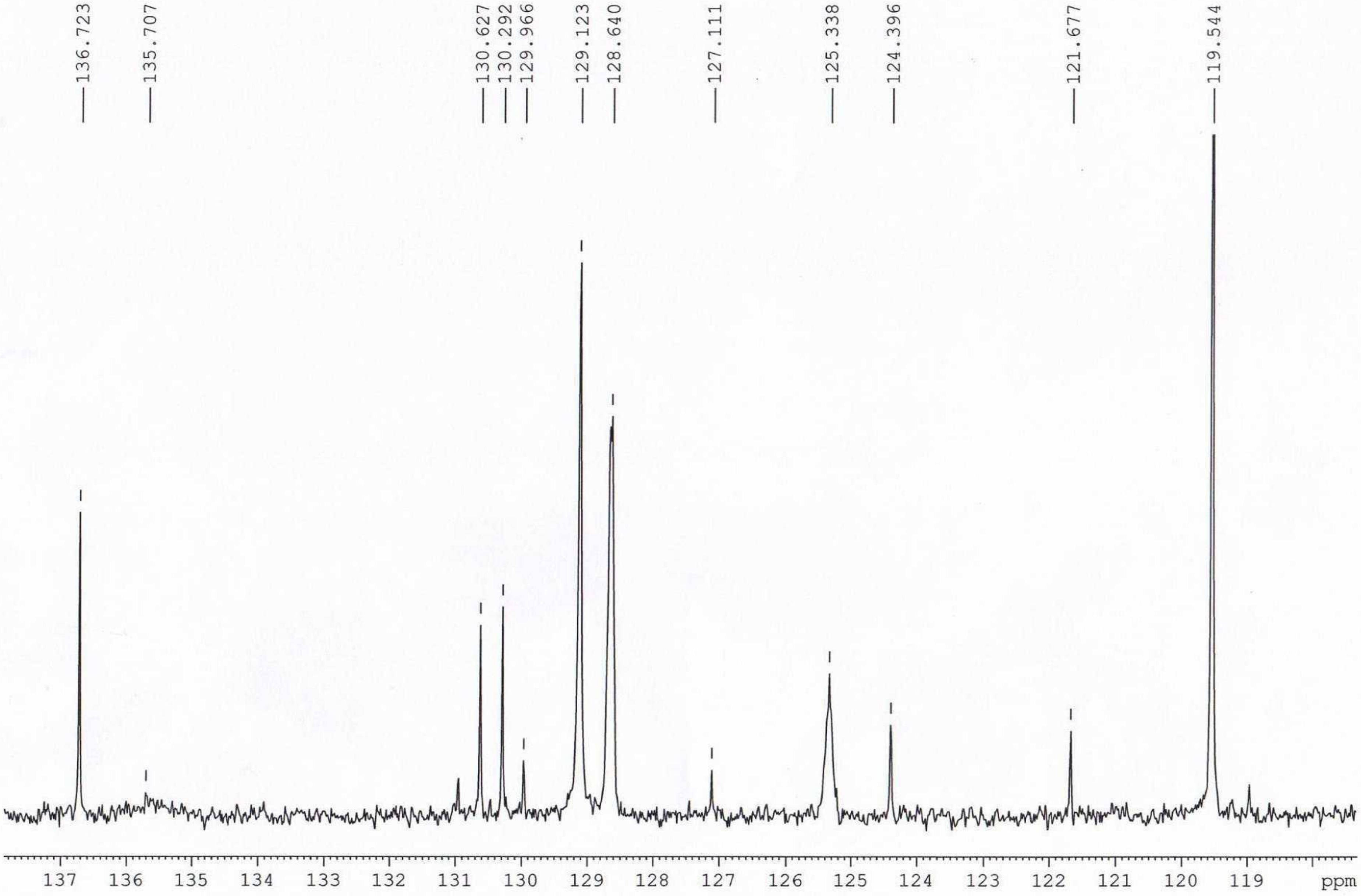

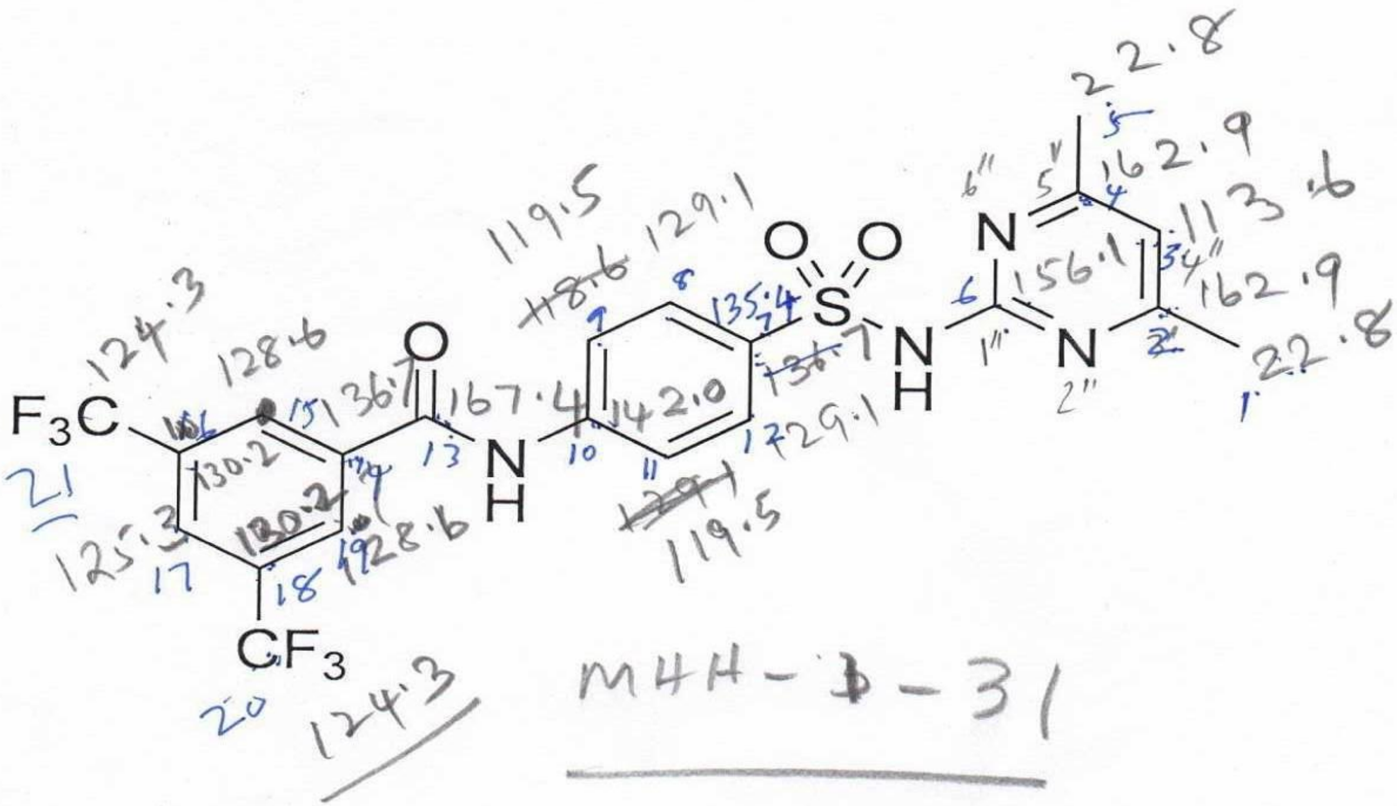

124.3

MHH - D - 31

Qu =

14 +

135.47

C - 11

CH 8

JEOL HX 110 MASS SPECTROMETER (FAB-HR)

STUDENT NAME	Haroon	SAMPLE CODE	DATE	23-05-17
SUPERVISOR NAME	Dr. Hina	MHH-I-31	FAB (+VE / -VE)	FAB+Ve

Mass	Theoretical Mass	Delta [ppm]	Delta [mmu]	RDB	Composition
519.0939	519.0934	1.0	0.5	32.5	$C_{37} H_{12} O_1 N_2 F_1$
	519.0945	-1.2	-0.6	28.5	$C_{34} H_{13} O_2 N_2 F_2$
	519.0932	1.3	0.7	21.5	$C_{29} H_{13} O_1 N_2 F_6$
	519.0929	1.9	1.0	27.0	$C_{34} H_{17} O_3 N_1 S_1$
	519.0928	2.2	1.1	16.0	$C_{26} H_{18} O_3 N_1 F_5 S_1$
	519.0927	2.3	1.2	23.5	$C_{29} H_{16} O_3 N_4 F_1 S_1$
	519.0926	2.6	1.3	12.5	$C_{21} H_{17} O_3 N_4 F_6 S_1$
	519.0954	-3.0	-1.5	20.5	$C_{29} H_{16} N_2 F_5 S_1$
	519.0922	3.2	1.7	36.5	$C_{40} H_{11} N_2$
	519.0956	-3.3	-1.7	31.5	$C_{37} H_{15} N_2 S_1$

File: MHH-I-31
Sample: DR.M.H.HAROON /DR. HINA
Instrument: JEOL MS 600H-1

Date Run: 02-10-2017 (Time Run: 09:47:08)

Ionization mode: EI+

Scan: 16
Base: m/z 453; 34.7%FS TIC: 1615602

R.T.: 1.33

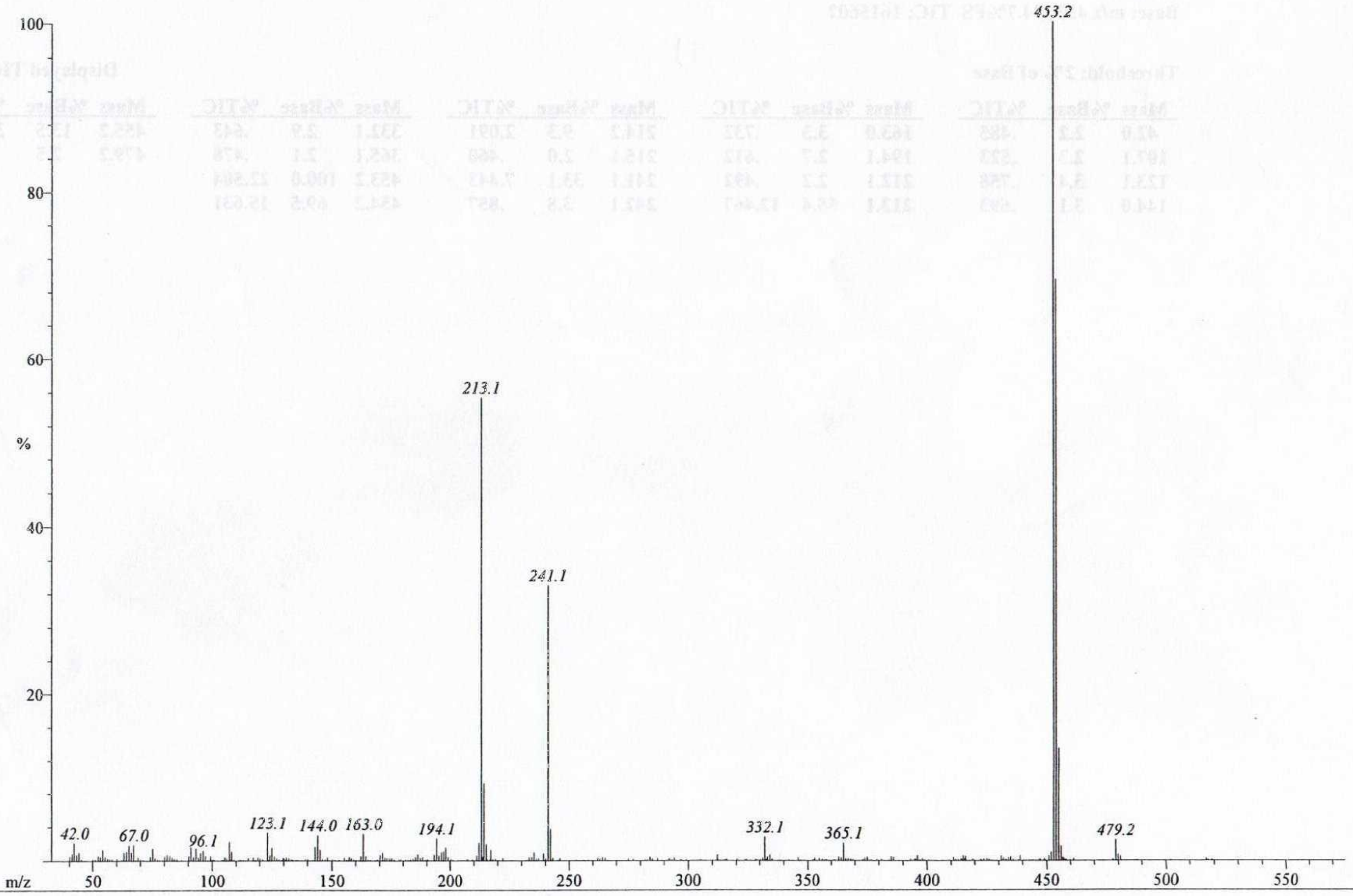

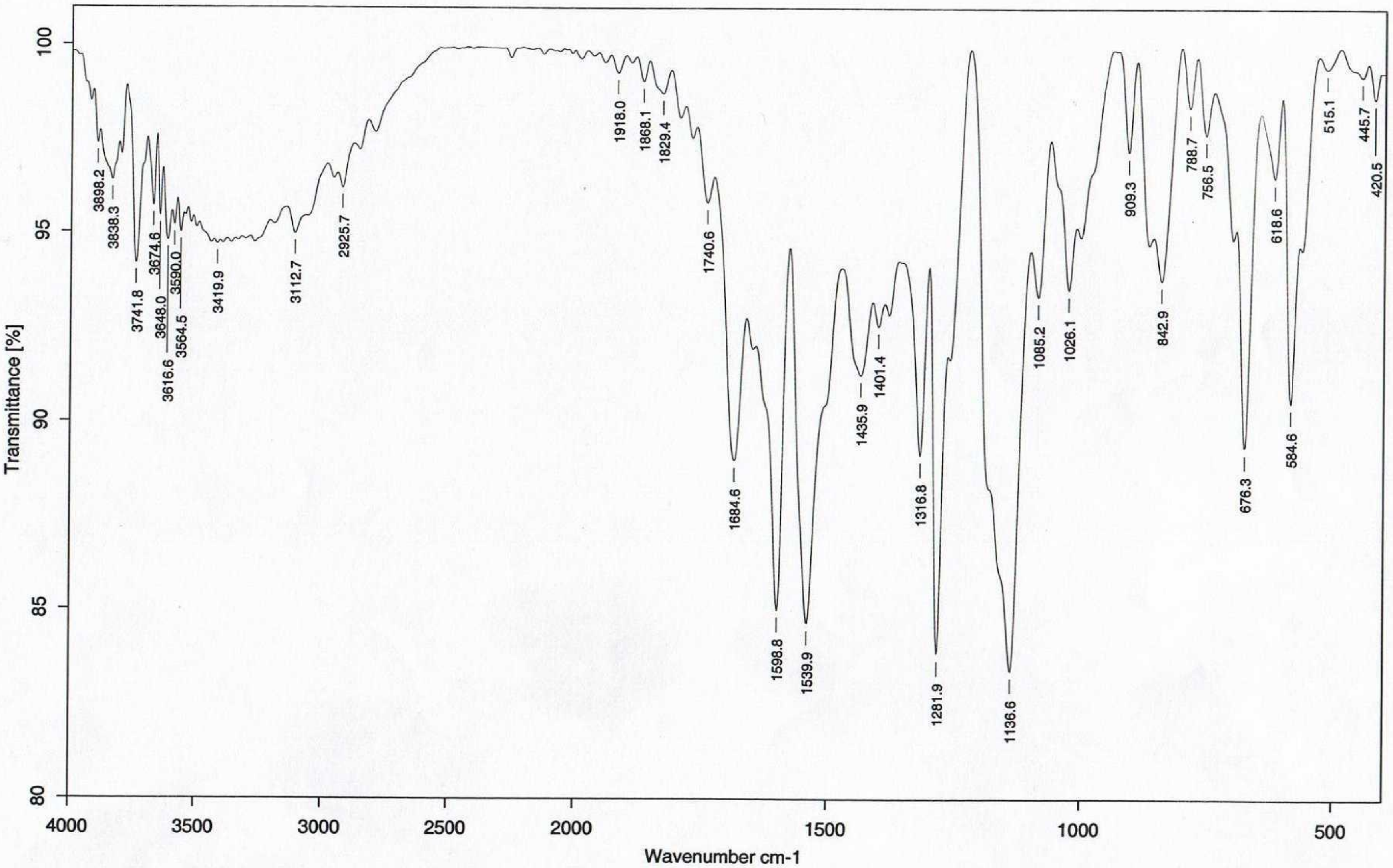

Sample : MHH-1-31/Dr.Haroon	Spectrum : MHH-1-31.0 (in D:\IRSTUDENT)
Measured : 27/01/2017 on VECTOR22	Technic : Liquid
Resolution : 4 cm-1 (10 scans)	Analyst : M. Asif

THERMO ELECTRON ~ VISIONpro SOFTWARE V4.10

Operator Name	ARSHAD ALAM.	Date of Report	1/30/2017
Department	Analytical Laboratory TWC # 004	Time of Report	10:37:51AM
Organization	ICCBS Karachi of Universty.		
Information	Dr Haron/Dr Hina		

Scan Graph

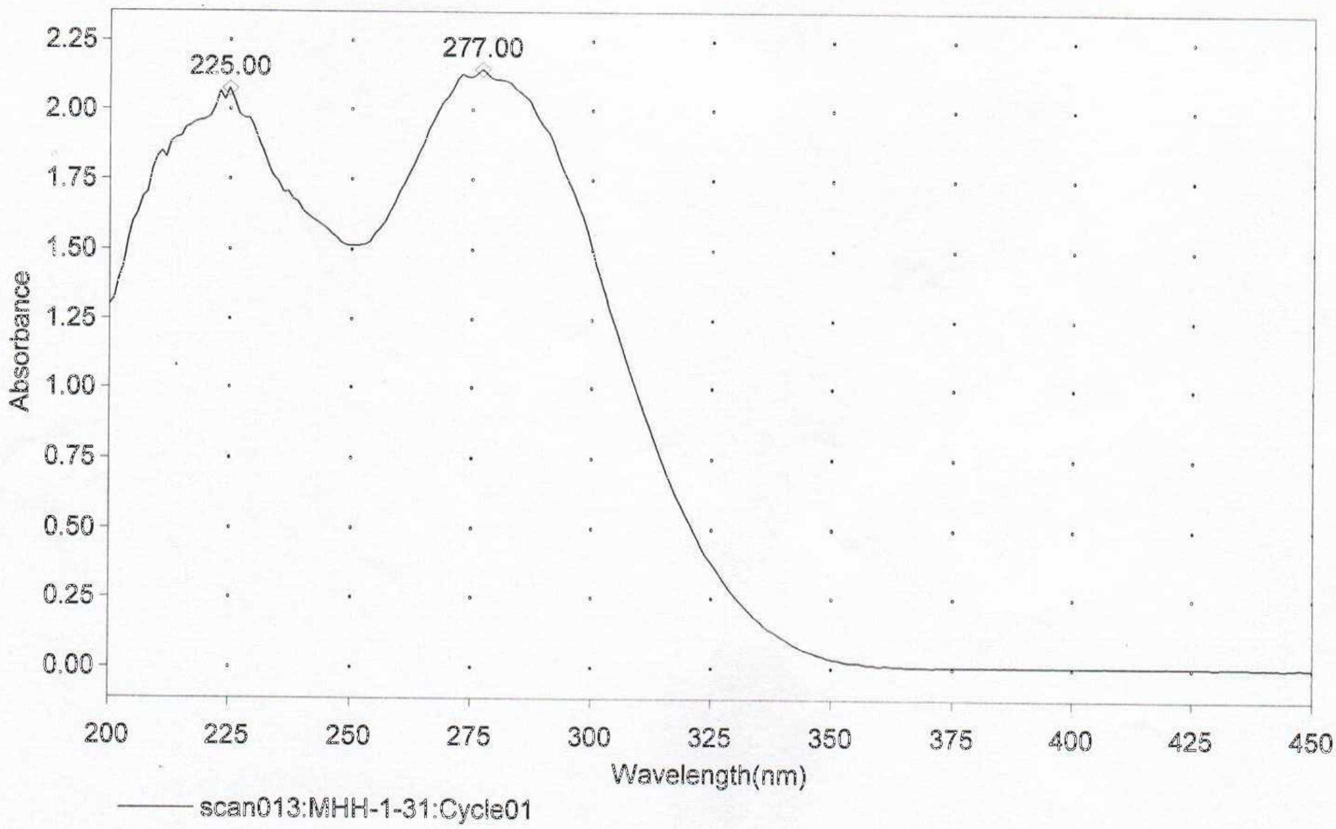

Results Table - MHH-1-31.sre,MHH-1-31,Cycle01

nm	A	**Peak Pick Method**
225.00	2.075	Find 8 Peaks Above -3.0000 A
277.00	2.145	Start Wavelength 200.00 nm
		Stop Wavelength 450.00 nm
		Sort By Wavelength

Sensitivity Auto
