# Supplementary material for: Sulphamethazine derivatives as immunomodulating agents: New therapeutic strategies for inflammatory diseases
Source: PLoS One. 2018 Dec 19;13(12):e0208933. doi: 10.1371/journal.pone.0208933 (PMC6300282; doi:10.1371/journal.pone.0208933)
Supplement: S5 Fig — (PDF) [file pone.0208933.s005.pdf]

AVANCE AV-400 MHz
Lab # 115

DR.HAROON/DR.HINA/MHH.I.17
1H

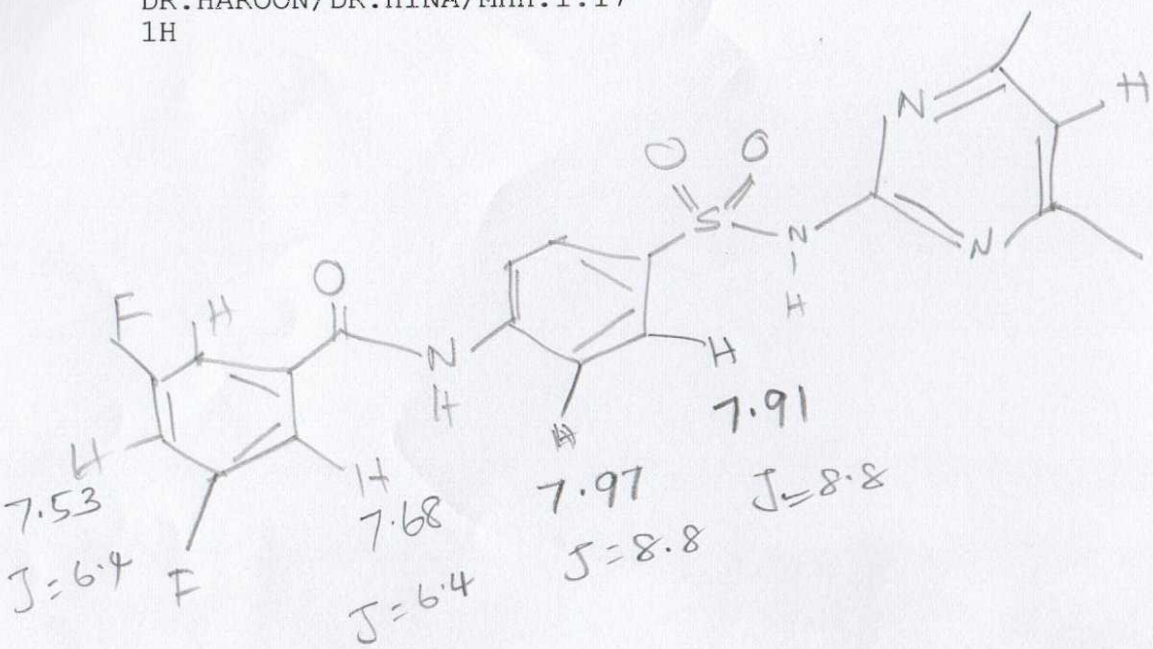

NAME	jan02-17
EXPNO	1
PROCNO	1
Date_	20170102
Time	10.27
INSTRUM	spect
PROBHD	5 mm SEI 1H-13
PULPROG	zg30
TD	65536
SOLVENT	DMSO
NS	64
DS	0
SWH	8012.820 Hz
FIDRES	0.122266 Hz
AQ	4.0894966 sec
RG	512
DW	62.400 usec
DE	6.50 usec
TE	300.0 K
D1	2.00000000 sec
TD0	1

======== CHANNEL f1 ========

NUC1	1H
P1	10.80 usec
PL1	3.00 dB
SFO1	400.0332002 MHz
SI	32768
SF	400.0300041 MHz
WDW	EM
SSB	0
LB	0.30 Hz
GB	0
PC	1.00

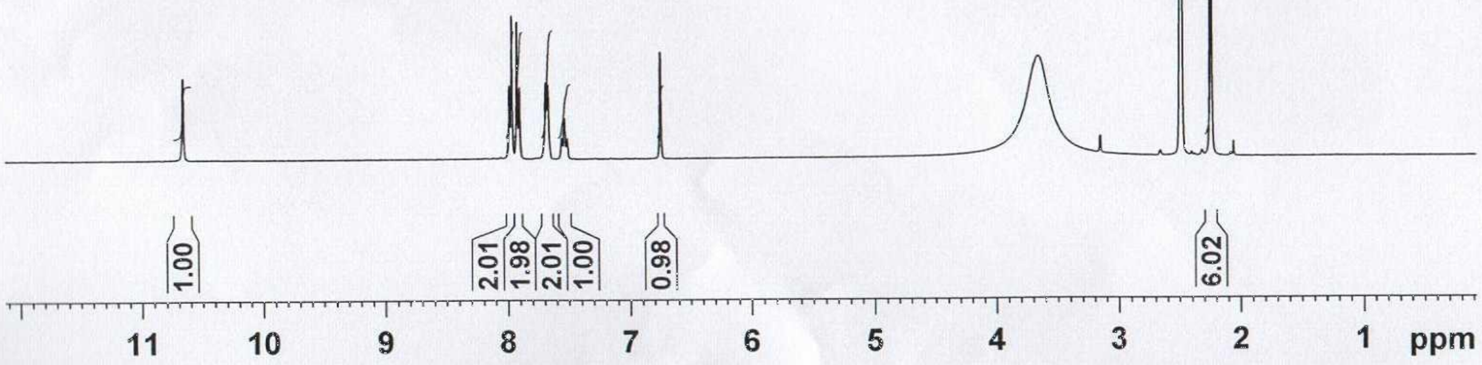

DR.HAROON/DR.HINA/MHH.I.17
1H

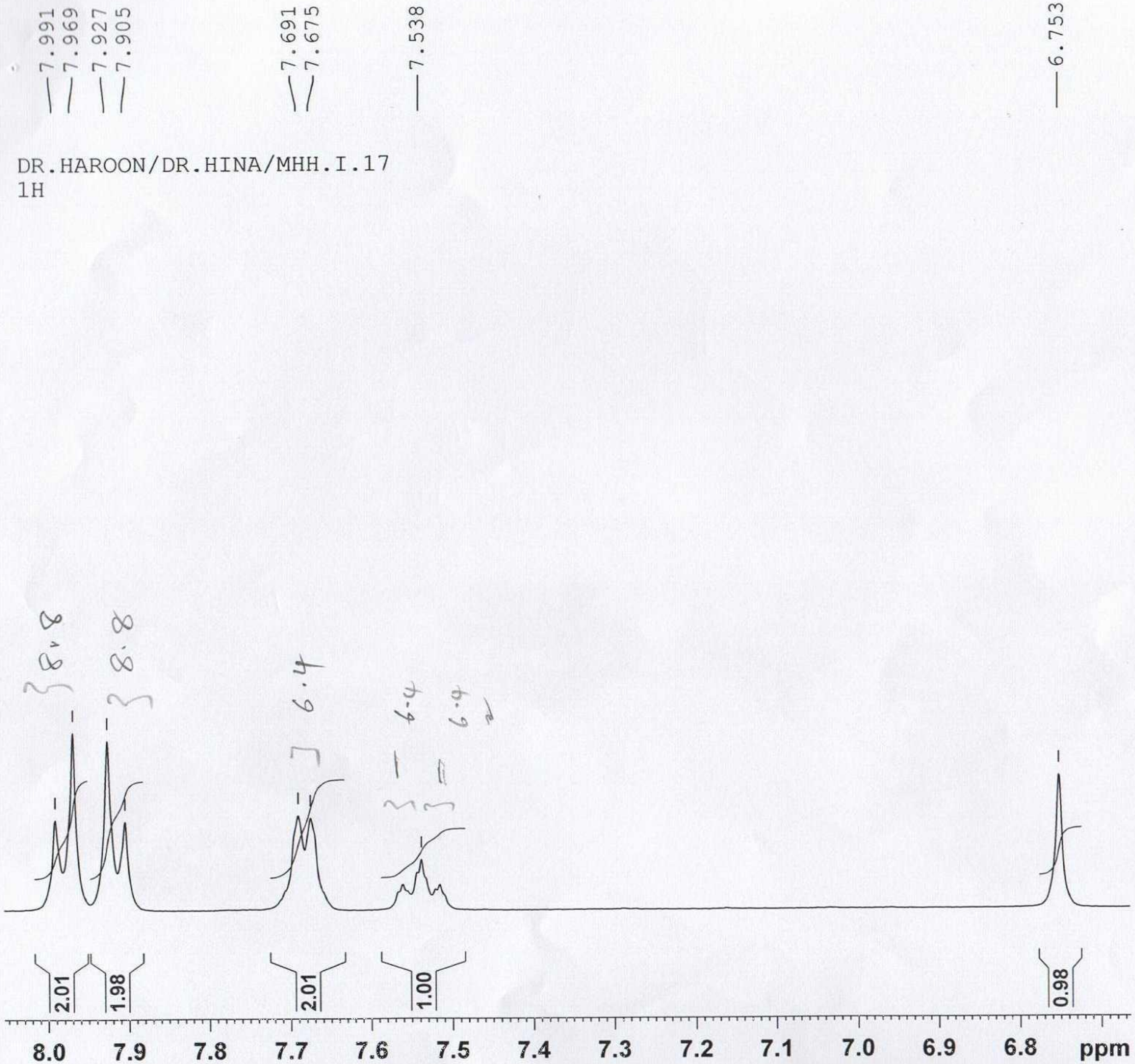

DR.M.H.HAROON/DR.HINA/MHH-1-17/DMSO
ICCBS,U.O.K/BB

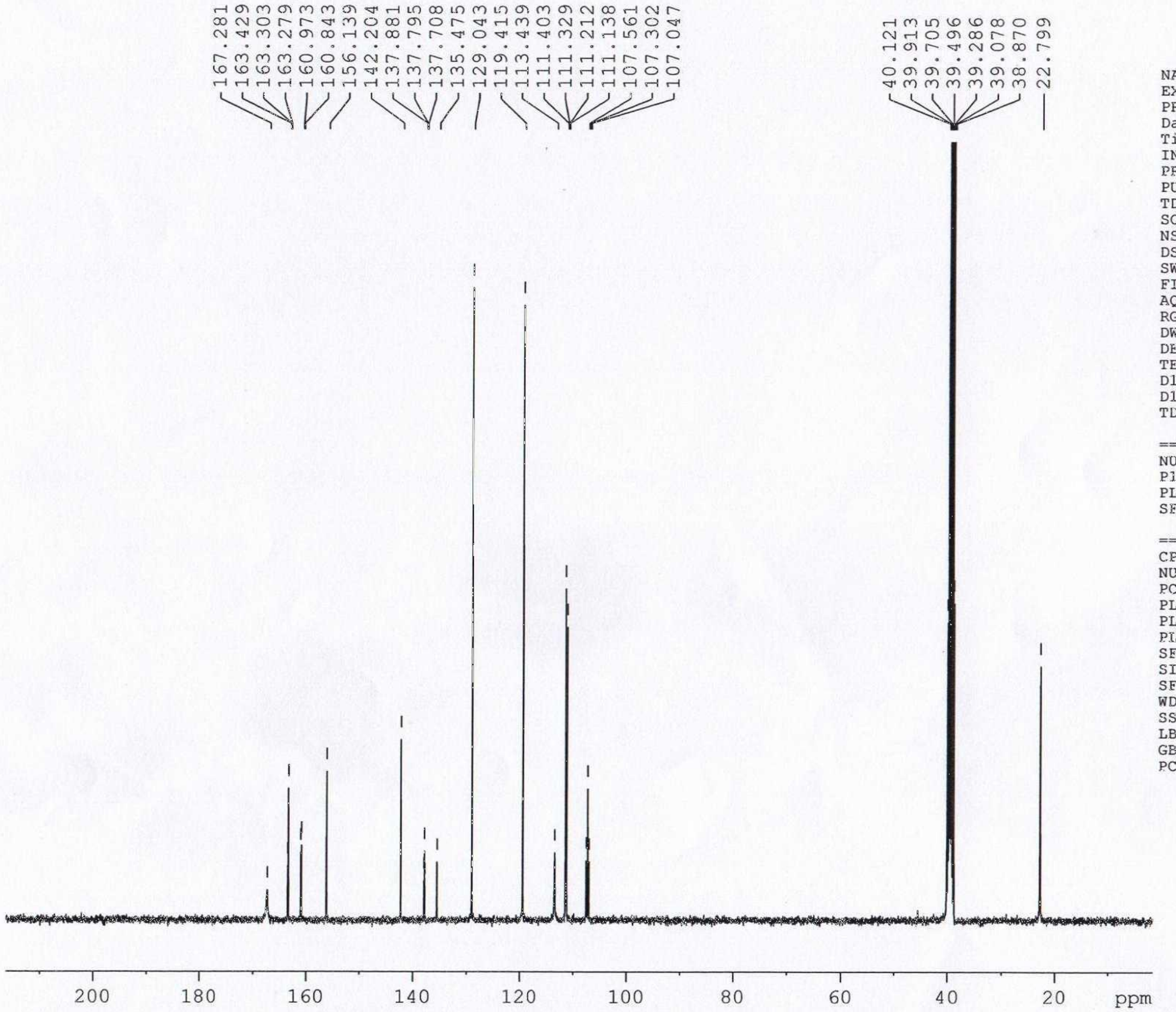

AVANCE 400
LAB NO 117

NAME	apr30-17
EXPNO	1
PROCNO	1
Date_	20170430
Time	13.28
INSTRUM	spect
PROBHD	5 mm DUL 13C-1
PULPROG	zgpg
TD	32768
SOLVENT	DMSO
NS	18432
DS	0
SWH	24154.590 Hz
FIDRES	0.737140 Hz
AQ	0.6783476 sec
RG	32768
DW	20.700 usec
DE	6.50 usec
TE	300.0 K
D1	2.00000000 sec
D11	0.03000000 sec
TD0	18

======== CHANNEL f1 ========

NUC1	13C
P1	8.55 usec
PL1	7.00 dB
SFO1	100.6243395 MHz

======== CHANNEL f2 ========

CPDPRG2	waltz16
NUC2	1H
PCPD2	80.00 usec
PL2	0.00 dB
PL12	19.00 dB
PL13	20.00 dB
SFO2	400.1324008 MHz
SI	16384
SF	100.6128205 MHz
WDW	EM
SSB	0
LB	1.00 Hz
GB	0
PC	1.00

DR.M.H.HAROON/DR.HINA/MHH-1-17/DMSO
ICCBS,U.O.K/BB

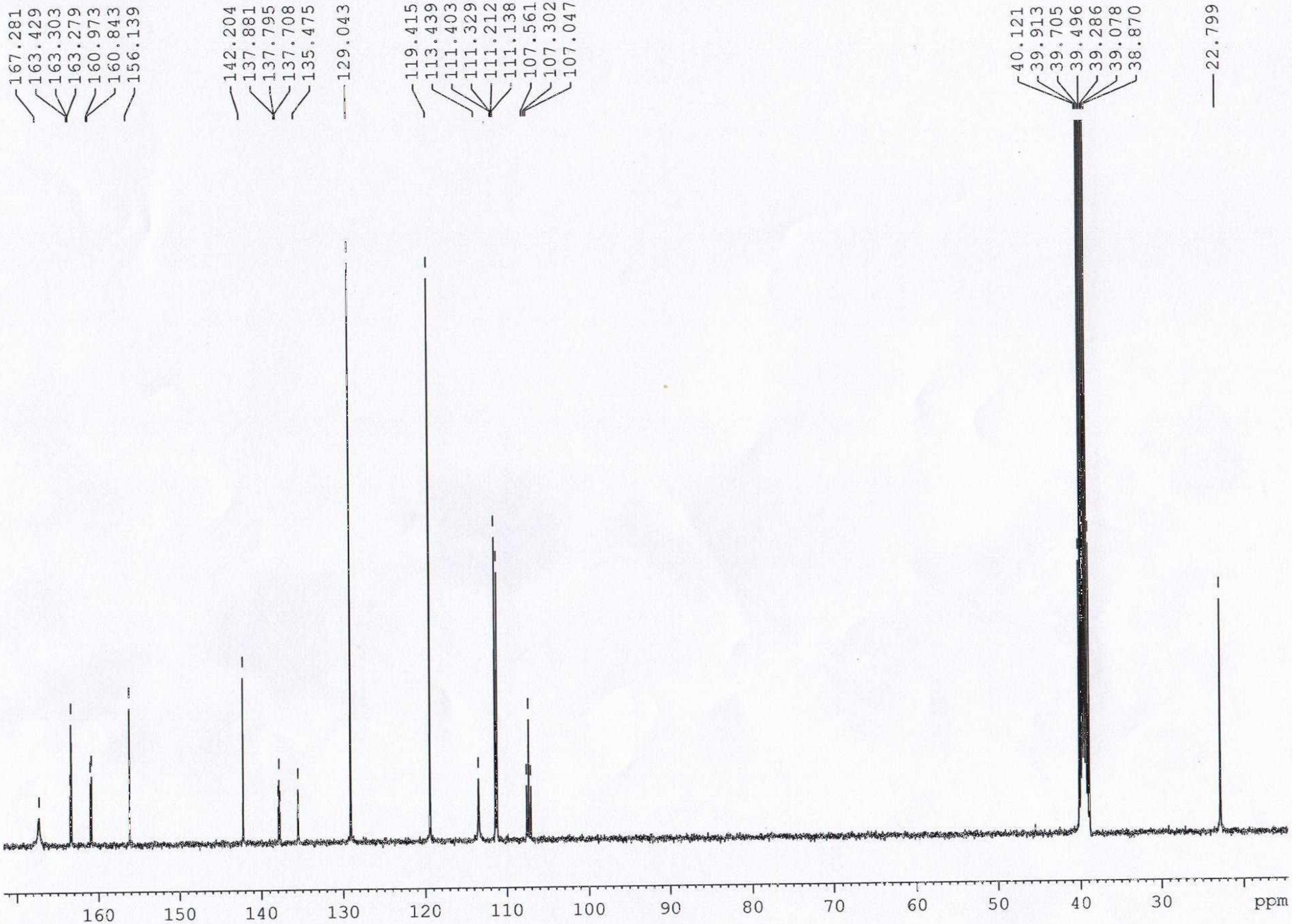

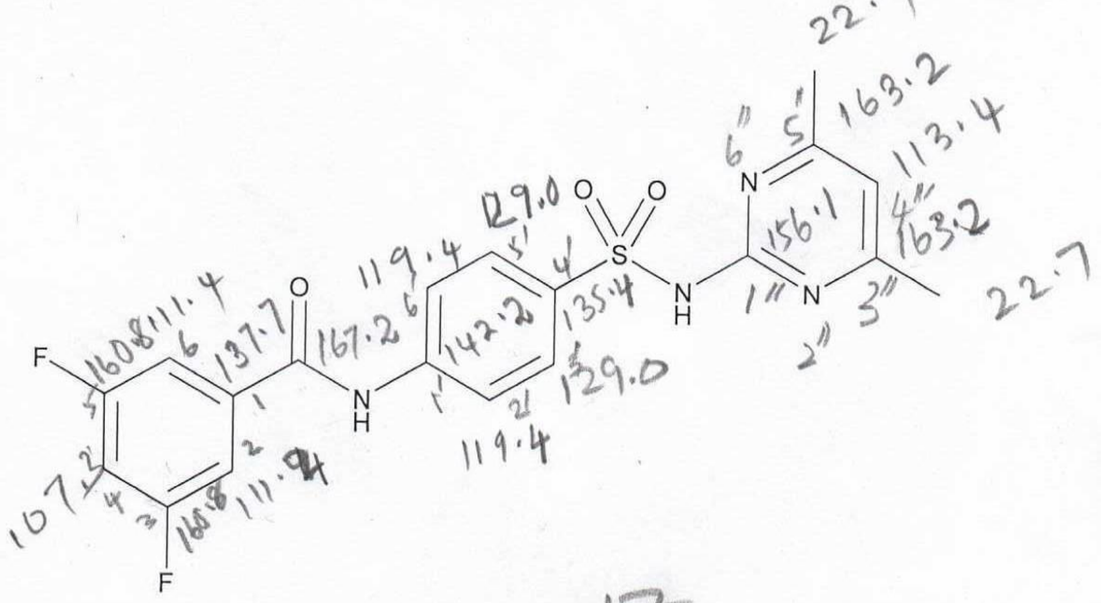

MHH-1-17

JEOL HX 110 MASS SPECTROMETER (FAB-HR)

STUDENT NAME	Dr. M.H. Haroon	SAMPLE CODE	DATE	25/5/17
SUPERVISOR NAME	Dr. Hina	MHH-I-17	FAB (+VE / -VE)	FAB+Ve

Mass	Theoretical Mass	Delta [ppm]	Delta [mmu]	RDB	Composition
419.0979	419.0980	-0.2	-0.1	20.0	$C_{27} H_{17} O_2 N_1 S_1$
	419.0978	0.2	0.1	16.5	$C_{22} H_{16} O_2 N_4 F_1 S_1$
	419.0985	-1.3	-0.6	25.5	$C_{30} H_{12} N_2 F_1$
	419.0989	-2.5	-1.0	12.5	→ $C_{19} H_{17} O_3 N_4 F_2 S_1$
	419.0967	2.9	1.2	13.0	$C_{22} H_{17} O_1 N_1 F_4 S_1$
	419.0991	-3.0	-1.2	16.0	$C_{24} H_{18} O_3 N_1 F_1 S_1$
	419.0967	3.0	1.2	20.5	$C_{25} H_{15} O_1 N_4 S_1$
	419.0996	-4.0	-1.7	21.5	$C_{27} H_{13} O_1 N_2 F_2$
	419.0958	5.1	2.1	21.0	$C_{27} H_{14} O_3 N_1 F_1$
	419.0956	5.6	2.3	17.5	$C_{22} H_{13} O_3 N_4 F_2$

HEJ-ICCBS
2/9/2017 12:19:28 PM

File: MHH-I-17 **Date Run: 02-09-2017 (Time Run: 12:04:13)**
Sample: DR.M.H.HAROON /DR. HINA
Instrument: JEOL MS 600H-1
Ionization mode: EI+

Scan: 20 **R.T.: 1.68**
Base: m/z 353; 99.5%FS TIC: 4651830

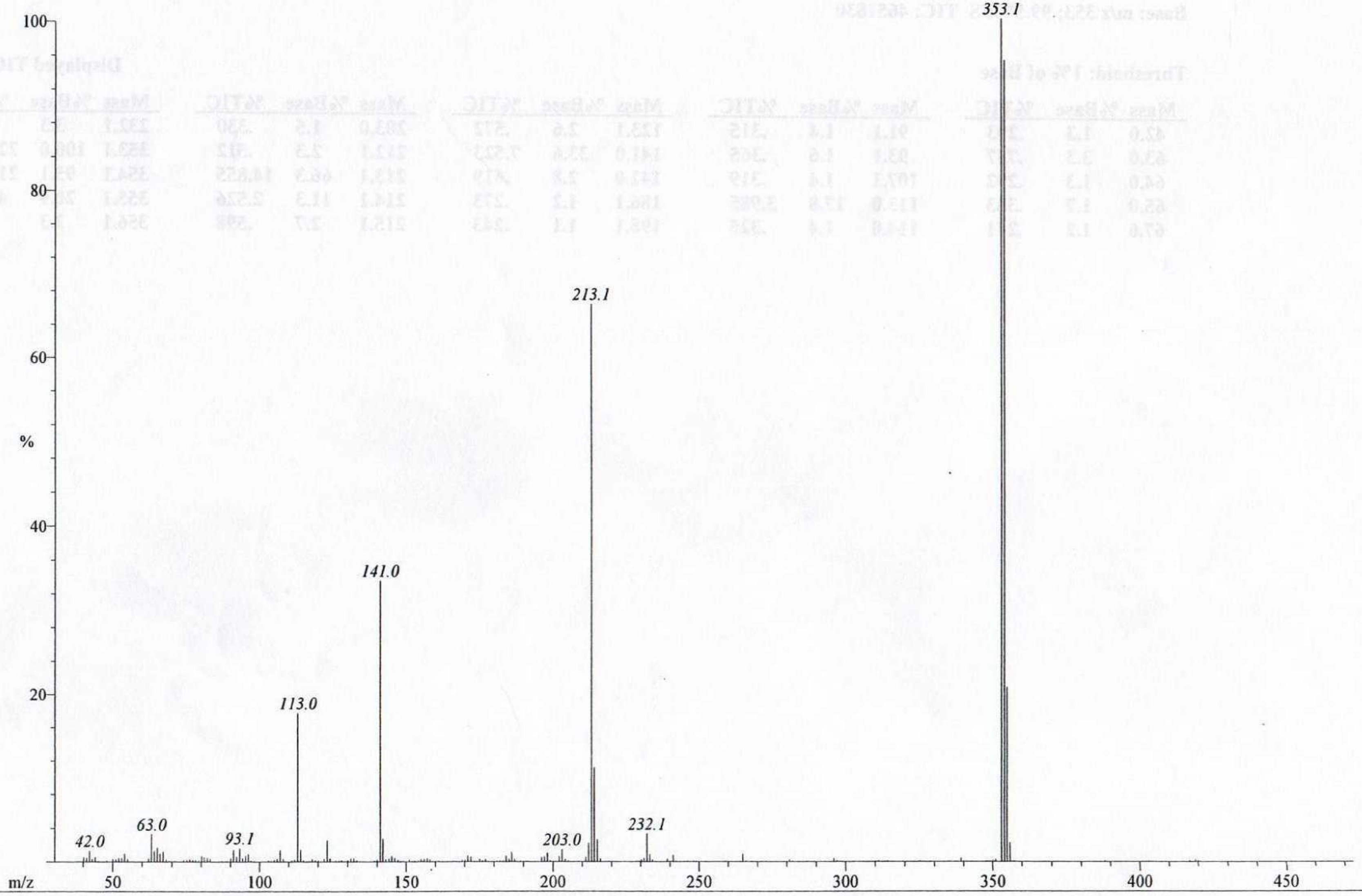

I. C.C.B.S., University of Karachi Analytical Laboratory - Pakistan

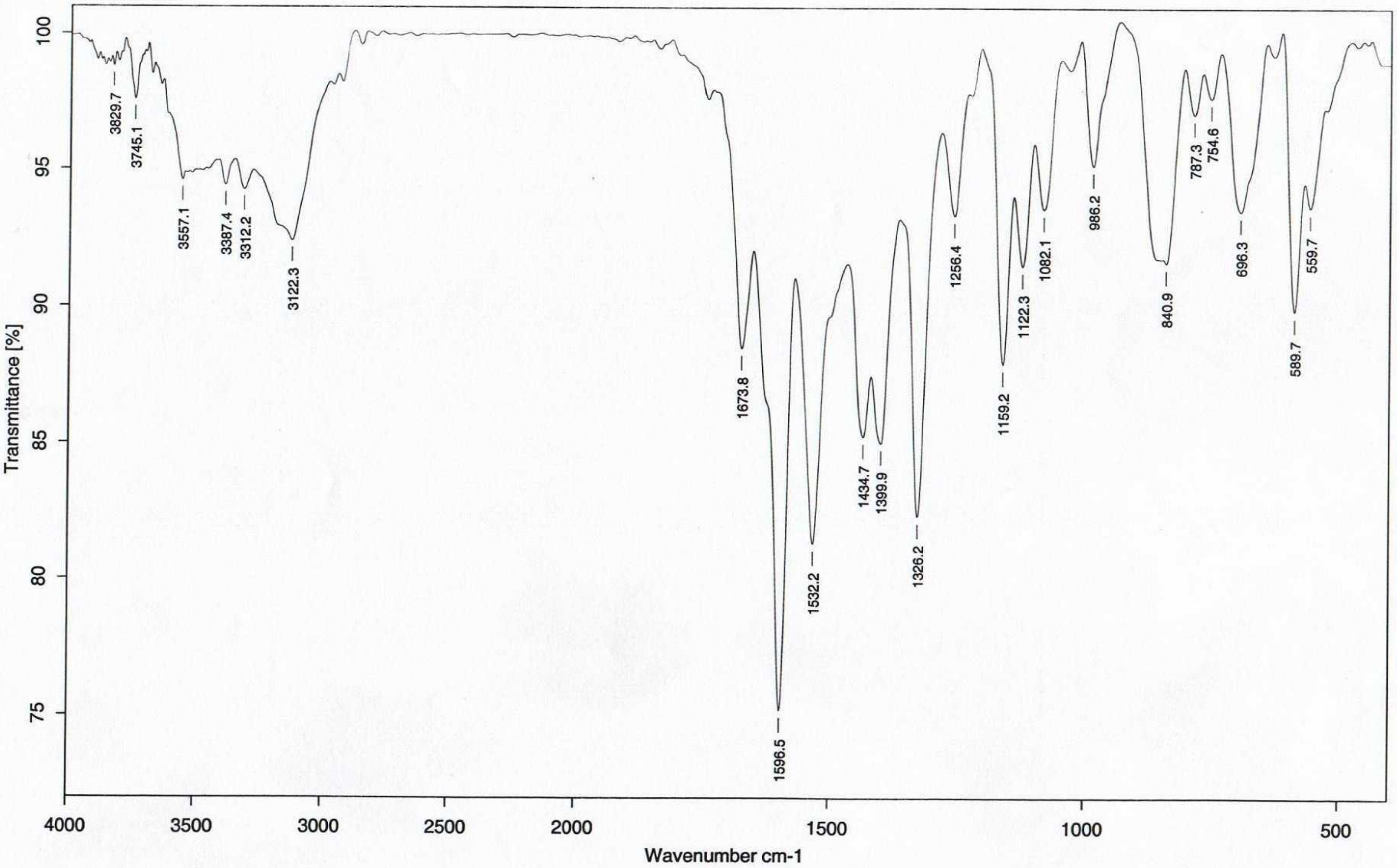

Sample : MHH-17/Dr.Haroon

Measured : 30/01/2017 on VECTOR22

Resolution : 4 cm-1 (10 scans)

Spectrum : MHH-17.0 (in D:\IRSTUDENT)

Technic : Solid

Analyst : Zubair Ahmad/ Jamshed

THERMO ELECTRON ~ VISIONpro SOFTWARE V4.10

Operator Name	ARSHAD ALAM.	Date of Report	1/31/2017
Department	Analytical Laboratory TWC # 004	Time of Report	10:04:15AM
Organization	ICCBS Karachi of Universty.		
Information	Dr Haroon/ Dr Hina		

Scan Graph

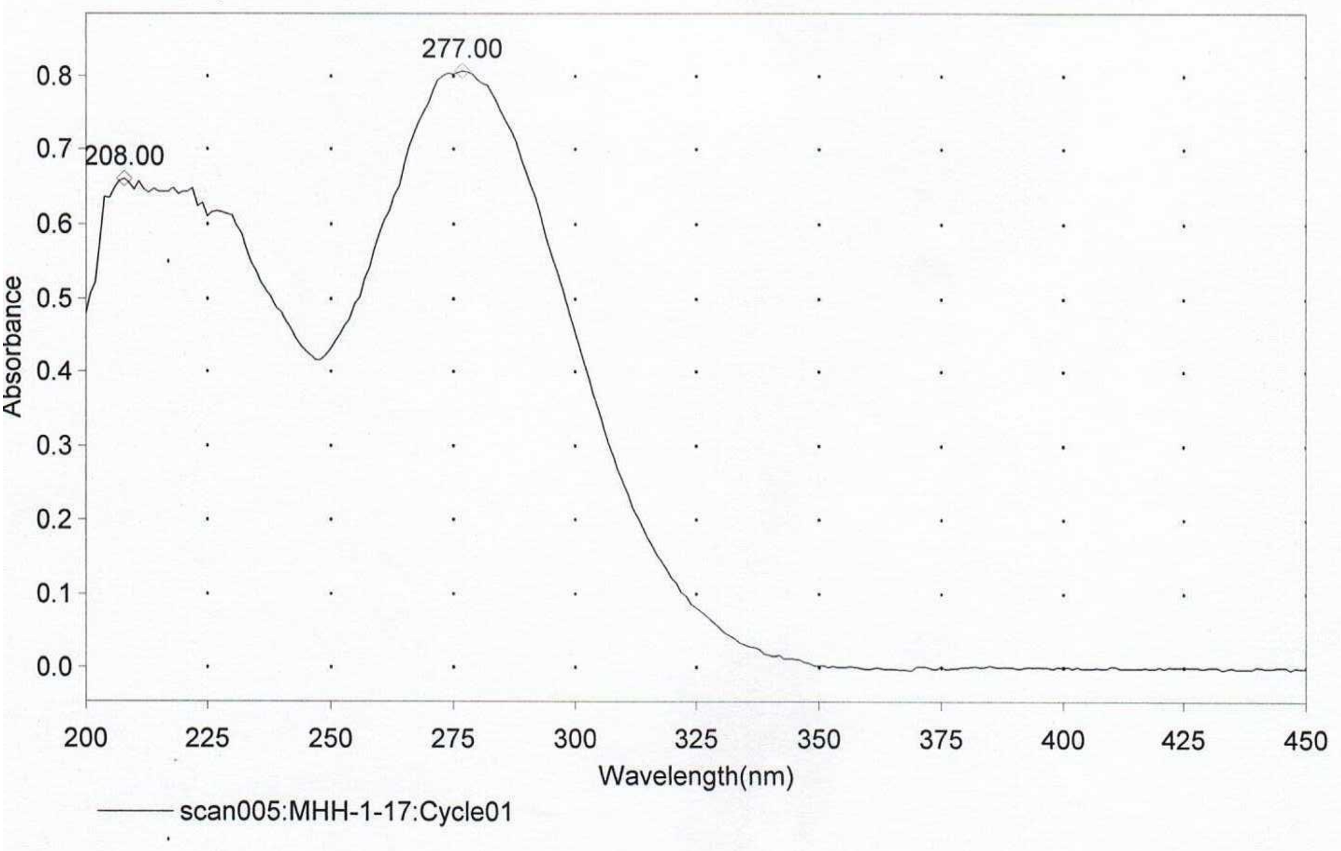

Results Table - MHH-1-17.sre,MHH-1-17,Cycle01

nm	A	**Peak Pick Method**
208.00	0.661	Find 8 Peaks Above -3.0000 A
277.00	0.807	Start Wavelength 200.00 nm
		Stop Wavelength 450.00 nm
		Sort By Wavelength

Sensitivity Auto
