# Supplementary material for: Sulphamethazine derivatives as immunomodulating agents: New therapeutic strategies for inflammatory diseases
Source: PLoS One. 2018 Dec 19;13(12):e0208933. doi: 10.1371/journal.pone.0208933 (PMC6300282; doi:10.1371/journal.pone.0208933)
Supplement: S6 Fig — (PDF) [file pone.0208933.s006.pdf]

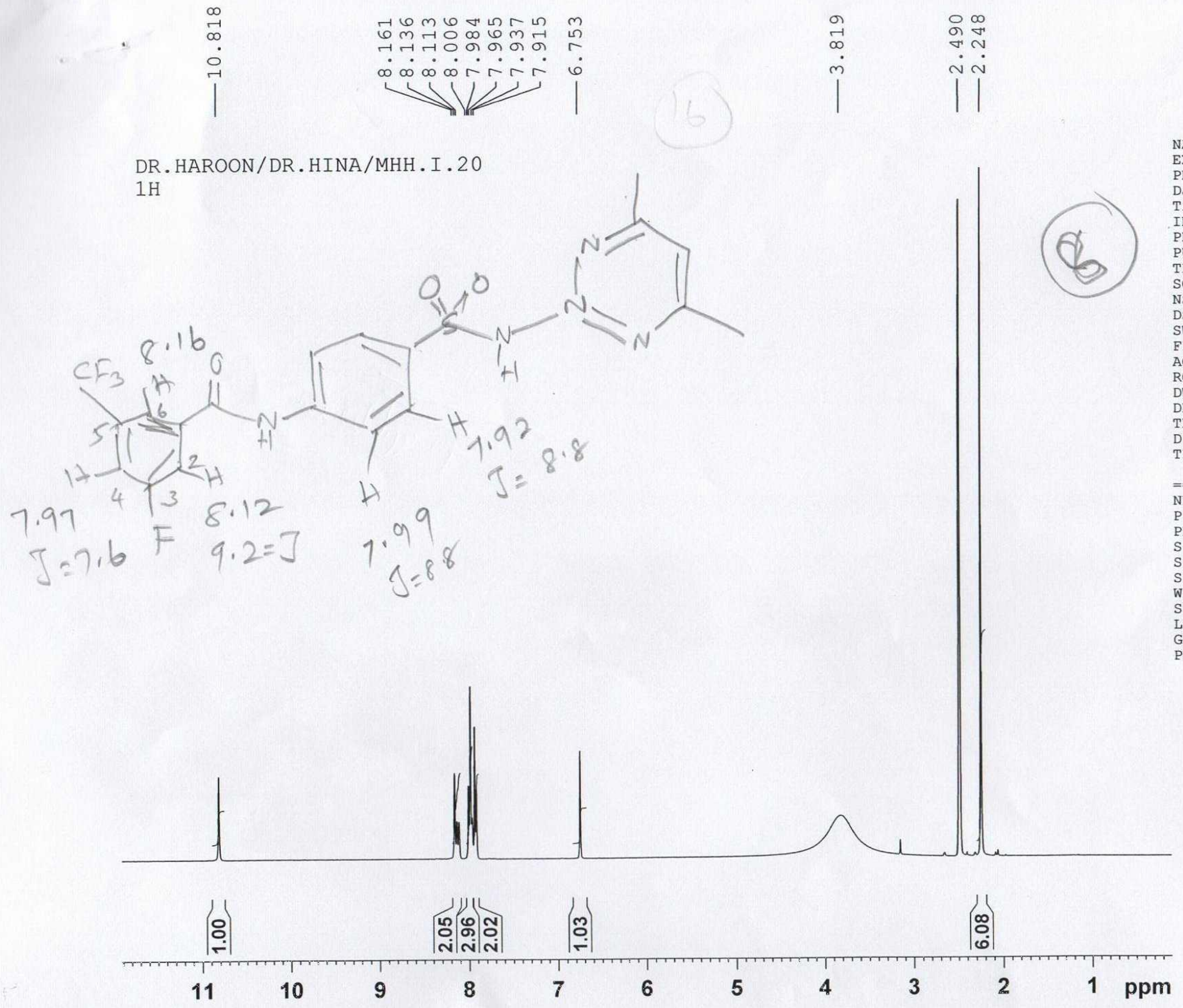

AVANCE AV-400 MHz
Lab # 115

NAME	dec30-16
EXPNO	10
PROCNO	1
Date_	20161230
Time	11.46
INSTRUM	spect
PROBHD	5 mm SEI 1H-13
PULPROG	zg30
TD	65536
SOLVENT	DMSO
NS	64
DS	0
SWH	8012.820 Hz
FIDRES	0.122266 Hz
AQ	4.0894966 sec
RG	574.7
DW	62.400 usec
DE	6.50 usec
TE	300.0 K
D1	2.00000000 sec
TD0	1

======== CHANNEL f1 ========

NUC1	1H
P1	10.80 usec
PL1	3.00 dB
SFO1	400.0332002 MHz
SI	32768
SF	400.0300041 MHz
WDW	EM
SSB	0
LB	0.30 Hz
GB	0
PC	1.00

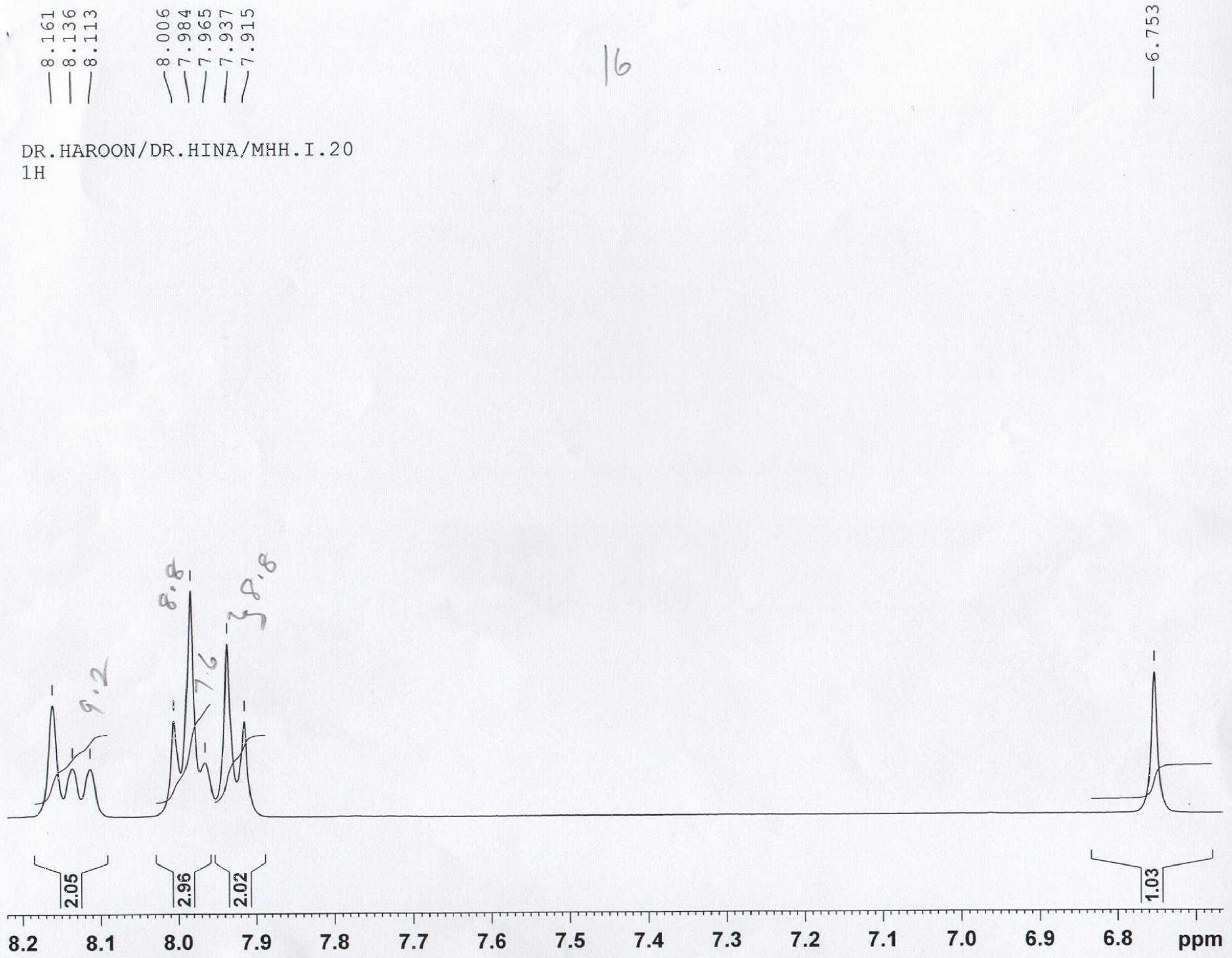
8.161
8.136
8.113
8.006
7.984
7.965
7.937
7.915
16
6.753
DR.HAROON/DR.HINA/MHH.I.20
1H
2.05
2.96
2.02
1.03
8.2
8.1
8.0
7.9
7.8
7.7
7.6
7.5
7.4
7.3
7.2
7.1
7.0
6.9
6.8
ppm

DR,M HAROON/DR,HINA/MHH-I-20/
ICCBS,U.O.K/BB

AVANCE 400
LAB NO 117

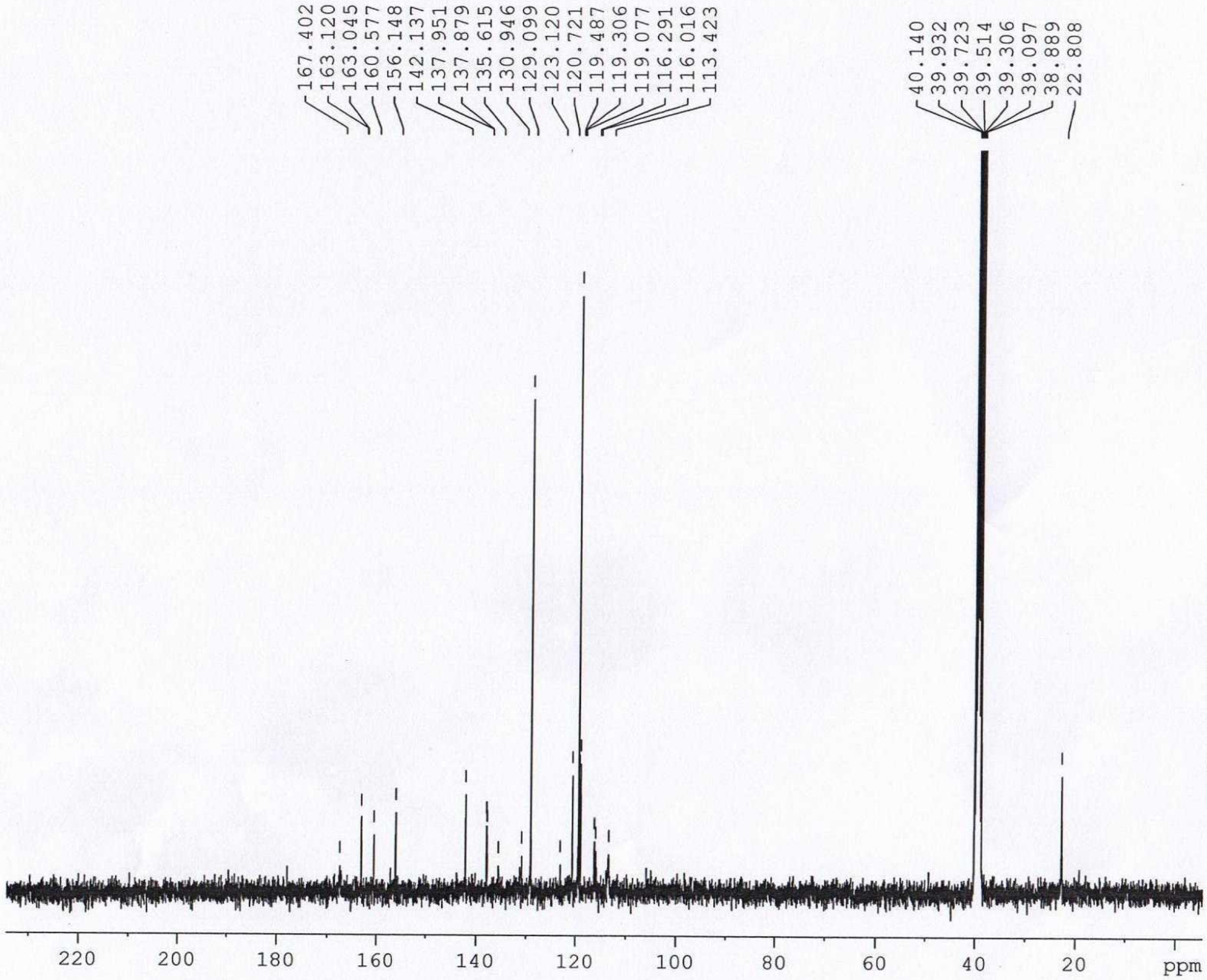

NAME	apr17-17
EXPNO	12
PROCNO	1
Date_	20170417
Time	15.40
INSTRUM	spect
PROBHD	5 mm DUL 13C-1
PULPROG	zgpg
TD	32768
SOLVENT	DMSO
NS	18432
DS	0
SWH	24154.590 Hz
FIDRES	0.737140 Hz
AQ	0.6783476 sec
RG	32768
DW	20.700 usec
DE	6.50 usec
TE	300.0 K
D1	2.00000000 sec
D11	0.03000000 sec
TD0	18
======== CHANNEL f1 ========	
NUC1	13C
P1	8.55 usec
PL1	7.00 dB
SFO1	100.6243395 MHz
======== CHANNEL f2 ========	
CPDPRG2	waltz16
NUC2	1H
PCPD2	80.00 usec
PL2	0.00 dB
PL12	19.00 dB
PL13	20.00 dB
SFO2	400.1324008 MHz
SI	16384
SF	100.6128195 MHz
WDW	EM
SSB	0
LB	1.00 Hz
GB	0
PC	0.80

DR,M HAROON/DR,HINA/MHH-I-20/
ICCBS,U.O.K/BB

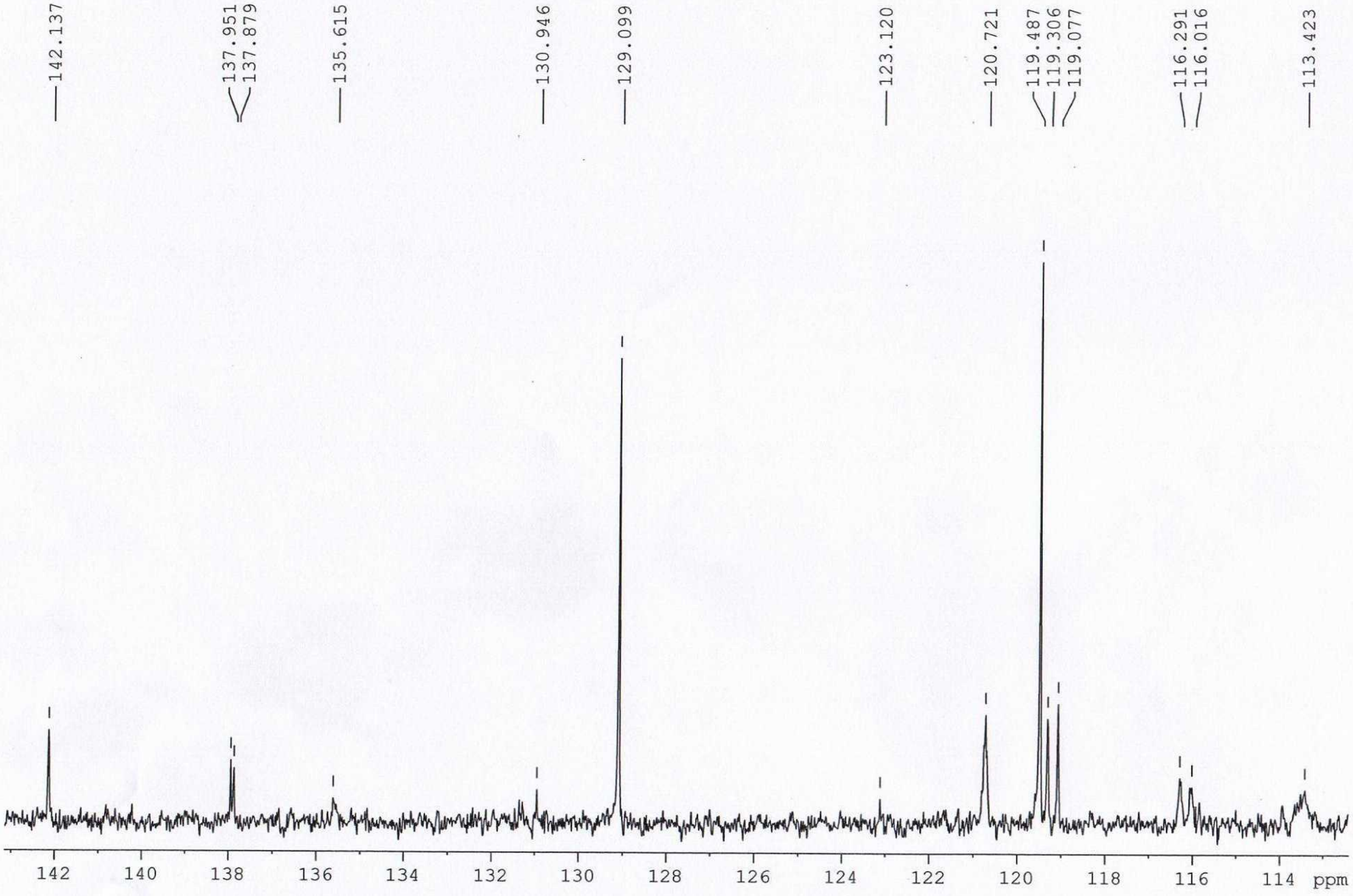

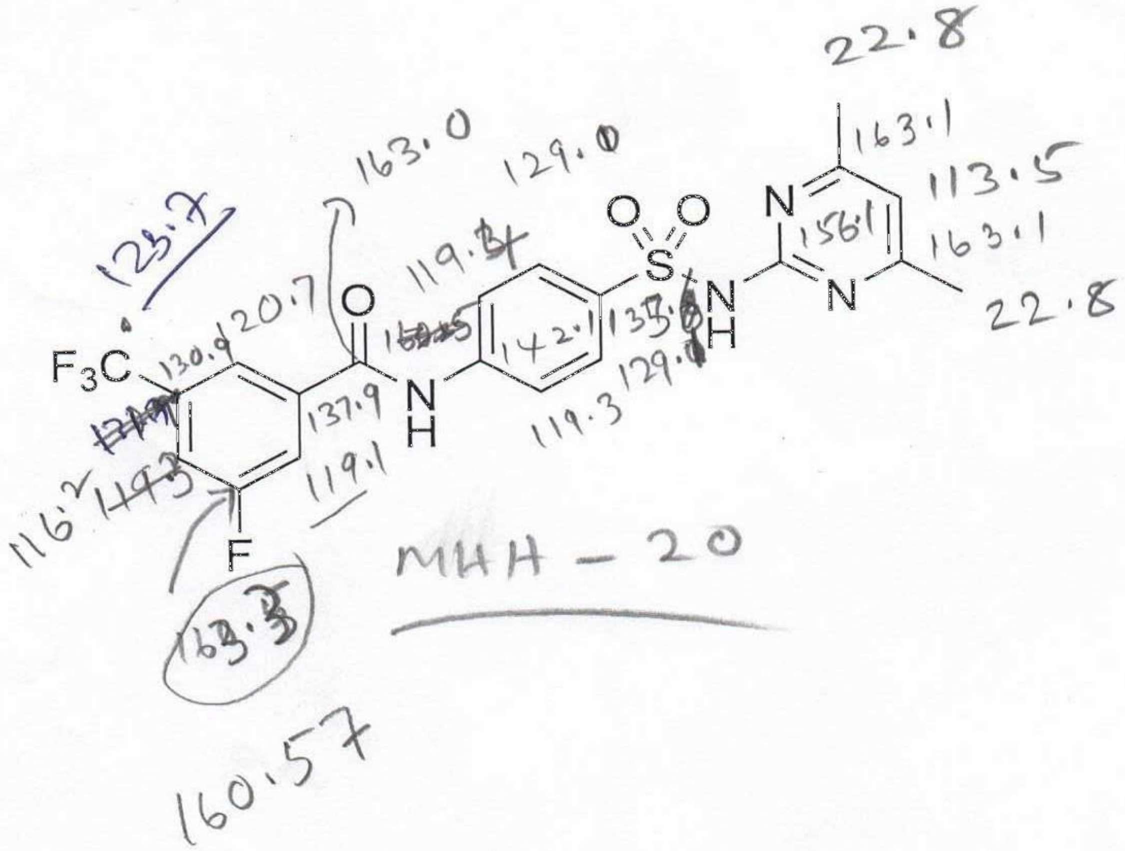

MHH - 20

0332- 3501076

Kamran

Sharmeen.

JEOL HX 110 MASS SPECTROMETER (FAB-HR)

STUDENT NAME	Dr. M.H. Haroon	SAMPLE CODE	DATE	24/5/17
SUPERVISOR NAME	Dr. Hina	MHH-I-20	FAB (+VE / -VE)	FAB+ve

Mass	Theoretical Mass	Delta [ppm]	Delta [mmu]	RDB	Composition
469.0979	469.0977	0.4	0.2	28.5	$C_{33} H_{13} O_2 N_2$
	469.0986	-1.6	-0.7	20.5	$C_{28} H_{16} N_2 F_3 S_1$
	469.0988	-2.0	-0.9	24.5	$C_{30} H_{14} O_3 N_2 F_1$
	469.0964	3.2	1.5	21.5	$C_{28} H_{13} O_1 N_2 F_4$
	469.0998	-4.0	-1.9	16.5	$C_{25} H_{17} O_1 N_2 F_4 S_1$
	469.0959	4.2	2.0	16.0	$C_{25} H_{18} O_3 N_1 F_3 S_1$
	469.0957	4.6	2.2	12.5	→ $C_{20} H_{17} O_3 N_4 F_4 S_1$
	469.0953	5.6	2.6	25.5	$C_{31} H_{12} N_2 F_3$
	469.0948	6.6	3.1	20.0	$C_{28} H_{17} O_2 N_1 F_2 S_1$
	469.1011	-6.8	-3.2	23.5	$C_{30} H_{17} O_2 N_2 S_1$

HEJ-ICCBS
2/15/2017 9:37:57 AM

File: MHH-I-20
Sample: DR.M.H.HAROON /DR. HINA
Instrument: JEOL MS 600H-1

Date Run: 02-15-2017 (Time Run: 09:26:18)

Ionization mode: EI+

Scan: 13-17
Base: m/z 403; 88.2%FS TIC: 7881238

R.T.: 1.24

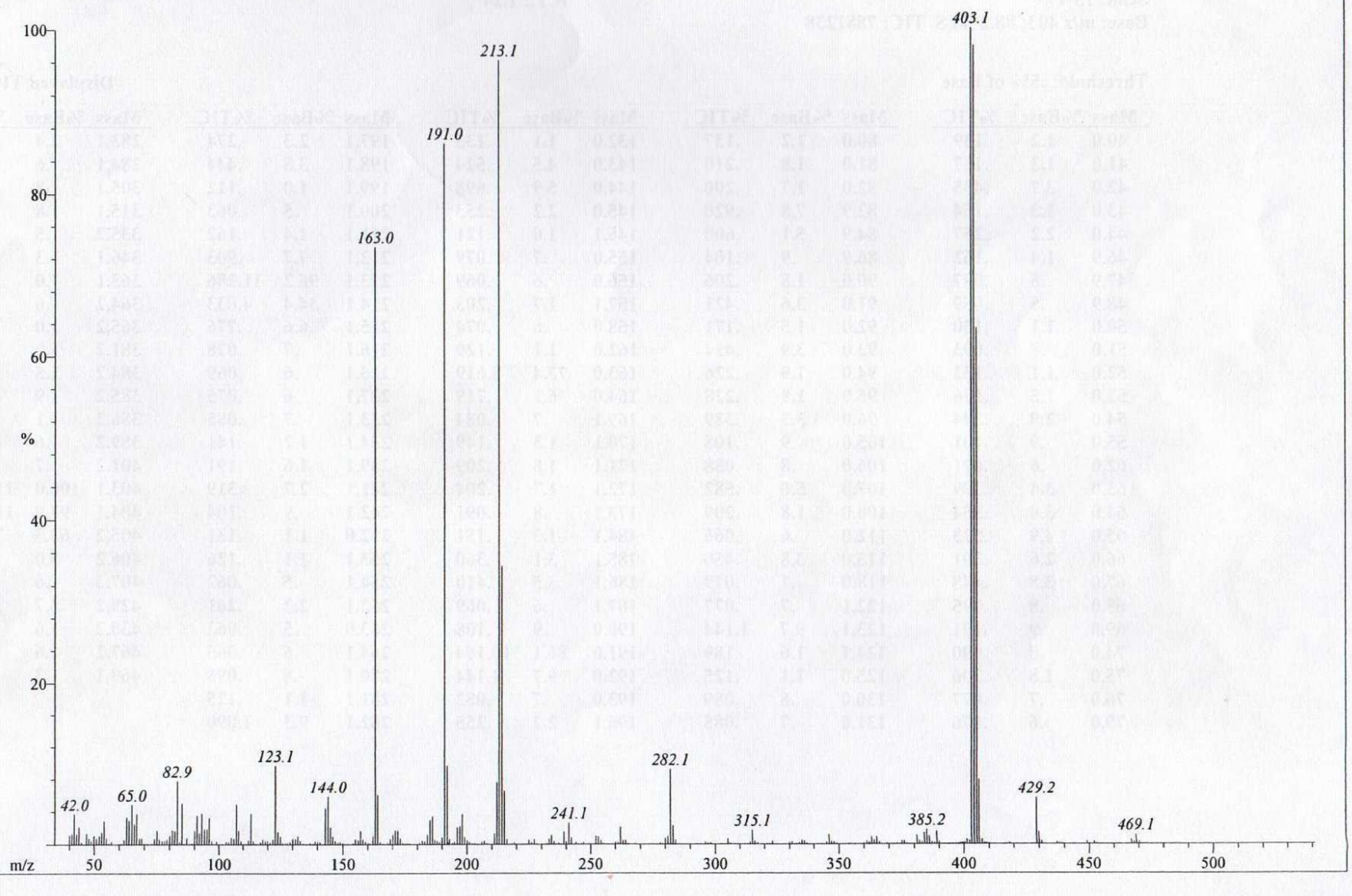

I. C.C.B.S., University of Karachi Analytical Laboratory - Pakistan

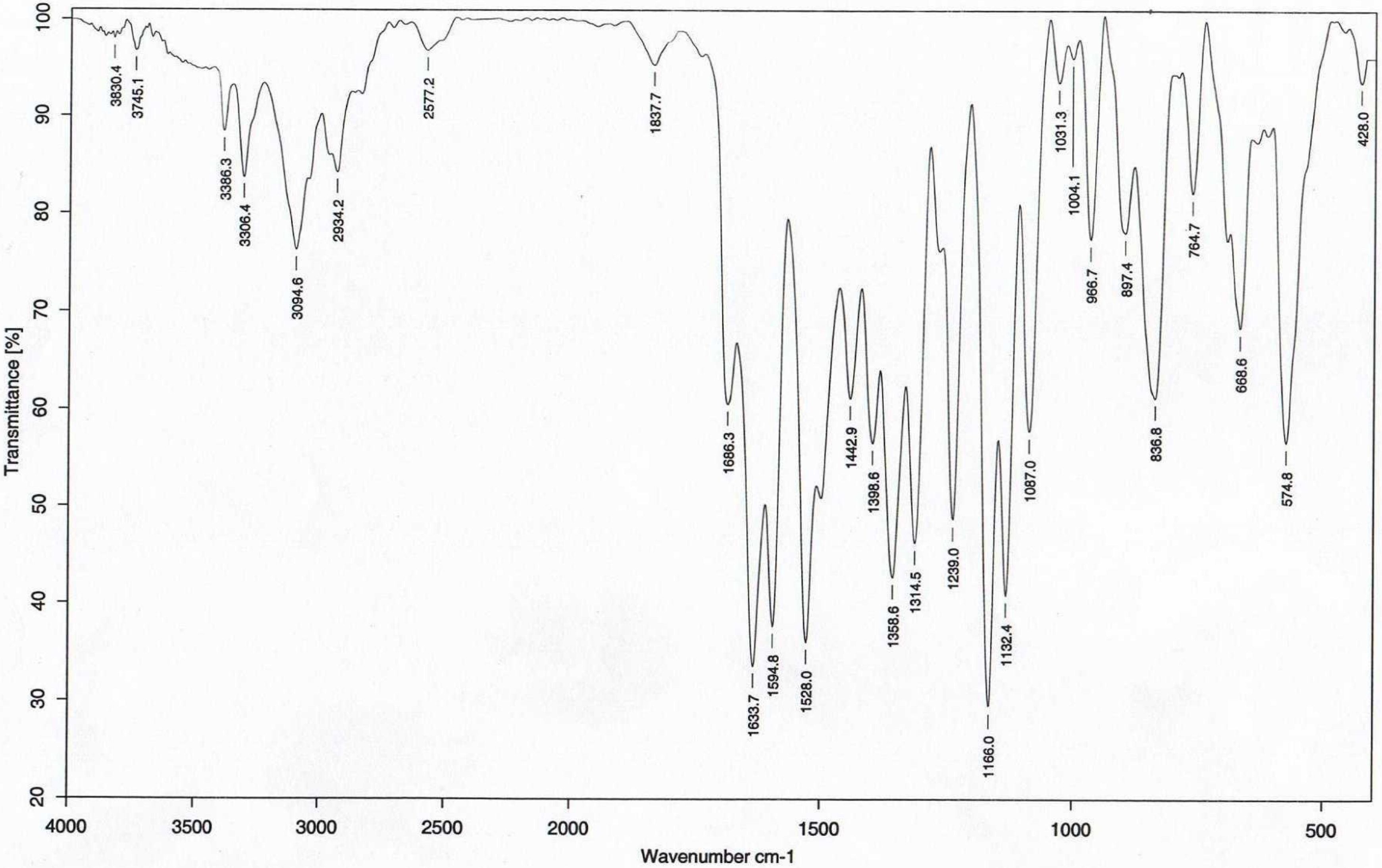

Sample : MHH-1-20/Haroon/Dr. Hina	Spectrum : MHH-1-20.0 (in D:\IRSTUDENT)
Measured : 01/02/2017 on VECTOR22	Technic : Solid
Resolution : 4 cm-1 (10 scans)	Analyst : ZA/Jamshed/M. Asif/Haroon

THERMO ELECTRON ~ VISIONpro SOFTWARE V4.10

Operator Name	ARSHAD ALAM.	Date of Report	2/2/2017
Department	Analytical Laboratory TWC # 004	Time of Report	3:10:12PM
Organization	ICCBS Karachi of Universty.		
Information	Dr.Haroon/ Dr.Hina		

Scan Graph

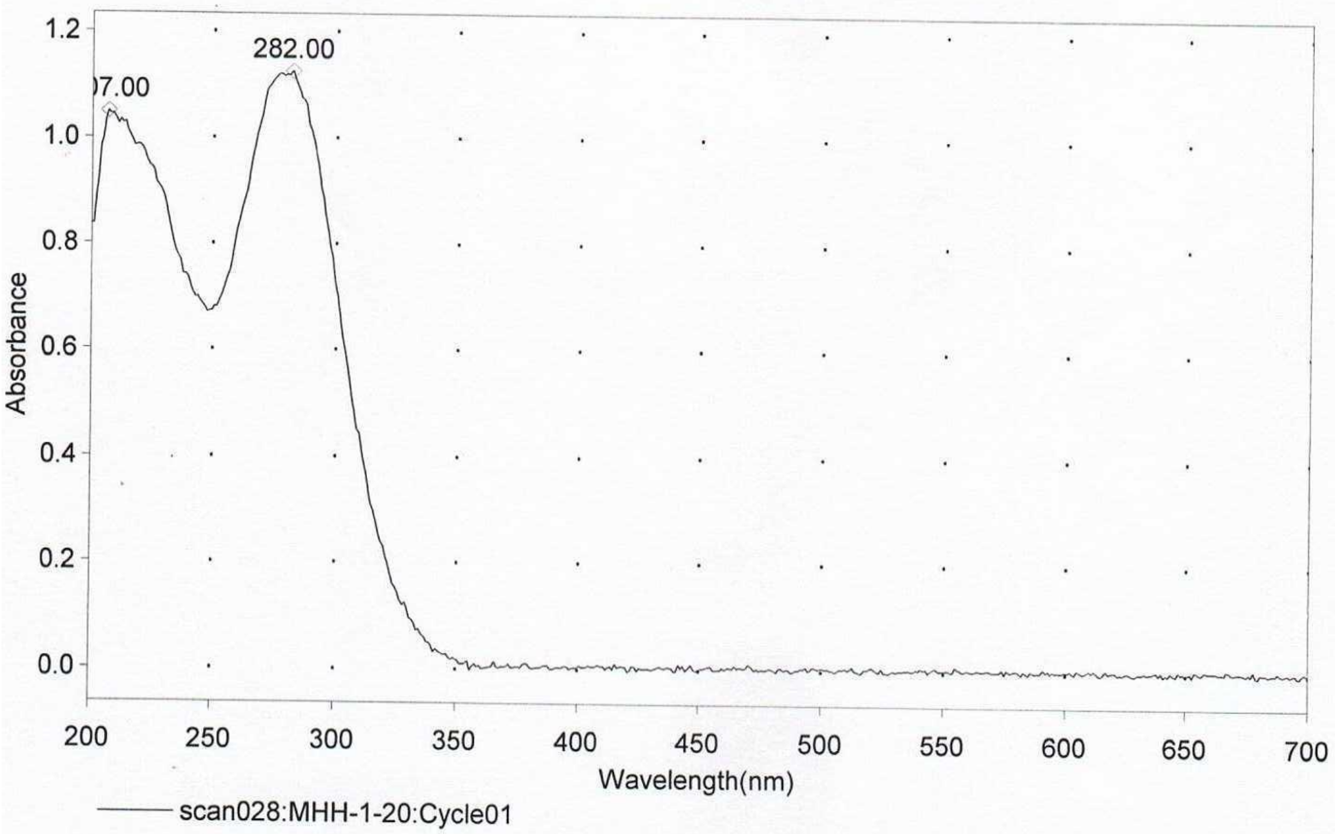

Results Table - MHH-1-20.sre,MHH-1-20,Cycle01

ım	A	**Peak Pick Method**
:07.00	1.048	Find 8 Peaks Above -3.0000 A
:82.00	1.125	Start Wavelength 200.00 nm
		Stop Wavelength 700.00 nm
		Sort By Wavelength

Sensitivity Auto
