# Supplementary material for: Sulphamethazine derivatives as immunomodulating agents: New therapeutic strategies for inflammatory diseases
Source: PLoS One. 2018 Dec 19;13(12):e0208933. doi: 10.1371/journal.pone.0208933 (PMC6300282; doi:10.1371/journal.pone.0208933)

DR.HAROON/DR.HINA/MHH.I.23
1H

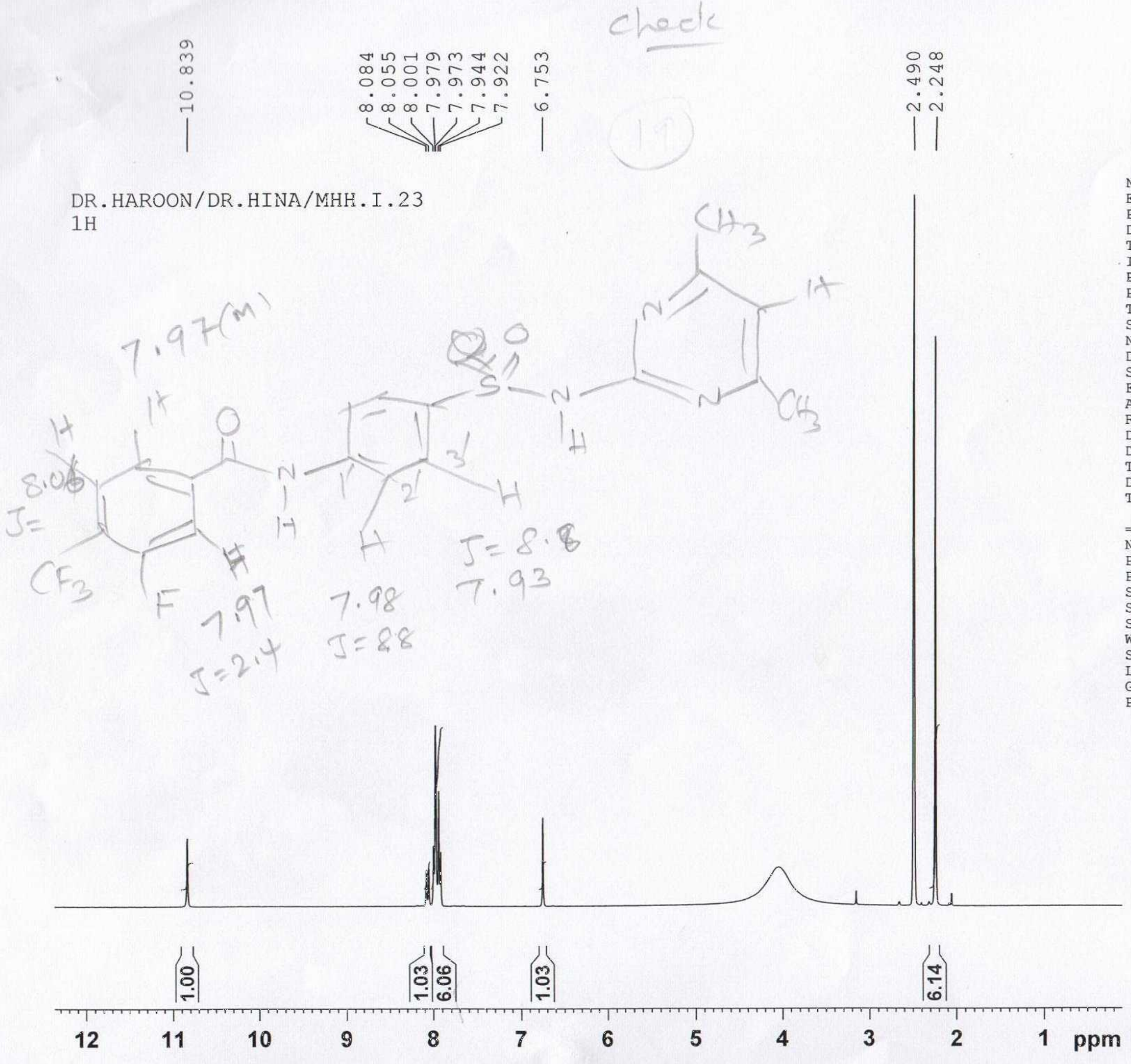

AVANCE AV-400 MHz
Lab # 115

NAME	dec30-16
EXPNO	7
PROCNO	1
Date_	20161230
Time	11.18
INSTRUM	spect
PROBHD	5 mm SEI 1H-13
PULPROG	zg30
TD	65536
SOLVENT	DMSO
NS	64
DS	0
SWH	8012.820 Hz
FIDRES	0.122266 Hz
AQ	4.0894966 sec
RG	574.7
DW	62.400 usec
DE	6.50 usec
TE	300.0 K
D1	2.00000000 sec
TD0	1

======== CHANNEL f1 ========

NUC1	1H
P1	10.80 usec
PL1	3.00 dB
SFO1	400.0332002 MHz
SI	32768
SF	400.0300041 MHz
WDW	EM
SSB	0
LB	0.30 Hz
GB	0
PC	1.00

(9)

DR.HAROON/DR.HINA/MHH.I.23
1H

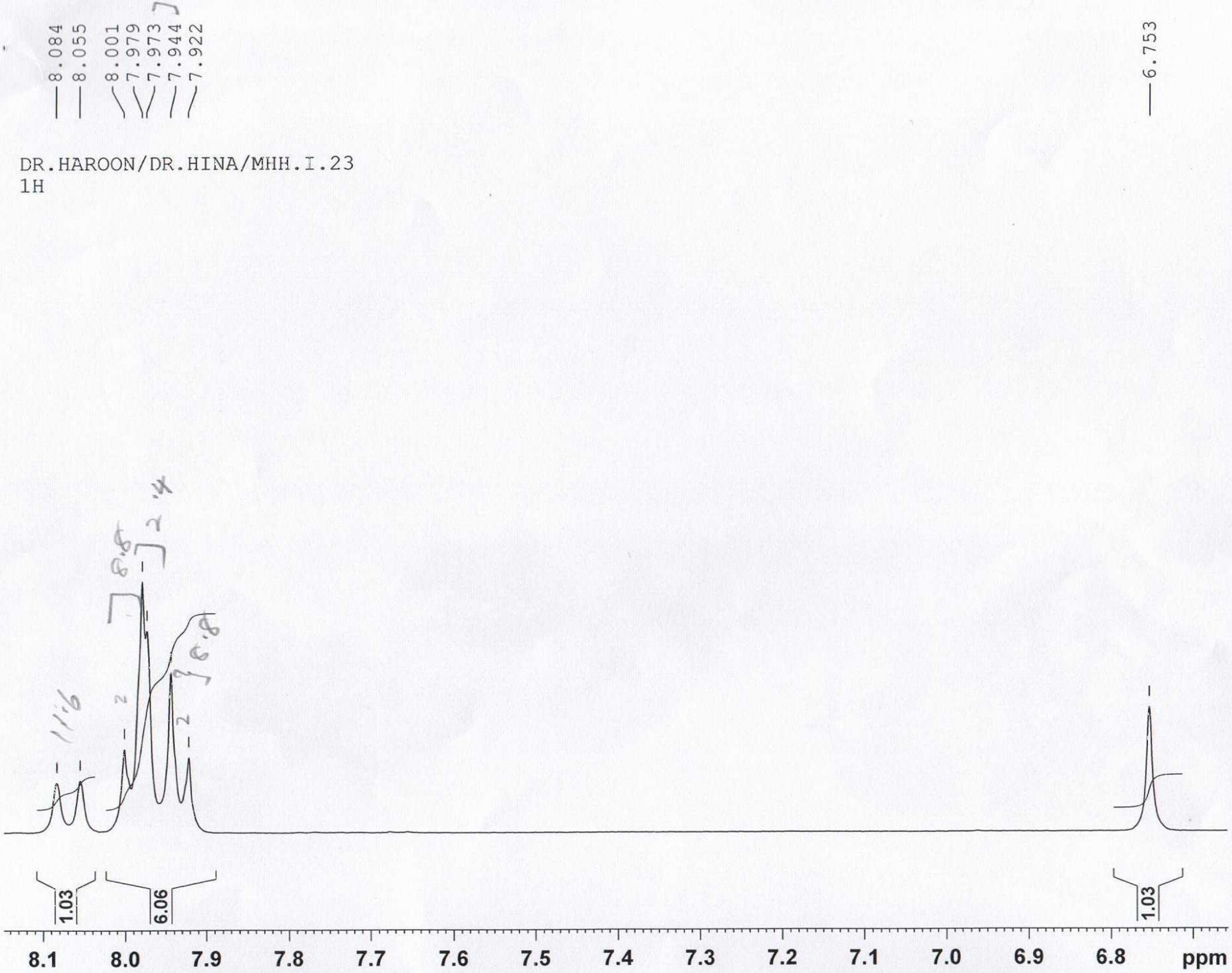

DR.M.H.HAROON/DR.HINA/23/DMSO
ICCBS,U.O.K/BB

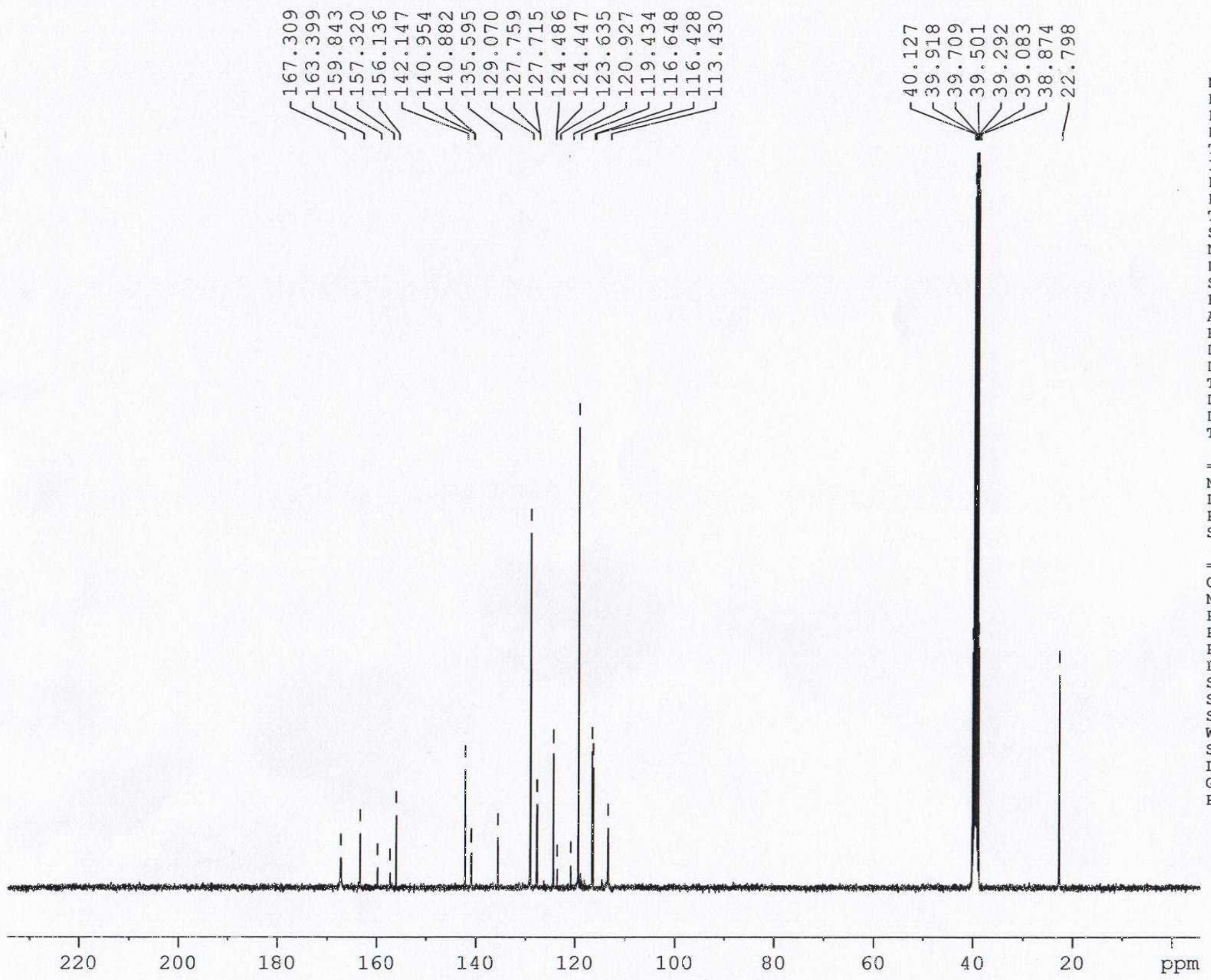

AVANCE 400
LAB NO 117

NAME	JuLY17-17
EXPNO	6
PROCNO	1
Date_	20170717
Time	15.43
INSTRUM	spect
PROBHD	5 mm DUL 13C-1
PULPROG	zgpg
TD	32768
SOLVENT	DMSO
NS	20480
DS	0
SWH	24154.590 Hz
FIDRES	0.737140 Hz
AQ	0.6783476 sec
RG	32768
DW	20.700 usec
DE	6.50 usec
TE	300.0 K
D1	1.50000000 sec
D11	0.03000000 sec
TD0	20

======== CHANNEL f1 ========

NUC1	13C
P1	8.55 usec
PL1	7.00 dB
SFO1	100.6243395 MHz

======== CHANNEL f2 ========

CPDPRG2	waltz16
NUC2	1H
PCPD2	80.00 usec
PL2	0.00 dB
PL12	19.00 dB
PL13	20.00 dB
SFO2	400.1324008 MHz
SI	16384
SF	100.6128205 MHz
WDW	EM
SSB	0
LB	1.00 Hz
GB	0
PC	1.00

DR.M.H.HAROON/DR.HINA/23/DMSO
ICCBS,U.O.K/BB

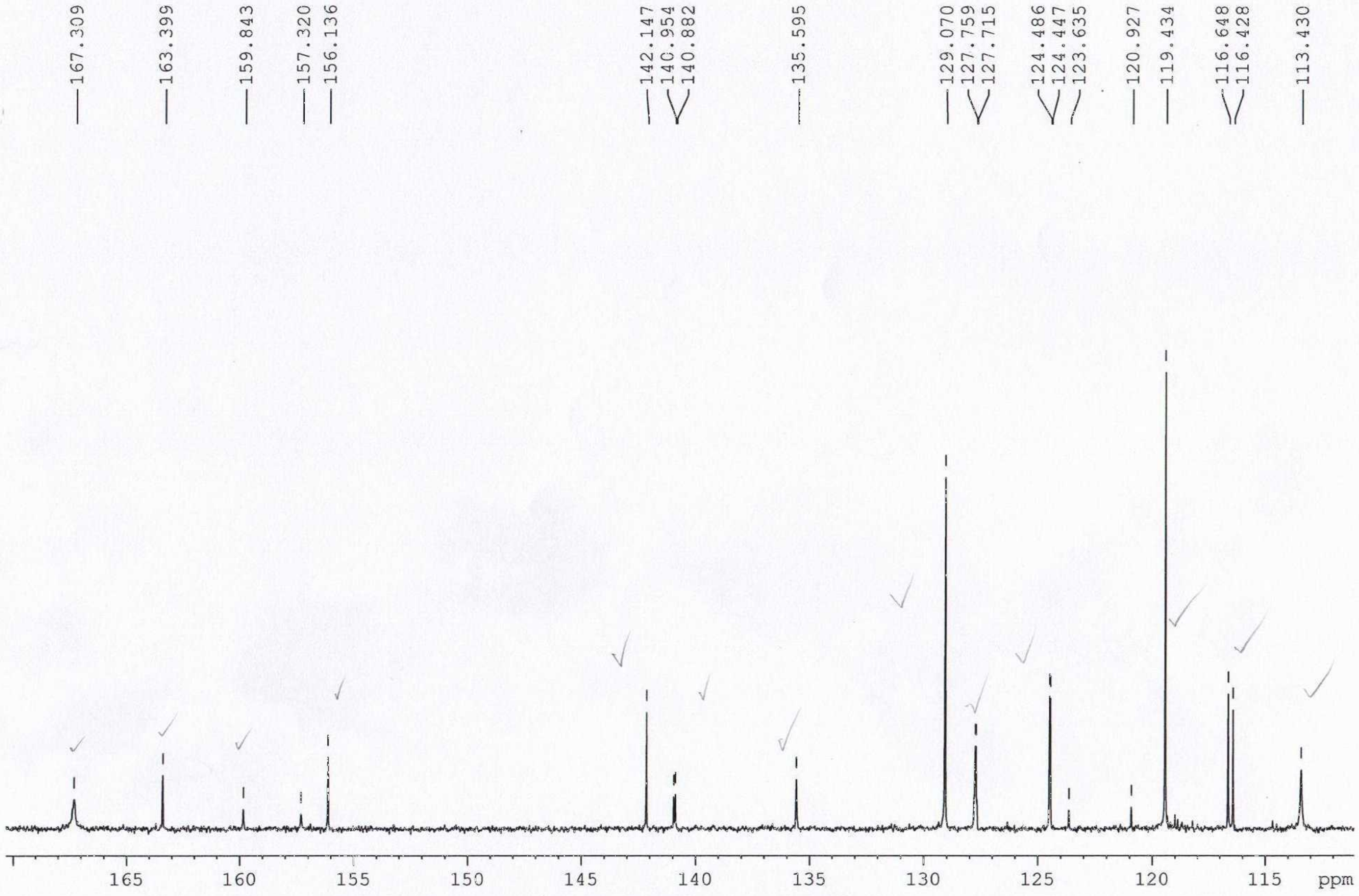

DR.M.H.HAROON/DR.HINA/23/DMSO
ICCBS,U.O.K/DEPT-135

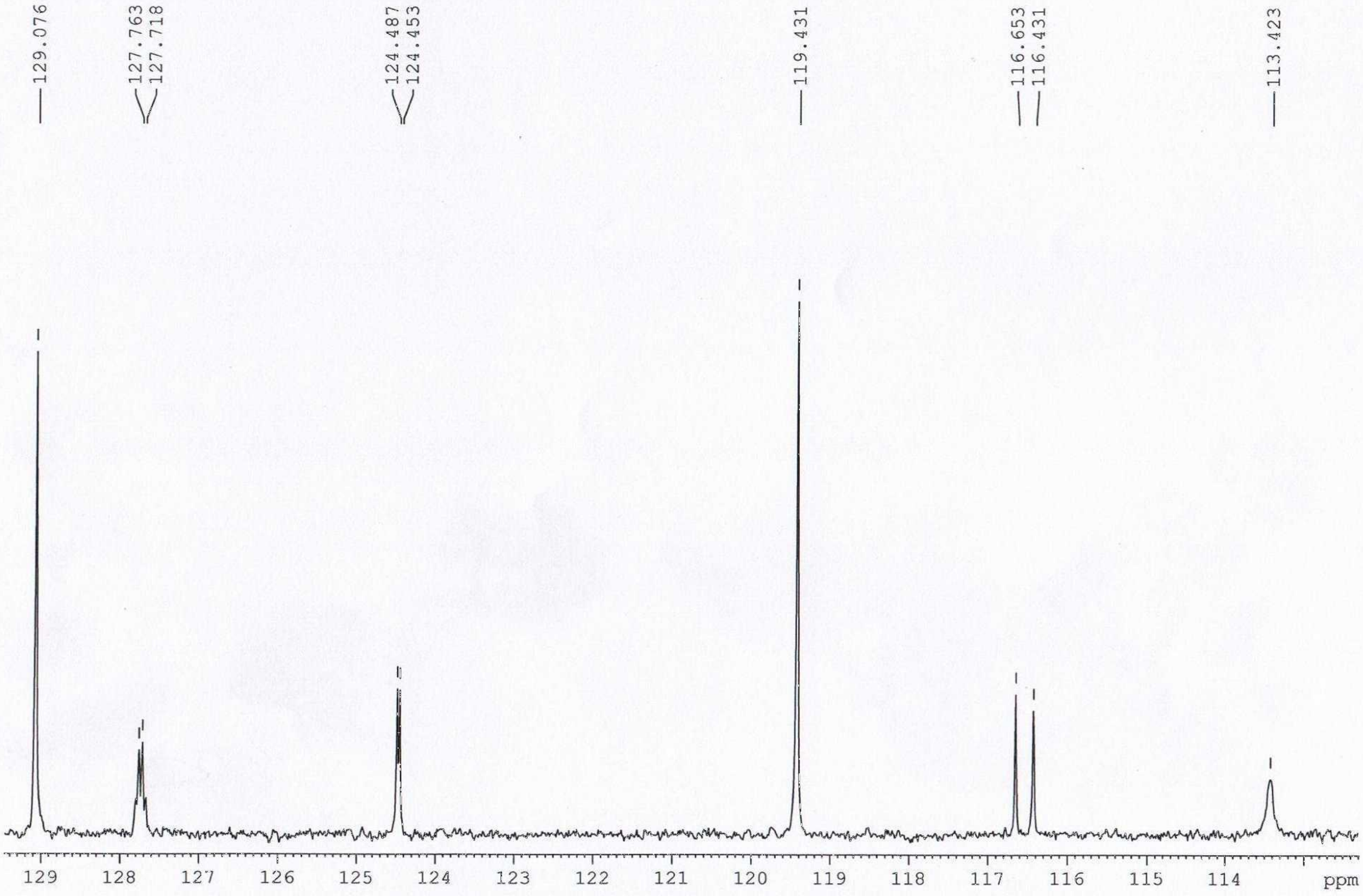

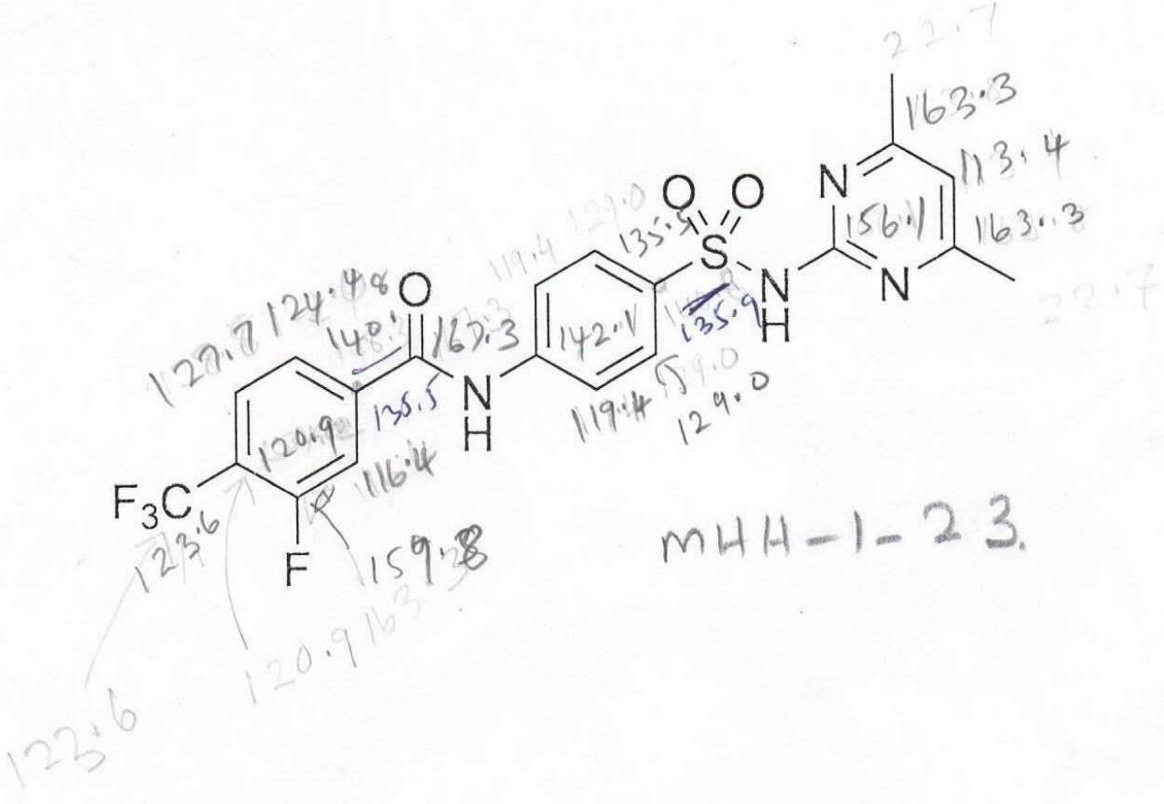

MHH-1-23.

~~must Discuss~~

JEOL HX 110 MASS SPECTROMETER (FAB-HR)

STUDENT NAME	Dr. M. H. Haroon	SAMPLE CODE	DATE	24-05-2017
SUPERVISOR NAME	Dr. Hina	MHH-I-23	FAB (+VE / -VE)	FAB+Ve

Mass	Theoretical Mass	Delta [ppm]	Delta [mmu]	RDB	Composition
469.0980	469.0977	0.6	0.3	28.5	$C_{33} H_{13} O_{2} N_{2}$
	469.0986	-1.3	-0.6	20.5	$C_{28} H_{16} N_{2} F_{3} S_{1}$
	469.0988	-1.8	-0.8	24.5	$C_{30} H_{14} O_{3} N_{2} F_{1}$
	469.0964	3.4	1.6	21.5	$C_{28} H_{13} O_{1} N_{2} F_{4}$
	469.0998	-3.8	-1.8	16.5	$C_{25} H_{17} O_{1} N_{2} F_{4} S_{1}$
	469.0959	4.4	2.1	16.0	$C_{25} H_{18} O_{3} N_{1} F_{3} S_{1}$
	469.0957	4.8	2.3	12.5	→ $C_{20} H_{17} O_{3} N_{4} F_{4} S_{1}$
	469.0953	5.8	2.7	25.5	$C_{31} H_{12} N_{2} F_{3}$
	469.1011	-6.6	-3.1	23.5	$C_{30} H_{17} O_{2} N_{2} S_{1}$
	469.0948	6.8	3.2	20.0	$C_{28} H_{17} O_{2} N_{1} F_{2} S_{1}$
	469.0946	7.2	3.4	16.5	$C_{23} H_{16} O_{2} N_{4} F_{3} S_{1}$
	469.1015	-7.5	-3.5	29.0	$C_{33} H_{12} N_{3} F_{1}$

HEJ-ICCBS
2/4/2017 3:24:26 PM

File: MHH-I-23-
Sample: DR.MH.HAROON /DR. HINA
Instrument: JEOL MS 600H-1

Date Run: 02-04-2017 (Time Run: 15:15:53)

Ionization mode: EI+

Scan: 28
Base: m/z 403; 84.5%FS TIC: 3600298

R.T.: 2.38

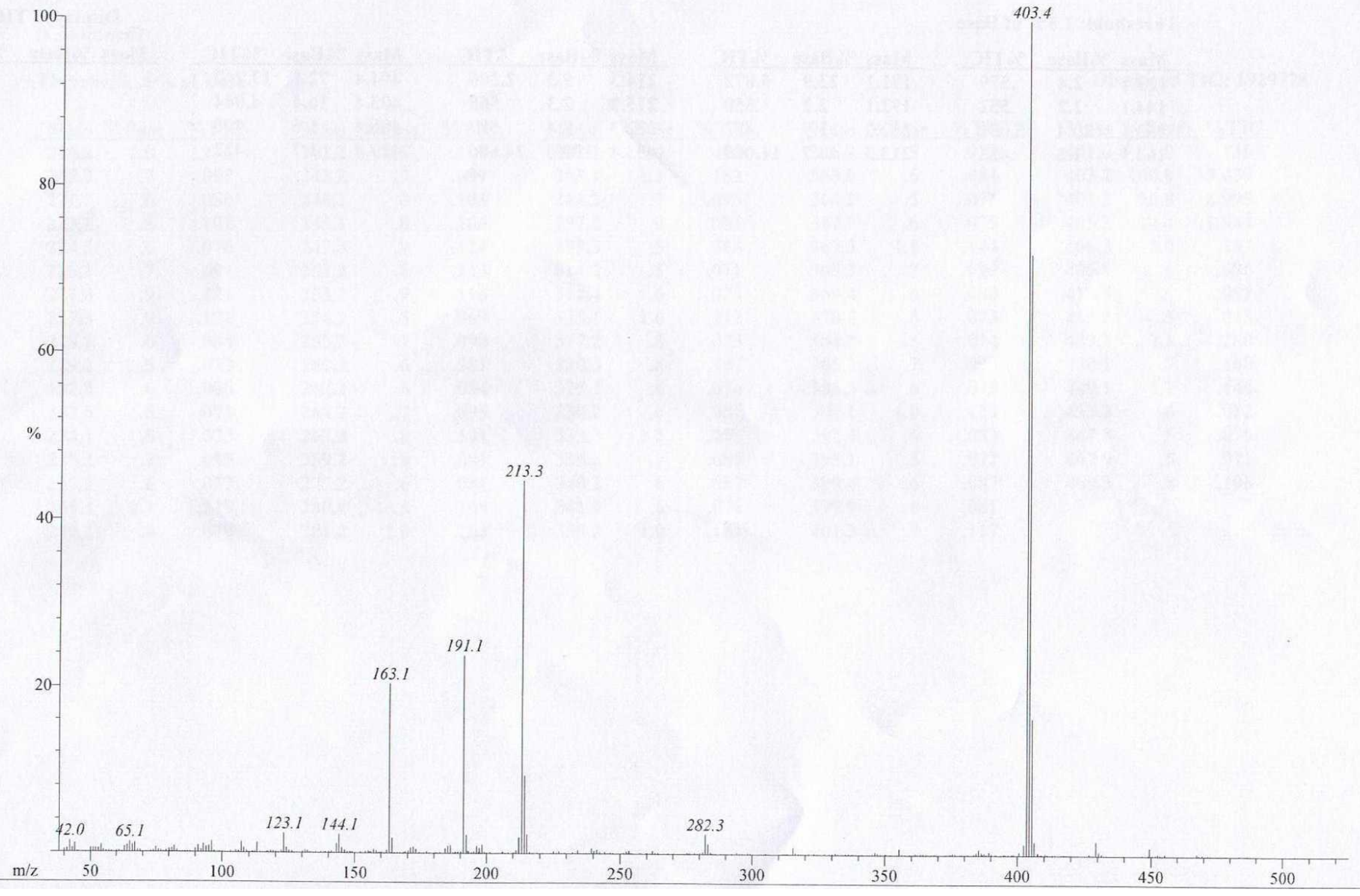

Supplement: S7 Fig — (PDF) [file pone.0208933.s007.pdf]
