# Supplementary material for: Sulphamethazine derivatives as immunomodulating agents: New therapeutic strategies for inflammatory diseases
Source: PLoS One. 2018 Dec 19;13(12):e0208933. doi: 10.1371/journal.pone.0208933 (PMC6300282; doi:10.1371/journal.pone.0208933)
Supplement: S8 Fig — (PDF) [file pone.0208933.s008.pdf]

AVANCE AV-400 MHz
Lab # 115

DR.HAROON/DR.HINA/MHH.I.21
1H

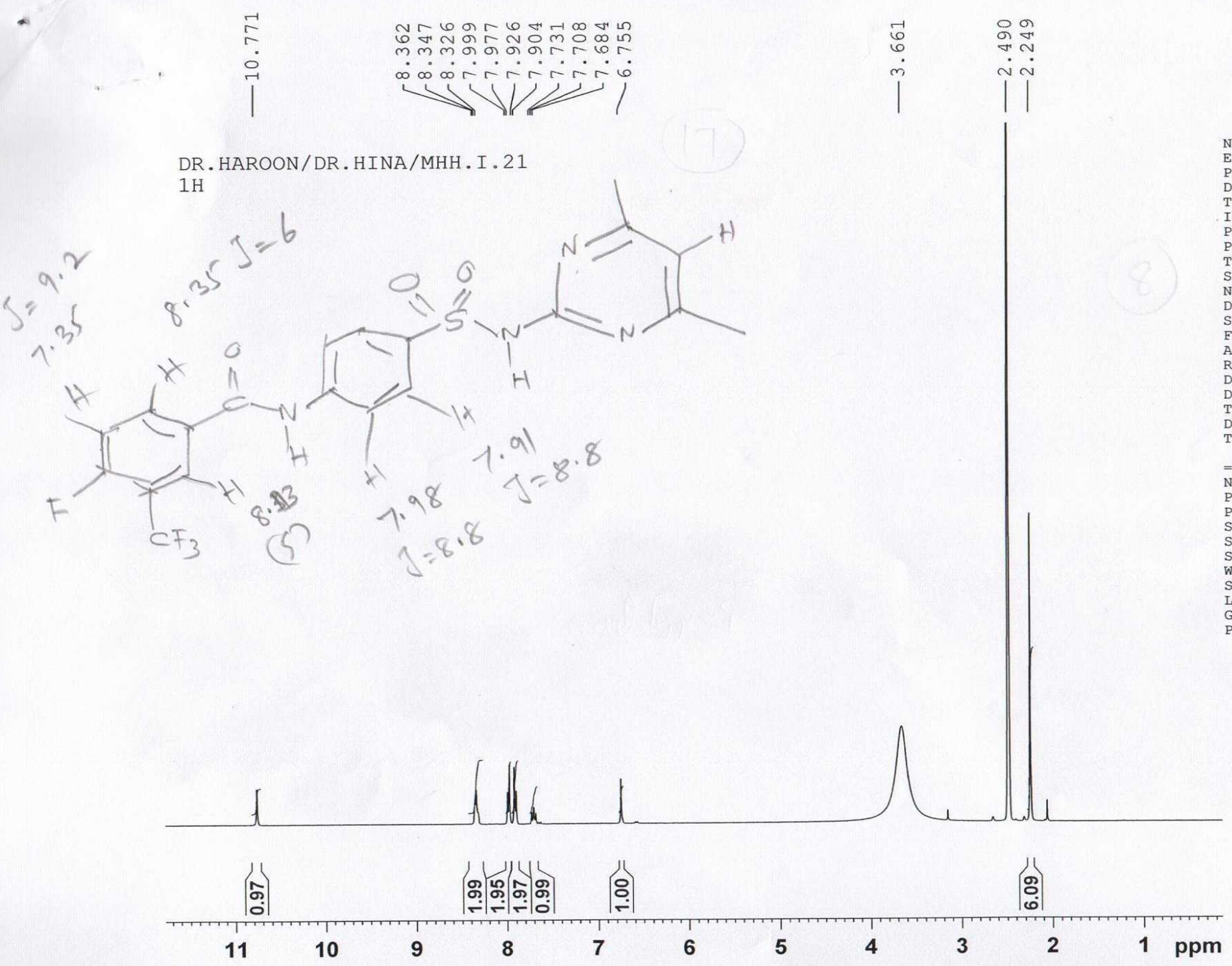

NAME	dec30-16
EXPNO	9
PROCNO	1
Date_	20161230
Time	11.37
INSTRUM	spect
PROBHD	5 mm SEI 1H-13
PULPROG	zg30
TD	65536
SOLVENT	DMSO
NS	64
DS	0
SWH	8012.820 Hz
FIDRES	0.122266 Hz
AQ	4.0894966 sec
RG	1149.4
DW	62.400 usec
DE	6.50 usec
TE	300.0 K
D1	2.00000000 sec
TD0	1

======== CHANNEL f1 ========

NUC1	1H
P1	10.80 usec
PL1	3.00 dB
SFO1	400.0332002 MHz
SI	32768
SF	400.0300041 MHz
WDW	EM
SSB	0
LB	0.30 Hz
GB	0
PC	1.00

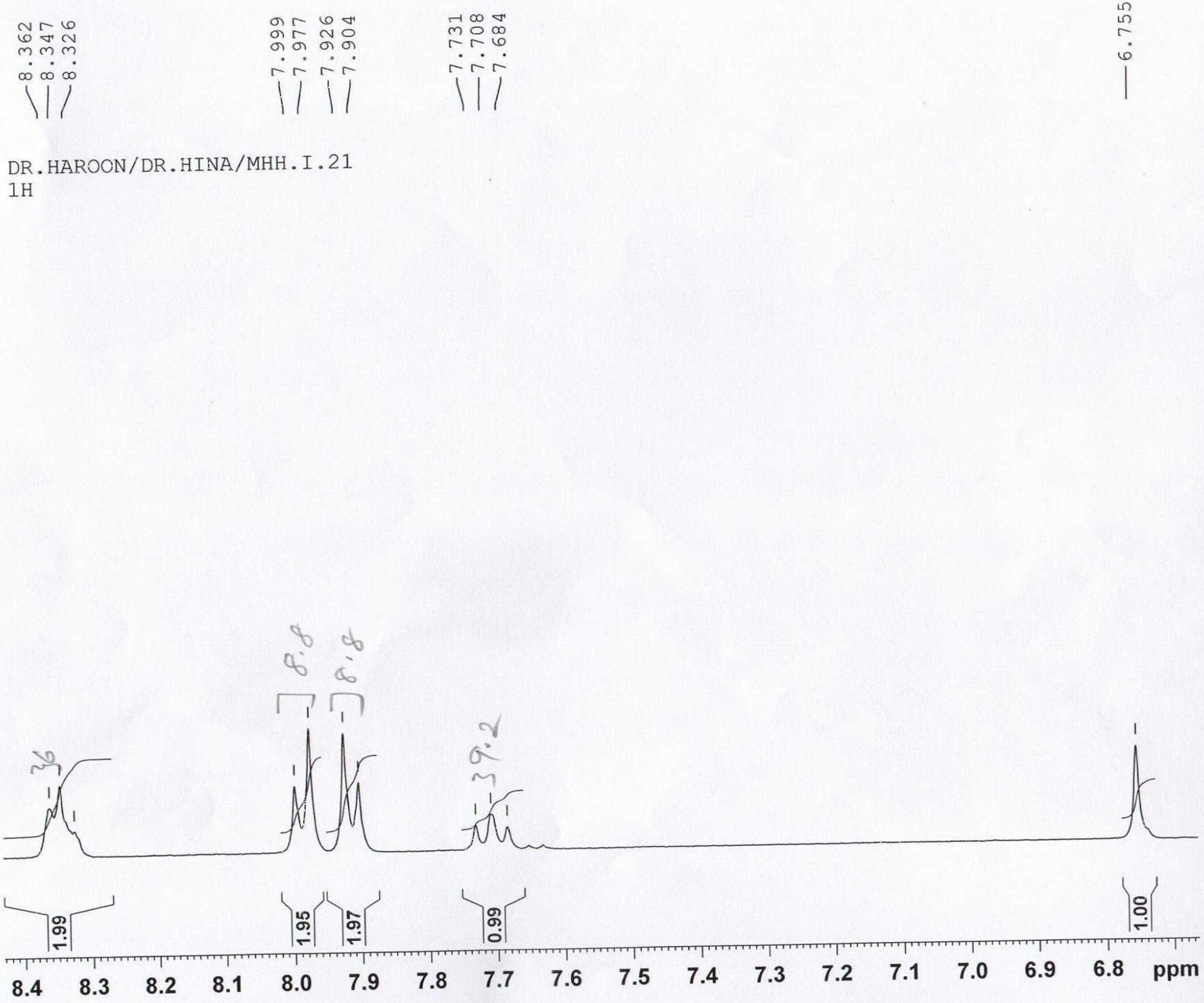
8.362
8.347
8.326
7.999
7.977
7.926
7.904
7.731
7.708
7.684
6.755
DR.HAROON/DR.HINA/MHH.I.21
1H
8.8
8.8
36
39.2
1.99
1.95
1.97
0.99
1.00
8.4 8.3 8.2 8.1 8.0 7.9 7.8 7.7 7.6 7.5 7.4 7.3 7.2 7.1 7.0 6.9 6.8 ppm

Dr. Haroon / Dr. Hina / MHH-I-21/
ICCBS,U.O.K/BB

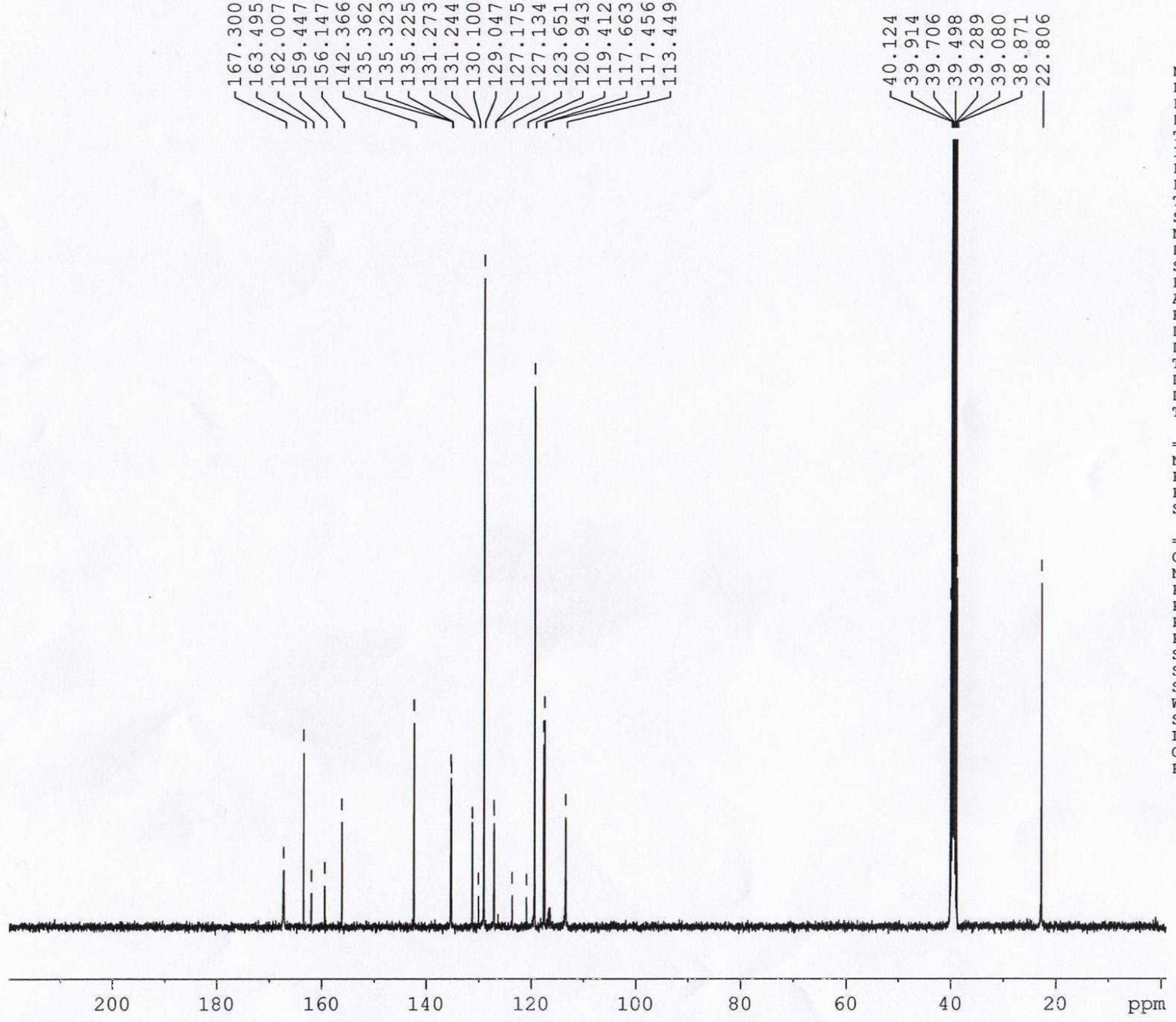

AVANCE 400
LAB NO 117

NAME	may05-17
EXPNO	9
PROCNO	1
Date_	20170505
Time	15.42
INSTRUM	spect
PROBHD	5 mm DUL 13C-1
PULPROG	zgpg
TD	32768
SOLVENT	DMSO
NS	18432
DS	0
SWH	24154.590 Hz
FIDRES	0.737140 Hz
AQ	0.6783476 sec
RG	32768
DW	20.700 usec
DE	6.50 usec
TE	300.0 K
D1	2.00000000 sec
D11	0.03000000 sec
TD0	18

======== CHANNEL f1 ========

NUC1	13C
P1	8.55 usec
PL1	7.00 dB
SFO1	100.6243395 MHz

======== CHANNEL f2 ========

CPDPRG2	waltz16
NUC2	1H
PCPD2	80.00 usec
PL2	0.00 dB
PL12	19.00 dB
PL13	20.00 dB
SFO2	400.1324008 MHz
SI	16384
SF	100.6128205 MHz
WDW	EM
SSB	0
LB	1.00 Hz
GB	0
PC	1.00

Dr. Haroon / Dr. Hina / MHH-I-21/
ICCBS,U.O.K/BB

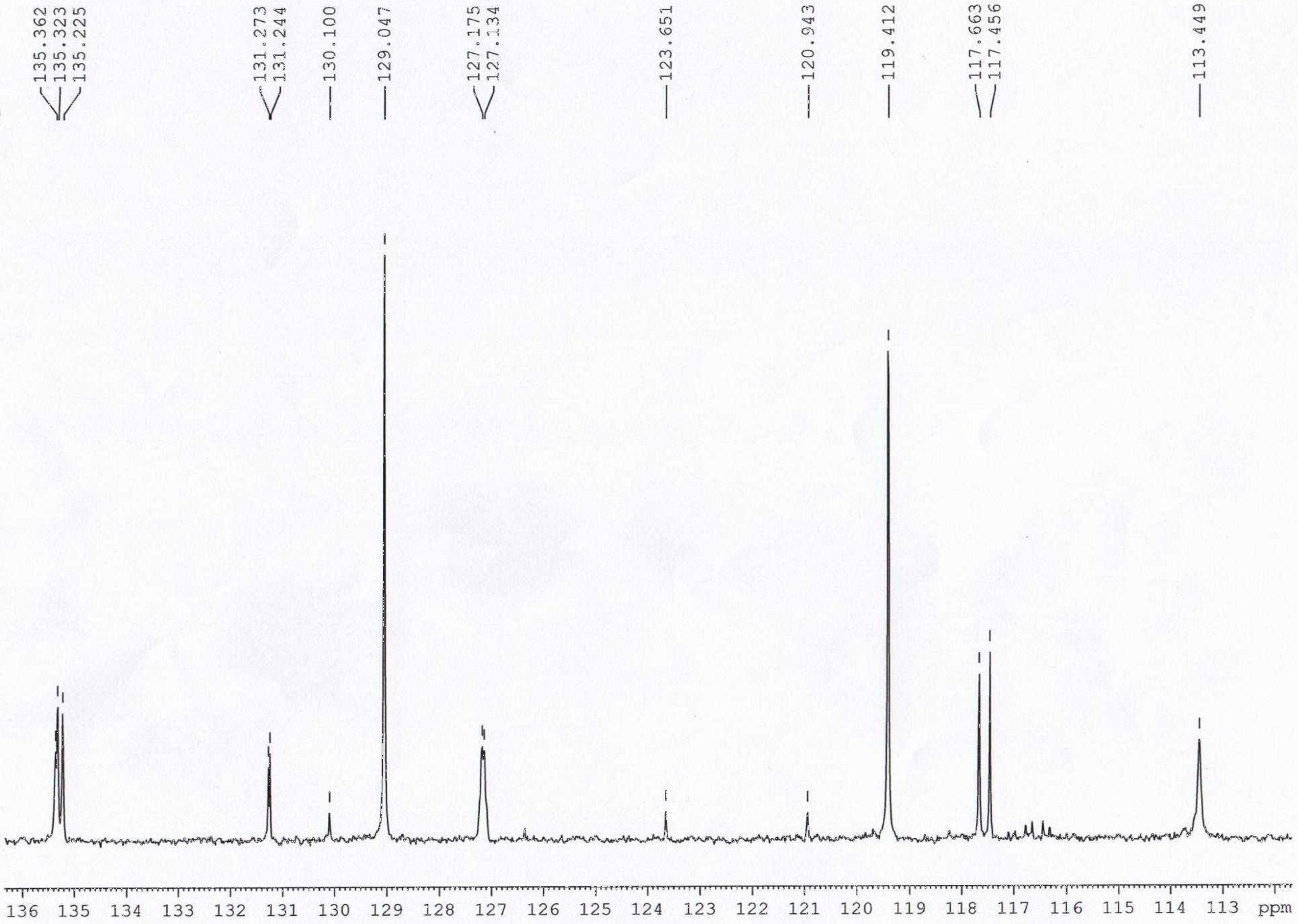

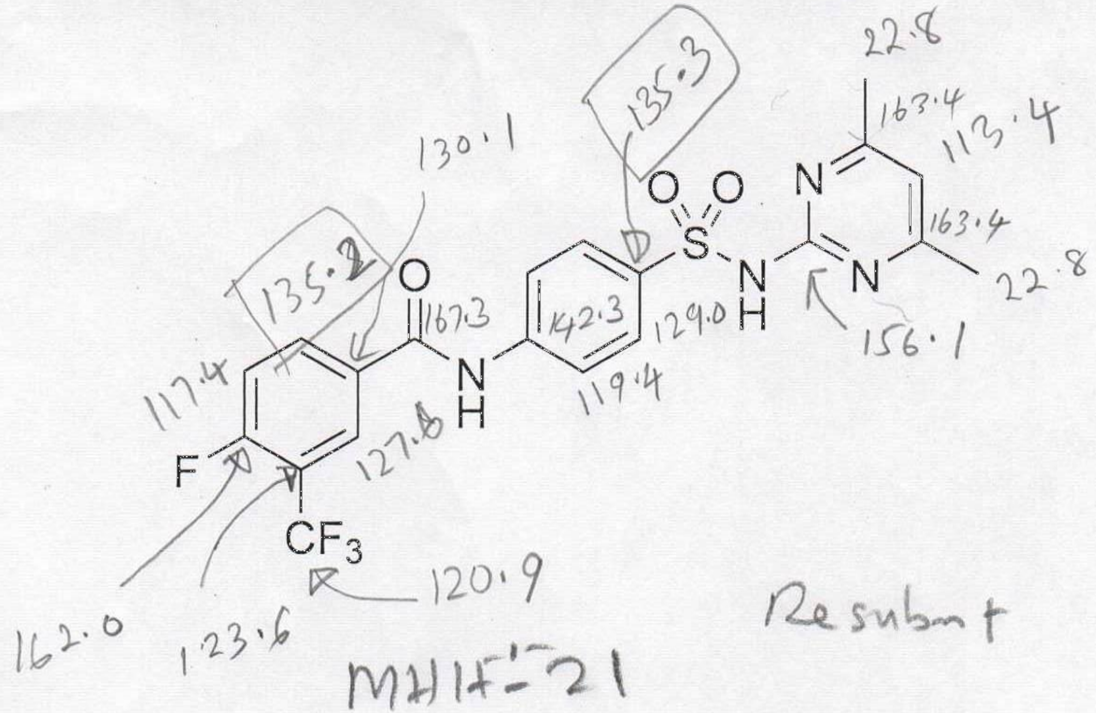

Resubmit

MHK-21

V. good

JEOL HX 110 MASS SPECTROMETER (FAB-HR)

STUDENT NAME	Dr. M. H. Haroon	SAMPLE CODE	DATE	24/5/17
SUPERVISOR NAME	Dr. Hina	MHH-I-21	FAB (+VE / -VE)	FAB +ve

Mass	Theoretical Mass	Delta [ppm]	Delta [mmu]	RDB	Composition
469.0960	469.0959	0.1	0.1	16.0	$C_{25} H_{18} O_3 N_1 F_3 S_1$
	469.0957	0.5	0.3	12.5	→ $C_{20} H_{17} O_3 N_4 F_4 S_1$
	469.0964	-0.9	-0.4	21.5	$C_{28} H_{13} O_1 N_2 F_4$
	469.0953	1.6	0.7	25.5	$C_{31} H_{12} N_2 F_3$
	469.0948	2.5	1.2	20.0	$C_{28} H_{17} O_2 N_1 F_2 S_1$
	469.0946	3.0	1.4	16.5	$C_{23} H_{16} O_2 N_4 F_3 S_1$

HEJ-ICCBS

2/15/2017 9:06:15 AM

File: MHH-I-21 **Date Run: 02-14-2017 (Time Run: 16:10:10)**

Sample: DR.M.H.HAROON /DR. HINA

Instrument: JEOL MS 600H-1

Ionization mode: EI+

Scan: 12-15 **R.T.: 1.11**

Base: m/z 403; 66.1%FS TIC: 4960137

Threshold: 1% of Base **Displayed TIC: 4960137**

Mass	%Base	%TIC	Mass	%Base	%TIC	Mass	%Base	%TIC	Mass	%Base	%TIC	Mass	%Base	%TIC
42.0	2.2	.301	90.0	1.0	.146	144.1	4.7	.656	196.2	1.3	.186	283.1	1.8	.256
44.0	1.6	.227	91.0	2.5	.349	145.1	1.5	.203	197.2	1.3	.175	315.2	2.0	.284
53.0	1.0	.143	93.0	2.5	.349	157.1	1.2	.162	198.2	2.5	.355	335.3	3.1	.440
54.0	1.8	.257	94.0	1.3	.185	163.1	53.8	7.525	212.2	4.2	.591	336.2	3.0	.413
63.0	2.1	.296	95.1	2.0	.278	164.1	4.2	.587	213.2	88.5	12.373	385.2	1.0	.145
64.1	2.0	.274	96.1	2.5	.344	171.1	1.3	.186	214.2	25.8	3.605	403.2	100.0	13.983
65.1	3.2	.443	107.1	3.1	.439	172.2	1.3	.177	215.2	5.9	.829	404.2	96.9	13.549
66.1	1.7	.240	108.1	1.2	.167	185.1	2.4	.329	239.2	1.2	.164	405.3	54.0	7.547
67.1	2.3	.323	113.0	2.0	.286	186.2	2.2	.302	241.2	1.8	.250	406.2	6.8	.945
75.0	1.3	.176	123.1	6.0	.833	191.1	83.4	11.667	262.1	1.2	.173	429.3	5.7	.790
81.1	1.2	.169	143.1	3.3	.462	192.1	11.2	1.566	282.1	7.0	.986	430.2	1.3	.176

I. C.C.B.S., University of Karachi Analytical Laboratory - Pakistan

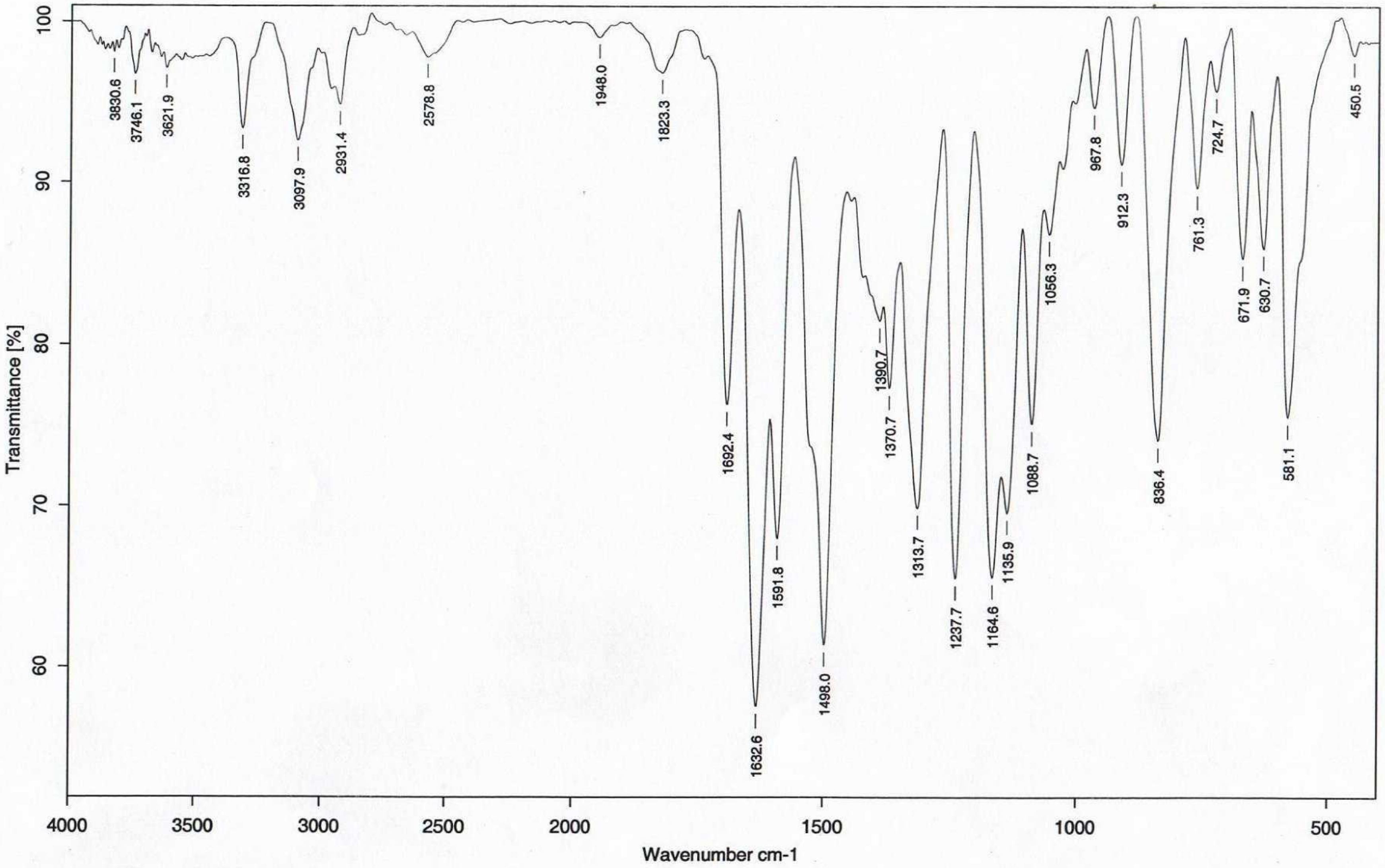

Sample : MHH-1-21/Haroon/Dr. Hina	Spectrum : MHH-1-21.0 (in D:\IRSTUDENT)
Measured : 01/02/2017 on VECTOR22	Technic : Solid
Resolution : 4 cm-1 (10 scans)	Analyst : ZA/Jamshed/M. Asif/Haroon

THERMO ELECTRON ~ VISIONpro SOFTWARE V4.10

Operator Name	ARSHAD ALAM.	Date of Report	2/2/2017
Department	Analytical Laboratory TWC # 004	Time of Report	2:36:29PM
Organization	ICCBS Karachi of Universty.		
Information	Dr.Haroon/ Dr.Hina		

Scan Graph

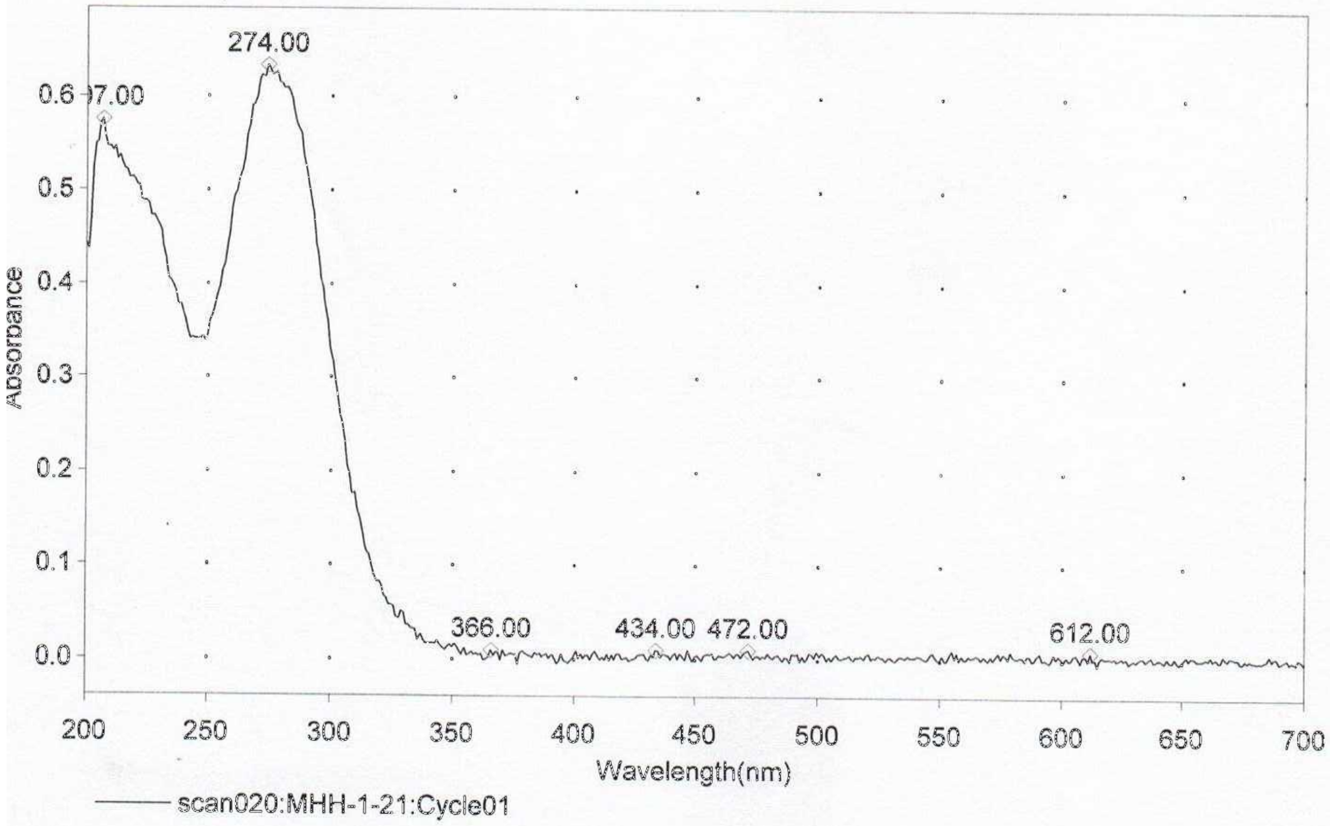

Results Table - MHH-1-21.sre,MHH-1-21,Cycle01

nm	A	Peak Pick Method	
207.00	0.575	Find 8 Peaks Above -3.0000 A	
274.00	0.634	Start Wavelength 200.00 nm	
366.00	0.009	Stop Wavelength 700.00 nm	
434.00	0.010	Sort By Wavelength	
472.00	0.010	Sensitivity	Manual
612.00	0.009	Rising Points	3
Falling Points	3		
Min. Change	0.0000		
