# Supplementary material for: Sulphamethazine derivatives as immunomodulating agents: New therapeutic strategies for inflammatory diseases
Source: PLoS One. 2018 Dec 19;13(12):e0208933. doi: 10.1371/journal.pone.0208933 (PMC6300282; doi:10.1371/journal.pone.0208933)
Supplement: S9 Fig — (PDF) [file pone.0208933.s009.pdf]

AVANCE AV-400 MHz
Lab # 115

DR.HAROON/DR.HINA/MHH.I.47
1H

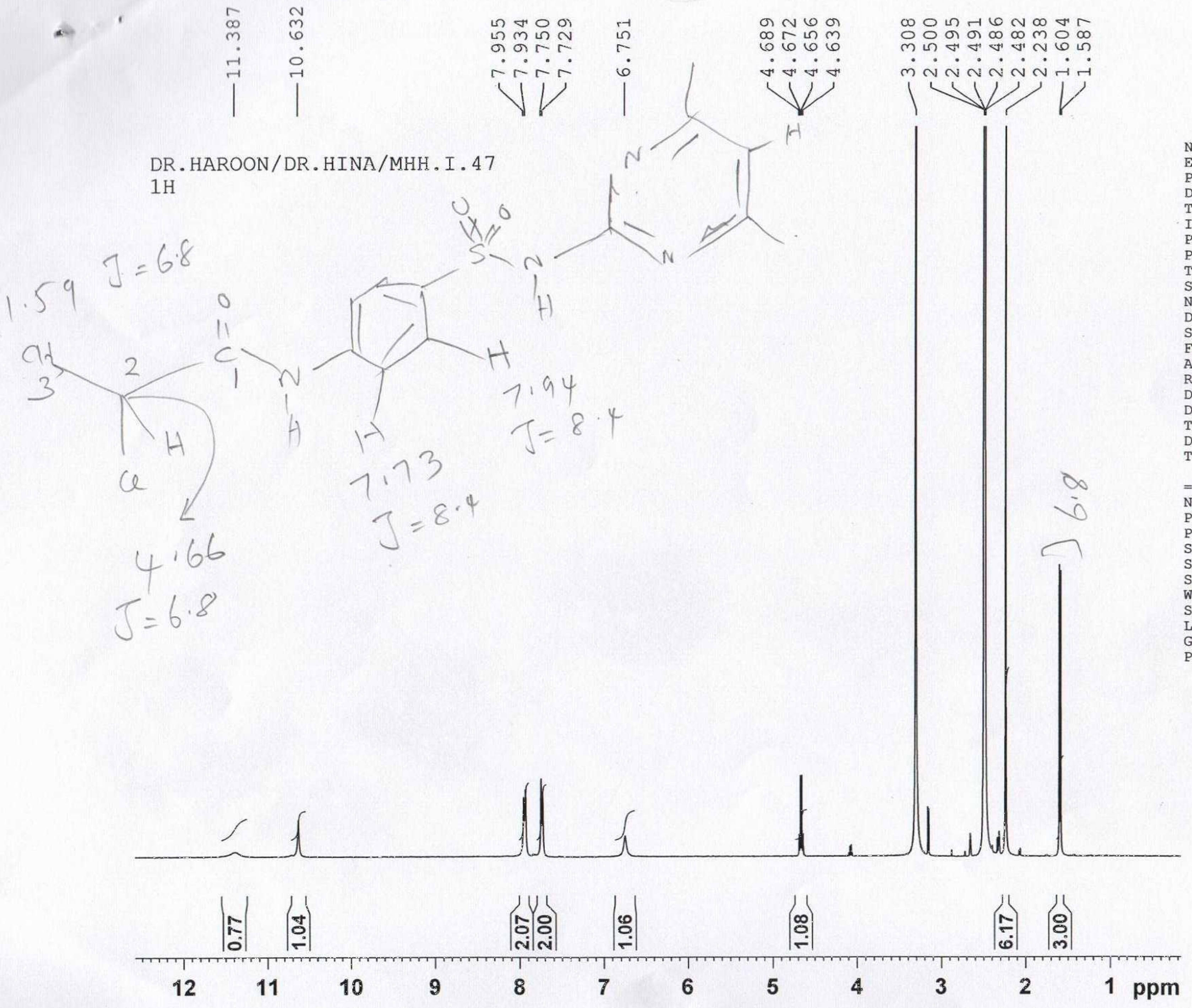

NAME	feb24-17
EXPNO	6
PROCNO	1
Date_	20170224
Time	14.39
INSTRUM	spect
PROBHD	5 mm SEI 1H-13
PULPROG	zg30
TD	65536
SOLVENT	DMSO
NS	64
DS	0
SWH	8012.820 Hz
FIDRES	0.122266 Hz
AQ	4.0894966 sec
RG	574.7
DW	62.400 usec
DE	6.50 usec
TE	300.0 K
D1	2.00000000 sec
TD0	1

======== CHANNEL f1 ========

NUC1	1H
P1	10.63 usec
PL1	2.00 dB
SFO1	400.0332002 MHz
SI	32768
SF	400.0300041 MHz
WDW	EM
SSB	0
LB	0.30 Hz
GB	0
PC	1.00

DR.HAROON/DR.HINA/MHH.I.47
1H

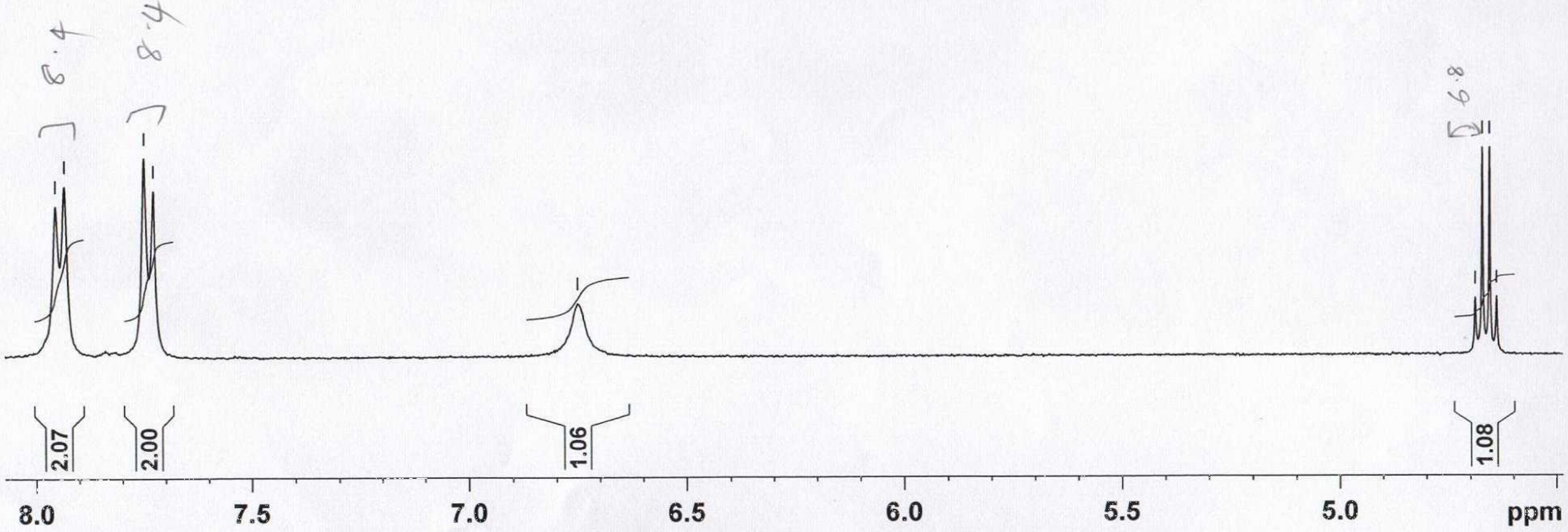

File: MHH-I-47-FABp
Sample: DR. M.H.HAROON /DR. HINA
Instrument: JEOL-600H-2
Inlet: Direct Probe

Date Run: 04-01-2017 (Time Run: 10:56:50)

Ionization mode: FAB+

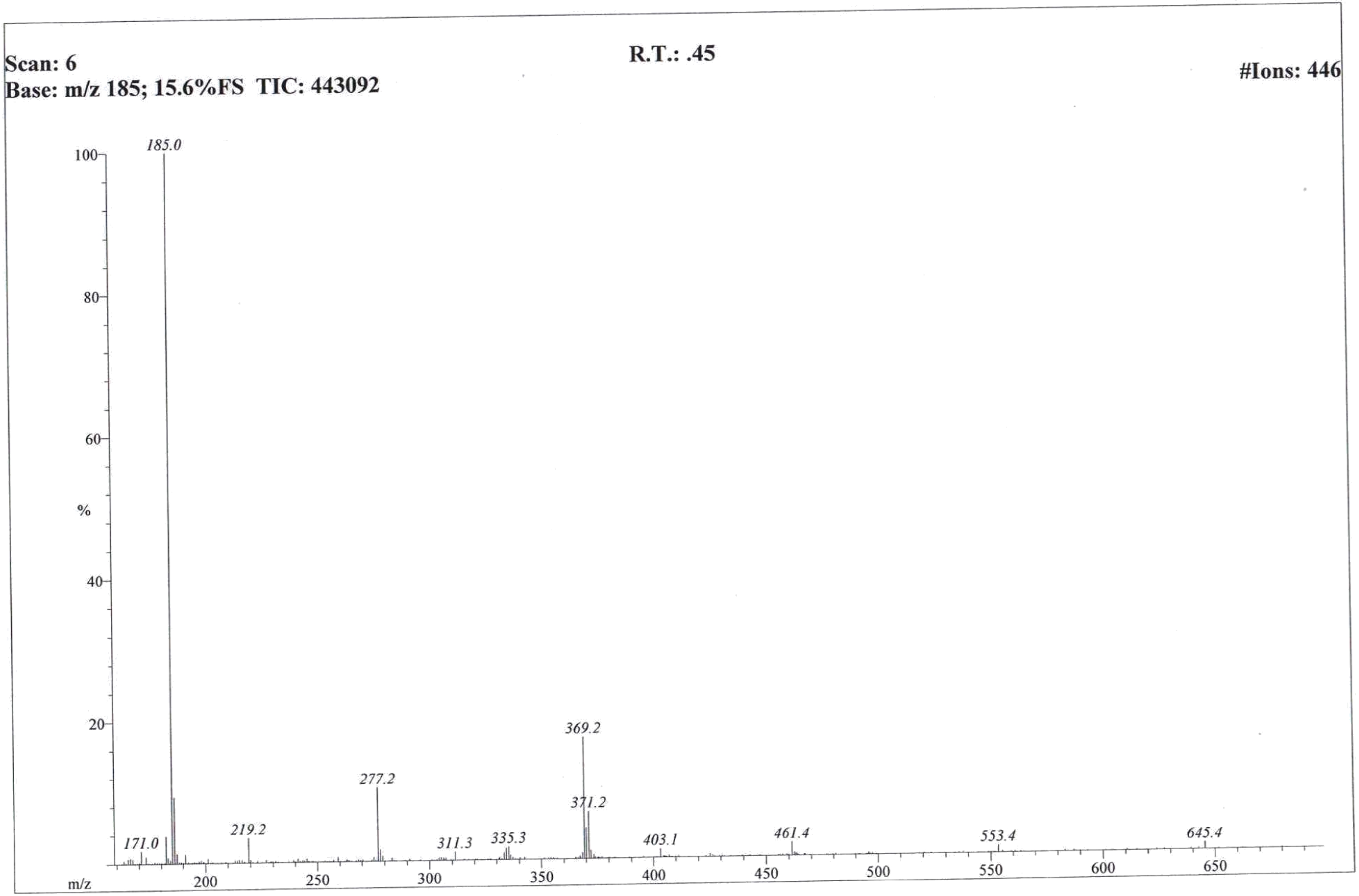

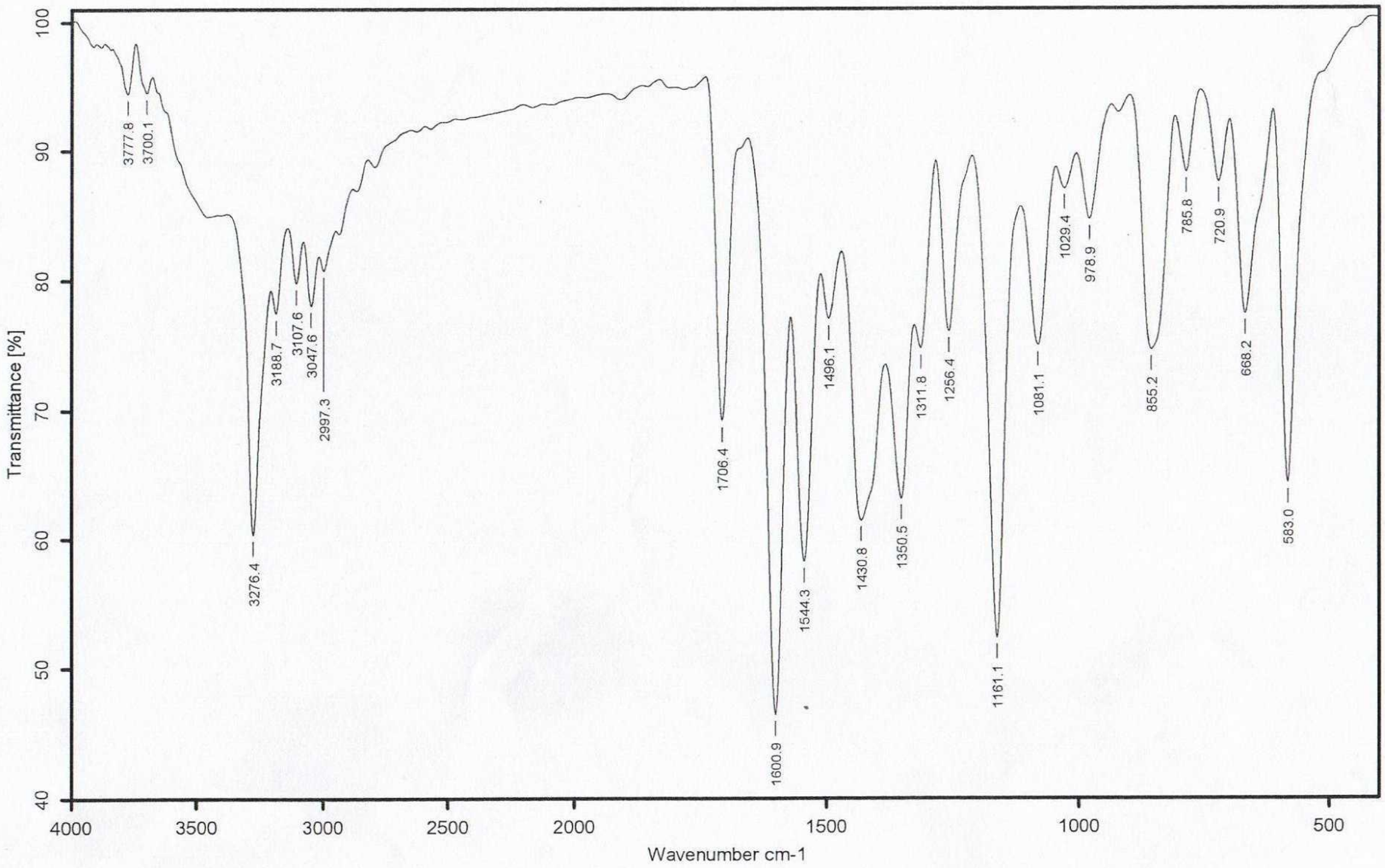

Sample : MHH-I-47/Dr. Haroon/Dr. Hina

Measured : 05/07/2017 on VECTOR22

Resolution : 4 cm-1 (10 scans)

Spectrum : MHH-I-47.0 (in D:\IRSTUDENT)

Technic : Solid

Analyst : MA/ZA

THERMO ELECTRON ~ VISIONpro SOFTWARE V4.10

)perator Name	ARSHAD ALAM	Date of Report	7/5/2017
)epartment	Analytical Laboratory TWC # 004	Time of Report	11:15:31AM
)rganization	ICCBS Karachi of Universty.		
nformation	Dr. Haroon /Dr. Hina		

;can Graph

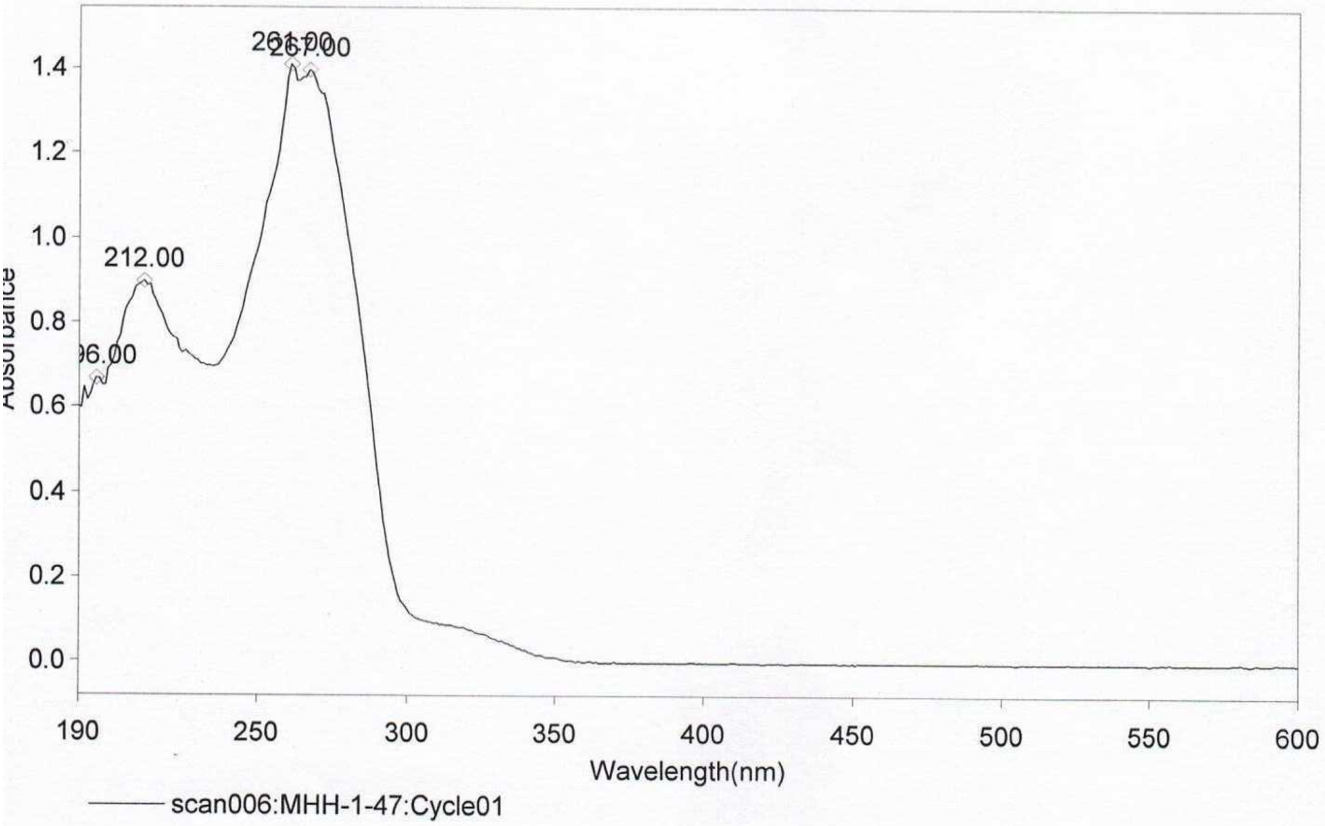

:esults Table - MHH-1-47.sre,MHH-1-47,Cycle01

m	A	Peak Pick Method
96.00	0.665	Find 4 Peaks Above -3.0000 A
12.00	0.898	Start Wavelength 190.00 nm
61.00	1.410	Stop Wavelength 600.00 nm
67.00	1.397	Sort By Wavelength
ensitivity	Medium	
