# Supplementary material for: Sulphamethazine derivatives as immunomodulating agents: New therapeutic strategies for inflammatory diseases
Source: PLoS One. 2018 Dec 19;13(12):e0208933. doi: 10.1371/journal.pone.0208933 (PMC6300282; doi:10.1371/journal.pone.0208933)
Supplement: S10 Fig — (PDF) [file pone.0208933.s010.pdf]

M. Haron / Dr. Hina / MHH-I-19
1H

$J_{2,(3,4f)} / J_{6(5,4f)} =$

AVANCE AV - III
300 MHz, LAB # 116

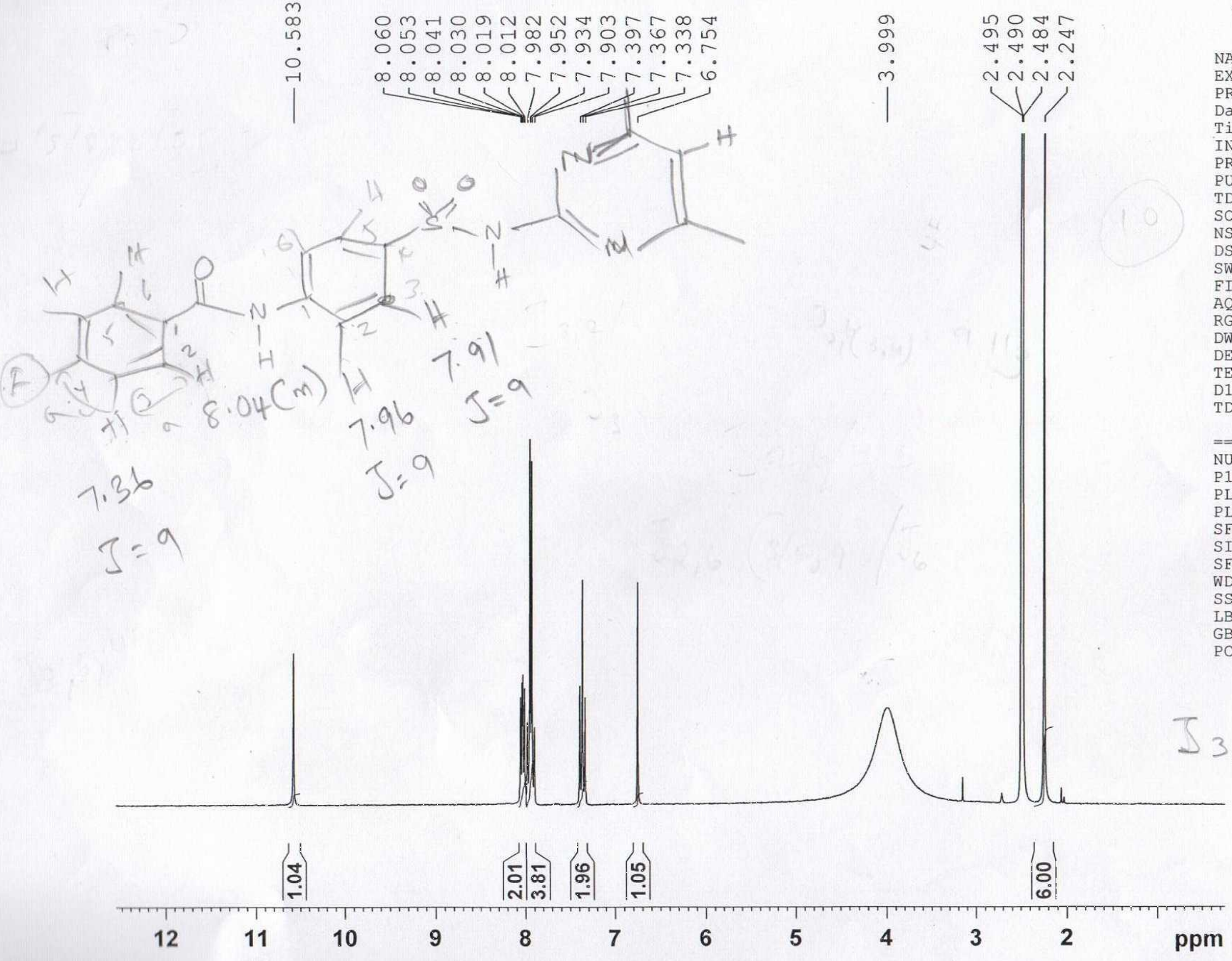

NAME	Dec29-16
EXPNO	7
PROCNO	1
Date_	20161229
Time	14.51
INSTRUM	Spect
PROBHD	5 mm BBO BB-1H
PULPROG	zg30
TD	32768
SOLVENT	DMSO
NS	32
DS	0
SWH	6009.615 Hz
FIDRES	0.183399 Hz
AQ	2.7263477 sec
RG	203
DW	83.200 usec
DE	6.50 usec
TE	300.0 K
D1	1.50000000 sec
TD0	1

======== CHANNEL f1 ========

NUC1	1H
P1	12.50 usec
PL1	0.00 dB
PL1W	13.16228485 W
SFO1	300.1324010 MHz
SI	16384
SF	300.1300041 MHz
WDW	EM
SSB	0
LB	0.30 Hz
GB	0
PC	1.00

(12)

$J_{3,(2,4f)} / J_{5,(6,4f)} =$

M. Haron / Dr. Hina / MHH-I-19
1H

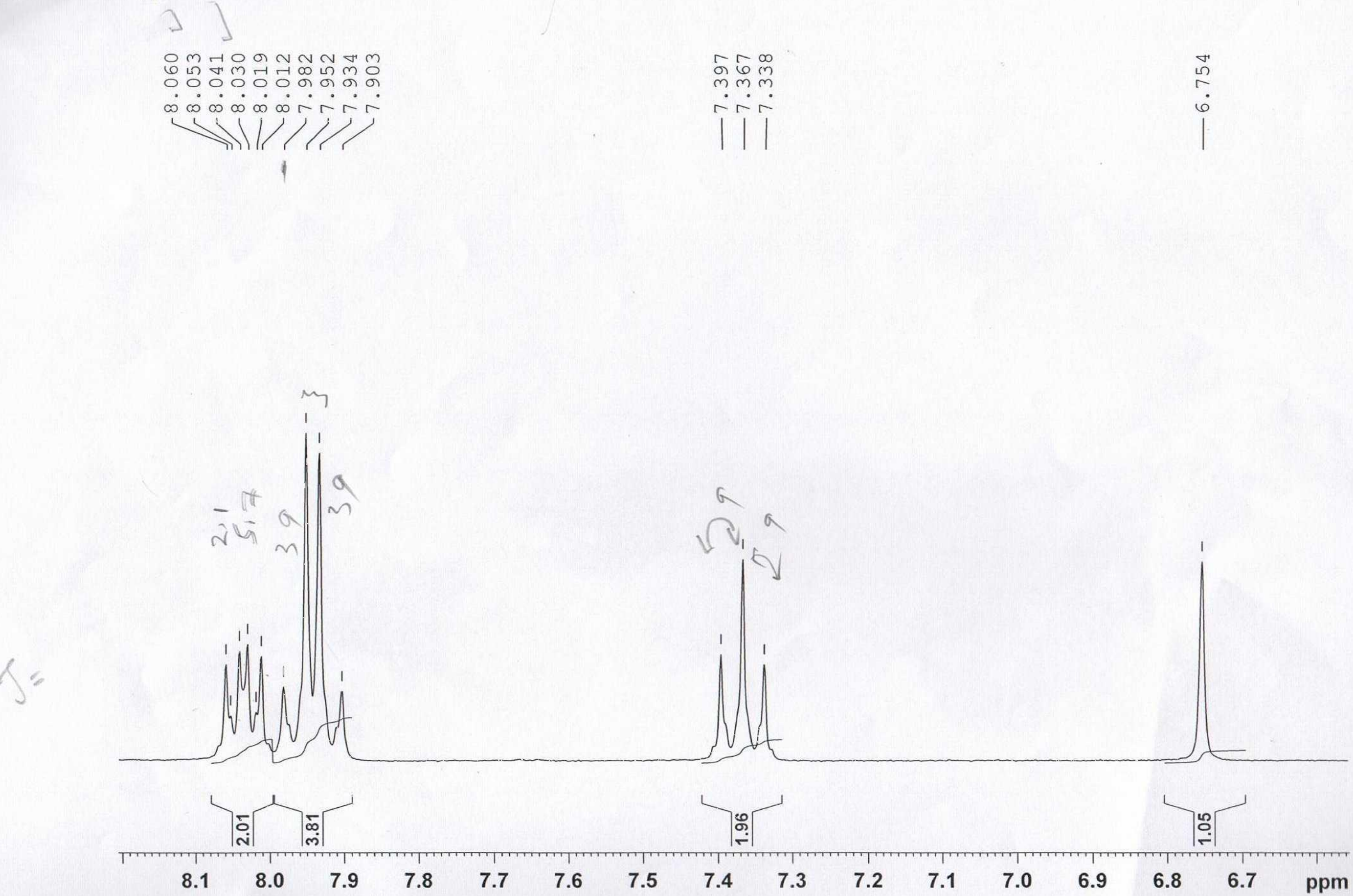

HEJ-ICCBS
2/14/2017 4:09:04 PM

File: MHH-I-19 **Date Run: 02-14-2017 (Time Run: 15:59:30)**
Sample: DR.M.H.HAROON /DR. HINA
Instrument: JEOL MS 600H-1
Ionization mode: EI+

Scan: 22-24 **R.T.: 1.95**
Base: m/z 123; 79.7%FS TIC: 5837445

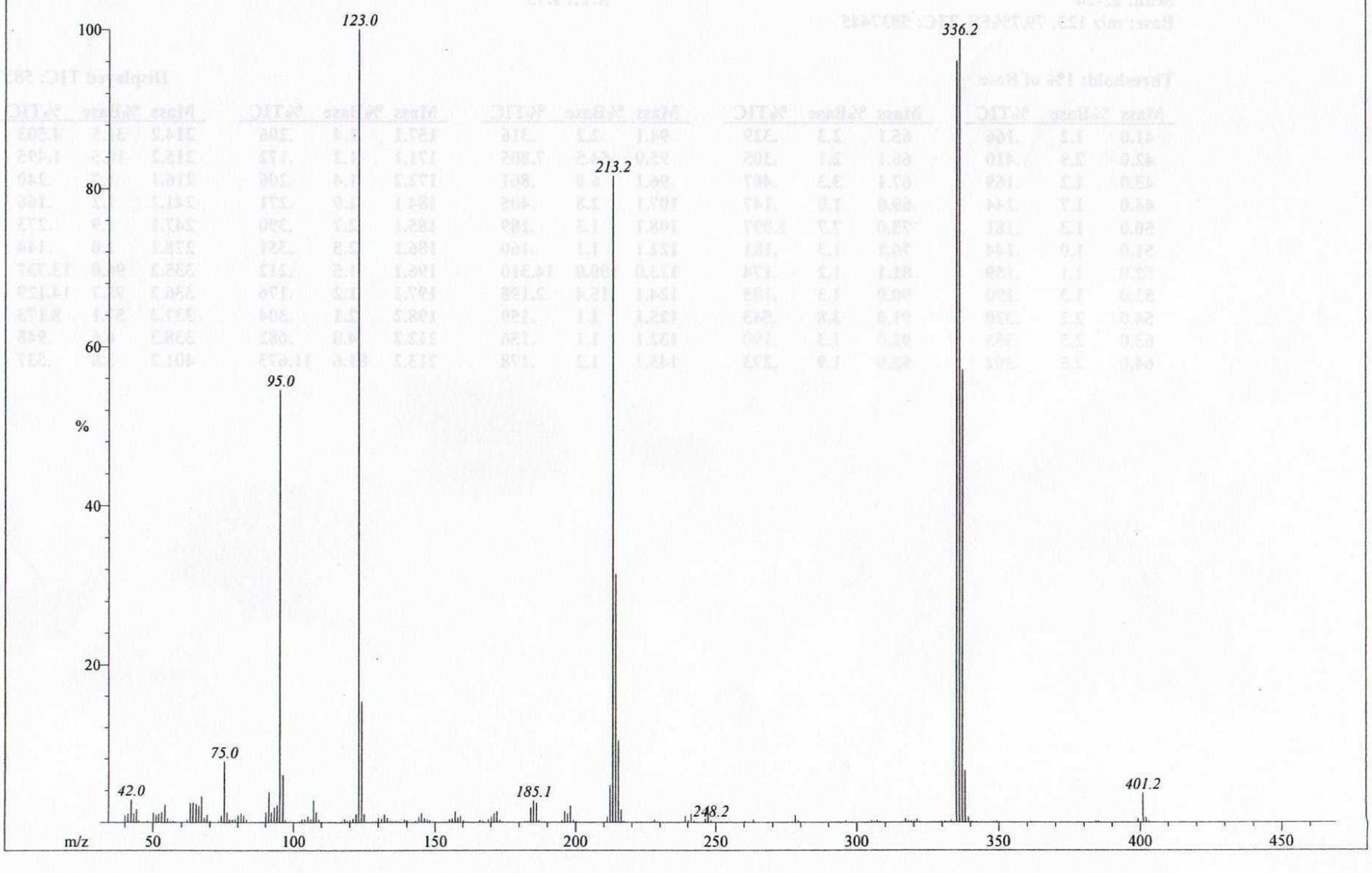

I. C.C.B.S., University of Karachi Analytical Laboratory - Pakistan

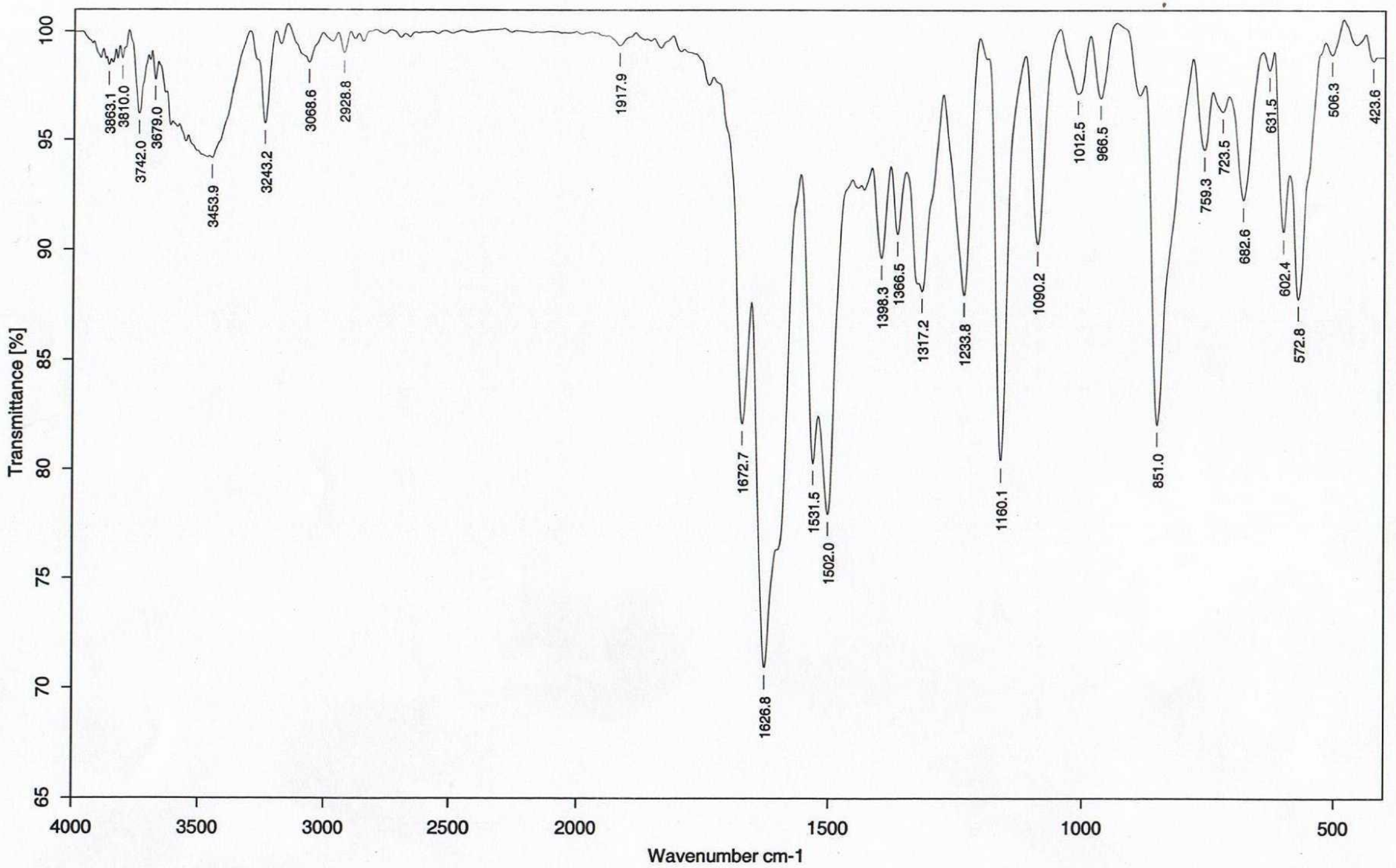

Sample : MHH-1-19/Haroon/Dr. Hina

Measured : 01/02/2017 on VECTOR22

Resolution : 4 cm-1 (10 scans)

Spectrum : MHH-1-19.0 (in D:\IRSTUDENT)

Technic : Solid

Analyst : ZA/Jamshed/M. Asif/Haroon

THERMO ELECTRON ~ VISIONpro SOFTWARE V4.10

)perator Name	ARSHAD ALAM.	Date of Report	2/2/2017
)epartment	Analytical Laboratory TWC # 004	Time of Report	2:59:48PM
)rganization	ICCBS Karachi of Universty.		
nformation	Dr.Haroon/ Dr.Hina		

;can Graph

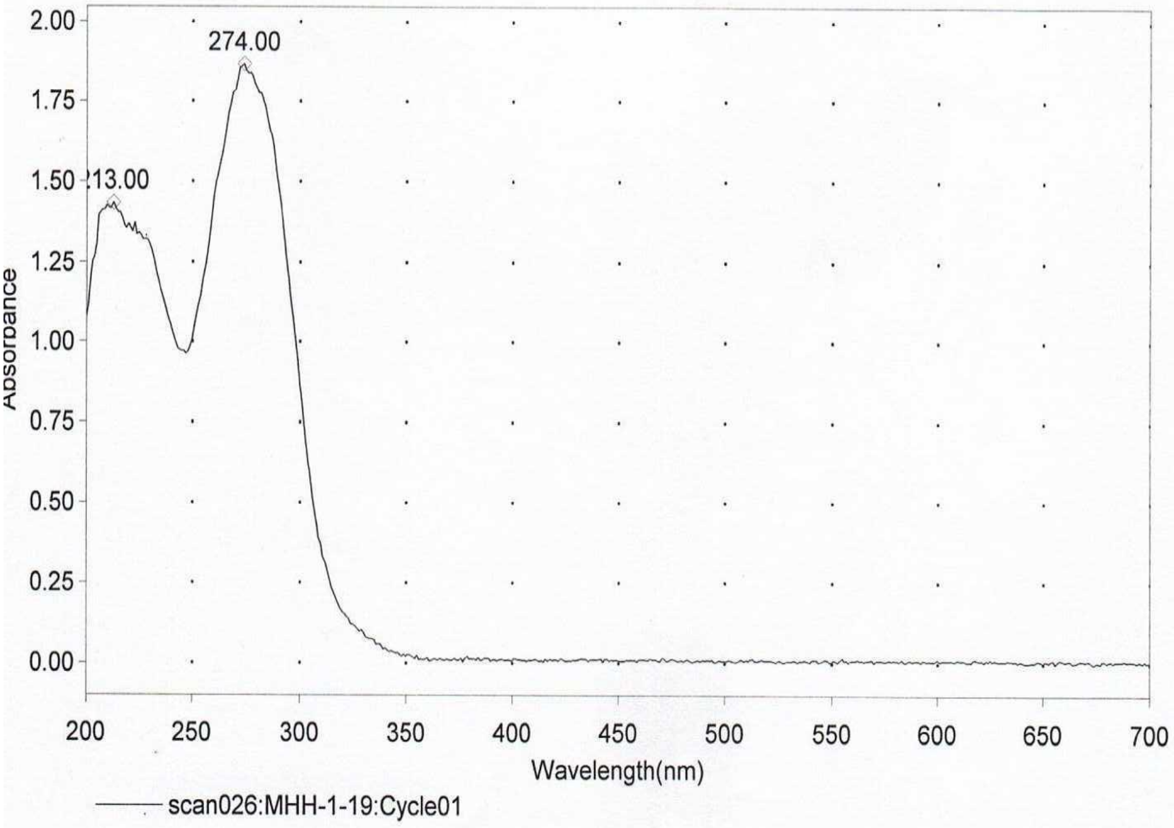

₹esults Table - MHH-1-19.sre,MHH-1-19,Cycle01

m	A	Peak Pick Method
13.00	1.433	Find 8 Peaks Above -3.0000 A
74.00	1.866	Start Wavelength 200.00 nm
		Stop Wavelength 700.00 nm
		Sort By Wavelength

;ensitivity Auto
