# Supplementary material for: Sulphamethazine derivatives as immunomodulating agents: New therapeutic strategies for inflammatory diseases
Source: PLoS One. 2018 Dec 19;13(12):e0208933. doi: 10.1371/journal.pone.0208933 (PMC6300282; doi:10.1371/journal.pone.0208933)
Supplement: S11 Fig — (PDF) [file pone.0208933.s011.pdf]

AVANCE AV-400 MHz
Lab # 115

DR.HAROON/DR.HINA/MHH.I.7
1H

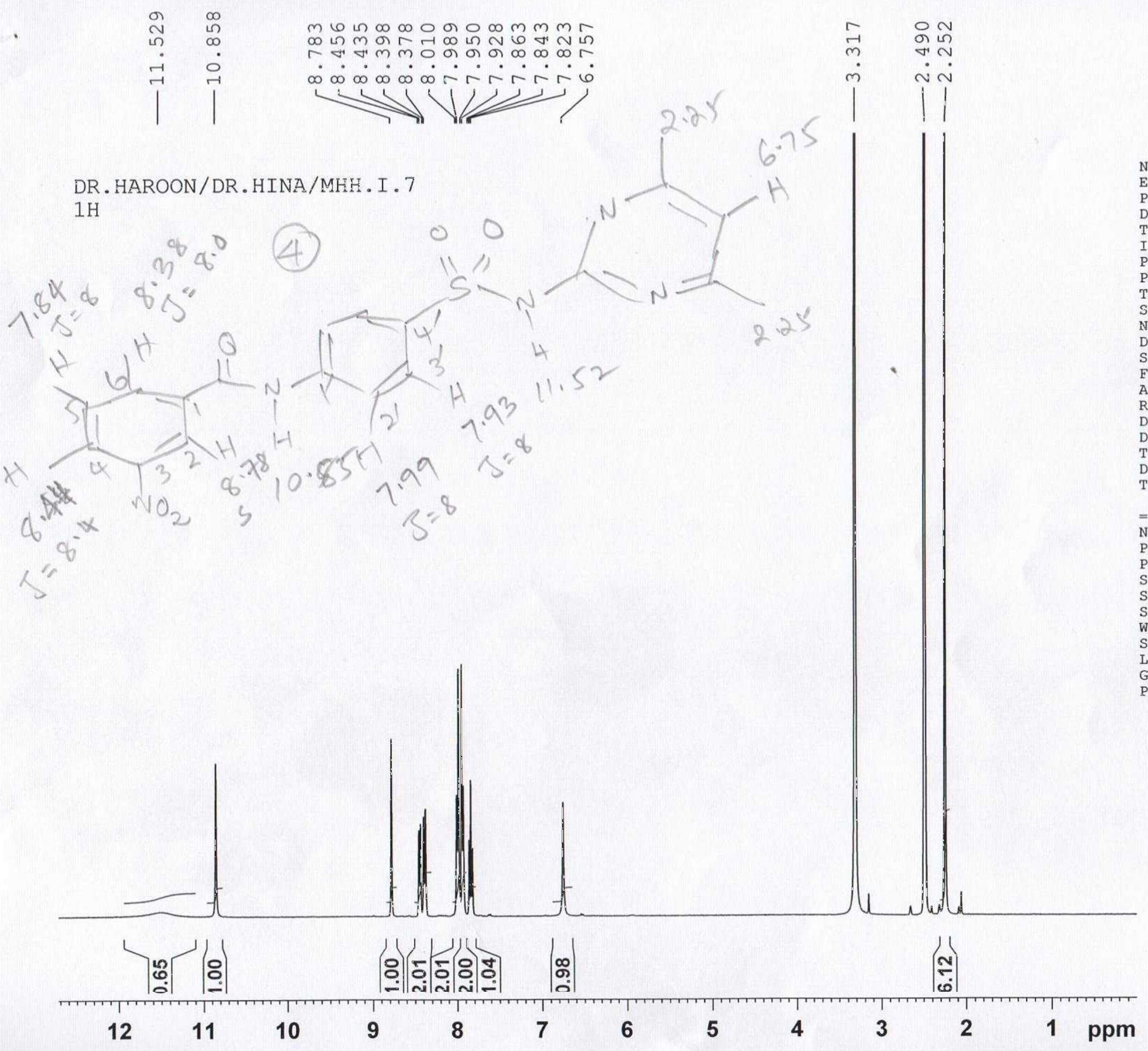

NAME	dec28-16
EXPNO	9
PROCNO	1
Date_	20161228
Time	13.25
INSTRUM	spect
PROBHD	5 mm SEI 1H-13
PULPROG	zg30
TD	65536
SOLVENT	DMSO
NS	64
DS	0
SWH	8012.820 Hz
FIDRES	0.122266 Hz
AQ	4.0894966 sec
RG	362
DW	62.400 usec
DE	6.50 usec
TE	300.0 K
D1	2.00000000 sec
TD0	1

======== CHANNEL f1 ========

NUC1	1H
P1	10.80 usec
PL1	3.00 dB
SFO1	400.0332002 MHz
SI	32768
SF	400.0300041 MHz
WDW	EM
SSB	0
LB	0.30 Hz
GB	0
PC	1.00

1B

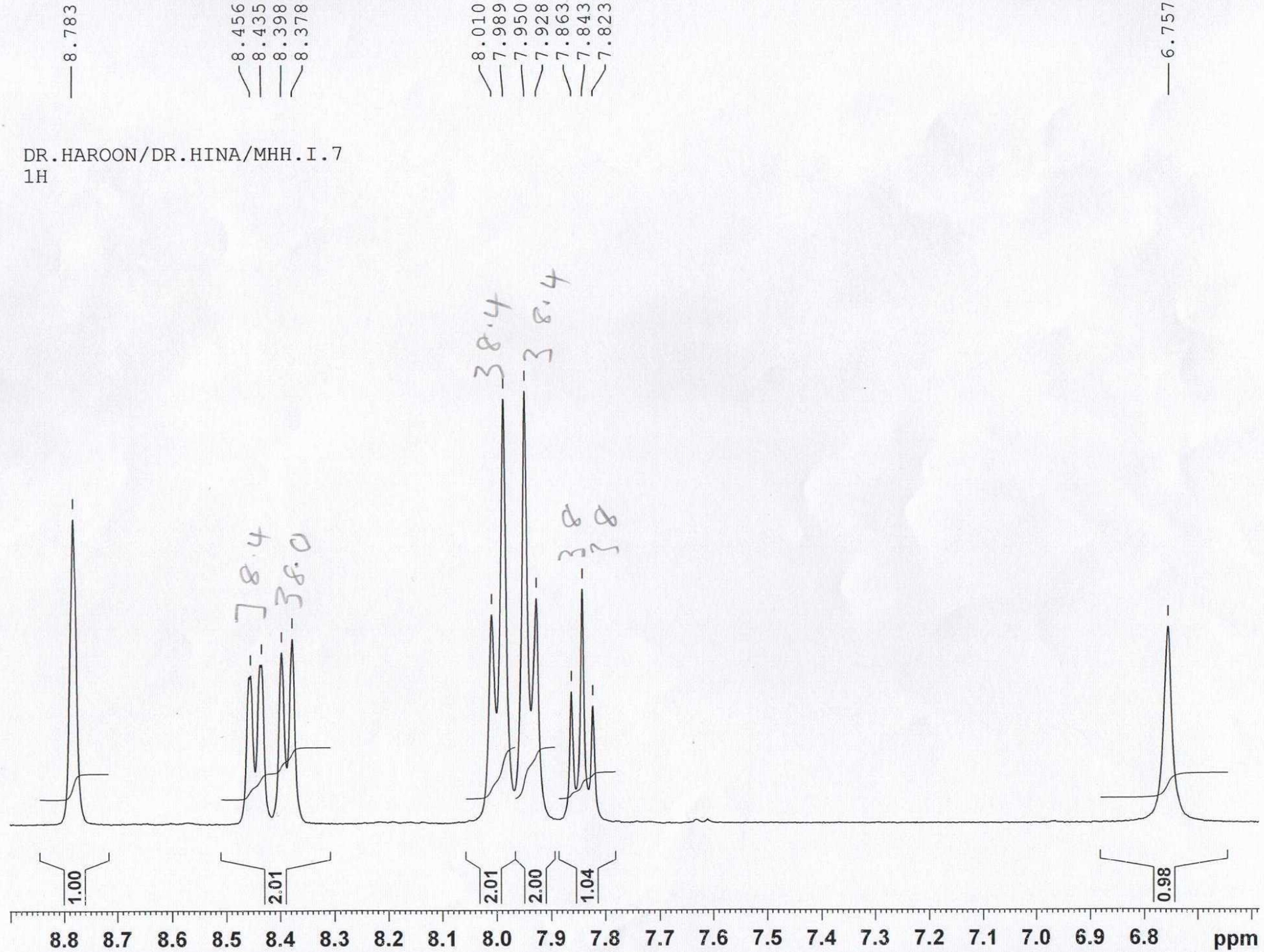
8.783
8.456
8.435
8.398
8.378
8.010
7.989
7.950
7.928
7.863
7.843
7.823
6.757
DR.HAROON/DR.HINA/MHH.I.7
1H
1.00
2.01
2.01
2.00
1.04
0.98
8.8 8.7 8.6 8.5 8.4 8.3 8.2 8.1 8.0 7.9 7.8 7.7 7.6 7.5 7.4 7.3 7.2 7.1 7.0 6.9 6.8 ppm

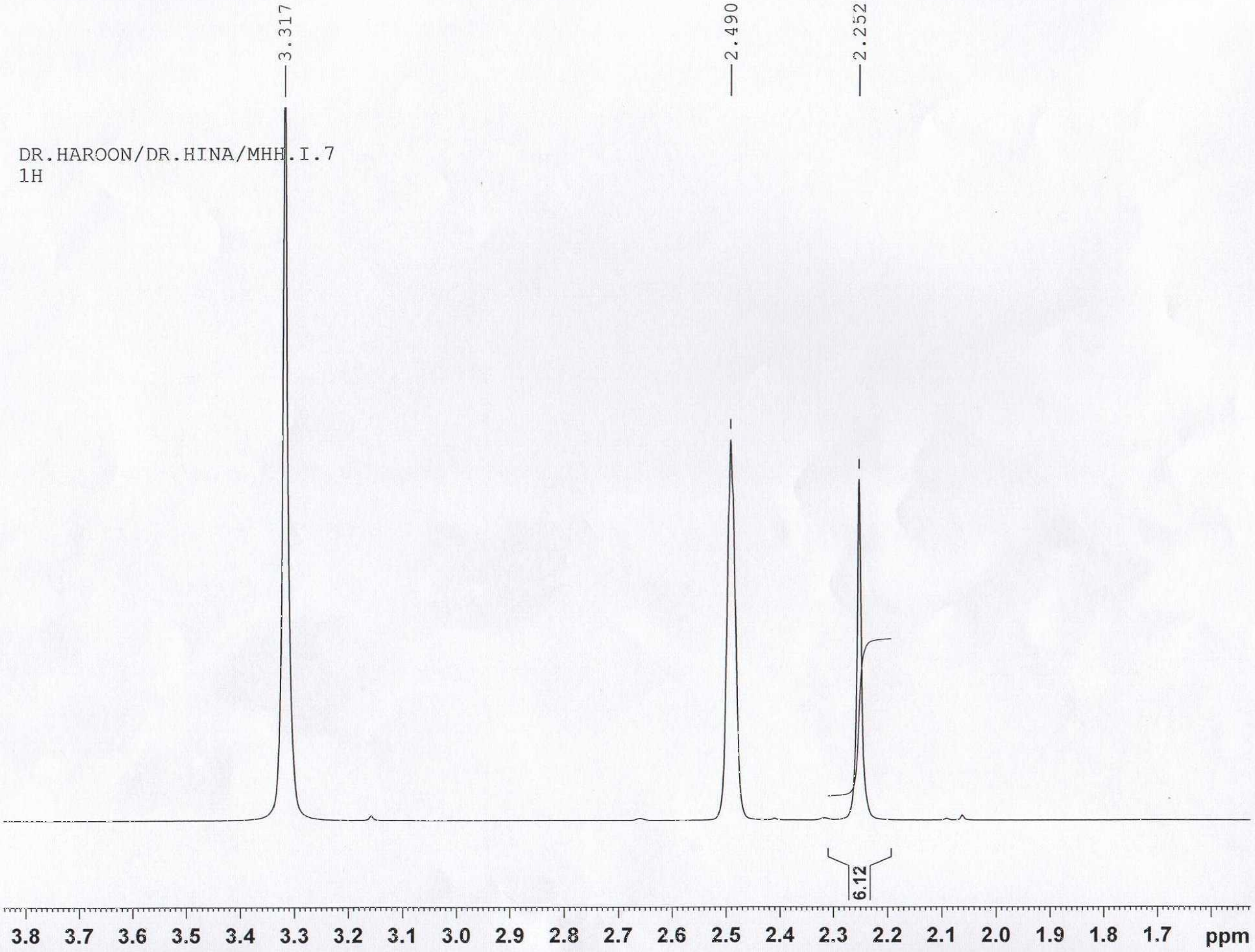
DR.HAROON/DR.HINA/MHH.I.7
1H
3.317
2.490
2.252
6.12
3.8 3.7 3.6 3.5 3.4 3.3 3.2 3.1 3.0 2.9 2.8 2.7 2.6 2.5 2.4 2.3 2.2 2.1 2.0 1.9 1.8 1.7
ppm

File: MHH-I-7-FABP
Sample: DR.M.H.HAROON /DR. HINA
Instrument: JEOL-600H-2
Inlet: Direct Probe

Date Run: 12-09-2016 (Time Run: 08:23:53)

Ionization mode: FAB+

Scan: 4 **R.T.: .27**
Base: m/z 185; 64.5%FS TIC: 995314 **#Ions: 31**

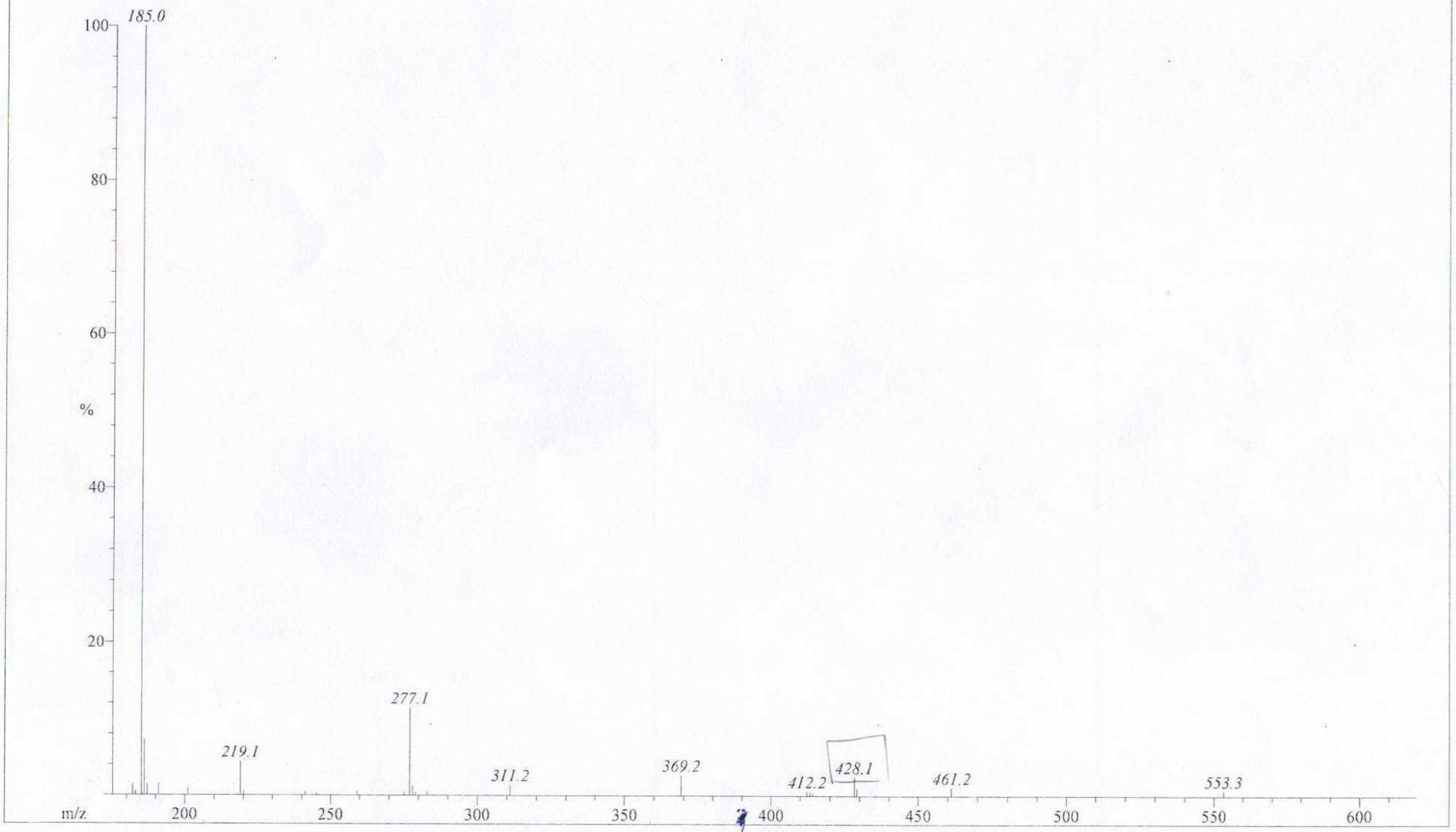

I. C.C.B.S., University of Karachi Analytical Laboratory - Pakistan

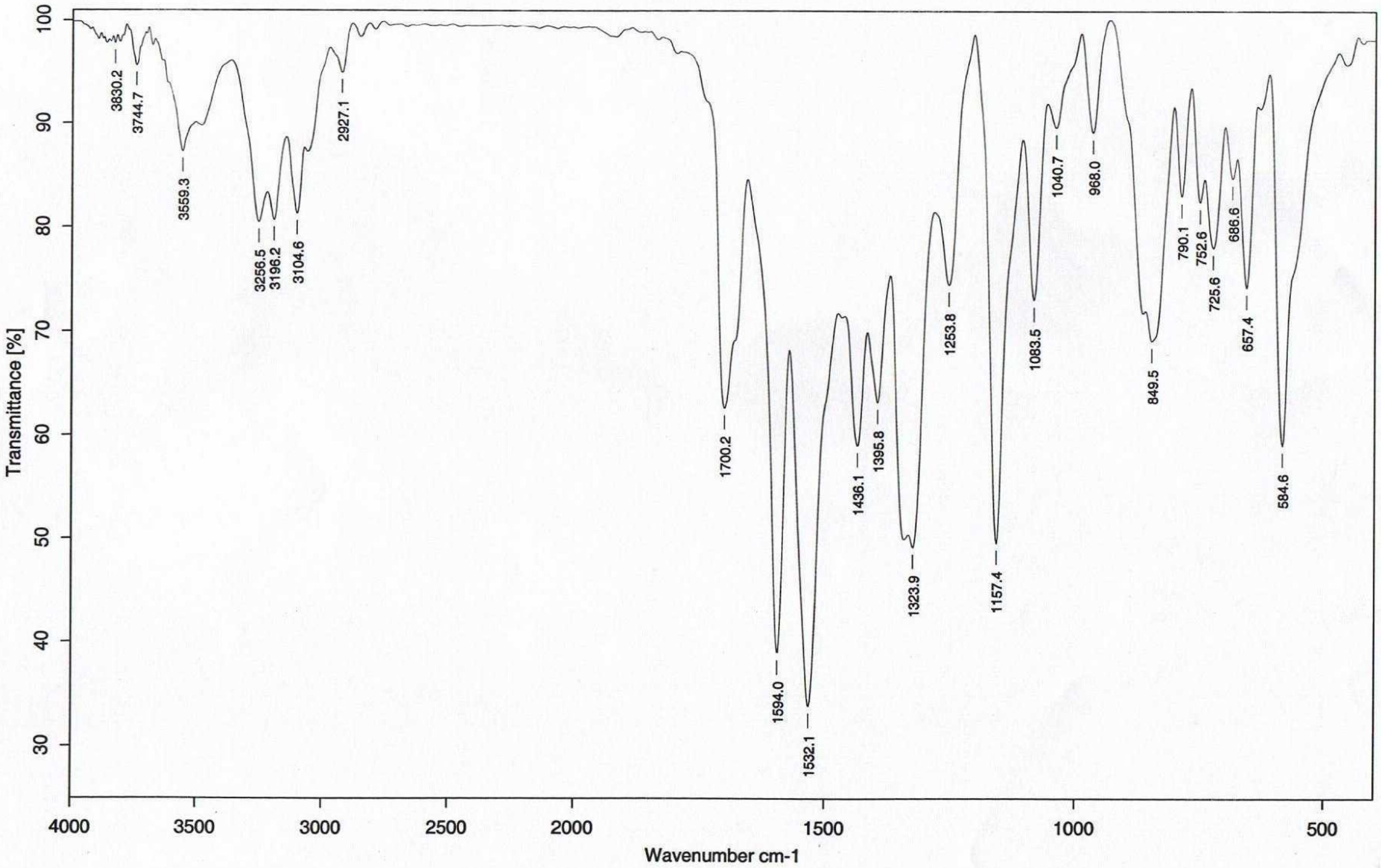

Sample : MHH-1-7/Haroon/Dr. Hina	Spectrum : MHH-1-14.0 (in D:\IRSTUDENT)
Measured : 31/01/2017 on VECTOR22	Technic : Solid
Resolution : 4 cm-1 (10 scans)	Analyst : Zubair Ahmad/ Jamshed/M. Asif/

THERMO ELECTRON ~ VISIONpro SOFTWARE V4.10

Operator Name	ARSHAD ALAM.	Date of Report	2/1/2017
Department	Analytical Laboratory TWC # 004	Time of Report	3:07:49PM
Organization	ICCBS Karachi of Universty.		
Information	Dr Haroon/Dr Hina		

Scan Graph

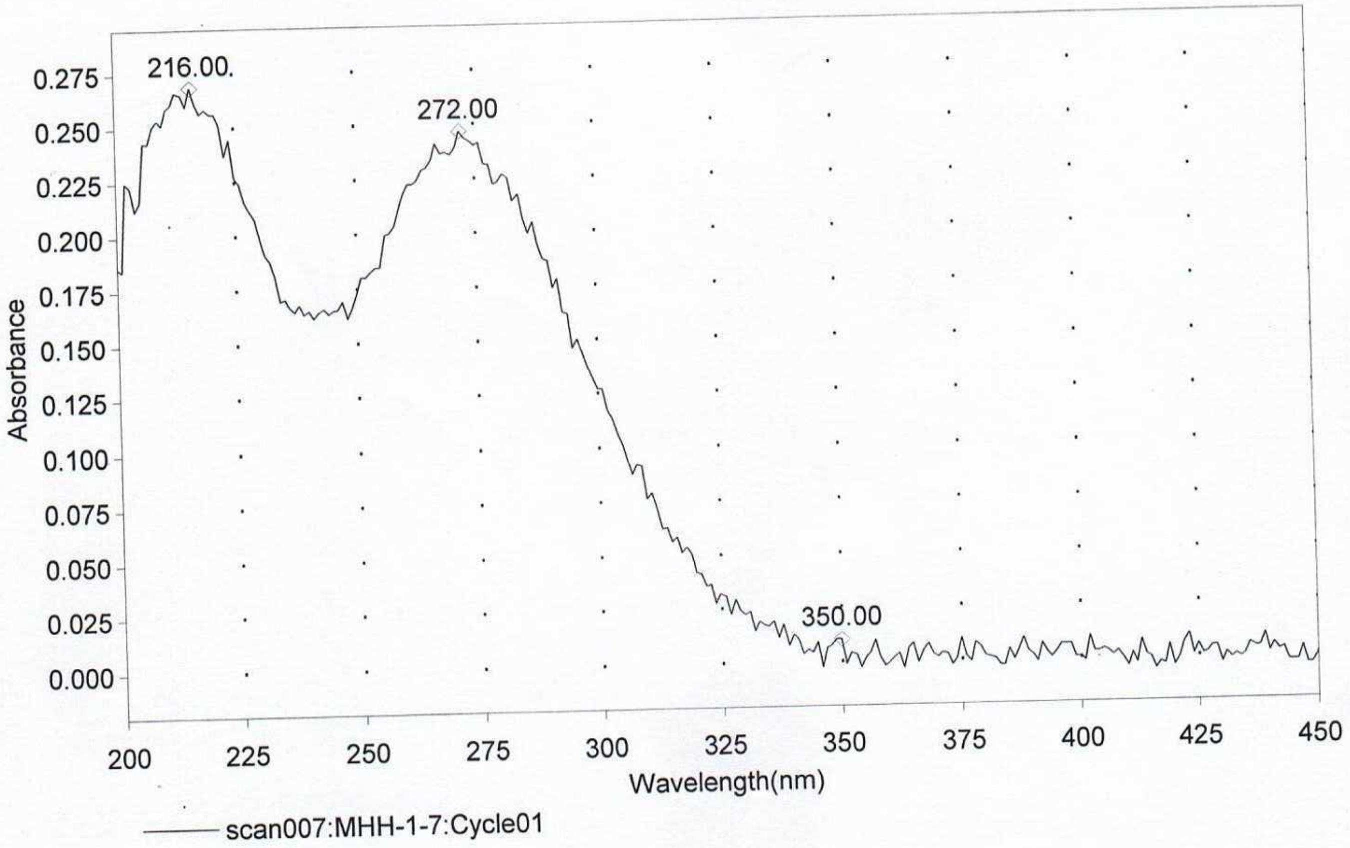

Results Table - MHH-1-7.sre,MHH-1-7,Cycle01

nm	A
216.00	0.269
272.00	0.247
350.00	0.010

Peak Pick Method

Find 8 Peaks Above -3.0000 A
Start Wavelength 200.00 nm
Stop Wavelength 450.00 nm
Sort By Wavelength

Sensitivity	Manual
Rising Points	3
Falling Points	3
Min. Change	0.0000
