# Supplementary material for: Sulphamethazine derivatives as immunomodulating agents: New therapeutic strategies for inflammatory diseases
Source: PLoS One. 2018 Dec 19;13(12):e0208933. doi: 10.1371/journal.pone.0208933 (PMC6300282; doi:10.1371/journal.pone.0208933)
Supplement: S12 Fig — (PDF) [file pone.0208933.s012.pdf]

AVANCE AV-400 MHz
Lab # 115

DR.HAROON/DR.HINA/MHH.I.22
1H

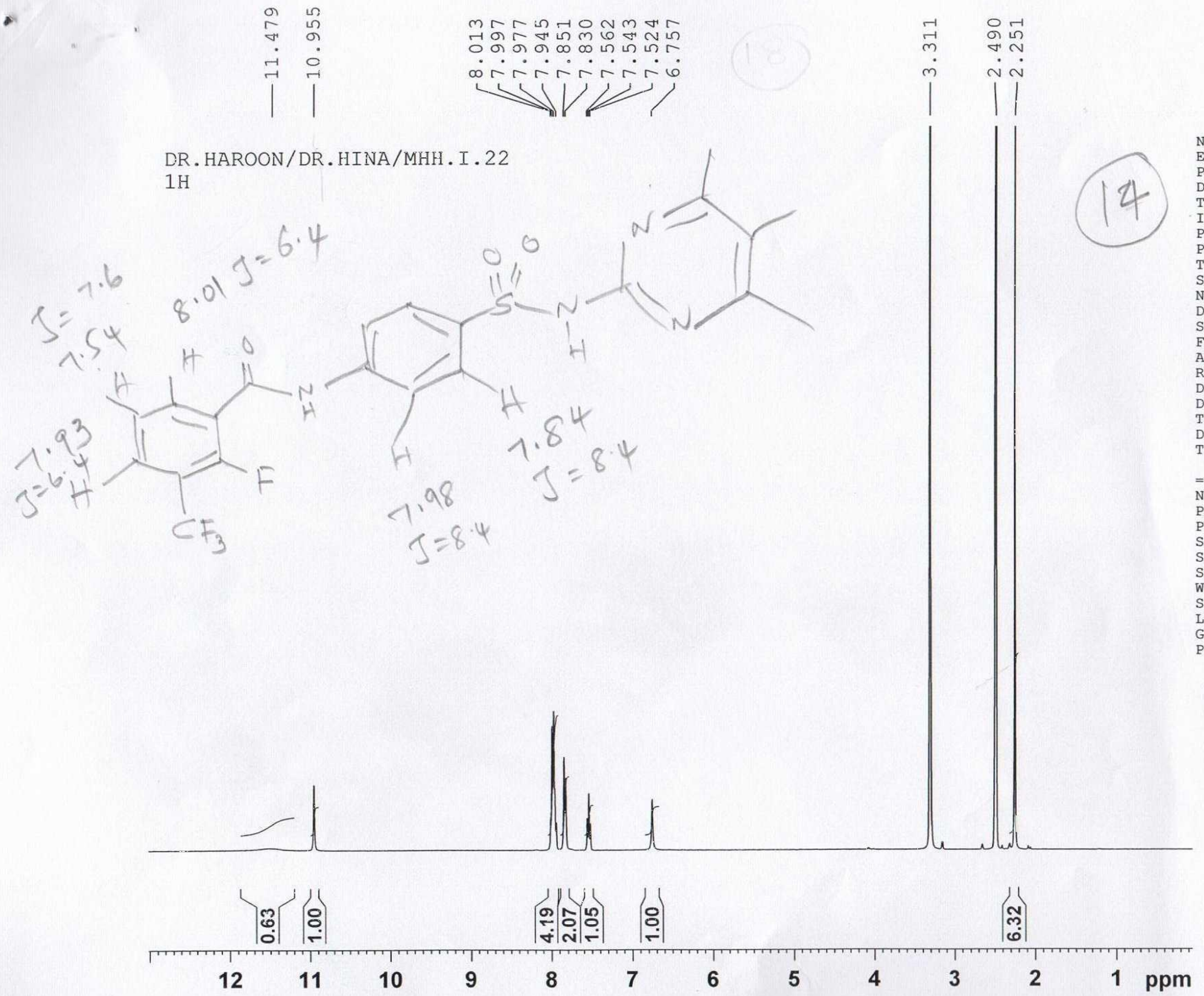

NAME	dec30-16
EXPNO	8
PROCNO	1
Date_	20161230
Time	11.29
INSTRUM	spect
PROBHD	5 mm SEI 1H-13
PULPROG	zg30
TD	65536
SOLVENT	DMSO
NS	64
DS	0
SWH	8012.820 Hz
FIDRES	0.122266 Hz
AQ	4.0894966 sec
RG	574.7
DW	62.400 usec
DE	6.50 usec
TE	300.0 K
D1	2.00000000 sec
TD0	1

======== CHANNEL f1 ========

NUC1	1H
P1	10.80 usec
PL1	3.00 dB
SFO1	400.0332002 MHz
SI	32768
SF	400.0300041 MHz
WDW	EM
SSB	0
LB	0.30 Hz
GB	0
PC	1.00

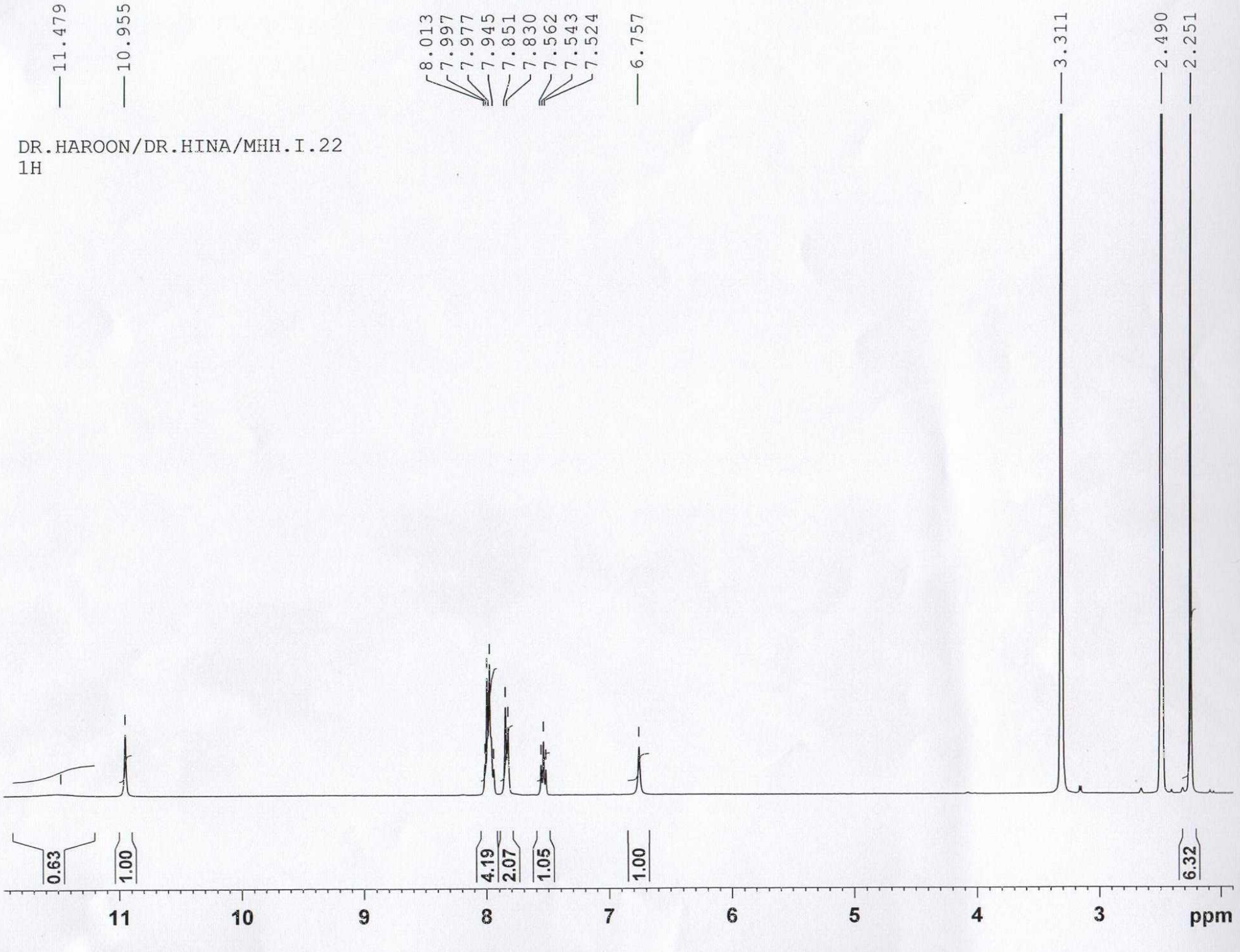
DR.HAROON/DR.HINA/MHH.I.22
1H
11.479
10.955
8.013
7.997
7.977
7.945
7.851
7.830
7.562
7.543
7.524
6.757
3.311
2.490
2.251
0.63
1.00
4.19
2.07
1.05
1.00
6.32
11
10
9
8
7
6
5
4
3
ppm

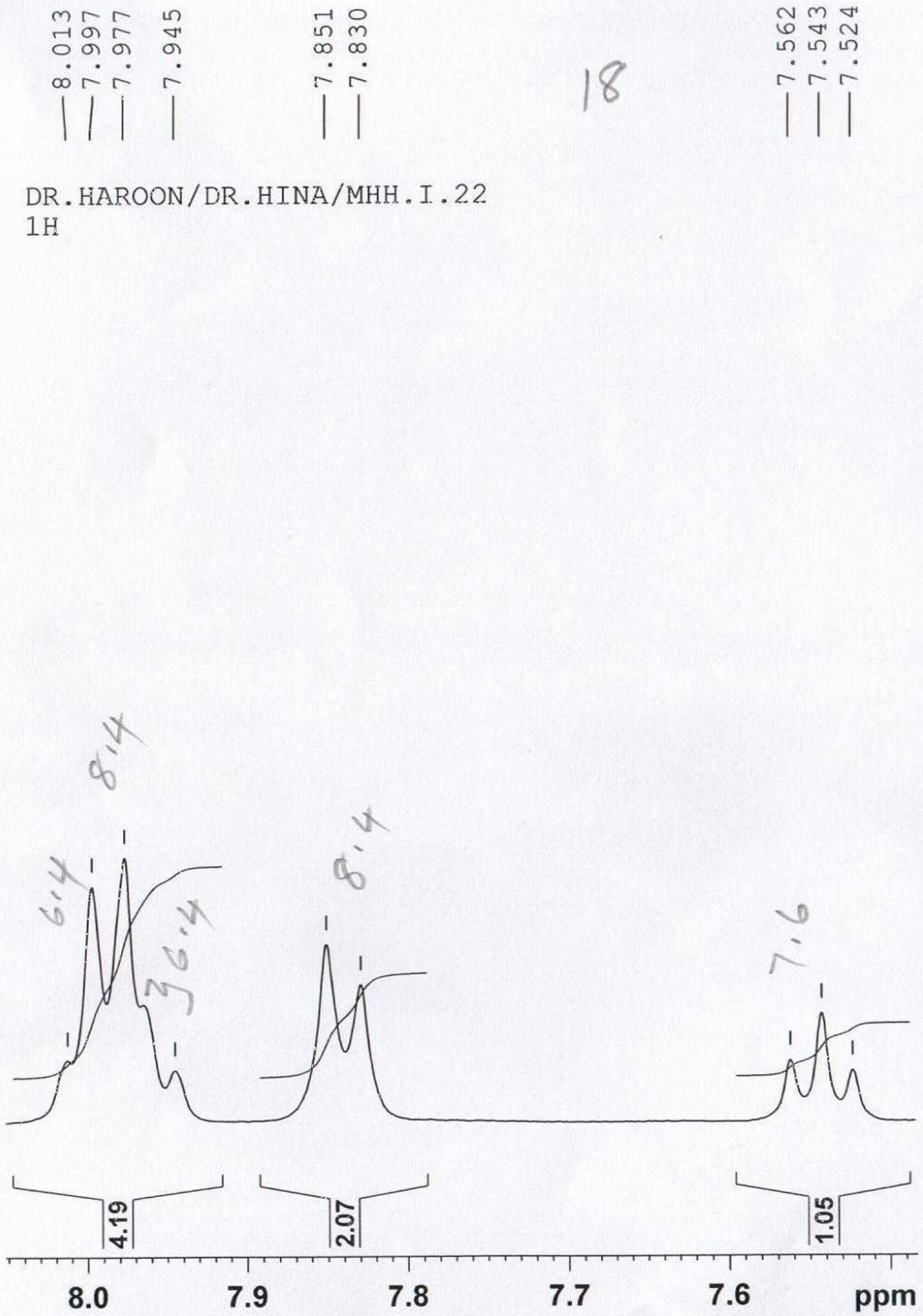
8.013
7.997
7.977
7.945
7.851
7.830
18
7.562
7.543
7.524
DR.HAROON/DR.HINA/MHH.I.22
1H
8.4
6.4
8.4
J 6.4
7.6
4.19
2.07
1.05
8.0
7.9
7.8
7.7
7.6
ppm

DR.M.H.HAROON/DR.HINA/MHH-1-22/DMSO
ICCBS,U.O.K/BB

AVANCE 400
LAB NO 117

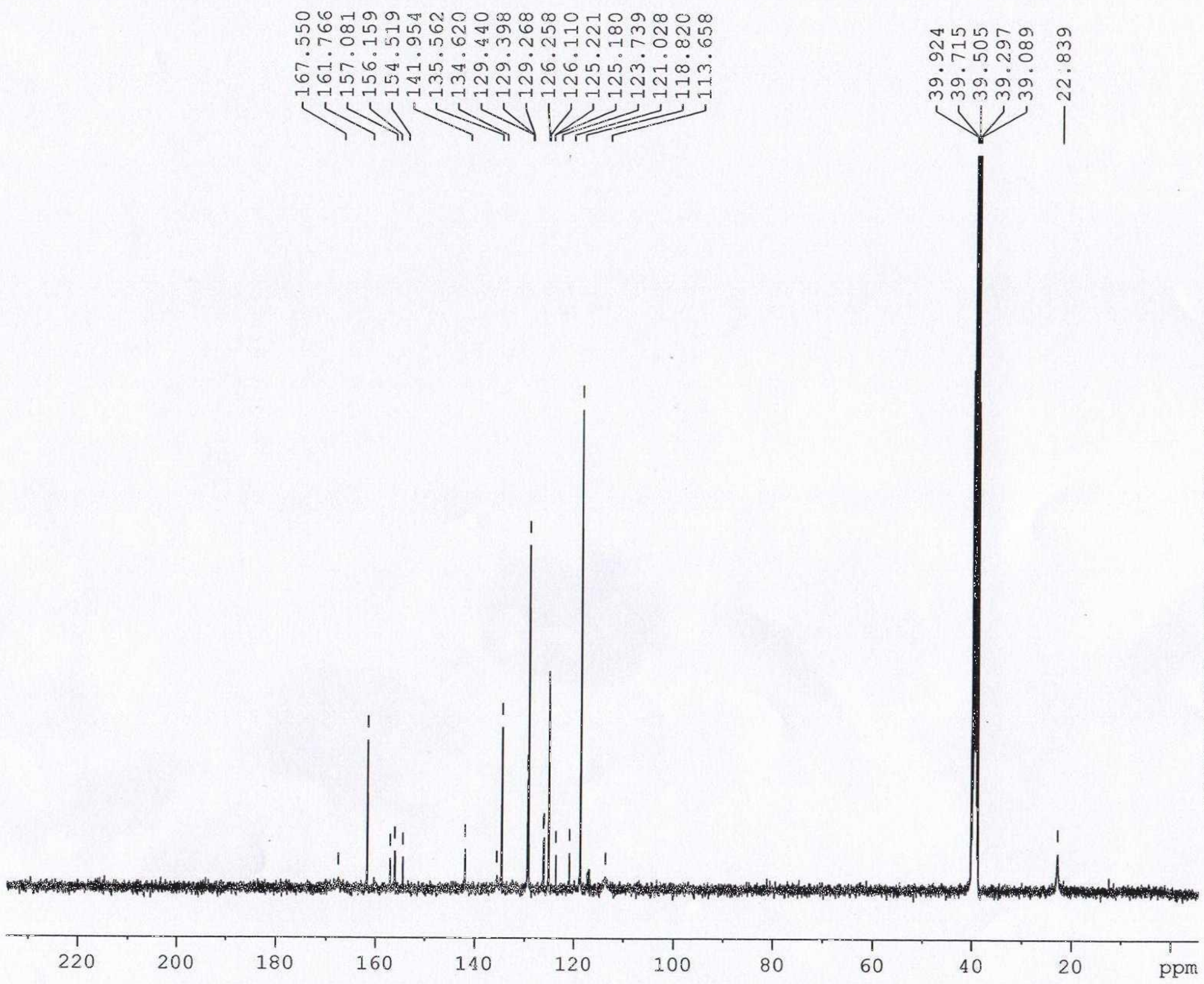

NAME	apr25-17
EXPNO	14
PROCNO	1
Date_	20170425
Time	15.40
INSTRUM	spect
PROBHD	5 mm DUL 13C-1
PULPROG	zgpg
TD	32768
SOLVENT	DMSO
NS	18432
DS	0
SWH	24154.590 Hz
FIDRES	0.737140 Hz
AQ	0.6783476 sec
RG	32768
DW	20.700 usec
DE	6.50 usec
TE	300.0 K
D1	2.00000000 sec
D11	0.03000000 sec
TD0	18

======== CHANNEL f1 ========

NUC1	13C
P1	8.55 usec
PL1	7.00 dB
SFO1	100.6243395 MHz

======== CHANNEL f2 ========

CPDPRG2	waltz16
NUC2	1H
PCPD2	80.00 usec
PL2	0.00 dB
PL12	19.00 dB
PL13	20.00 dB
SFO2	400.1324008 MHz
SI	16384
SF	100.6128205 MHz
WDW	EM
SSB	0
LB	1.00 Hz
GB	0
PC	1.00

DR.M.H.HAROON/DR.HINA/MHH-1-22/DMSO
ICCBS,U.O.K/DEPT-135

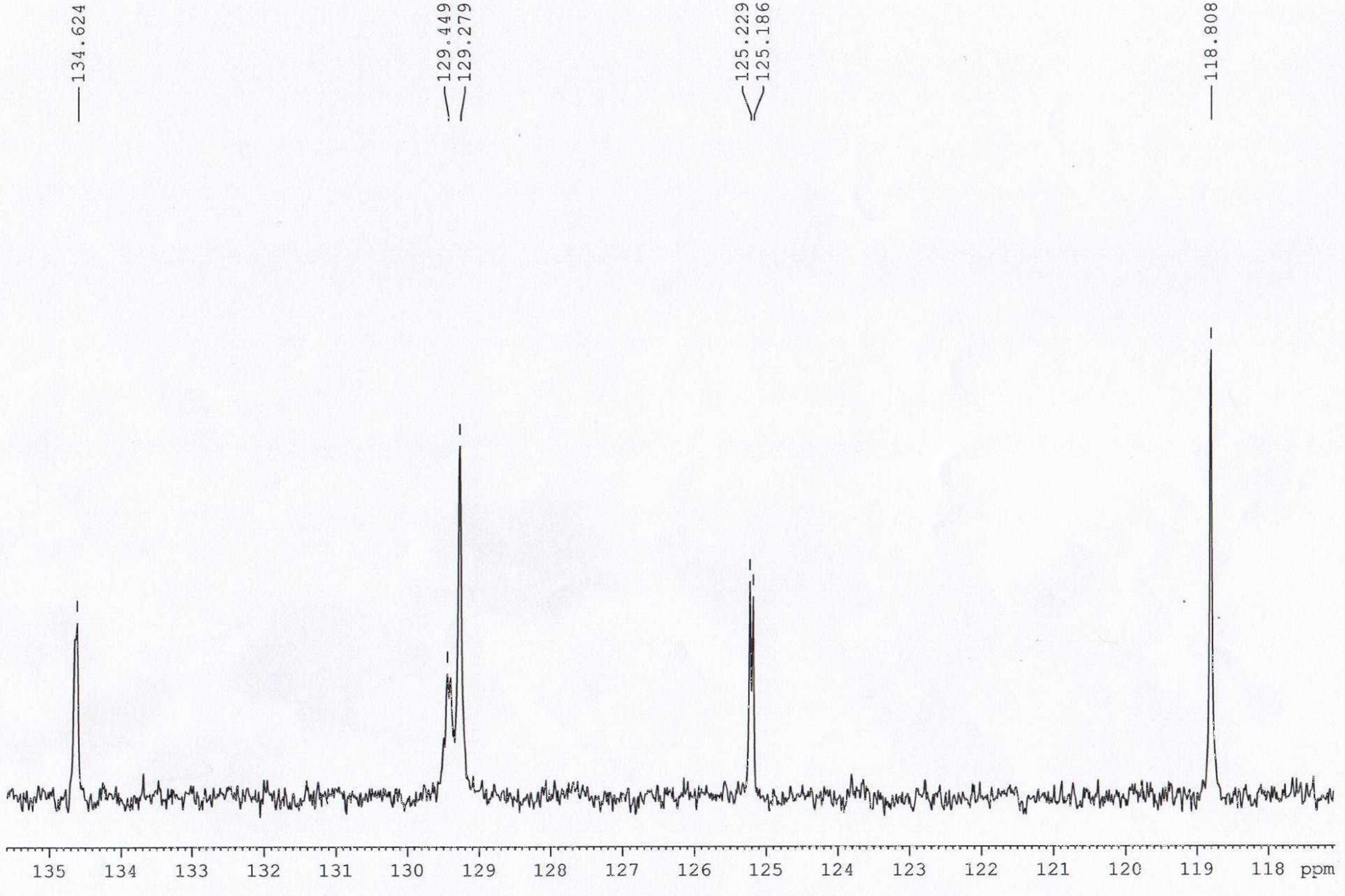

DR.M.H.HAROON/DR.HINA/MHH-1-22/DMSO
ICCBS,U.O.K/BB

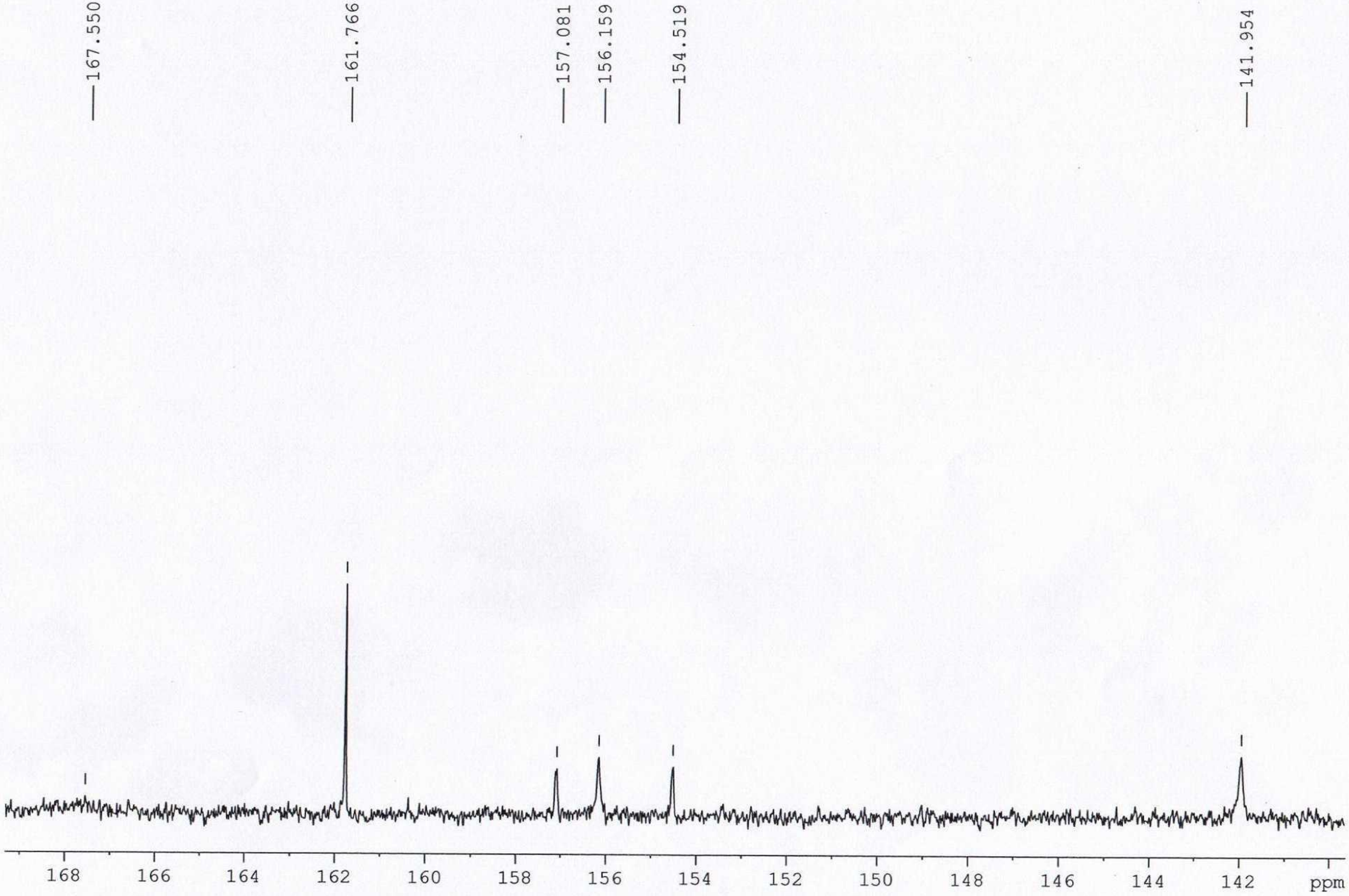

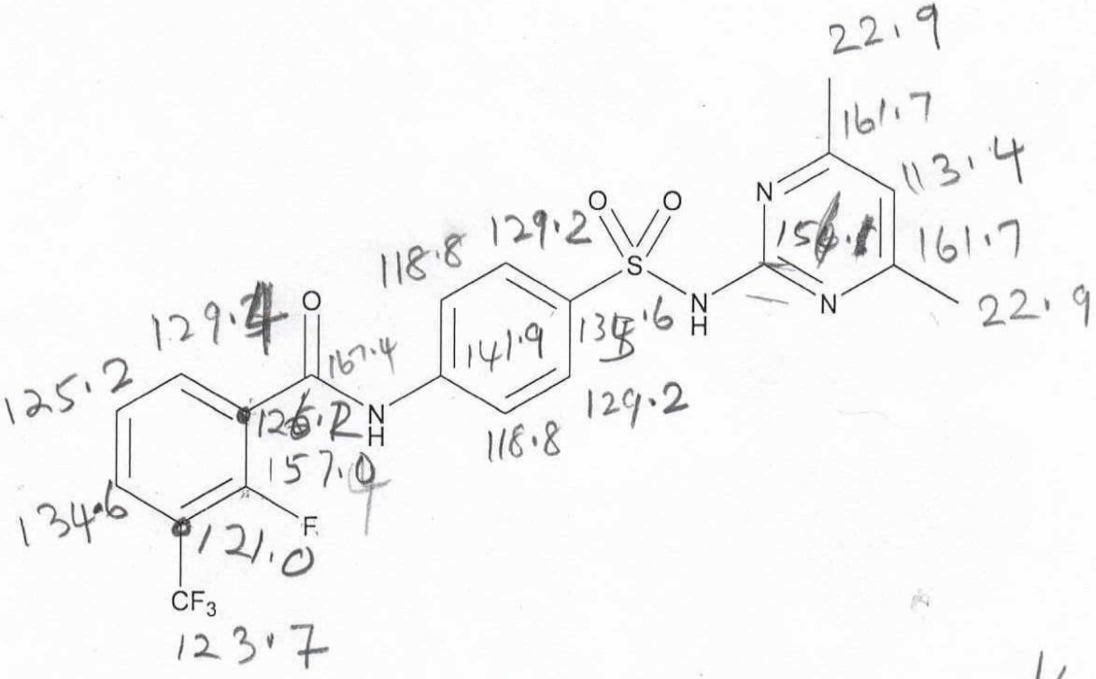

MHA-1 – 22

Re check

check

for one peak

[illegible]

JEOL HX 110 MASS SPECTROMETER (FAB-HR)

STUDENT NAME	Dr. Haroon	SAMPLE CODE	DATE	24-5-17
SUPERVISOR NAME	Dr. Hina	MHH-I-22	FAB (+VE / -VE)	+ve

Mass	Theoretical Mass	Delta [ppm]	Delta [mmu]	RDB	Composition
469.0989	469.0988	0.1	0.1	24.5	$C_{30} H_{14} O_3 N_2 F_1$
	469.0986	0.6	0.3	20.5	$C_{28} H_{16} N_2 F_3 S_1$
	469.0998	-1.9	-0.9	16.5	$C_{25} H_{17} O_1 N_2 F_4 S_1$
	469.0977	2.6	1.2	28.5	$C_{33} H_{13} O_2 N_2$
	469.1011	-4.6	-2.2	23.5	$C_{30} H_{17} O_2 N_2 S_1$
	469.0964	5.3	2.5	21.5	$C_{28} H_{13} O_1 N_2 F_4$
	469.1015	-5.6	-2.6	29.0	$C_{33} H_{12} N_3 F_1$
	469.1017	-6.0	-2.8	32.5	$C_{38} H_{13}$
	469.0959	6.3	3.0	16.0	$C_{25} H_{18} O_3 N_1 F_3 S_1$
	469.0957	6.7	3.2	12.5	→ $C_{20} H_{17} O_3 N_4 F_4 S_1$
	469.1022	-7.1	-3.3	19.5	$C_{27} H_{18} O_3 N_2 F_1 S_1$
	469.0953	7.8	3.6	25.5	$C_{31} H_{12} N_2 F_3$
	469.1027	-8.0	-3.8	25.0	$C_{30} H_{13} O_1 N_3 F_2$
	469.1029	-8.5	-4.0	28.5	$C_{35} H_{14} O_1 F_1$
	469.0948	8.7	4.1	20.0	$C_{28} H_{17} O_2 N_1 F_2 S_1$

$[M+H]^+$

File: MHH-I-22 **Date Run: 02-15-2017 (Time Run: 09:12:55)**
Sample: DR.M.H.HAROON /DR. HINA
Instrument: JEOL MS 600H-1
Ionization mode: EI+

Scan: 16 **R.T.: 1.33**
Base: m/z 403; 99.5%FS TIC: 4595572

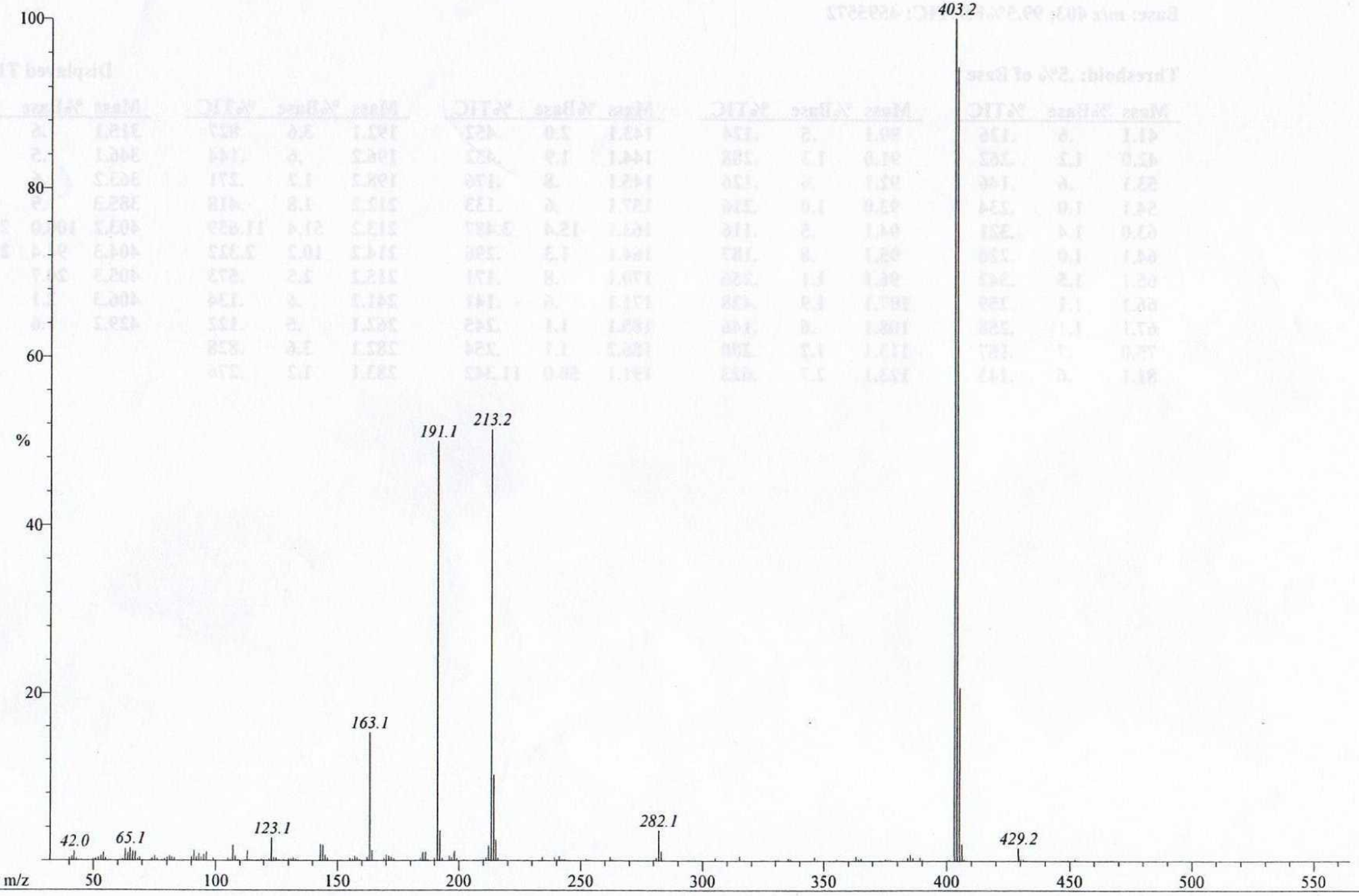

I. C.C.B.S., University of Karachi Analytical Laboratory - Pakistan

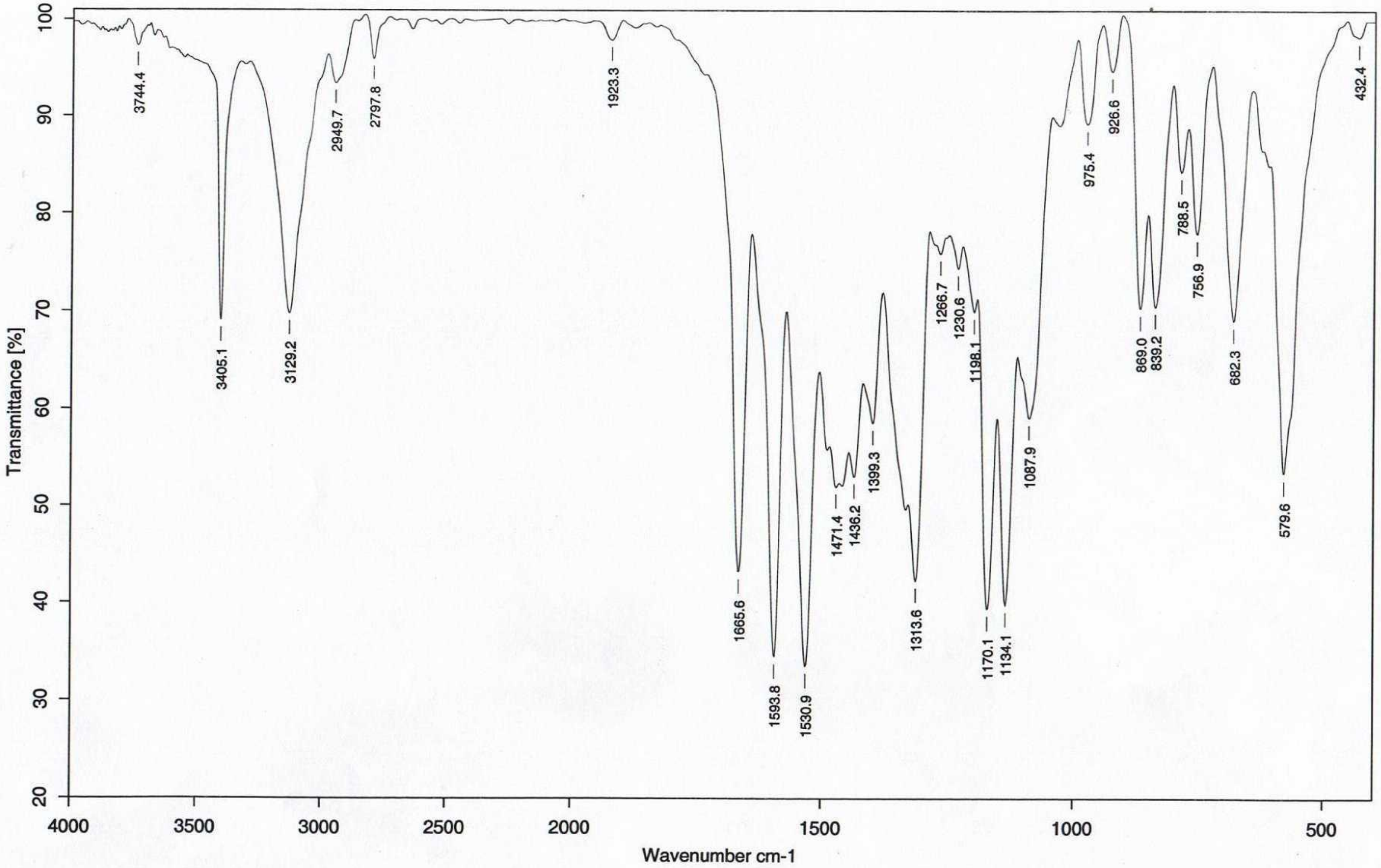

Sample : MHH-1-22/Haroon/Dr. Hina	Spectrum : MHH-1-22.0 (in D:\IRSTUDENT)
Measured : 01/02/2017 on VECTOR22	Technic : Solid
Resolution : 4 cm-1 (10 scans)	Analyst : ZA/Jamshed/M. Asif/Haroon

ᛐHERMO ELECTRON ~ VISIONpro SOFTWARE V4.10

)perator Name	ARSHAD ALAM.	Date of Report	2/2/2017
)epartment	Analytical Laboratory TWC # 004	Time of Report	3:23:36PM
)rganization	ICCBS Karachi of Universty.		
ıformation	Dr.Haroon/ Dr.Hina		

;can Graph

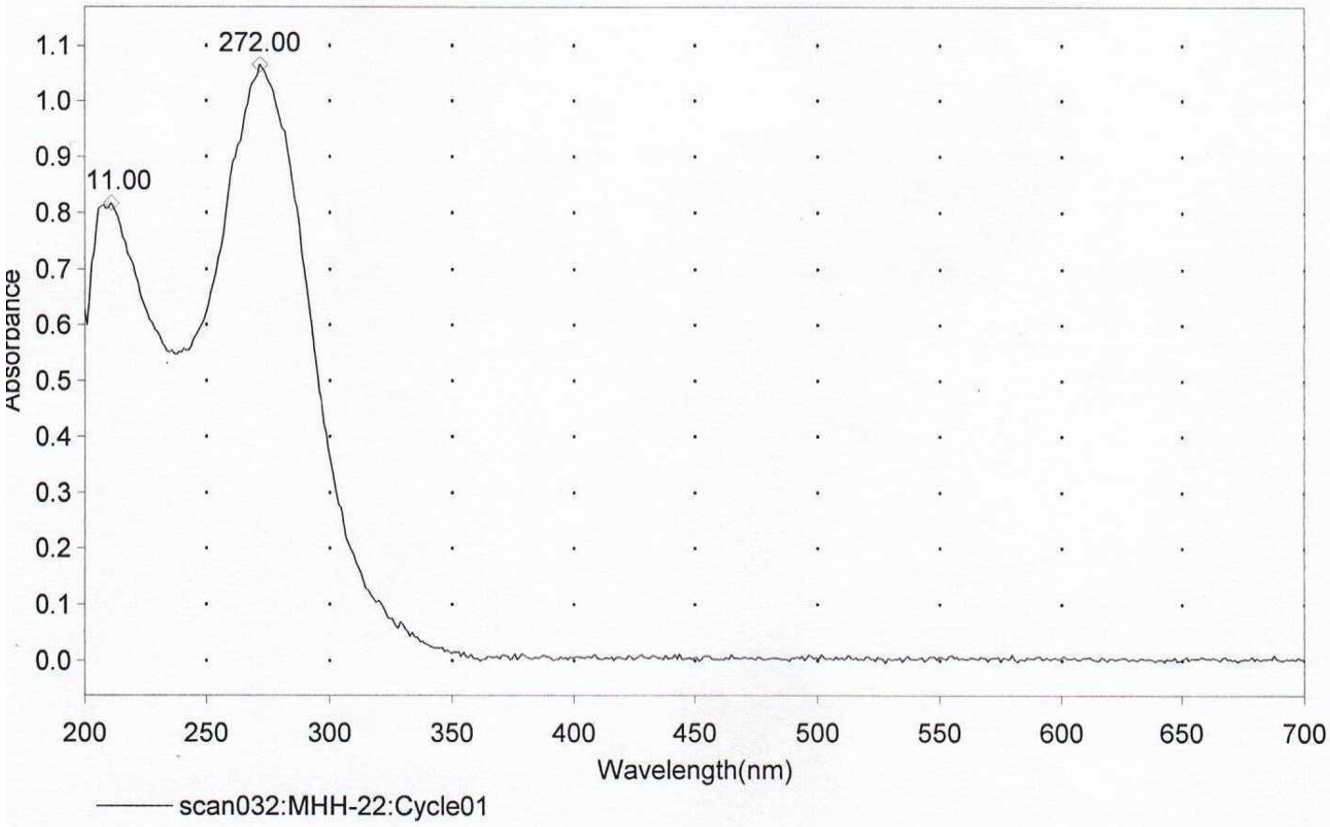

ƎResults Table - MHH-1-22.sre,MHH-22,Cycle01

ım	A	**Peak Pick Method**
:11.00	0.818	Find 8 Peaks Above -3.0000 A
:72.00	1.065	Start Wavelength 200.00 nm
		Stop Wavelength 700.00 nm
		Sort By Wavelength

;ensitivity Auto
