# Supplementary material for: Sulphamethazine derivatives as immunomodulating agents: New therapeutic strategies for inflammatory diseases
Source: PLoS One. 2018 Dec 19;13(12):e0208933. doi: 10.1371/journal.pone.0208933 (PMC6300282; doi:10.1371/journal.pone.0208933)
Supplement: S13 Fig — (PDF) [file pone.0208933.s013.pdf]

DR.HAROON/DR.HINA/MHH.I.49
1H

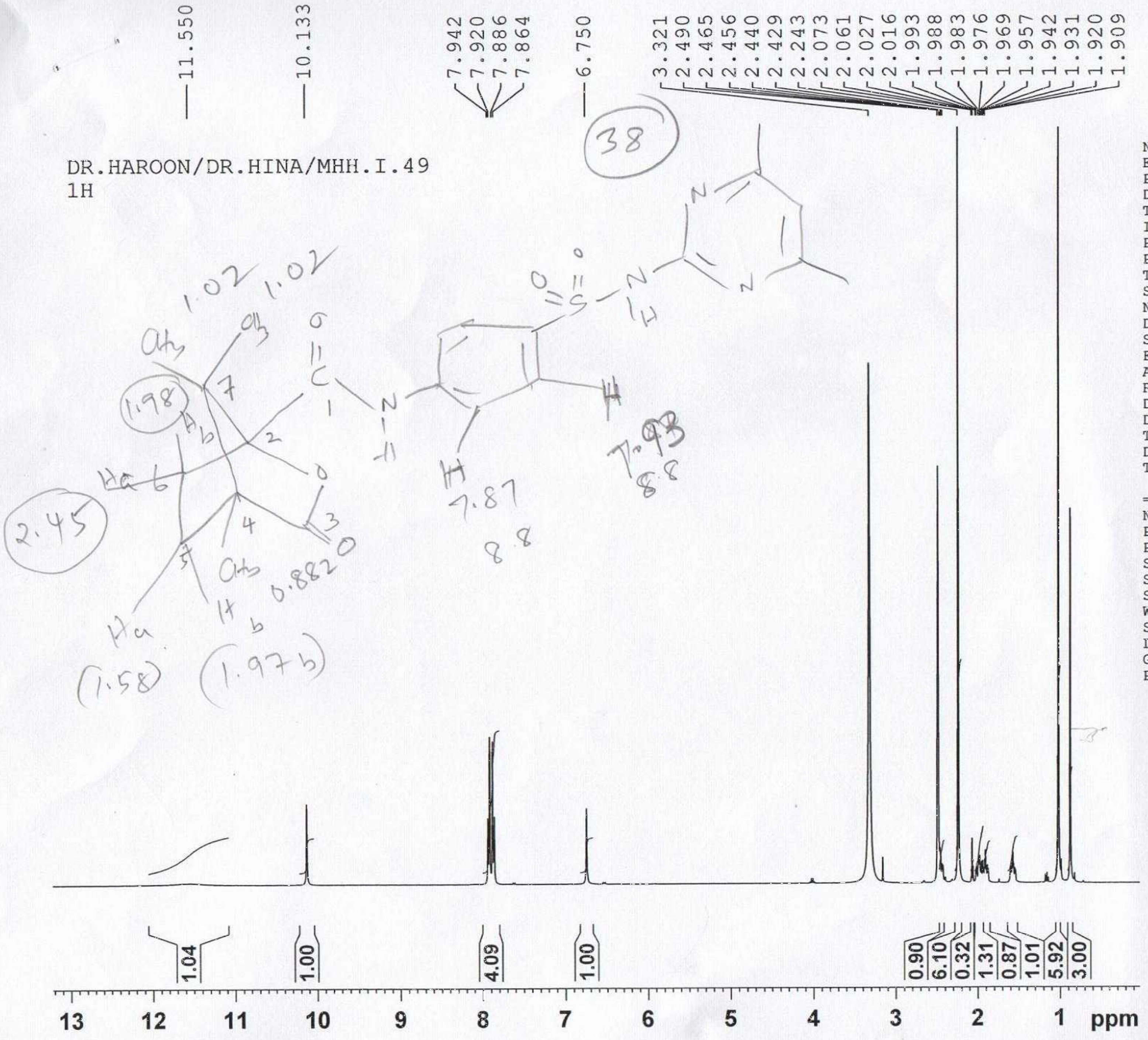

AVANCE AV-400 MHz
Lab # 115

15

NAME	march02-17
EXPNO	3
PROCNO	1
Date_	20170302
Time	10.43
INSTRUM	spect
PROBHD	5 mm SEI 1H-13
PULPROG	zg30
TD	32768
SOLVENT	DMSO
NS	64
DS	0
SWH	8012.820 Hz
FIDRES	0.244532 Hz
AQ	2.0447731 sec
RG	228.1
DW	62.400 usec
DE	6.50 usec
TE	300.0 K
D1	2.00000000 sec
TD0	1

======== CHANNEL f1 ========

NUC1	1H
P1	10.63 usec
PL1	2.00 dB
SFO1	400.0332002 MHz
SI	16384
SF	400.0300041 MHz
WDW	EM
SSB	0
LB	0.30 Hz
GB	0
PC	1.00

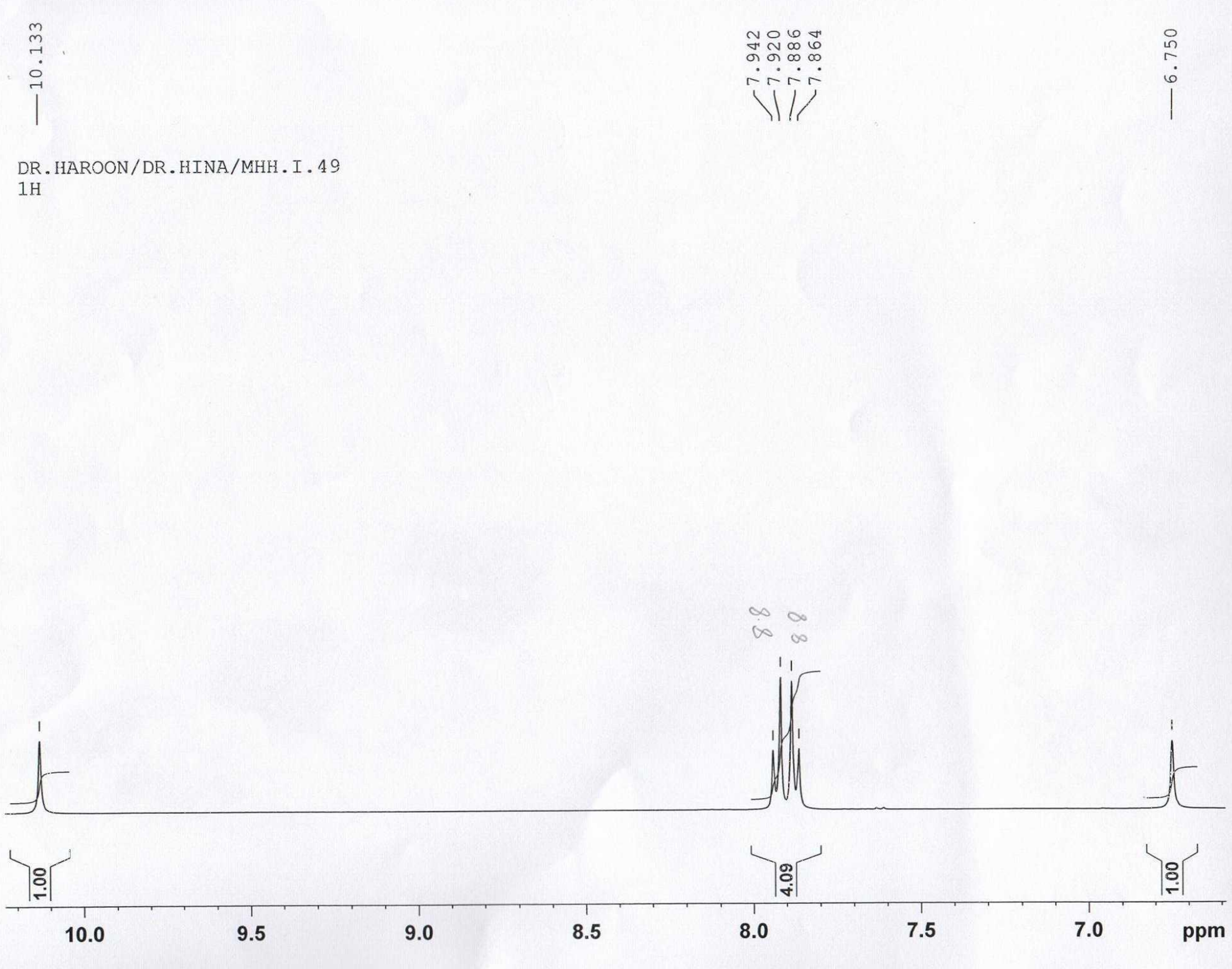

10.133
7.942
7.920
7.886
7.864
6.750
DR.HAROON/DR.HINA/MHH.I.49
1H
8.8
8.8
1.00
4.09
1.00
10.0
9.5
9.0
8.5
8.0
7.5
7.0
ppm

Dr. Haroon / Dr. Hina / MHH-I-49
ICCBS,U.O.K/BB

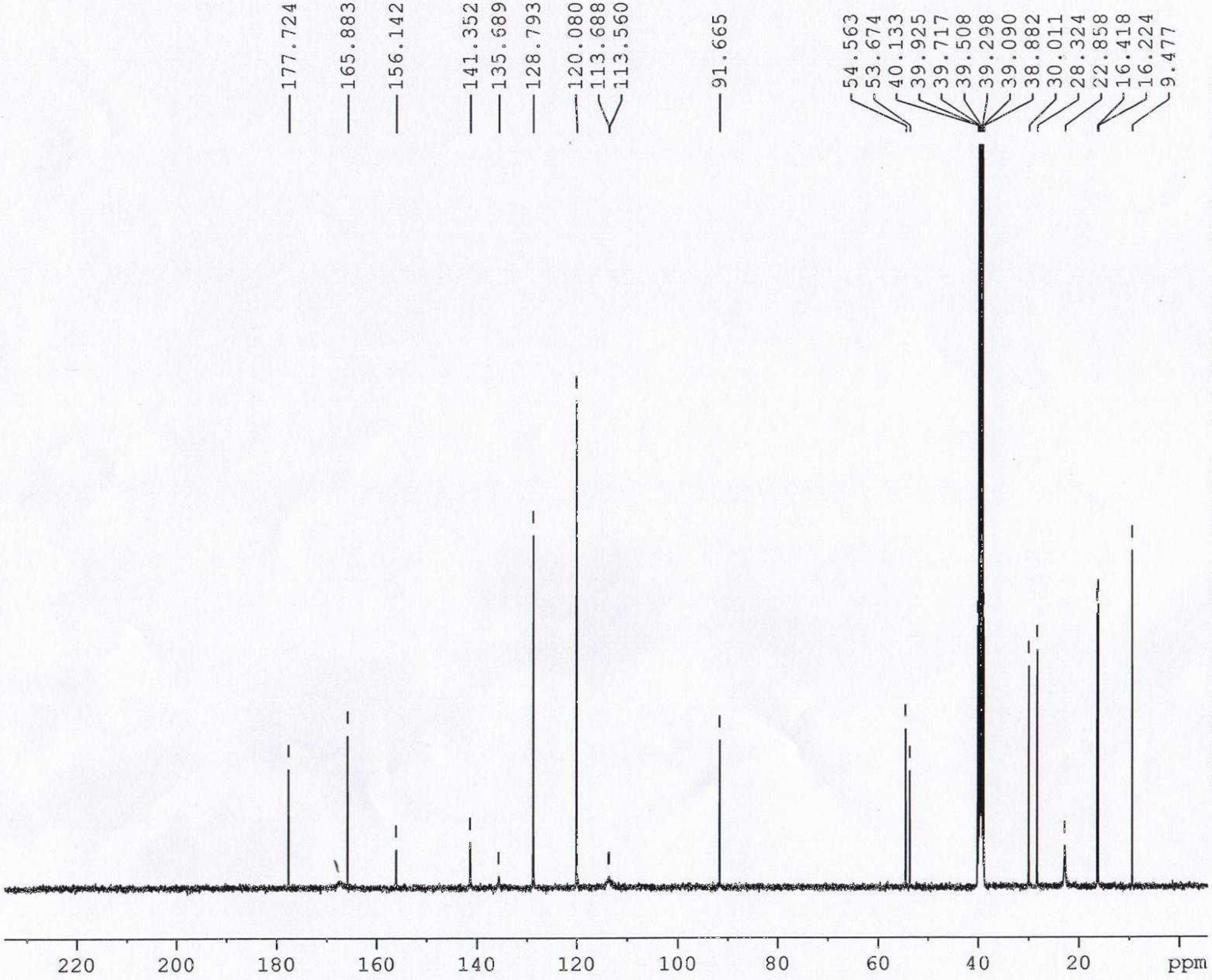

AVANCE 400
LAB NO 117

NAME	may04-17
EXPNO	7
PROCNO	1
Date_	20170504
Time	15.24
INSTRUM	spect
PROBHD	5 mm DUL 13C-1
PULPROG	zgpg
TD	32768
SOLVENT	DMSO
NS	18432
DS	0
SWH	24154.590 Hz
FIDRES	0.737140 Hz
AQ	0.6783476 sec
RG	32768
DW	20.700 usec
DE	6.50 usec
TE	300.0 K
D1	2.00000000 sec
D11	0.03000000 sec
TD0	18

======== CHANNEL f1 ========

NUC1	13C
P1	8.55 usec
PL1	7.00 dB
SFO1	100.6243395 MHz

======== CHANNEL f2 ========

CPDPRG2	waltz16
NUC2	1H
PCPD2	80.00 usec
PL2	0.00 dB
PL12	19.00 dB
PL13	20.00 dB
SFO2	400.1324008 MHz
SI	16384
SF	100.6128205 MHz
WDW	EM
SSB	0
LB	1.00 Hz
GB	0
PC	1.00

Dr. Haroon / Dr. Hina / MHH-I-49
ICCBS,U.O.K/BB

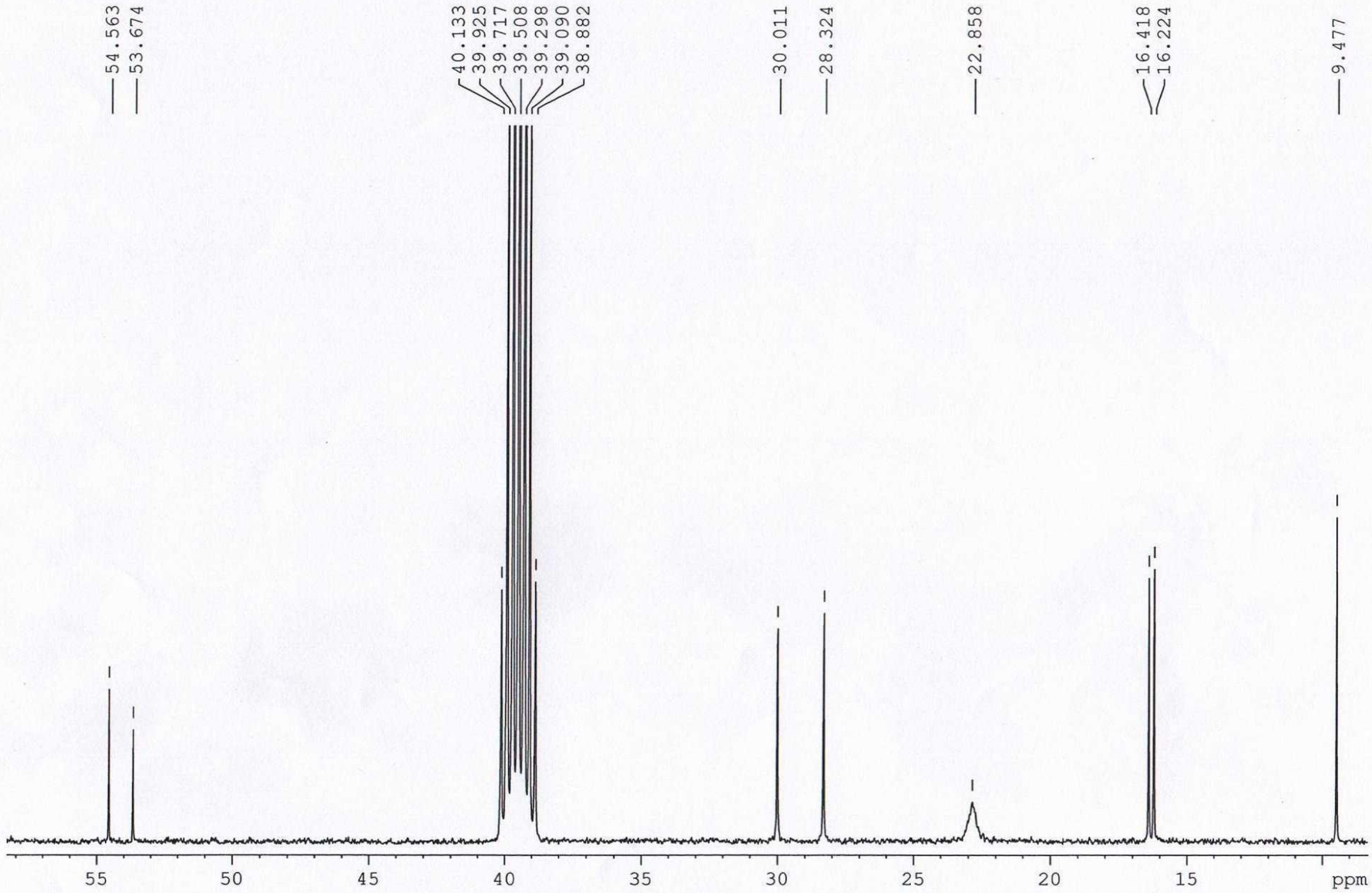

Dr. Haroon / Dr. Hina / MHH-I-49
ICCBS,U.O.K/DEPT-135

AVANCE 400
LAB NO 117

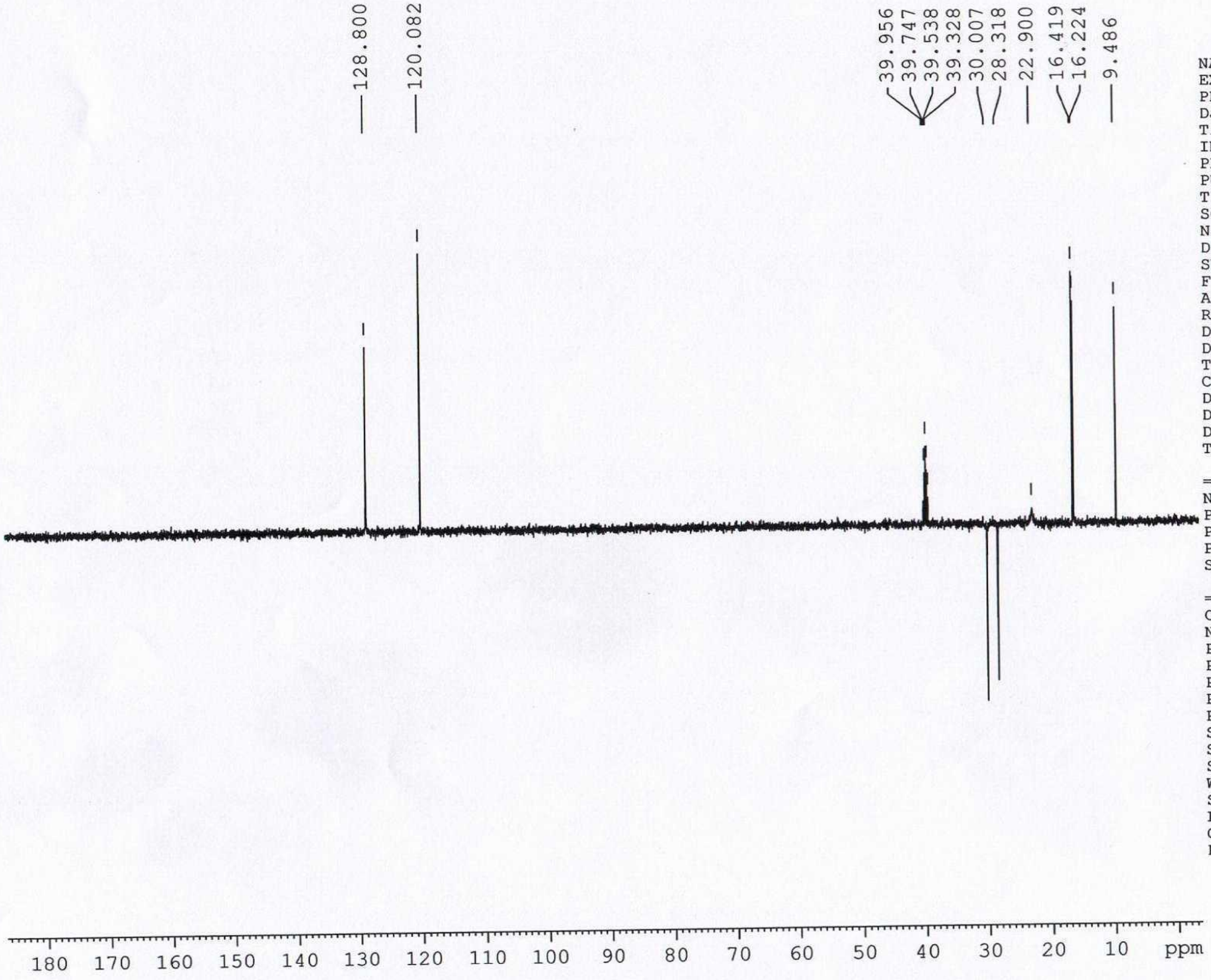

NAME	may04-17
EXPNO	8
PROCNO	1
Date_	20170505
Time	5.29
INSTRUM	spect
PROBHD	5 mm DUL 13C-1
PULPROG	dept135
TD	32768
SOLVENT	DMSO
NS	5308
DS	2
SWH	19157.088 Hz
FIDRES	0.584628 Hz
AQ	0.8552948 sec
RG	32768
DW	26.100 usec
DE	6.50 usec
TE	300.0 K
CNST2	145.0000000
D1	2.00000000 sec
D2	0.00344828 sec
D12	0.00002000 sec
TD0	9

======== CHANNEL f1 ========

NUC1	13C
P1	8.55 usec
P2	17.10 usec
PL1	7.00 dB
SFO1	100.6220254 MHz

======== CHANNEL f2 ========

CPDPRG2	waltz16
NUC2	1H
P3	9.50 usec
P4	19.00 usec
PCPD2	80.00 usec
PL2	0.00 dB
PL12	19.00 dB
SFO2	400.1320007 MHz
SI	16384
SF	100.6128205 MHz
WDW	EM
SSB	0
LB	1.00 Hz
GB	0
PC	1.40

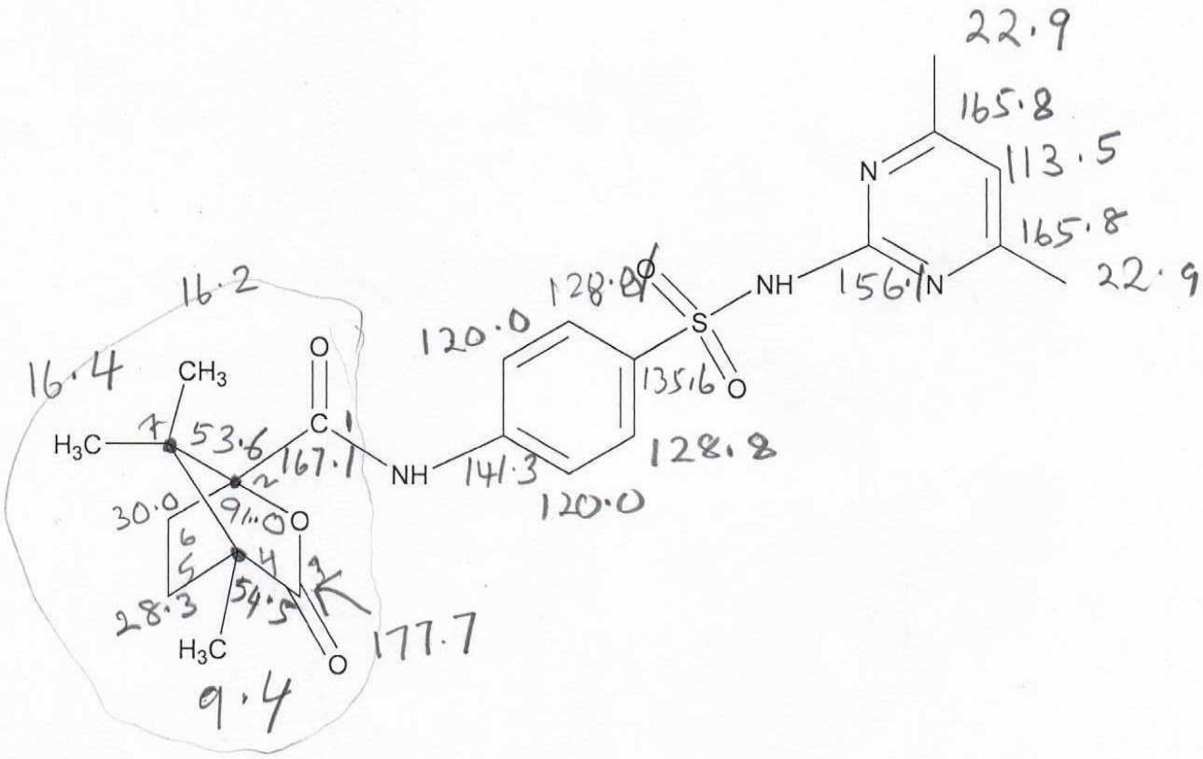
22.9
165.8
113.5
165.8
22.9
156.1
NH
N
N
O
S
O
128.8
135.6
128.8
120.0
141.3
120.0
NH
O
C
167.1
16.2
16.4
CH3
H3C
53.6
30.0
91.0
O
28.3
54.5
177.7
H3C
O
9.4

MHH 1-49

JEOL HX 110 MASS SPECTROMETER (FAB-HR)

STUDENT NAME	Haroon	SAMPLE CODE	DATE	23/5/17
SUPERVISOR NAME	Dr. Hira	MHH-I-49	FAB (+VE / -VE)	+Ve

Mass	Theoretical Mass	Delta [ppm]	Delta [mmu]	RDB	Composition
459.1721	459.1716	1.2	0.5	11.0	$C_{24} H_{29} O_6 N_1 S_1$
	459.1727	-1.3	-0.6	7.5	$C_{18} H_{27} O_{10} N_4$
	459.1709	2.7	1.2	20.5	$C_{30} H_{23} O_3 N_2$
	459.1735	-3.2	-1.4	25.0	$C_{33} H_{21} N_3$
	459.1702	4.1	1.9	11.5	$C_{22} H_{27} O_5 N_4 S_1$

File: MHH-1_49-FABP
Date Run: 03-15-2017 (Time Run: 09:09:08)
Sample: DR.M.H.HAROON /DR. HINA
Instrument: JEOL-600H-2
Inlet: Direct Probe
Ionization mode: FAB+

Scan: 10 **R.T.: .8**
Base: m/z 185; 8.4%FS TIC: 395614 **#Ions: 697**

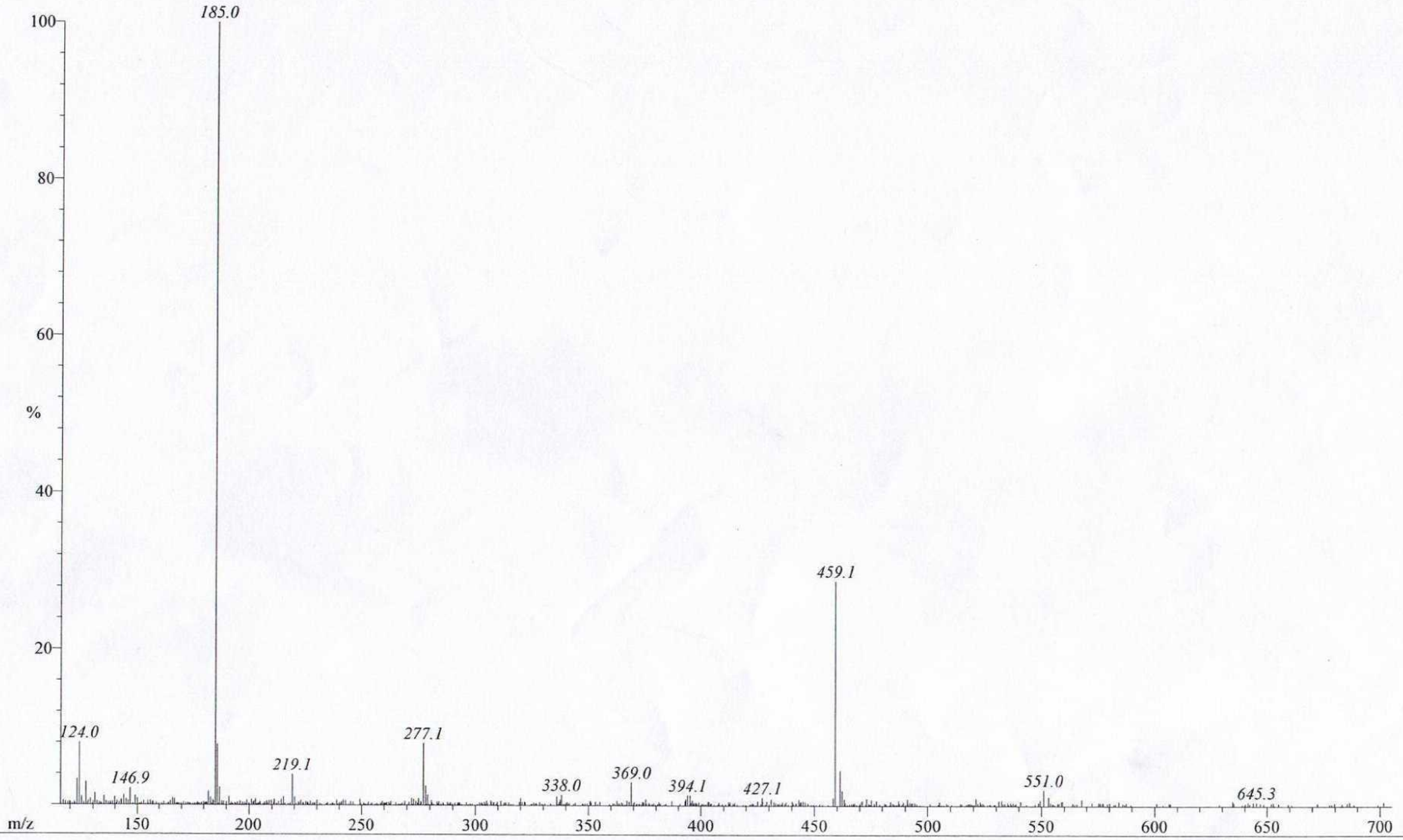

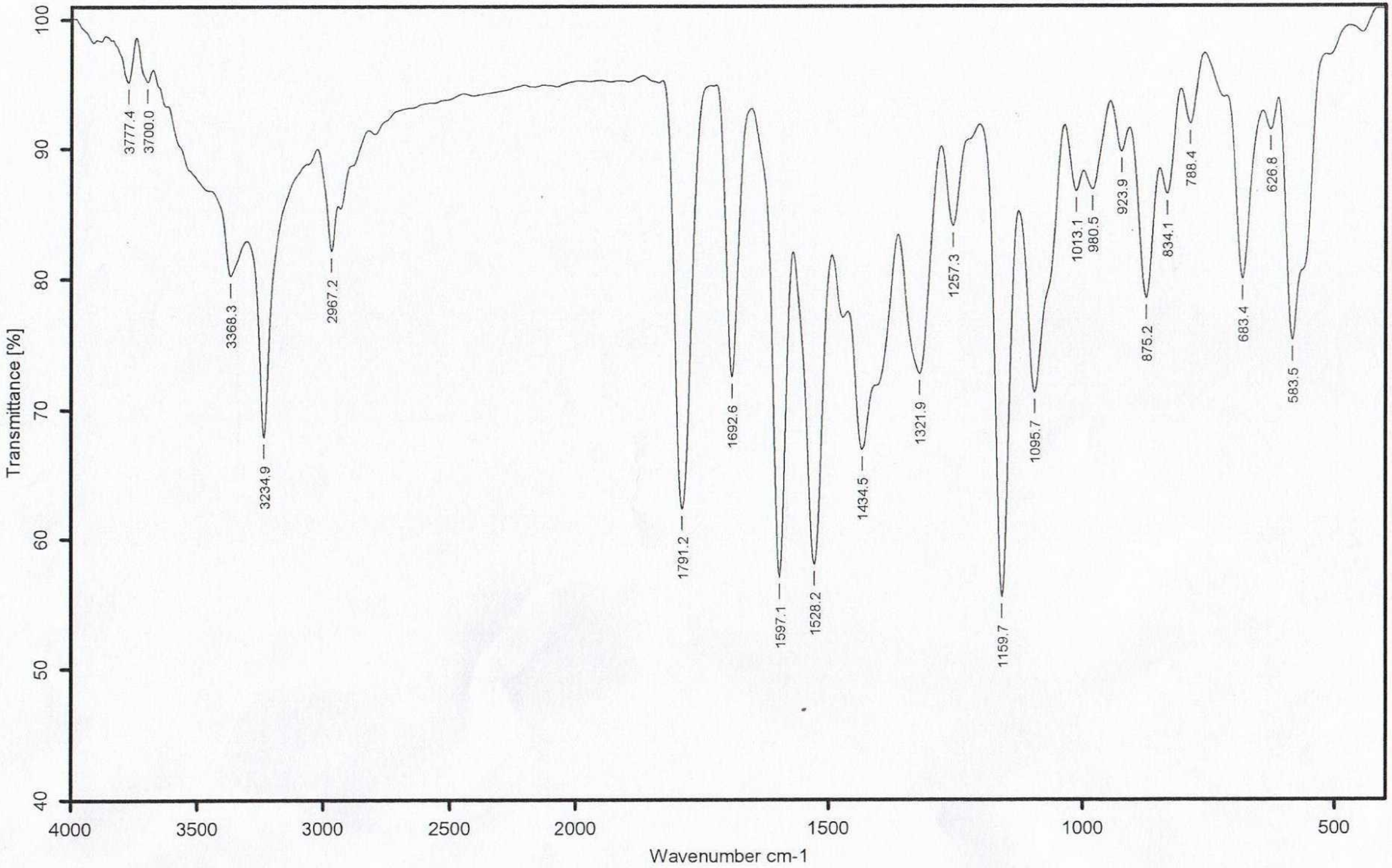

Sample : MHH-I-49/Dr. Haroon/Dr. Hina

Measured : 05/07/2017 on VECTOR22

Resolution : 4 cm-1 (10 scans)

Spectrum : MHH-I-49.0 (in D:\IRSTUDENT)

Technic : Solid

Analyst : MA/ZA

'HERMO ELECTRON ~ VISIONpro SOFTWARE V4.10

'perator Name	ARSHAD ALAM	Date of Report	7/5/2017
'epartment	Analytical Laboratory TWC # 004	Time of Report	11:25:53AM
'rganization	ICCBS Karachi of Universty.		
ıformation	Dr. Haroon /Dr. Hina		

can Graph

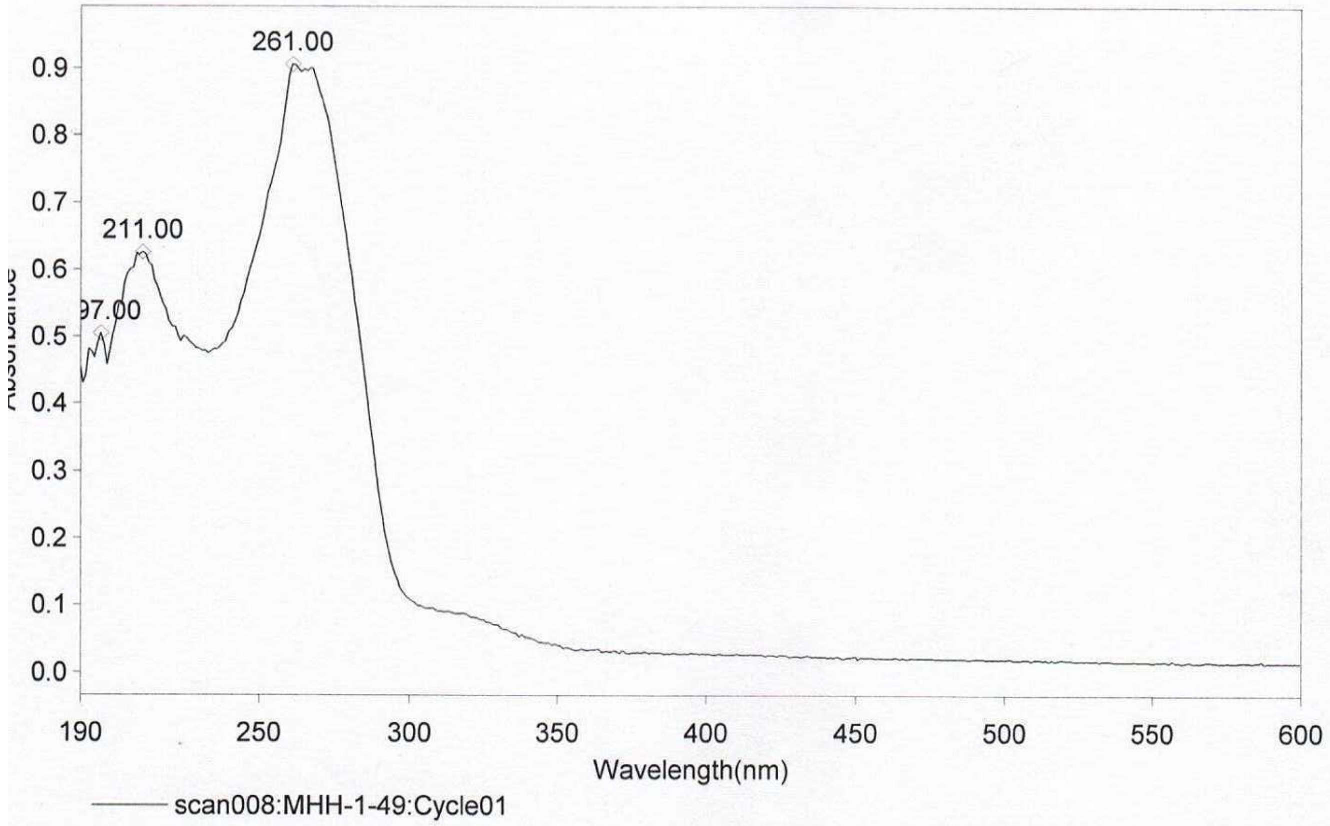

esults Table - MHH-1-49.sre,MHH-1-49,Cycle01

m	A	**Peak Pick Method**
97.00	0.505	Find 4 Peaks Above -3.0000 A
11.00	0.626	Start Wavelength 190.00 nm
51.00	0.906	Stop Wavelength 600.00 nm
		Sort By Wavelength

ensitivity Auto
