# Supplementary material for: Sulphamethazine derivatives as immunomodulating agents: New therapeutic strategies for inflammatory diseases
Source: PLoS One. 2018 Dec 19;13(12):e0208933. doi: 10.1371/journal.pone.0208933 (PMC6300282; doi:10.1371/journal.pone.0208933)
Supplement: S14 Fig — (PDF) [file pone.0208933.s014.pdf]

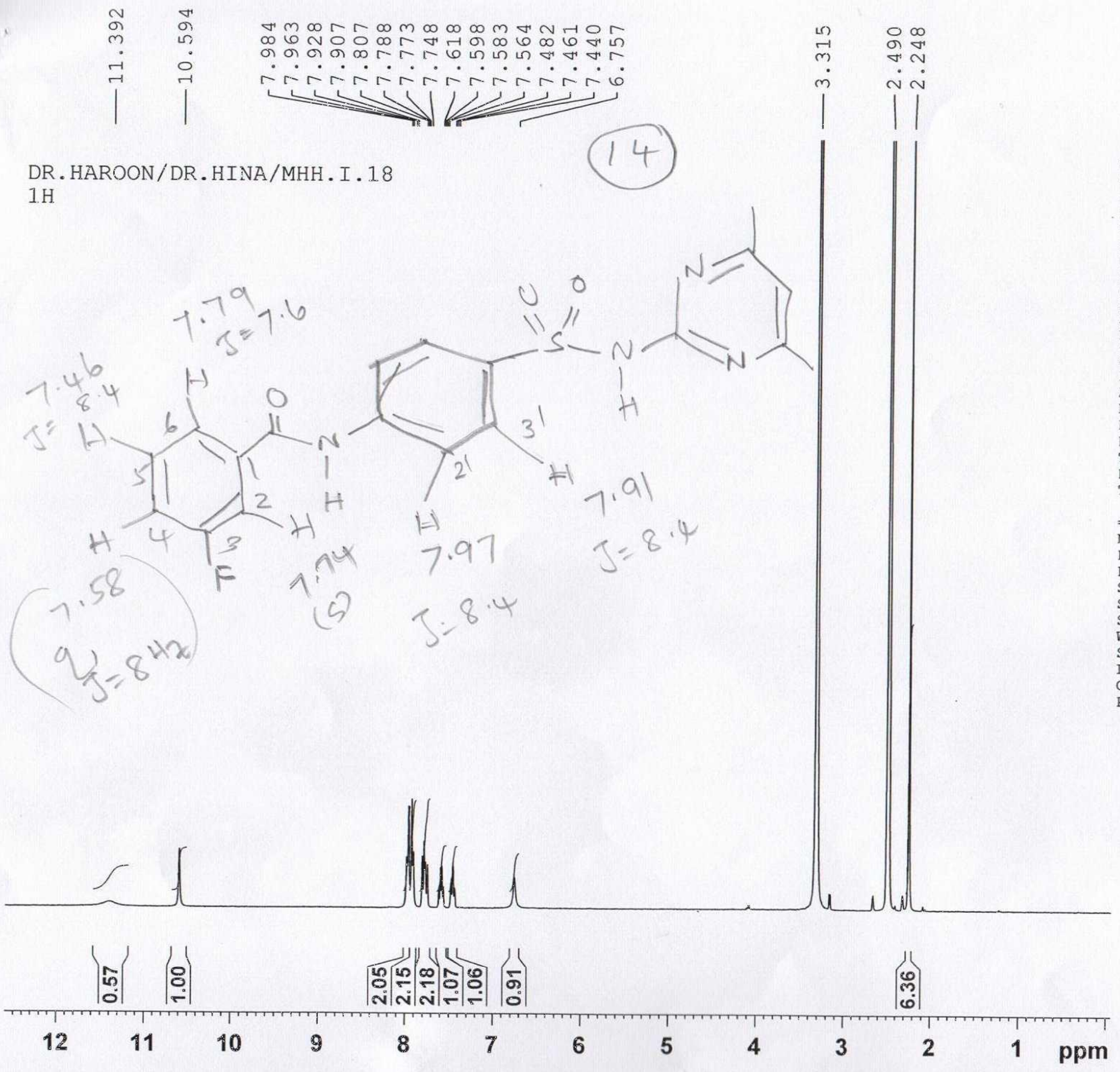

AVANCE AV-400 MHz
Lab # 115

NAME	dec26-16
EXPNO	1
PROCNO	1
Date_	20161226
Time	10.42
INSTRUM	spect
PROBHD	5 mm SEI 1H-13
PULPROG	zg30
TD	65536
SOLVENT	DMSO
NS	128
DS	0
SWH	8012.820 Hz
FIDRES	0.122266 Hz
AQ	4.0894966 sec
RG	512
DW	62.400 usec
DE	6.50 usec
TE	300.0 K
D1	1.50000000 sec
TD0	1

======== CHANNEL f1 ========

NUC1	1H
P1	10.80 usec
PL1	3.00 dB
SFO1	400.0332002 MHz
SI	32768
SF	400.0300041 MHz
WDW	EM
SSB	0
LB	0.30 Hz
GB	0
PC	1.00

(16)

DR.HAROON/DR.HINA/MHH.I.18
1H

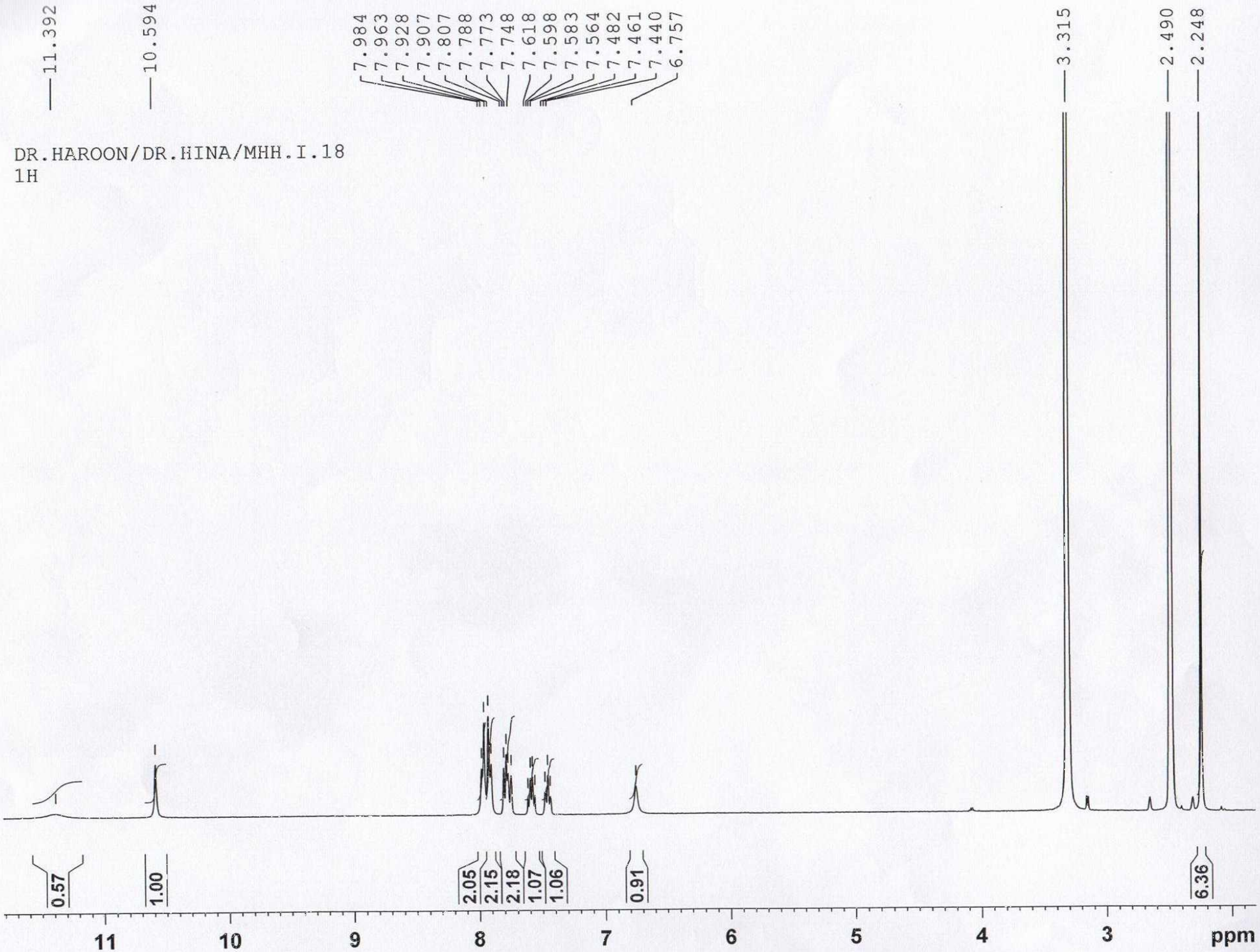

DR.HAROON/DR.HINA/MHH.I.18
1H

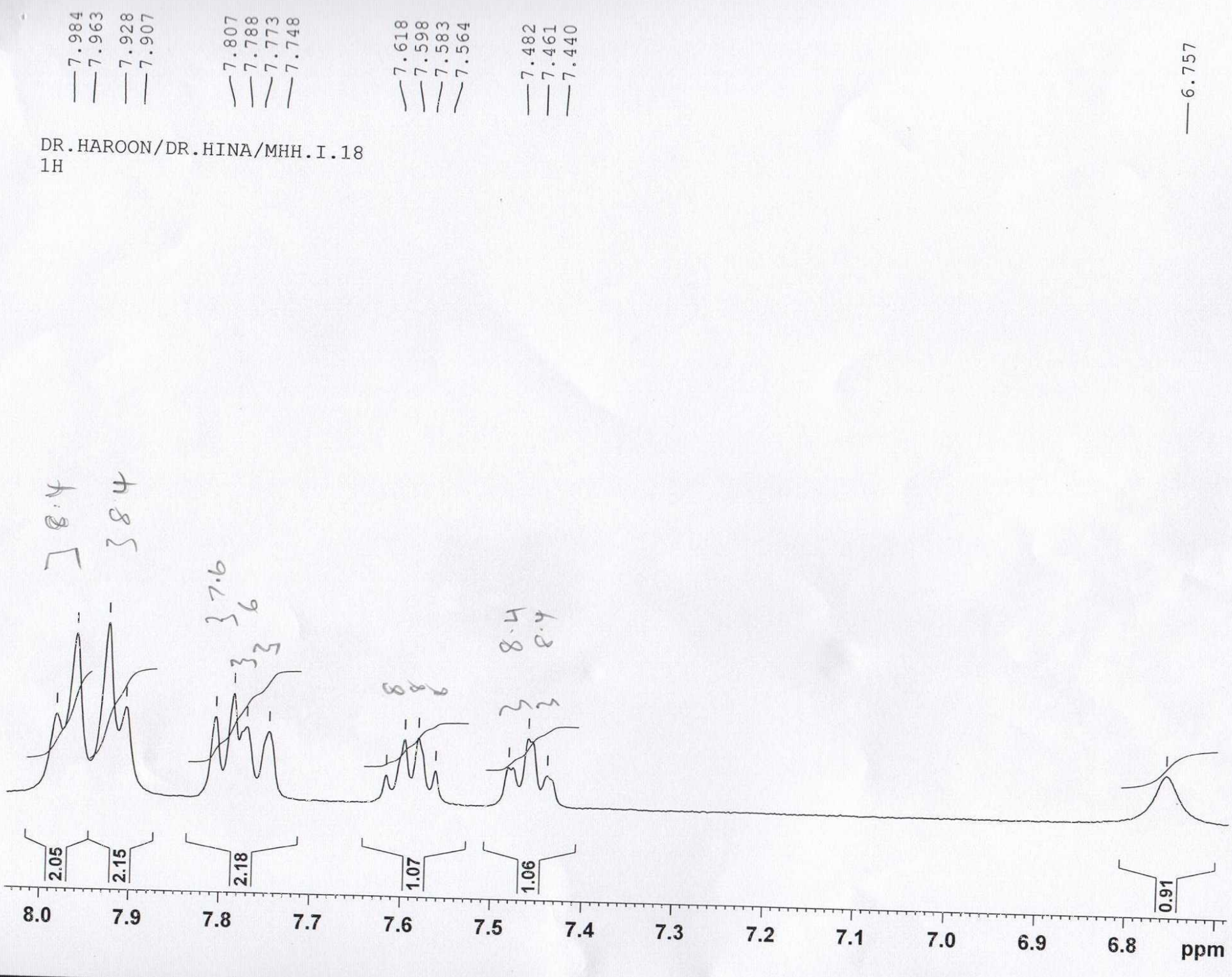

File: MHH-I-18 Date Run: 02-09-2017 (Time Run: 12:20:26)
Sample: DR.M.H.HAROON /DR. HINA
Instrument: JEOL MS 600H-1
Ionization mode: EI+

Scan: 20 R.T.: 1.68
Base: m/z 335; 85.5%FS TIC: 4106090

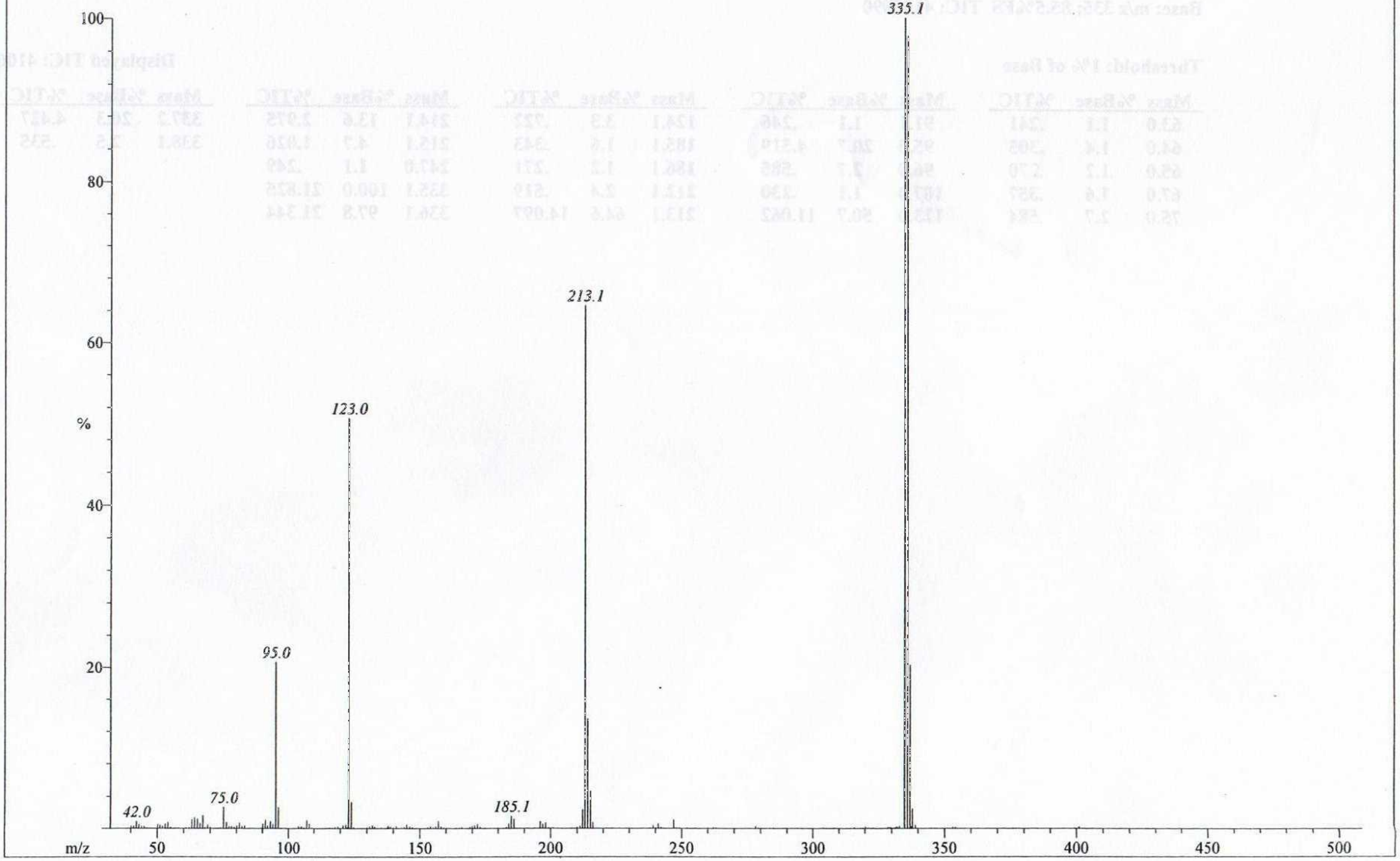

I. C.C.B.S., University of Karachi Analytical Laboratory - Pakistan

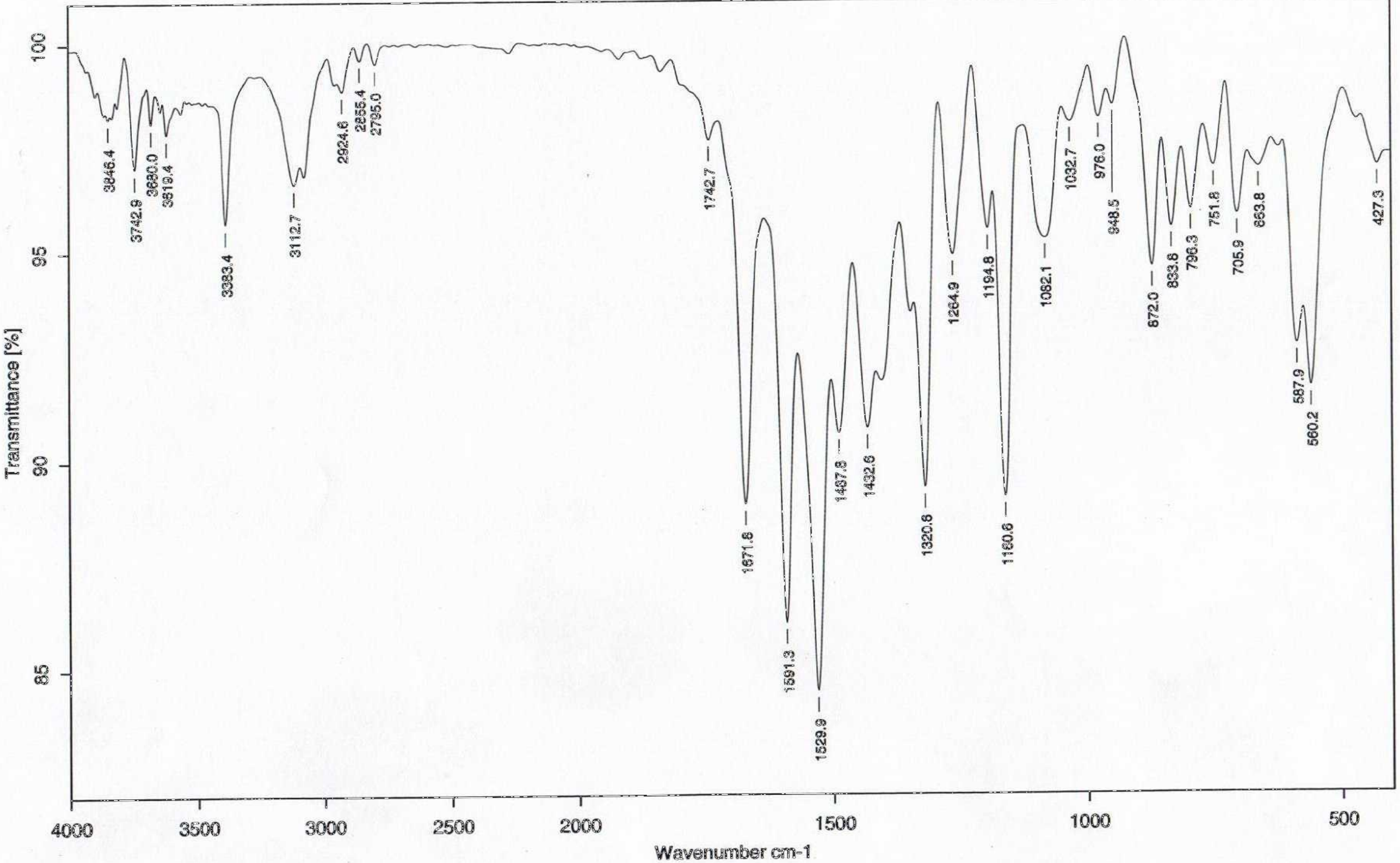

Sample : MHH-1-18/Haroon/Dr. Hina	Spectrum : MHH-1-18.0 (in D:\IRSTUDENT)
Measured : 01/02/2017 on VECTOR22	Technic : Solid
Resolution : 4 cm-1 (10 scans)	**Analyst : ZA/Jamshed/M. Asif/Haroon**

THERMO ELECTRON ~ VISIONpro SOFTWARE V4.10

Operator Name	ARSHAD ALAM.	Date of Report	2/2/2017
Department	Analytical Laboratory TWC # 004	Time of Report	2:53:24PM
Organization	ICCBS Karachi of Universty.		
Information	Dr.Haroon/ Dr.Hina		

Scan Graph

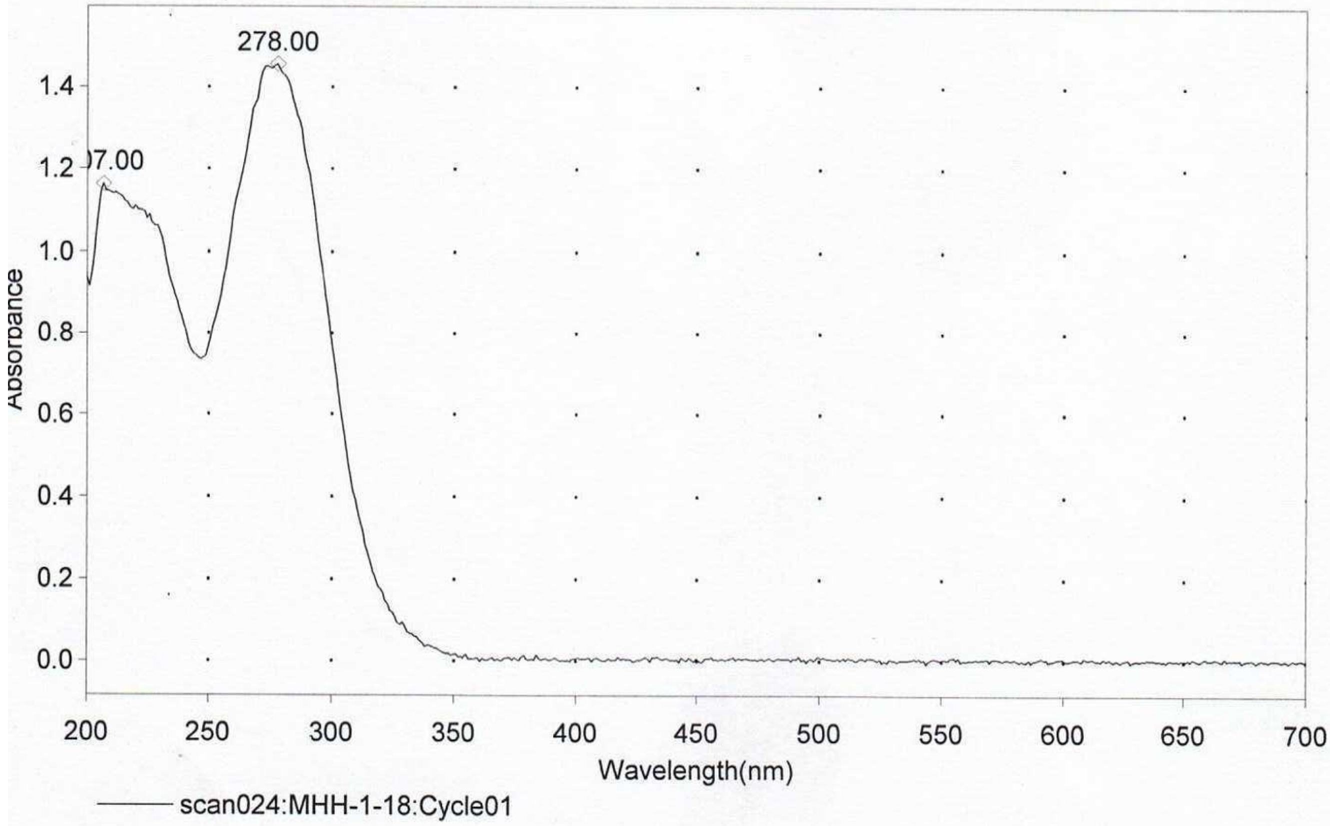

Results Table - MHH-1-18.sre,MHH-1-18,Cycle01

nm	A	**Peak Pick Method**
07.00	1.164	Find 8 Peaks Above -3.0000 A
78.00	1.455	Start Wavelength 200.00 nm
		Stop Wavelength 700.00 nm
		Sort By Wavelength

Sensitivity Auto
