# Supplementary material for: Sulphamethazine derivatives as immunomodulating agents: New therapeutic strategies for inflammatory diseases
Source: PLoS One. 2018 Dec 19;13(12):e0208933. doi: 10.1371/journal.pone.0208933 (PMC6300282; doi:10.1371/journal.pone.0208933)

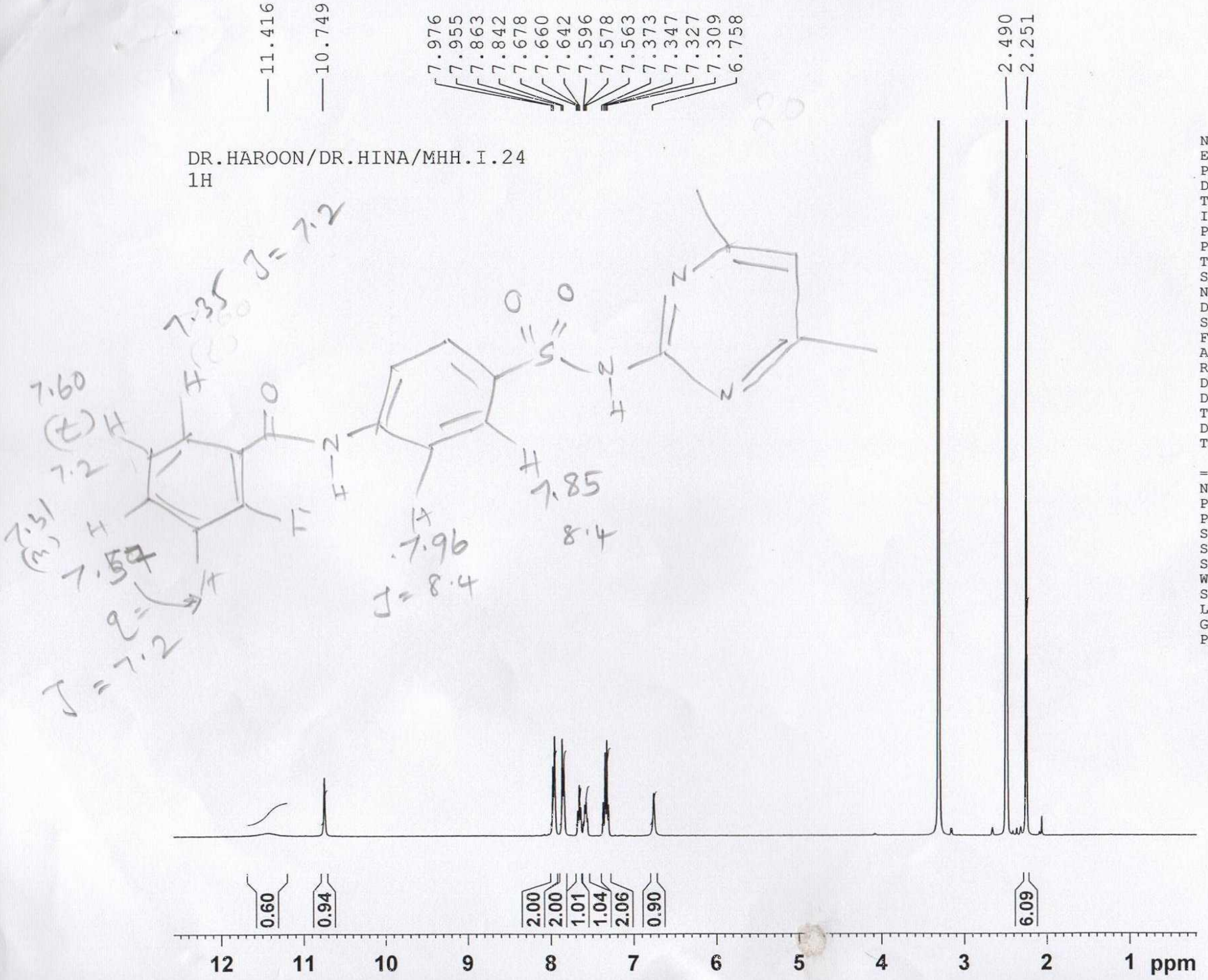

AVANCE AV-400 MHz
Lab # 115

(17)

NAME	jan02-17
EXPNO	3
PROCNO	1
Date_	20170102
Time	11.18
INSTRUM	spect
PROBHD	5 mm SEI 1H-13
PULPROG	zg30
TD	65536
SOLVENT	DMSO
NS	64
DS	0
SWH	8012.820 Hz
FIDRES	0.122266 Hz
AQ	4.0894966 sec
RG	812.7
DW	62.400 usec
DE	6.50 usec
TE	300.0 K
D1	2.00000000 sec
TD0	1

======== CHANNEL f1 ========

NUC1	1H
P1	10.80 usec
PL1	3.00 dB
SFO1	400.0332002 MHz
SI	32768
SF	400.0300041 MHz
WDW	EM
SSB	0
LB	0.30 Hz
GB	0
PC	1.00

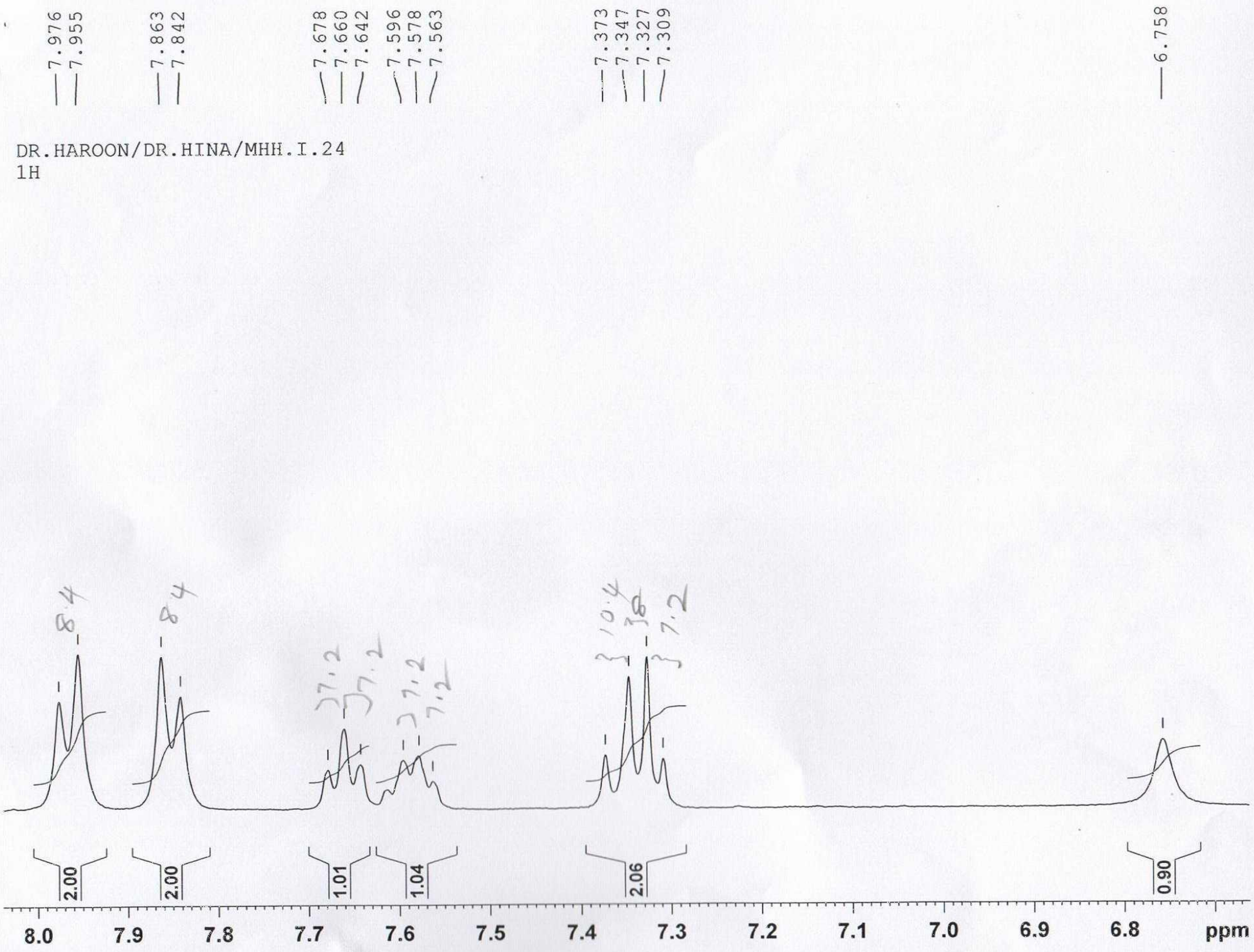
7.976
7.955
7.863
7.842
7.678
7.660
7.642
7.596
7.578
7.563
7.373
7.347
7.327
7.309
6.758
DR.HAROON/DR.HINA/MHH.I.24
1H
2.00
2.00
1.01
1.04
2.06
0.90
8.0
7.9
7.8
7.7
7.6
7.5
7.4
7.3
7.2
7.1
7.0
6.9
6.8
ppm

2/4/2017 3:07:50 PM

File: MHH-I-24
Sample: DR.MH.HAROON /DR. HINA
Instrument: JEOL MS 600H-1

Date Run: 02-04-2017 (Time Run: 14:57:18)

Ionization mode: EI+

Scan: 15
Base: m/z 336; 26.4%FS TIC: 1800146

R.T.: 1.25

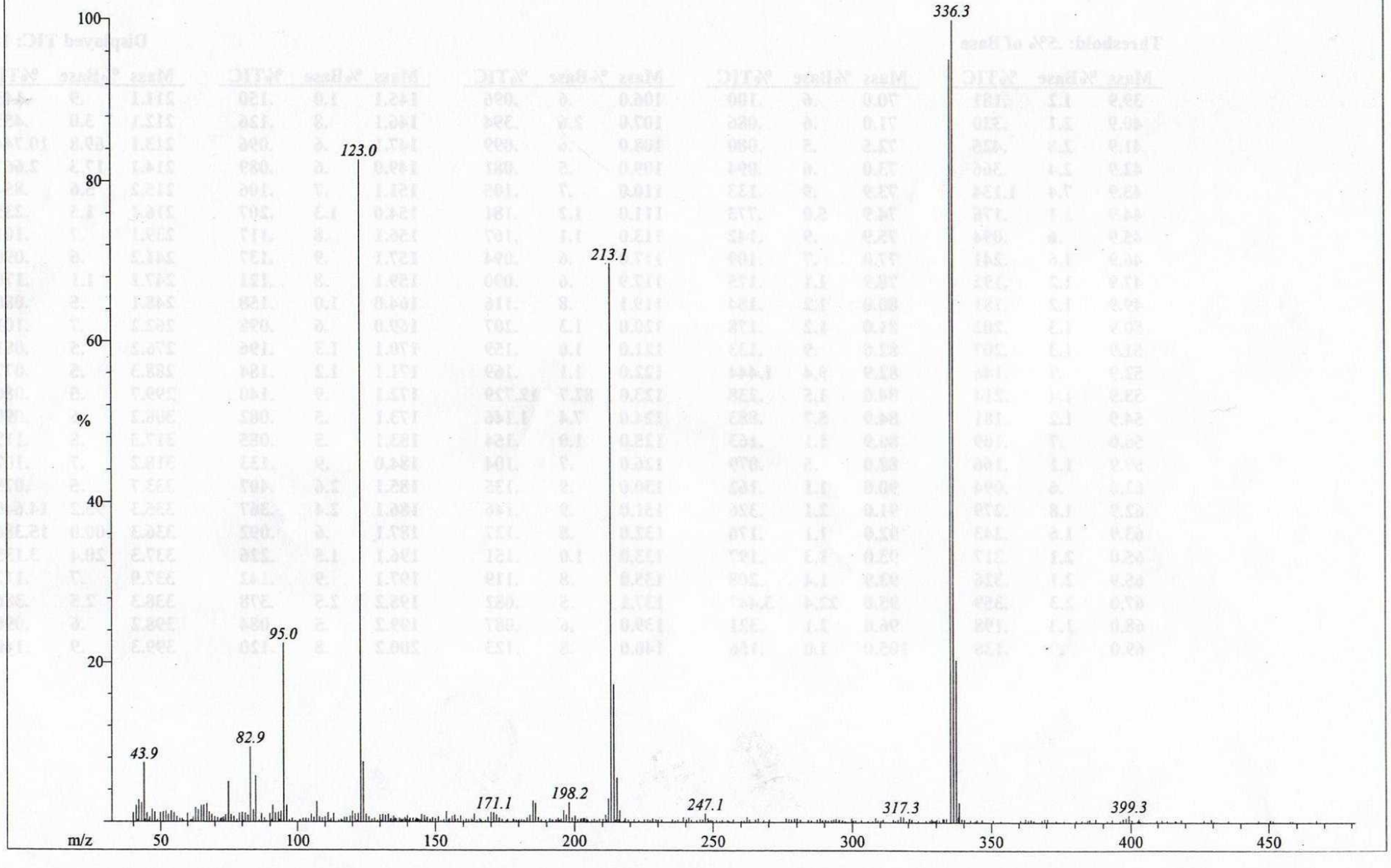

Supplement: S15 Fig — (PDF) [file pone.0208933.s015.pdf]
