# Supplementary material for: Sulphamethazine derivatives as immunomodulating agents: New therapeutic strategies for inflammatory diseases
Source: PLoS One. 2018 Dec 19;13(12):e0208933. doi: 10.1371/journal.pone.0208933 (PMC6300282; doi:10.1371/journal.pone.0208933)
Supplement: S16 Fig — (PDF) [file pone.0208933.s016.pdf]

DR.HAROON/DR.HINA/MHH.I.16
1H

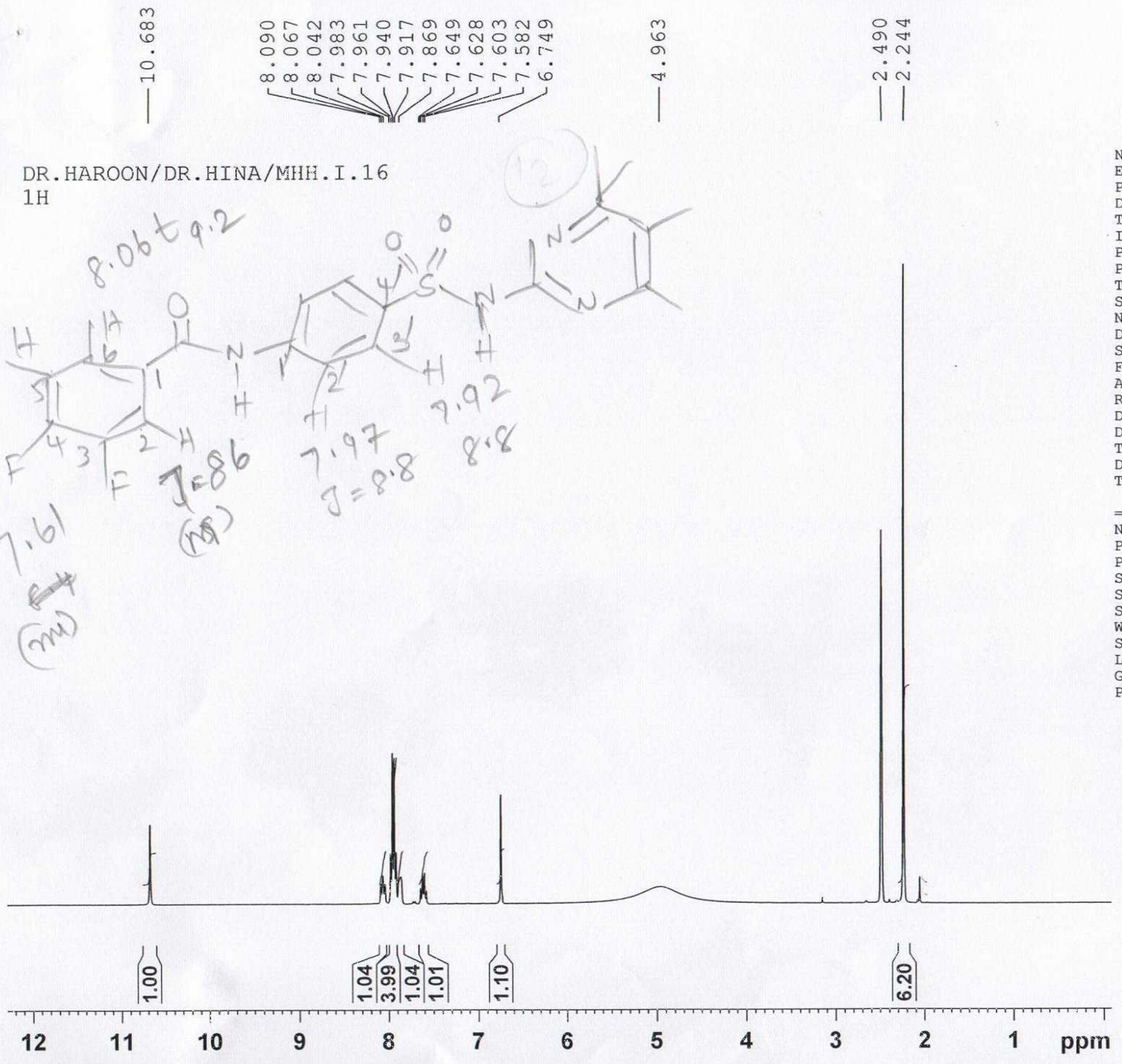

AVANCE AV-400 MHz
Lab # 115

NAME	jan02-17
EXPNO	2
PROCNO	1
Date_	20170102
Time	11.06
INSTRUM	spect
PROBHD	5 mm SEI 1H-13
PULPROG	zg30
TD	65536
SOLVENT	DMSO
NS	64
DS	0
SWH	8012.820 Hz
FIDRES	0.122266 Hz
AQ	4.0894966 sec
RG	512
DW	62.400 usec
DE	6.50 usec
TE	300.0 K
D1	2.00000000 sec
TD0	1

======== CHANNEL f1 ========

NUC1	1H
P1	10.80 usec
PL1	3.00 dB
SFO1	400.0332002 MHz
SI	32768
SF	400.0300041 MHz
WDW	EM
SSB	0
LB	0.30 Hz
GB	0
PC	1.00

(18)

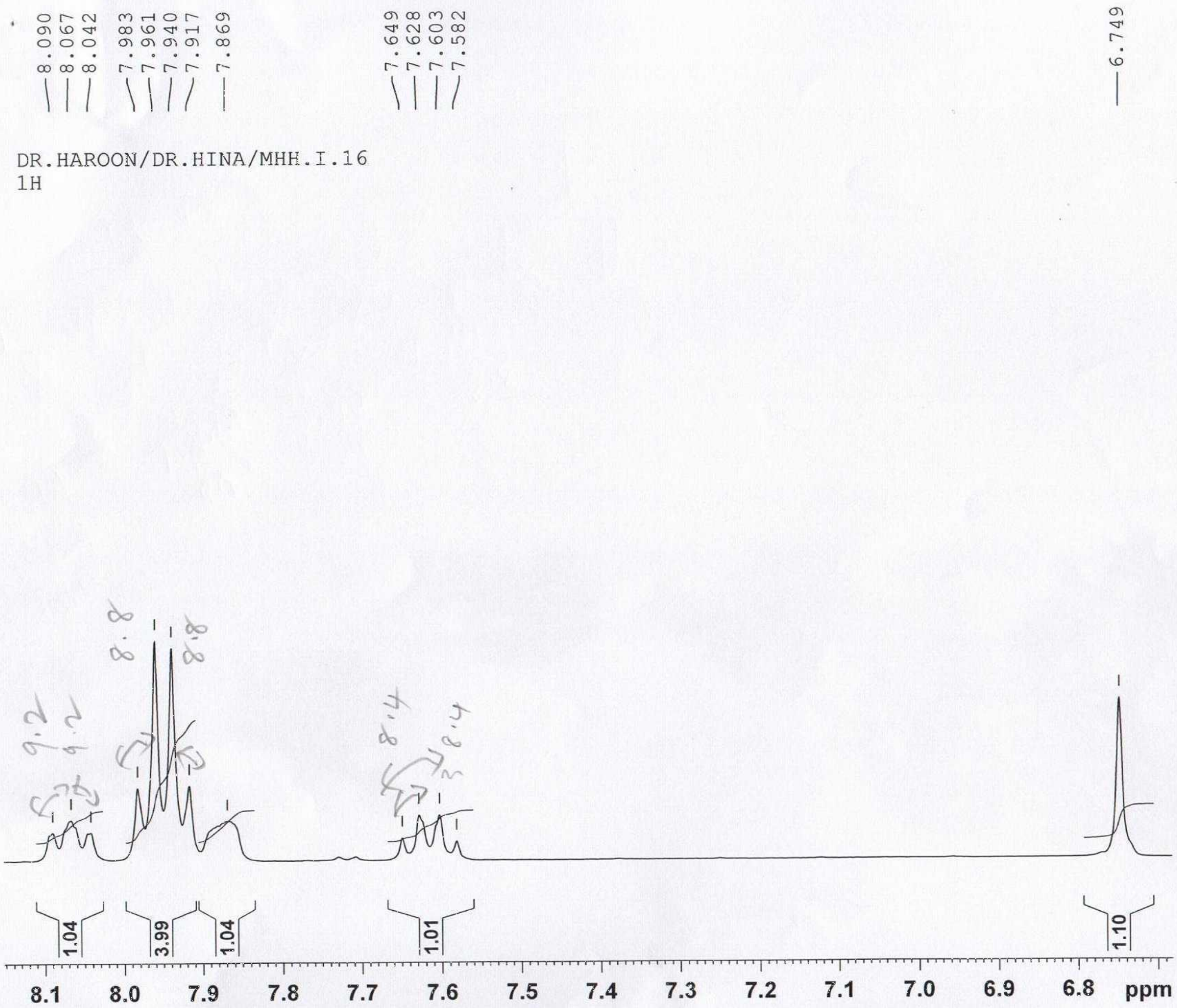
8.090
8.067
8.042
7.983
7.961
7.940
7.917
7.869
7.649
7.628
7.603
7.582
6.749
DR.HAROON/DR.HINA/MHH.I.16
1H
1.04
3.99
1.04
1.01
1.10
8.1
8.0
7.9
7.8
7.7
7.6
7.5
7.4
7.3
7.2
7.1
7.0
6.9
6.8
ppm

HEJ-ICCBS
2/7/2017 3:21:55 PM

File: MHH-I-16
Sample: DR.M.H.HAROON /DR. HINA
Instrument: JEOL MS 600H-1

Date Run: 02-07-2017 (Time Run: 15:15:06)

Ionization mode: EI+

Scan: 12
Base: m/z 353; 90.8%FS TIC: 4430270

R.T.: .98

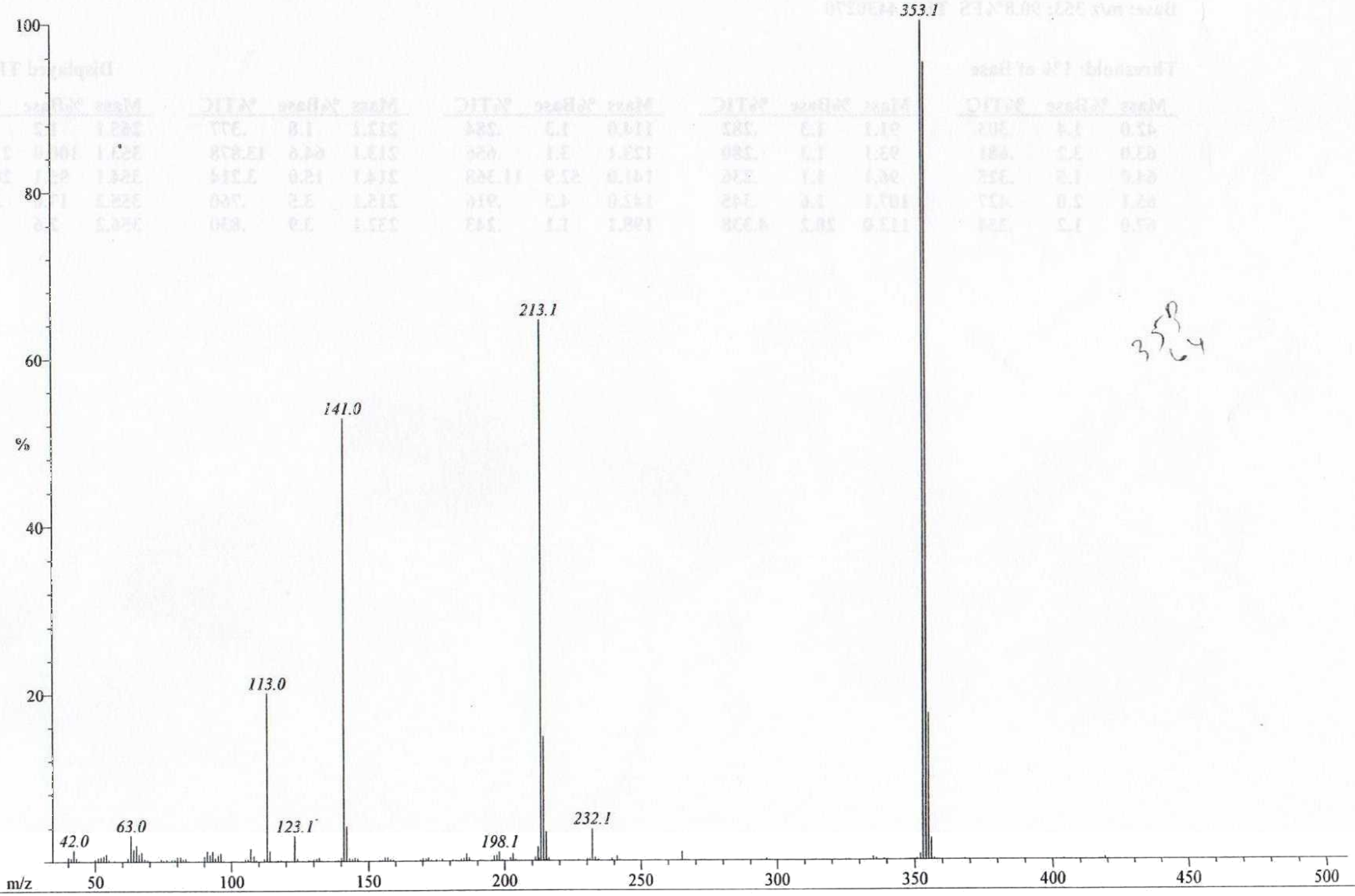

I. C.C.B.S., University of Karachi Analytical Laboratory - Pakistan

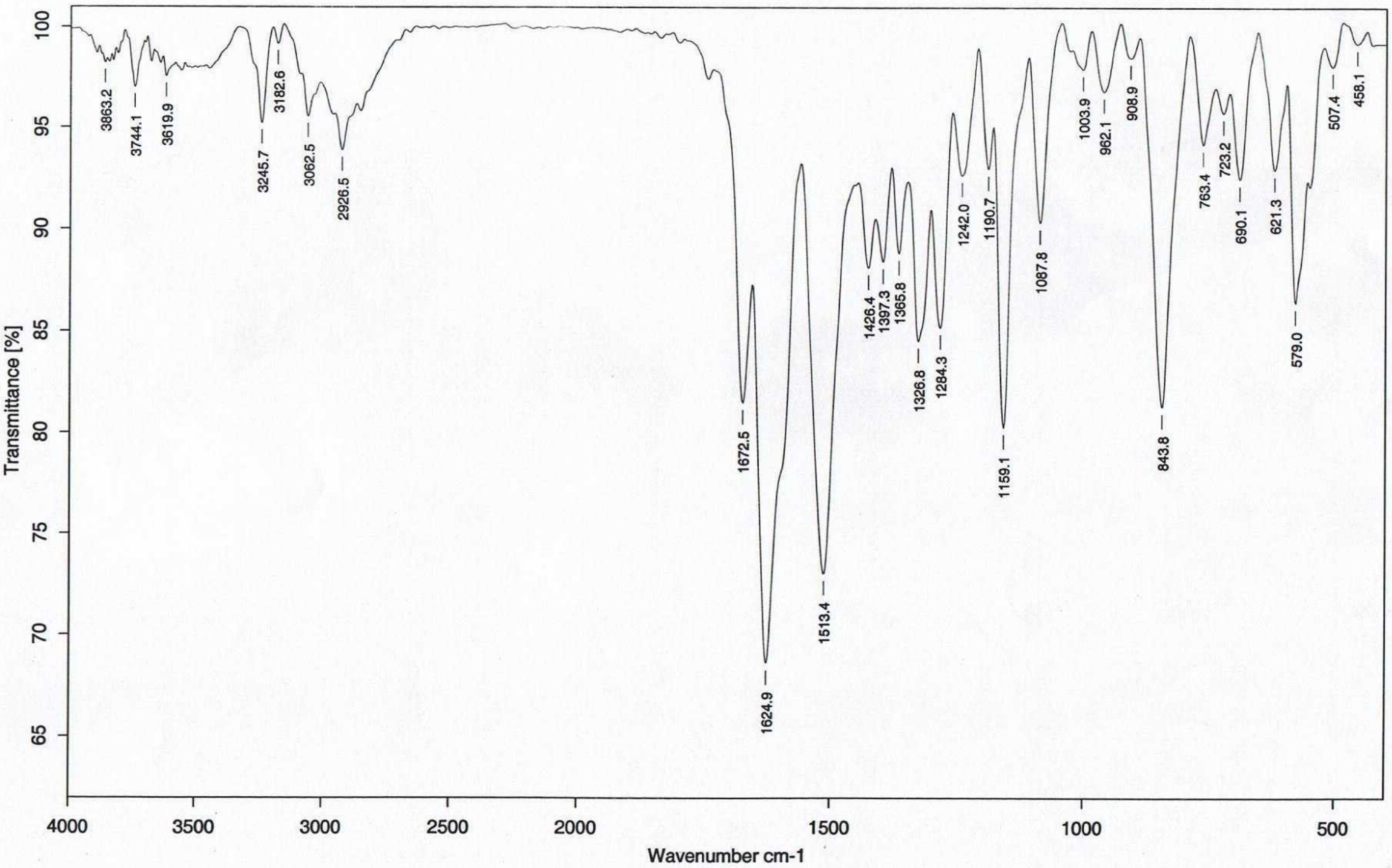

Sample : MHH-1-16/Haroon/Dr. Hina	Spectrum : MHH-1-16.0 (in D:\IRSTUDENT)
Measured : 01/02/2017 on VECTOR22	Technic : Solid
Resolution : 4 cm-1 (10 scans)	Analyst : ZA/Jamshed/M. Asif/Haroon

THERMO ELECTRON ~ VISIONpro SOFTWARE V4.10

Operator Name	ARSHAD ALAM.	Date of Report	2/2/2017
Department	Analytical Laboratory TWC # 004	Time of Report	2:46:20PM
Organization	ICCBS Karachi of Universty.		
nformation	Dr.Haroon/ Dr.Hina		

Scan Graph

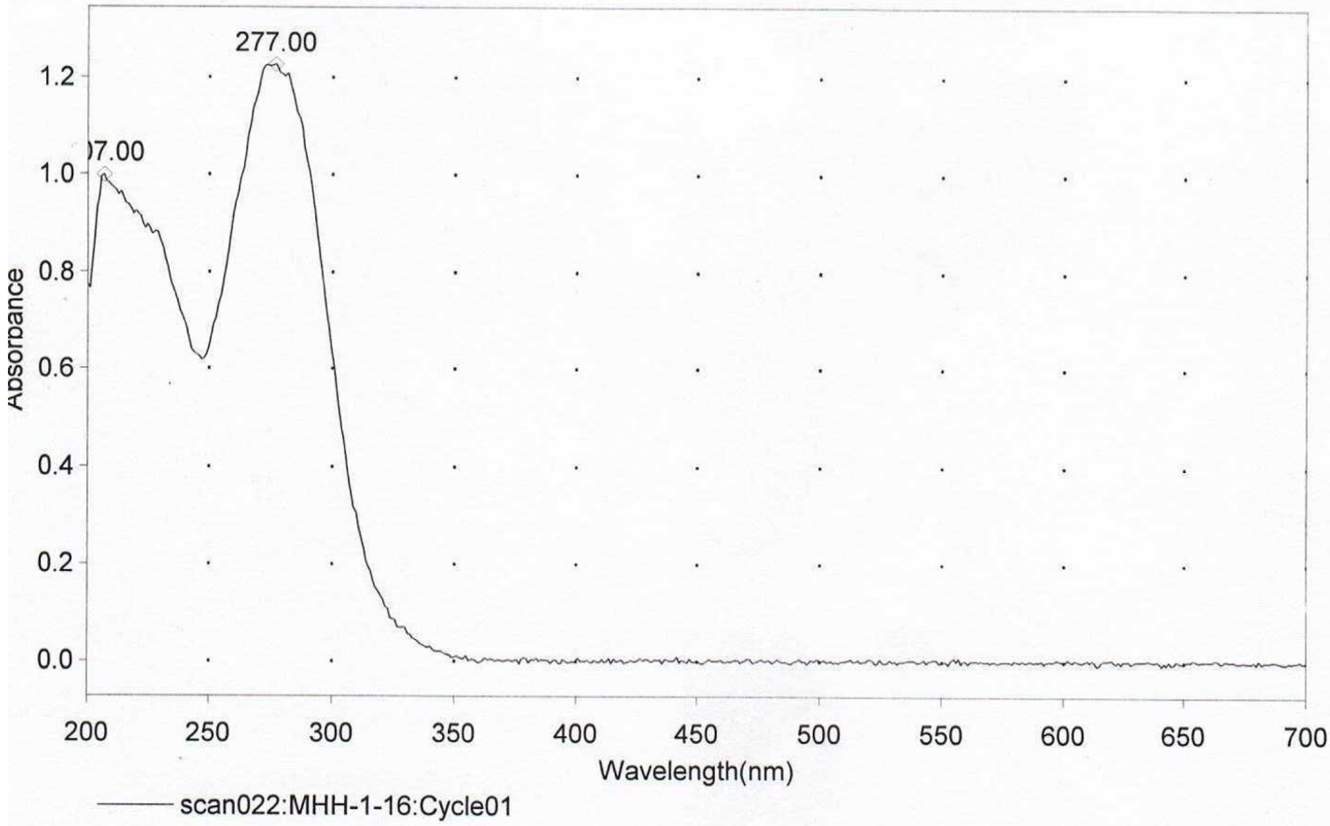

Results Table - MHH-1-16.sre,MHH-1-16,Cycle01

m	A	**Peak Pick Method**
07.00	0.999	Find 8 Peaks Above -3.0000 A
77.00	1.227	Start Wavelength 200.00 nm
		Stop Wavelength 700.00 nm
		Sort By Wavelength

Sensitivity Auto
