# Supplementary material for: Sulphamethazine derivatives as immunomodulating agents: New therapeutic strategies for inflammatory diseases
Source: PLoS One. 2018 Dec 19;13(12):e0208933. doi: 10.1371/journal.pone.0208933 (PMC6300282; doi:10.1371/journal.pone.0208933)
Supplement: S18 Fig — (PDF) [file pone.0208933.s018.pdf]

AVANCE AV-400 MHz
Lab # 115

DR.HAROON/DR.HINA/MHH.I.30
1H

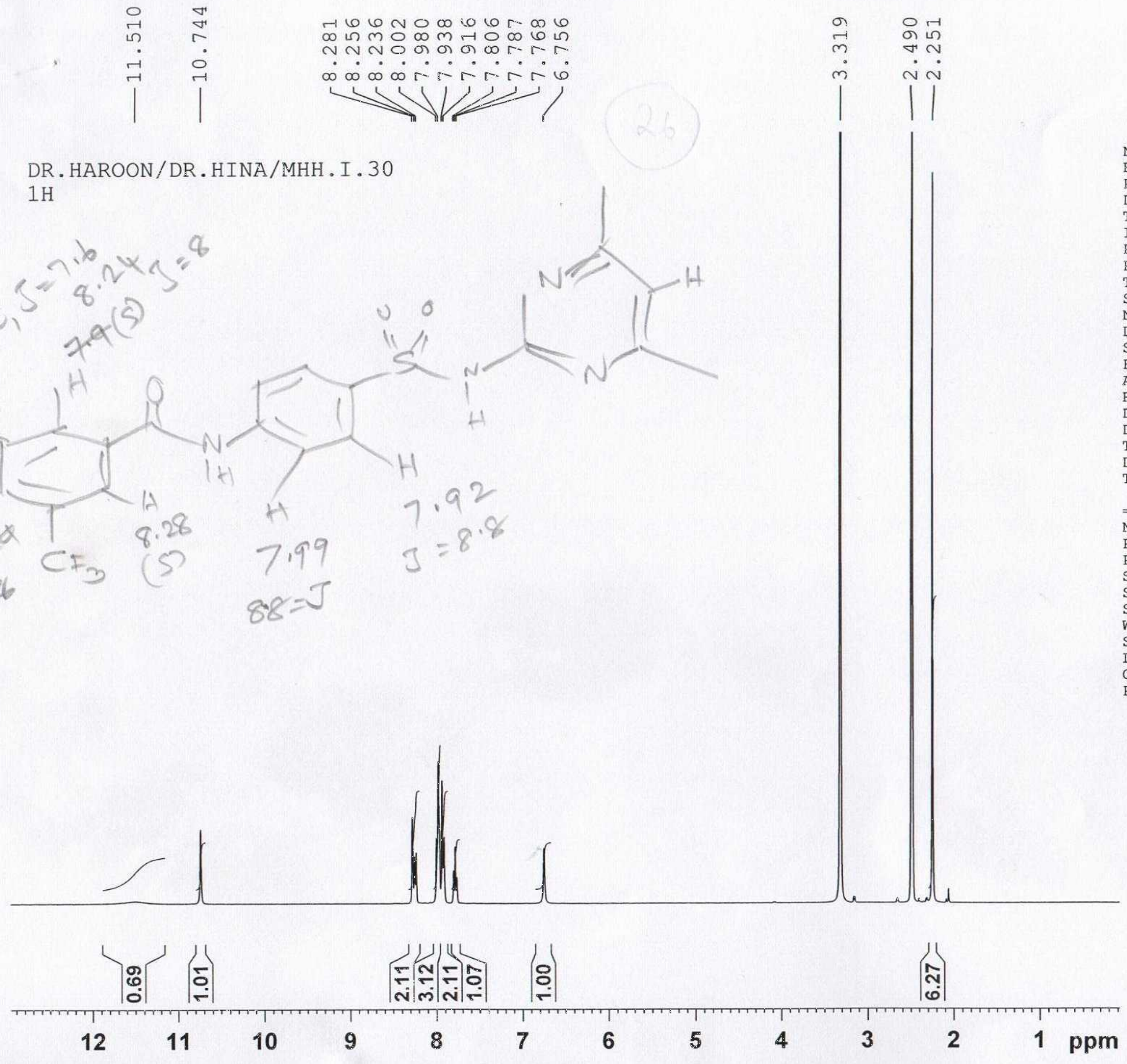


| | |
|---|---|
| NAME | jan03-17 |
| EXPNO | 1 |
| PROCNO | 1 |
| Date_ | 20170103 |
| Time | 10.37 |
| INSTRUM | spect |
| PROBHD | 5 mm SEI 1H-13 |
| PULPROG | zg30 |
| TD | 65536 |
| SOLVENT | DMSO |
| NS | 64 |
| DS | 0 |
| SWH | 8012.820 Hz |
| FIDRES | 0.122266 Hz |
| AQ | 4.0894966 sec |
| RG | 512 |
| DW | 62.400 usec |
| DE | 6.50 usec |
| TE | 300.0 K |
| D1 | 2.00000000 sec |
| TD0 | 1 |

======== CHANNEL f1 ========

| | |
|---|---|
| NUC1 | 1H |
| P1 | 10.80 usec |
| PL1 | 3.00 dB |
| SFO1 | 400.0332002 MHz |
| SI | 32768 |
| SF | 400.0300041 MHz |
| WDW | EM |
| SSB | 0 |
| LB | 0.30 Hz |
| GB | 0 |
| PC | 1.00 |

DR.HAROON/DR.HINA/MHH.I.30
1H

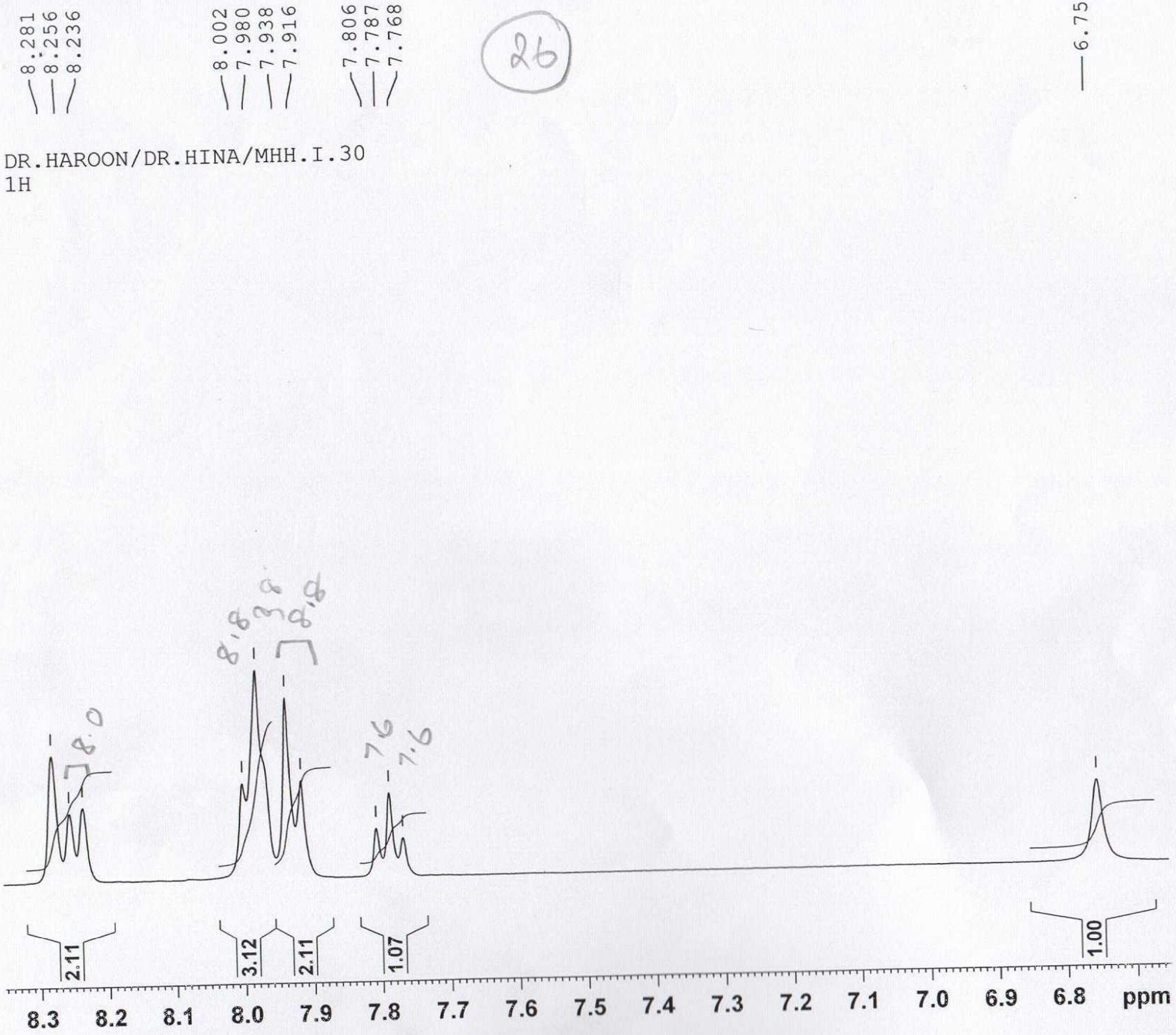

2/10/2017 9:43:01 AM

File: MHH-I-30
Sample: DR.M.H.HAROON /DR. HINA
Instrument: JEOL MS 600H-1

Date Run: 02-10-2017 (Time Run: 09:28:38)

Ionization mode: EI+

Scan: 21
Base: m/z 385; 40.3%FS TIC: 2222474

R.T.: 1.77

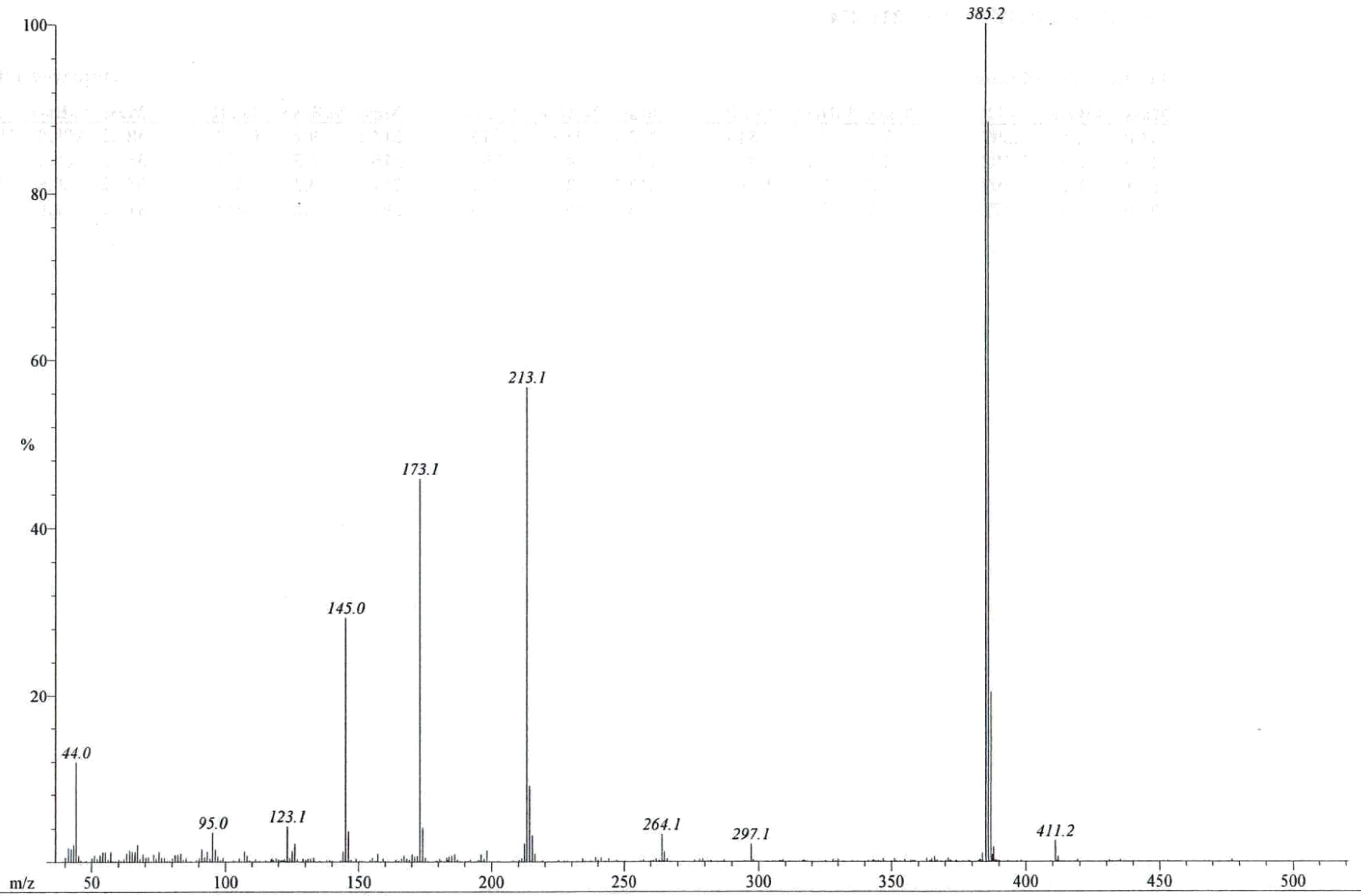

I. C.C.B.S., University of Karachi Analytical Laboratory - Pakistan

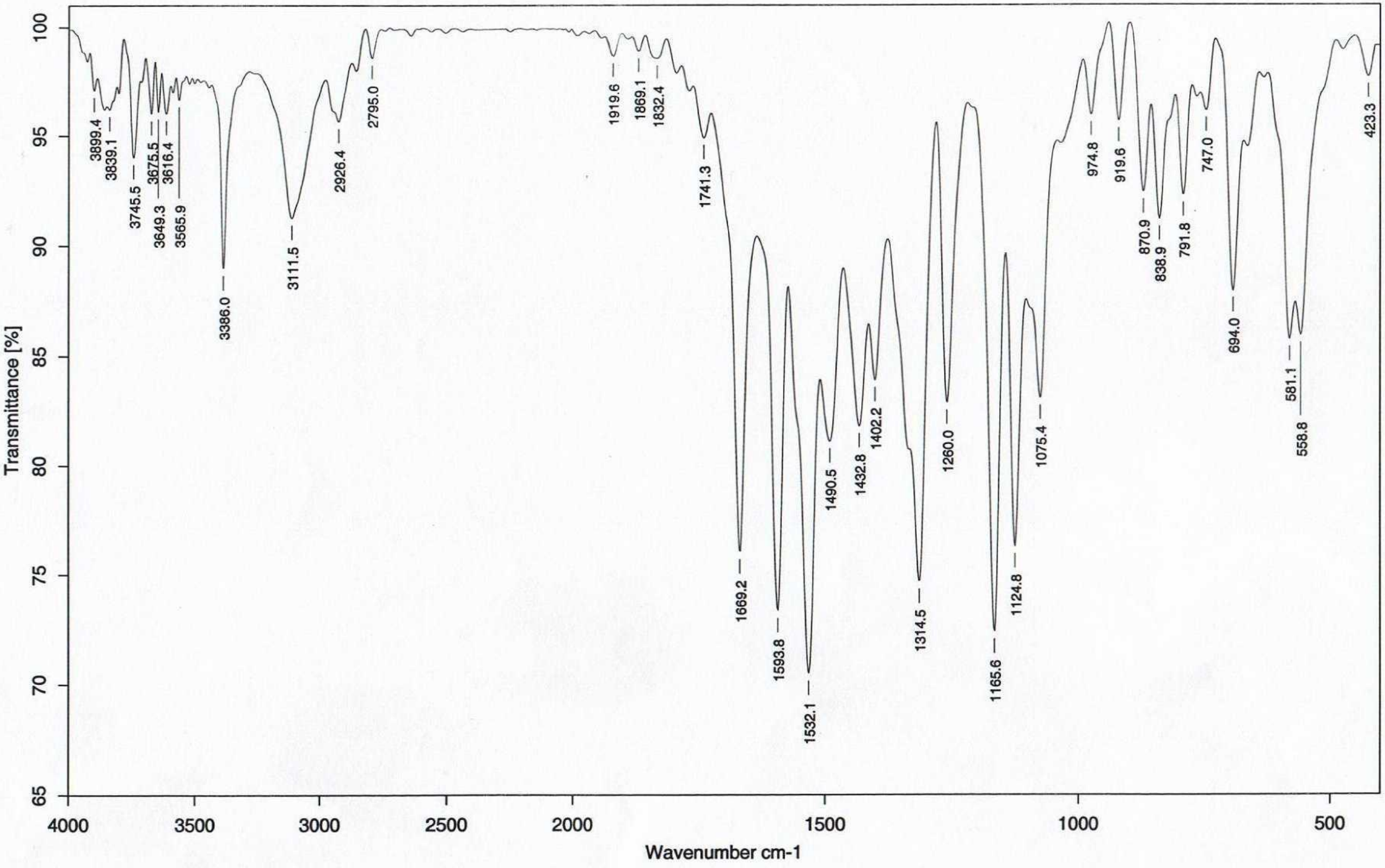


Sample : MHH-1-30/Dr.Haroon

Measured : 27/01/2017 on VECTOR22

Resolution : 4 cm-1 ( 10 scans )

Spectrum : MHH-1-30.0 ( in D:\IRSTUDENT)

Technic : Liquid

Analyst : M. Asif

# THERMO ELECTRON ~ VISIONpro SOFTWARE V4.10

| | | | |
|---|---|---|---|
| Operator Name | ARSHAD ALAM. | Date of Report | 1/30/2017 |
| Department | Analytical Laboratory TWC # 004 | Time of Report | 10:34:22AM |
| Organization | ICCBS Karachi of Universty. | | |
| Information | Dr Haron/Dr Hina | | |

**Scan Graph**

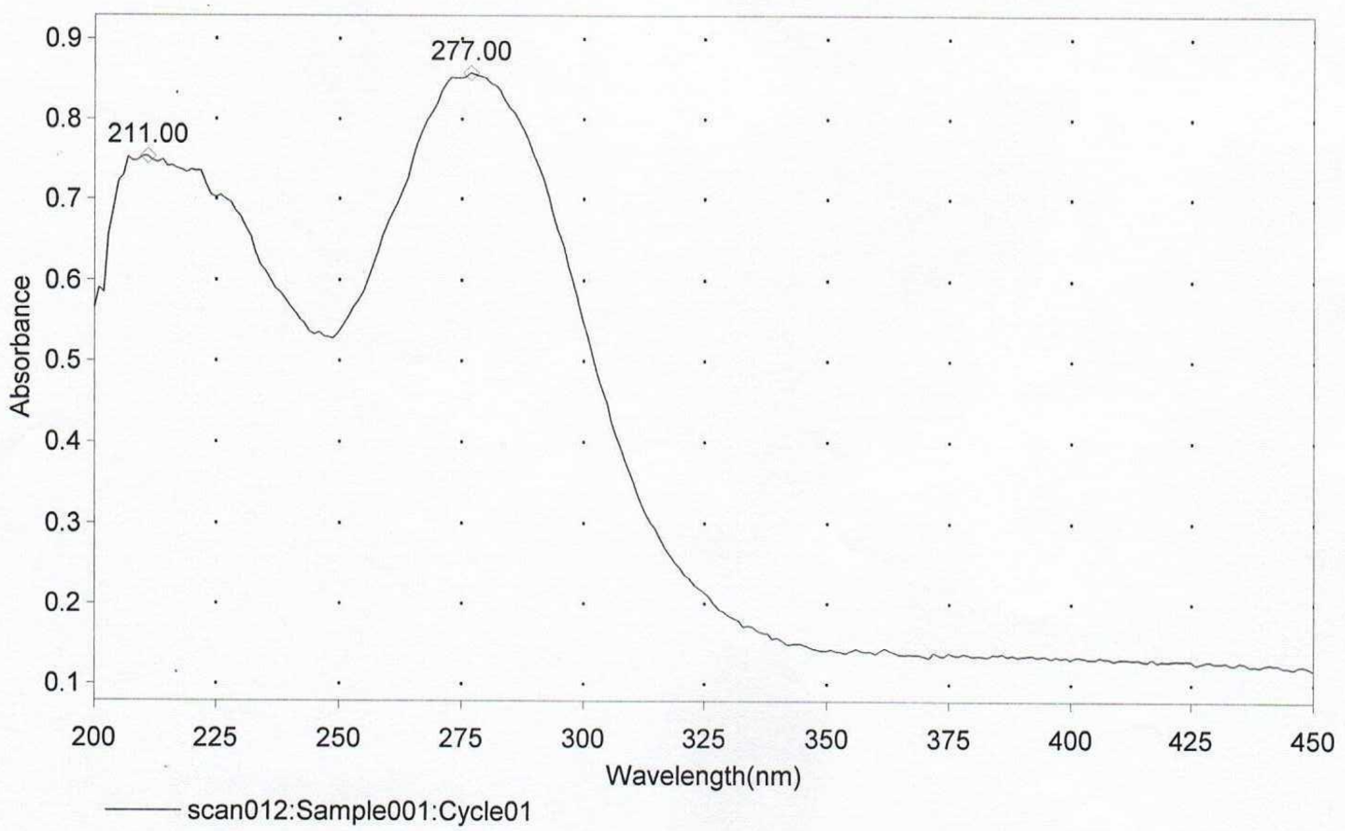


**Results Table - MHH-1-30.sre,Sample001,Cycle01**

| nm | A | **Peak Pick Method** |
|---|---|---|
| 211.00 | 0.753 | Find 8 Peaks Above -3.0000 A |
| 277.00 | 0.857 | Start Wavelength 200.00 nm |
| | | Stop Wavelength 450.00 nm |
| | | Sort By Wavelength |

Sensitivity Auto
