# Supplementary material for: Sulphamethazine derivatives as immunomodulating agents: New therapeutic strategies for inflammatory diseases
Source: PLoS One. 2018 Dec 19;13(12):e0208933. doi: 10.1371/journal.pone.0208933 (PMC6300282; doi:10.1371/journal.pone.0208933)
Supplement: S19 Fig — (PDF) [file pone.0208933.s019.pdf]

AVANCE AV-400 MHz
Lab # 115

DR.HAROON/DR.HINA/MHH.I.12
1H

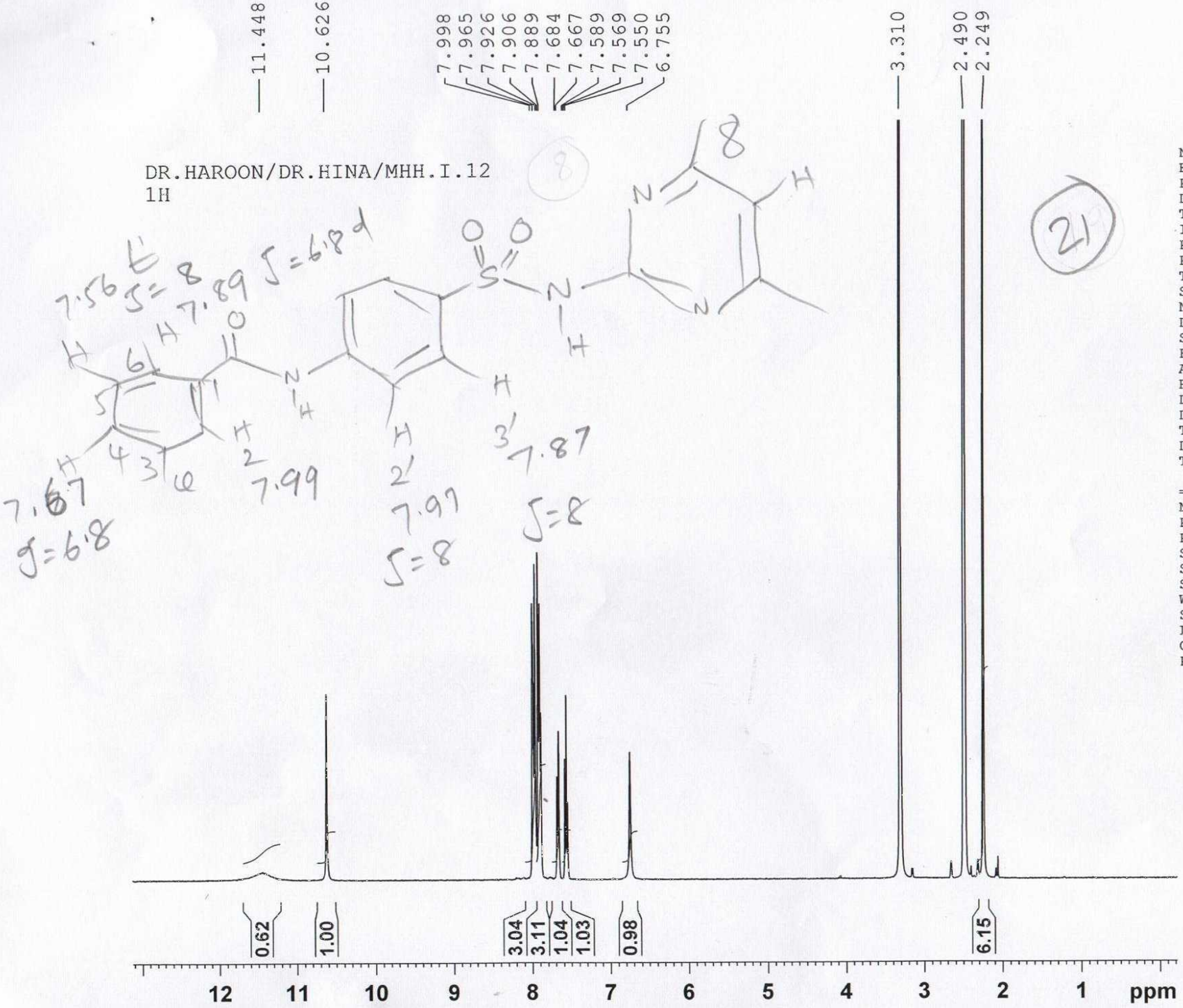

NAME	dec22-16
EXPNO	4
PROCNO	1
Date_	20161222
Time	11.32
INSTRUM	spect
PROBHD	5 mm SEI 1H-13
PULPROG	zg30
TD	65536
SOLVENT	DMSO
NS	64
DS	0
SWH	8012.820 Hz
FIDRES	0.122266 Hz
AQ	4.0894966 sec
RG	574.7
DW	62.400 usec
DE	6.50 usec
TE	300.0 K
D1	2.00000000 sec
TD0	1

======== CHANNEL f1 ========

NUC1	1H
P1	10.80 usec
PL1	3.00 dB
SFO1	400.0332002 MHz
SI	32768
SF	400.0300041 MHz
WDW	EM
SSB	0
LB	0.30 Hz
GB	0
PC	1.00

DR.HAROON/DR.HINA/MHH.I.12
1H

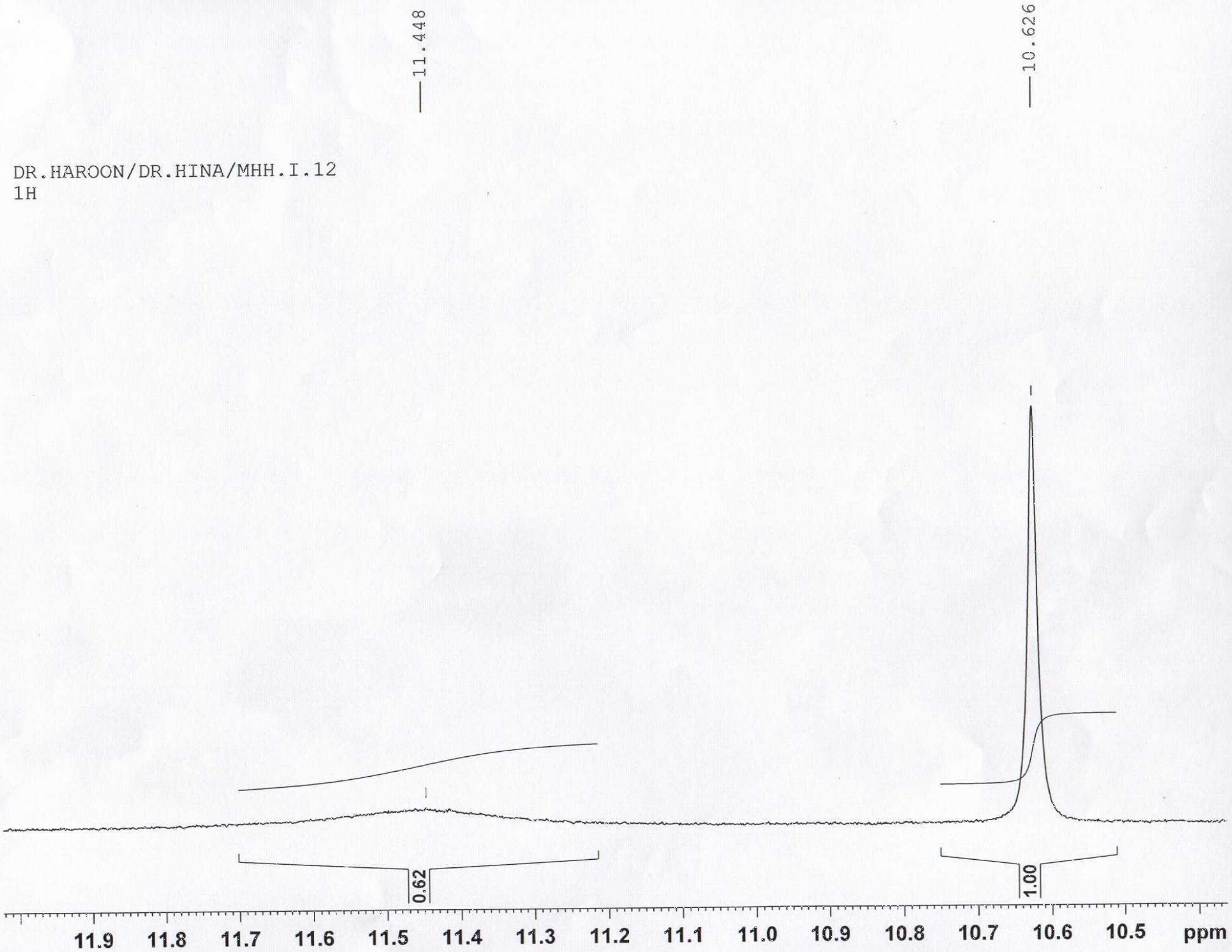

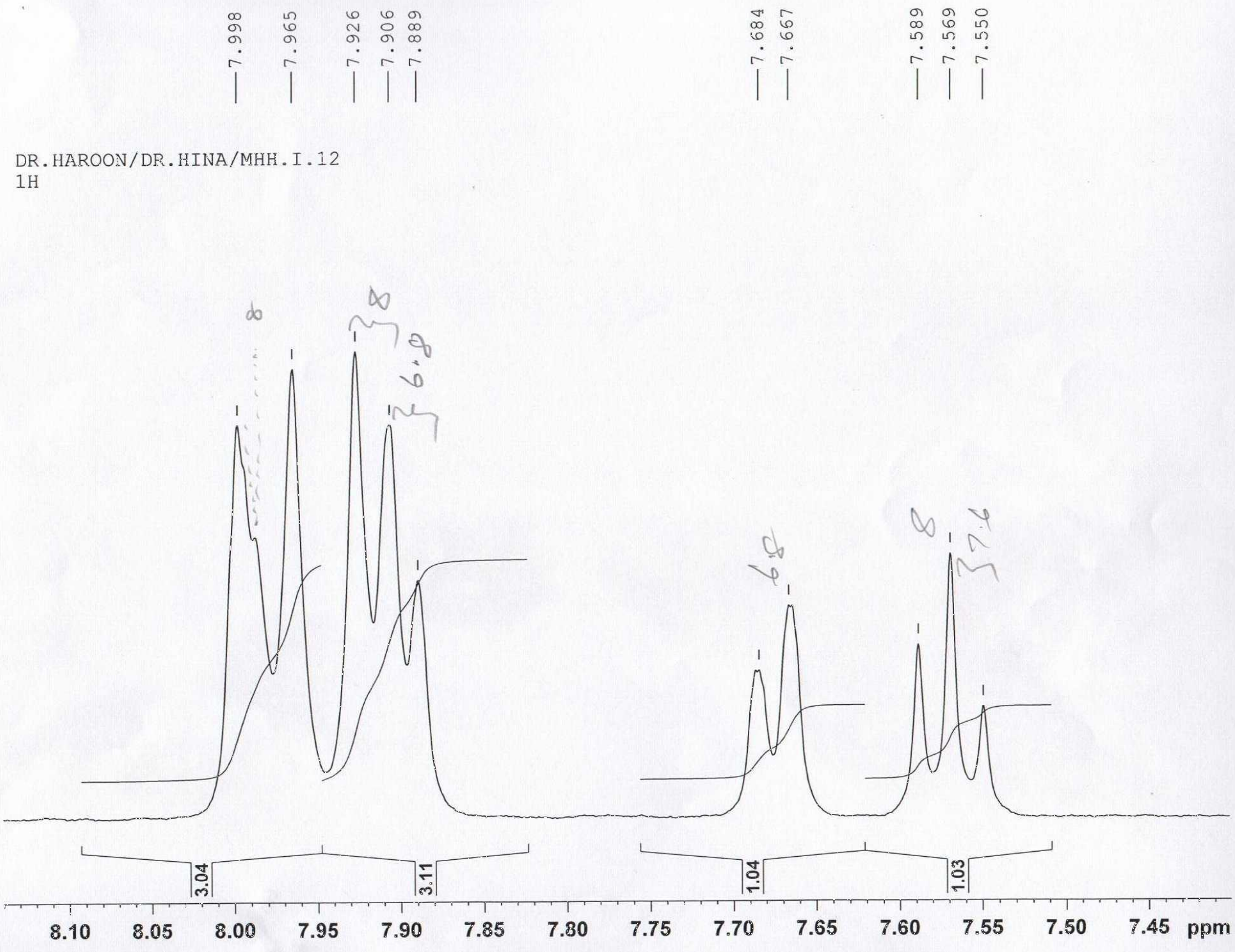

7.998
7.965
7.926
7.906
7.889
7.684
7.667
7.589
7.569
7.550
DR.HAROON/DR.HINA/MHH.I.12
1H
3.04
3.11
1.04
1.03
8.10
8.05
8.00
7.95
7.90
7.85
7.80
7.75
7.70
7.65
7.60
7.55
7.50
7.45
ppm

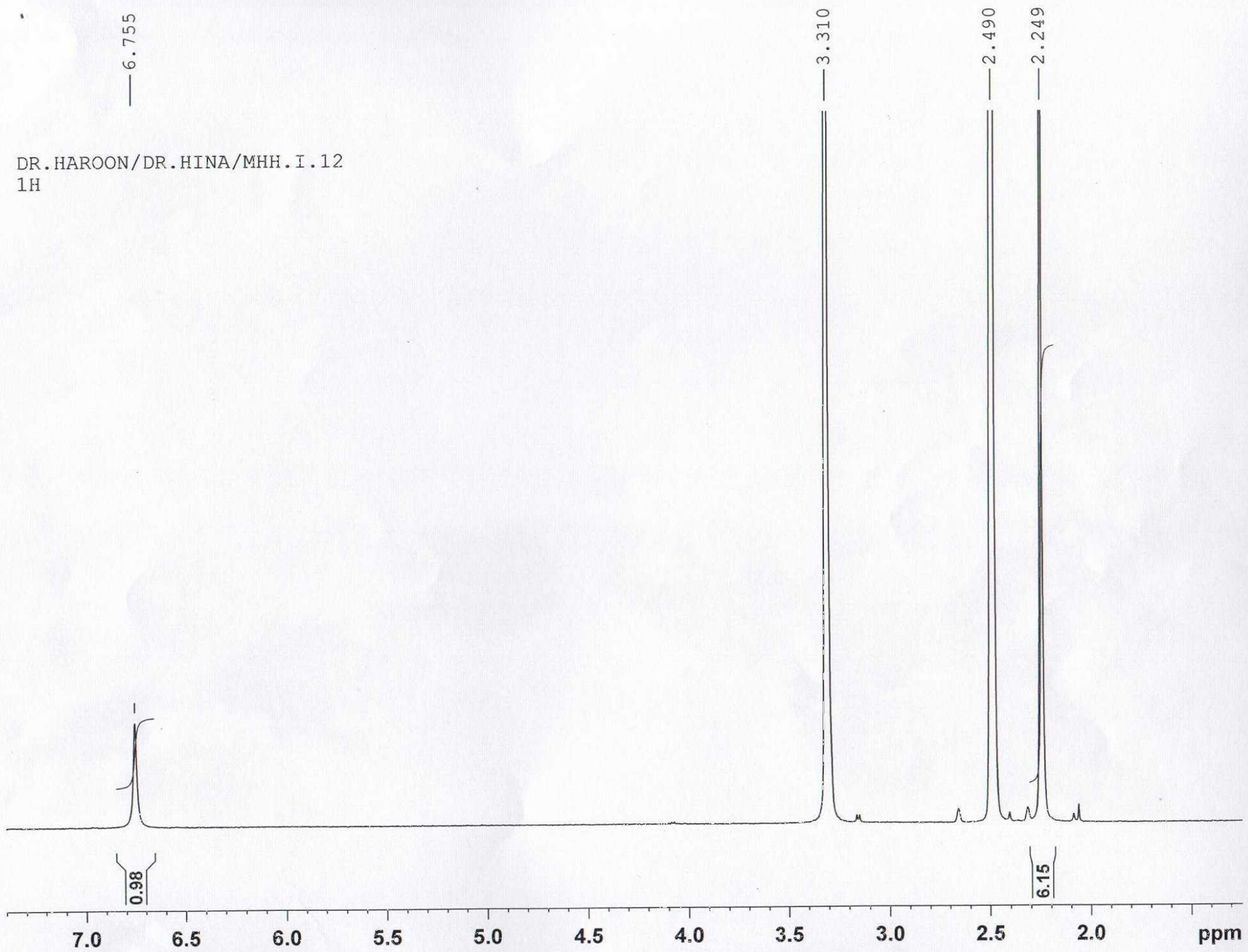

6.755
3.310
2.490
2.249
DR.HAROON/DR.HINA/MHH.I.12
1H
0.98
6.15
7.0
6.5
6.0
5.5
5.0
4.5
4.0
3.5
3.0
2.5
2.0
ppm

File: MHH-I-12 **Date Run: 02-08-2017 (Time Run: 14:45:47)**
Sample: DR. M.H.HAROON /DR. HINA
Instrument: JEOL MS 600H-1
Ionization mode: EI+

Scan: 26 **R.T.: 2.22**
Base: m/z 352; 87.3%FS TIC: 5194922

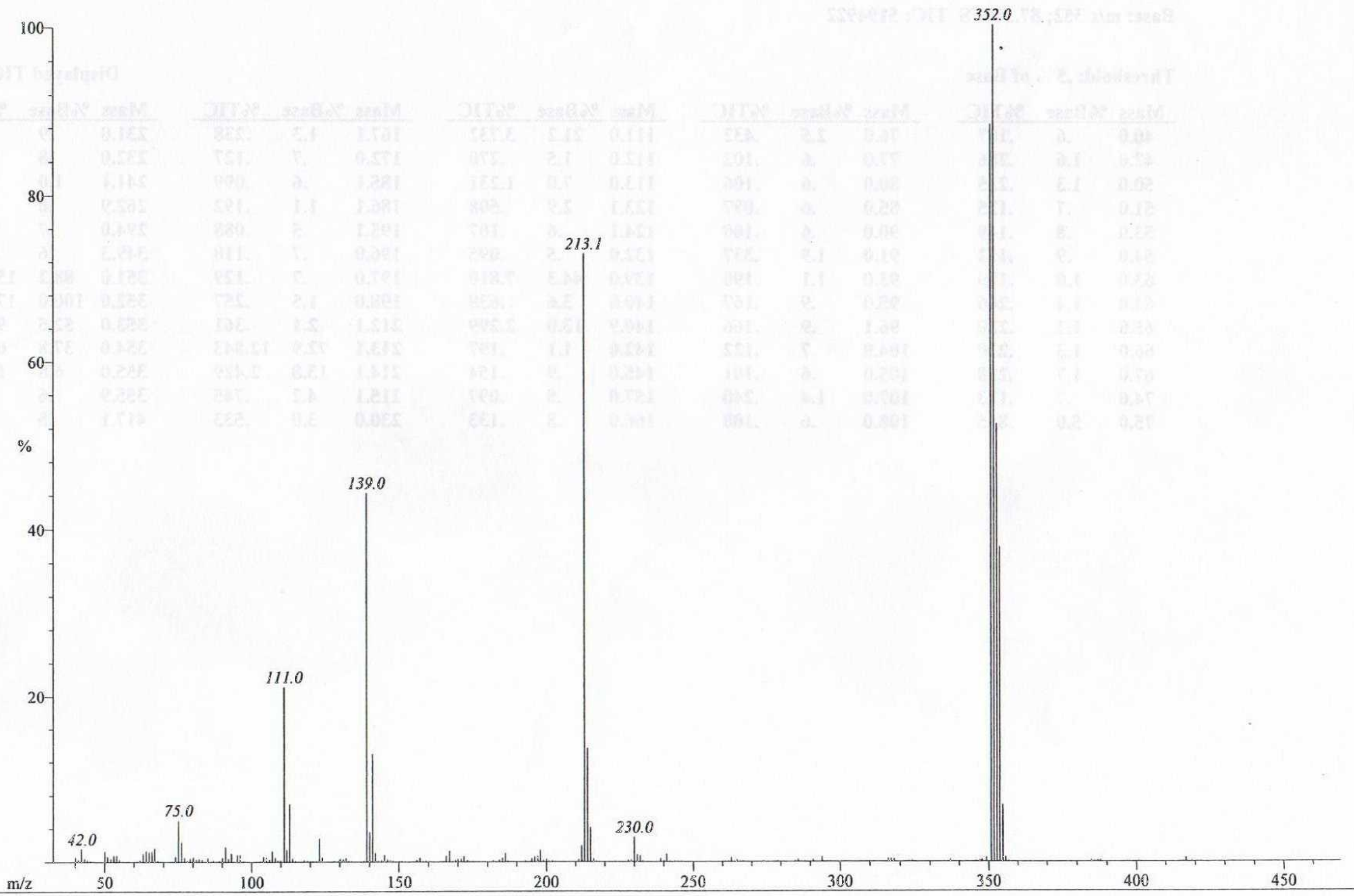

I. C.C.B.S., University of Karachi Analytical Laboratory - Pakistan

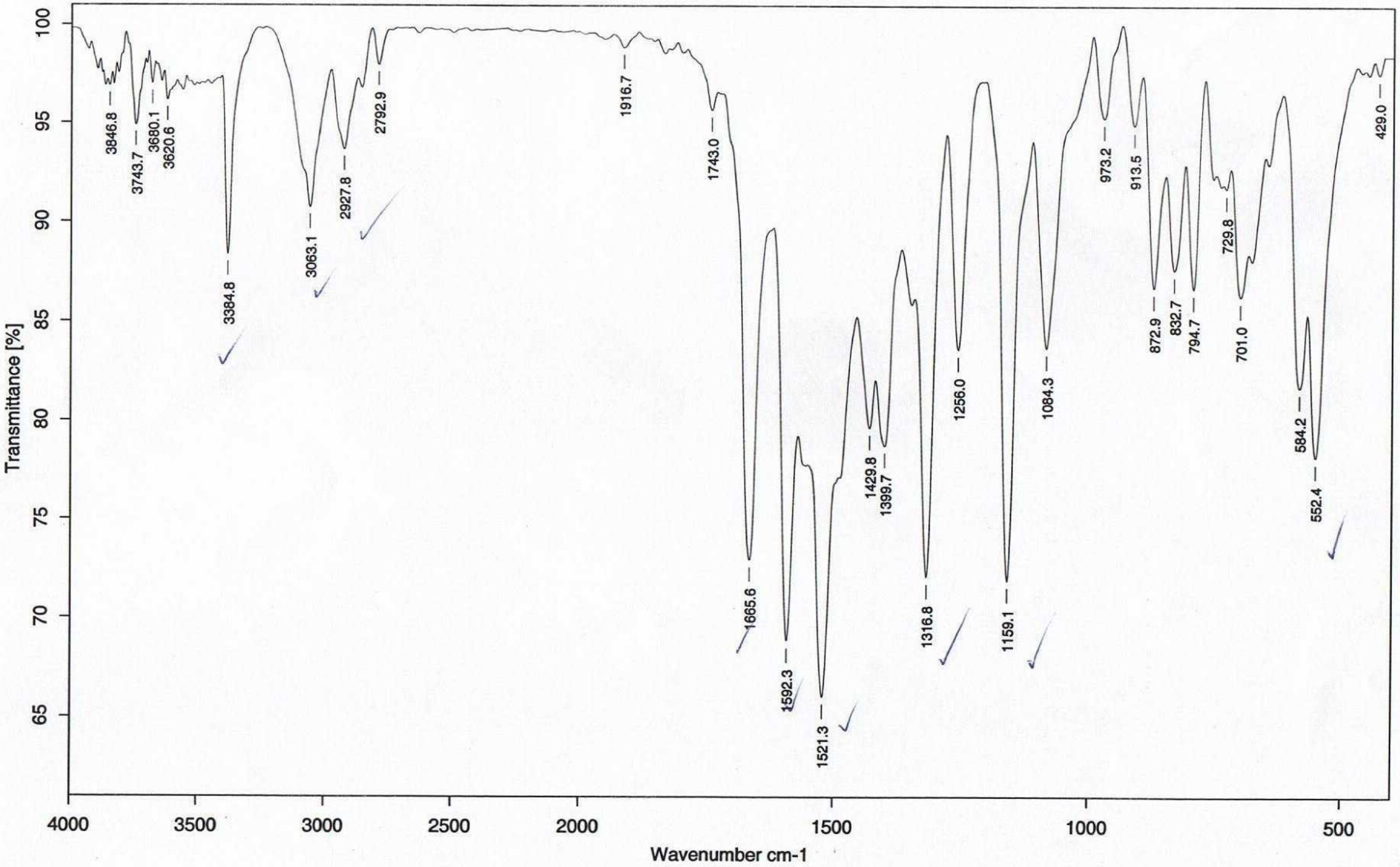

Sample : MHH-1-12/Haroon/Dr. Hina

Measured : 31/01/2017 on VECTOR22

Resolution : 4 cm-1 (10 scans)

Spectrum : MHH-1-12.0 (in D:\IRSTUDENT)

Technic : Solid

Analyst : Zubair Ahmad/ Jamshed/M. Asif/

THERMO ELECTRON ~ VISIONpro SOFTWARE V4.10

Operator Name	ARSHAD ALAM.	Date of Report	2/1/2017
Department	Analytical Laboratory TWC # 004	Time of Report	4:04:23PM
Organization	ICCBS Karachi of Universty.		
Information	Dr Haroon/Dr Hina		

Scan Graph

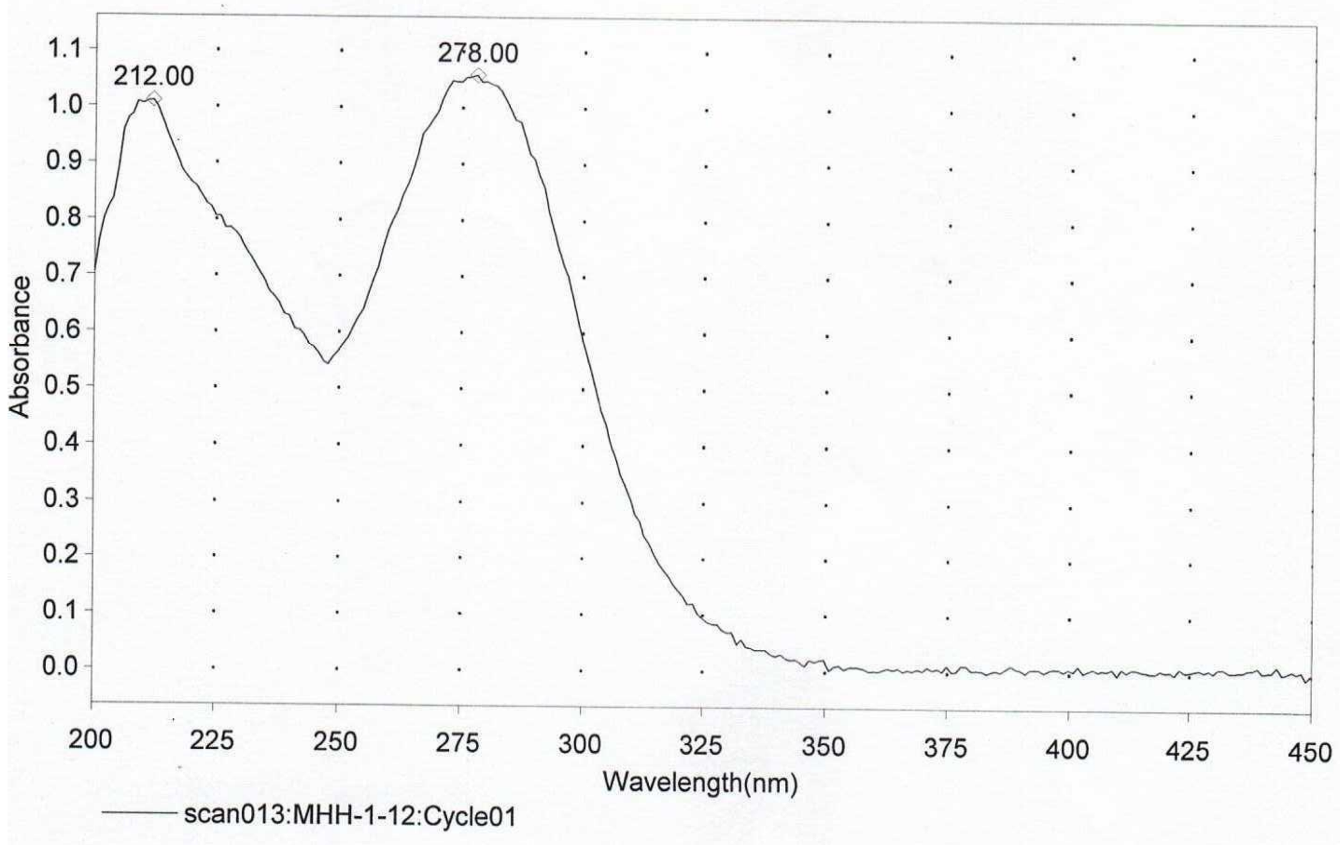

Results Table - MHH-1-12.sre,MHH-1-12,Cycle01

nm	A	Peak Pick Method
212.00	1.011	Find 8 Peaks Above -3.0000 A
278.00	1.058	Start Wavelength 200.00 nm
		Stop Wavelength 450.00 nm
		Sort By Wavelength

Sensitivity Auto
