# Supplementary material for: Sulphamethazine derivatives as immunomodulating agents: New therapeutic strategies for inflammatory diseases
Source: PLoS One. 2018 Dec 19;13(12):e0208933. doi: 10.1371/journal.pone.0208933 (PMC6300282; doi:10.1371/journal.pone.0208933)
Supplement: S20 Fig — (PDF) [file pone.0208933.s020.pdf]

DR.HAROON/DR.HINA/MHH.I.13
1H

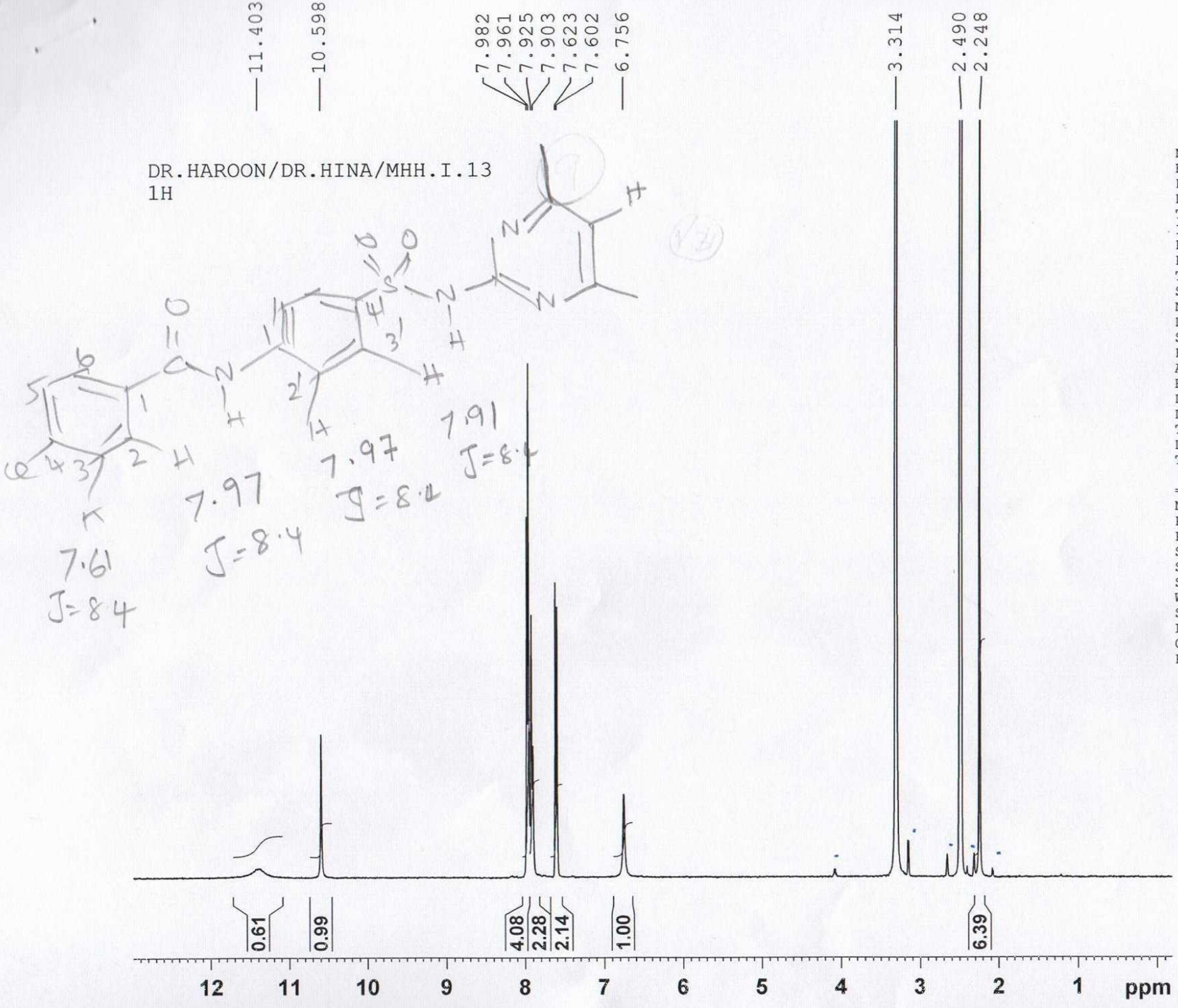

AVANCE AV-400 MHz
Lab # 115

(22)

NAME	dec28-16
EXPNO	12
PROCNO	1
Date_	20161228
Time	13.56
INSTRUM	spect
PROBHD	5 mm SEI 1H-13
PULPROG	zg30
TD	65536
SOLVENT	DMSO
NS	64
DS	0
SWH	8012.820 Hz
FIDRES	0.122266 Hz
AQ	4.0894966 sec
RG	574.7
DW	62.400 usec
DE	6.50 usec
TE	300.0 K
D1	2.00000000 sec
TD0	1

======== CHANNEL f1 ========

NUC1	1H
P1	10.80 usec
PL1	3.00 dB
SFO1	400.0332002 MHz
SI	32768
SF	400.0300041 MHz
WDW	EM
SSB	0
LB	0.30 Hz
GB	0
PC	1.00

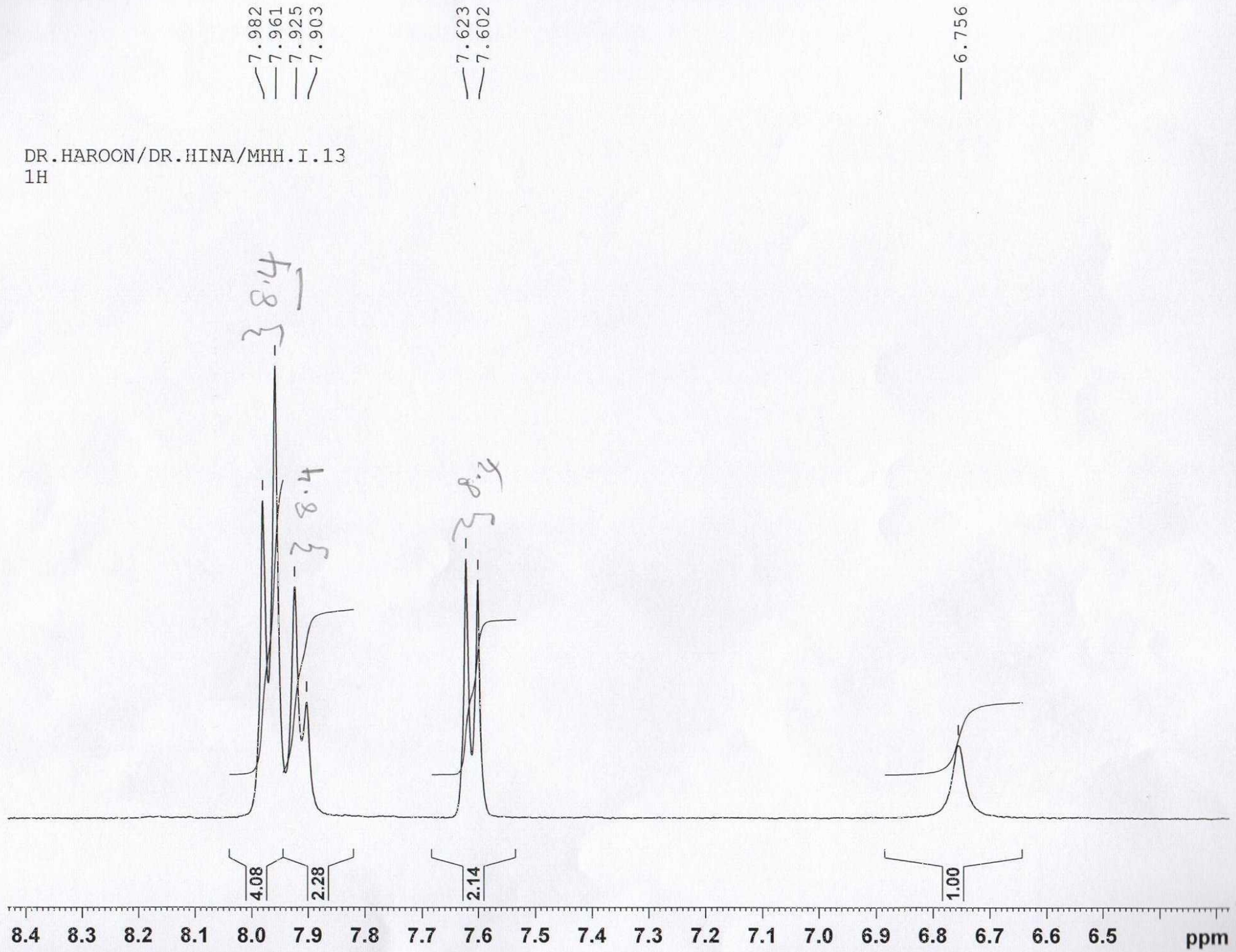
7.982
7.961
7.925
7.903
7.623
7.602
6.756
DR.HAROON/DR.HINA/MHH.I.13
1H
4.08
2.28
2.14
1.00
8.4 8.3 8.2 8.1 8.0 7.9 7.8 7.7 7.6 7.5 7.4 7.3 7.2 7.1 7.0 6.9 6.8 6.7 6.6 6.5
ppm

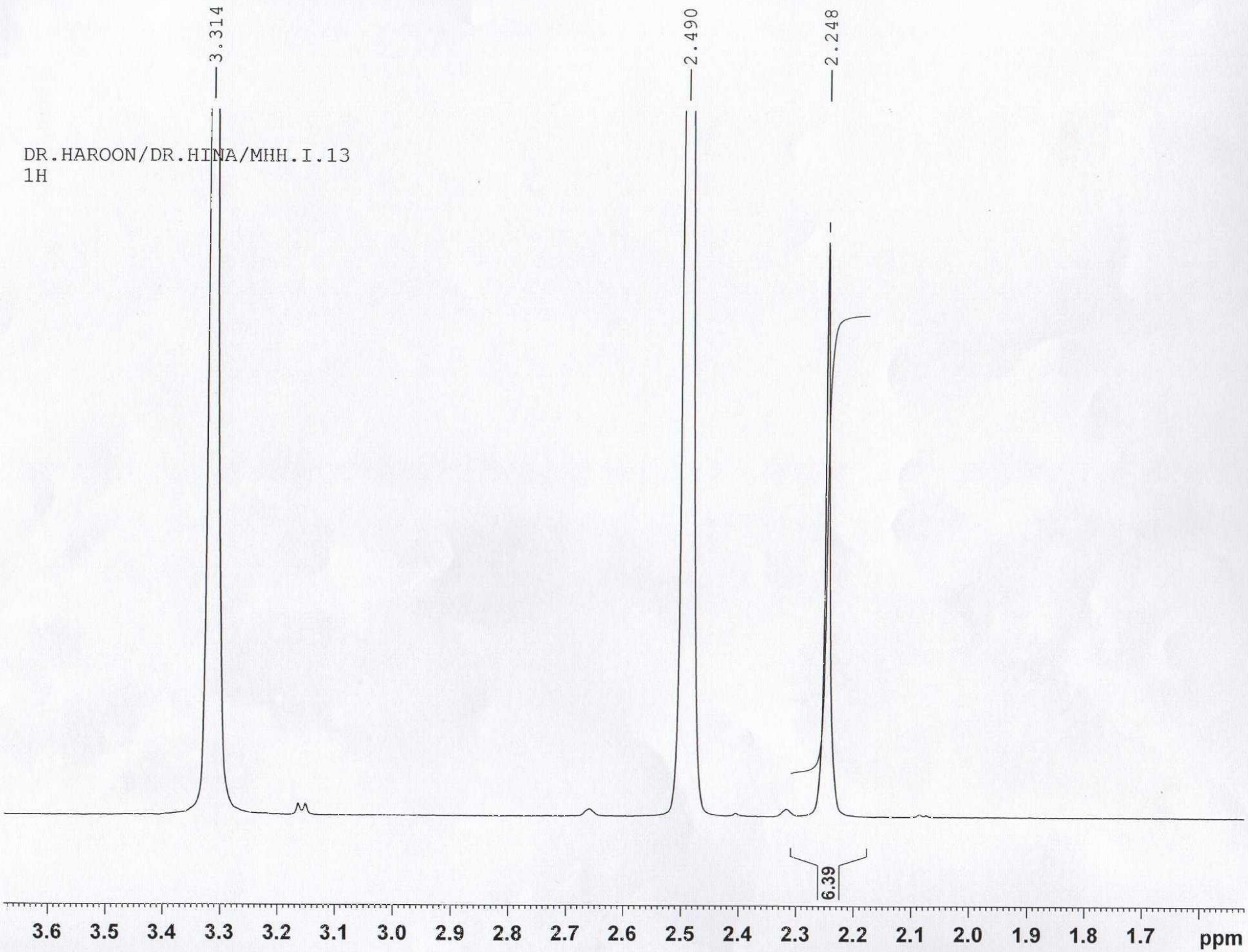
DR.HAROON/DR.HINA/MHH.I.13
1H
3.314
2.490
2.248
6.39
3.6 3.5 3.4 3.3 3.2 3.1 3.0 2.9 2.8 2.7 2.6 2.5 2.4 2.3 2.2 2.1 2.0 1.9 1.8 1.7
ppm

2/7/2017 3:37:24 PM

File: MHH-I-13
Sample: DR.M.H.HAROON /DR. HINA
Instrument: JEOL MS 600H-1

Date Run: 02-07-2017 (Time Run: 15:25:33)

Ionization mode: EI+

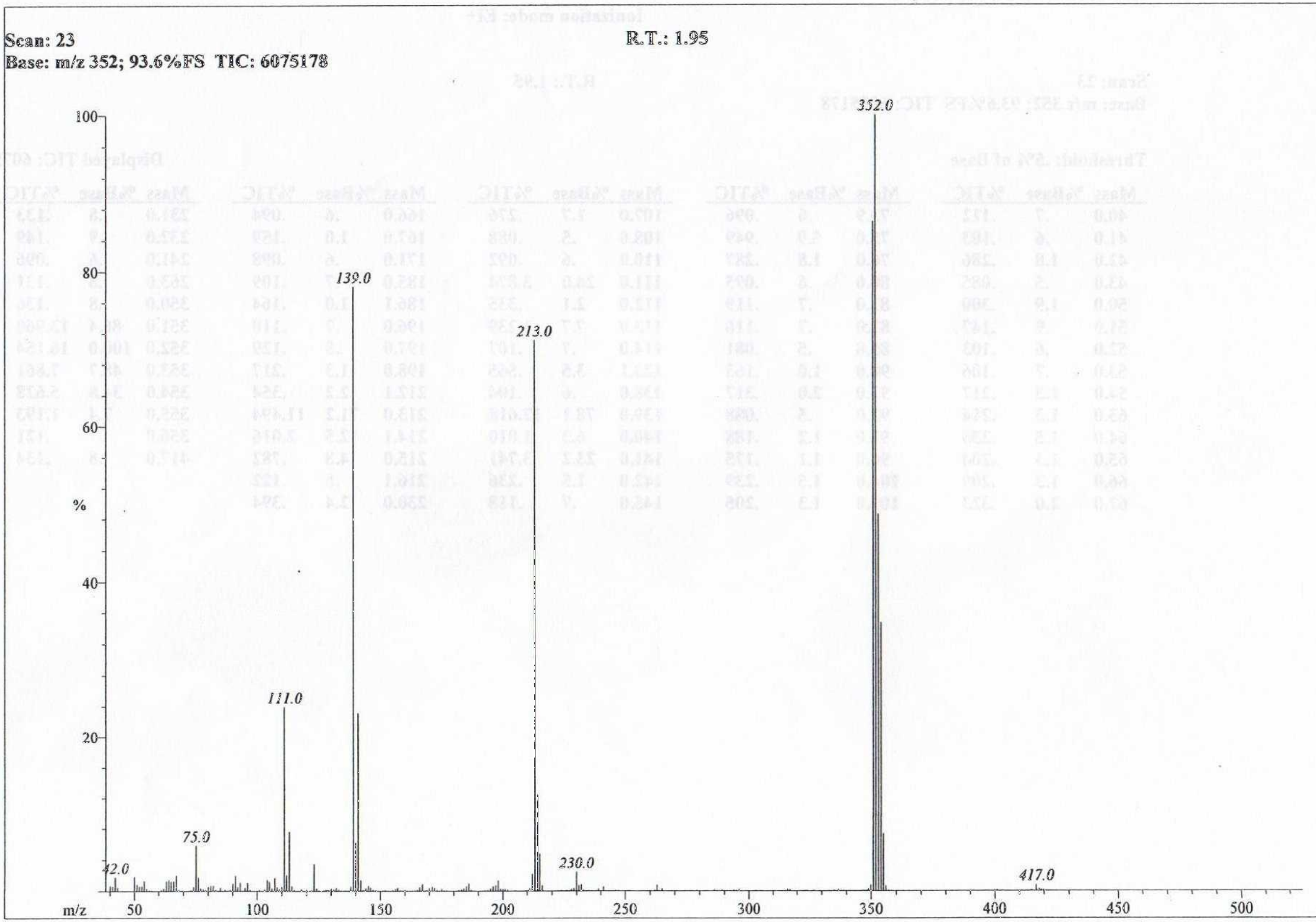

I. C.C.B.S., University of Karachi Analytical Laboratory - Pakistan

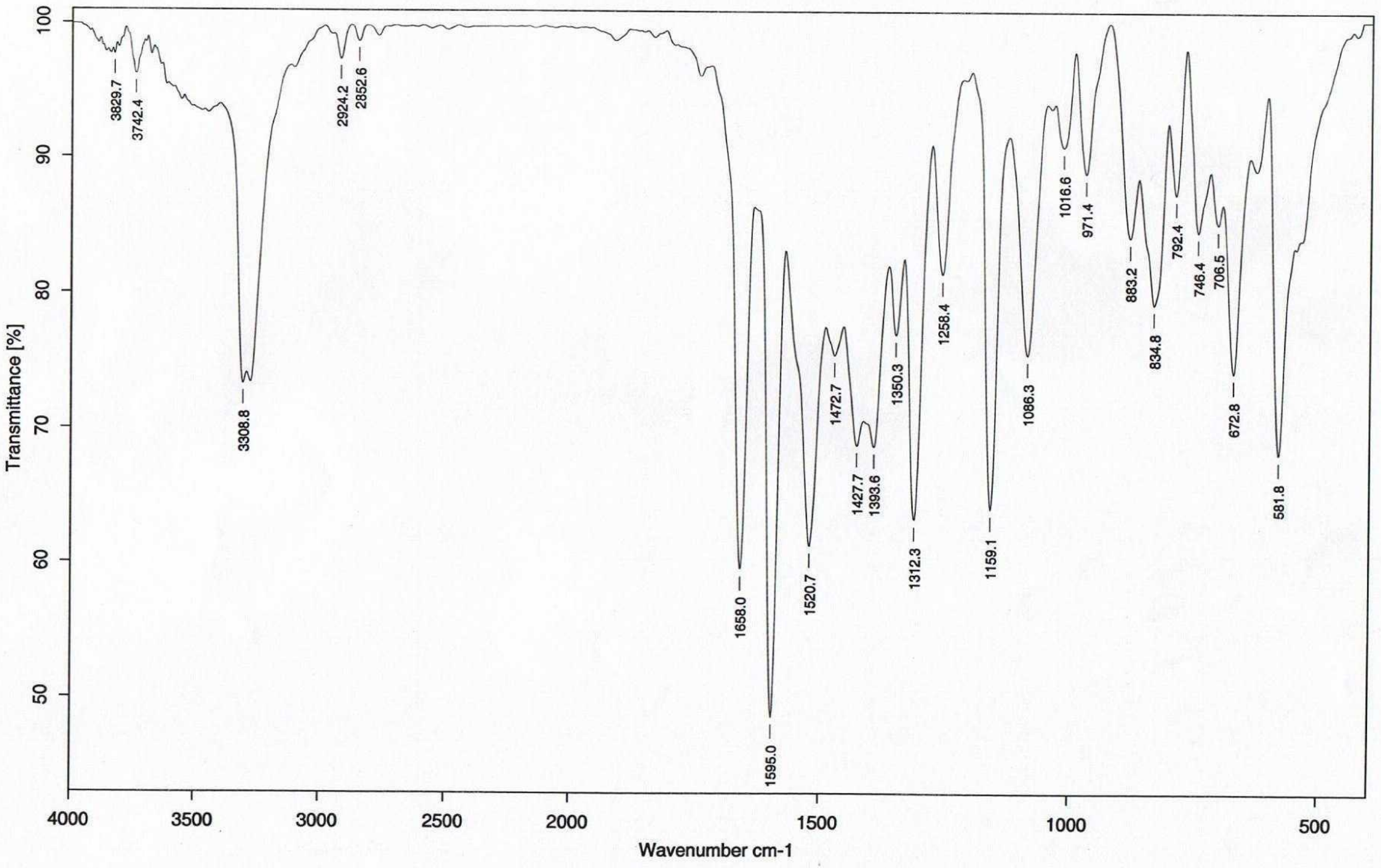

Sample : MHH-1-13/Haroon/Dr. Hina	Spectrum : MHH-1-13.0 (in D:\IRSTUDENT)
Measured : 31/01/2017 on VECTOR22	Technic : Solid
Resolution : 4 cm-1 (10 scans)	Analyst : Zubair Ahmad/ Jamshed/M. Asif/

ΓHERMO ELECTRON ~ VISIONpro SOFTWARE V4.10

)perator Name	ARSHAD ALAM.	Date of Report	2/1/2017
)epartment	Analytical Laboratory TWC # 004	Time of Report	3:21:59PM
)rganization	ICCBS Karachi of Universty.		
nformation	Dr Haroon/Dr Hina		

;can Graph

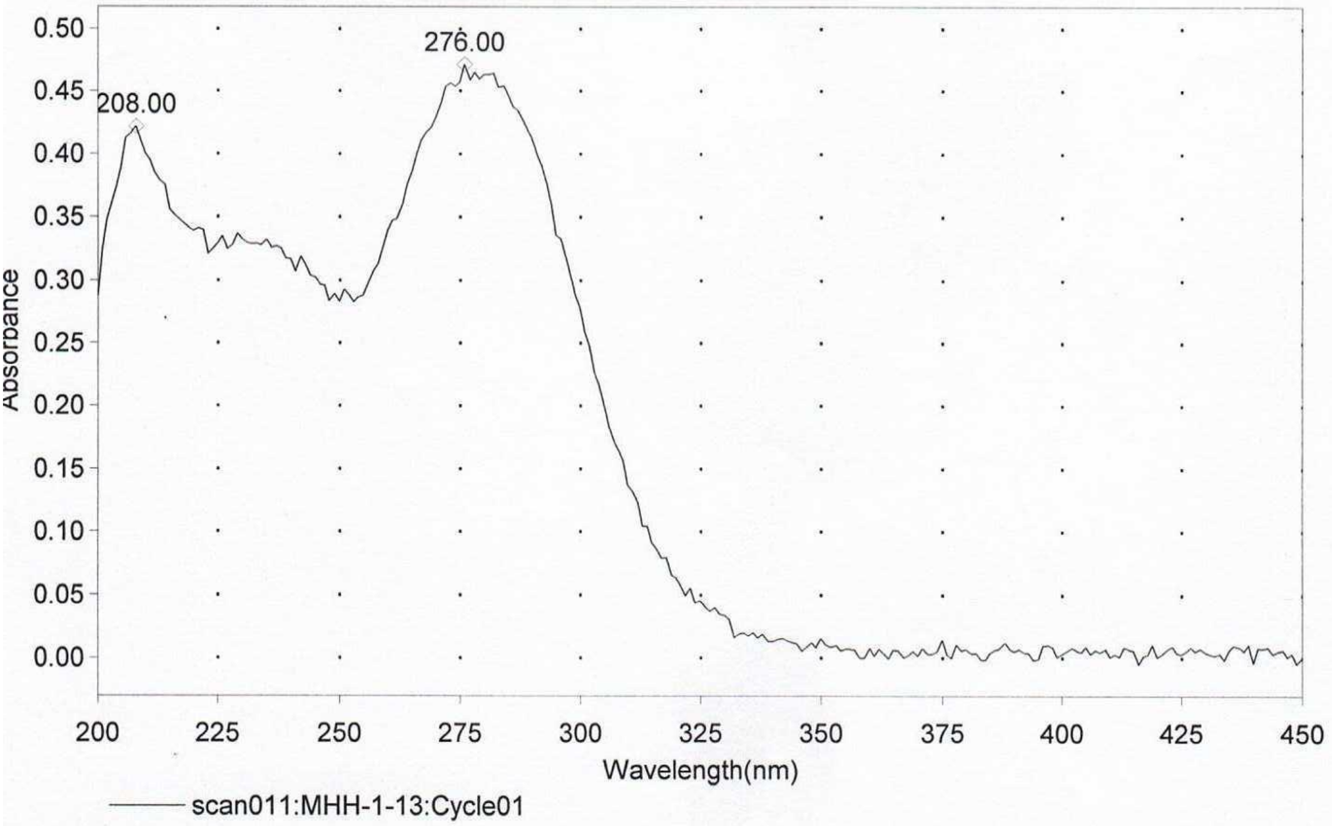

łesults Table - MHH-1-4.sre,MHH-1-13,Cycle01

ım	A	**Peak Pick Method**
:08.00	0.421	Find 8 Peaks Above -3.0000 A
:76.00	0.471	Start Wavelength 200.00 nm
		Stop Wavelength 450.00 nm
		Sort By Wavelength

;ensitivity Auto
