# Supplementary material for: Sulphamethazine derivatives as immunomodulating agents: New therapeutic strategies for inflammatory diseases
Source: PLoS One. 2018 Dec 19;13(12):e0208933. doi: 10.1371/journal.pone.0208933 (PMC6300282; doi:10.1371/journal.pone.0208933)
Supplement: S21 Fig — (PDF) [file pone.0208933.s021.pdf]

DR.HAROON/DR.HINA/MHH.I.32
1H

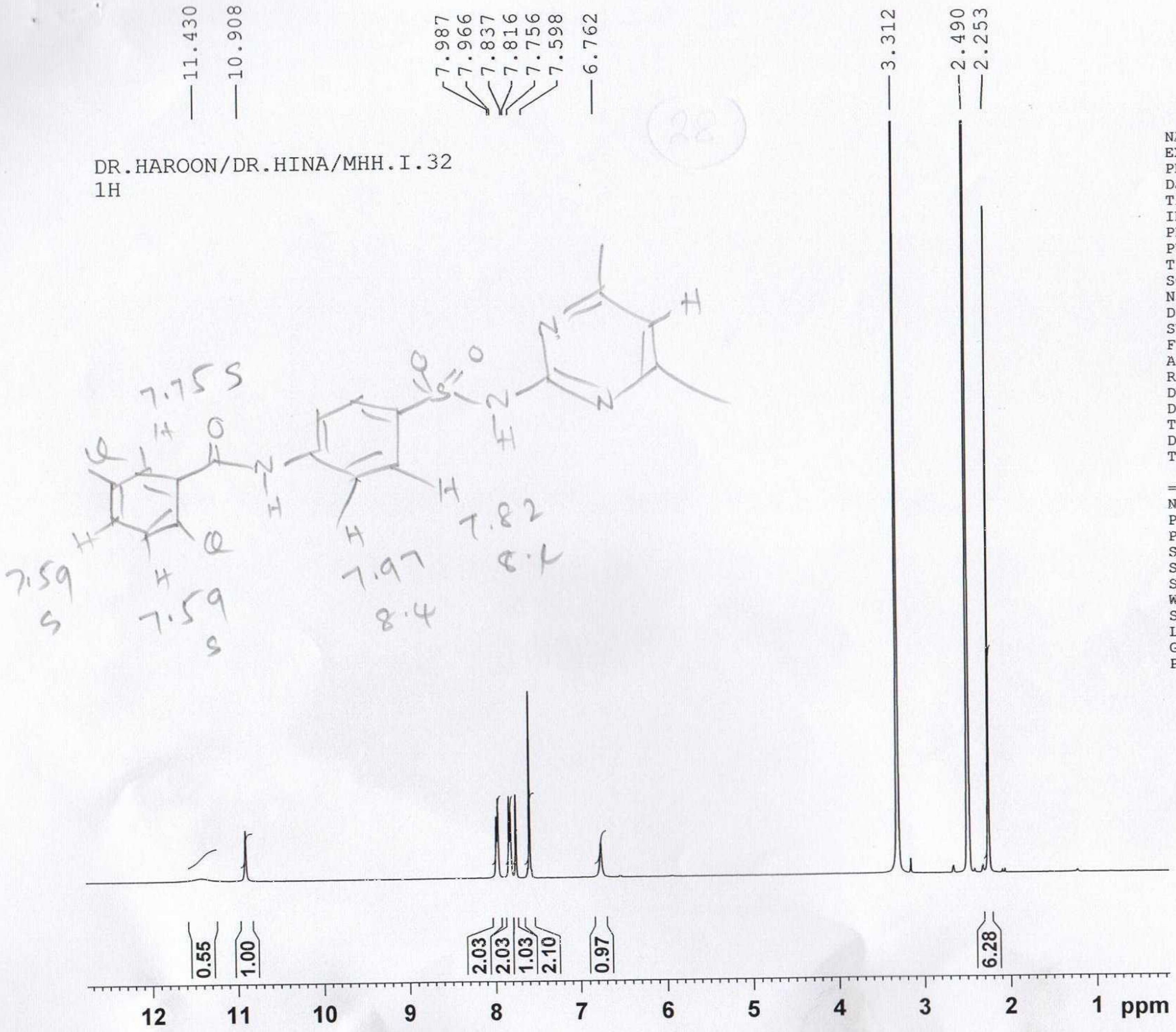

AVANCE AV-400 MHz
Lab # 115

(23)

NAME	jan05-17
EXPNO	4
PROCNO	1
Date_	20170105
Time	11.35
INSTRUM	spect
PROBHD	5 mm SEI 1H-13
PULPROG	zg30
TD	65536
SOLVENT	DMSO
NS	64
DS	0
SWH	8012.820 Hz
FIDRES	0.122266 Hz
AQ	4.0894966 sec
RG	362
DW	62.400 usec
DE	6.50 usec
TE	300.0 K
D1	2.00000000 sec
TD0	1

======== CHANNEL f1 ========

NUC1	1H
P1	10.80 usec
PL1	3.00 dB
SFO1	400.0332002 MHz
SI	32768
SF	400.0300041 MHz
WDW	EM
SSB	0
LB	0.30 Hz
GB	0
PC	1.00

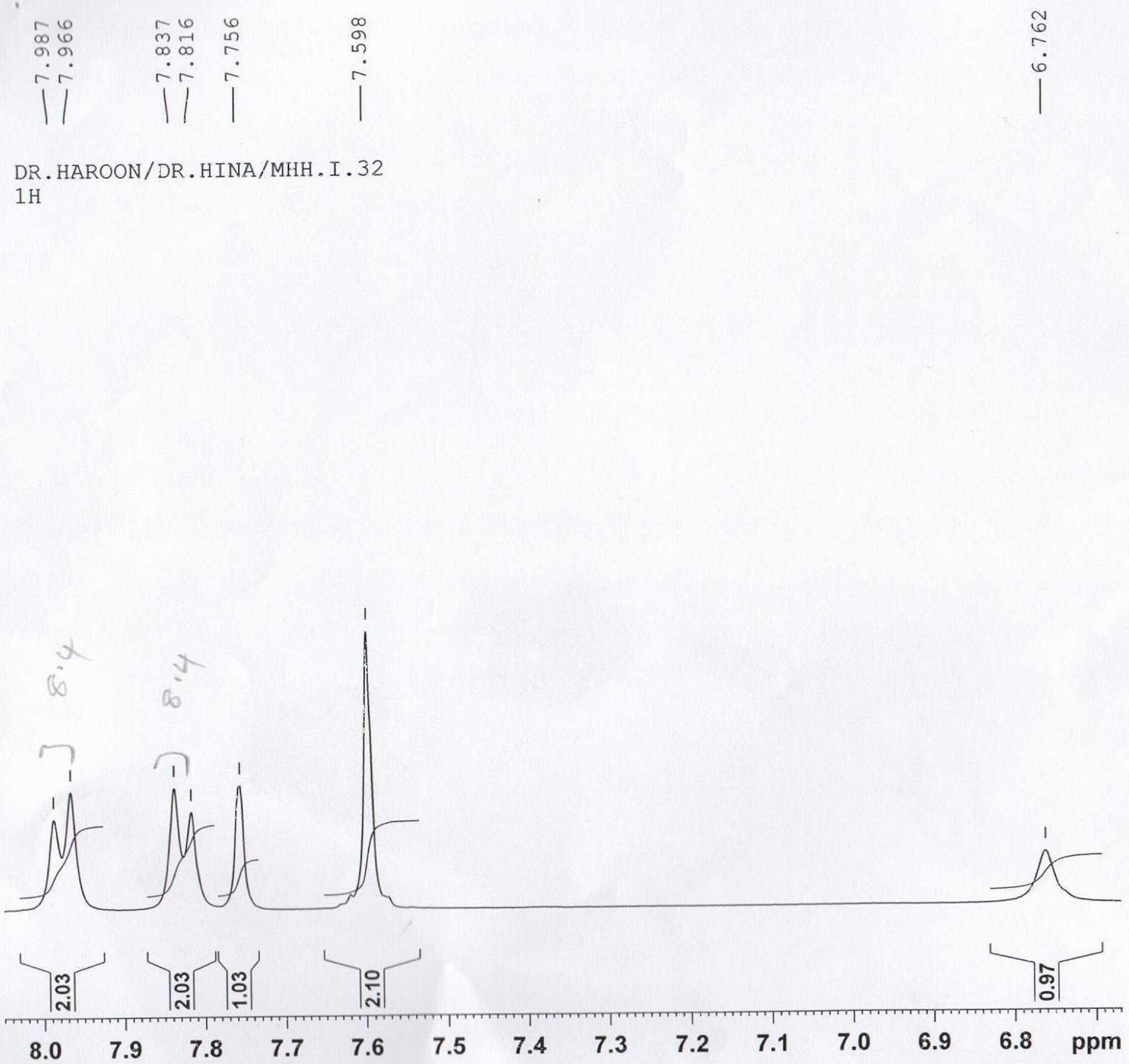

7.987
7.966
7.837
7.816
7.756
7.598
6.762
DR.HAROON/DR.HINA/MHH.I.32
1H
8.4
8.4
2.03
2.03
1.03
2.10
0.97
8.0
7.9
7.8
7.7
7.6
7.5
7.4
7.3
7.2
7.1
7.0
6.9
6.8
ppm

2/10/2017 10:05:47 AM

File: MHH-I-32
Sample: DR.M.H.HAROON /DR. HINA
Instrument: JEOL MS 600H-1

Date Run: 02-10-2017 (Time Run: 09:57:28)

Ionization mode: EI+

Scan: 17
Base: m/z 213; 99.5%FS TIC: 8674944

R.T.: 1.42

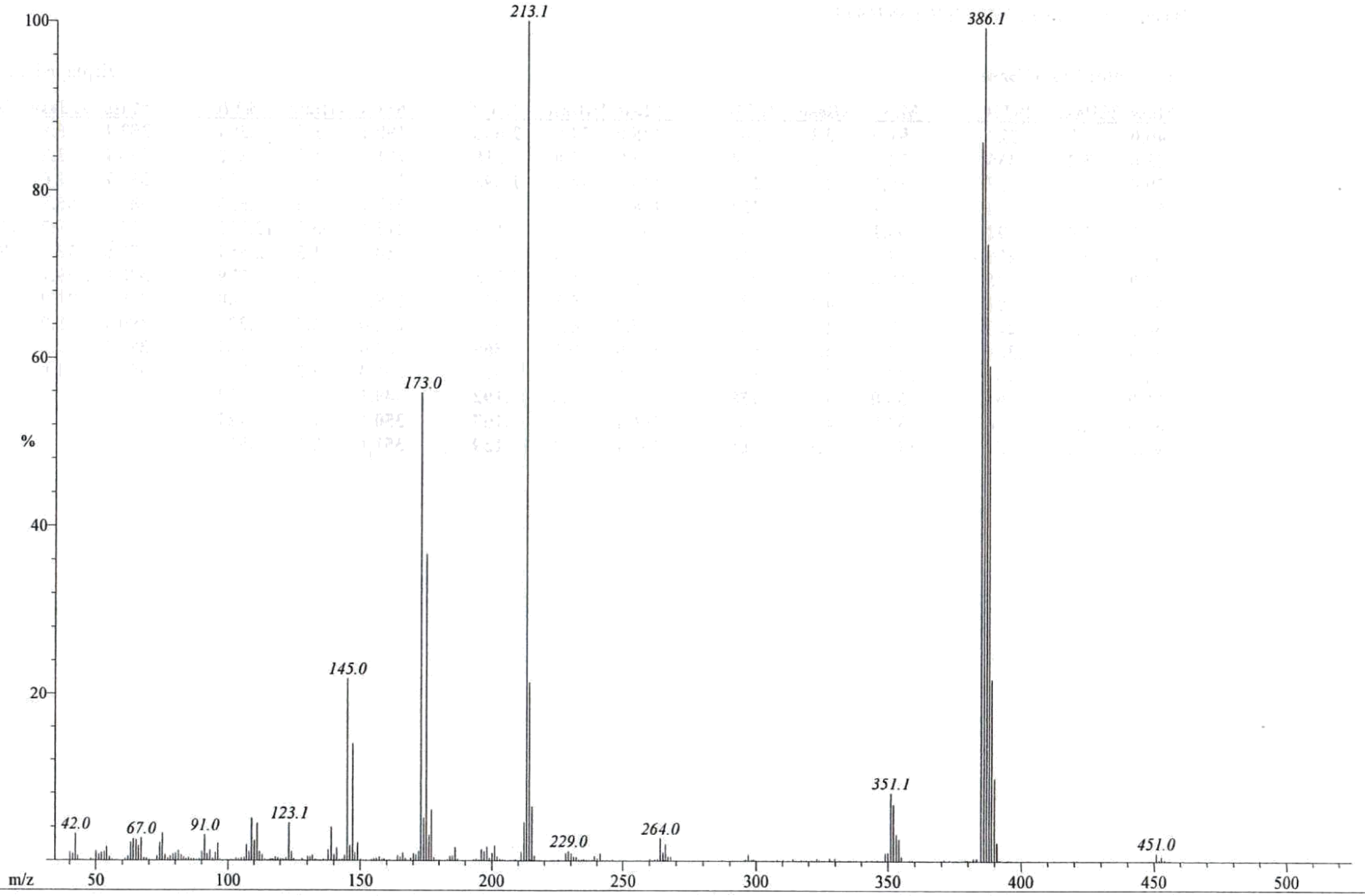

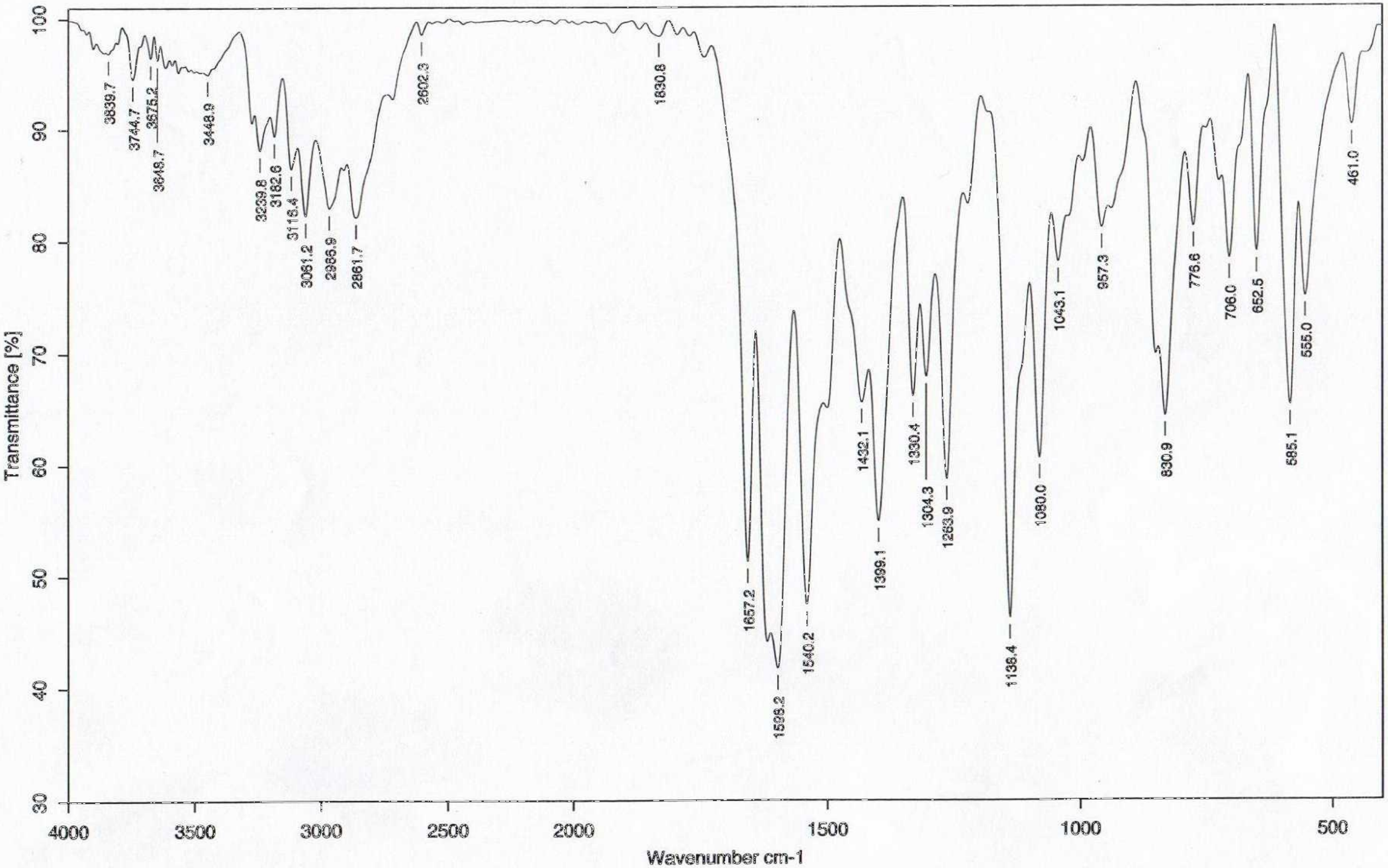

Sample : MHH-1-32/Dr.Haroon	Spectrum : MHH-1-32.0 (in D:\IRSTUDENT)
Measured : 27/01/2017 on VECTOR22	Technic : Liquid
Resolution : 4 cm-1 (10 scans)	Analyst : M. Asif

THERMO ELECTRON ~ VISIONpro SOFTWARE V4.10

Operator Name	ARSHAD ALAM.	Date of Report	1/30/2017
Department	Analytical Laboratory TWC # 004	Time of Report	10:46:54AM
Organization	ICCBS Karachi of Universty.		
Information	Dr Haron/Dr Hina		

Scan Graph

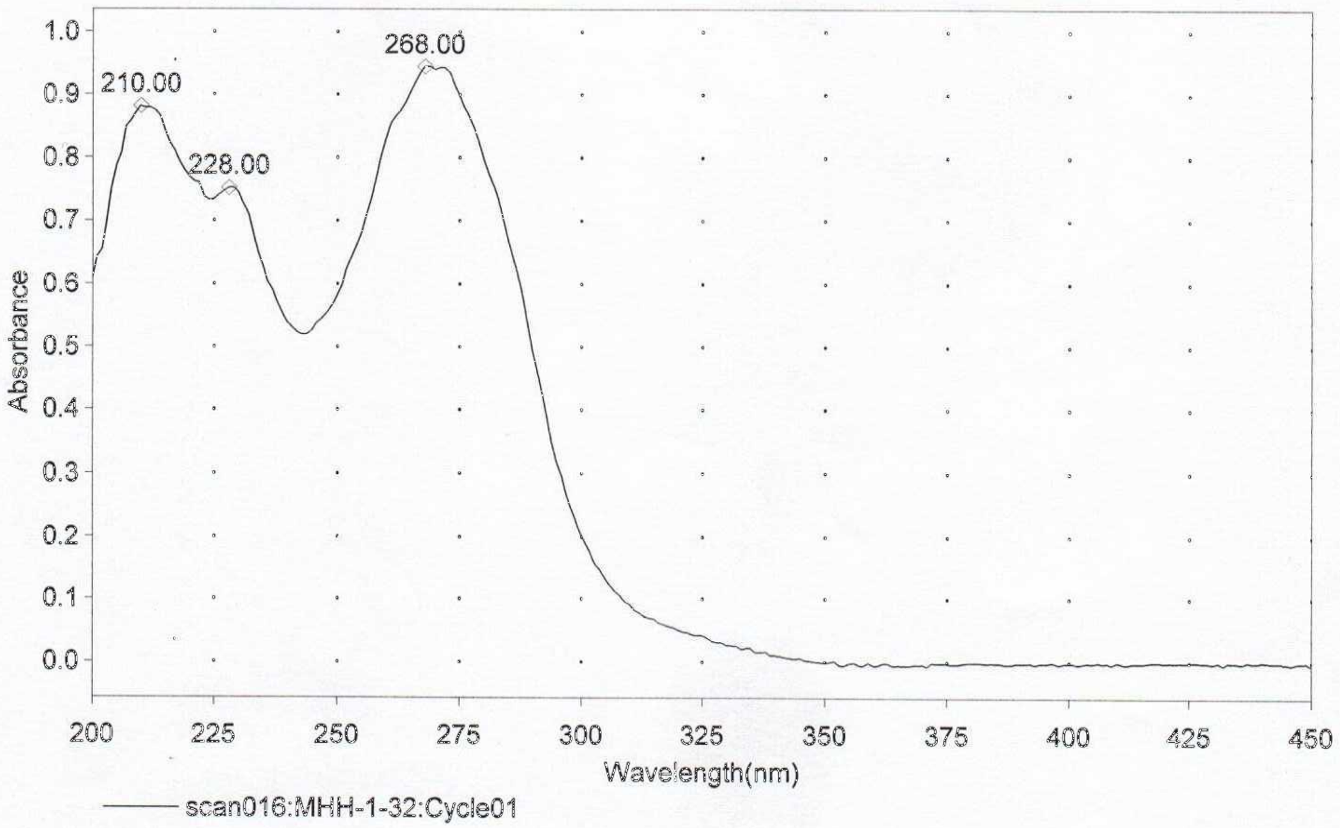

Results Table - MHH-1-32.sre,MHH-1-32,Cycle01

nm	A	
210.00	0.882	**Peak Pick Method**
228.00	0.752	Find 8 Peaks Above -3.0000 A
268.00	0.945	Start Wavelength 200.00 nm
		Stop Wavelength 450.00 nm
		Sort By Wavelength

Sensitivity Low
