# Supplementary material for: Sulphamethazine derivatives as immunomodulating agents: New therapeutic strategies for inflammatory diseases
Source: PLoS One. 2018 Dec 19;13(12):e0208933. doi: 10.1371/journal.pone.0208933 (PMC6300282; doi:10.1371/journal.pone.0208933)
Supplement: S22 Fig — (PDF) [file pone.0208933.s022.pdf]

AVANCE AV-400 MHz
Lab # 115

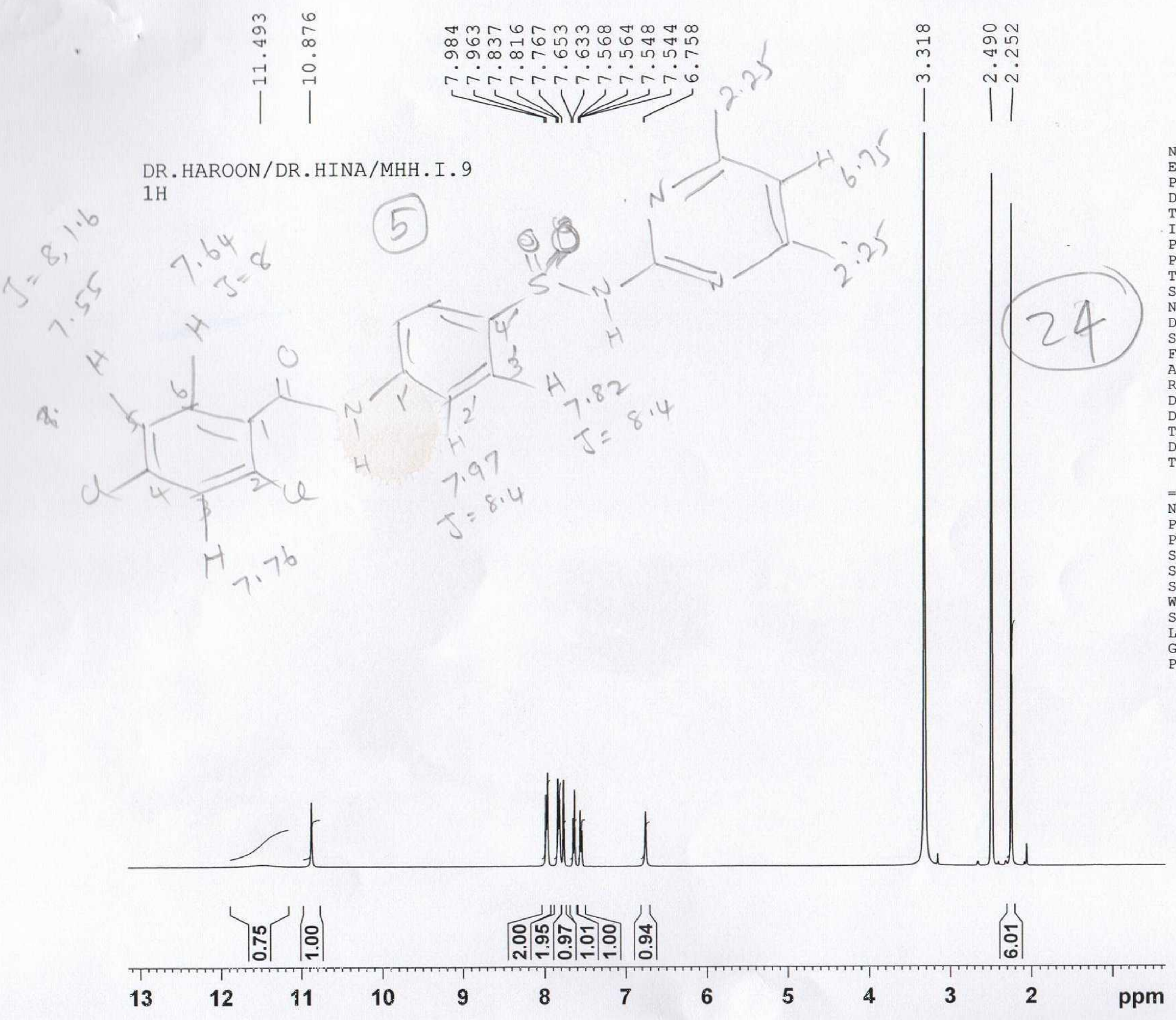

NAME	jan05-17
EXPNO	3
PROCNO	1
Date_	20170105
Time	11.24
INSTRUM	spect
PROBHD	5 mm SEI 1H-13
PULPROG	zg30
TD	65536
SOLVENT	DMSO
NS	64
DS	0
SWH	8012.820 Hz
FIDRES	0.122266 Hz
AQ	4.0894966 sec
RG	362
DW	62.400 usec
DE	6.50 usec
TE	300.0 K
D1	2.00000000 sec
TD0	1

======== CHANNEL f1 ========

NUC1	1H
P1	10.80 usec
PL1	3.00 dB
SFO1	400.0332002 MHz
SI	32768
SF	400.0300041 MHz
WDW	EM
SSB	0
LB	0.30 Hz
GB	0
PC	1.00

DR.HAROON/DR.HINA/MHH.I.9
1H

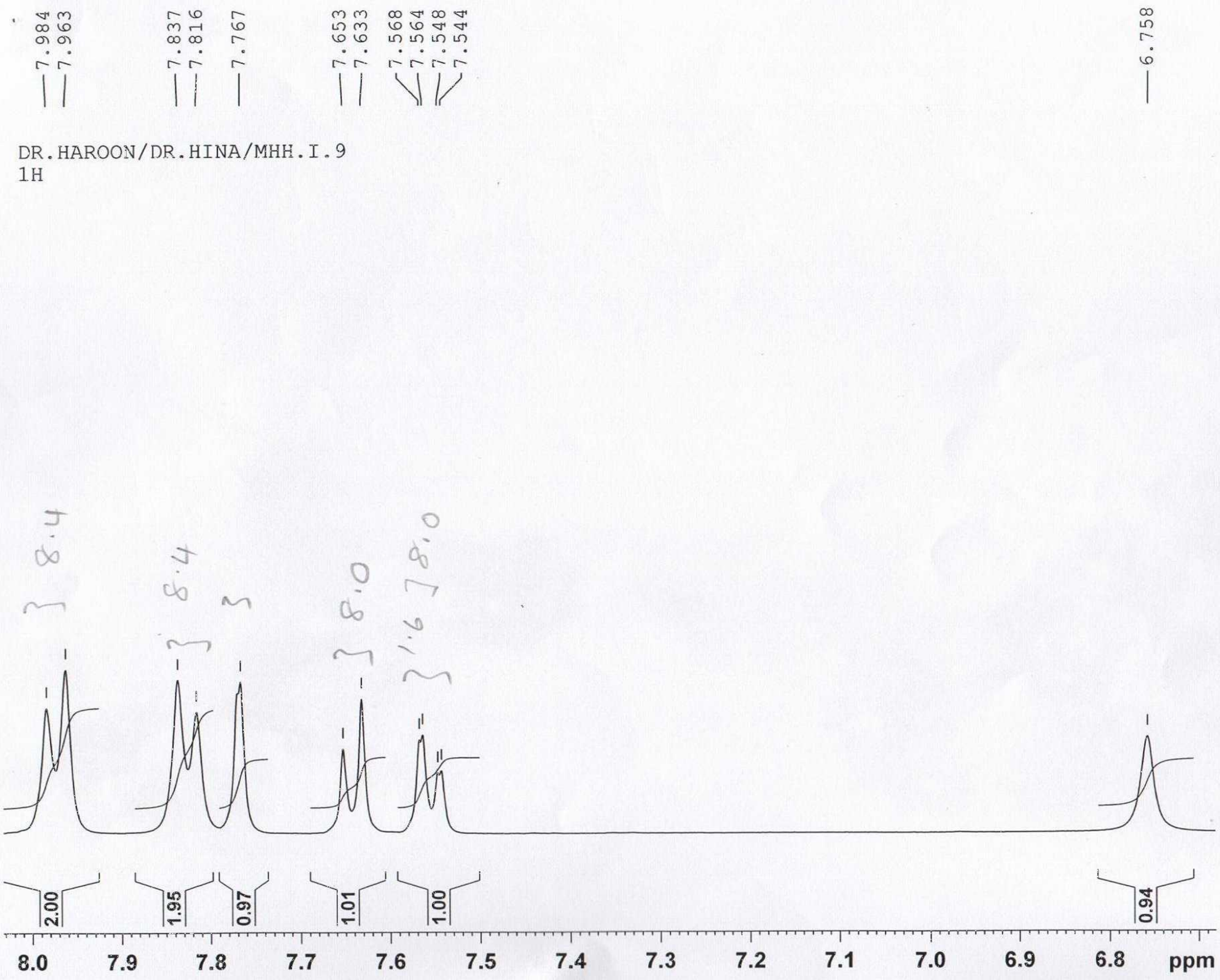

File: MHH-I-9-FABP-
Sample: DR.M.H.HAROON /DR. HINA
Instrument: JEOL-600H-2
Inlet: Direct Probe

Date Run: 02-27-2017 (Time Run: 11:38:30)

Ionization mode: FAB+

Scan: 10
Base: m/z 185; 100%FS TIC: 1801382

R.T.: .8

#Ions: 61

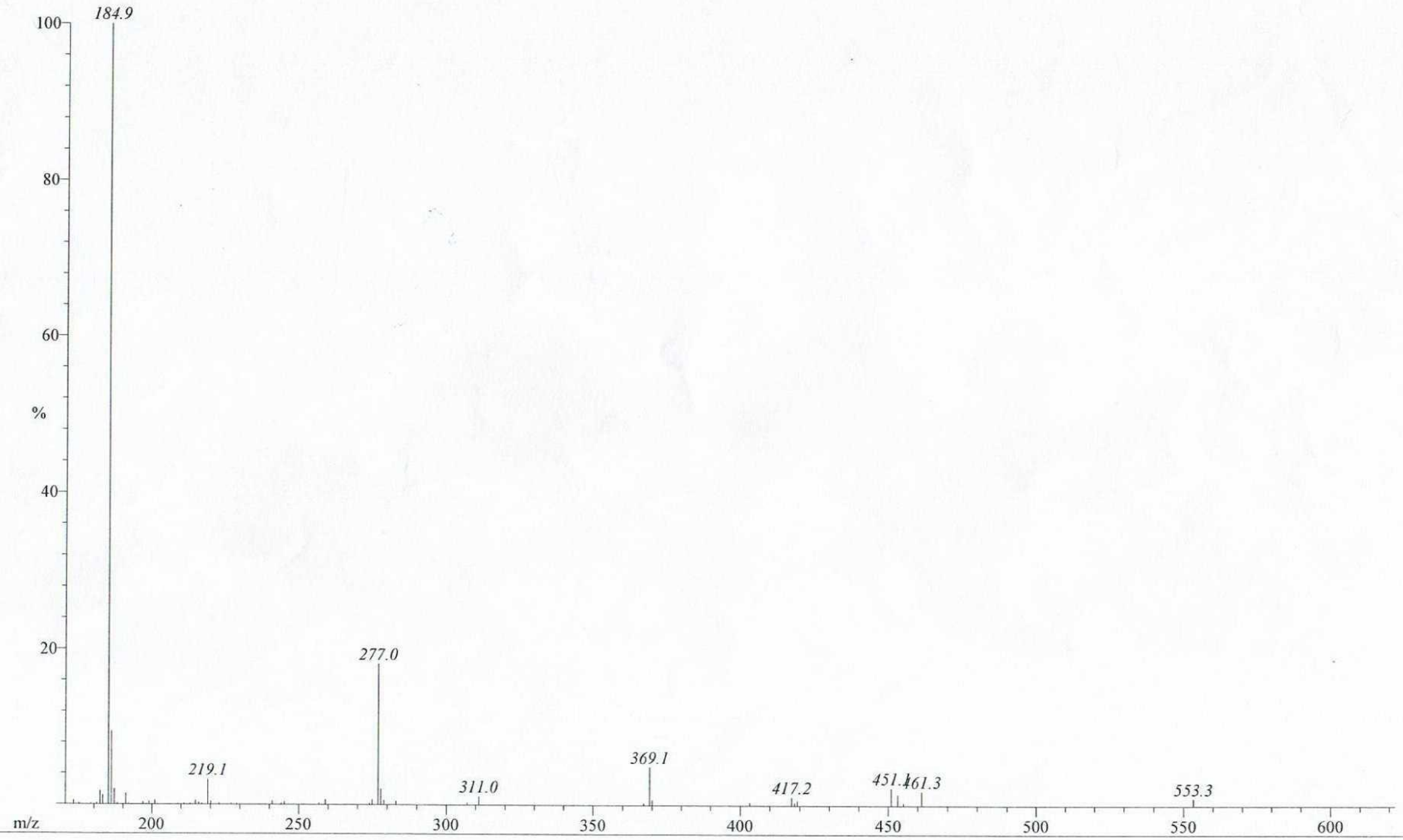

I. C.C.B.S., University of Karachi Analytical Laboratory - Pakistan

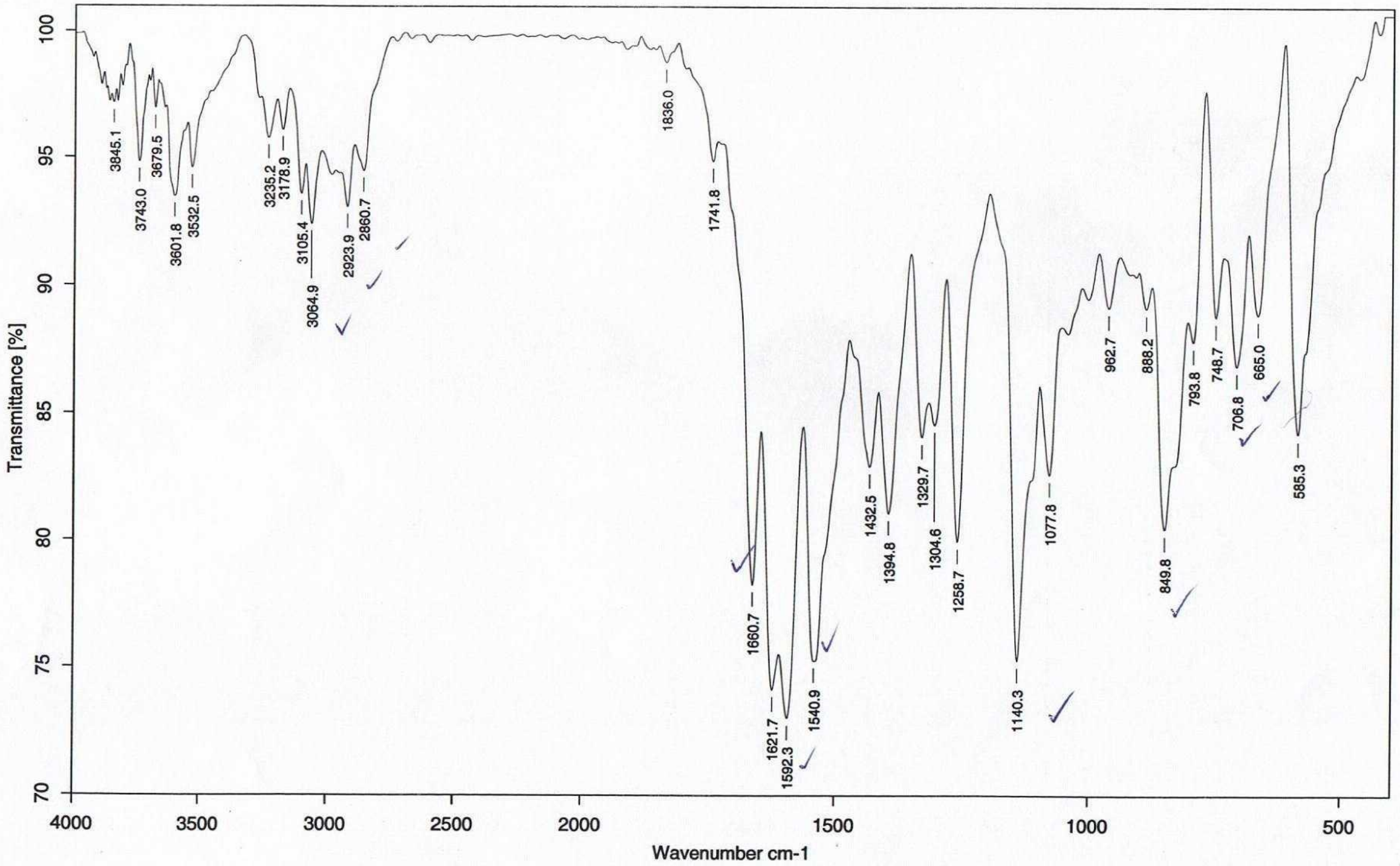

Sample : MHH-1-9/Haroon/Dr. Hina

Measured : 31/01/2017 on VECTOR22

Resolution : 4 cm-1 (10 scans)

Spectrum : MHH-1-9.0 (in D:\IRSTUDENT)

Technic : Solid

Analyst : Zubair Ahmad/ Jamshed/M. Asif/

THERMO ELECTRON ~ VISIONpro SOFTWARE V4.10

Operator Name	ARSHAD ALAM.	Date of Report	2/1/2017
Department	Analytical Laboratory TWC # 004	Time of Report	4:07:19PM
Organization	ICCBS Karachi of Universty.		
Information	Dr Haroon/Dr Hina		

Scan Graph

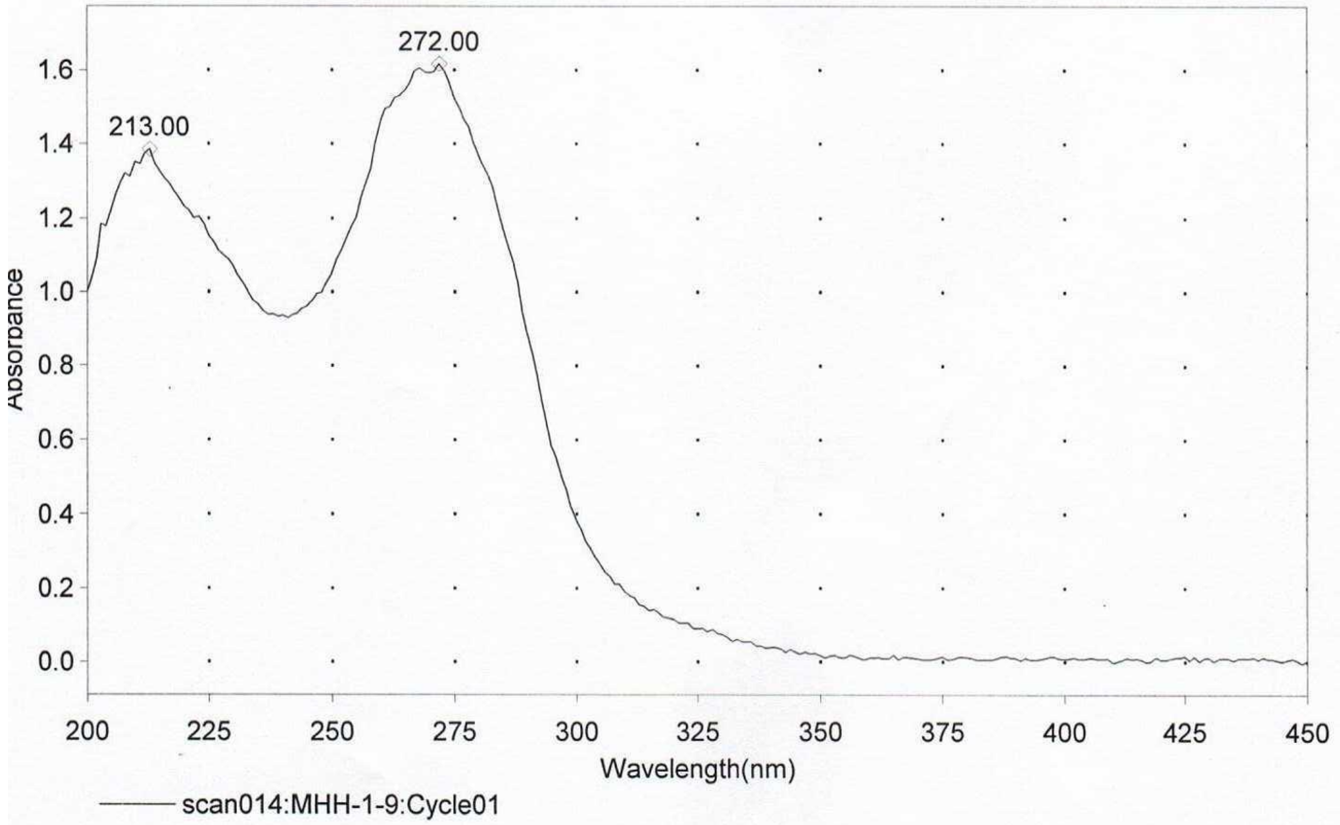

Results Table - MHH-1-9.sre,MHH-1-9,Cycle01

nm	A	**Peak Pick Method**
213.00	1.385	Find 8 Peaks Above -3.0000 A
272.00	1.617	Start Wavelength 200.00 nm
		Stop Wavelength 450.00 nm
		Sort By Wavelength

Sensitivity Auto
