# Supplementary material for: Sulphamethazine derivatives as immunomodulating agents: New therapeutic strategies for inflammatory diseases
Source: PLoS One. 2018 Dec 19;13(12):e0208933. doi: 10.1371/journal.pone.0208933 (PMC6300282; doi:10.1371/journal.pone.0208933)
Supplement: S23 Fig — (PDF) [file pone.0208933.s023.pdf]

MHH-1-6

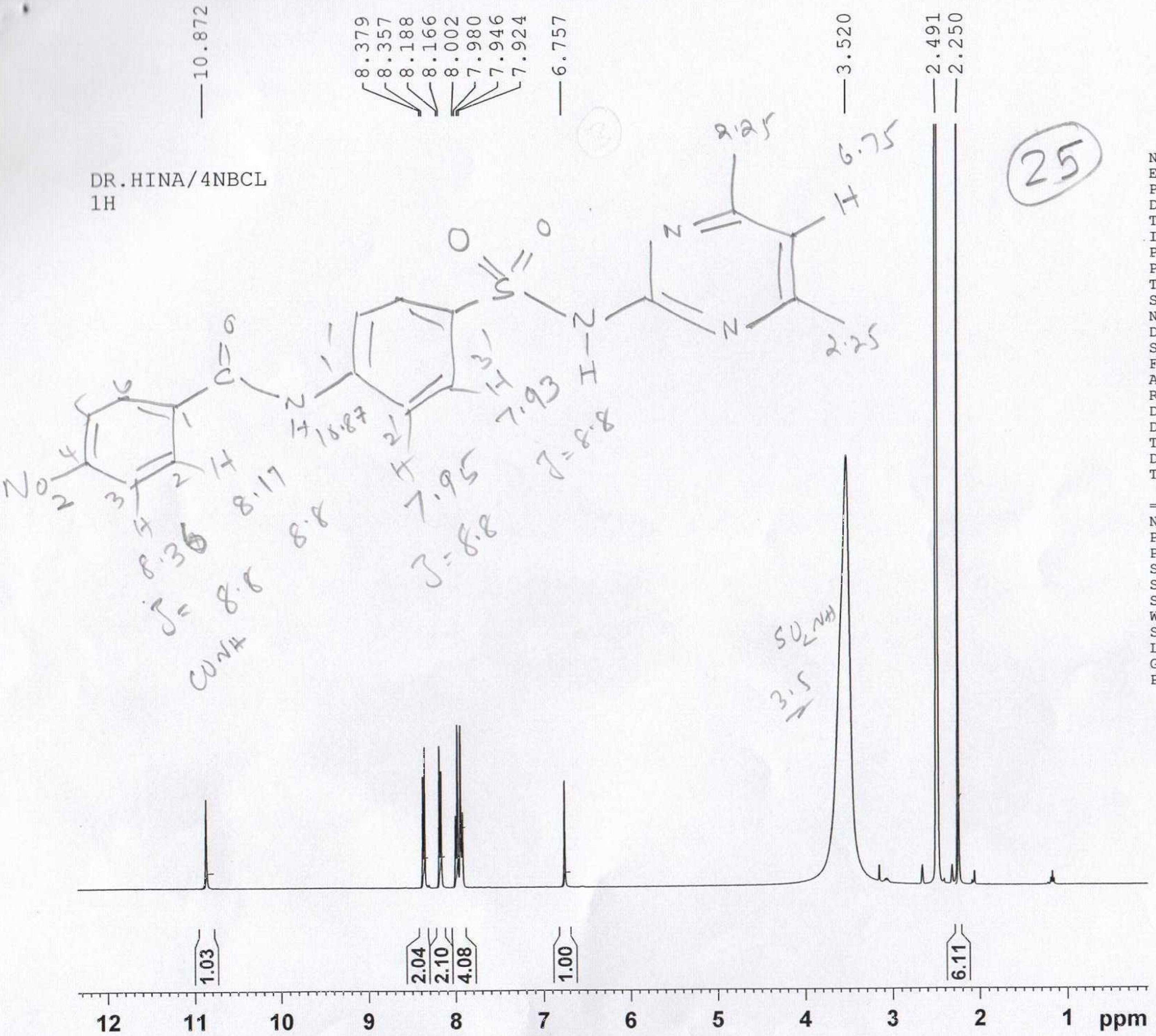

AVANCE AV-400 MHz
Lab # 115

NAME	dec05-16
EXPNO	1
PROCNO	1
Date_	20161205
Time	8.51
INSTRUM	spect
PROBHD	5 mm SEI 1H-13
PULPROG	zg30
TD	65536
SOLVENT	DMSO
NS	128
DS	0
SWH	8012.820 Hz
FIDRES	0.122266 Hz
AQ	4.0894966 sec
RG	362
DW	62.400 usec
DE	6.50 usec
TE	300.0 K
D1	1.50000000 sec
TD0	1

======== CHANNEL f1 ========

NUC1	1H
P1	10.80 usec
PL1	3.00 dB
SFO1	400.0332002 MHz
SI	32768
SF	400.0300041 MHz
WDW	EM
SSB	0
LB	0.30 Hz
GB	0
PC	1.00

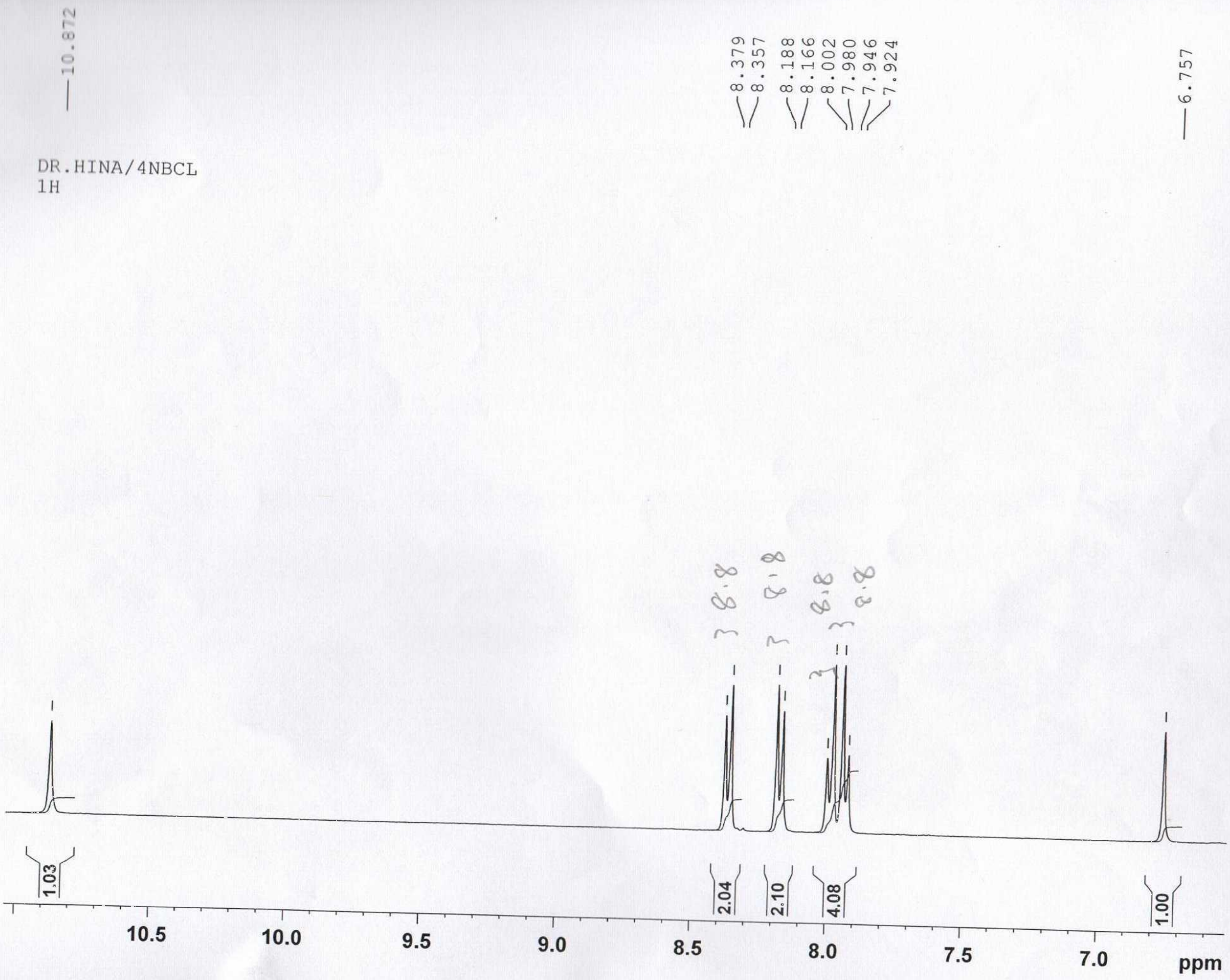
10.872
8.379
8.357
8.188
8.166
8.002
7.980
7.946
7.924
6.757
DR.HINA/4NBCL
1H
1.03
2.04
2.10
4.08
1.00
10.5
10.0
9.5
9.0
8.5
8.0
7.5
7.0
ppm

2/13/2017 2:07:06 PM

File: MHH-I-06
Sample: DR.M.H.HAROON /DR. HINA
Instrument: JEOL MS 600H-1

Date Run: 02-13-2017 (Time Run: 13:56:12)

Ionization mode: EI+

Scan: 26
Base: m/z 362; 29.3%FS TIC: 1569366

R.T.: 2.22

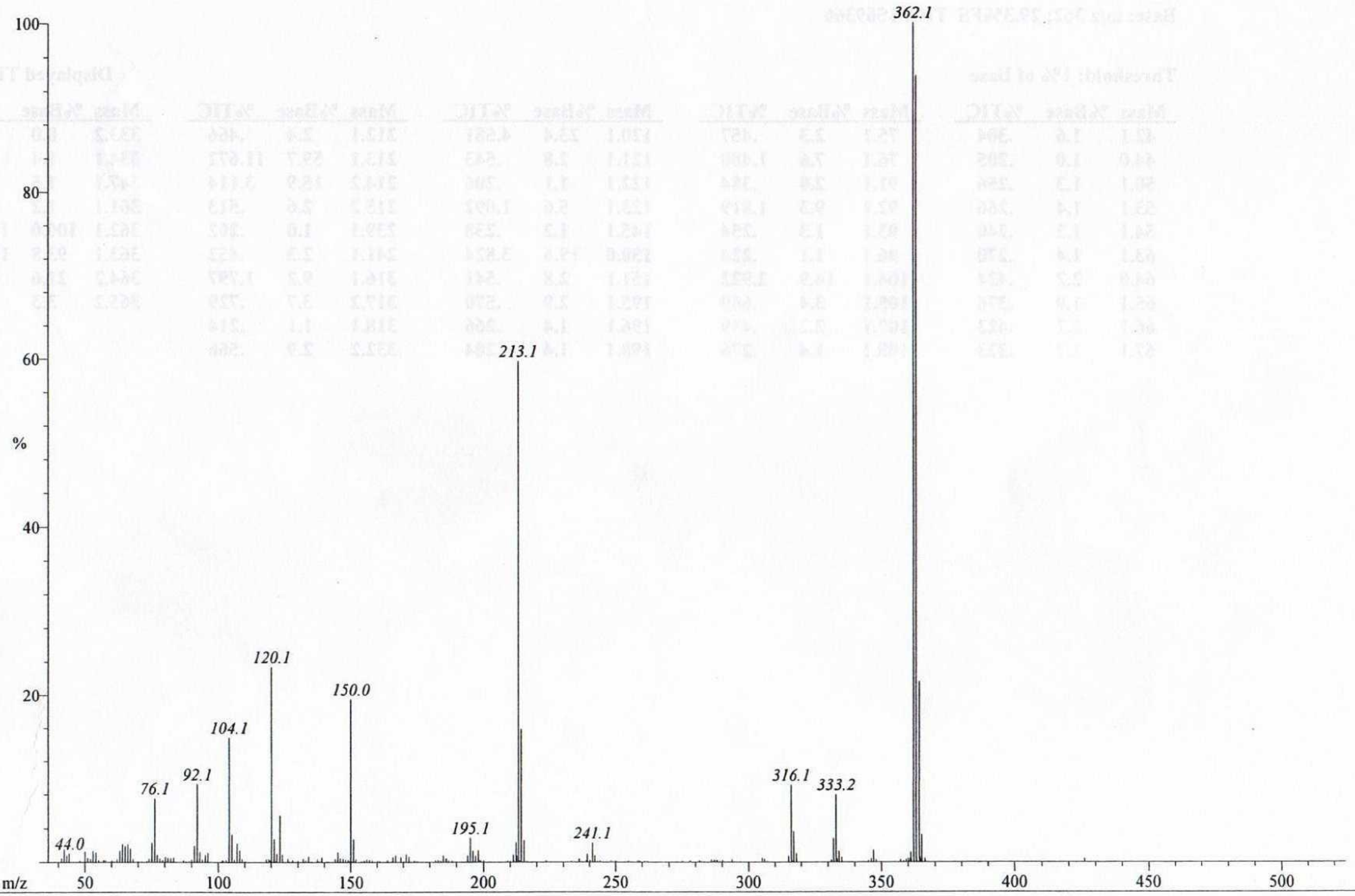

I. C.C.B.S., University of Karachi Analytical Laboratory - Pakistan

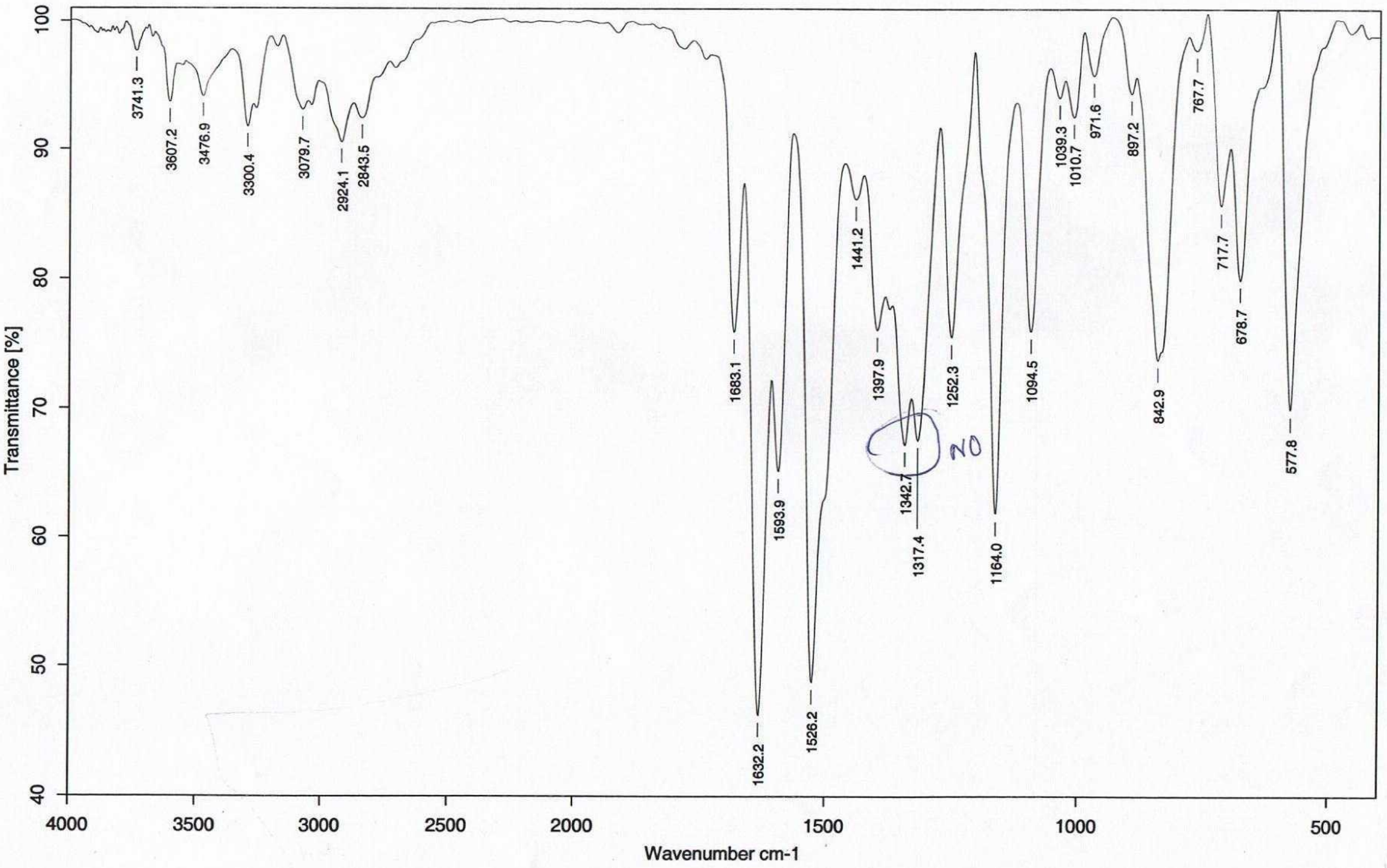

Sample : MHH-1-6/Haroon/Dr. Hina

Measured : 01/02/2017 on VECTOR22

Resolution : 4 cm-1 (10 scans)

Spectrum : MHH-1-6.0 (in D:\IRSTUDENT)

Technic : Solid

Analyst : ZA/Jamshed/M. Asif/Haroon

ƬHERMO ELECTRON ~ VISIONpro SOFTWARE V4.10

Ɔperator Name	ARSHAD ALAM.	Date of Report	2/2/2017
Ɔepartment	Analytical Laboratory TWC # 004	Time of Report	3:20:21PM
Ɔrganization	ICCBS Karachi of Universty.		
nformation	Dr.Haroon/ Dr.Hina		

Ƨcan Graph

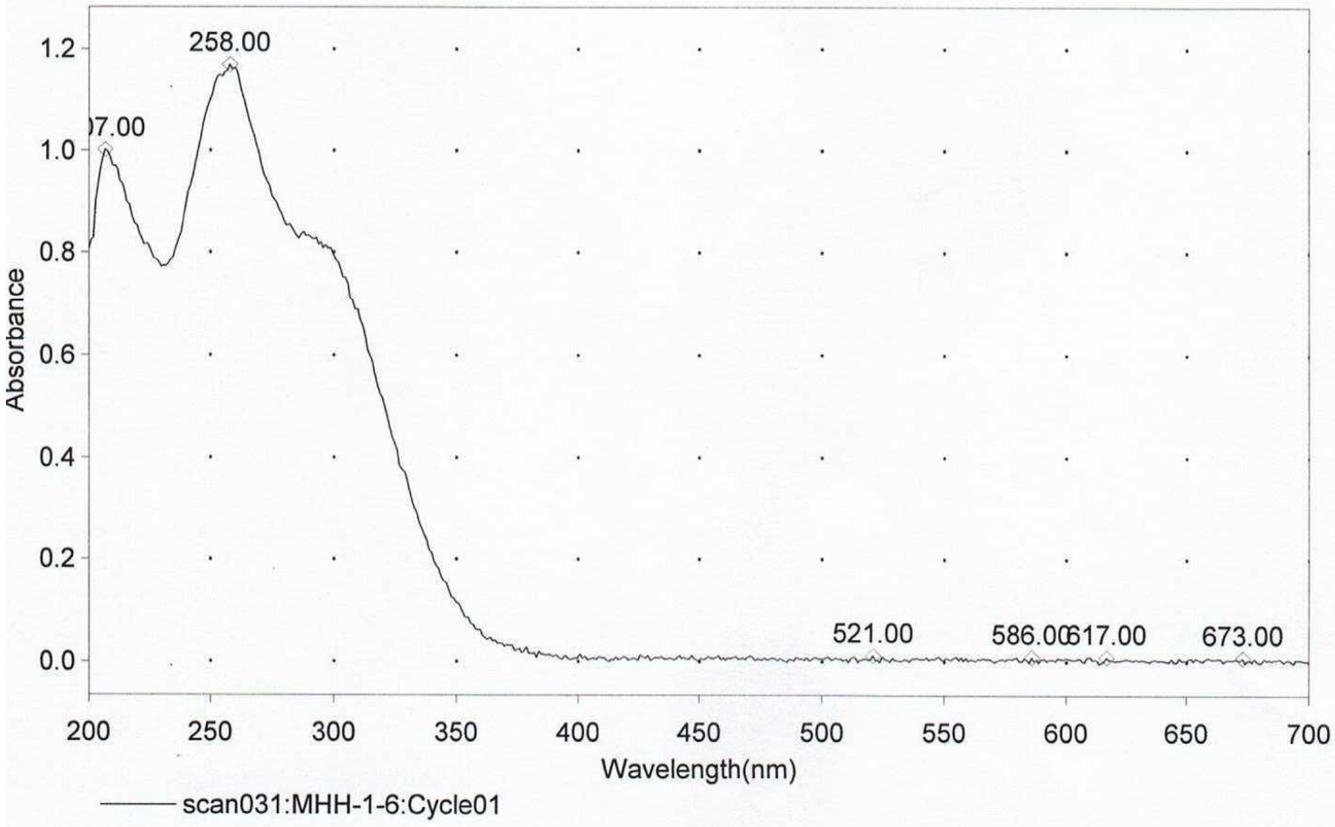

Ɽesults Table - MHH-1-6.sre,MHH-1-6,Cycle01

ım	A	Peak Pick Method	
ɂ07.00	1.001	Find 8 Peaks Above -3.0000 A	
ɂ58.00	1.169	Start Wavelength 200.00 nm	
ƽ21.00	0.012	Stop Wavelength 700.00 nm	
ƽ86.00	0.009	Sort By Wavelength	
ƽ17.00	0.009	Sensitivity	Manual
ƽ73.00	0.007	Rising Points	3
₣alling Points	3		
ʎin. Change	0.0000		
