# Supplementary material for: Sulphamethazine derivatives as immunomodulating agents: New therapeutic strategies for inflammatory diseases
Source: PLoS One. 2018 Dec 19;13(12):e0208933. doi: 10.1371/journal.pone.0208933 (PMC6300282; doi:10.1371/journal.pone.0208933)
Supplement: S24 Fig — (PDF) [file pone.0208933.s024.pdf]

Dr. Haroon / Dr. hina / MHH-I-5
ICCBS,U.O.K/
1H

AVANCE AV - III
300 MHz, LAB # 116

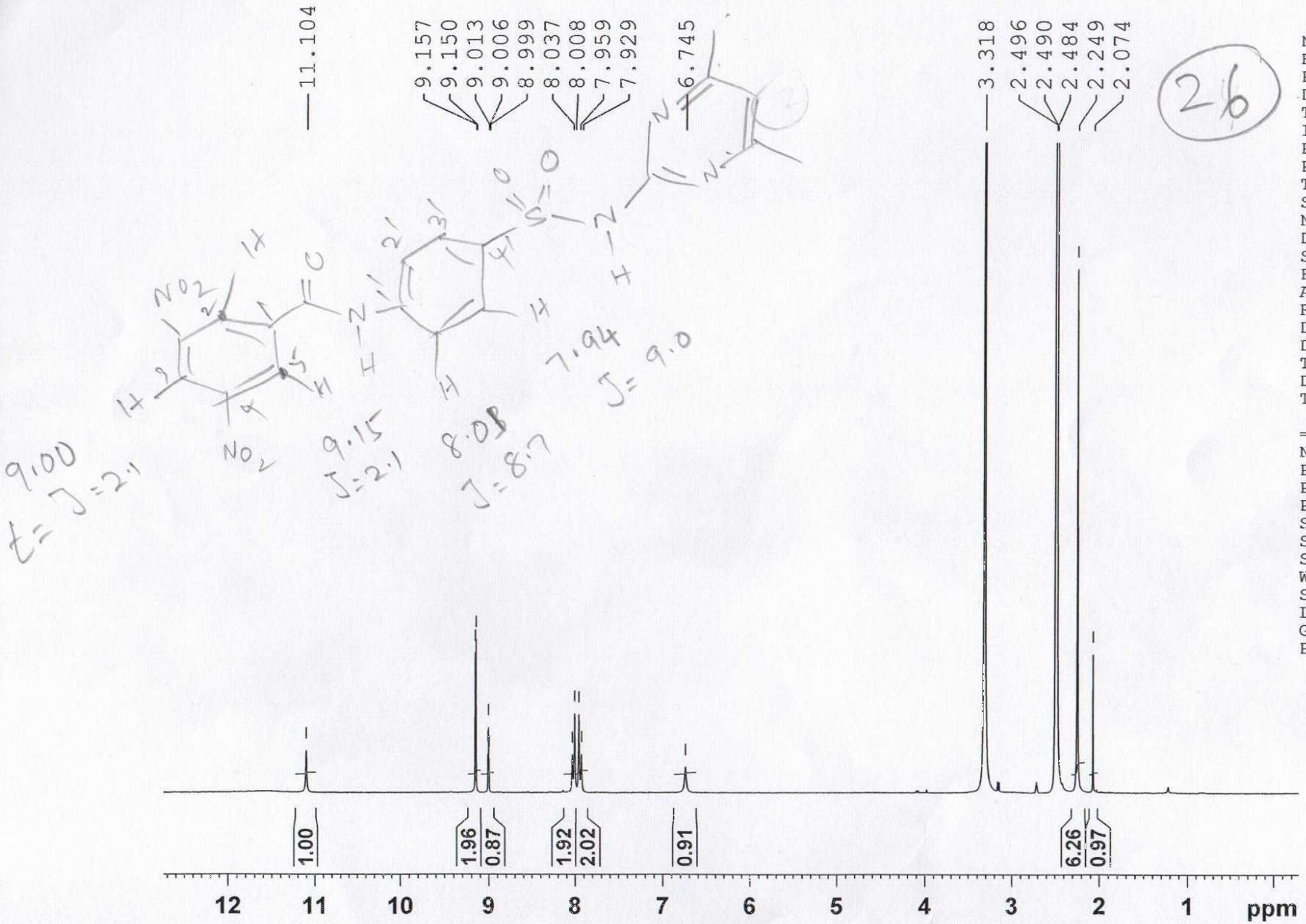

(26)

NAME	Dec9-16	
EXPNO	14	
PROCNO	1	
Date_	20161214	
Time	14.27	
INSTRUM	Spect	
PROBHD	5 mm BBO BB-1H	
PULPROG	zg30	
TD	32768	
SOLVENT	DMSO	
NS	128	
DS	0	
SWH	6009.615	Hz
FIDRES	0.183399	Hz
AQ	2.7263477	sec
RG	203	
DW	83.200	usec
DE	6.50	usec
TE	300.0	K
D1	1.50000000	sec
TD0	1	

======== CHANNEL f1 ========

NUC1	1H	
P1	12.50	usec
PL1	0.00	dB
PL1W	13.16228485	W
SFO1	300.1324010	MHz
SI	16384	
SF	300.1300039	MHz
WDW	EM	
SSB	0	
LB	0.30	Hz
GB	0	
PC	1.00	

Dr. Haroon / Dr. hina / MHH-I-5
ICCBS,U.O.K/
1H

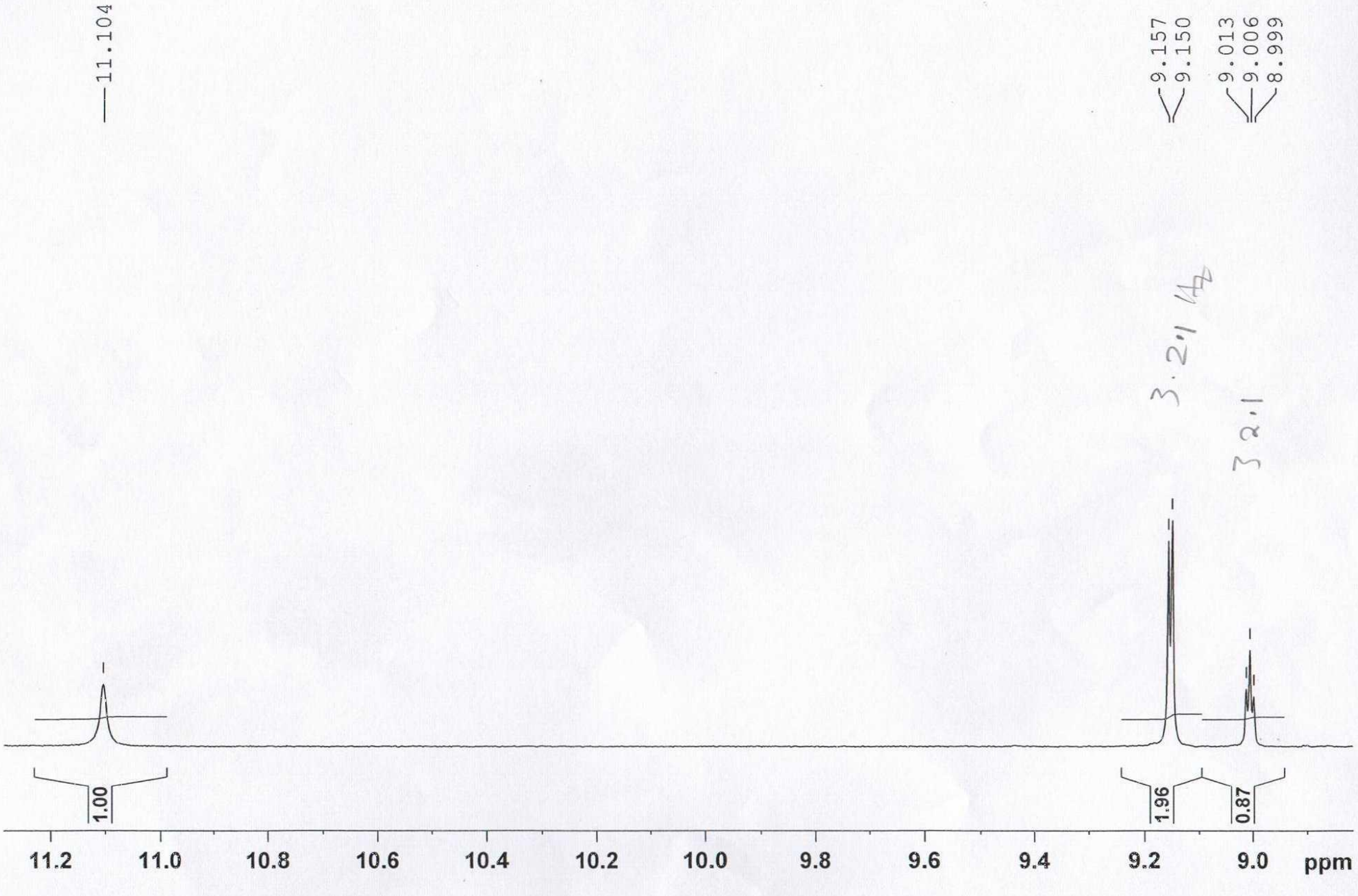

Dr. Haroon / Dr. hina / MHH-I-5
ICCBS,U.O.K/
1H

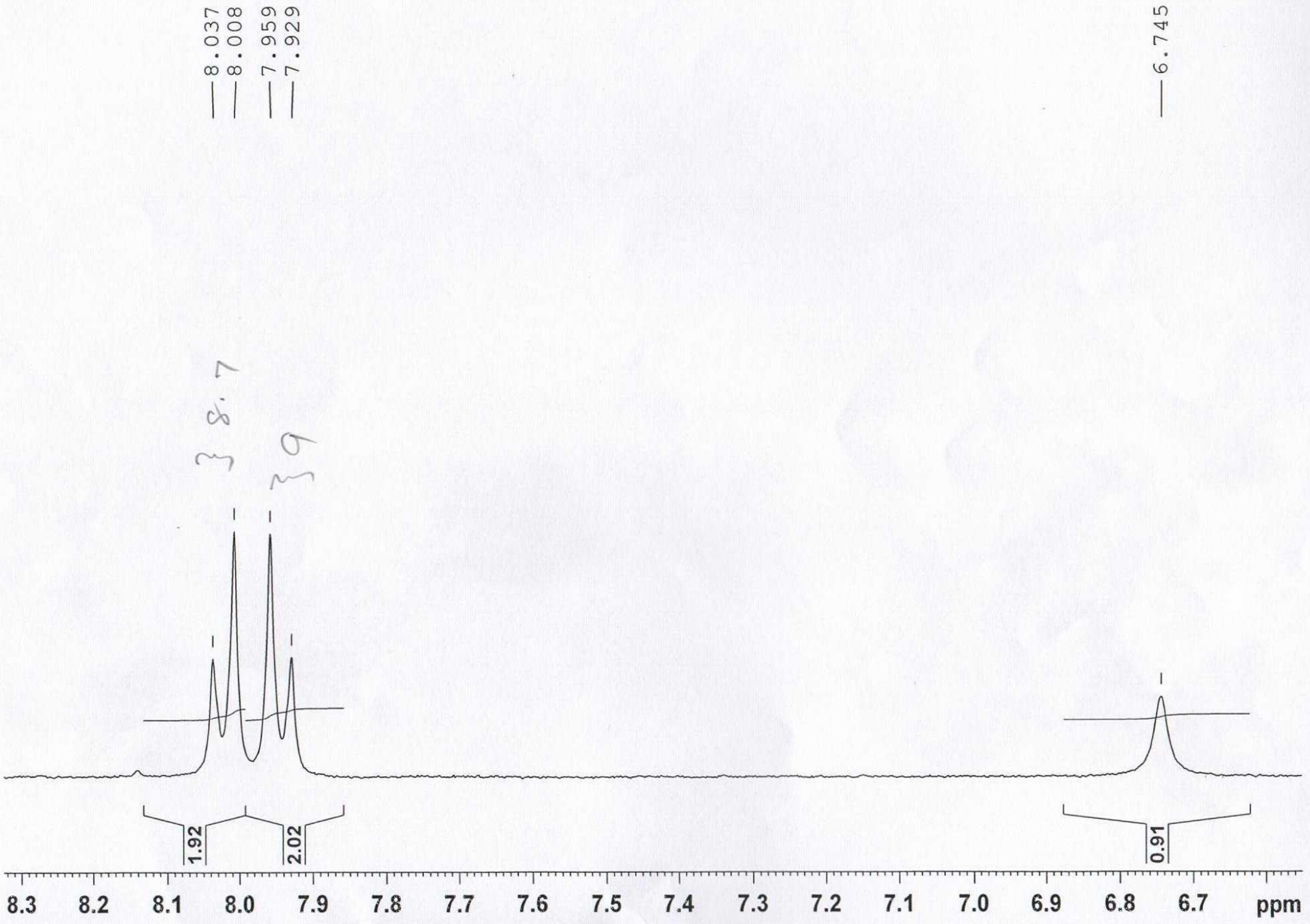

File: MHH-I-05
Sample: DR.M.H.HAROON /DR. HINA
Instrument: JEOL MS 600H-1

Date Run: 02-13-2017 (Time Run: 13:00:48)

Ionization mode: EI+

Scan: 32
Base: m/z 407; 2.3%FS TIC: 185250

R.T.: 2.75

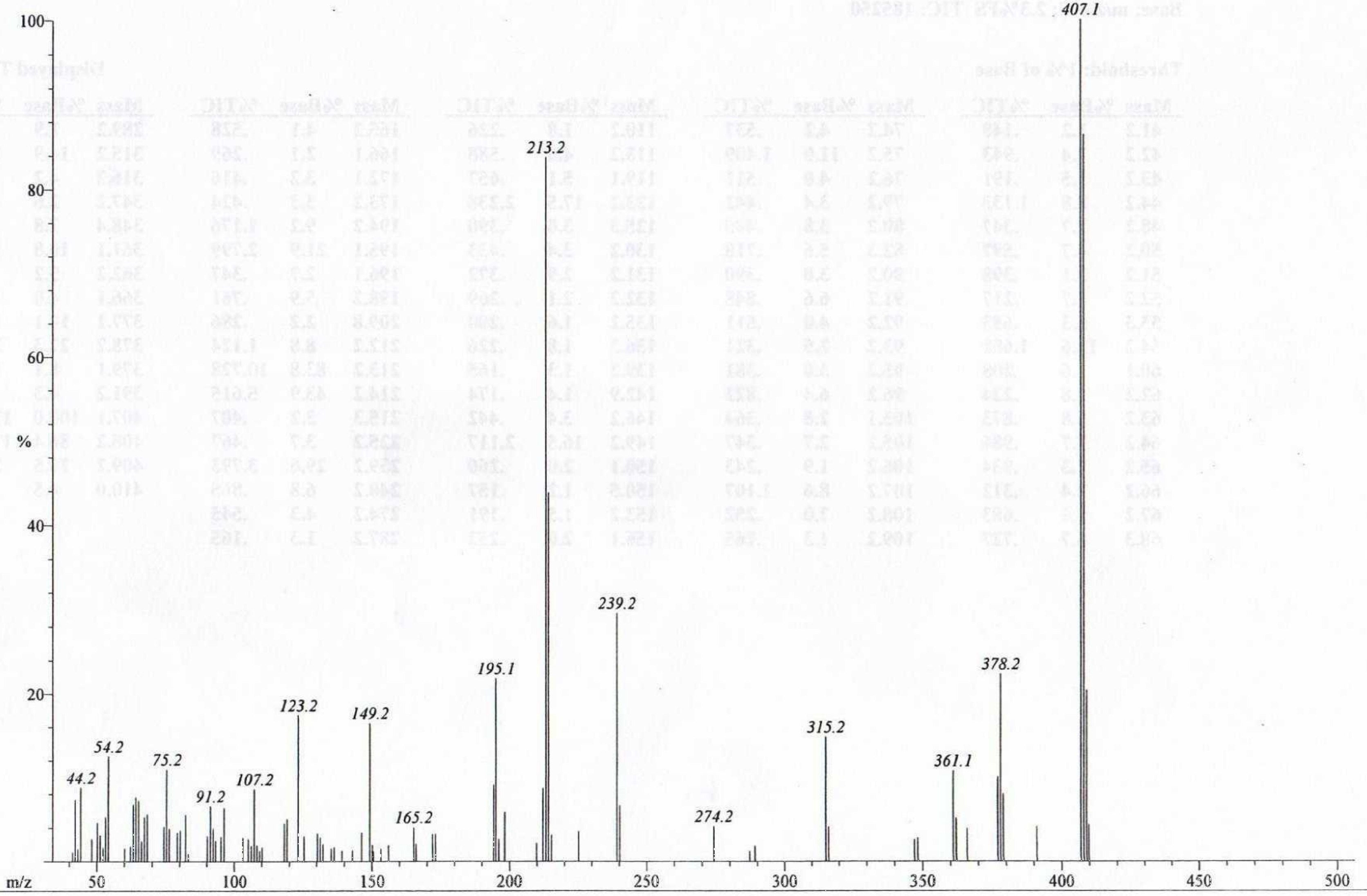

I. C.C.B.S., University of Karachi Analytical Laboratory - Pakistan

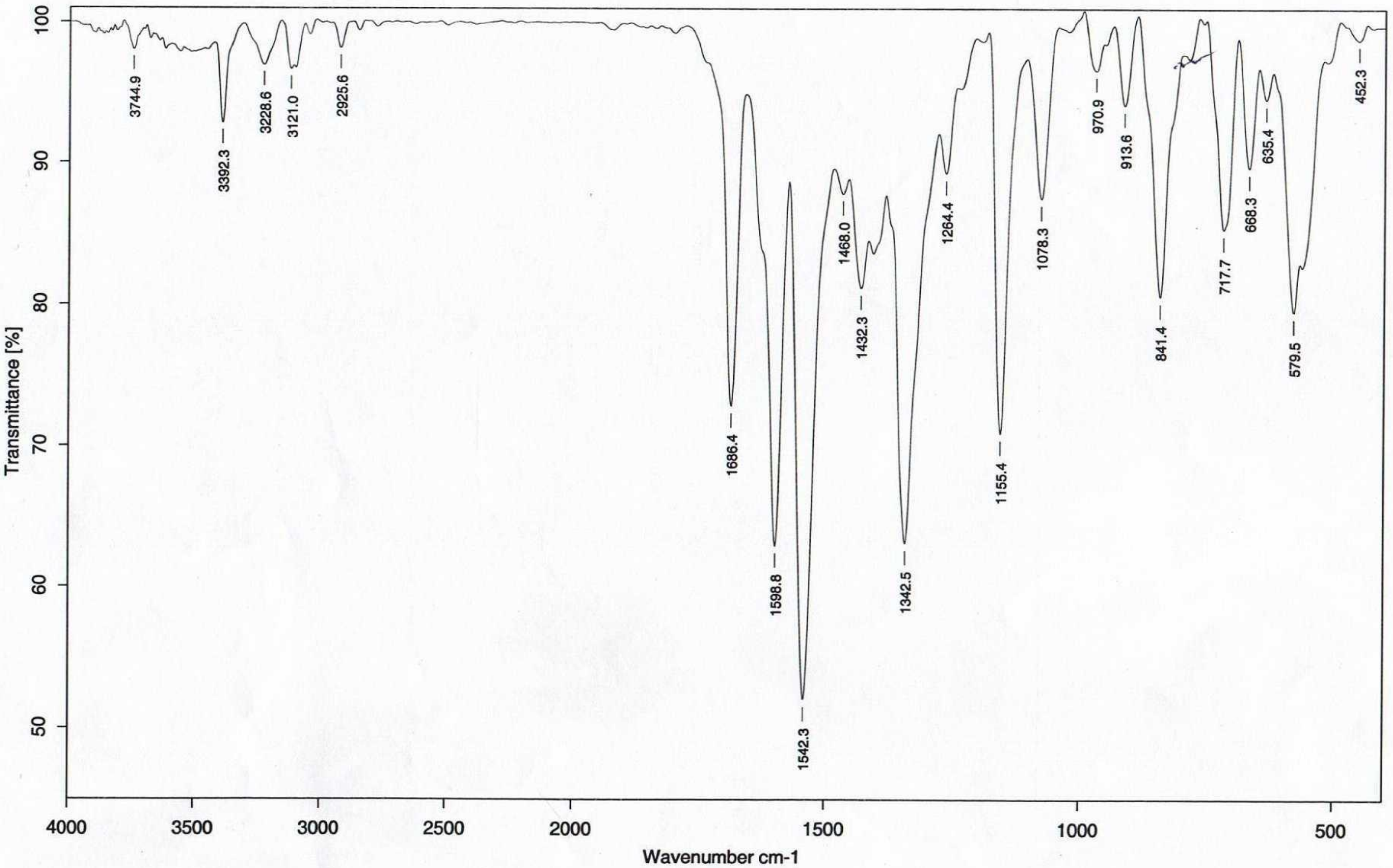

Sample : MHH-1-5/Haroon/Dr. Hina	Spectrum : MHH-1-5.0 (in D:\IRSTUDENT)
Measured : 01/02/2017 on VECTOR22	Technic : Solid
Resolution : 4 cm-1 (10 scans)	Analyst : ZA/Jamshed/M. Asif/Haroon

THERMO ELECTRON ~ VISIONpro SOFTWARE V4.10

Operator Name	ARSHAD ALAM.	Date of Report	2/2/2017
Department	Analytical Laboratory TWC # 004	Time of Report	3:35:49PM
Organization	ICCBS Karachi of Universty.		
Information	Dr.Haroon/ Dr.Hina		

Scan Graph

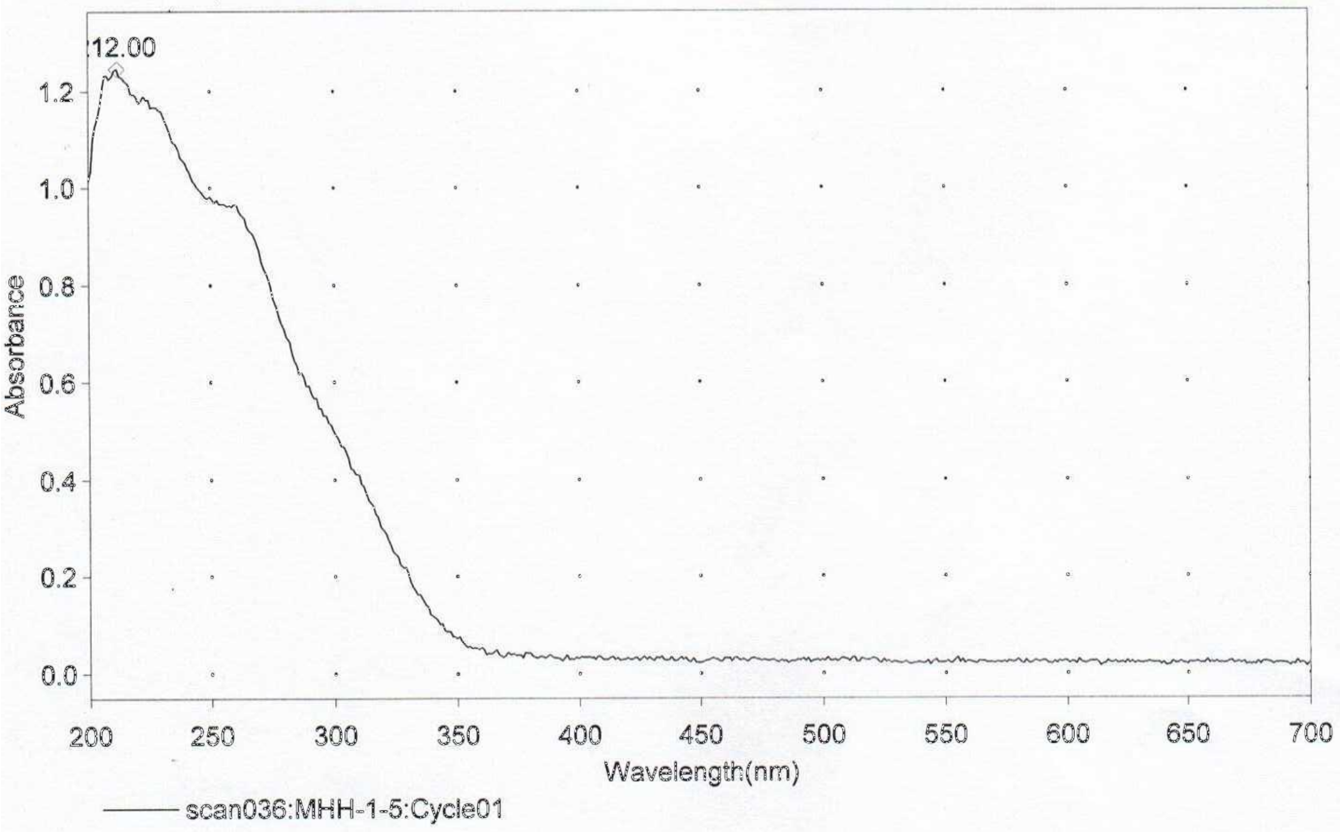

Results Table - MHH-1-5.sre,MHH-1-5,Cycle01

nm	A	**Peak Pick Method**
212.00	1.247	Find 8 Peaks Above -3.0000 A
		Start Wavelength 200.00 nm
		Stop Wavelength 700.00 nm
		Sort By Wavelength

Sensitivity Auto
