# Supplementary material for: Sulphamethazine derivatives as immunomodulating agents: New therapeutic strategies for inflammatory diseases
Source: PLoS One. 2018 Dec 19;13(12):e0208933. doi: 10.1371/journal.pone.0208933 (PMC6300282; doi:10.1371/journal.pone.0208933)
Supplement: S25 Fig — (PDF) [file pone.0208933.s025.pdf]

DR.HAROON/DR.HINA/MHH.I.14
1H

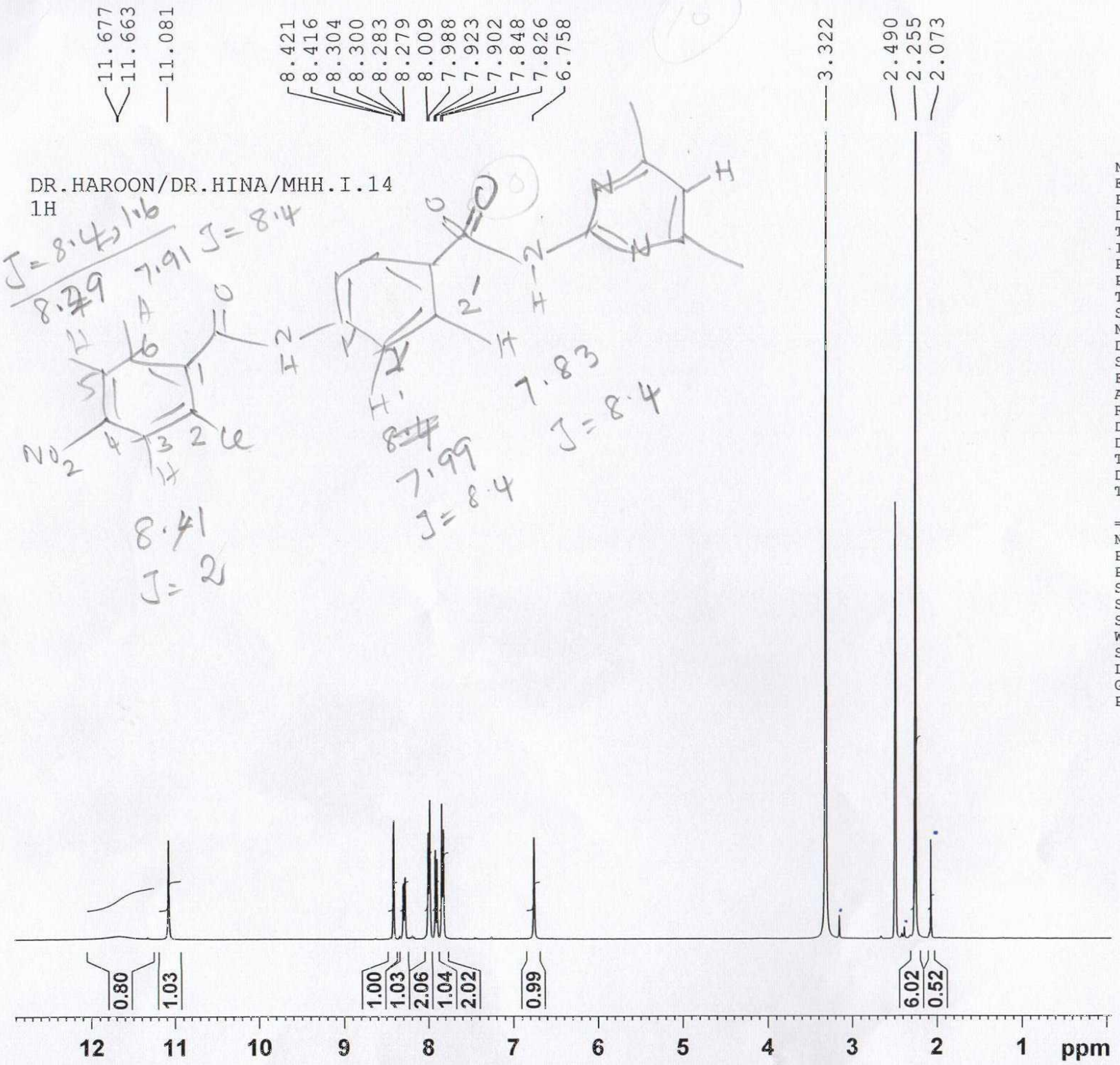

AVANCE AV-400 MHz
Lab # 115

NAME	dec28-16
EXPNO	10
PROCNO	1
Date_	20161228
Time	13.36
INSTRUM	spect
PROBHD	5 mm SEI 1H-13
PULPROG	zg30
TD	65536
SOLVENT	DMSO
NS	64
DS	0
SWH	8012.820 Hz
FIDRES	0.122266 Hz
AQ	4.0894966 sec
RG	362
DW	62.400 usec
DE	6.50 usec
TE	300.0 K
D1	2.00000000 sec
TD0	1

======== CHANNEL f1 ========

NUC1	1H
P1	10.80 usec
PL1	3.00 dB
SFO1	400.0332002 MHz
SI	32768
SF	400.0300041 MHz
WDW	EM
SSB	0
LB	0.30 Hz
GB	0
PC	1.00

DR.HAROON/DR.HINA/MHH.I.14
1H

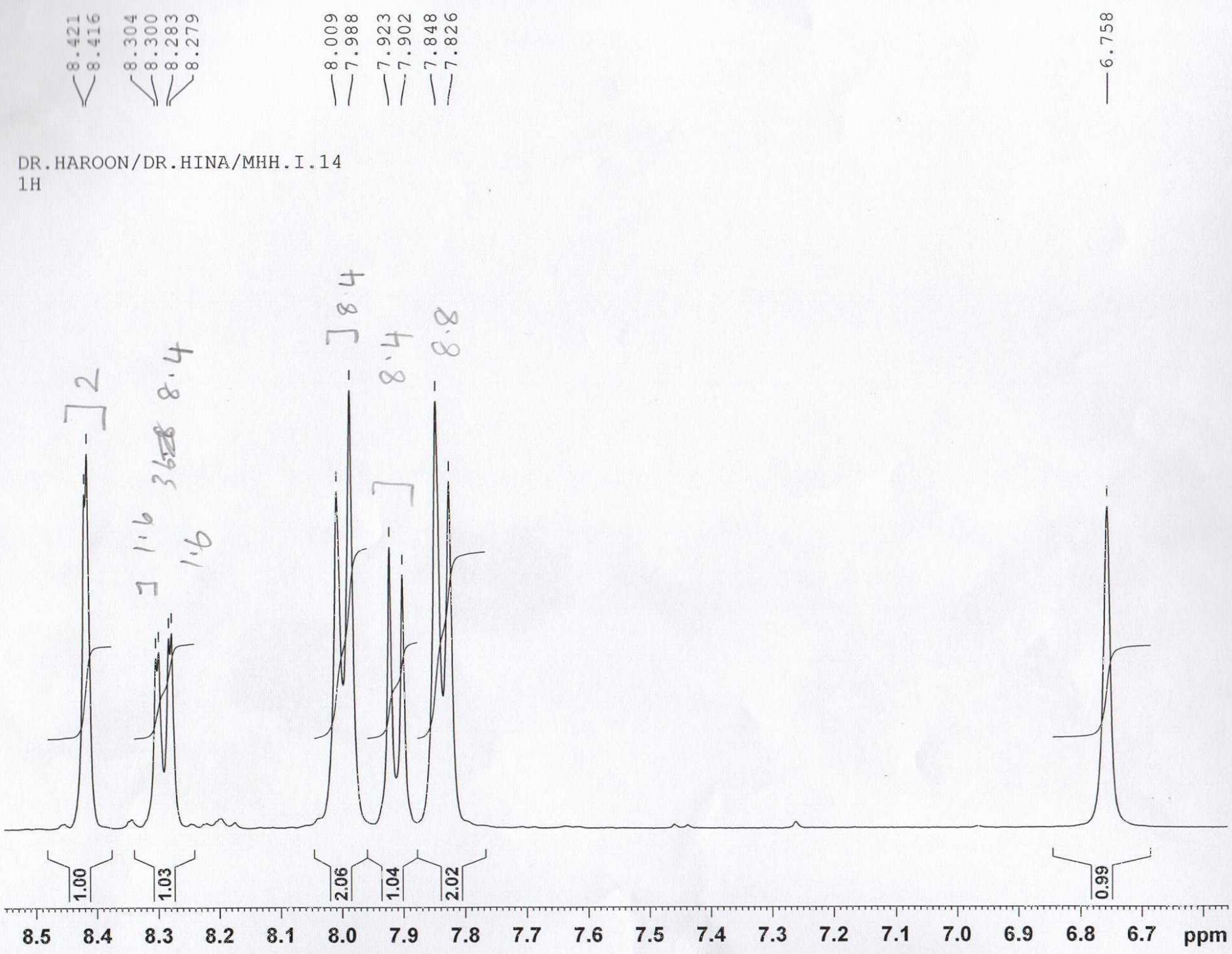

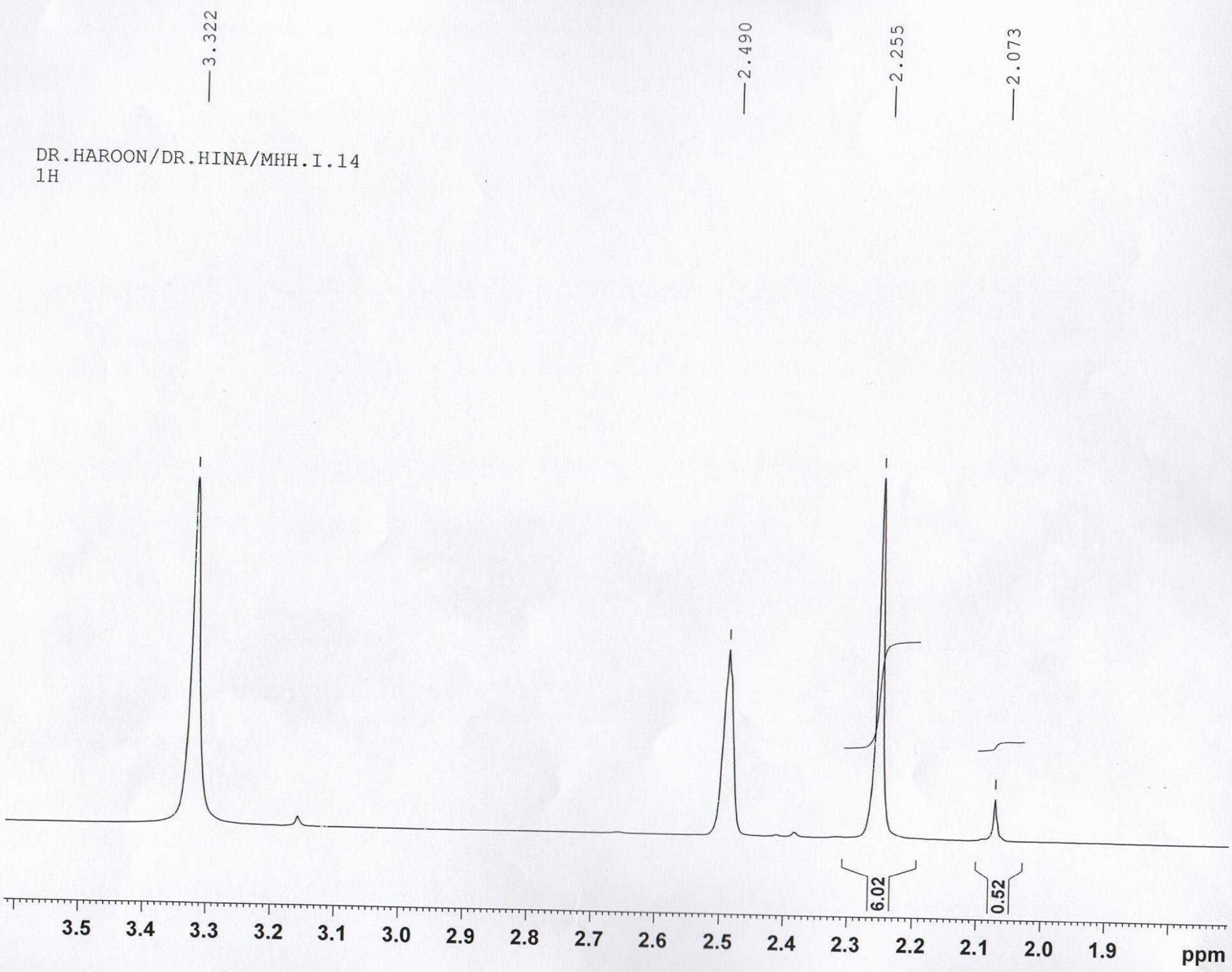

3.322
2.490
2.255
2.073
DR.HAROON/DR.HINA/MHH.I.14
1H
6.02
0.52
3.5
3.4
3.3
3.2
3.1
3.0
2.9
2.8
2.7
2.6
2.5
2.4
2.3
2.2
2.1
2.0
1.9
ppm

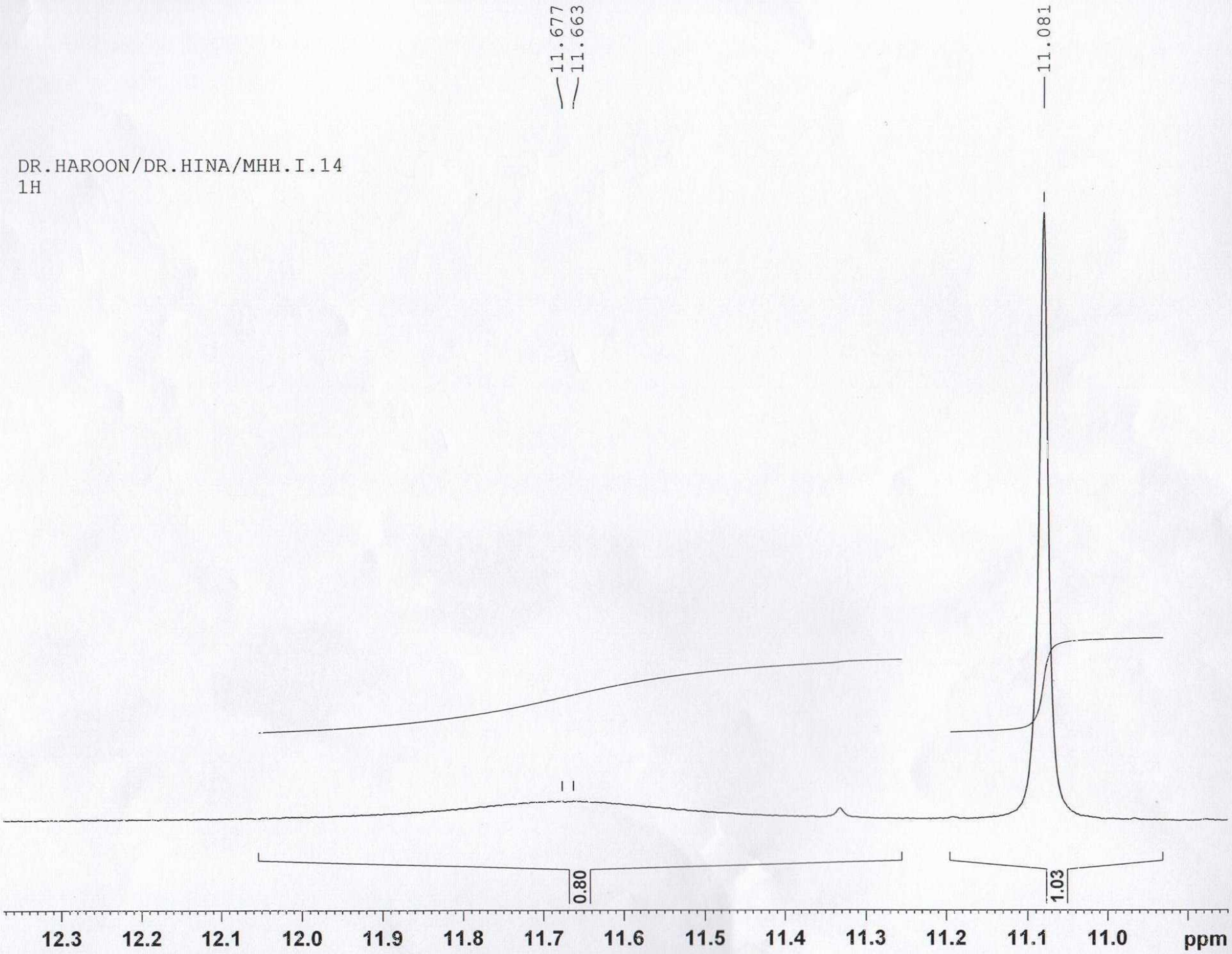
DR.HAROON/DR.HINA/MHH.I.14
1H
11.677
11.663
11.081
0.80
1.03
12.3
12.2
12.1
12.0
11.9
11.8
11.7
11.6
11.5
11.4
11.3
11.2
11.1
11.0
ppm

[illegible]

2/9/2017 12:09:20 PM

File: MHH-I-14- **Date Run: 02-09-2017 (Time Run: 11:51:25)**

Sample: DR.M.H.HAROON /DR. HINA

Instrument: JEOL MS 600H-1

Ionization mode: EI+

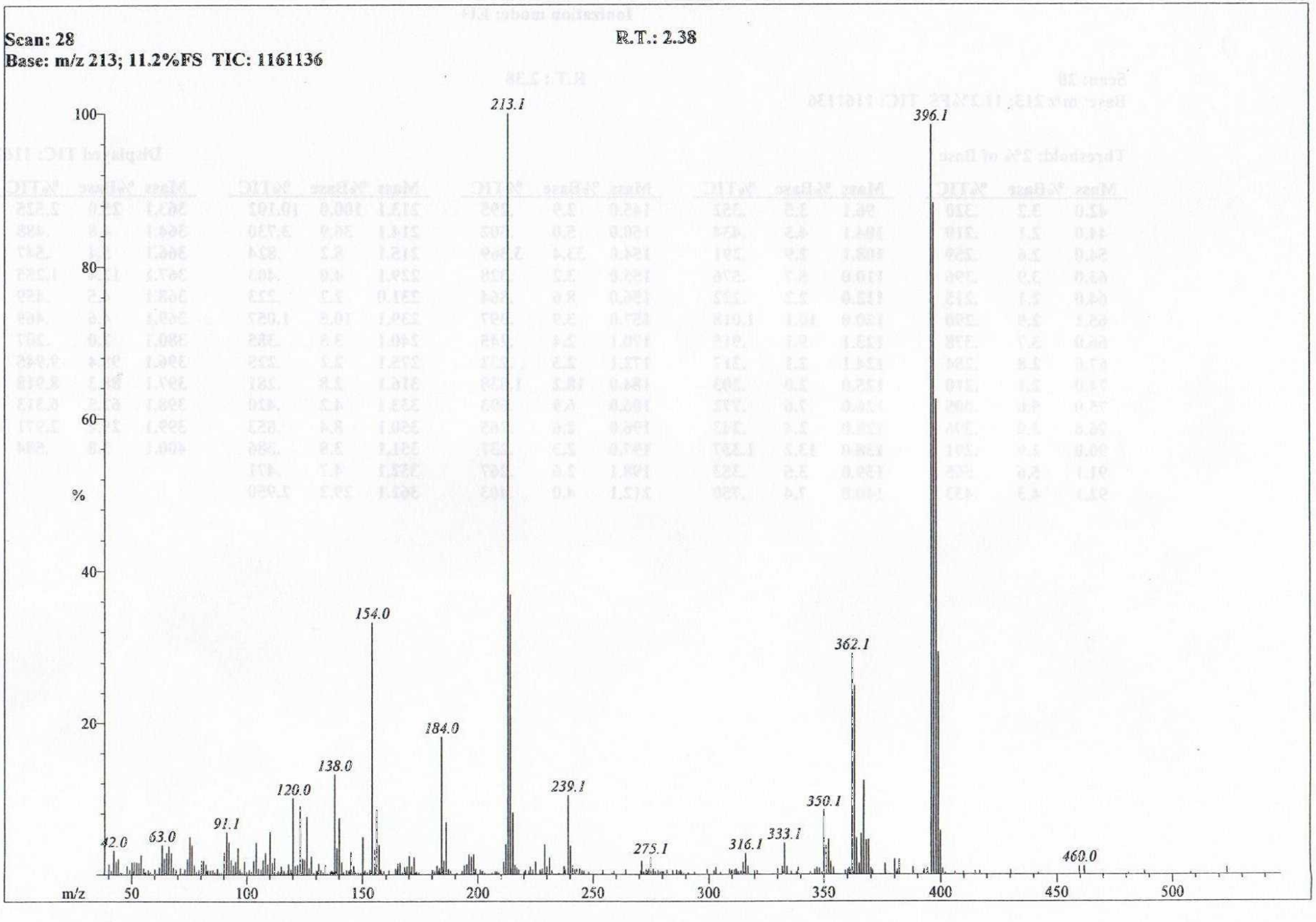

I. C.C.B.S., University of Karachi Analytical Laboratory - Pakistan

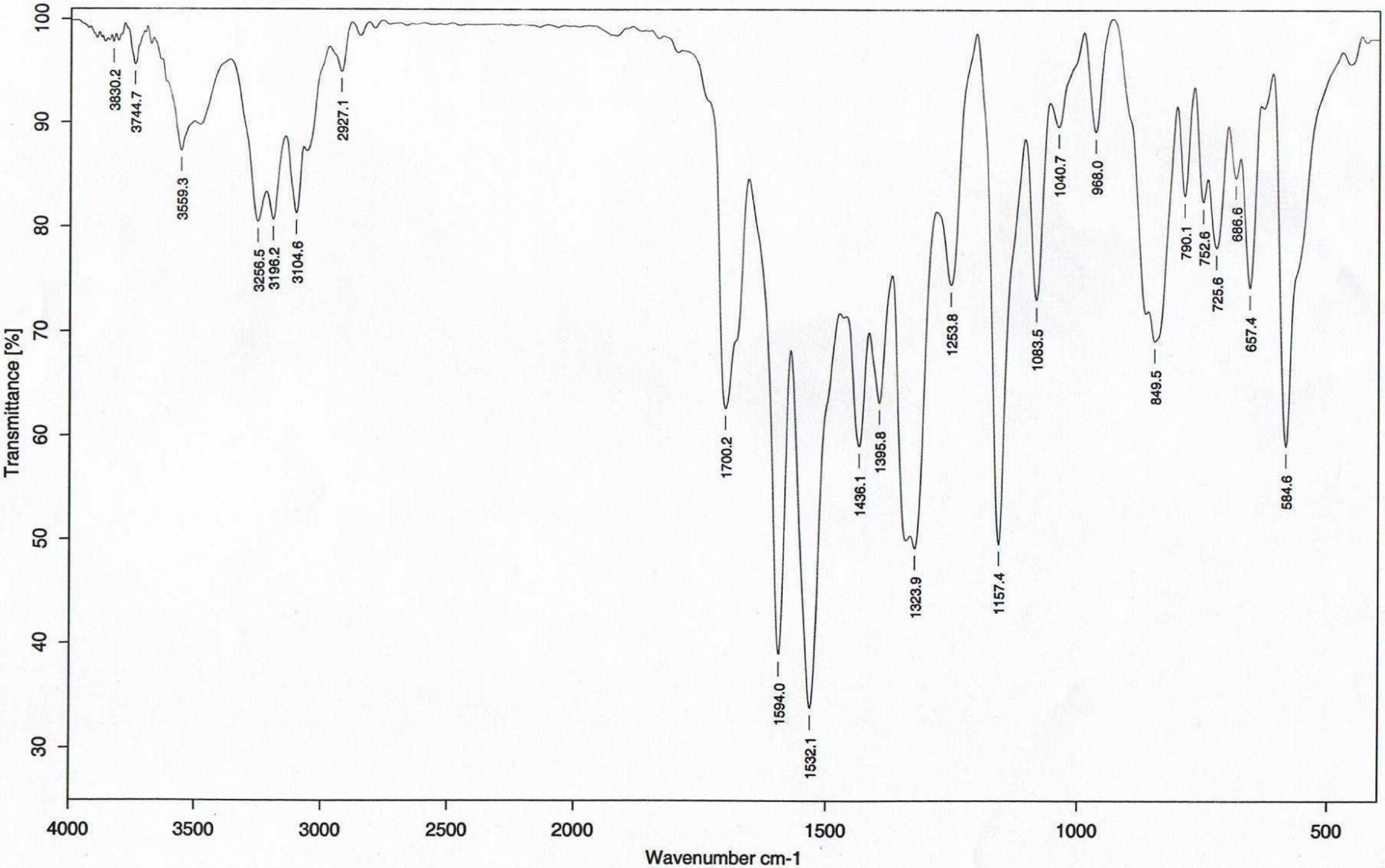

Sample : MHH-1-7/Haroon/Dr. Hina

Measured : 31/01/2017 on VECTOR22

Resolution : 4 cm-1 (10 scans)

Spectrum : MHH-1-14.0 (in D:\IRSTUDENT)

Technic : Solid

Analyst : Zubair Ahmad/ Jamshed/M. Asif/

THERMO ELECTRON ~ VISIONpro SOFTWARE V4.10

Operator Name	ARSHAD ALAM.	Date of Report	2/1/2017
Department	Analytical Laboratory TWC # 004	Time of Report	3:12:51PM
Organization	ICCBS Karachi of Universty.		
Information	Dr Haroon/Dr Hina		

Scan Graph

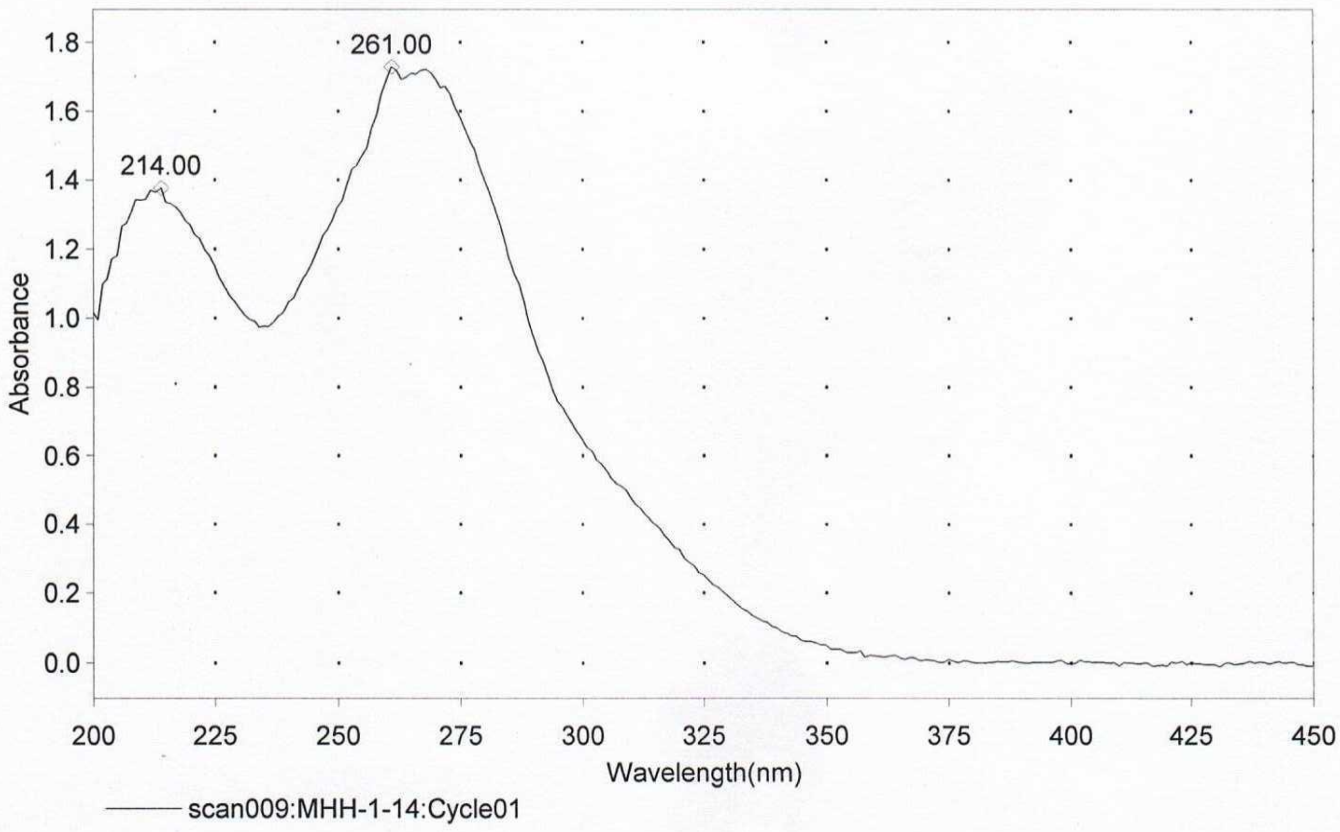

Results Table - MHH-1-14.sre,MHH-1-14,Cycle01

nm	A	**Peak Pick Method**
214.00	1.375	Find 8 Peaks Above -3.0000 A
261.00	1.729	Start Wavelength 200.00 nm
		Stop Wavelength 450.00 nm
		Sort By Wavelength

Sensitivity Auto
