# Supplementary material for: Sulphamethazine derivatives as immunomodulating agents: New therapeutic strategies for inflammatory diseases
Source: PLoS One. 2018 Dec 19;13(12):e0208933. doi: 10.1371/journal.pone.0208933 (PMC6300282; doi:10.1371/journal.pone.0208933)
Supplement: S28 Fig — (PDF) [file pone.0208933.s028.pdf]

M. Haron / Dr. Hina / MHH-I-15
1H

AVANCE AV - III
300 MHz, LAB # 116

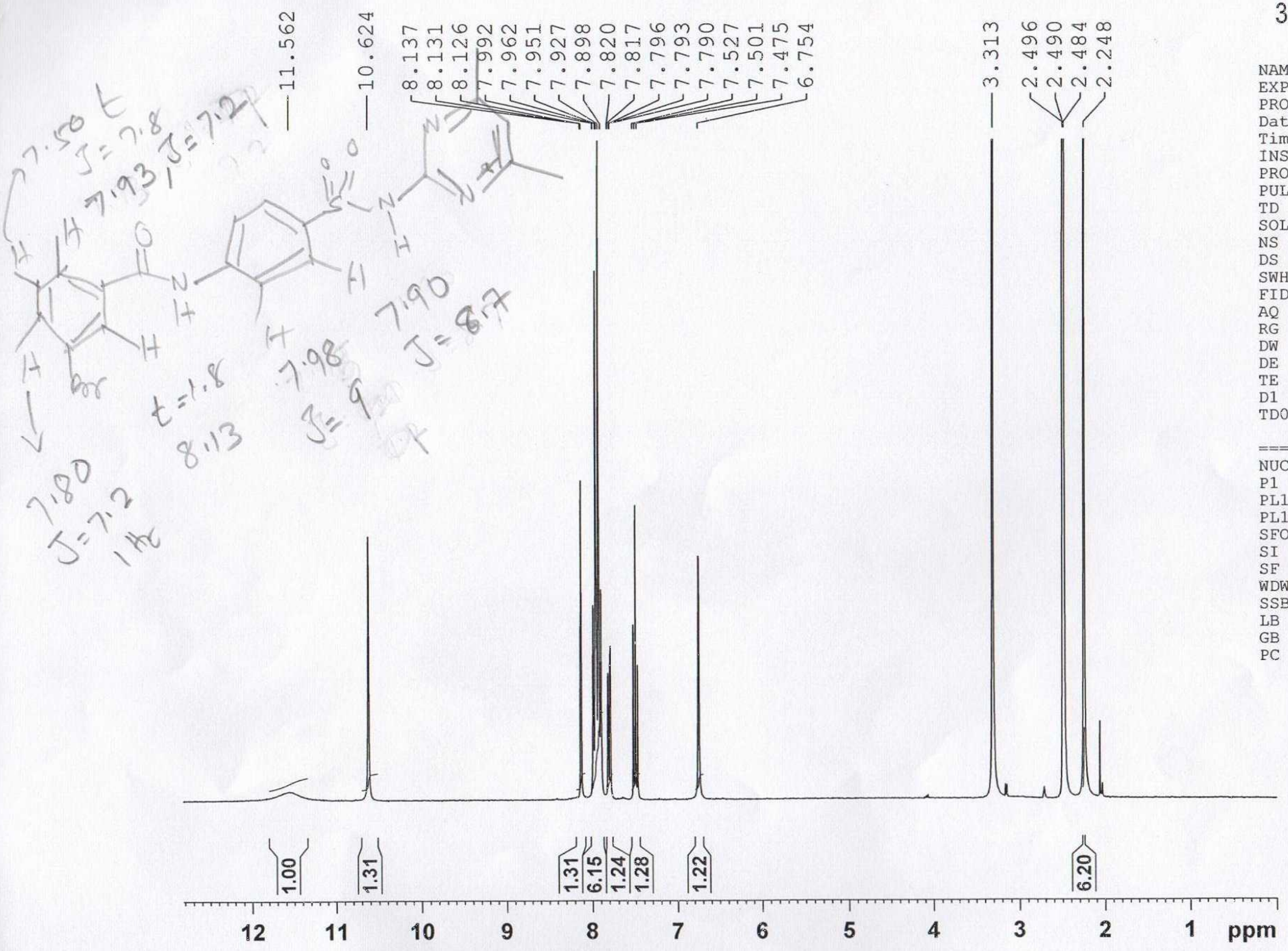

NAME	Dec29-16
EXPNO	5
PROCNO	1
Date_	20161229
Time	14.31
INSTRUM	Spect
PROBHD	5 mm BBO BB-1H
PULPROG	zg30
TD	32768
SOLVENT	DMSO
NS	64
DS	0
SWH	6009.615 Hz
FIDRES	0.183399 Hz
AQ	2.7263477 sec
RG	203
DW	83.200 usec
DE	6.50 usec
TE	300.0 K
D1	1.50000000 sec
TD0	1

======== CHANNEL f1 ========

NUC1	1H
P1	12.50 usec
PL1	0.00 dB
PL1W	13.16228485 W
SFO1	300.1324010 MHz
SI	16384
SF	300.1300040 MHz
WDW	EM
SSB	0
LB	0.30 Hz
GB	0
PC	1.00

M. Haron / Dr. Hina / MHH-I-15
1H

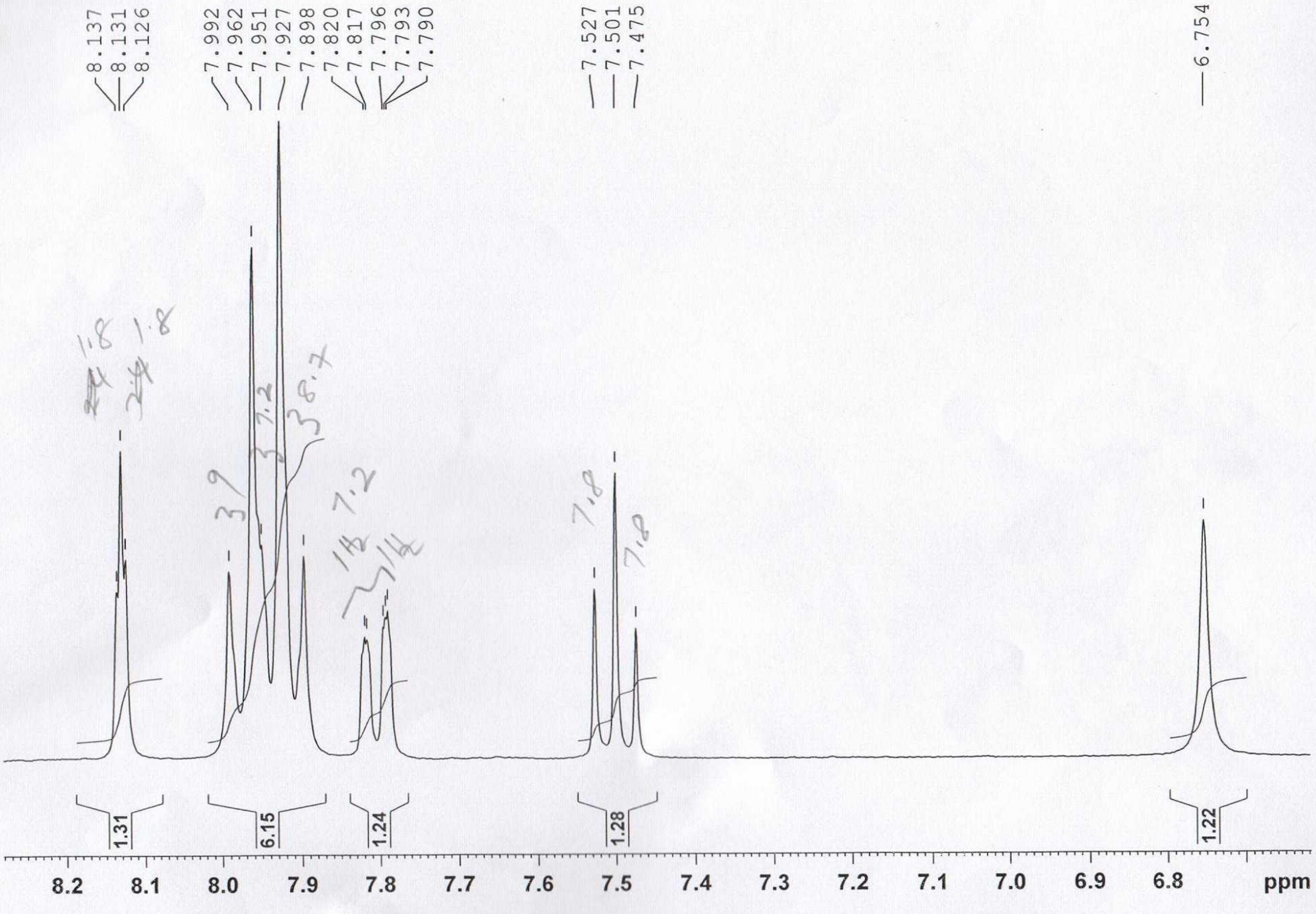

M. Haron / Dr. Hina / MHH-I-15
1H

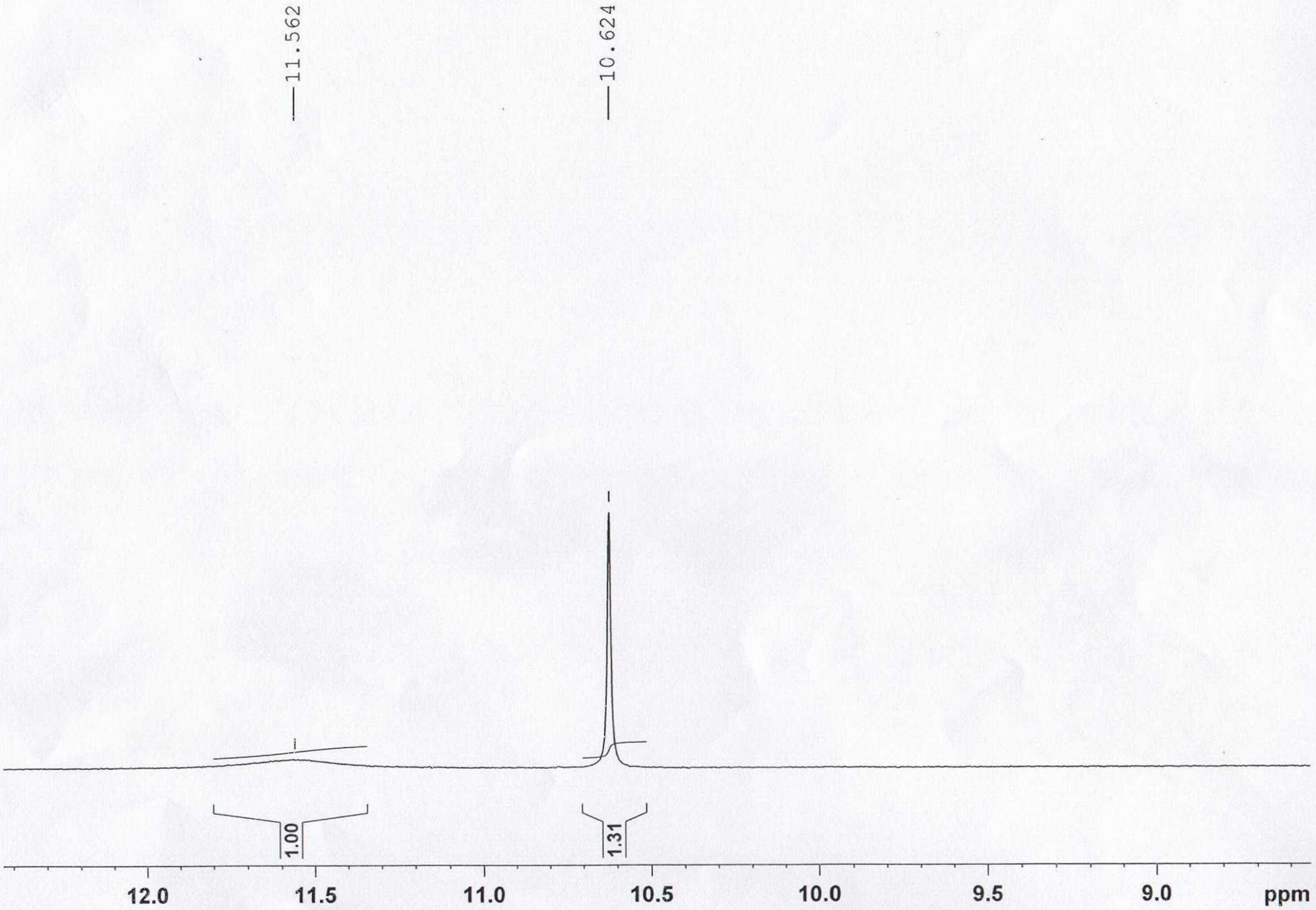

[illegible]
2/7/2017 3:48:52 PM

File: MHH-I-15
Sample: DR.M.H.HAROON /DR. HINA
Instrument: JEOL MS 600H-1

Date Run: 02-07-2017 (Time Run: 15:38:42)

Ionization mode: EI+

Scan: 28
Base: m/z 397; 24.5%FS TIC: 1793510

R.T.: 2.38

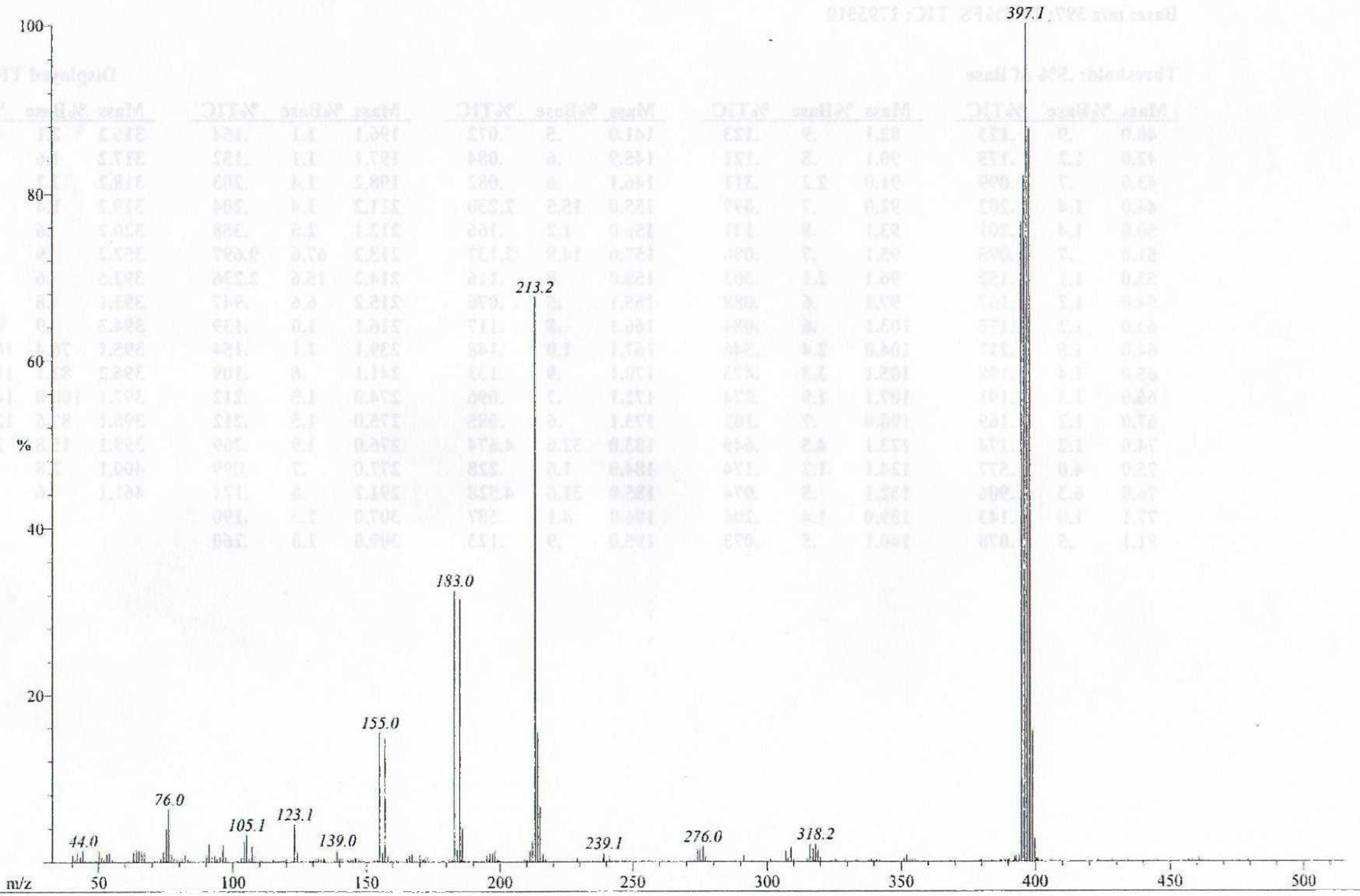

I. C.C.B.S., University of Karachi Analytical Laboratory - Pakistan

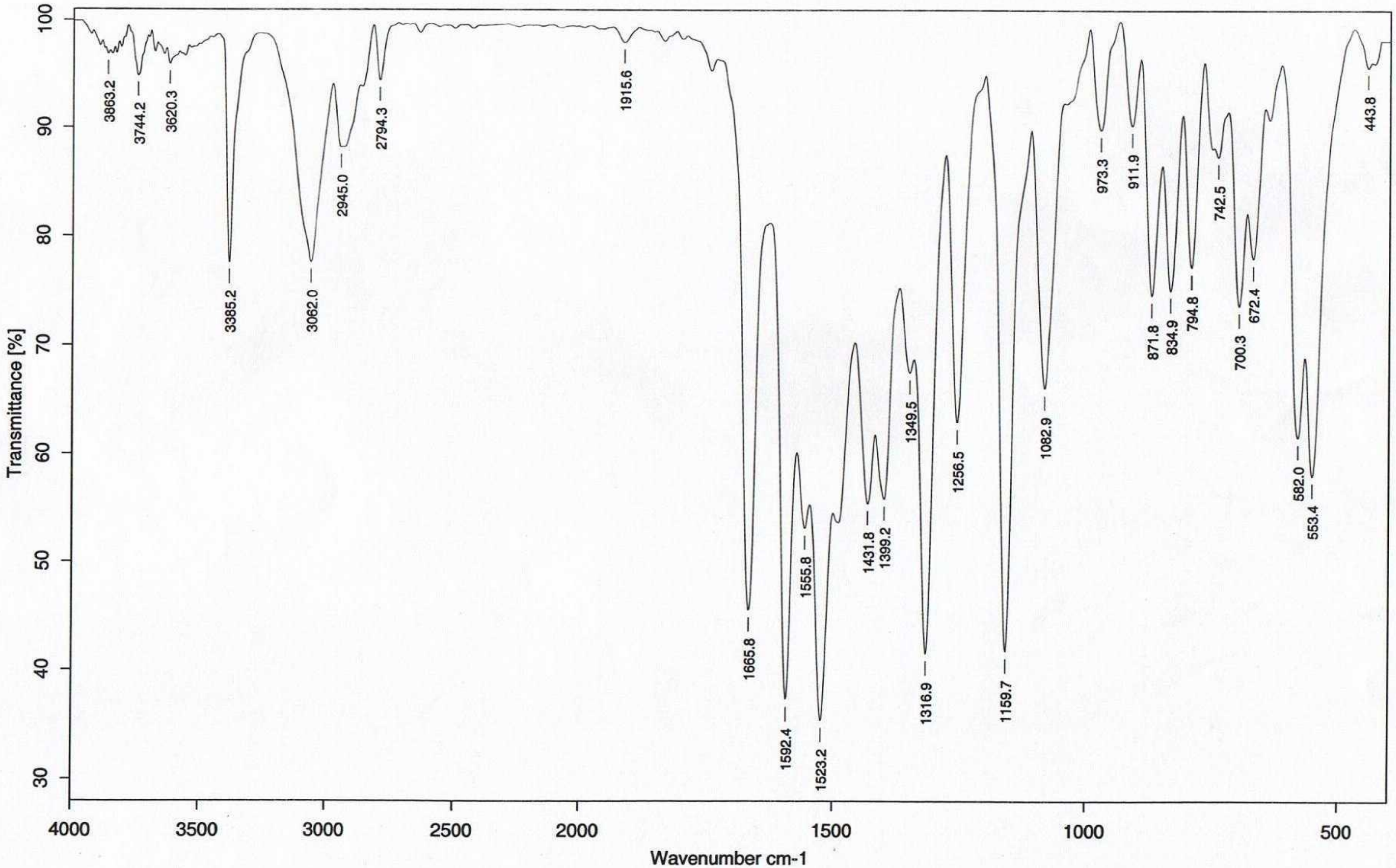

Sample : MHH-1-15/Haroon/Dr. Hina	Spectrum : MHH-1-15.0 (in D:\IRSTUDENT)
Measured : 31/01/2017 on VECTOR22	Technic : Solid
Resolution : 4 cm-1 (10 scans)	Analyst : Zubair Ahmad/ Jamshed/M. Asif/

THERMO ELECTRON ~ VISIONpro SOFTWARE V4.10

Operator Name	ARSHAD ALAM.	Date of Report	2/1/2017
Department	Analytical Laboratory TWC # 004	Time of Report	3:56:54PM
Organization	ICCBS Karachi of Universty.		
Information	Dr Haroon/Dr Hina		

Scan Graph

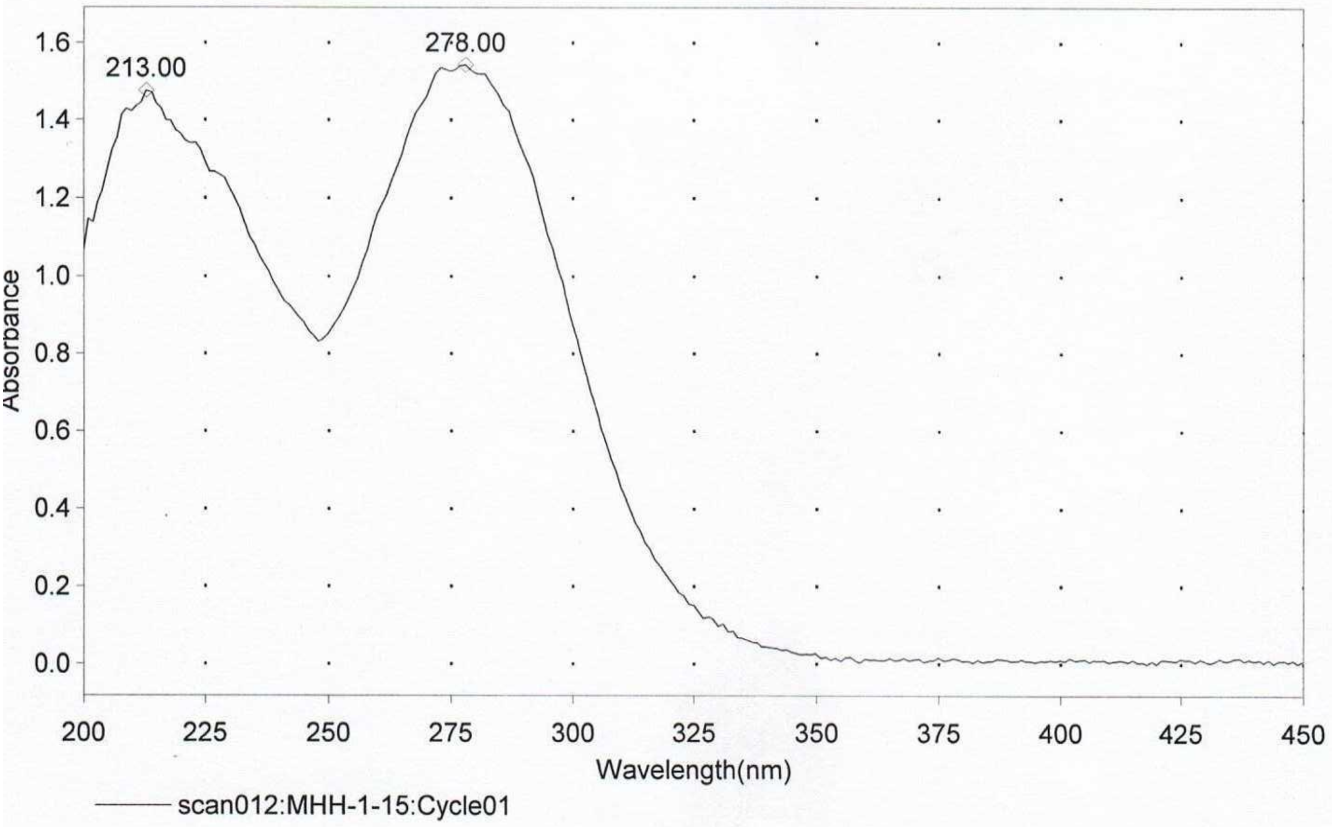

Results Table - MHH-1-15.sre,MHH-1-15,Cycle01

nm	A	**Peak Pick Method**
213.00	1.476	Find 8 Peaks Above -3.0000 A
278.00	1.543	Start Wavelength 200.00 nm
		Stop Wavelength 450.00 nm
		Sort By Wavelength

Sensitivity Auto
