# Supplementary material for: Sulphamethazine derivatives as immunomodulating agents: New therapeutic strategies for inflammatory diseases
Source: PLoS One. 2018 Dec 19;13(12):e0208933. doi: 10.1371/journal.pone.0208933 (PMC6300282; doi:10.1371/journal.pone.0208933)
Supplement: S29 Fig — (PDF) [file pone.0208933.s029.pdf]

AVANCE AV-400 MHz
Lab # 115

DR.HAROON/DR.HINA/MHH.I.34
1H

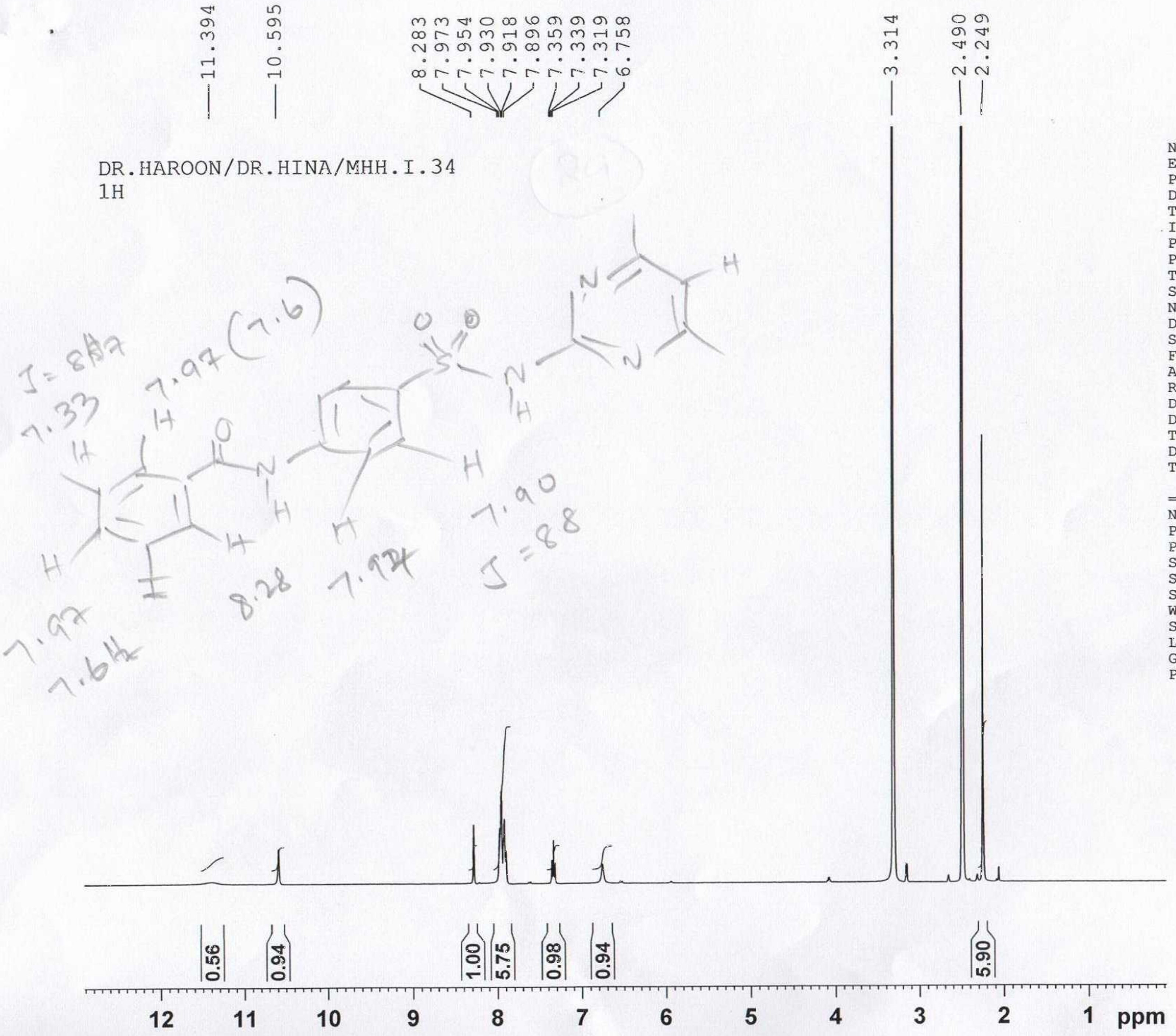

NAME	jan05-17
EXPNO	5
PROCNO	1
Date_	20170105
Time	11.44
INSTRUM	spect
PROBHD	5 mm SEI 1H-13
PULPROG	zg30
TD	65536
SOLVENT	DMSO
NS	64
DS	0
SWH	8012.820 Hz
FIDRES	0.122266 Hz
AQ	4.0894966 sec
RG	362
DW	62.400 usec
DE	6.50 usec
TE	300.0 K
D1	2.00000000 sec
TD0	1

======== CHANNEL f1 ========

NUC1	1H
P1	10.80 usec
PL1	3.00 dB
SFO1	400.0332002 MHz
SI	32768
SF	400.0300041 MHz
WDW	EM
SSB	0
LB	0.30 Hz
GB	0
PC	1.00

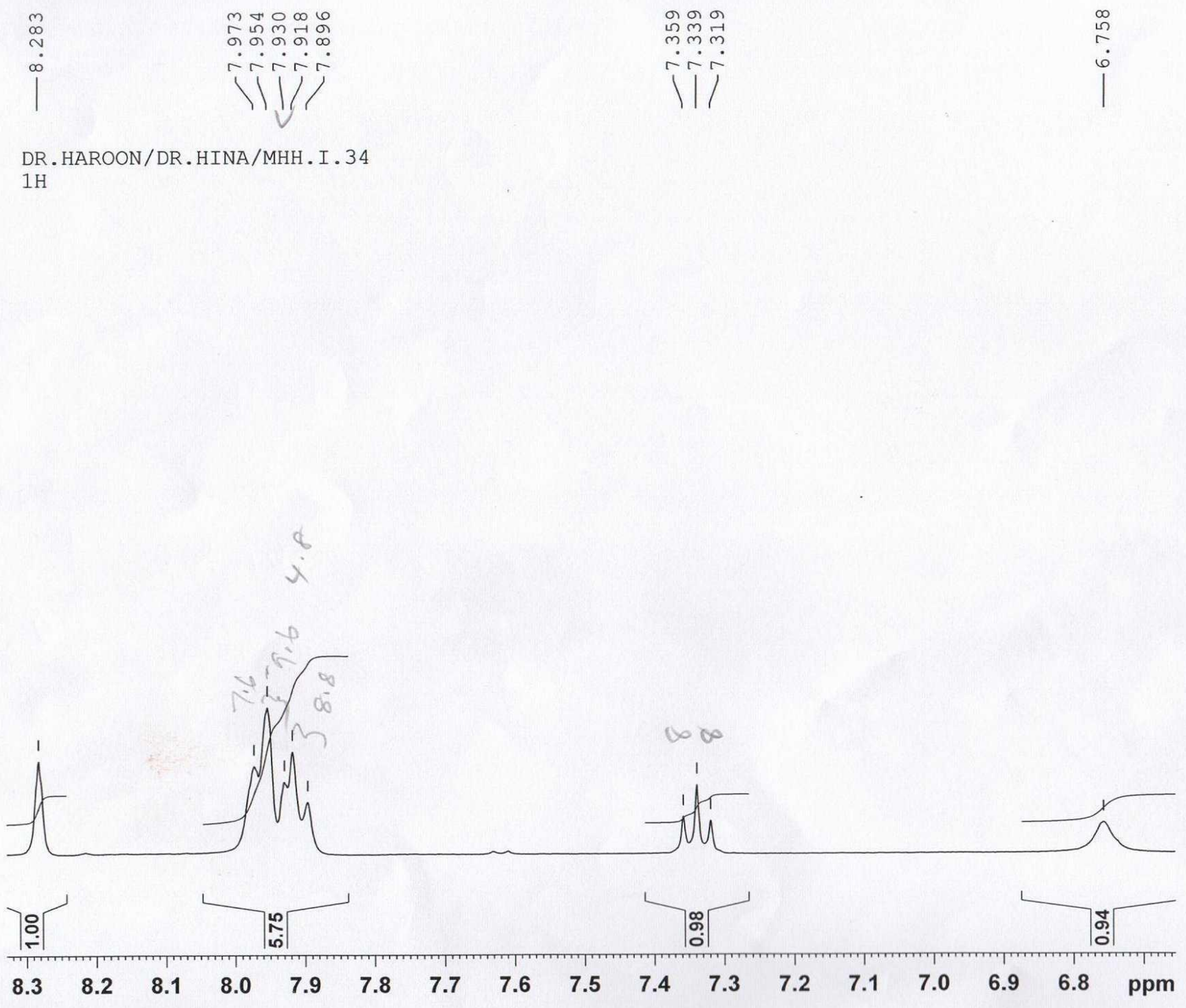
8.283
7.973
7.954
7.930
7.918
7.896
7.359
7.339
7.319
6.758
DR.HAROON/DR.HINA/MHH.I.34
1H
1.00
5.75
0.98
0.94
8.3 8.2 8.1 8.0 7.9 7.8 7.7 7.6 7.5 7.4 7.3 7.2 7.1 7.0 6.9 6.8 ppm

File: MHH-I-34
Sample: DR.M.H.HAROON /DR. IQBAL
Instrument: JEOL MS 600H-1

Date Run: 02-10-2017 (Time Run: 12:45:00)

Ionization mode: EI+

Scan: 13
Base: m/z 444; 99.5%FS TIC: 4661970

R.T.: 1.07

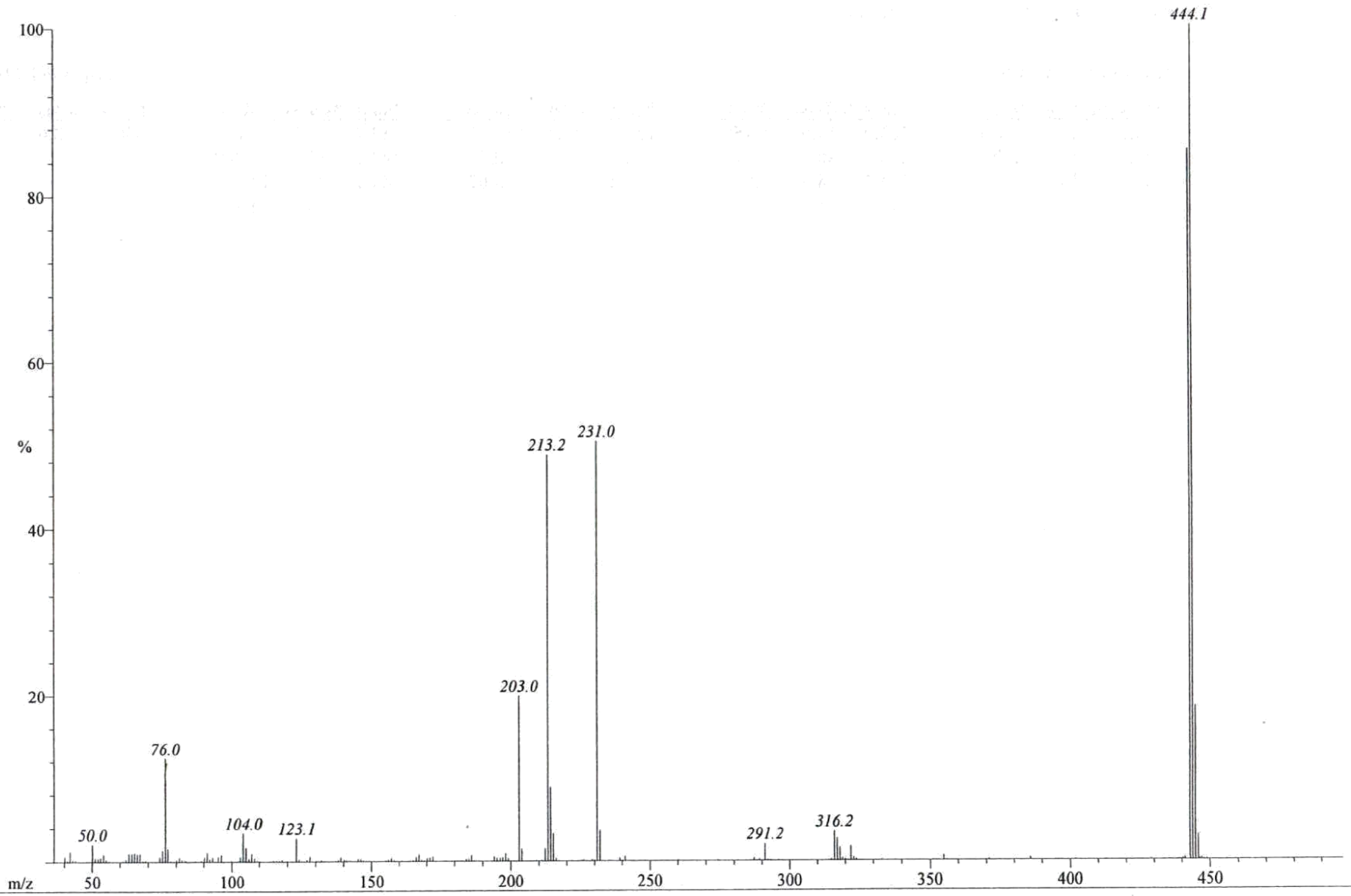

I. C.C.B.S., University of Karachi Analytical Laboratory - Pakistan

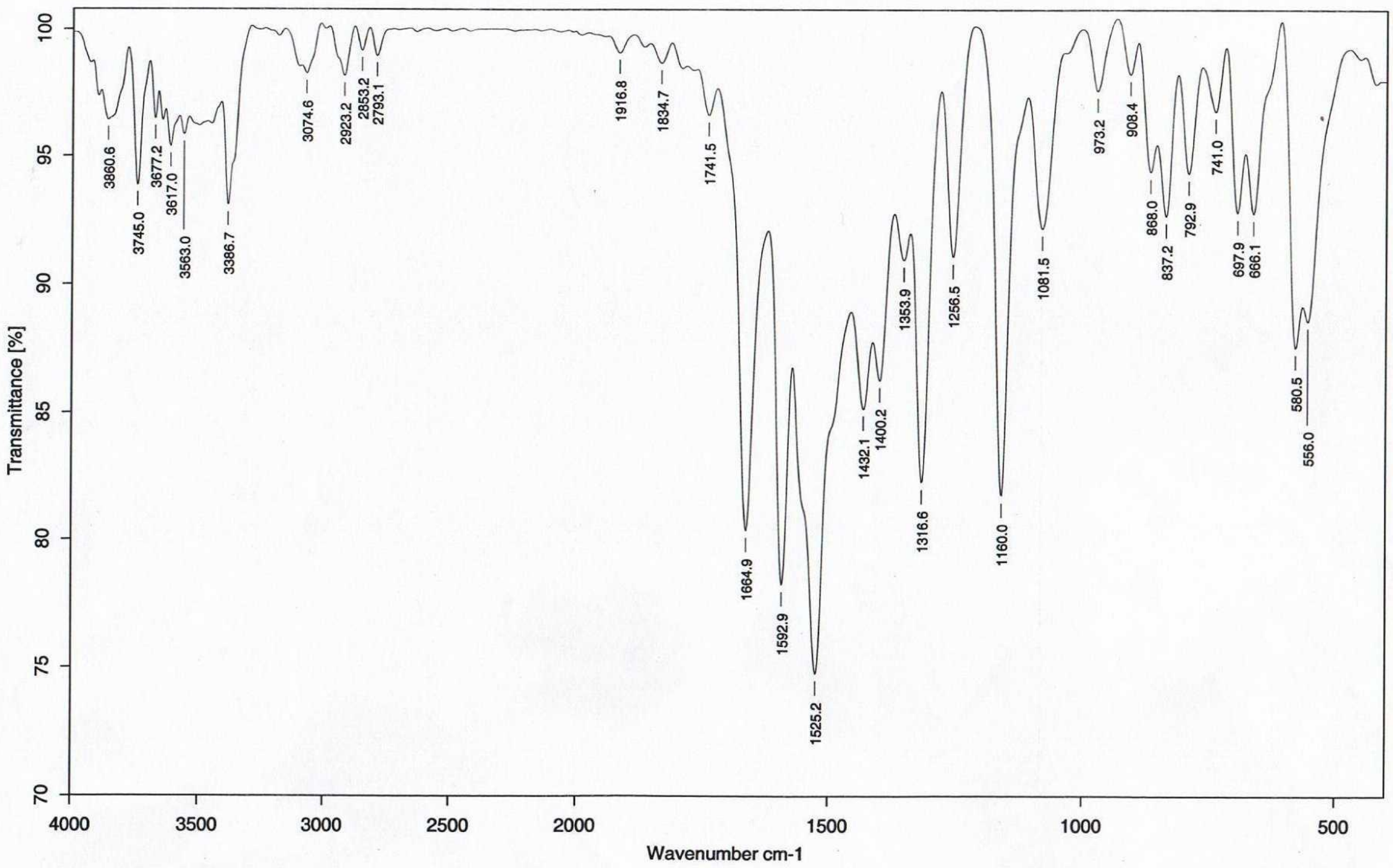

Sample : MHH-1-34/Dr.Haroon/Dr. Hina	Spectrum : MHH-1-34.0 (in D:\IRSTUDENT)
Measured : 02/02/2017 on VECTOR22	Technic : Solid
Resolution : 4 cm-1 (10 scans)	**Analyst : Zubair Ahmad**

HERMO ELECTRON ~ VISIONpro SOFTWARE V4.10

perator Name	ARSHAD ALAM.	Date of Report	2/2/2017
epartment	Analytical Laboratory TWC # 004	Time of Report	3:54:33PM
rganization	ICCBS Karachi of Universty.		
nformation	Dr.Haroon/ Dr.Hina		

can Graph

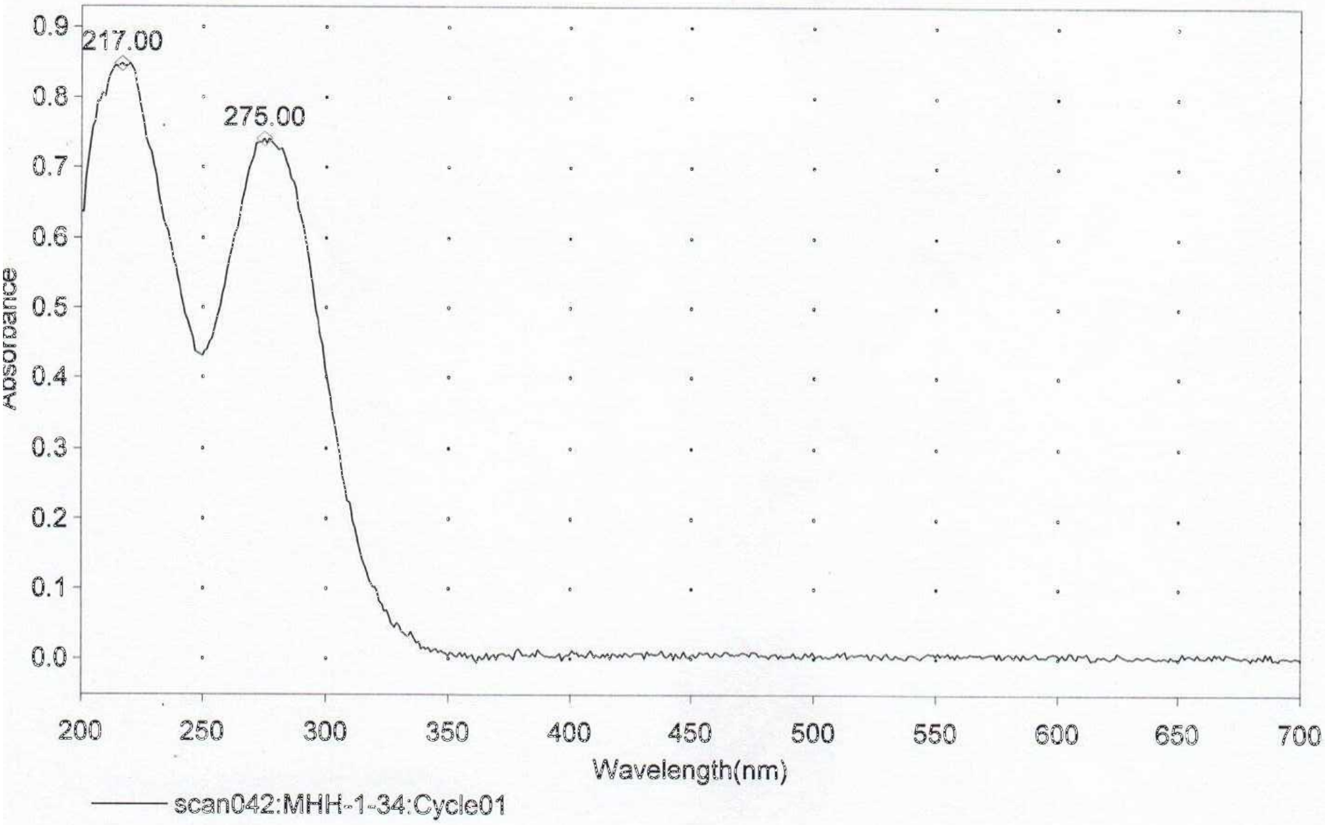

esults Table - MHH-1-34.sre,MHH-1-34,Cycle01

m	A	**Peak Pick Method**
17.00	0.847	Find 8 Peaks Above -3.0000 A
75.00	0.740	Start Wavelength 200.00 nm
		Stop Wavelength 700.00 nm
		Sort By Wavelength

ensitivity Auto
