# Supplementary material for: Sulphamethazine derivatives as immunomodulating agents: New therapeutic strategies for inflammatory diseases
Source: PLoS One. 2018 Dec 19;13(12):e0208933. doi: 10.1371/journal.pone.0208933 (PMC6300282; doi:10.1371/journal.pone.0208933)
Supplement: S33 Fig — (PDF) [file pone.0208933.s033.pdf]

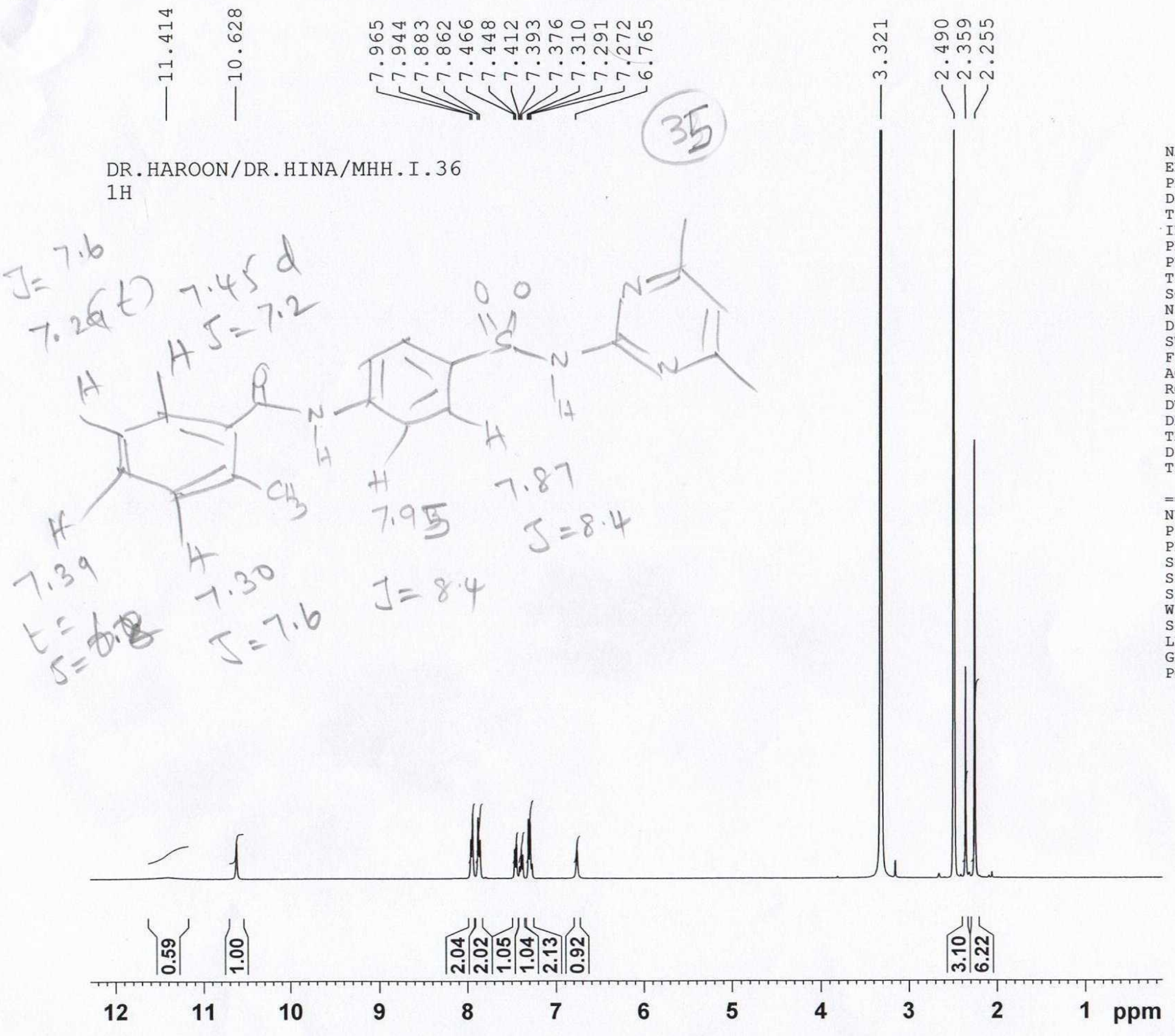

AVANCE AV-400 MHz
Lab # 115

NAME	jan06-17
EXPNO	5
PROCNO	1
Date_	20170106
Time	11.10
INSTRUM	spect
PROBHD	5 mm SEI 1H-13
PULPROG	zg30
TD	65536
SOLVENT	DMSO
NS	64
DS	0
SWH	8012.820 Hz
FIDRES	0.122266 Hz
AQ	4.0894966 sec
RG	512
DW	62.400 usec
DE	6.50 usec
TE	300.0 K
D1	2.00000000 sec
TD0	1

======== CHANNEL f1 ========

NUC1	1H
P1	10.80 usec
PL1	3.00 dB
SFO1	400.0332002 MHz
SI	32768
SF	400.0300041 MHz
WDW	EM
SSB	0
LB	0.30 Hz
GB	0
PC	1.00

DR.HAROON/DR.HINA/MHH.I.36
1H

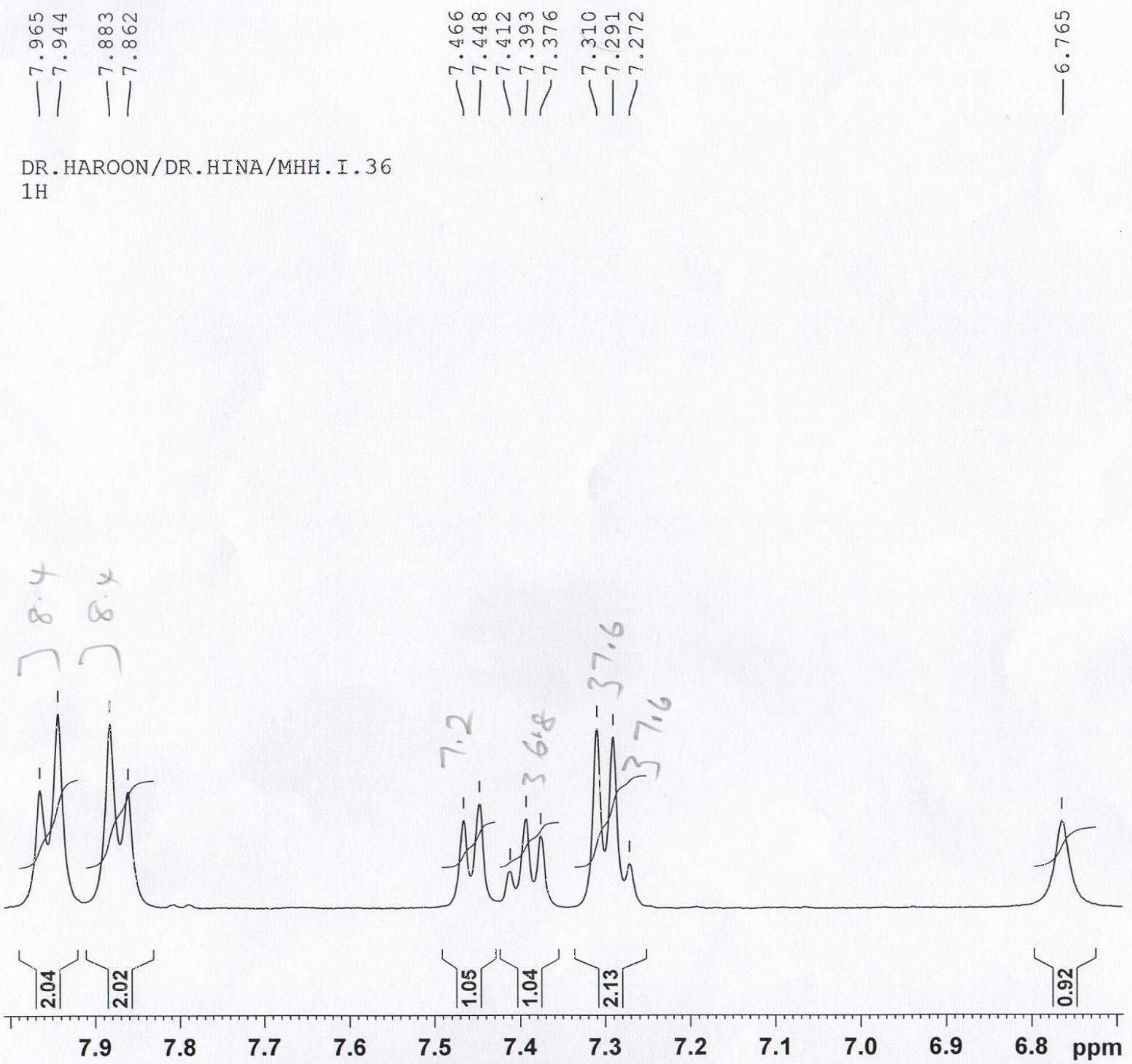

File: MHH-I-36
Sample: DR.M.H.HAROON /DR. IQBAL
Instrument: JEOL MS 600H-1

Date Run: 02-10-2017 (Time Run: 14:29:48)

Ionization mode: EI+

Scan: 22
Base: m/z 119; 99.5%FS TIC: 4703262

R.T.: 1.87

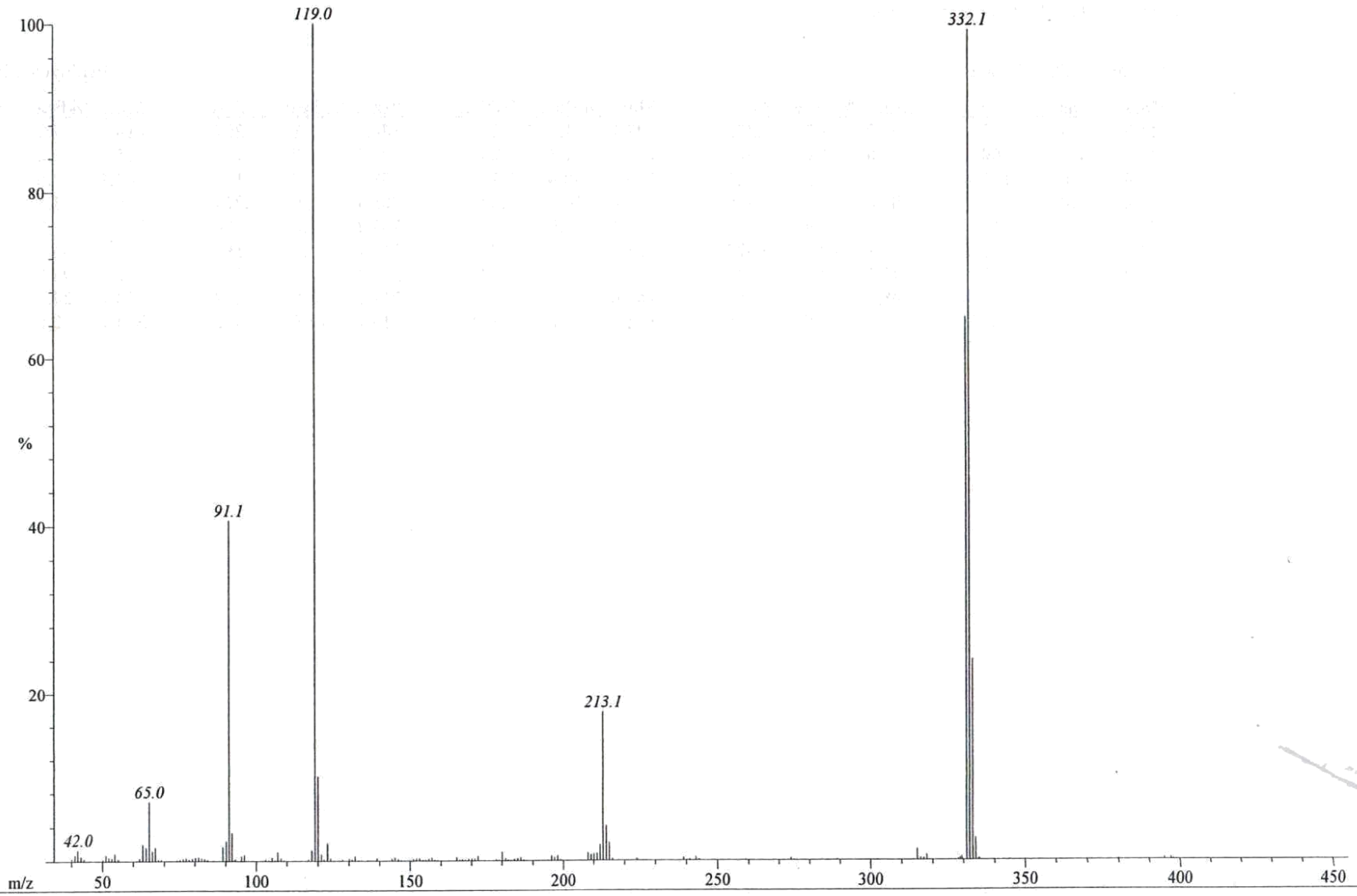

I. C.C.B.S., University of Karachi Analytical Laboratory - Pakistan

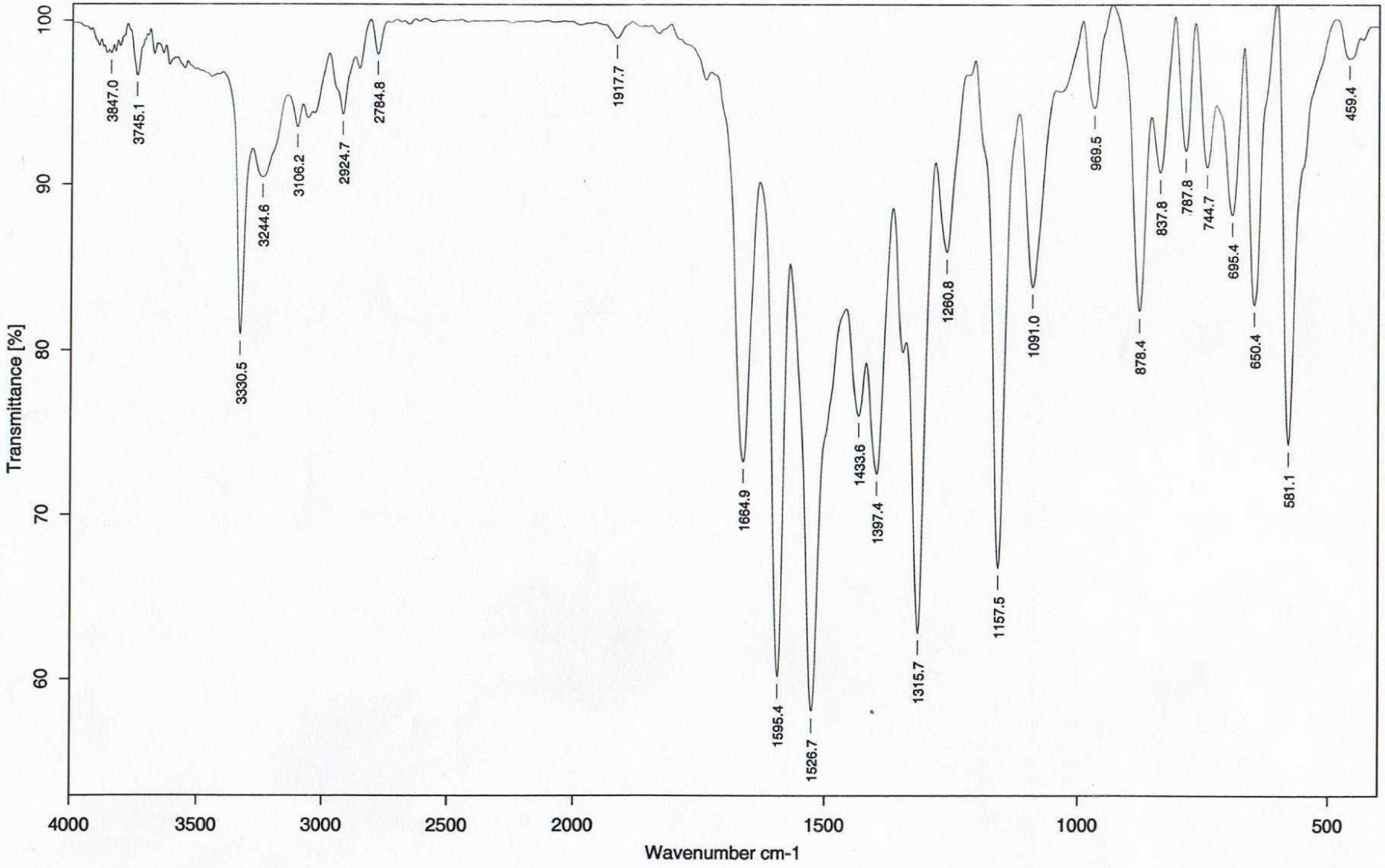

Sample : MHH-1-36/Dr.Haroon	Spectrum : MHH-1-36.0 (in D:\IRSTUDENT)
Measured : 30/01/2017 on VECTOR22	Technic : Solid
Resolution : 4 cm-1 (10 scans)	**Analyst : Zubair Ahmad/ Jamshed**

THERMO ELECTRON ~ VISIONpro SOFTWARE V4.10

Operator Name	ARSHAD ALAM.	Date of Report	1/31/2017
Department	Analytical Laboratory TWC # 004	Time of Report	10:14:56AM
Organization	ICCBS Karachi of Universty.		
Information	Dr Haroon/ Dr Hina		

Scan Graph

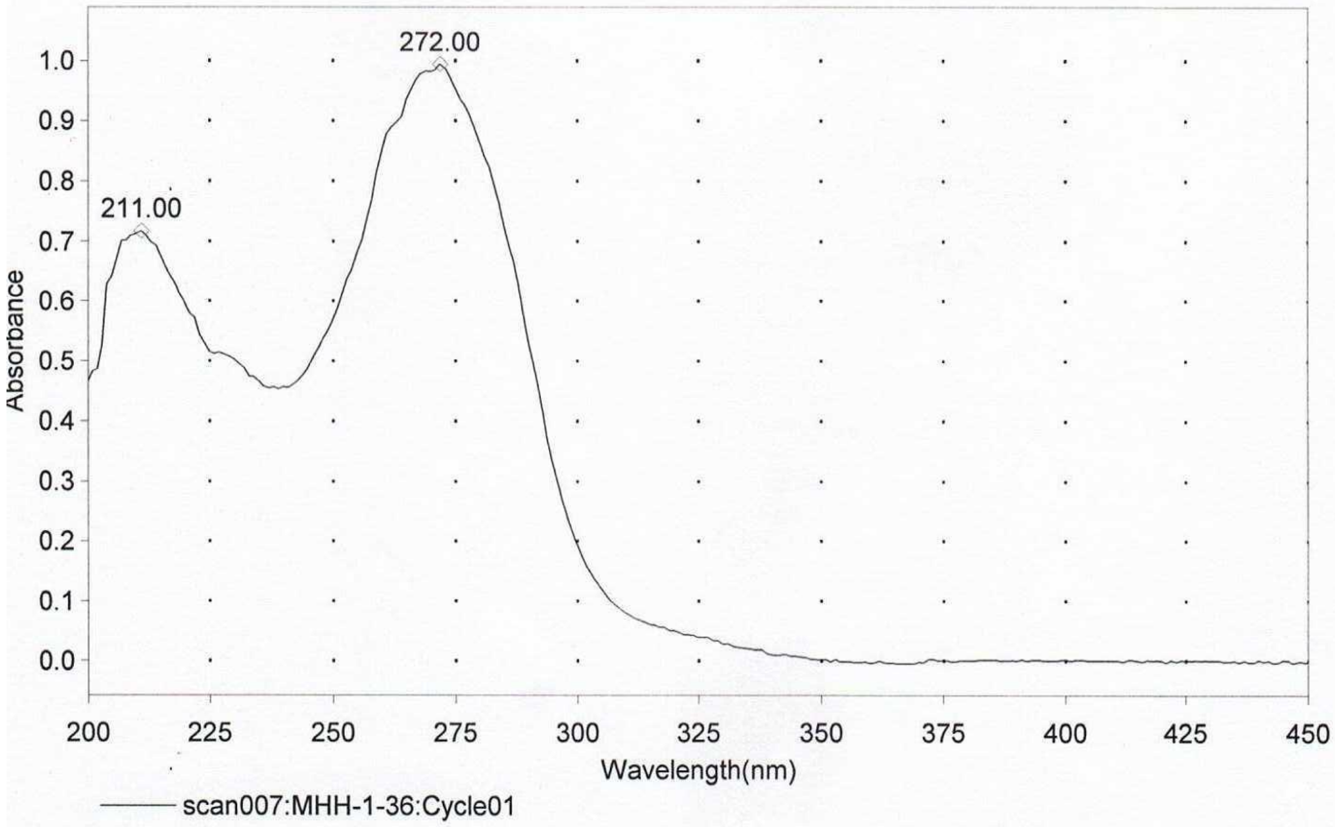

Results Table - MHH-1-36.sre,MHH-1-36,Cycle01

nm	A	**Peak Pick Method**
211.00	0.718	Find 8 Peaks Above -3.0000 A
272.00	0.994	Start Wavelength 200.00 nm
		Stop Wavelength 450.00 nm
		Sort By Wavelength

Sensitivity Auto
