# Supplementary material for: Sulphamethazine derivatives as immunomodulating agents: New therapeutic strategies for inflammatory diseases
Source: PLoS One. 2018 Dec 19;13(12):e0208933. doi: 10.1371/journal.pone.0208933 (PMC6300282; doi:10.1371/journal.pone.0208933)
Supplement: S35 Fig — (PDF) [file pone.0208933.s035.pdf]

HAROON/DR,HINA/MHH-I-43/
ICCBS,U.O.K/

AF (37)

AVANCE AV - III
300 MHz, LAB # 116

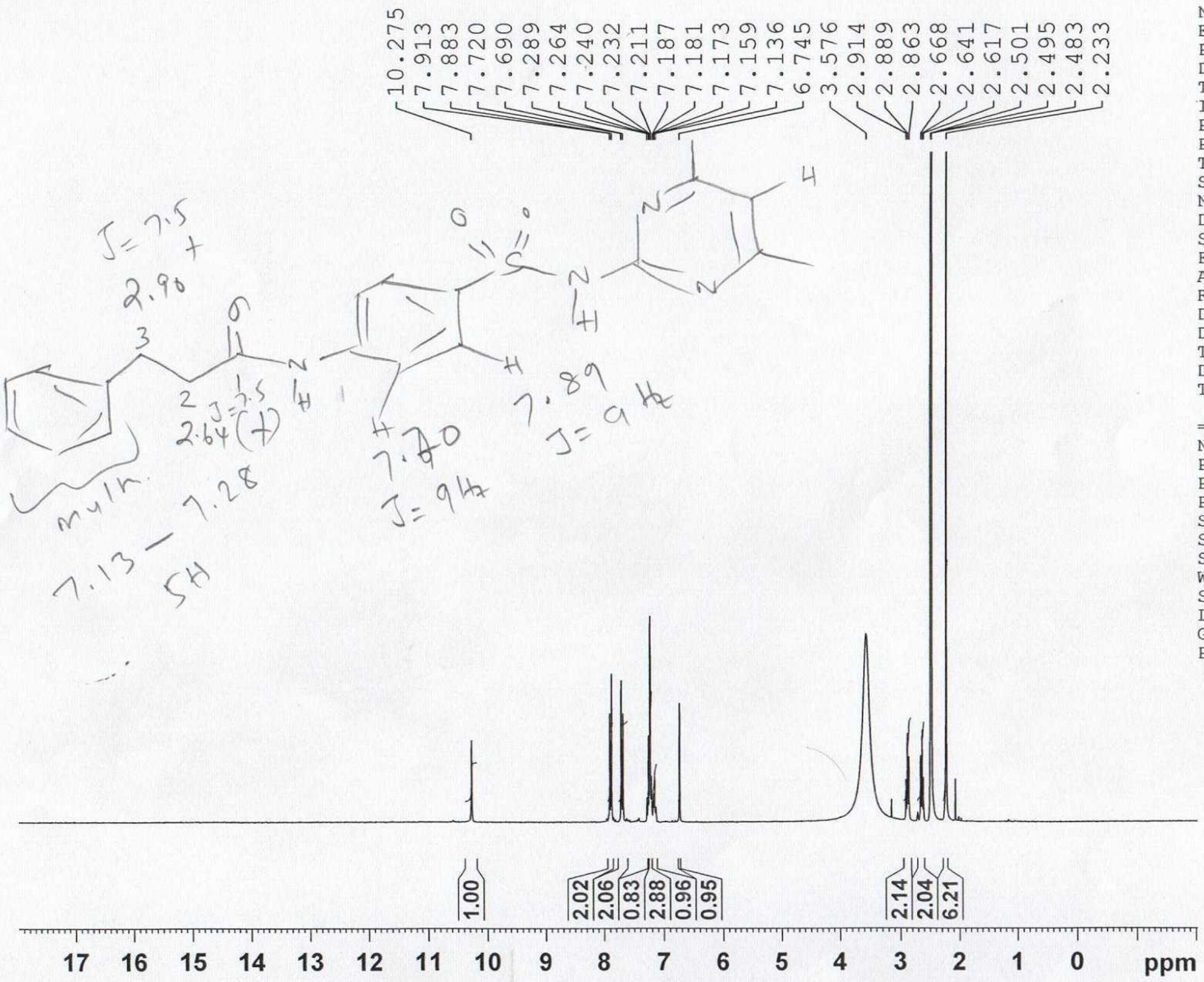

NAME	jan23-17
EXPNO	9
PROCNO	1
Date_	20170123
Time	14.37
INSTRUM	Spect
PROBHD	5 mm BBO BB-1H
PULPROG	zg30
TD	32768
SOLVENT	DMSO
NS	64
DS	0
SWH	6009.615 Hz
FIDRES	0.183399 Hz
AQ	2.7263477 sec
RG	203
DW	83.200 usec
DE	6.50 usec
TE	300.0 K
D1	1.50000000 sec
TD0	1

======== CHANNEL f1 ========

NUC1	1H
P1	12.50 usec
PL1	0.00 dB
PL1W	13.16228485 W
SFO1	300.1324010 MHz
SI	16384
SF	300.1300040 MHz
WDW	EM
SSB	0
LB	0.30 Hz
GB	0
PC	1.00

HAROON/DR,HINA/MHH-I-43/
ICCBS,U.O.K/

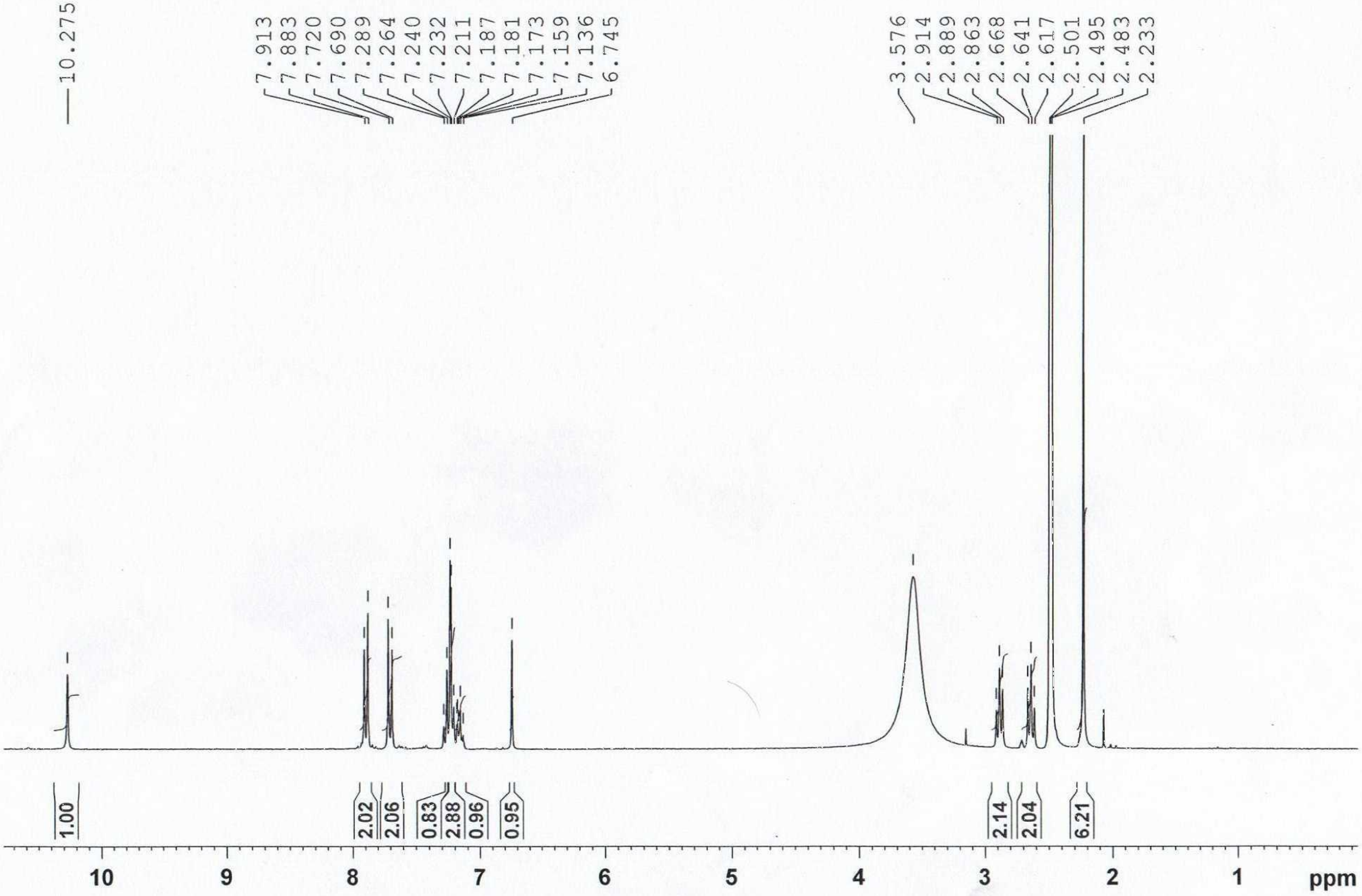

HAROON/DR,HINA/MHH-I-43/
ICCBS,U.O.K/

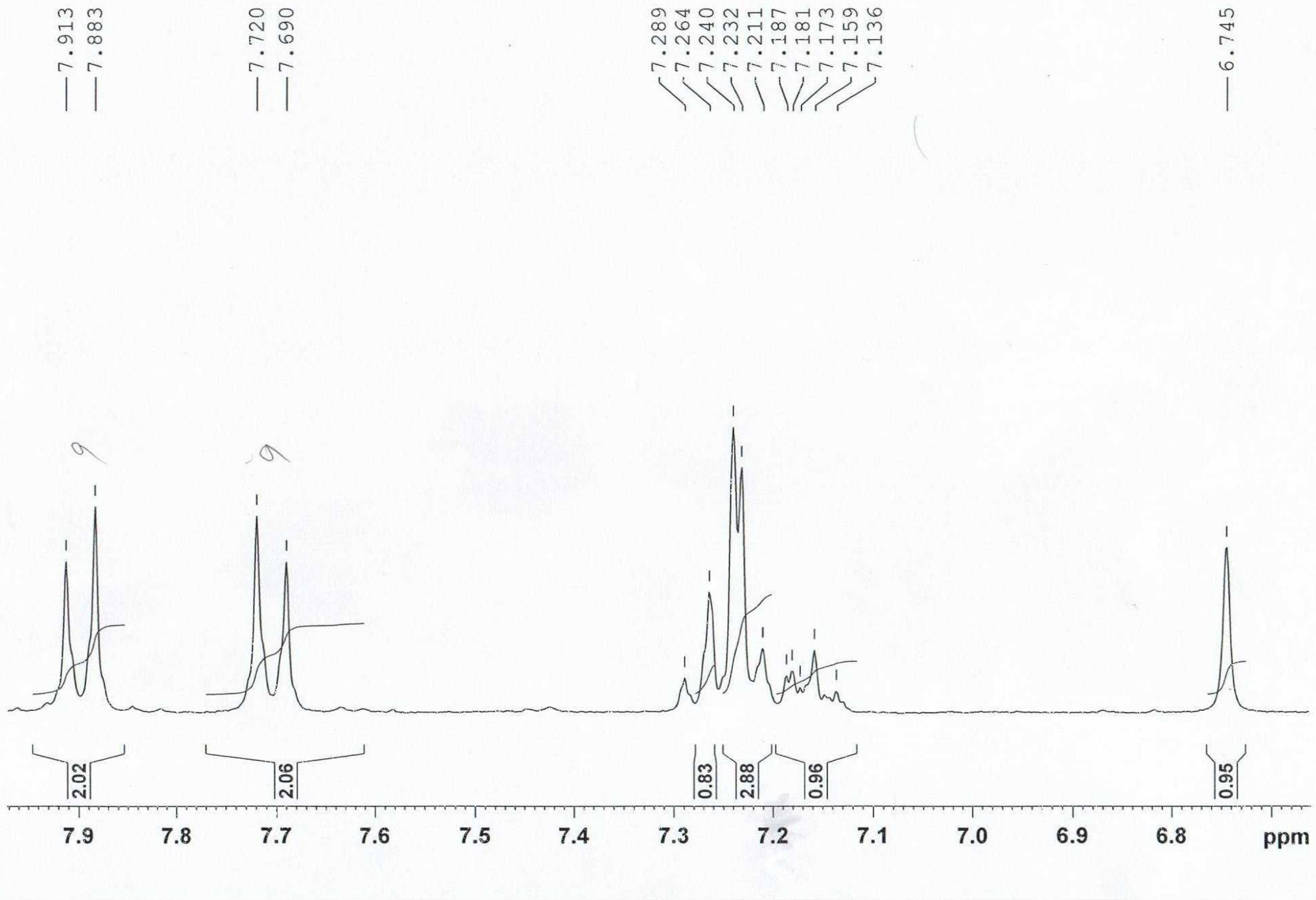

HAROON/DR,HINA/MHH-I-43/
ICCBS,U.O.K/

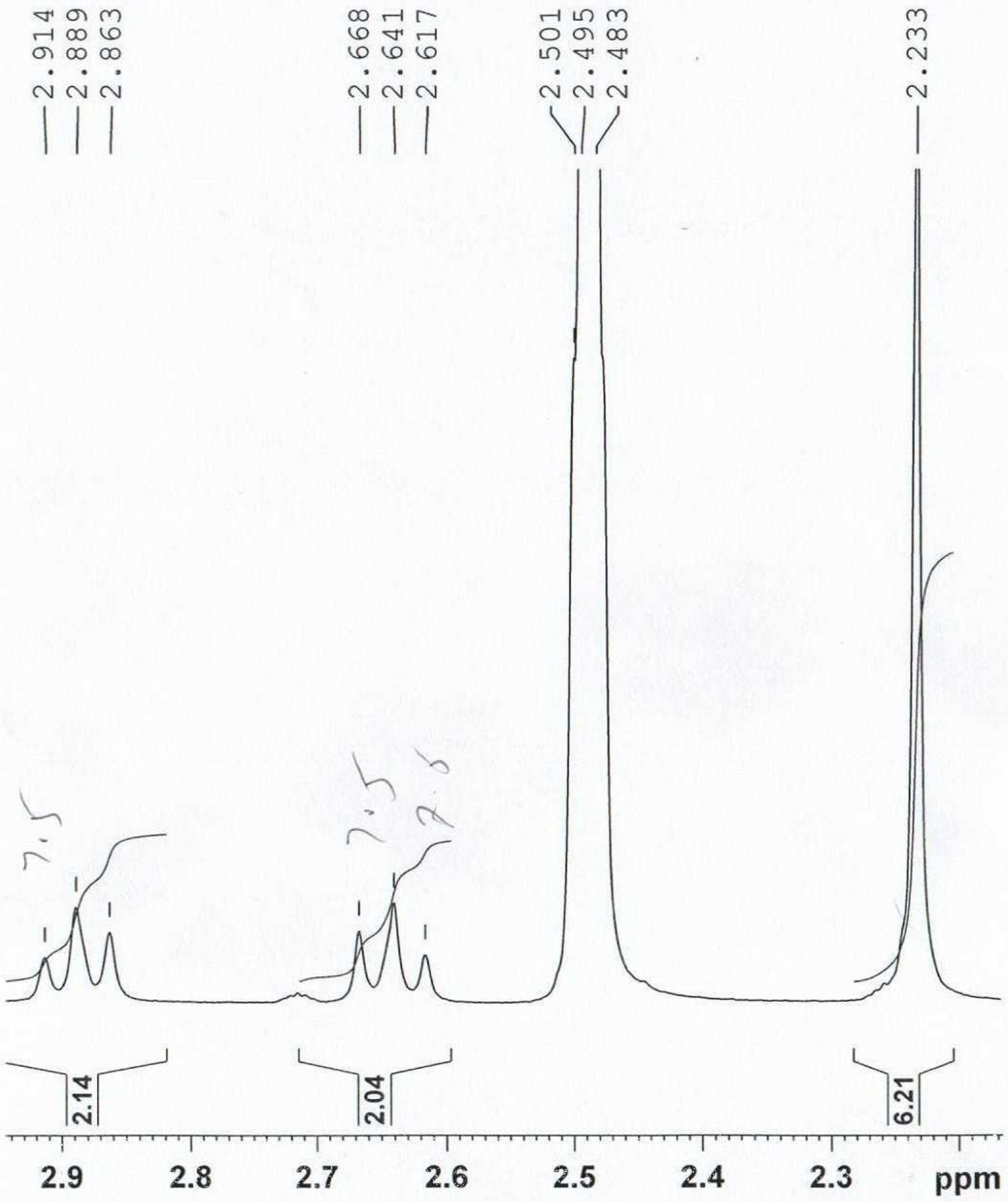

File: MHH-I-43
Sample: DR.M.H.HAROON /DR. HINA
Instrument: JEOL MS 600H-1

Date Run: 02-13-2017 (Time Run: 12:08:52)

Ionization mode: EI+

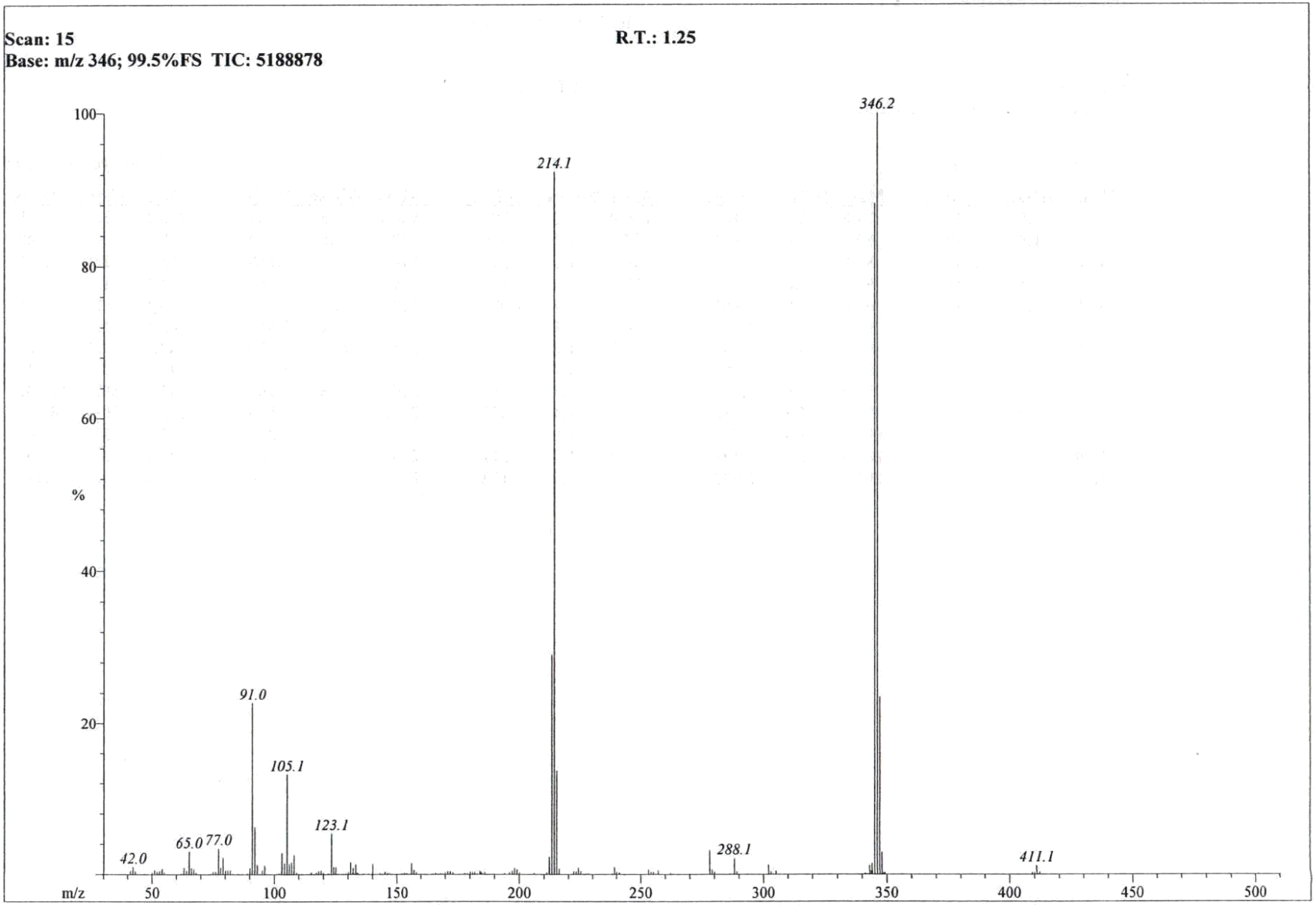

I. C.C.B.S., University of Karachi Analytical Laboratory - Pakistan

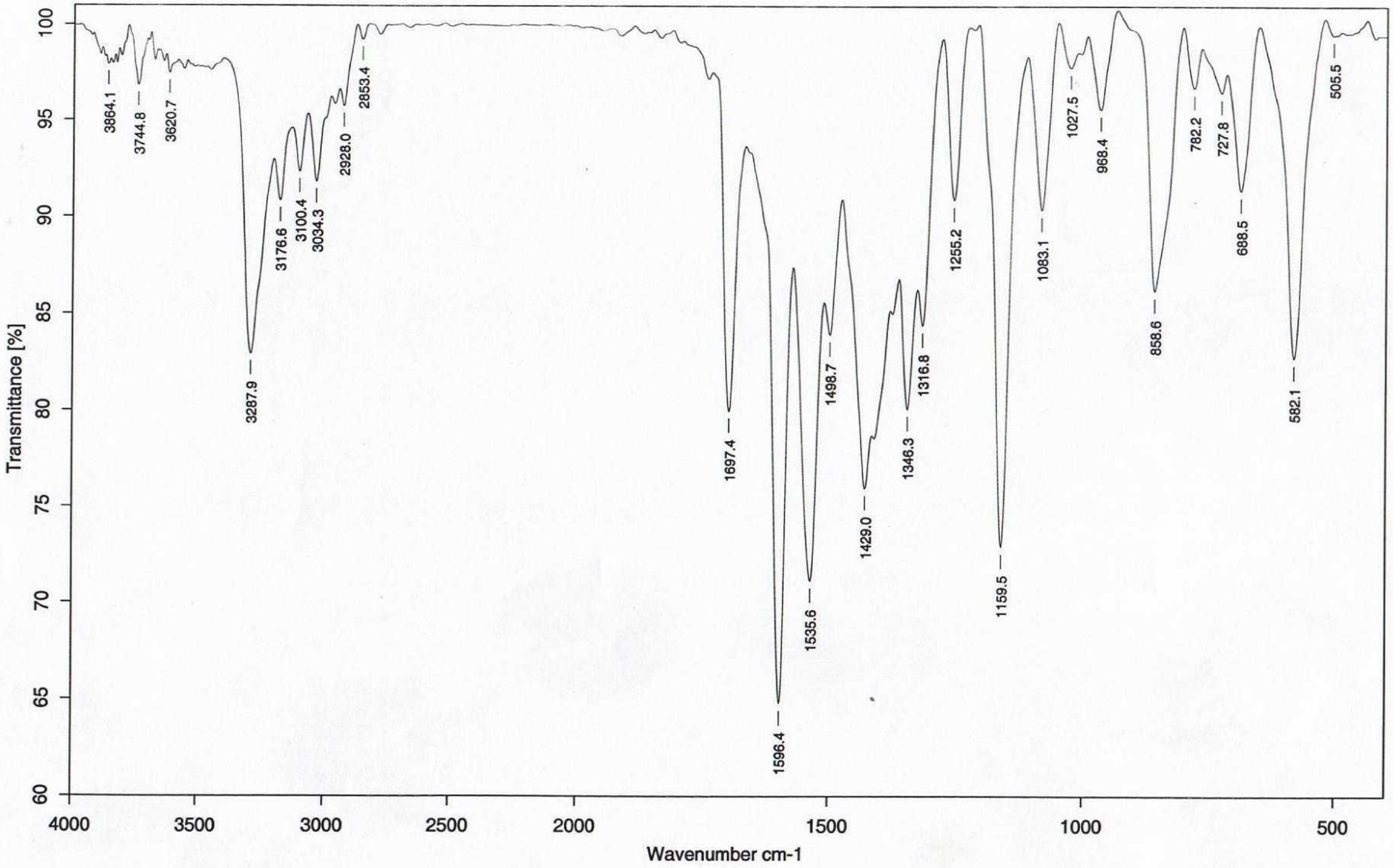

Sample : MHH-1-43/Dr.Haroon

Measured : 30/01/2017 on VECTOR22

Resolution : 4 cm-1 (10 scans)

Spectrum : MHH-1-43.0 (in D:\IRSTUDENT)

Technic : Solid

Analyst : Zubair Ahmad/ Jamshed

THERMO ELECTRON ~ VISIONpro SOFTWARE V4.10

Operator Name	ARSHAD ALAM.	Date of Report	1/31/2017
Department	Analytical Laboratory TWC # 004	Time of Report	10:09:53AM
Organization	ICCBS Karachi of Universty.		
Information	Dr Haroon/ Dr Hina		

Scan Graph

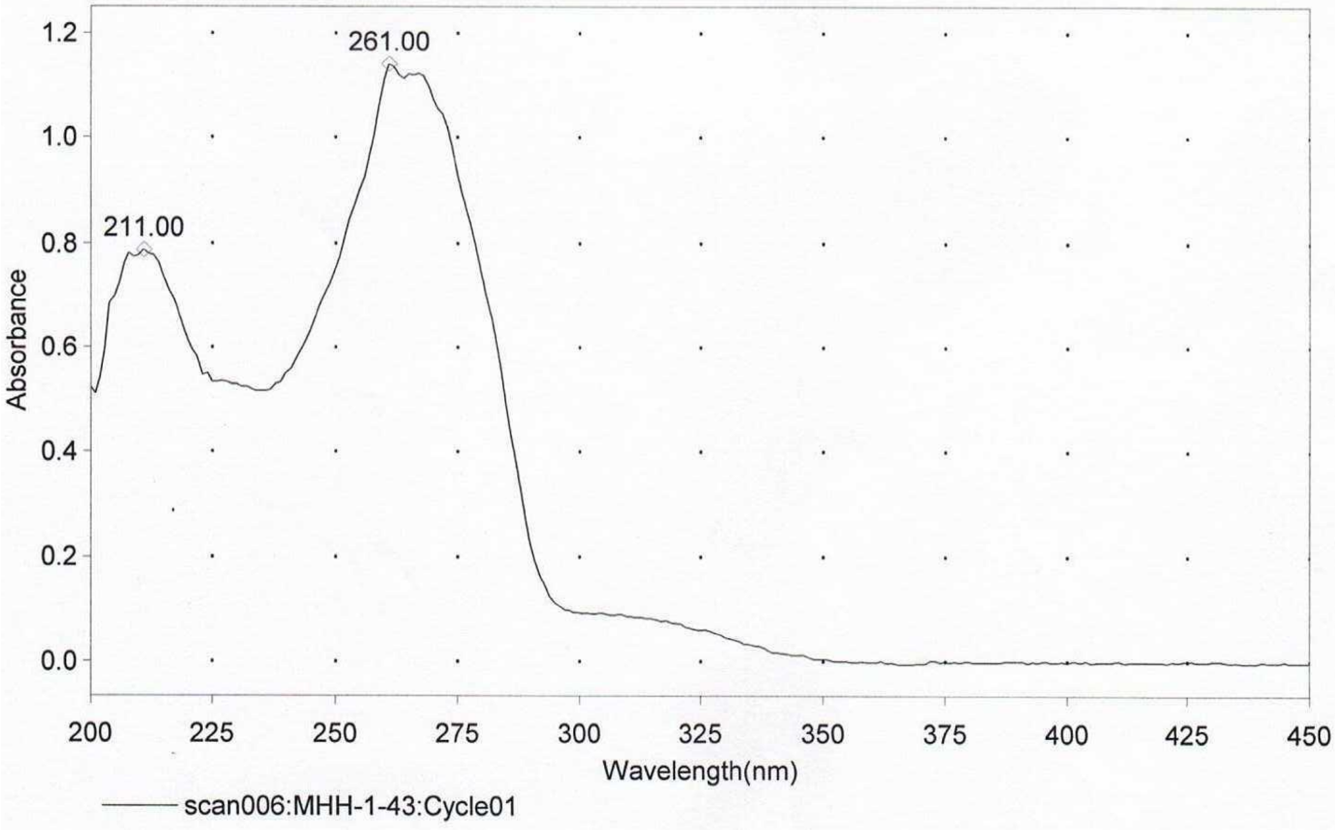

Results Table - MHH-1-43.sre,MHH-1-43,Cycle01

nm	A	**Peak Pick Method**
211.00	0.787	Find 8 Peaks Above -3.0000 A
261.00	1.141	Start Wavelength 200.00 nm
		Stop Wavelength 450.00 nm
		Sort By Wavelength

Sensitivity Auto
