# Supplementary material for: Sulphamethazine derivatives as immunomodulating agents: New therapeutic strategies for inflammatory diseases
Source: PLoS One. 2018 Dec 19;13(12):e0208933. doi: 10.1371/journal.pone.0208933 (PMC6300282; doi:10.1371/journal.pone.0208933)
Supplement: S36 Fig — (PDF) [file pone.0208933.s036.pdf]

AVANCE AV-400 MHz
Lab # 115

DR.HAROON/DR.HINA/MHH.I.48
1H

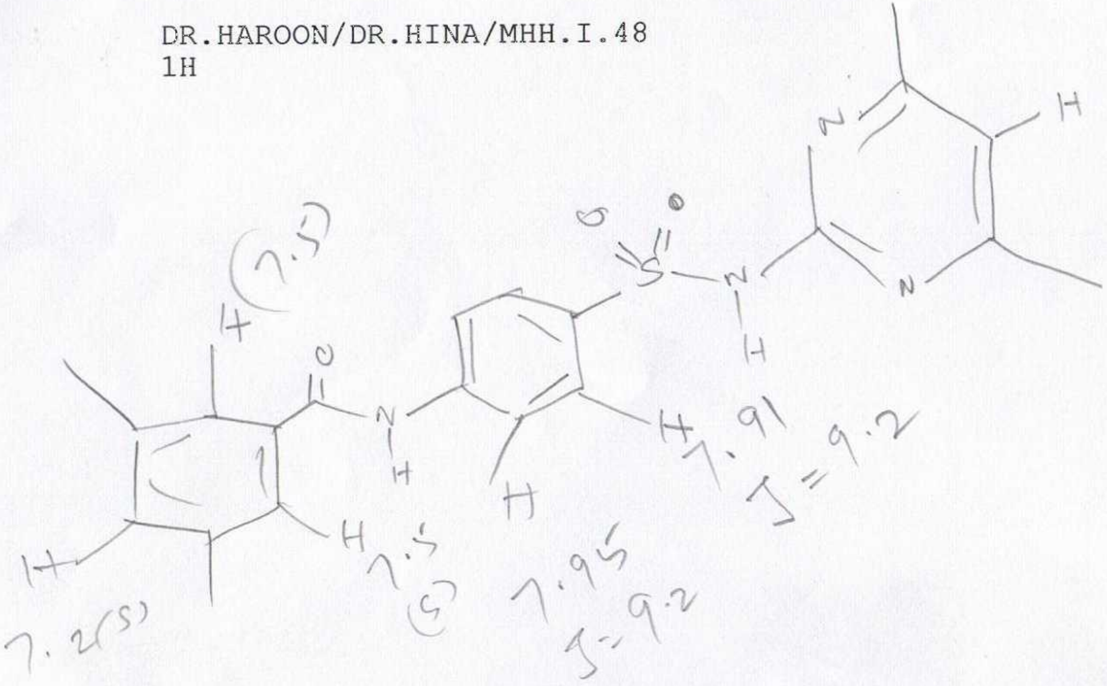

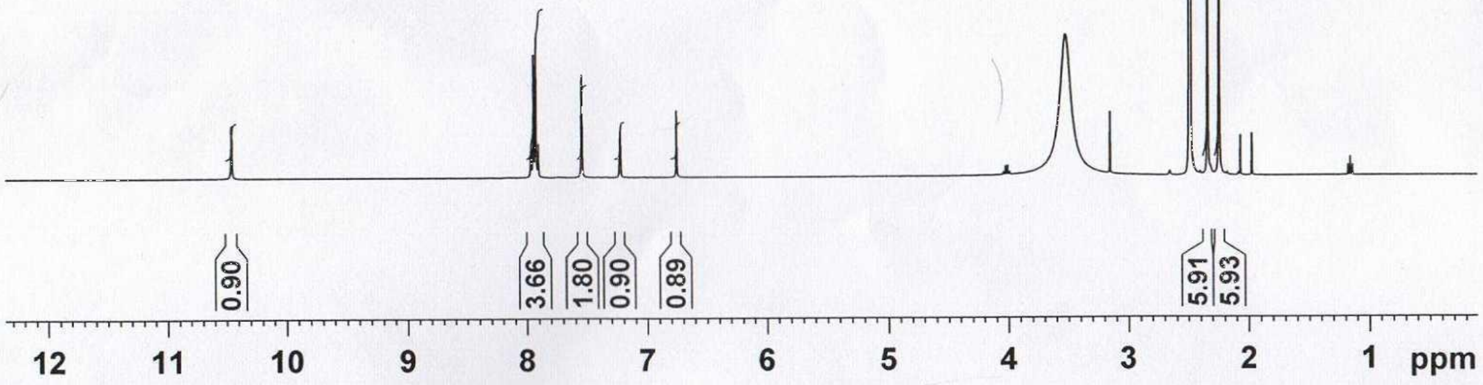

NAME	march02-17
EXPNO	2
PROCNO	1
Date_	20170302
Time	10.36
INSTRUM	spect
PROBHD	5 mm SEI 1H-13
PULPROG	zg30
TD	32768
SOLVENT	DMSO
NS	64
DS	0
SWH	8012.820 Hz
FIDRES	0.244532 Hz
AQ	2.0447731 sec
RG	362
DW	62.400 usec
DE	6.50 usec
TE	300.0 K
D1	2.00000000 sec
TD0	1

======== CHANNEL f1 ========

NUC1	1H
P1	10.63 usec
PL1	2.00 dB
SFO1	400.0332002 MHz
SI	16384
SF	400.0300041 MHz
WDW	EM
SSB	0
LB	0.30 Hz
GB	0
PC	1.00

DR.HAROON/DR.HINA/MHH.I.48
1H

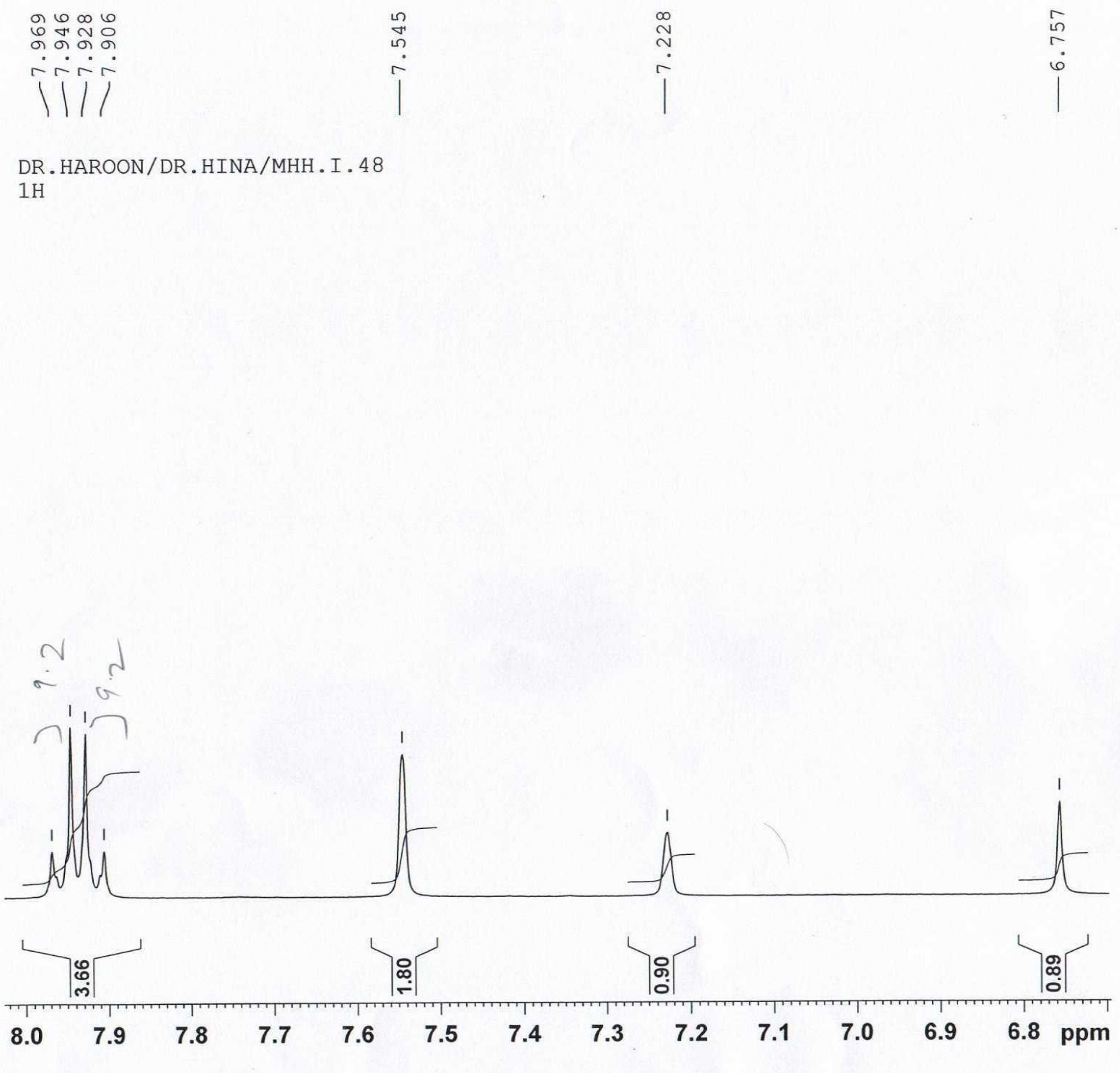

File: MHH-I-48-FABP
Sample: DR.M.H.HAROON /DR. HINA
Instrument: JEOL-600H-2
Inlet: Direct Probe

Date Run: 03-15-2017 (Time Run: 08:48:44)

Ionization mode: FAB+

Scan: 9
Base: m/z 185; 24.9%FS TIC: 1318368

R.T.: .72

#Ions: 928

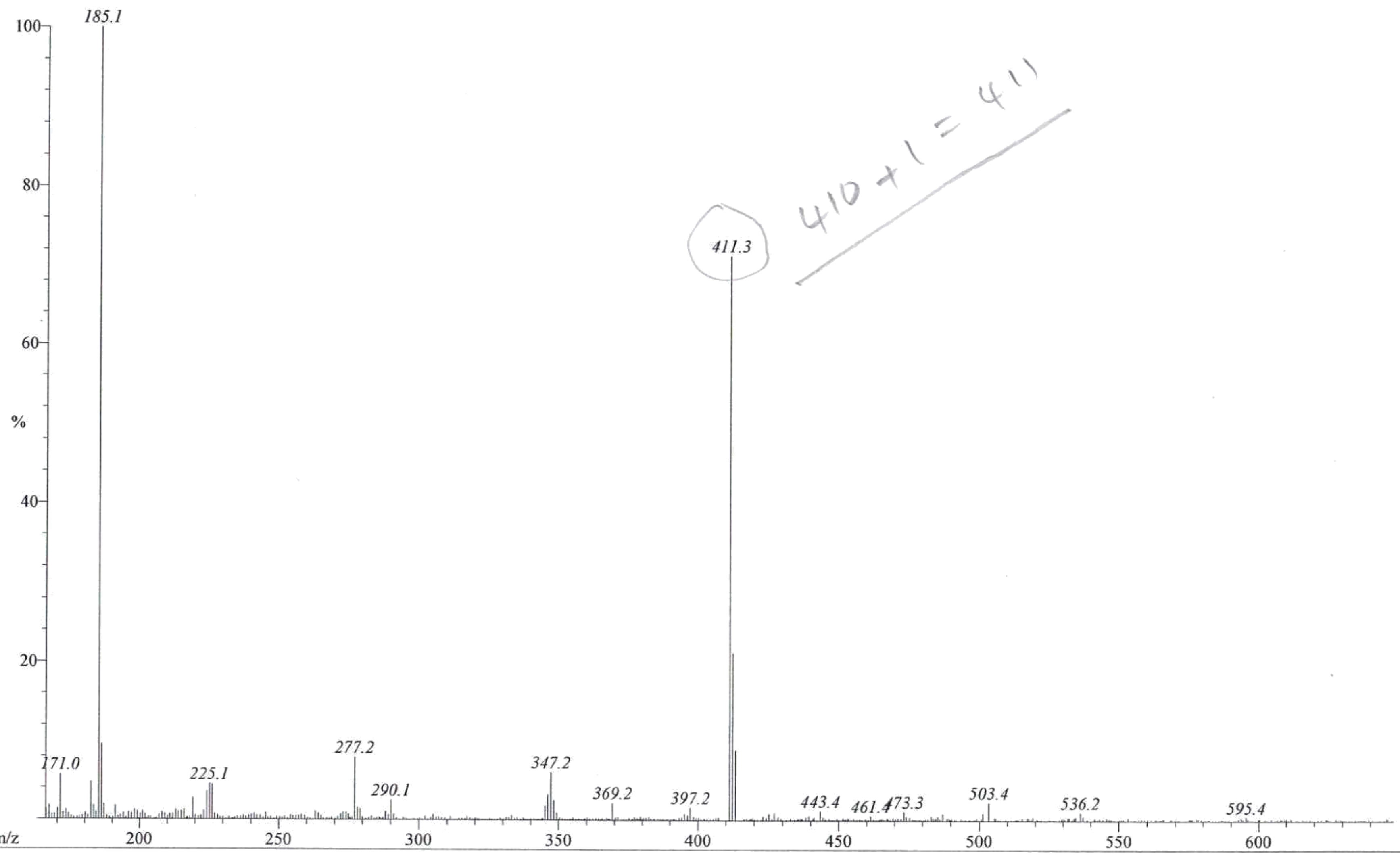

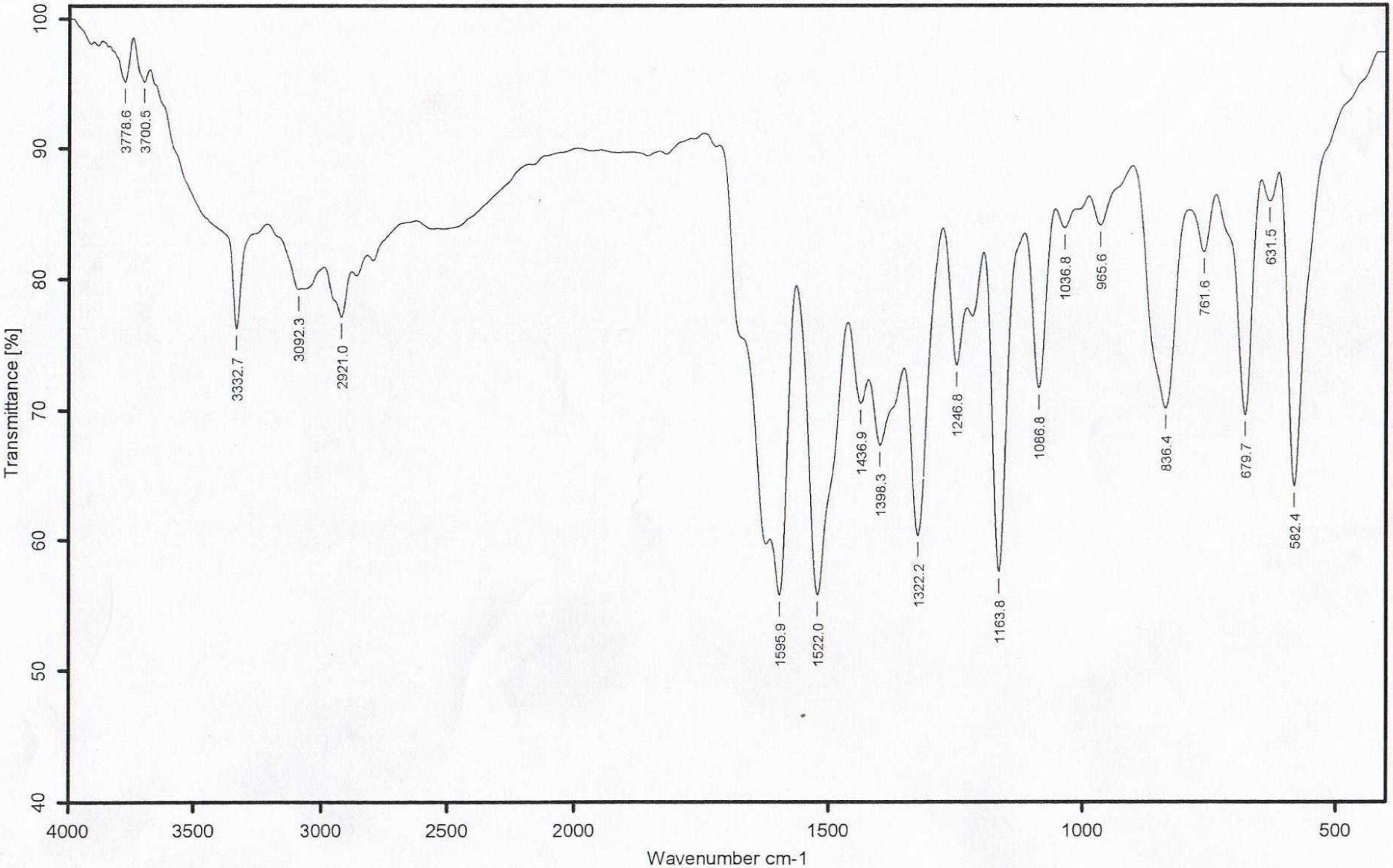

Sample : MHH-I-48/Dr. Haroon/Dr. Hina

Measured : 05/07/2017 on VECTOR22

Resolution : 4 cm-1 (10 scans)

Spectrum : MHH-I-48.0 (in D:\IRSTUDENT)

Technic : Solid

Analyst : MA/ZA

HERMO ELECTRON ~ VISIONpro SOFTWARE V4.10

perator Name	ARSHAD ALAM	Date of Report	7/5/2017
epartment	Analytical Laboratory TWC # 004	Time of Report	10:46:42AM
rganization	ICCBS Karachi of Universty.		
formation	DR. Haroon/ Dr. Hina.		

can Graph

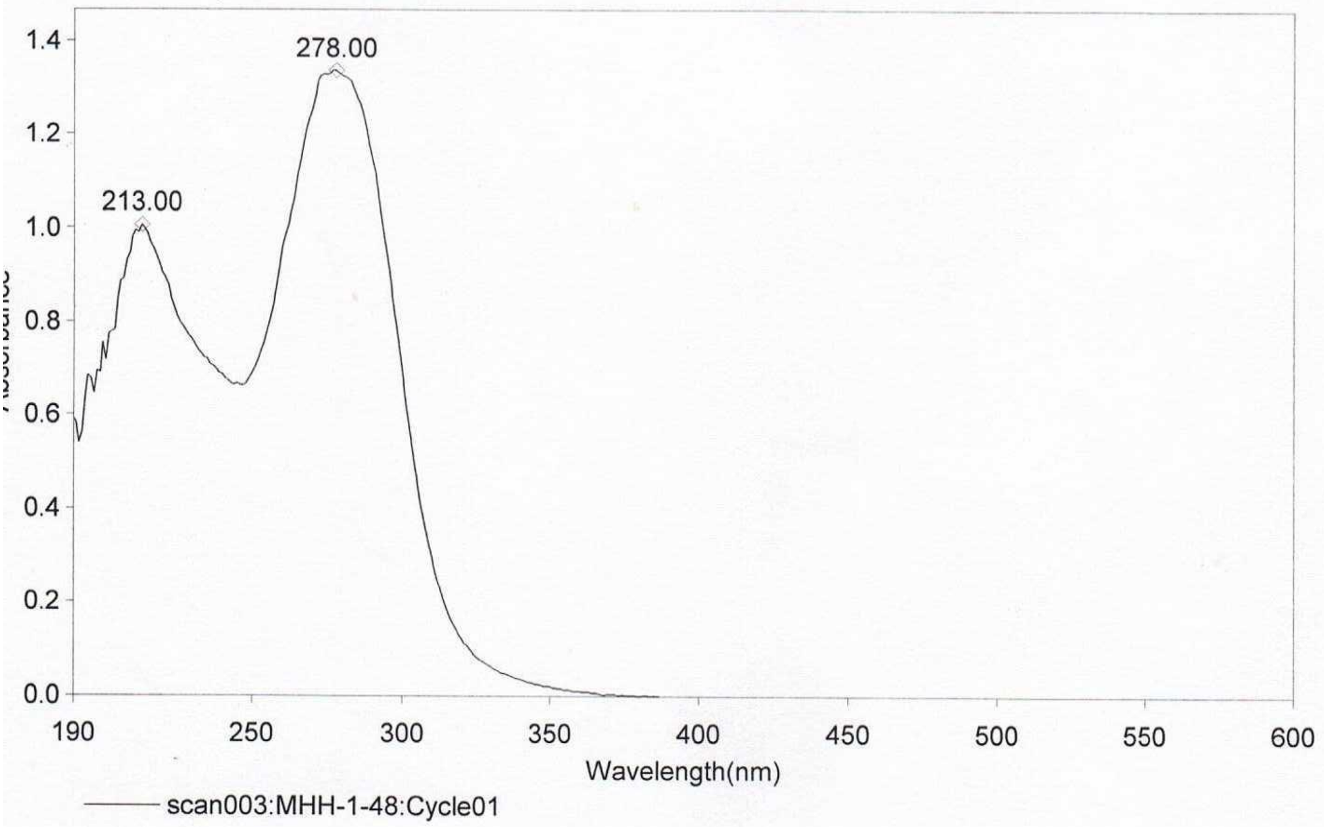

esults Table - MHH-1-48.sre,MHH-1-48,Cycle01

n	A	**Peak Pick Method**
13.00	1.006	Find 8 Peaks Above -3.0000 A
78.00	1.337	Start Wavelength 190.00 nm
		Stop Wavelength 600.00 nm
		Sort By Wavelength

ensitivity Auto
