# Supplementary material for: Sulphamethazine derivatives as immunomodulating agents: New therapeutic strategies for inflammatory diseases
Source: PLoS One. 2018 Dec 19;13(12):e0208933. doi: 10.1371/journal.pone.0208933 (PMC6300282; doi:10.1371/journal.pone.0208933)
Supplement: S37 Fig — (PDF) [file pone.0208933.s037.pdf]

AVANCE AV-400 MHz
Lab # 115

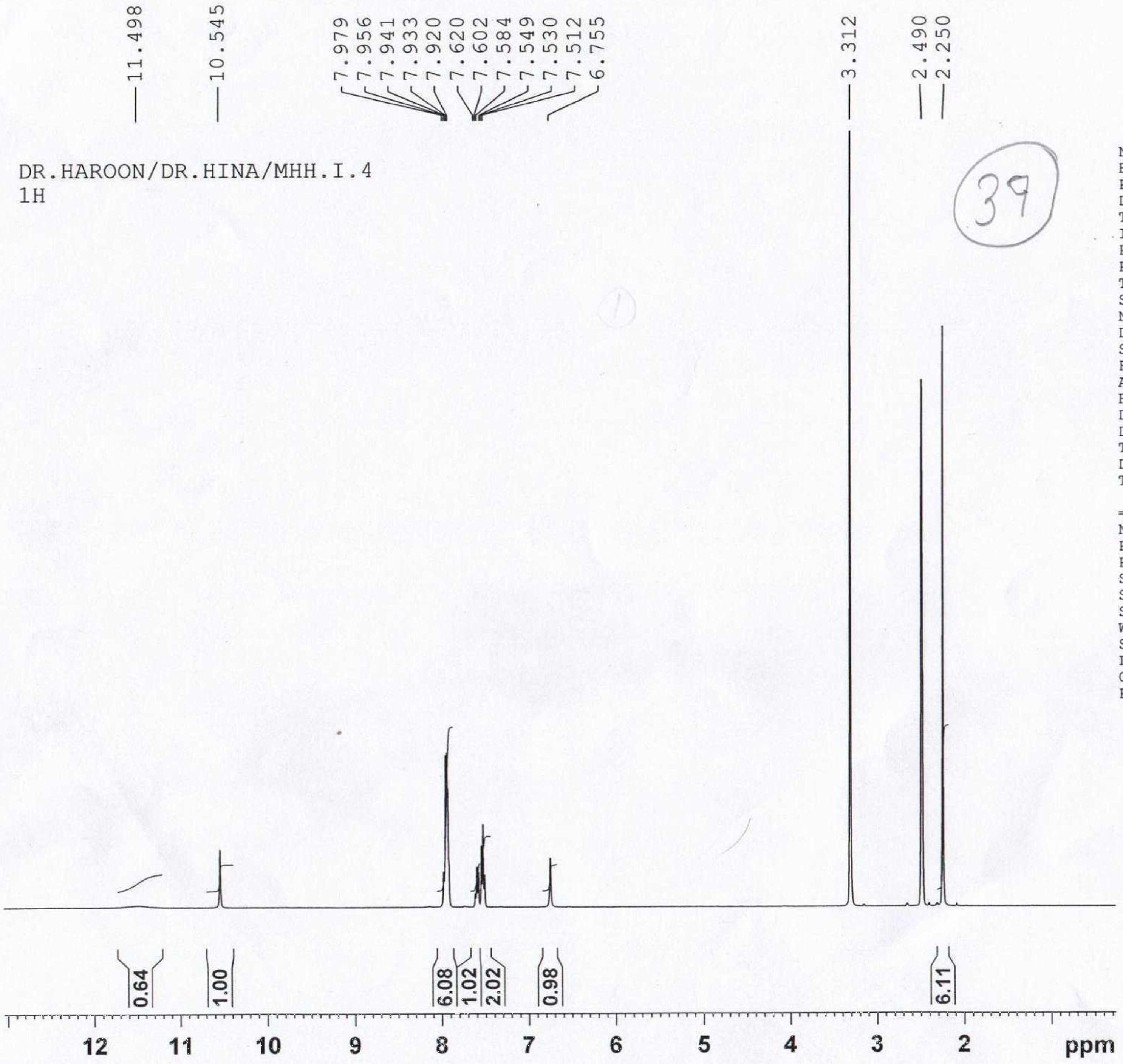

NAME	dec26-16
EXPNO	2
PROCNO	1
Date_	20161226
Time	10.50
INSTRUM	spect
PROBHD	5 mm SEI 1H-13
PULPROG	zg30
TD	65536
SOLVENT	DMSO
NS	64
DS	0
SWH	8012.820 Hz
FIDRES	0.122266 Hz
AQ	4.0894966 sec
RG	512
DW	62.400 usec
DE	6.50 usec
TE	300.0 K
D1	1.50000000 sec
TD0	1

======== CHANNEL f1 ========

NUC1	1H
P1	10.80 usec
PL1	3.00 dB
SFO1	400.0332002 MHz
SI	32768
SF	400.0300041 MHz
WDW	EM
SSB	0
LB	0.30 Hz
GB	0
PC	1.00

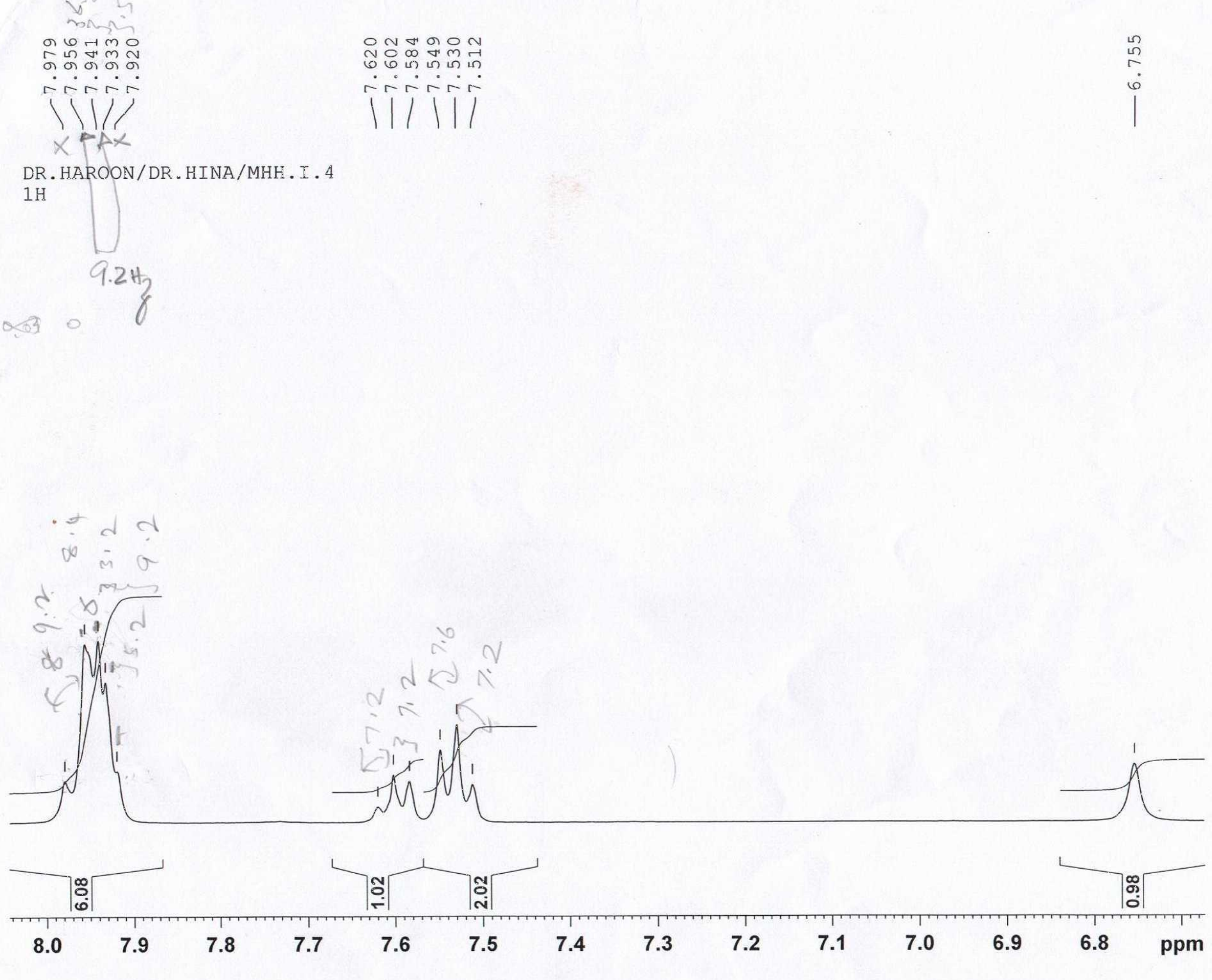
7.979
7.956
7.941
7.933
7.920
7.620
7.602
7.584
7.549
7.530
7.512
6.755
DR.HAROON/DR.HINA/MHH.I.4
1H
6.08
1.02
2.02
0.98
8.0
7.9
7.8
7.7
7.6
7.5
7.4
7.3
7.2
7.1
7.0
6.9
6.8
ppm

HEJ-ICCBS

12/2/2016 4:24:22 PM

File: MHH-I-4- **Date Run: 12-02-2016 (Time Run: 16:10:15)**

Sample: DR. M.H. HAROON /DR. HINA

Instrument: JEOL MS 600H-1

Ionization mode: EI+

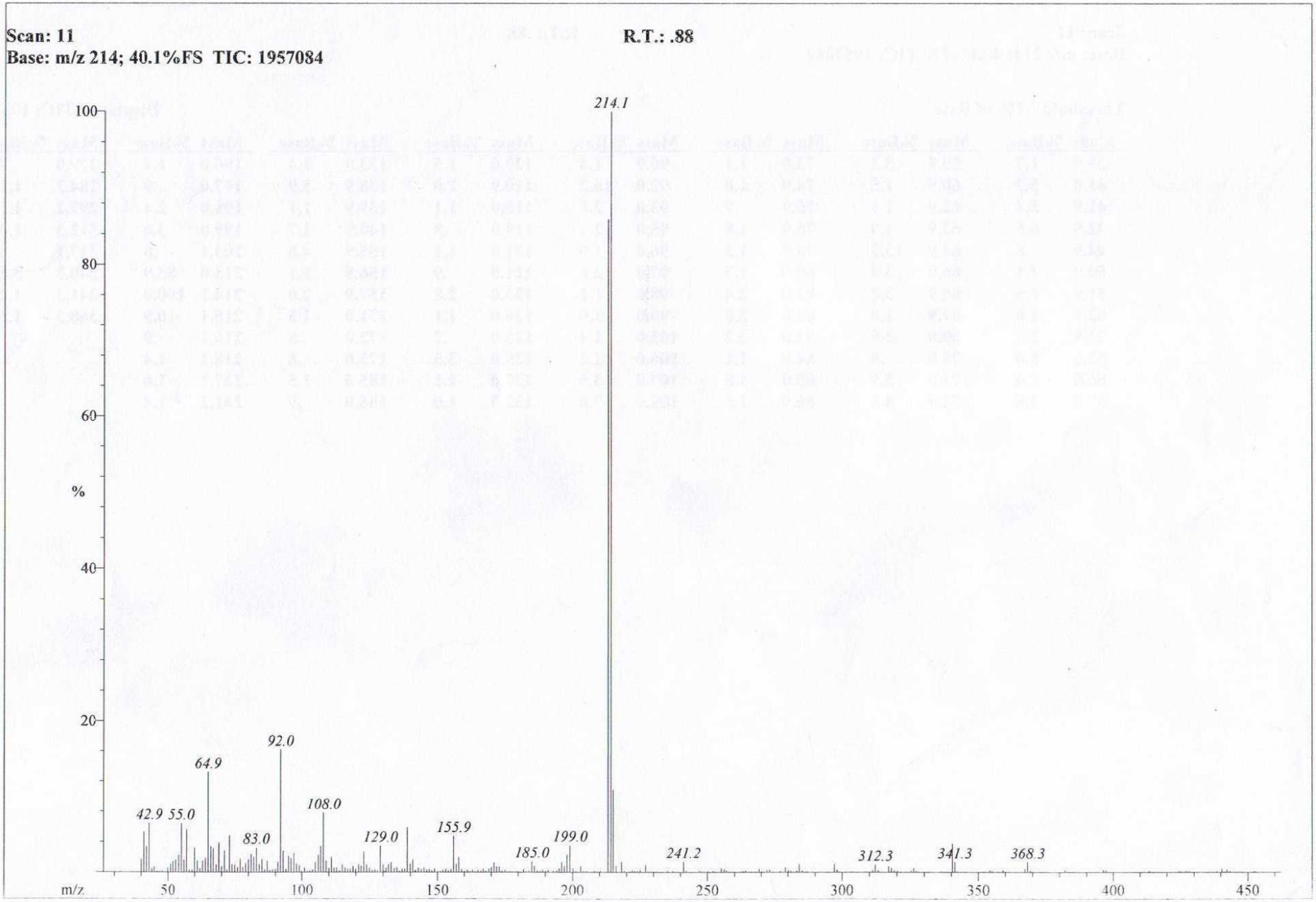

I. C.C.B.S., University of Karachi Analytical Laboratory - Pakistan

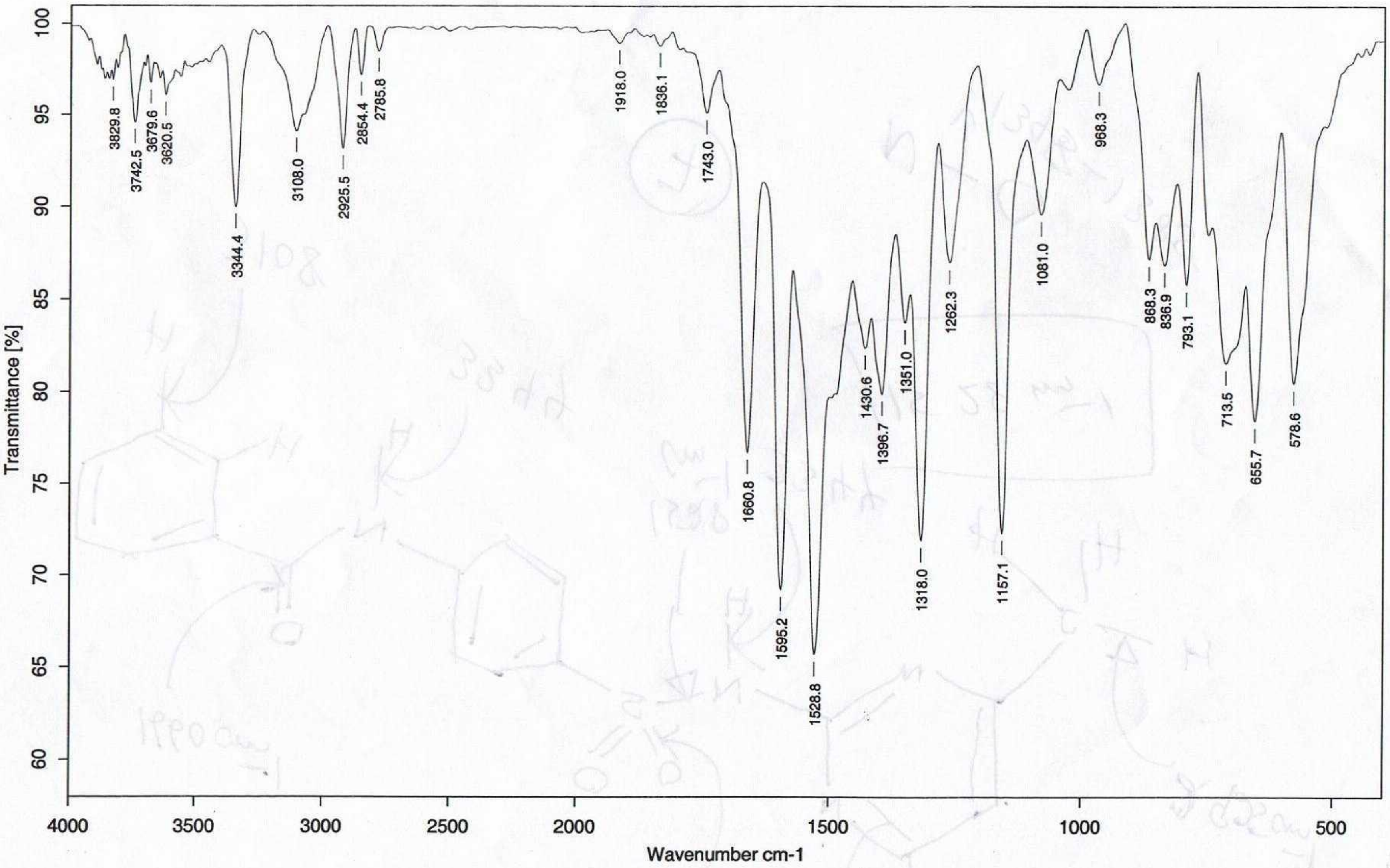

Sample : MHH-1-4/Haroon/Dr. Hina

Measured : 31/01/2017 on VECTOR22

Resolution : 4 cm-1 (10 scans)

Spectrum : MHH-1-4.0 (in D:\IRSTUDENT)

Technic : Solid

Analyst : Zubair Ahmad/ Jamshed/M. Asif/

ꓕHERMO ELECTRON ~ VISIONpro SOFTWARE V4.10

)perator Name	ARSHAD ALAM.	Date of Report	2/1/2017
)epartment	Analytical Laboratory TWC # 004	Time of Report	3:16:20PM
)rganization	ICCBS Karachi of Universty.		
nformation	Dr Haroon/Dr Hina		

;can Graph

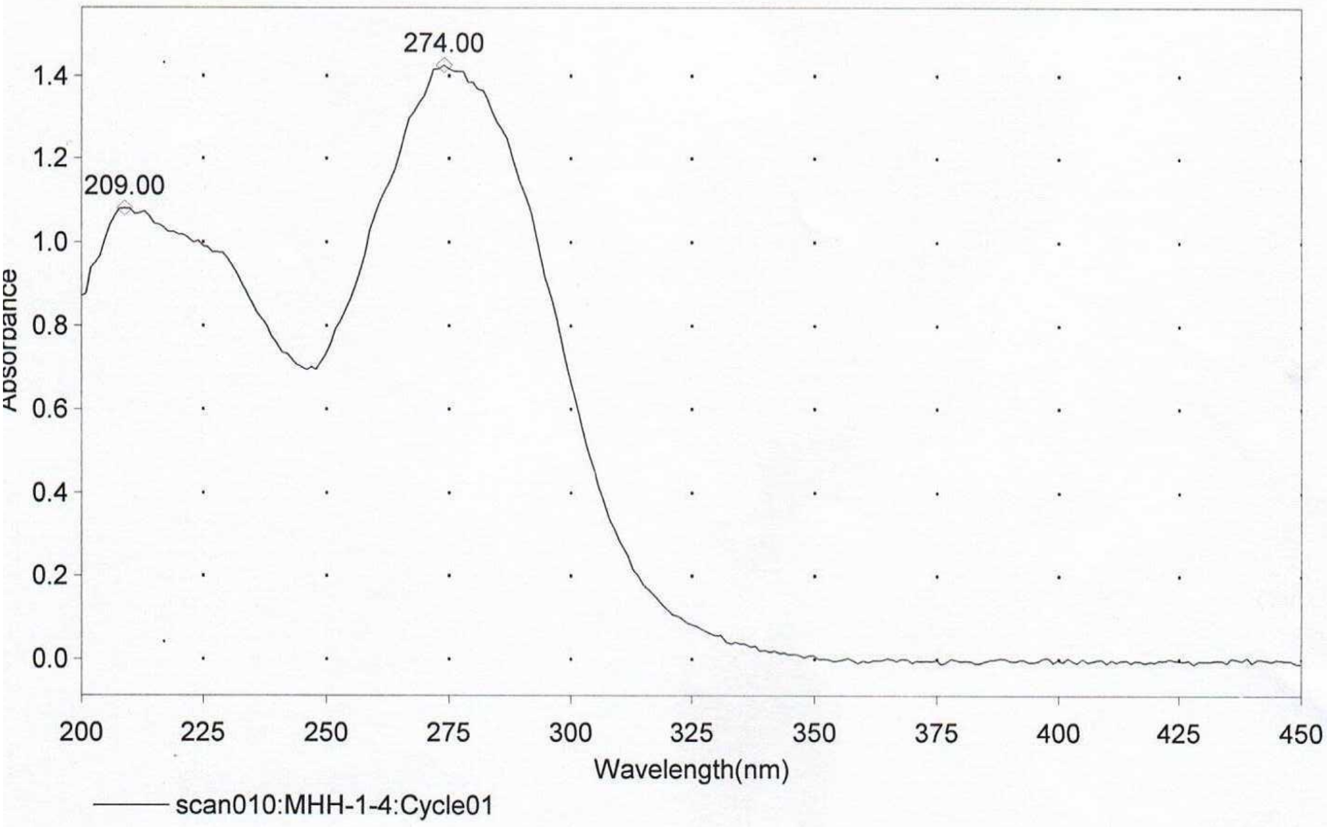

ₐesults Table - MHH-1-4.sre,MHH-1-4,Cycle01

ım	A	**Peak Pick Method**
:09.00	1.080	Find 8 Peaks Above -3.0000 A
:74.00	1.426	Start Wavelength 200.00 nm
		Stop Wavelength 450.00 nm
		Sort By Wavelength

;ensitivity Auto
