# Supplementary material for: Sulphamethazine derivatives as immunomodulating agents: New therapeutic strategies for inflammatory diseases
Source: PLoS One. 2018 Dec 19;13(12):e0208933. doi: 10.1371/journal.pone.0208933 (PMC6300282; doi:10.1371/journal.pone.0208933)
Supplement: S38 Fig — (PDF) [file pone.0208933.s038.pdf]

INDEPENDENT ETHICS COMMITTEE
INTERNATIONAL CENTER FOR CHEMICAL AND BIOLOGICAL SCIENCES

Date: April 13, 2015

Dr. Almas Jabeen,
Dr. Panjwani Center for Molecular Medicine and Drug Research,
International Center for Chemical & Biological Sciences

Subject: **IEC Approval for the Protocol ICCBS/IEC-008-BC-2015/Protocol/1.0**

IEC, ICCBS has gone through the submitted protocol; having following particulars:

Title: Screening of natural / synthetic products for immunomodulatory activity on human blood and isolated cells
Study #: -008-BC-2015
Protocol #: ICCBS/IEC--008-BC-2015/Protocol/1.0
Version #: 1.0
Version date: 13th April 2015
Principal Investigator: Dr. Almas Jabeen
Study Site: Dr. Panjwani Center for Molecular Medicine and Drug Research, International Center for Chemical & Biological Sciences

The members of the committee approved the protocol without having any reservations and we are pleased to inform you that approval for blood sample collection has been granted.

Regards,

Chairperson
Independent Ethics Committee,
ICCBS
University of Karachi

IEC (ICCBS) Approved
